# Axial shielding of Pd(II) complexes enables perfect stereoretention in Suzuki-Miyaura cross-coupling of Csp^3^ boronic acids

**DOI:** 10.1038/s41467-019-09249-z

**Published:** 2019-03-20

**Authors:** Jonathan W. Lehmann, Ian T. Crouch, Daniel J. Blair, Melanie Trobe, Pulin Wang, Junqi Li, Martin D. Burke

**Affiliations:** 1 Department of Chemistry, University of Illinois at Urbana-Champaign, 454 Roger Adams Laboratory, 600S Mathews Avenue, Urbana, 61801 IL USA; 2 Carle Illinois College of Medicine, Institute for Genomic Biology, Beckman Institute, and Department of Biochemistry, University of Illinois at Urbana-Champaign, Urbana, 61801 IL USA

## Abstract

Stereocontrolled Csp^3^ cross-coupling can fundamentally change the types of chemical structures that can be mined for molecular functions. Although considerable progress in achieving the targeted chemical reactivity has been made, controlling stereochemistry in Csp^3^ cross-coupling remains challenging. Here we report that ligand-based axial shielding of Pd(II) complexes enables Suzuki-Miyaura cross-coupling of unactivated Csp^3^ boronic acids with perfect stereoretention. This approach leverages key differences in spatial orientation between competing pathways for stereoretentive and stereoinvertive transmetalation of Csp^3^ boronic acids to Pd(II). We show that axial shielding enables perfectly stereoretentive cross-coupling with a range of unactivated secondary Csp^3^ boronic acids, as well as the stereocontrolled synthesis of xylarinic acid B and all of its Csp^3^ stereoisomers. We expect these ligand design principles will broadly enable the continued search for practical and effective methods for stereospecific Csp^3^ cross-coupling.

## Abstract (web summary)

Despite the progress in C(sp^3^) cross-coupling reactions, full control over the stereochemistry remains a challenge. Here, the authors show that phosphine-containing axially shielded Pd(II) complexes enable Suzuki-Miyaura cross-couplings of unactivated C(sp^3^) boronic acids with perfect stereoretention.

## Introduction

The synthesis of stereochemically complex Csp^3^-rich small molecules remains a rate-limiting factor in the discovery and development of next generation medicines, agrochemicals, biological probes, materials, and many other types of societally impactful compounds^1,2^. Suzuki-Miyaura cross-coupling has profoundly enabled progress with Csp^2^-rich molecules in all of these areas. Expanding this methodology to include stereocontrolled Csp^3^ cross-coupling could fundamentally change the types of molecular solutions that can be practically developed^1–3^.

**Fig. 1:**
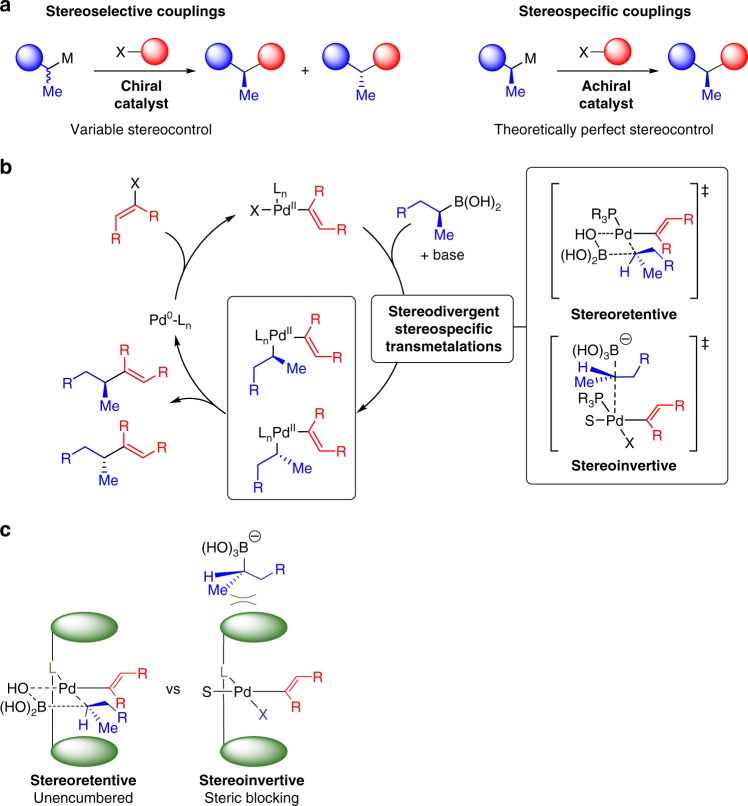
Challenges in stereocontrolled Csp^3^ couplings. **a** Stereoselective couplings vs. stereospecific couplings. **b** Competing stereodivergent stereospecific transmetalation pathways. **c** Axial shielding—a ligand design principle for controlling stereochemistry in Csp^3^ couplings. Projecting steric bulk above and below the Pd (II) square plane should selectively inhibit stereoinvertive transmetalation, thus favoring stereoretention

Stereochemical control in Csp^3^ cross-coupling can be pursued via either stereoselective or stereospecific pathways^4^. In stereoselective Csp^3^ cross-coupling, stereocontrol typically relies on subtle interactions between chiral catalysts and chiral substrates in stereoconvergent processes^2,4^. Stereoselectivities thus tend to vary based on the structures of the cross-coupling partners employed (Fig. 1a, left)^2–4^. In contrast, stereospecific Csp^3^ coupling has the theoretical potential to employ achiral catalysts that perfectly translate into targeted products the stereochemistry that has been pre-installed into configurationally stable building blocks (Fig. 1a, right)^3–6^. Achieving this goal with generally non-toxic Csp^3^ boronic acids or their derivatives is particularly attractive^1–4,7–9^. In a landmark paper^10^, Crudden and coworkers first demonstrated in 2009 that configurationally stable, chiral non-racemic benzylic pinacol boronic esters can be cross-coupled with retention of stereochemistry, opening the door to this approach.

There is a fundamental challenge, however, that can make it difficult to achieve perfect stereocontrol in such reactions. There are two different stereospecific pathways for transmetalation of Csp^3^ boronic acids to Pd(II), and these two pathways are stereodivergent; one leads to inversion and the other leads to retention of configuration at the Csp^3^ carbon that is being transferred (Fig. 1b)^3,11,12^. Consequently, the stereo-outcome for cross-couplings of even perfectly stereodefined Csp^3^ boronates can vary widely, or even reverse, depending on the substrate and/or reaction conditions^3,4,10–19^. A number of reports have shown that competition between these two pathways can be at least partially controlled via specifically positioned activating groups in the Csp^3^ boronate substrates^3,10–16,18^. A potential advantage of the existence of the two competing stereochemical courses is that a single enantioenriched substrate can provide both enantiomers of coupling products by careful tuning of reaction conditions^3^. This was first demonstrated by Suginome and coworkers using externally added Lewis acids to control the reversible intramolecular ligation between pinacol boronic esters and proximal Lewis basic amides^11^. However, with all of these approaches, variable levels of stereocontrol is observed depending on the structures of the cross-coupling partners employed, which can lead to challenging-to-separate mixtures of stereoisomeric products. In order to access the still largely untapped potential for stereospecific cross-coupling of Csp^3^ boronates to enable modular synthesis of complex Csp^3^-rich targets, approaches for rationally differentiating between the stereodivergent pathways for transmetalation to Pd(II) are needed. Herein we report that ligand-mediated axial shielding of a square planar Pd(II) complex enables Suzuki-Miyaura cross-couplings of a wide range of unactivated Csp^3^ boronic acids to proceed with perfect stereoretention.

## Results

### Axial shielding to promote stereoretentive Csp^3^ couplings

Multiple lines of evidence support a model in which transmetalation of Csp^3^ boronic acids can proceed through the two stereodivergent pathways depicted in Fig. 1b^3,8,9^. Stereoretentive transmetalations likely involves a closed, four-membered transition state within the Pd(II) square plane^3^. This is similar to the transmetalation pathway that has recently been characterized for Csp^2^ boronic acids^20–23^. In that case, kinetic studies suggested that coordination of the boronic acid with a palladium hydroxo complex precedes transmetalation^20,21^, and rapid injection, low-temperature NMR studies have recently characterized such intermediates^22,24^. Computational studies have further shown that the transition state in which the nascent Pd–C bond is *trans* to the phosphine ligand is nearly 10 kcal/mol lower in energy relative to the *cis* counterpart due to less steric crowding^24^. Related inner-sphere transmetalations with other Csp^3^ organometallic coupling partners proceed with retention of stereochemistry^25–27^. Alternatively, the transition state for stereoinvertive transmetalation likely involves backside electrophilic attack by Pd(II) on the boron-bearing carbon of an anionic trihydroxyborate^3,11,12^. Ligand substitution reactions on 16-electron d8 square planar complexes usually proceed through associative mechanisms which involve 18-electron complexes and ligand approach at the axial positions above or below the metal square plane^26,28^. Related stereoinvertive transmetalations of alkylstannanes similarly occur through an associative mechanism involving approach of the alkylstannanes orthogonal to the Pd(II) square plane^26^.

Spatial differences between these stereodivergent transmetalation pathways suggested an opportunity to rationally discriminate between them via ligand-based axial shielding. Specifically, we reasoned that ligands which preferentially shield the axial positions by projecting steric bulk above and below the Pd(II) square plane should selectively block stereoinvertive transmetalation, thus favoring stereoretention (Fig. 1c). Encouraging precedent for this concept can be found in Brookhart’s classic studies on the rates of associative addition of ethylene to Pd(II) complexes with variably shielded axial sites and the corresponding impacts on ethylene polymerization^29,30^.

**Fig. 2:**
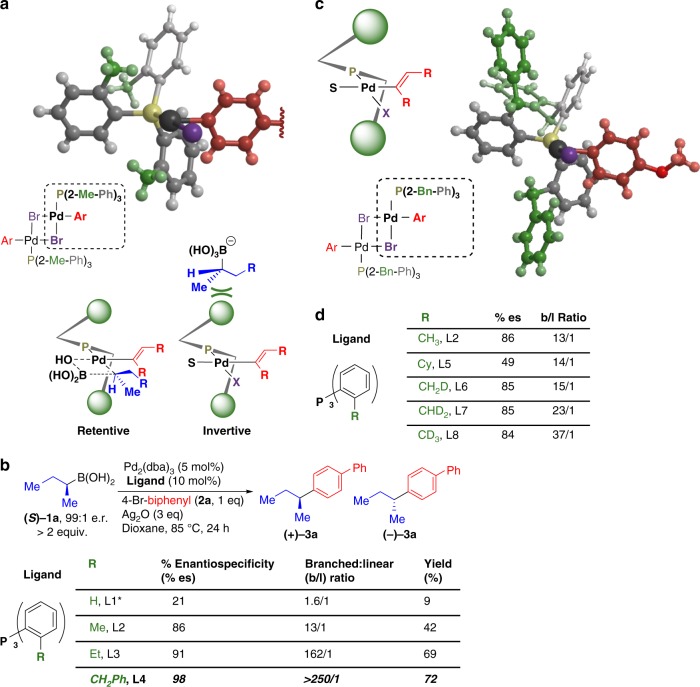
Tuning of ligand sterics. **a** Crystal structure of {[(2-Me-Ph)_3_P]Pd(4-*n*Bu-Ph)(Br)}_2_ and concept of blocking of the axial sites on Pd. **b** Model coupling reaction for optimizing ligands (*ortho*-substituent optimization) Pd(PPh_3_)_4_ was used due to insufficient yield with Pd_2_dba_3_/PPh_3_. **c** Crystal structure of {[(2-Bn-Ph)_3_P]Pd(4-OMe-Ph)(Br)}_2_ is consistent with the axial shielding ligand design principle. **d** % es and *b*/*l* ratio do not necessarily correlate as a function of ligand structure

With this axial shielding design principle in mind, we were intrigued by the X-ray crystal structure of the oxidative addition adduct {[(2-Me-Ph)_3_P]Pd(4-*n*Bu-Ph)(Br)}_2_ (Fig. 2a)^31^. We noted that in this Pd(II) complex, one *ortho*-methyl group projects above and another projects below the Pd(II) square plane. The resulting zig-zag-like orientation of these methyl groups is represented schematically below the crystal structure. This analysis suggests that axial shielding by these ortho-methyl groups will disfavor the invertive transmetalation pathway. As a result, relative to triphenyl phosphine, tri-ortho-tolyl phosphine would be expected to show increased stereorention in cross-coupling of Csp^3^ boronates. We reasoned that further increasing steric bulk at the *ortho*-positions should increasingly block the stereoinvertive pathway via enhanced axial shielding while leaving the Pd(II) square plane unencumbered, thus leading to highly stereoretentive Csp^3^ cross-couplings. P(*o*-tol)_3_ has also been shown to disfavor β-hydride elimination^32^, which is an important additional advantage for Csp^3^ coupling (vide infra).

We thus tested how different *ortho*-substituted triarylphosphine ligands impact the enantiospecificity of a representative cross-coupling of an unactivated chiral Csp^3^ boronic acid **(*S*)-1a** with *para*-bromobiphenyl **2a**. (Fig. 2b). We began our studies with Pd(PPh_3_)_4_ as the catalyst and Ag_2_O as base, as originally reported by Crudden and coworkers in the stereospecific cross-coupling of benzylic pinacol esters^10–13^. A survey of alternative bases and solvents demonstrated that Ag_2_O and dioxane were optimal. A reduced yield was observed with Ag_2_CO_3_, and non-silver-containing bases provided no yield (Supplementary Figure 1a)^33,34^. Attempts to couple the Bpin derivative of **1a** yielded none of the cross-coupling product. Using **(*S*)-1a** as the substrate and Pd(PPh_3_)_4_ as catalyst, we observed the stereoretentive product **(+)-3a**, but with only 21% enantiospecificity (Fig. 2b, entry 1). Replacing triphenylphosphine with P(*o*-tol)_3_ (entry 2) increased the enantiospecificity to 86%. Further increasing the size of the *ortho* substituents to ethyl groups (entry 3) increased the enantiospecificity to 91%. Finally, near perfect enantiospecificity (98%) was achieved with the ligand, tri(2-benzyl-phenyl)phosphine, **L4** (entry 4). Notably, across the same series of ligands, the yield and branched/linear (*b*/*l*) ratios also increased in parallel, such that **L4** provided a yield of 72% and a near-perfect *b*/*l* ratio (>250/1). Alternatively performing this reaction with 1 equiv. of boronic acid **1a** and 2 equiv. of halide **2a** provided a 30% yield based on the limiting boronic acid (Supplementary Figure 1b).

To gain insight into how **L4** promotes such high levels of stereoretention, we obtained a crystal structure of the dimeric Pd(II) oxidative addition adduct {[(2-Bn-Ph)_3_P]Pd(4-OMe-Ph)(Br)}_2_ (Fig. 2c). Like the corresponding P(*o*-tol)_3_ complex (Fig. 2a), one of the benzyl groups is rotated to project away from the Pd center, and the remaining two sterically bulky benzyl substituents are projected above and below the square plane of the Pd(II) complex. These results are consistent with the model in which increased axial shielding selectively inhibits a competing stereoinvertive transmetalation pathway (Fig. 1c).

We also considered the alternative possibility that the improved enantiospecificity for ligands **L1**-**L4** was attributable to mitigation of a potential alternative pathway for racemization involving β-hydride elimination followed by hydropalladation of a prochiral olefin (Supplementary Figure 2)^3^. In this scenario, the enantiospecificity would be expected to increase in parallel with the *b*/*l* ratio, as we observed for **L1**-**L4**. However, three distinct lines of evidence suggest that the enantiospecificities and *b*/*l* ratios are not mechanistically coupled.

**Fig. 3:**
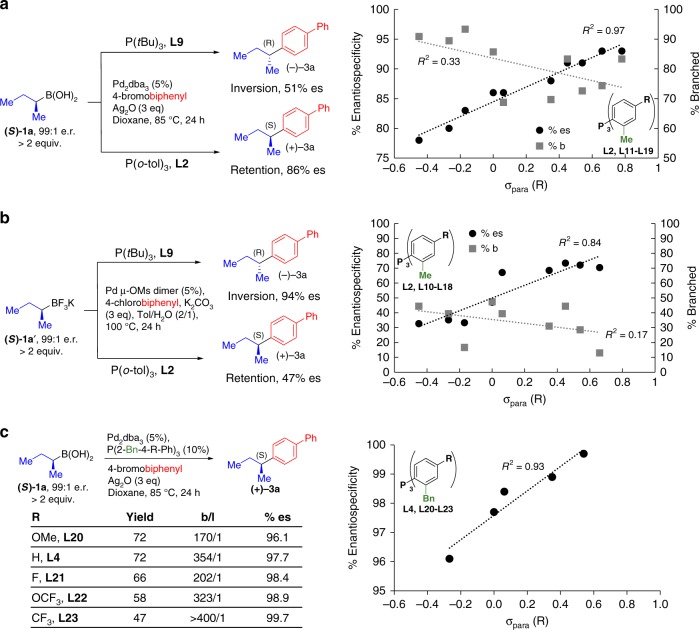
Tuning of ligand electronics. The effect of ligand electronic tuning on stereooutcome and *b*/*l* ratio under both anhydrous (**a**) and biphasic (**b**) conditions (%branched = %*b*–%*l*). **c** Electronic tuning of **L4** leads to perfect enantiospecificity for the cross-coupling of challenging substrate (***S***)-**1a**

First, we also prepared a tri-ortho-cyclohexyl phenyl phosphine ligand, **L5**, which may be too large and therefore sterically encumber the Pd(II) square plane. This ligand provided the product (+)**-3a** with a nearly identical *b*/*l* ratio but substantially reduced enantiospecificity relative to the same reaction performed under otherwise identical conditions with **L2** (Fig. 2d). Second, we synthesized a series of derivatives of P(*o*-tol)_3_ with 1,2, or 3 deuterium atoms incorporated into each of the *ortho*-methyl groups (**L6**-**L8**). Such deuteration would not be expected to significantly modify the extent of axial shielding, but may impact the rate of β-hydride elimination^35^. We observed no increase in enantiospecificity but progressive increases in *b*/*l* ratios with increasing levels of deuteration (Fig. 2d). Third, as detailed below, we observed anti-correlations between enantiospecificity and *b*/*l* ratios when **L2** was electronically tuned (Fig. 3a, b).

### Combining axial shielding with ligand electronic effects

During the course of our studies, we also observed interesting effects on both stereooutcome and *b*/*l* ratios when we combined the axial shielding approach with electronic tuning of the ligands. We were initially intrigued by our observations of stereodivergence when we cross-coupled **(*S*)-1a** or its trifluoroborate counterpart (**(*S*)-1a’**) under identical conditions, including both aqueous basic conditions as well as anhydrous conditions (Fig. 3a, b). As recently shown, P(*t*Bu)_3_ promotes the primarily stereoinvertive Csp^3^ cross-coupling of 2-butyl potassium trifluoroborate **(*S*)-1a’** with aryl chloride **2a’**^16^, whereas we found that P(*o*-tol)_3_ **L2** leads primarily to stereorentention under these coupling conditions. We noted that these two ligands have similar cone angles [(P(*t*Bu)_3_, cone angle θ = 182°; **L2**, θ = 194°)^36^] but different Tolman Parameters [(P(*t*Bu)_3_, ν = 2056.1 cm^−1^; **L2**, *ν* = 2066.6 cm^−1^)^36^. We thus questioned whether ligand electronics might provide a second handle to further fine tune stereochemical control in combination with axial shielding.

We therefore synthesized and tested (Supplementary Figure 3) a systematically varied series of P(*o*-tol)_3_ derivatives **L10**-**L19** with a range of electron-donating and electron-withdrawing functional groups at the position *para* to the phosphorus atom (Fig. 3a, b). Such modifications represent more fine-tuning of the electronic nature of the phosphine ligands (Tolman Parameters for P(4-R-Ph)_3_: R = OMe, ν = 2066.1 cm^−1^; R = H, *ν* = 2068.9 cm^−1^; R = F, *ν* = 2071.3 cm^−1^)^36^. Under both sets of cross-coupling conditions, we observed a strong positive correlation (*R*^2^ = 0.97 and 0.84) between the electron-deficient nature of the phosphine ligand and the enantiospecificity of the coupling reaction under both aqueous and anhydrous conditions (Fig. 3a, b). While this paper was under review, similar electronic trends were reported by Biscoe and Sigman^37^.

We also observed generally decreased *b*/*l* ratios as ligands became more electron-deficient (Fig. 3a, b, Supplementary Figure 3). As noted above, this provides a third line of evidence that enantiospecificity and *b*/*l* ratios are not necessarily linked via a common mechanism. The more electron-deficient ligands also tended to provide lower yields. Moreover, even with the most electron-deficient variants of **L2**, the levels of stereoretention were inferior to those observed with the more axially shielding ligand P(2-Bn-Ph)_3_ **L4**.

We thus also synthesized a similar series of electronically tuned variants of **L4**. We again observed a strong positive correlation (*R*^2^ = 0.93) between electron deficiency and enantiospecificity, with the most electron-poor derivative P(2-Bn-4-CF_3_-Ph)_3_ **L23** yielding perfect stereoretention (>99%), albeit with reduced yield (Fig. 3c).

Collectively, these results show that axial shielding can be combined with electronic tuning to maximize stereoretention in the cross-coupling of a very small and thus especially challenging Csp^3^ boronic acid such as **(*S*)-1a**. However, given the corresponding reductions in yield and/or *b*/*l* ratios, we questioned whether more sterically bulky Csp^3^ boronic acids, which should be more sensitive to steric effects, might be coupled with near perfect stereoretention using only the axial shielding approach.

### Axial shielding yields perfect stereoretention

Exploring the reaction scope for cross-coupling with axially shielding ligand **L4** first required access to a series of unactivated chiral nonracemic boronic acids in highly enantiomerically enriched form. Chiral derivatives of *N*-methyliminodiacetic acid (MIDA) have previously been used to promote diastereoselective epoxidations^38^ and resolution of atropdiastereomeric biaryl boronic acids^39^. We found that upon complexing a range of racemic secondary alkylboronic acids with a homochiral MIDA variant, *N*-2-benzyloxycyclopentyl-iminodiacetic acid (BIDA), the diastereomeric BIDA boronates could be readily separated by chromatography and/or recrystallization to provide highly enantioenriched boronic acids (≥99:1enantiomeric ratio (e.r.)) masked as air-stable building blocks (Supplementary Figure 4).

**Fig. 4:**
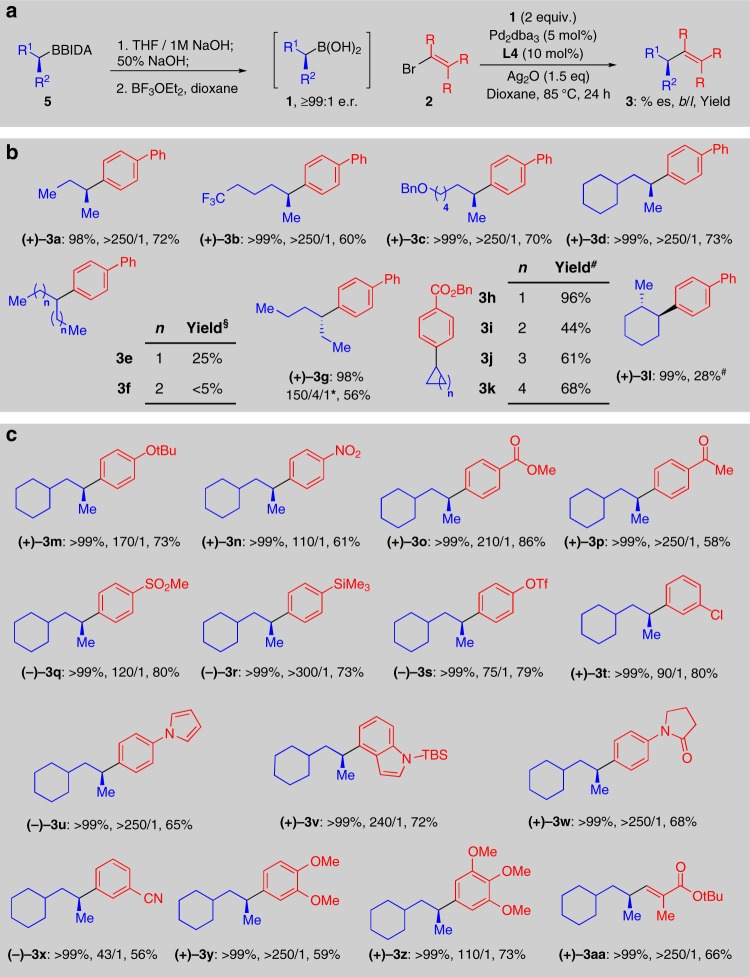
Substrate scope of the sterospecific cross-coupling reaction. **a** Reaction conditions. **b** Scope of the Csp^3^ boronic acid coupling partner, (^§^: no improvement when using **L2**, ^*^: branched on C3/branched on C2/linear (Supplementary Figure 5), ^#^: **L2** was used, **L4** gave lower yields). **c** Scope of the bromide coupling partner. All reactions were performed in duplicate. Branched/linear ratios were determined by HPLC or GC analysis of the crude reaction using an authentic linear product standard. Yields were determined by isolation. Enantiospecificities were determined by chiral HPLC of the purified product

We found that best results in the subsequent Csp^3^ coupling reactions were achieved using anhydrous solutions of pure boronic acids. To prepare such solutions without forming boroxines, we hydrolyzed the BIDA boronates, added sodium hydroxide to generate the corresponding sodium trihydroxyborate salts^40^, collected them by filtration and then treated these salts with one equivalent of BF_3_^●^OEt_2_ to generate anhydrous solutions of the free boronic acids (Fig. 4a).

Using this methodology, a range of unactivated secondary Csp^3^ boronic acids (**1a**–**1d**) were prepared in ≥99:1 e.r. (Supplementary Figure 4) and tested in cross-coupling reactions promoted by **L4** (Fig. 4b). In general, the boronic acids are fully consumed in these reactions. Perfect stereoretention was observed in most cases. A trifluoromethyl group (**3b**), a benzyl ether (**3c**) and beta branching (**3d**) were compatible with this reaction. All of these boronic acids also coupled in good yields and with *b*/*l* ratios > 250/1 (**1a**–**1d**). Moving the boronic acid to a more internal position along a linear alkyl chain was somewhat tolerated (**3e** and **3g**), but not beyond 2 carbon atoms (**3f**). For 3-hexylboronic acid (**1g**), a unique example of cross-coupling an unactivated ethyl-branched chiral Csp^3^ boronic acid, we observed a 150/4/1 product distribution of the desired 3-hexyl (**3g**) to 2-hexyl (**3g’**) to 1-hexyl isomers (**4g**) and near perfect stereoretention (Supplementary Figure 5). A range of cyclic boronic acids (**3h-k**) were also effective using **L2** rather than **L4**. We also prepared and cross-coupled non-racemic 2-methyl cyclohexyl boronic acid (**1l**), and observed perfect diastereo- and enantiospecificity (**3l**). The latter result shows that the Pd catalyst does not walk around the ring via sequential cycles of β-hydride elimination and hydropalladation to yield the enantiomeric product (Supplementary Figure 6).

Perfect stereoretention was also observed for cross-coupling with a wide range of electron-rich and electron-poor aryl halides (Fig. 4c). The functional group tolerance of this method was also found to include ethers (**3m**, **3y**, **3z**), nitro groups (**3n**), esters (**3o**), ketones (**3p**), sulfones (**3q**), silanes (**3r**), and nitriles (**3×**). Although aryl triflates and aryl chlorides were poor coupling partners, their lack of reactivity provided the opportunity for halide-selective coupling (**3s**, **3t**). Some heterocycles, including pyrroles (**3u**) and indoles (**3v**), were also well-tolerated. Finally, we observed a cross-coupling in which perfect stereoretention is observed at both the Csp^3^ and Csp^2^ centers between an unactivated secondary boronic acid and an activated vinyl halide (**3aa**).

### Perfectly stereocontrolled synthesis of Csp^3^-rich molecules

**Fig. 5:**
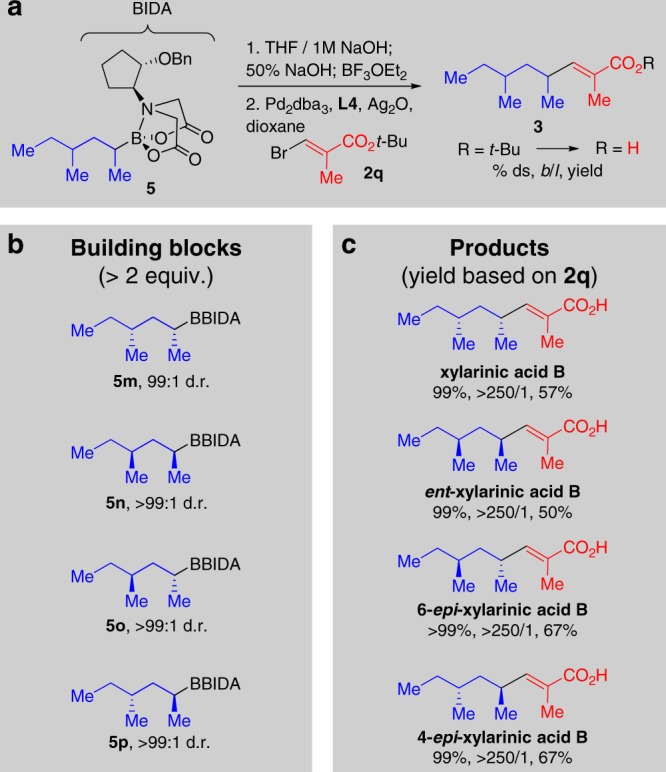
Building block-based synthesis of all possible Csp^3^ stereoisomers of xylarinic acid B. **a** Conditions for deprotection and coupling. **b** Air-stable BIDA boronate building blocks prepared by resolution. **c** Xylarinic acid B and all possible Csp^3^ steroisomers accessed by stereospecific cross-coupling of stereodefined building blocks. % ds, *b*/*l*, and yield refer to the cross-coupling step

We finally tested the capacity of this method to enable stereocontrolled Lego-like assembly^9^ of Csp^3^-rich natural products. One of the key strengths of this approach is the theoretical potential to access all possible Csp^3^ stereoisomers with perfect stereocontrol by employing the corresponding series of pre-fabricated, stereochemically defined chiral building blocks. The recently discovered antifungal natural product xylarinic acid B^41,42^ served as an excellent case study (Fig. 5).

Leveraging the simple BIDA boronate resolution method, all of the required secondary Csp^3^ boronate building blocks were prepared in ≥99:1 diastereomeric ratio (d.r.) (Fig. 5b). All four of these building blocks proved to be air- and chromatographically stable crystalline solids.

This set the stage for the simple modular assembly of xylarinic acid B, and all of its Csp^3^ stereoisomers, via cross-coupling of these four stereodefined building blocks. Each building block was deprotected to the corresponding boronic acid, stereospecifically cross-coupled to vinyl bromide **2q** using axially shielding ligand **L4**, and then deprotected under standard conditions (TFA/DCM). Through this simple approach, xylarinic acid B and all three of its Csp^3^ stereoisomers were readily accessed in perfect diastereospecificity (≥99%), >250/1 *b*/*l* ratios, and ≥50% yield (Fig. 5c).

We have found that axial shielding of Pd(II) complexes can lead to perfect stereoretention in the Suzuki–Miyaura cross-coupling of unactivated Csp^3^ boronic acids. Optimizations can also be achieved with especially challenging substrates by combining axial shielding with ligand electronic tuning. A wide range of phosphine ligands may be good candidates for similar steric and/or electronic tuning to rationally promote stereocontrolled Csp^3^ coupling. These principles might also be extended to the stereospecific coupling of other types of configurationally stable coupling partners, such as alkylstannanes and alkylsilanes, which have also been proposed to proceed via energetically competitive stereodivergent transmetalation pathways with open and closed transition states^27,43,44^. Continued progress in such directions stands to help enable the transformative potential impact of stereocontrolled Csp^3^ cross-coupling to be realized.

## Methods

### Synthesis of MIDA and BIDA boronates

To a 250-mL round-bottom flask with a stir bar was added racemic boronic acid (10 mmol, 1.0 equiv.), *N*-methyliminodiacetic acid (MIDA) (1.77 g, 12 mmol, 1.2 equiv.) and DMSO (10 mL, 1.0 M in boronic acid), and toluene (90 mL, 0.11 M in boronic acid). The mixture was fitted with a Dean Stark trap, on top of which was fitted a reflux condenser. The mixture was heated to reflux and water was collected in the trap for 2 h, at which point complete conversion of the boronic acid was confirmed by TLC. The toluene was then removed by rotary evaporation, H_2_O (75 mL) was added, and the mixture was extracted with EtOAc (5 × 75 mL). The combined organic layers were washed with H_2_O (5 × 75 mL). The organic phase was then dried over Na_2_SO_4_ and concentrated under vacuum to give the corresponding MIDA boronate as a white solid which was used without purification. The synthesis of BIDA boronates was performed with this same procedure, using 2,2’-(((1*S*,2*S*)-2-(benzyloxy)cyclopentyl) azanediyl)diacetic acid (BIDA) (2.55 g, 8.3 mmol, 0.83 equiv.) instead of MIDA. The resulting diastereomeric mixtures were resolved by recrystallization from acetone and/or column chromatography and then used directly for the synthesis of sodium alkyltrihydroxyborate salts. For full experimental details, ^1^H NMR, and ^13^C NMR spectra see Supplementary Methods.

### Synthesis of sodium alkyltrihydroxyborate salts

To a stir bar-equipped 250-mL round-bottom flask under air was added MIDA or BIDA boronate (6.499 mmol, 1.00 equiv.), THF (33 mL, 0.20 M) and freshly prepared aqueous NaOH (1 M, 33 mL, 5.0 equiv.). The mixture was stirred at 23 °C until complete conversion was confirmed by TLC. THF was removed under rotary evaporation (bath temperature 40 °C). When most of the THF was removed, the receiving flask was emptied, dried and rotary evaporation was then continued until water began to collect in the receiving flask indicating complete removal of THF. Saturated NH_4_Cl (33 mL) was added to the resulting aqueous solution and the aqueous layer was extracted with MTBE (4 × 33 mL). The combined organic layers dried over Na_2_SO_4_, filtered and partially concentrated (volume ~33 mL, 0.20 M) by rotary evaporation. To this solution was added aqueous 50% NaOH (0.343 mL, 0.520 g solution, 0.260 g NaOH, 6.50 mmol) over 1 min with rapid stirring. The suspension was stirred for 20 min at 23 °C, causing a white precipitate to form. The flask was then sonicated for 5 min and the white precipitate was collected by concentration in vacuo or by filtration through a medium porosity glass frit, rinsing with MTBE. The product was dried under vacuum at <1 mbar at 23 °C for 10 h to give the corresponding trihydroxyborate salt as a colorless, free-flowing powder. For full experimental details, ^1^H NMR, and ^13^C NMR spectra see Supplementary Methods.

### Synthesis of boronic acids as dioxane solutions

Sodium alkyltrihydroxyborate (1.50 mmol, 1.00 equiv.) was added to a 2-mL screw-cap vial with a stir bar. Anhydrous dioxane (1.15 mL, 1.3 M) was added and the slurry was vigorously stirred. BF_3_·OEt_2_ (0.185 mL, 0.213 g, 1.50 mmol, 1.00 equiv.) was added dropwise over 15 min under air. If the mixture became unstirrable, it was periodically capped and shaken by hand. After completion of the addition, the vial was capped and stirred for 20 min. The resulting fine suspension was filtered by passing through a Pasteur pipette containing 40 mg of Celite over a small cotton plug, using air pressure. The residue from the vial was washed through with additional dioxane (0.35 mL, 1.0 M theoretical concentration). The resulting homogeneous solution amounted to 1.15 mL. An aliquot of this solution (30 µl, 30 µmol theoretical) was combined with a standard solution DMSO-d_6_ and 1,4-dimethoxybenzene (0.050 M, 0.60 mL, 30 µmol 1,4-dimethoxybenzene) in an NMR tube. The boronic acid was analyzed by ^1^H-NMR and the concentration of boronic acid was determined. This solution was diluted to 0.91 M by adding dry dioxane (0.20 mL) and was then transferred in a capped vial into a glovebox and used directly in cross coupling reactions. For full experimental details, ^1^H NMR, and ^13^C NMR spectra see Supplementary Methods.

### Cross-coupling of secondary boronic acids

To a stir bar-equipped 7 mL vial were added **L4** (5.3 mg, 0.010 mmol, 10 mol%), Pd_2_dba_3_ (4.6 mg, 0.0050 mmol, 5 mol%), aryl or vinyl halide (0.100 mmol, 1.00 equiv.), and Ag_2_O (34.8 mg, 0.3 mmol, 1.5 equiv.). A dioxane solution of freshly prepared boronic acid (0.91 M, 0.220 mL, 0.200 mmol, 2.00 equiv.) was added by pipette. The vial was tightly sealed with a teflon-lined screw cap and stirred at 200 rpm at 85 °C for 24 h. Upon completion, the reaction mixture was filtered through a silica gel plug in a Pasteur pipette, rinsing with EtOAc or Et_2_O. An aliquot of the crude reaction mixture was subjected to HPLC analysis to determine the *b*/*l* product ratio, comparing with an authentic sample of the linear product isomer. The crude reaction was then purified by column chromatography on silica gel, and the enantiospecificity was determined by chiral HPLC. For full experimental details, ^1^H NMR, and ^13^C NMR spectra see Supplementary Methods.

## Supplementary information

Supporting Information

## Data Availability

The authors declare that the data supporting the findings of this study are available within the article and its supplementary information files. The X-ray crystallographic coordinates for structures reported in this study have been deposited at the Cambridge Crystallographic Data Centre (CCDC), under deposition numbers **SI-45** 1838473, **SI-46** 1838474, **5a** 1838475 and **6****l** 1894457. These data can be obtained free of charge from The Cambridge Crystallographic Data Centre via www.ccdc.cam.ac.uk/data_request/cif
