# Supplementary material for: Axial shielding of Pd(II) complexes enables perfect stereoretention in Suzuki-Miyaura cross-coupling of Csp3 boronic acids
Source: Nat Commun. 2019 Mar 20;10:1263. doi: 10.1038/s41467-019-09249-z (PMC6427018; doi:10.1038/s41467-019-09249-z)
Supplement: Supplementary file 1 — Supporting Information [file 41467_2019_9249_MOESM1_ESM.pdf]

Supplementary Information for:

Axial shielding of Pd(II) complexes enables perfect stereoretention in Suzuki-Miyaura cross-coupling of Csp^3 boronic acids

Lehmann et al.

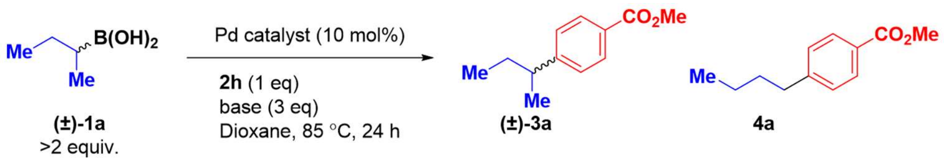

catalyst	base	Yield (%)	b/l Ratio
$Pd(PPh_3)_4$	Ag_2O	45	0.9/1
$Pd(PPh_3)_4$	Ag_2CO_3	35	0.8/1
$Pd(PPh_3)_4$	K_2CO_3	<5	-
Pd_2dba_3/dppf	Ag_2O	<5	-
Pd[P(*t*-Bu)$_3$]$_2$	Ag_2O	13	0.8/1
Pd_2dba_3/SPhos	Ag_2O	<5	-
Pd_2dba_3/P(*o*-tol)$_3$	Ag_2O	48	>20:1
Pd_2dba_3/PPh_3	Ag_2O	30	0.7/1
Pd[P(*o*-tol)$_3$]$_2$	Ag_2O	75	14/1

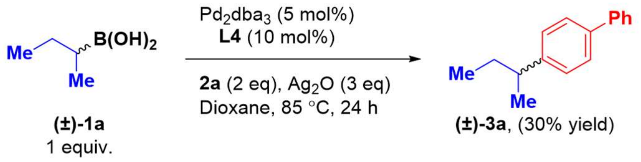

Supplementary Figure 1│Reaction optimization data. a, Model reaction for optimizing reaction conditions. **b,** reversed stochiometry

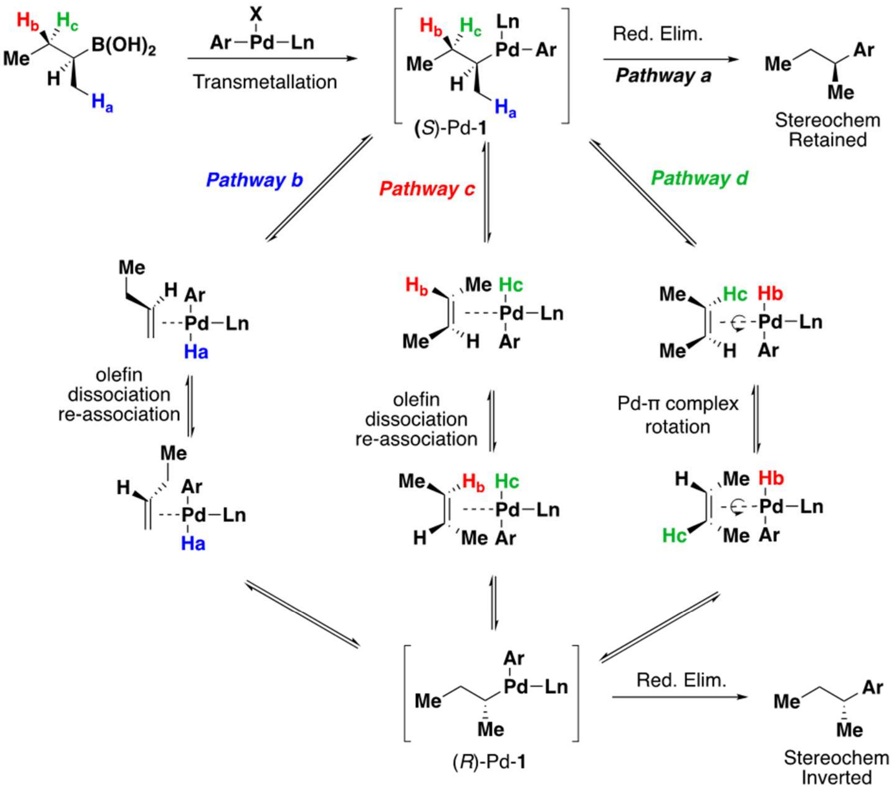

Supplementary Figure 2│Theoretical potential influence of Beta Hydride Elimination on Stereospecificity. Pathways for stereoinverted coupled product through beta hydride elimination from transmetallation intermediate (S)-Pd-**1**

a

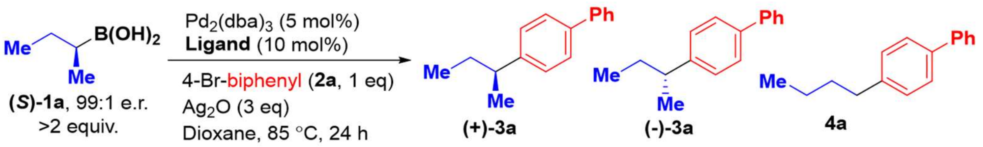

b

Ligand

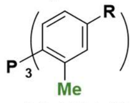

L2, **L11**-**L19**

R	σ_p	%es	b/l Ratio	Yield (%)
O*i*Pr, **L11**	-0.45	78	21/1	26
OMe, **L12**	-0.268	80	18/1	26
Me, **L13**	-0.17	83	29/1	38
H, **L2**	0	86	13/1	43
F, **L14**	0.062	86	5.4/1	42
OCF_3, **L15**	0.35	88	5.6/1	46
CO_2Me, **L16**	0.45	91	11/1	54
CF_3, **L17**	0.54	91	6.3/1	53
CN, **L18**	0.66	93	6.8	19
NO_2, **L19**	0.78	93	11/1	27

c

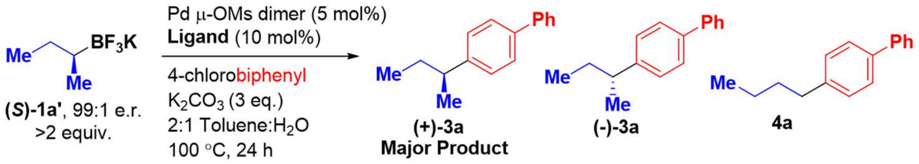

d

Ligand

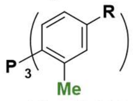

L2, **L10**-**L18**

R	σ_p	Yield	b/l	%es
NMe_2, **L10**	-0.83	1	N/A	9
OiPr, **L11**	-0.45	25	2.6/1	33
OMe, **L12**	-0.268	29	2.3/1	35
Me, **L13**	-0.17	33	2.4/1	33
H, **L2**	0	37	2.8/1	47
F, **L14**	0.062	36	2.3/1	67
OCF_3, **L15**	0.35	26	1.9/1	69
CO_2Me, **L16**	0.45	23	2.6/1	73
CF_3, **L17**	0.54	14	1.8/1	72
CN, **L18**	0.66	4	1.3/1	70

Supplementary Figure 3 | The effect of modifying ligand electronics. a, Model reaction for testing ligand electronics under silver oxide conditions. **b**, Testing *para*-electronics on $P(2\text{-}Me\text{-}4\text{-}R\text{-}Ph)_3$ **c,** Model reaction for testing ligand electronics under biphasic conditions. **d,** Testing *para*-electronics on $P(2\text{-}Me\text{-}4\text{-}R\text{-}Ph)_3$.

Yield based on 50% theoretical yield.

a

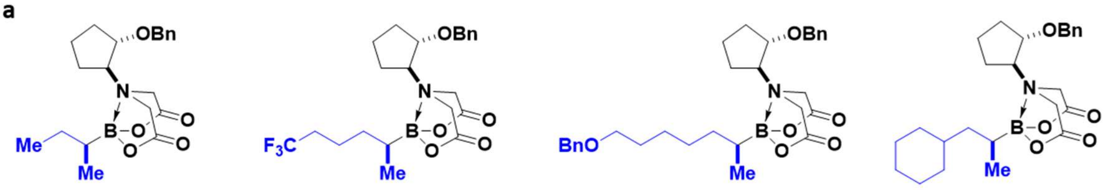

5a, 22% yield ≥99:1 d.r.

5b, 37% yield ≥99:1 d.r.

5c, 31% yield ≥99:1 d.r.

5d, 12% yield ≥99:1 d.r.

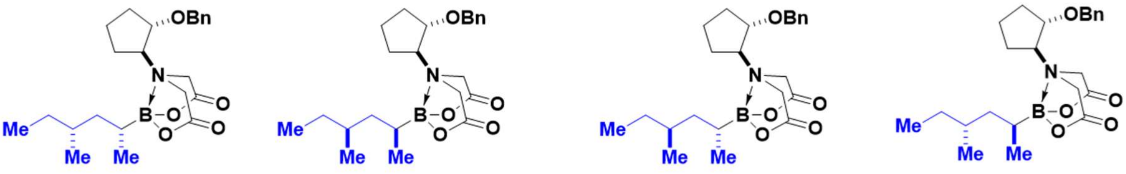

5m, 19% yield 98.7 : 1.3 d.r.

5n, 24% yield ≥99:1 d.r.

5o, 36% yield ≥99:1 d.r.

5p, 21% yield ≥99:1 d.r.

b

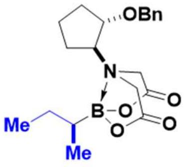

5a

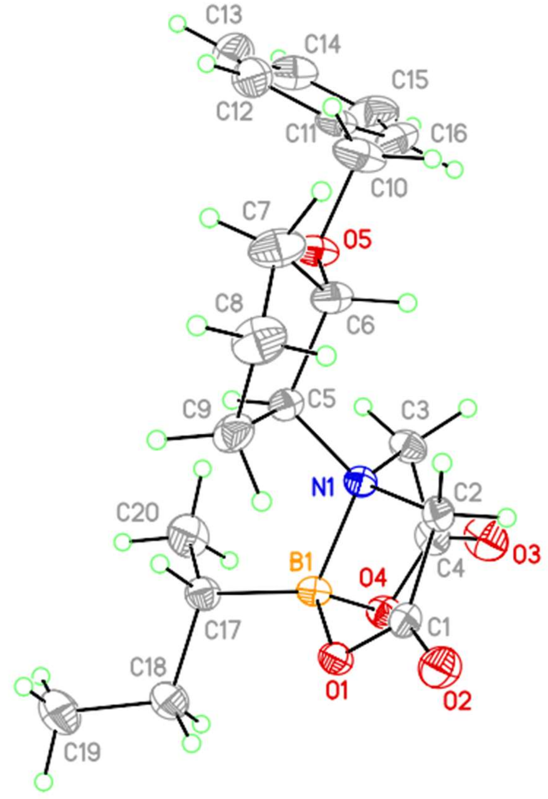

Supplementary Figure 4│BIDA boronate resolution. a, Scope of BIDA boronate resolution method. Yields are based on a 50% theoretical maximum **b**, Crystal structure of 2-butyl BIDA boronate.

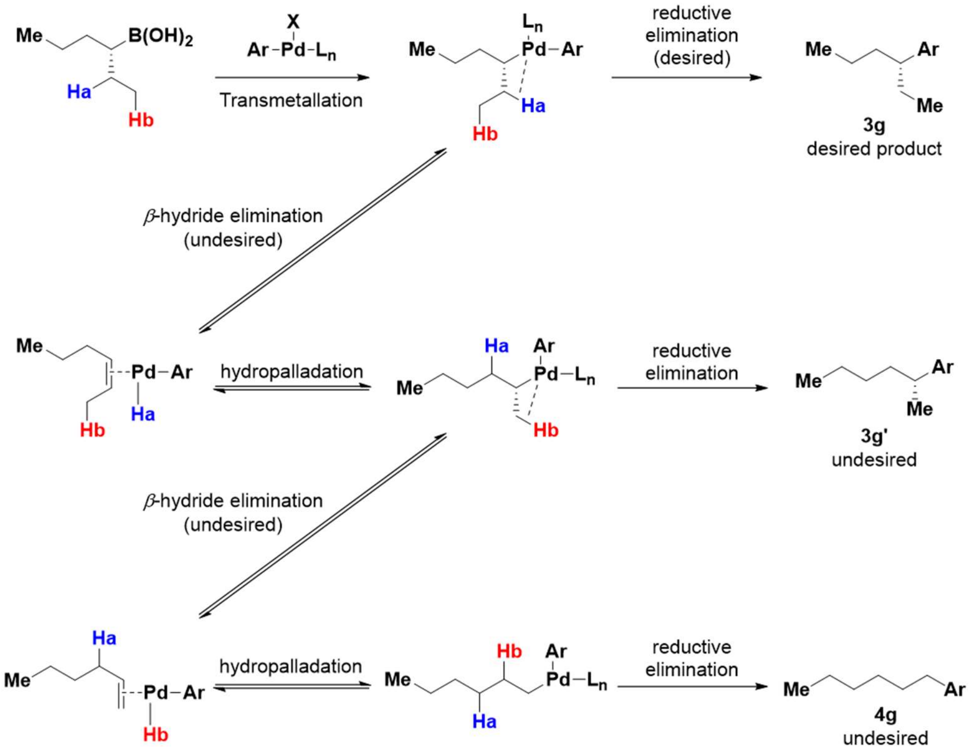

Supplementary Figure 5 | Pathways leading to branching on C2 (3g’) and the corresponding linear by-product 4g.

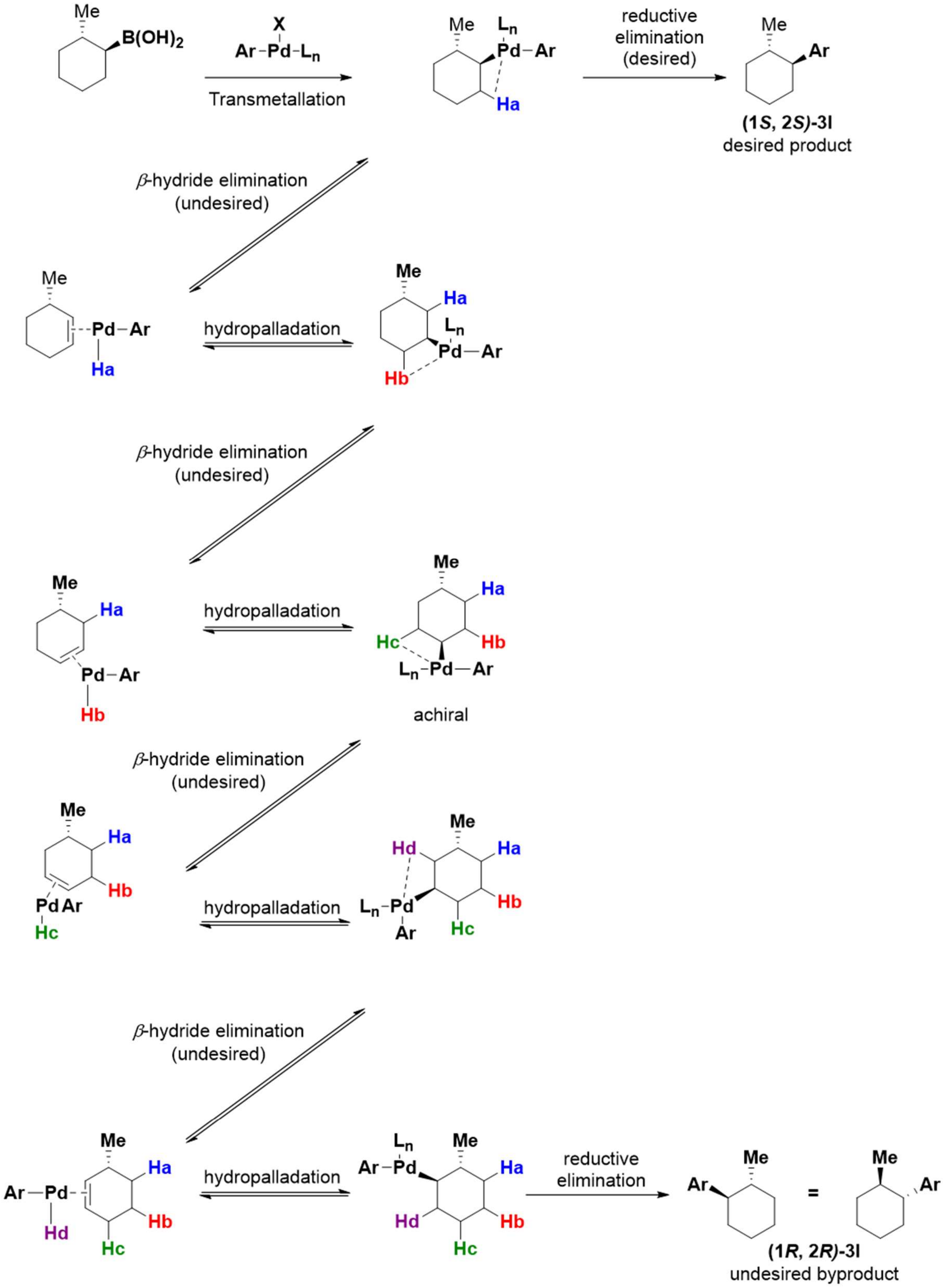

Supplementary Figure 6 | Potential pathway leading to racemization of product 3l. Path to racemization of distal stereocenter in **1l** via iterative β-hydride elimination/hydropalladation.

Supplementary Methods

Materials. Commercial reagents were purchased from Sigma-Aldrich, Fisher Scientific, Alfa Aesar, TCI America, Frontier Scientific, or Matrix Scientific, and were used without further purification, with the following exceptions. All commercially available aryl halides were purified by either column chromatography or silica gel filtration to remove baseline impurities, followed by concentration under vacuum. Ag_2O was purchased from Sigma-Aldrich. A gift of $Pd(P(o\text{-tol})_3)_2$ was donated by Johnson Matthey. Solvents were purified via passage through packed columns as described by Pangborn and coworkers[1] (THF, Et_2O, CH_3CN, CH_2Cl_2: dry neutral alumina; hexane, benzene, and toluene, dry neutral alumina and Q5 reactant; DMSO, DMF: activated molecular sieves). Acetone was dried by stirring 24 hours with boric anhydride followed by distillation. Anhydrous 1,4-dioxane was purchased from Sigma-Aldrich and used without further manipulation. All water was deionized prior to use.

General experimental procedures. Unless noted, all reactions were performed in round bottom flasks fitted with rubber septa or Teflon-lined screw-cap vials (vials: VWR catalog number 66022-300; vial caps: VWR catalog number 16198-911) under argon or nitrogen. Organic solutions were concentrated via rotary evaporation under reduced pressure with a bath temperature of 30°C unless otherwise noted. Reactions were monitored by analytical thin layer chromatography (TLC) performed using the indicated solvent on normal phase Merck silica gel 60 F254 plates (0.25mm) or reverse phase Merck silica gel 60 RP-18 $F_{254}s$ plates. Compounds were visualized by exposure to a UV lamp (λ = 254 nm), and/or a solution of KMnO4 and/or a solution of cerium ammonium molybdate followed by brief heating using a Varitemp heat gun. Normal phase column chromatography was performed using Merck silica gel grade 9385 60Å (230-400 mesh), and reverse phase column chromatography was performed with Luknova C-18 silica gel SGFLASHC18-1. Preparative HPLC was performed with a Waters SunFire™ Prep C18 OBD™ 5µm 30mm x 150mm column, Part No. 186002797. Chiral HPLC was performed with a Chiralcel® OD-H column (4.6mm x 250mm, 5µm particle size, part No. 14325), a Chiralcel® AD-H column (4.6mm x 250mm, 5µm particle size, part No. 19325), and a Chiralcel AD-RH column (4.6mm x 150mm, 5µm particle size, part No. 19724). Chiral GC was performed with a Cyclodex-B column (30m x 0.250mm, 0.25µm film, part. No. 112-2532).

Structural analysis. ^{1}H-NMR and ^{13}C-NMR spectra were recorded on Varian Unity 500, Varian Unity Inova 500NB, Varian Unity 400, Varian Unity 500, or Carver B500 instruments. ^{11}B-NMR were recorded on a Varian Unity 400 spectrometer. ^{31}P-NMR and ^{19}F-NMR were recorded on Varian Unity Inova 500, Varian Unity 500, or Varian Unity 400 instruments. Chemical shifts (δ) are reported in parts per million (ppm) downfield from tetramethylsilane and referenced to residual protium in the NMR solvent (benzene, δ = 7.16; $CHCl_3$, δ = 7.26; acetone, δ = 2.05, center line; DMSO δ = 2.50, center line) or to added tetramethylsilane (δ = 0.00). ^{13}C NMR spectra in D_2O are referenced to added acetonitrile (δ = 119.68, 1.47). For ^{1}H spectra taken at 50°C, the HOD peak was set to 4.496 ppm.[2] Data are reported as follows: chemical shift, multiplicity (s = singlet, d = doublet, t = triplet, q = quartet, quint = quintet, sept = septet, m = multiplet, b = broad, app = apparent), coupling constant (*J*) in Hertz (Hz), and integration. Chemical shifts (δ) for ^{13}C NMR are reported in ppm downfield from tetramethylsilane and referenced to carbon resonances in the NMR solvent (C_6D_6, δ = 128.06, center line; $CDCl_3$, δ = 77.0, center line; acetone-d_6, δ = 29.5, center line; DMSO-d_6 δ = 39.52, center line). Carbons bearing boron substituents were not observed (quadrupolar relaxation).

General Procedures

A. Synthesis of MIDA and BIDA Boronates

To a 250-mL round-bottom flask with a stir bar was added **(±)-1a** (1.02 g, 10 mmol, 1.0 eq, obtained from Frontier Scientific), *N*-methyliminodiacetic acid (MIDA) (1.77 g, 12 mmol, 1.2 eq), DMSO (10 mL, 1.0 Molar in boronic acid), and toluene (90 mL, 0.11 Molar in boronic acid). The mixture was fitted with a Dean Stark trap, on top of which was fitted a reflux condenser. The mixture was heated to reflux and water was collected in the trap for 2 hours, at which point complete conversion of the boronic acid was confirmed by TLC (100% EtOAc, $KMnO_4$). The toluene was then removed by rotary evaporation. H_2O (75 mL) was added, and the mixture was extracted with EtOAc (5 x 75 mL). The combined organic phase was washed with H_2O (5 x 75 mL). The organic phase was then dried over Na_2SO_4 and concentrated under vacuum to give **(±)-6a** as a white solid (1.49 g, 70%), which was used without purification. This material was stable in a capped vial under air on a bench top for at least 4 months.

The synthesis of BIDA boronates was performed with this same procedure, using BIDA (0.83 eq) instead of MIDA. The resulting diastereomeric mixtures were resolved by recrystallization and/or column chromatography. Specifically, crude BIDA boronate (mixture of **5a** and ***epi*-5a**, prepared from 41.2 mmol of boronic acid **(±)-1a** and 34.4 mmol BIDA) was filtered through a pad of silica gel, rinsing with acetone. After rotary evaporation, the crude product (12.03 g, 94% crude yield) was dissolved under nitrogen in anhydrous boiling acetone (35 mL). After cooling to room temperature, anhydrous Et_2O (70 mL) was added gradually. The mixture was cooled to 0°C and filtered through a medium porosity glass frit, affording a partially resolved product (4.72 g, 12.7 mmol). This diastereomeric mixture was recrystallized in the same manner (24 mL acetone, 48 mL Et_2O, giving 9.37 mmol product, 98:2 d.r.). A third recrystallization (20 mL acetone, 40 mL Et_2O) gave the ≥99:1 d.r. BIDA boronate (2.836 g, 7.598 mmol, 22% yield). ^{1}H-NMR in $CDCl_3$ showed a diastereomeric ratio of ≥99:1 by integrating the methyl signals of **5a** and ***epi*-5a** at 0.88 and 1.00 ppm, respectively.

5a, 99:1 d.r.: ^{1}H NMR (500 MHz, $CDCl_3$) δ 7.43 – 7.35 (m, 3H), 7.32 – 7.28 (m, 2H), 4.65 (d, *J* = 11.5 Hz, 1H), 4.38 (d, *J* = 11.5 Hz, 1H), 4.01 (d, *J* = 16.7 Hz, 1H), 3.90 (q, *J* = 6.8 Hz, 1H), 3.65 – 3.58 (m, 2H), 3.44 (d, *J* = 16.6 Hz, 1H), 3.32 (d, *J* = 16.9 Hz, 1H), 2.23 (m, 1H), 2.07 (m, 1H), 1.93 – 1.62 (m, 4H), 1.49 (m, 1H), 1.27 (m, 1H), 0.93 (t, *J* = 7.4 Hz, 3H), 0.88 (d, *J* = 7.0 Hz, 3H), 0.76 (m, 1H).

^{13}C NMR (126 MHz, $CDCl_3$) δ 168.91, 167.35, 136.41, 128.99, 128.76, 128.45, 79.16, 72.11, 72.09, 61.07, 56.13, 29.62, 26.60, 25.25, 21.42, 14.30, 12.79.

^{11}B NMR (128 MHz, $CDCl_3$) δ 14.03.

HRMS (ESI$^+$) Calculated for $C_{20}H_{29}BNO_5$ $(M+H)^+$: 374.2139, Found: 374.2140

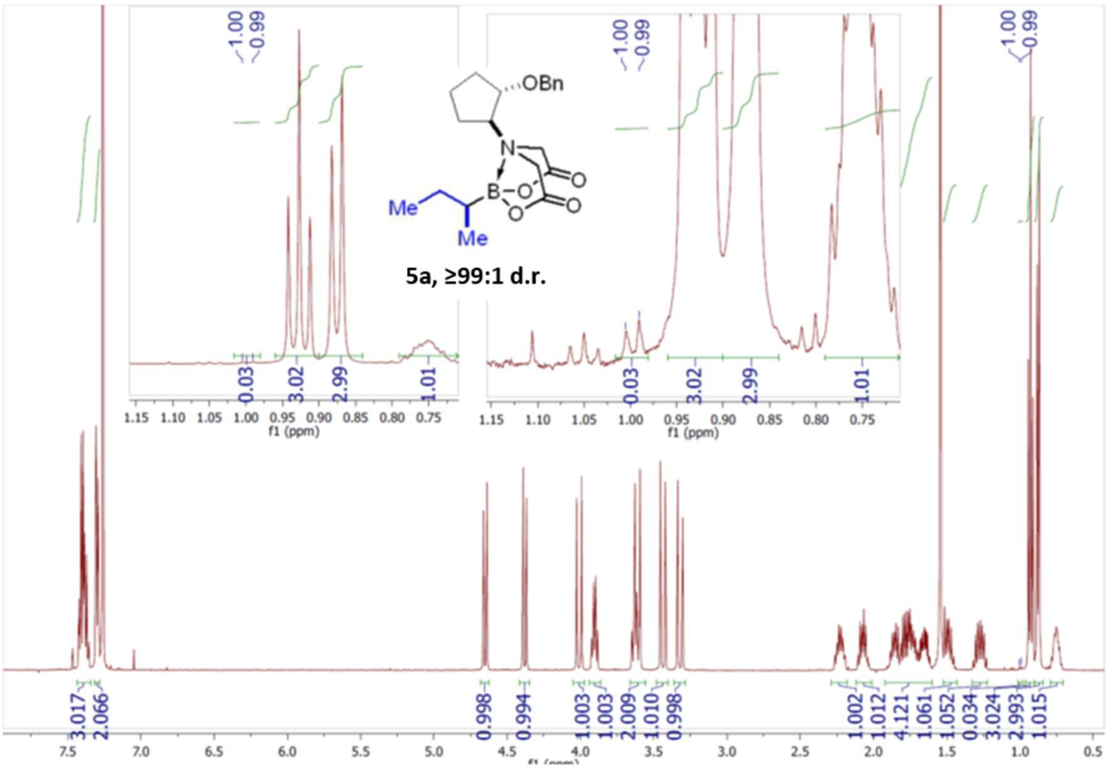
OBn
N
B
O
Me
Me
5a, ≥99:1 d.r.
f1 (ppm)

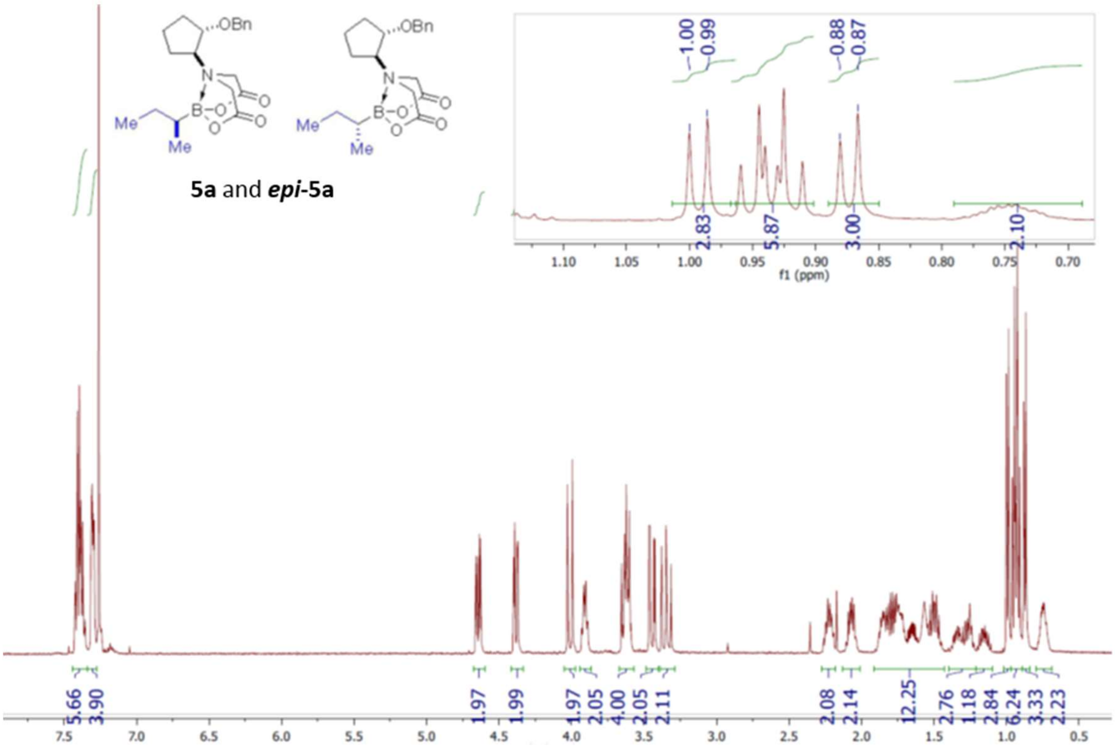
OBn
N
B
O
Me
Me
5a and *epi*-5a
f1 (ppm)

B. Synthesis of Sodium Alkyltrihydroxyborate Salts

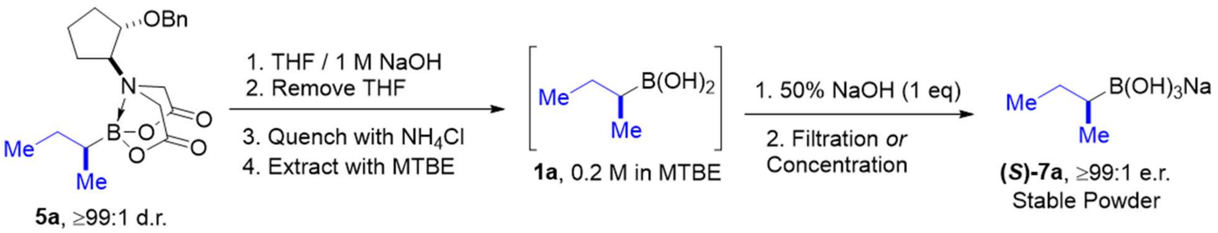

To a stir bar-equipped 250-mL round-bottom flask under air was added BIDA boronate **5a** (2.426 g, 6.499 mmol, 1.00 eq), THF (33 mL, 0.20 Molar) and freshly prepared 1 Molar NaOH (33 mL, 5.0 eq). The mixture was stirred at 23 °C until complete conversion was confirmed by TLC (1:1 Hex/EtOAc, $KMnO_4$). THF was removed under rotary evaporation (bath temperature 40°C). When most of the THF was removed, the receiving flask was emptied and dried and rotary evaporation was then continued until water condensation began to collect in the receiving flask. Saturated NH_4Cl (33 mL) was added to the resulting aqueous solution and this was extracted with MTBE (4x33 mL) in a separatory funnel. The combined MTBE phase was dried over Na_2SO_4 and partially concentrated (volume = 33 mL, 0.20 Molar) by rotary evaporation. To this solution was added aqueous 50% NaOH (0.343 mL, 0.520 g solution, 0.260 g NaOH, 6.50 mmol) over one minute with rapid stirring. The suspension was stirred for 20 minutes at 23°C, causing a white precipitate to form. The flask was then sonicated for 5 minutes. The white precipitate was collected by concentration *in vacuo* or by filtration through a medium porosity glass frit, rinsing with MTBE. The product was dried under vacuum at < 1 mbar at 23 °C for 10 hours to give **(*S*)-7a** (0.8982 g, 6.328 mmol, 97% yield)), as a colorless, free-flowing powder. This product was generally stored at 23 °C under nitrogen. In a stability test, this material was stable on the benchtop under air for at least 4 months.

^{1}H NMR (500 MHz, D_2O, 50°C) δ 1.48 – 1.36 (m, 1H), 1.00 – 0.89 (m, 1H), 0.85 (t, *J* = 7.2 Hz, 3H), 0.75 (d, *J* = 7.5 Hz, 3H), 0.20 (m, 1H).

^{13}C NMR (126 MHz, D_2O, 50°C) δ 26.56, 15.52, 14.14.

^{11}B NMR (128 MHz, D_2O, 50°C) δ 8.29.

C. Synthesis of Boronic Acids as Dioxane Solutions

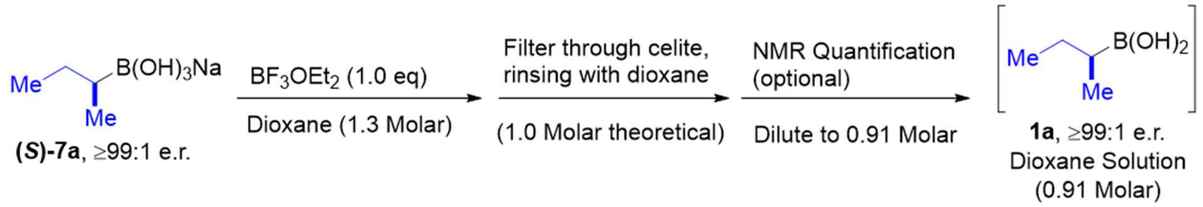

Sodium alkyltrihydroxyborate **(*S*)-7a** (0.213 g, 1.50 mmol, 1.00 eq) was added to a 2 mL screw-cap vial with a stir bar. Anhydrous dioxane (1.15 mL, 1.3 Molar) was added and the slurry was vigorously stirred. $BF_3{\cdot}OEt_2$ (0.185 mL, 0.213 g, 1.50 mmol, 1.00 eq) was added dropwise over 15 minutes under air. If the mixture became unstirrable, it was periodically capped and shaken by hand. After completion of the addition, the vial was capped and stirred for 20 minutes. The resulting thin suspension was filtered by passing through a Pasteur pipette containing 40 mg of Celite over a small cotton plug, using pressure from an applied air hose. The residue from the vial was washed through with additional dioxane (0.35 mL, 1.0 Molar theoretical concentration). The resulting homogeneous solution amounted to 1.15 mL. An aliquot of this solution (30 µl, 30 µmol theoretical) was combined with a standard solution DMSO-d_6 and 1,4-dimethoxybenzene (0.050 Molar, 0.60 mL, 30 µmol 1,4-dimethoxybenzene) in an NMR tube. The boronic acid was analyzed by ^{1}H-NMR with the relaxation delay (d1) set to 10 seconds. The concentration of boronic acid **1a** was determined to be 1.07M, giving a yield of 82%. The only visible impurity was diethyl ether (see spectrum of **(*S*)-1a** and standard). This solution was diluted to 0.91 Molar by adding dioxane (0.20 mL) and was then transferred in a capped vial into a glovebox and used in the cross coupling step. In a stability test, this boronic acid solution was stored on the benchtop under air for 4 months and showed less than 10% decomposition.

This procedure proved to be scalable and could also be carried out in 7 mL vials using 3-4 mmol of the sodium alkyltrihydroxyborate.

^{1}H NMR (400 MHz, DMSO-d_6) δ 7.25 (s, 2H), 1.33 (dq, *J* = 15.2, 7.8 Hz, 1H), 1.17 (dq, *J* = 13.5, 6.7 Hz, 1H), 0.88 – 0.74 (m, 6H), 0.69 (quint, *J* = 7.2 Hz, 1H).

D. Set-up of the Csp^3 Cross-Coupling Reaction

Ligand Testing

To a stir bar-equipped 7 mL vial were added phosphine ligand (0.010 mmol, 10 mol%), Pd_2dba_3 (4.6 mg, 0.0050 mmol, 5 mol%), 4-bromobiphenyl **2a** (23.3 mg, 0.100 mmol, 1.00 eq), and Ag_2O (69.5 mg, 0.3 mmol, 3 eq). A dioxane solution of boronic acid **1a** (0.91 Molar, 0.220 mL, 0.200 mmol, 2.00 eq) was added by pipette. The vial was tightly sealed with a teflon-lined screw cap and stirred at 200 rpm at 85°C for 24 hours. Upon completion, the reaction mixture was filtered through a silica gel plug in a Pasteur pipette, rinsing with HPLC grade hexanes. The filtrate was collected in a 25 mL volumetric flask and diluted with hexanes up to the mark. After thorough mixing, an aliquot of this solution was transferred to an HPLC vial and immediately subjected to HPLC analysis (OD-H chiral column, 2.0 mL/min, isocratic 100% hexanes, 214.4 nm absorbance). On each new day of HPLC analysis, a standard solution of the branched product standard was analyzed in duplicate to confirm the bulb brightness and to adjust response factors if necessary. If peak retention times drifted, standards were repeated as necessary to confirm the identity of the peaks.

Substrate Table

The reaction was assembled as described above, except using 1.5 equivalents of Ag_2O (34.8 mg, 0.15 mmol). After 24 hours, the reactions were cooled and filtered through silica gel in a glass pipet, rinsing Et_2O or EtOAc. An aliquot of the crude reaction mixture was first subjected to HPLC analysis to determine the branched/linear product ratio, comparing with an authentic sample of the linear product isomer. The crude reaction was then purified by column chromatography, and then the enantiospecificity was determined by chiral HPLC.

E. Improved Synthesis and Recovery of the BIDA Ligand

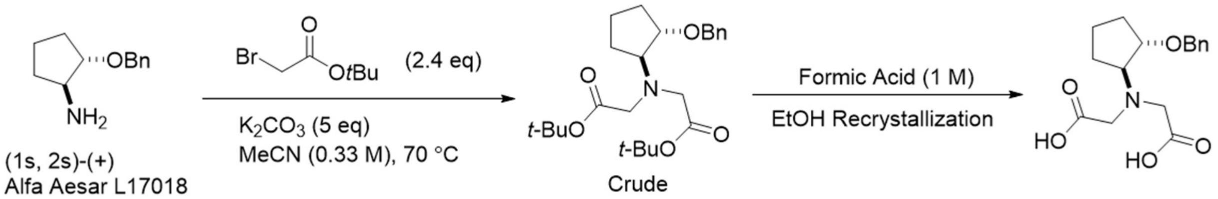

2,2'-(((1*S*,2*S*)-2-(benzyloxy)cyclopentyl)azanediyl)diacetic acid (BIDA). A stir bar-equipped, 3-neck 5 liter round bottom flask was fitted with a reflux condenser and thermometer. Under air, this flask was charged with K_2CO_3 (213 g, 1.54 mol, 5.00 eq), MeCN (500 mL), and (1*S*,2*S*)-(+)-2-benzyloxycyclopentylamine (58.8 g, 308 mmol, 1.00 eq). This mixture was cooled to 0°C, and a solution of *tert*-butyl bromoacetate (109 mL, 144 g, 740 mmol, 2.40 eq) in MeCN (250 mL) was added, rinsing with MeCN (for a total volume of 935 mL MeCN, 0.33 Molar). The mixture was stirred at 70°C for 24 hours.

The next day, the mixture was filtered through celite, rinsing with EtOAc. The filtrate was concentrated thoroughly *in vacuo* to afford a viscous oil. Using formic acid (310 mL, 378 g, 8.21 mol, 26.7 eq), the crude product was transferred to a stir bar equipped 3-neck 3 liter round bottom flask. The flask was equipped with a Vigreux condenser and heated at 85°C for 2 hours. An aliquot of the reaction was examined by NMR, confirming that the deprotection was complete.

The reaction was thoroughly concentrated *in vacuo* to remove all formic acid. The resulting viscous red oil was dissolved in hot EtOH (1250 mL), creating a super saturated solution. Soon after, a white powder began to crash out. The mixture was cooled to 0°C and filtered through a medium porosity glass frit, rinsing with additional cold EtOH. The product was dried *in vacuo* to afford an off-white powder (56.6 g, 184 mmol, 70% overall yield). Product characterization matched a previous report.[3]

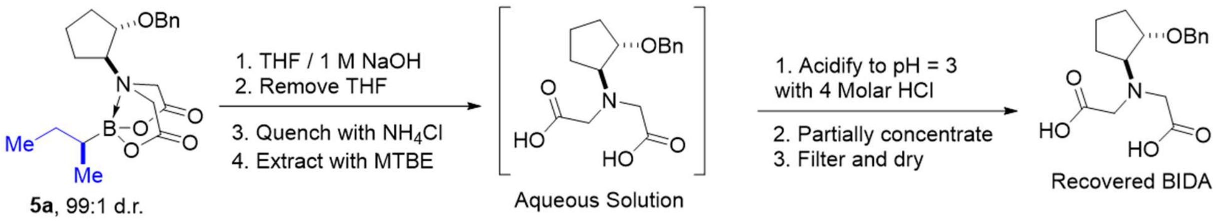

5a, 99:1 d.r.

BIDA Recovery. After hydrolysis of BIDA boronates and the preparation of sodium alkyltrihydroxyborate salts, the BIDA ligand could be recovered in 85% yield. Specifically, BIDA boronate **5a** (≥99:1 d.r., 0.933 g, 2.50 mmol) was converted to sodium alkyltrihydroxyborate salt **(*S*)-7a** by General Procedure B. After addition of saturated aqueous NH_4Cl and MTBE extraction, the aqueous phase was acidified to pH 3 with 4 Molar HCl and then concentrated by rotary evaporation with a 40°C bath until precipitation began. The solution was then stirred in an ice bath for one hour. Filtration through a medium porosity glass frit followed by drying on high vacuum afforded BIDA as a white solid (0.651 g, 2.12 mmol).

Synthesis and characterization of phosphine ligands

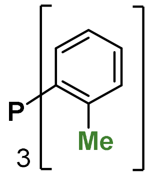

L2, σ_{para} = 0.00

Tris(2-methylphenyl)phosphine L2. This ligand was purchased from Sigma Aldrich (Product number 287822, Lot number MKBK8331V).

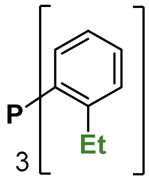

L3

Tris(2-ethylphenyl)phosphine L3. An oven dried, 50 mL 3-neck round bottom flask equipped with a stir bar was fitted with a thermometer adapter, a nitrogen inlet, and a rubber septum. The apparatus was vac-filled three times with nitrogen and charged with 1-bromo-2-ethylbenzene (0.55 mL, 740 mg, 4.0 mmol, 1.0 equiv.) and THF (6.5 mL, 1.6 Molar). The RBF was lowered into a dry ice/acetone bath and allowed to equilibrate for over 20 minutes. *n*butyllithium (1.6 Molar in hexanes, 2.4 mL, 3.8 mmol, 0.95 equiv.) was added dropwise over 10 minutes, keeping temperature below -60°C. The reaction appearance changed from colorless to green. Following the addition, the reaction mixture was allowed to stir for one hour and 50 minutes in the dry ice / acetone bath.

An oven dried 40 mL vial equipped with a stir bar was backfilled with nitrogen and charged with phosphorus trichloride (0.36 mL, 0.565 g, 4.12 mmol) and THF (8.0 mL; 0.515 Molar). 2.1 mL of the resulting PCl_3 solution (1.08 mmol, 0.27 eq) was transferred to the reaction mixture over 10 minutes (maintaining a temperature below -54°C). Following the addition, the reaction mixture was allowed to stir and warm to room temp overnight. The reaction mixture was cooled to 0°C in an ice / water bath and was quenched with 0.7mL water and 8mL NH_4Cl following equilibration. The quenched solution was then transferred to a separatory funnel, rinsing with water and toluene. After removing the organic layer, the aqueous layer was extracted with toluene (2 x 10 mL). Combined organics were washed with brine, dried over Na_2SO_4, filtered, and concentrated by rotary evaporation to afford a sticky white/yellow solid was allowed to dry overnight on high vac. The crude product was purified by normal phase column chromatography (4.5 x 9 cm silica gel column, isocratic 100% hexanes), affording the pure **L3** as a white solid (251.4 mg, 0.7256 mmol, 67% yield).

^{1}H NMR (500 MHz, acetone-*d6*) δ 7.30-7.23 (m, 6H), 7.05 (m, 3H), 6.70 (m, 3H), 2.76 (dq, *J* = 7.5, 1.2 Hz, 6H), 1.07 (t, *J* = 7.5 Hz, 9H).

^{13}C NMR (126 MHz, acetone-*d6*) δ 149.31 (d, J_{C-P} = 25.7 Hz), 135.60 (d, J_{C-P} = 11.6 Hz), 134.60, 130.06, 129.45 (d, J_{C-P} = 5.0 Hz), 126.96, 28.19 (d, J_{C-P} = 22.4 Hz), 15.69 (d, J_{C-P} = 3.0 Hz).

^{31}P NMR (202 MHz, acetone-*d6*, referenced to H_3PO_4 in D_2O) δ -35.62.

Rf = 0.31 on normal phase TLC in 100% hexanes

HRMS (ESI$^+$) Calculated for $C_{24}H_{28}P$ $(M+H)^+$: 347.1929, Found: 347.1926

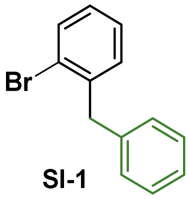

SI-1

1-benzyl-2-bromobenzene SI-1. This procedure was based on a previous report of the selective coupling of benzylic bromides.[4] While in an argon-filled atmosphere glovebox, $Pd(PPh_3)_4$ (0.6933 g, 0.600 mmol, 2 mol%) was massed out into a stir bar-equipped, 3-neck 500 mL round bottom flask. The flask was sealed with three septa, brought out into a fume hood, and then equipped with a reflux condenser attached to a nitrogen inlet. After the system was put under nitrogen, additional reagents and solvents were added by briefly removing a septum while under a positive nitrogen pressure. In this manner, ethanol (48 mL, 0.63 Molar), water (13 mL, 2.3 Molar), toluene (58 mL, 0.52 Molar), 2-bromobenzyl bromide (7.498 g, 30.0 mmol, 1.00 eq), phenylboronic acid (3.66 g, 30.0 mmol, 1.00 eq), and an aqueous solution of sodium carbonate (3.58 g, 33.8 mmol, 1.13 eq in 34 mL H_2O) were added to the reaction.

The reaction was heated to 80°C for 24 hours, cooled to room temperature, and then filtered through celite. After concentration, the crude material was diluted with H_2O and extracted with Et_2O. Combined organics were washed with brine, dried with Na_2SO_4, decanted, and reconcentrated. The product was purified by normal phase column chromatography (100% hexanes) followed by vacuum distillation using a kugelrohr, giving aryl bromide **SI-1** as a clear colorless oil (4.189 g, 16.95 mmol, 56% yield).

^1H NMR (500 MHz, $CDCl_3$) δ 7.57 (dd, *J* = 8.0, 1.3 Hz, 1H), 7.30 (t, *J* = 7.4 Hz, 2H), 7.25-7.18 (m, 4H), 7.14 (dd, *J* = 7.6, 1.7 Hz, 1H), 7.09 (td, *J* = 7.6, 1.7 Hz, 1H), 4.13 (s, 2H).

^{13}C NMR (126 MHz, $CDCl_3$) δ 140.50, 139.60, 132.98, 131.21, 129.13, 128.60, 128.01, 127.58, 126.38, 125.03, 41.87.

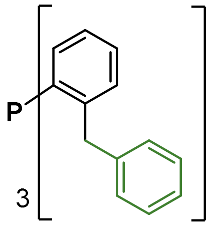

L4, σ_{para} = 0.00

Tris(2-benzyl-phenyl)phosphine L4. A flame dried, 250 mL, 1 neck round bottom flask was equipped with a stir bar, sealed with a septum, and put under nitrogen. To a separate, flame-dried 40 mL vial was added 2-benzyl-1-bromobenzene **SI-1** (4.19 g, 16.95 mmol, 1.00 eq). The vial put under nitrogen, and THF (28 mL total) was used to transfer the aryl bromide to the reaction flask, with rinsing for quantitative transfer. The solution was cooled by submerging in a dry ice / acetone bath. To the -78°C mixture was added *n*butyllithium (1.6 Molar in hexanes, 9.5 mL, 15.3 mmol, 0.90 eq) dropwise over 5-10 minutes, causing the reaction to turn a cloudy brownish/yellow. The reaction was allowed to stir for 1.5 hours at -78°C. Phosphorus trichloride (0.37 mL, 0.582 g, 4.24 mmol, 0.25 eq) was added neat in a dropwise manner over 2-3 minutes. The reaction was allowed to gradually warm to room temperature and stirred overnight.

After 12 hours, the reaction was quenched with NH_4Cl (40 mL). Water (80 mL) and DCM (120 mL) were added, and the aqueous layer was extracted (3x120 mL DCM). The combined organics were washed with brine (250 mL), dried (Na_2SO_4), decanted, and concentrated, giving a crude mixture consisting of a white solid and an oil. The crude product was purified by normal phase column chromatography (6 cm diameter, 500 mL SiO_2, isocratic 4/1 Hex/DCM), giving 1.96 grams of mostly pure product. This mixture was recrystallized from boiling hexanes (200-250 mL). After cooling to room temperature and then to 0°C, the product was filtered, giving **L4** as a white crystalline powder (1.385 grams, 2.60 mmol, 61% yield).

^{1}H NMR (500 MHz, $CDCl_3$) δ 7.25 (td, *J* = 7.3, 0.9 Hz, 3H), 7.14 (t, *J* = 7.5 Hz, 6H), 7.12-7.06 (m, 9H), 7.02 (dd, *J* = 7.0, 1.7 Hz, 6H), 6.82 (ddd, *J* = 7.7, 4.0, 1.4 Hz, 3H), 4.09 (s, 6H).

^{13}C NMR (126 MHz, $CDCl_3$) δ 145.78 (d, J_{C-P} = 26.3 Hz), 140.73, 134.99 (d, J_{C-P} = 11.7 Hz), 134.31, 130.13 (d, J_{C-P} = 5.0 Hz), 129.44, 129.06, 128.27, 126.66, 125.94, 40.23 (d, J_{C-P} = 22.3 Hz).

^{31}P NMR (202 MHz, $CDCl_3$, referenced to H_3PO_4 in D_2O) δ -31.42

HRMS (EI^+) Calculated for $C_{39}H_{33}P$ $(M)^+$: 532.23199, Found: 532.23141

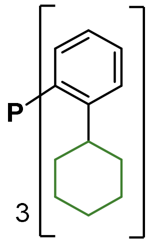

L5

Tris(2-cyclohexylphenyl)phosphine L5. *n*-butyllithium (1.6 Molar in hexanes, 0.625 mL, 1.00 mmol, 0.97 eq) was added to a -78°C solution of 1-bromo-2-cyclohexylbenzene (0.246 g, 1.03 mmol, 1.00 eq) in THF (2.0 mL). The reaction was allowed to stir for one hour at -78°C, and then phosphorus trichloride (29 µL, 45.4 mg, 0.332 mmol, 0.32 eq.) was added (as a 1.0 Molar solution, dropwise over five minutes). The reaction was allowed to continue stirring for one hour and then warmed to room temperature.

The reaction was quenched by addition of saturated NH_4Cl, and the aqueous layer was extracted with EtOAc. Combined organics were dried with Na_2SO_4 and concentrated *in vacuo*. The crude product was purified by normal phase column chromatography (isocratic 100% hexanes, R_f = 0.20), giving product as a white powder (70 mg, 0.138 mmol, 42% yield).

^{1}H NMR (500 MHz, $CDCl_3$) δ 7.30 (m, 6H), 7.03 (m, 3H), 6.81 (qdd, *J* = 3.8, 1.1, 0.6 Hz, 3H), 3.28 (tdt, *J* = 11.4, 7.7, 3.1 Hz, 1H), 1.77-1.63 (m, 15H), 1.43-1.31 (m, 6H), 1.28-1.15 (m, 9H).

^{13}C NMR (126 MHz, $CDCl_3$) δ 152.23 (d, *J* = 24.3 Hz), 135.53 (d, *J* = 11.2 Hz), 134.36, 128.90, 125.93 (d, *J* = 4.9 Hz), 125.88, 42.16 (d, *J* = 24.9 Hz), 34.27, 27.17, 26.42.

^{31}P NMR (202 MHz, $CDCl_3$, referenced to H_3PO_4 in D_2O) δ -37.11.

HRMS (ES^+)
Calculated for $C_{36}H_{46}P$: 509.3337
Found: 509.3343

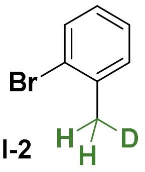

SI-2

SI-2. In an argon-filled glovebox, a dry 40 mL vial was charged with $LiAlD_4$ (84.0 mg, 2.00 mmol, 1.00 eq), followed by a stir bar. The vial was capped, brought out of the glovebox and into a fume hood, and put under nitrogen. Dry THF (1.0 mL) was added, and the mixture was cooled to 0°C in an ice/water bath. In a separate dry 40 mL vial under nitrogen, a solution was prepared of 2-bromobenzyl bromide (499.9 mg,

2.00 mmol, 1.00 eq) in dry THF (1.0 mL). This solution was then added to the $LiAlD_4$ suspension dropwise over two minutes, rinsing with THF (1.0 mL) for quantitative transfer. After 10 minutes of stirring at 0°C, the reaction was allowed to warm to room temperature. The sides of the vial were rinsed with an additional 1.0 mL THF.

After another 10 minutes, the reaction was worked up by the Fieser method.[5] Et_2O (5.3 mL) was added, and the reaction was cooled to 0°C. Deionized water (0.08 mL) was then added, followed by 15% NaOH (0.23 mL) and another portion of deionized water (0.08 mL). The mixture was then allowed to warm to room temperature and stir overnight. The next day, $MgSO_4$ was added to soak up the remaining water. After 15 minutes of stirring, the mixture was filtered through a 2 cm long SiO_2 plug, rinsing with pentane. The crude product was concentrated by rotary evaporation in a 0°C ice/water bath to retain the volatile product. This material was purified by normal phase silica gel chromatography using 100% pentane, followed by rotary evaporation in a 0°C ice/water bath to give pure product as a colorless oil of low viscosity (252.5 mg, 1.47 mmol, 73%).

^{1}H NMR (500 MHz, $CDCl_3$) δ 7.52 (dd, *J* = 7.9, 1.2 Hz, 1H), 7.25-7.18 (m, 2H), 7.04 (t, *J* = 7.8 Hz, 1H), 2.38 (app. t, *J* = 2.1 Hz, 2H).

^{13}C NMR (126 MHz, $CDCl_3$) δ 137.97, 132.46, 130.97, 127.45, 127.37, 125.08, 22.81 (app. t, $J_{C\text{-}D}$ = 19.7 Hz).

HRMS (EI^+) Calculated for C_7H_6BrD $(M)^+$: 170.97938, Found: 170.97946

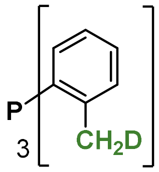

L6

L6. A dry, stir bar-equipped 25 mL recovery flask was put under nitrogen. Aryl bromide **SI-2** (224.0 mg, 1.30 mmol, 1.00 eq) was massed out in a separate dry 40 mL vial and put under nitrogen. Dry THF (1.0 mL) was added, and the solution of **SI-2** was transferred to the reaction flask, using 1.2 mL THF for quantitative transfer. This solution was cooled with stirring to -78°C in a dry ice / acetone bath. *n*butyllithium (1.6 M in hexanes, 0.73 mL, 1.17 mmol, 0.90 eq) was added dropwise over 5 minutes. The reaction appearance changed to cloudy and slightly off-white. After stirring for one hour and 45 minutes at -78°C, the reaction was treated with PCl_3 (neat, 28.3 µL, 44.6 mg, 0.325 mmol, 0.25 eq, dropwise over 3-4 minutes). The reaction, which had turned bright orange, was allowed to stir overnight, gradually warming to room temperature over four hours.

The reaction was quenched with saturated aqueous NH_4Cl (5 mL). The crude mixture was diluted with water (10 mL) and DCM (15 mL). The aqueous layer was extracted with DCM (3 x 15mL), and the combined organics were washed with brine (50 mL), dried with Na_2SO_4, decanted, and concentrated. The crude product was purified by normal phase column chromatography (2 cm diameter, 35 mL SiO_2, isocratic 20/1 hexanes/DCM), giving the pure product as a free-flowing white powder (62.7 mg, 0.204 mmol, 63% yield).

^{1}H NMR (500 MHz, $CDCl_3$) δ 7.29-7.21 (m, 6H), 7.08 (t, *J* = 7.2 Hz, 3H), 6.73 (ddd, *J* = 7.6, 4.6, 1.4 Hz, 3H), 2.38 (s, 6H).

^{13}C NMR (126 MHz, $CDCl_3$) δ 142.81 (d, J_{C-P} = 26.3 Hz), 134.55 (d, J_{C-P} = 10.6 Hz), 133.17, 130.19 (d, J_{C-P} = 4.8 Hz), 128.80, 126.29, 21.07 (dt, J_{C-P} = 21.4 Hz, J_{C-D} = 19.5 Hz).

^{31}P NMR (202 MHz, $CDCl_3$, referenced to H_3PO_4 in D_2O) δ -28.89

HRMS (EI^+) Calculated for $C_{21}H_{18}D_3P$ $(M)^+$: 307.15692, Found: 307.15628

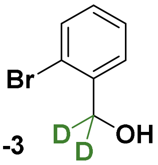

SI-3

SI-3. In an argon-filled glovebox, a flame-dried, stir bar-equipped, 3-neck 100 mL round bottom flask was charged with $LiAlD_4$ (420 mg, 10.0 mmol, 1.09 eq). The flask was sealed with a rubber septum, brought out of the glovebox and into a chemical fume hood, and put under nitrogen atmosphere. Dry THF (8 mL) was added, and the stirring suspension was cooled to 0°C. In a separate dry 40 mL vial under nitrogen, a solution was prepared of methyl 2-bromobenzoate (1.97 g, 9.16 mmol, 1.00 eq) and dry THF (3.5 mL). The solution was added to the $LiAlD_4$ suspension dropwise over 5 minutes.

After stirring for an hour at 0°C, the reaction was worked up by the Fieser method.[5] Et_2O (12 mL) was added. Deionized water (0.42 mL) was then added (dropwise over five minutes, causing bubbling), followed by 15% NaOH (0.42 mL) and another portion of deionized water (1.26 mL). The mixture was then allowed to warm to room temperature and stir overnight. The next day, $MgSO_4$ was added to soak up the remaining water. After 15 minutes of stirring, the mixture was filtered through celite, rinsing with Et_2O. After rotary evaporation, the crude product was purified by normal phase silica gel chromatography (3 cm diameter, 100 mL SiO_2, isocratic 3/1 pentane/Et_2O), giving the pure product as a fluffy white solid (1.563 g, 8.268 mmol, 90% yield).

^{1}H NMR (500 MHz, $CDCl_3$) δ 7.55 (dd, *J* = 7.9, 1.2 Hz, 1H), 7.48 (dd, *J* = 7.6, 1.7 Hz, 1H), 7.34 (td, *J* = 7.5, 1.2 Hz, 1H), 7.17 (ddd, *J* = 8.1, 7.3, 1.7 Hz, 1H), 1.93 (p, *J* = 1.0 Hz, 1H).

^{13}C NMR (126 MHz, $CDCl_3$) δ 139.77, 132.75, 129.31, 129.15, 127.80, 122.79, 64.61 (p, J_{C-D} = 22.3 Hz).

HRMS (EI^+) Calculated for $C_7H_5OBrD_2$ $(M)^+$: 187.98058, Found: 187.98046

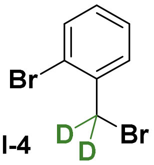

SI-4

SI-4. A flame-dried, stir bar-equipped 200 mL round bottom flask was charged with PPh_3 (2.75 g, 10.5 mmol, 1.50 eq) and imidazole (0.715 g, 10.5 mmol, 1.50 eq) and put under nitrogen. Dry DCM (14 mL) was added, and the stirring mixture was cooled to 0°C. Bromine (0.54 mL, 1.68 g, 10.5 mmol) was added dropwise over five minutes, using DCM to rinse the sides of the flask. The reaction was allowed to warm

to room temperature, stirred for an additional 10 minutes, and then cooled to 0°C again. A solution of the benzylic alcohol **SI-3** (1.32 g, 7.00 mmol, 1.00 eq) in dry DCM (10 mL) was added dropwise over five minutes, causing a precipitate to form. Additional DCM (2.0 mL) was used to rinse for quantitative transfer of the alcohol. The reaction was allowed to warm to room temperature and stir for an additional 40 minutes.

At this point, the stir bar was removed, and the crude reaction was concentrated by rotary evaporation. Pentane (20 mL) was added to the crude product, and it was again concentrated. The crude material was purified by normal phase column chromatography (6 cm diameter, 400 mL SiO_2, isocratic 100% pentane), giving the pure product as a colorless oil of low viscosity (981 mg, 3.89 mmol, 56% yield).

^{1}H NMR (500 MHz, $CDCl_3$) δ 7.58 (dd, J = 8.0, 1.2 Hz, 1H), 7.46 (dd, J = 7.6, 1.7 Hz, 1H), 7.30 (td, J = 7.5, 1.2 Hz, 1H), 7.17 (td, J = 7.7, 1.7 Hz, 1H).

^{13}C NMR (126 MHz, $CDCl_3$) δ 137.07, 133.49, 131.38, 130.25, 128.08, 124.60, 33.09 (p, $J_{C\text{-}D}$ = 23.6 Hz).

HRMS (EI$^+$) Calculated for $C_7H_4Br_2D_2$ $(M)^+$: 249.8962, Found: 249.8961

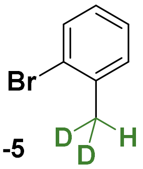

SI-5

SI-5. To a dry 40 mL vial was added $LiAlH_4$ (56.9 mg, 1.50 mmol, 1.00 eq), followed by a stir bar. The vial was capped and put under nitrogen. Dry THF (1.0 mL) was added, and the stirring suspension was cooled to 0°C with an ice/water bath. In a separate dry 40 mL vial under nitrogen, a solution of benzylic bromide **SI-4** (379 mg, 1.50 mmol, 1.00 eq) was prepared in dry THF (1.0 mL). This solution was added to the $LiAlH_4$ vial dropwise over two minutes, using THF (1.0 mL) for quantitative transfer and THF (1.0 mL) to rinse the sides of the reaction vial.

After another 10 minutes of stirring, the reaction was worked up according the Fieser method.[5] Et_2O (4.0 mL) was added. Deionized water (0.06 mL) was then added, followed by 15% NaOH (0.17 mL) and another portion of deionized water (0.06 mL). The mixture was then allowed to warm to room temperature and stir overnight. The next day, $MgSO_4$ was added to soak up the remaining water. After 15 minutes of stirring, the mixture was filtered through a 2 cm long SiO_2 plug, rinsing with pentane. The filtrate was concentrated by rotary evaporation in a 0°C ice/water bath to retain the volatile product, which was isolated without further purification as a colorless oil of low viscosity (250.6 mg, 1.44 mmol, 96%).

^{1}H NMR (500 MHz, $CDCl_3$) δ 7.52 (dd, J = 7.9, 1.2 Hz, 1H), 7.25-7.18 (m, 2H), 7.04 (m, 1H), 2.37 (p, J = 2.4 Hz, 1H).

^{13}C NMR (126 MHz, $CDCl_3$) δ 137.94, 132.47, 130.97, 127.46, 127.37, 125.09, 22.52 (p, J = 19.6 Hz).

HRMS (EI$^+$) Calculated for $C_7H_5BrD_2$ $(M)^+$: 171.9857, Found: 171.9861

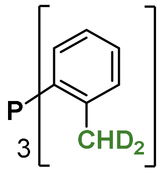

L7

L7. A dry, stir bar-equipped 25 mL recovery flask was put under nitrogen. Aryl bromide **SI-5** (207.7 mg, 1.20 mmol, 1.00 eq) was massed out in a separate dry 40 mL vial and put under nitrogen. Dry THF (1.0 mL) was added, and the solution of **SI-5** was transferred to the reaction flask, using 1.0 mL THF for quantitative transfer. This solution was cooled with stirring to -78°C in a dry ice / acetone bath. *n*butyllithium (1.6 M in hexanes, 0.675 mL, 1.08 mmol, 0.90 eq) was added dropwise over 5 minutes. The reaction appearance changed to cloudy and slightly off-white. After stirring for one hour and 30 minutes at -78°C, the reaction was treated with PCl_3 (neat, 26.2 µL, 41.2 mg, 0.300 mmol, 0.25 eq, dropwise over 3-4 minutes). The reaction, which had turned bright orange, was allowed to stir overnight, gradually warming to room temperature over four hours.

The reaction was quenched with saturated aqueous NH_4Cl (5 mL). The crude mixture was diluted with water (10 mL) and DCM (15 mL). The aqueous layer was extracted with DCM (3 x 15mL), and the combined organics were washed with brine (50 mL), dried with Na_2SO_4, decanted, and concentrated. The crude product was purified by normal phase column chromatography (isocratic 100% hexanes), giving the pure product as a free-flowing white powder (53.0 mg, 0.169 mmol, 56% yield).

^1H NMR (500 MHz, $CDCl_3$) δ 7.28-7.20 (m, 6H), 7.08 (t, *J* = 7.6 Hz, 3H), 6.72 (ddd, *J* = 7.6, 4.3, 1.3 Hz, 1H), 2.36 (s, 1H).

^{13}C NMR (126 MHz, $CDCl_3$) δ 142.80 (d, J_{C-P} = 26.2 Hz), 134.64 (d, J_{C-P} = 10.8 Hz), 133.20, 130.19 (d, J_{C-P} = 4.8 Hz), 128.79, 126.29, 20.79 (dp, J_{C-P} = 21.2 Hz, J_{C-D} = 19.4 Hz).

^{31}P NMR (202 MHz, $CDCl_3$, referenced to H_3PO_4 in D_2O) δ -28.90.

HRMS (EI^+) Calculated for $C_{21}H_{15}D_6P$ $(M)^+$: 310.1757, Found: 310.1761

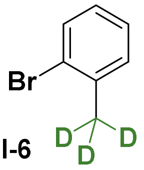

SI-6

SI-6. To a dry 40 mL vial was added $LiAlD_4$ (63.0 mg, 1.50 mmol, 1.01 eq), followed by a stir bar. The vial was capped and put under nitrogen. Dry THF (1.0 mL) was added, and the stirring suspension was cooled to 0°C with an ice/water bath. In a separate dry 40 mL vial under nitrogen, a solution of benzylic bromide **SI-4** (373 mg, 1.48 mmol, 1.00 eq) was prepared in dry THF (1.0 mL). This solution was added to the $LiAlD_4$ vial dropwise over two minutes, using THF (1.0 mL) for quantitative transfer and THF (1.0 mL) to rinse the sides of the reaction vial.

After another 10 minutes of stirring, the reaction was worked up according the Fieser method.[5] Et_2O (4.0 mL) was added. Deionized water (0.06 mL) was then added, followed by 15% NaOH (0.17 mL) and another portion of deionized water (0.06 mL). The mixture was then allowed to warm to room temperature and stir overnight. The next day, $MgSO_4$ was added to soak up the remaining water. After 15 minutes of stirring, the mixture was filtered through a 2 cm long SiO_2 plug, rinsing with pentane. The filtrate was concentrated by rotary evaporation in a 0°C ice/water bath to retain the volatile product, which was isolated without further purification as a colorless oil of low viscosity (238.2 mg, 1.368 mmol, 93% yield).

^{1}H NMR (500 MHz, $CDCl_3$) δ 7.52 (dt, J = 8.0, 1.0 Hz, 3H), 7.24-1.18 (m, 6H), 7.04 (m, 3H).

^{13}C NMR (126 MHz, $CDCl_3$) δ 137.91, 132.47, 130.98, 127.47, 127.37, 125.09.

HRMS (EI$^+$) Calculated for $C_7H_4BrD_3$ $(M)^+$: 172.9919, Found: 172.9923

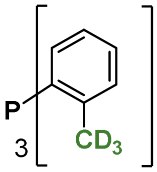

L8

L8. A dry, stir bar-equipped 25 mL recovery flask was put under nitrogen. Aryl bromide **SI-6** (208.9 mg, 1.20 mmol, 1.00 eq) was massed out in a separate dry 40 mL vial and put under nitrogen. Dry THF (1.0 mL) was added, and the solution of **SI-6** was transferred to the reaction flask, using 1.0 mL THF for quantitative transfer. This solution was cooled with stirring to -78°C in a dry ice / acetone bath. *n*butyllithium (1.6 M in hexanes, 0.675 mL, 1.08 mmol, 0.90 eq) was added dropwise over five minutes. The reaction appearance changed to cloudy and slightly off-white. After stirring for one hour and 30 minutes at -78°C, the reaction was treated with PCl_3 (neat, 26.2 µL, 41.2 mg, 0.300 mmol, 0.25 eq, dropwise over two minutes). The reaction, which had turned bright orange, was allowed to stir overnight, gradually warming to room temperature over four hours.

The reaction was quenched with saturated aqueous NH_4Cl (5 mL). The crude mixture was diluted with water (10 mL) and DCM (15 mL). The aqueous layer was extracted with DCM (3 x 15mL), and the combined organics were washed with brine (50 mL), dried with Na_2SO_4, decanted, and

concentrated. The crude product was purified by normal phase column chromatography (2 cm diameter, 50 mL SiO_2, isocratic 100% hexanes), giving the pure product as a free-flowing white powder (53.0 mg, 0.169 mmol, 56% yield).

^{1}H NMR (500 MHz, $CDCl_3$) δ 7.28-7.20 (m, 6H), 7.08 (td, *J* = 7.3, 1.5 Hz, 1H), 6.72 (ddd, *J* = 7.8, 4.4, 1.3 Hz, 1H).

^{13}C NMR (126 MHz, $CDCl_3$) δ 142.75 (d, J = 25.5 Hz), 134.61 (d, J_{C-P} = 10.8 Hz), 133.17 , 130.18 (d, *J* = 4.7 Hz), 128.79, 126.29.

^{31}P NMR (202 MHz, $CDCl_3$, referenced to H_3PO_4 in D_2O) δ -28.87.

HRMS (EI$^+$) Calculated for $C_{21}H_{12}D_9P$ (M)$^+$: 313.1946, Found: 313.1949

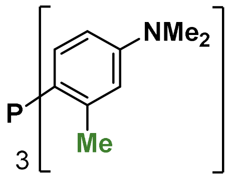

L10, σ_{para} = -0.83

Tris(2-methyl-4-dimethylaminophenyl)phosphine L10. This procedure was based on previously reported synthesis of this compound.[6] A dry, stir bar-equipped 50 mL 3-neck round bottom flask was fitted with a reflux condenser and put under nitrogen. *N*,*N*,3-trimethylaniline (1.45 mL, 1.352 g, 10.0 mmol, 3.03 eq) was added, followed by pyridine (5.0 mL), resulting in a clear, homogeneous, slightly yellow solution. The mixture was cooled to 0°C, and phosphorus tribromide (0.31 mL, 0.901 g, 3.33 mmol, 1.00 eq) was added in a neat fashion dropwise over three or four minutes. The reaction immediately changed to a yellow color and precipitate began to gradually form. After five minutes of stirring at 0°C, the reaction was heated to 125°C for one hour and then cooled to room temperature.

The crude reaction was diluted in benzene (50 mL) and washed with 6 N NaOH (20 mL), H_2O (20 mL), and brine (20 mL). The organic layer was dried with Na_2SO_4, decanted, and concentrated on strong vacuum to remove all solvent. The crude material was then transferred to a small round bottom flask (50 mL) and dissolved in degassed acetone. After heating to boiling and stirring, a white powder was present that still would not dissolve. The mixture was cooled to room temperature and filtered through a medium porosity glass frit. The filtrate was reconcentrated and triturated again, and this process was repeated once more. The combined crystals were triturated with acetone and filtered again, this time under nitrogen. The crystalline product **L10** was crushed to a white powder and stored under in an argon-filled glovebox (0.233 g, 0.537 mmol, 16% yield).

^{1}H NMR (500 MHz, C_6D_6) δ 7.19 (dd, *J* = 8.5, 4.2 Hz, 3H), 6.63 (app. t, *J* = 3.4 Hz, 3H), 6.40 (dd, *J* = 8.5, 2.7 Hz, 3H), 2.69 (s, 9H), 2.51 (s, 18H).

^{13}C NMR (126 MHz, $CDCl_3$) δ 150.54, 143.32 (d, J_{C-P} = 26.6 Hz), 134.36, 122.10 (d, J_{C-P} = 6.3 Hz), 114.10 (d, J_{C-P} = 5.0 Hz), 110.37, 40.46, 21.85 (d, J_{C-P} = 21.0 Hz).

^{31}P NMR (202 MHz, C_6D_6, referenced to H_3PO_4 in D_2O) δ -34.90.

HRMS (ESI^+) Calculated for $C_{27}H_{37}N_3P$ $(M+H)^+$: 434.2725, Found: 434.2714

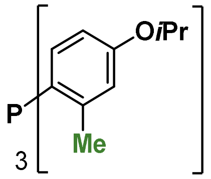

L11, σ_{para} = -0.45

Tris(4-isopropoxy-2-methylphenyl)phosphine L11. A solution of 1-bromo-4-isopropoxy-2-methylbenzene (0.6874 g, 3.00 mmol, 1.00 eq) in dry THF (5.0 mL) in a three-neck, 100 mL round bottom flask was cooled to -78°C before adding *n*butyllithium (1.6 Molar in hexanes, 1.84 mL, 2.94 mmol, 0.98 eq) dropwise over five minutes. The reaction was allowed to stir for 1 hour and 45 minutes at -78°C, resulting in a cloudy, orange/sherbet-colored suspension. A solution of phosphorus trichloride (78 µL, 0.124 g, 0.90 mmol, 0.30 eq) in THF (2.0 mL) was added to the reaction dropwise over about five minutes. The resulting clear, homogeneous orange-colored reaction was stirred for 40 minutes at -78°C and then allowed to warm to room temperature.

The reaction was quenched with saturated NH_4Cl (10 mL), and diluted with H_2O (20 mL) and DCM (30 mL). The aqueous layer was extracted with DCM (3x30 mL). The combined organics were washed with brine (100 mL), dried ($MgSO_4$), filtered, and concentrated to afford a viscous yellow oil. The crude product was purified by normal phase column chromatography (5 cm diameter, 200 mL silica gel, isocratic 30/1 Hex/EtOAc), giving **L11** as a white powder (0.1876 g, 0.3920 mmol, 44% yield).

^{1}H NMR (500 MHz, C_6D_6) δ 7.08 (dd, *J* = 8.4, 4.0 Hz, 3H), 6.88 (dd, *J* = 4.0, 2.6 Hz, 3H), 6.59 (dd, *J* = 8.4, 2.6 Hz, 3H), 4.20 (sext, J = 6.0 Hz, 3H), 2.50 (s, 9H), 1.11 (d, J = 6.0 Hz, 18H).

^{13}C NMR (126 MHz, $CDCl_3$) δ 158.44, 144.20 (d, J_{C-P} = 27.4 Hz), 134.56, 126.11 (d, J_{C-P} = 8.3 Hz), 117.77 (d, *J* = 5.1 Hz), 112.99 (d, *J* = 0.9 Hz), 69.61, 22.30, 21.49 (d, *J* = 21.4 Hz).

^{31}P NMR (202 MHz, C_6D_6, referenced to H_3PO_4 in D_2O) δ -33.72

Rf = 0.20 on normal phase TLC in 20/1 Hex/EtOAc

HRMS (ESI^+) Calculated for $C_{30}H_{40}O_3P$ $(M+H)^+$: 479.2715, Found: 479.2710

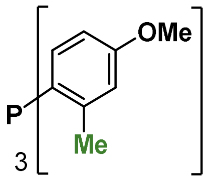

L12, σ_{para} = -0.268

Tris(4-methoxy-2-methylphenyl)phosphine L12. This compound has been previously synthesized and characterized.[7] A dry, stir bar-equipped 100 mL recovery flask was charged with magnesium turnings (0.3975 g, 16.35 mmol, 1.01 eq) in an argon-filled glovebox. The flask was sealed with a septum,

brought out into the hood, equipped with a reflux condenser, attached to a Schlenk line, and put under nitrogen. Dry THF (8 mL) was added, followed by 1-bromo-4-methoxy-2-methylbenzene (2.29 mL, 3.26 g, 16.2 mmol, 1.00 eq) dropwise over eight minutes. An additional 8 mL of dry THF was added. The reaction began to reflux without any external heat or initiating agent. In a separate round bottom flask, a solution of phosphorus trichloride (0.44 mL, 0.69 g, 5.0 mmol, 0.31 eq) in dry THF (14 mL) was prepared under nitrogen. After the Grignard had turned to a cloudy grey and most of the magnesium was gone (about one hour), the solution of phosphorus trichloride was added at 0°C dropwise over 10 minutes. The reaction was allowed to warm to room temperature and stirred overnight.

The next day, the reaction was quenched with addition of saturated NH_4Cl (40 mL) and diluted with H_2O (80 mL) and toluene (100 mL). The aqueous layer was extracted with toluene (3x100 mL), and the combined organics were washed with brine, dried with $MgSO_4$, filtered, and concentrated. The crude product was purified by column chromatography (4 cm diameter, 130 mL silica gel, 30/1 Hex/EtOAc), giving **L12** as a white powder (0.4652 g, 1.179 mmol, 24% yield).

^{1}H NMR (500 MHz, $CDCl_3$) δ 6.78 (m, 3H), 6.65-6.62 (m, 6H), 3.79 (s, 9H), 2.36 (s, 9H).

^{13}C NMR (126 MHz, $CDCl_3$) δ 160.13, 144.23 (d, $J_{C\text{-}P}$ = 27.6 Hz), 134.53, 126.37 (d, $J_{C\text{-}P}$ = 8.5 Hz), 116.02 (d, $J_{C\text{-}P}$ = 5.2 Hz), 111.60 55.20, 21.49 (d, $J_{C\text{-}P}$ = 21.4 Hz).

^{31}P NMR (202 MHz, C_6D_6, referenced to H_3PO_4 in D_2O) δ -33.95

HRMS (EI$^+$) Calculated for $C_{24}H_{27}O_3P$ $(M)^+$: 394.16979, Found: 394.16897

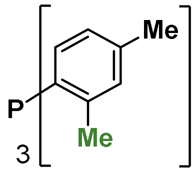

L13, σ_{para} = -0.170

Tris(2,4-dimethylphenyl)phosphine L13. This ligand was purchased from Sigma-Aldrich (Product number 710547, Lot number MKBB9858V).

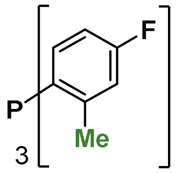

L14, σ_{para} = +0.062

Tris(4-fluoro-2-methylphenyl)phosphine L14. A dry, stir bar-equipped 25 mL three neck round bottom flask was charged with magnesium turnings (0.1535 g, 6.314 mmol, 1.05 eq) in an argon-filled glovebox. The flask was sealed with septa, brought out into the hood, equipped with a reflux condenser, attached to a Schlenk line, and put under nitrogen. To a separate 10 mL pear flask under nitrogen was added 1-

bromo-4-fluoro-2-methylbenzene (0.76 mL, 1.136 g, 6.011 mmol, 1.00 eq) and dry THF (6.0 mL). This solution was added in a dropwise fashion to the magnesium-containing flask, causing initiation of the Grignard reaction. After about 1.5 hours, the reaction had turned cloudy and a brownish/grey color and cooled to room temperature. The Grignard was added to a -78°C solution of phosphorus trichloride (0.155 mL, 0.243 g, 1.77 mmol, 0.295 eq) in THF (5.0 mL) in a 50 mL pear flask. The reaction was allowed to gradually warm to room temperature and stir overnight.

The next day, the reaction was cooled to 0°C and quenched with addition of saturated NH_4Cl (20 mL) and diluted with H_2O (10 mL) and toluene (30 mL). The aqueous layer was extracted with toluene (3x30 mL), and the combined organics were washed with brine, dried with $MgSO_4$, filtered, and concentrated. The crude product was purified by normal phase column chromatography (2.5 cm diameter, 40 mL silica gel, isocratic 3/1 Hex/DCM, then a second column of same dimensions with isocratic 100% hexanes), giving **L14** as a white powder (0.2611 g, 0.7286 mmol, 41% yield).

^{1}H NMR (500 MHz, $CDCl_3$) δ 6.96 (dt, J = 9.8, 3.3 Hz, 3H), 6.80 (td, J = 8.5, 2.7 Hz, 3H), 6.64 (ddd, J = 8.6, 6.3, 3.7 Hz, 3H), 2.36 (s, 9H).

^{13}C NMR (126 MHz, $CDCl_3$) δ 163.58 (d, J_{C-F} = 248.5 Hz), 145.35 (dd, J = 28.4, 7.8 Hz), 134.83 (d, J = 8.1 Hz), 129.66 (dd, J = 10.6, 3.2 Hz), 117.45 (dd, J = 20.7, 5.1 Hz), 113.45 (d, J = 20.2 Hz), 21.31 (dd, J_{C-P} = 21.8, J_{C-F} 1.6 Hz).

^{31}P NMR (202 MHz, C_6D_6, referenced to H_3PO_4 in D_2O) δ -33.00 (q, J = 3.5 Hz).

^{19}F NMR (470 MHz, $CDCl_3$, referenced to $CFCl_3$ in $CDCl_3$) δ -114.60 (q, J = 8.3 Hz).

HRMS (EI^+) Calculated for $C_{21}H_{18}F_3P$ $(M)^+$: 358.10982, Found: 358.10935

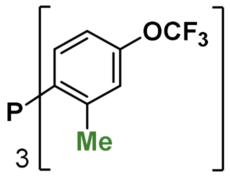

L15, σ_{para} = +0.35

Tris(2-methyl-4-(trifluoromethoxy)phenyl)phosphine L15. Caution: the synthesis of these ligands through the arylmagnesium species is potentially hazardous.[8] A solution of 1-bromo-2-methyl-4-(trifluoromethoxy)benzene (0.82 mL, 1.28 g, 5.00 mmol, 1.00 eq) in dry THF (8.0 mL) in a three-neck, 100 mL round bottom flask was cooled to -78°C before adding *n*butyllithium (1.6 Molar in hexanes, 2.97 mL, 4.75 mmol, 0.95 eq). The resulting homogeneous, clear, slightly orange solution was allowed to stir for 1.5 hours at -78°C. A solution of phosphorus trichloride (125 µL, 0.196 g, 1.43 mmol, 0.285 eq) in THF (3.0 mL) was added to the reaction dropwise over about three minutes. The resulting clear, homogeneous orange/red-colored reaction was stirred for five hours at -78°C and then allowed to warm to room temperature.

The reaction was quenched with saturated NH_4Cl (10 mL), and diluted with H_2O (20 mL) and toluene (30 mL). The aqueous layer was extracted with toluene (3x30 mL). The combined organics were washed with brine (100 mL), dried ($MgSO_4$), filtered, and concentrated. The crude product was purified

by normal phase column chromatography (4 cm diameter, 150 mL silica gel, isocratic 100% hexanes), giving **L15** as a white powder (0.4389 g, 0.7889 mmol, 55% yield).

^{1}H NMR (500 MHz, $CDCl_3$) δ 7.11 (app. s, 3H), 6.97 (d, *J* = 8.4 Hz, 3H), 6.69 (dd, *J* = 8.4, 3.7 Hz, 3H), 2.39 (s, 9H).

^{13}C NMR (126 MHz, $CDCl_3$) δ 150.22 (q, J_{C-F} = 1.8 Hz), 145.10 (d, J_{C-P} = 28.3 Hz), 134.52, 132.25 (d, J_{C-P} = 11.5 Hz), 122.53 (d, J_{C-P} = 5.0 Hz), 120.59 (q, J_{C-F} = 258.0 Hz), 118.53, 21.39 (d, J_{C-P} = 21.7 Hz).

^{31}P NMR (202 MHz, C_6D_6 referenced to H_3PO_4 in D_2O) δ -32.12

^{19}F NMR (470 MHz, C_6D_6, referenced to $CFCl_3$ in $CDCl_3$) δ -57.79

Rf = 0.29 on normal phase TLC in 100% hexanes

HRMS (ESI$^+$) Calculated for $C_{24}H_{19}O_3F_9P$ $(M+H)^+$: 557.0928, Found: 557.0918

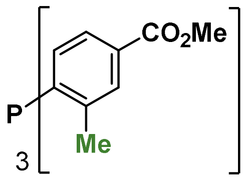

L16, σ_{para} = +0.45

Trimethyl 4,4',4''-phosphanetriyltris(3-methylbenzoate) L16. This synthesis was based on a procedure previously reported for the corresponding phosphine lacking the *ortho*-methyl groups.[9] To a dry, 100 mL 3-neck round bottom flask were added $CoBr_2$ (0.1094 g, 0.50 mmol, 0.10 eq), zinc (1.092 g, 16.7 mmol, 3.33 eq), and $ZnBr_2$ (0.1126 g, 0.50 mmol, 0.10 eq). The mixture was crushed to a fine powder and then heated at 160°C for two hours on high vac while the stir bar agitated the powder. Upon cooling to room temperature, MeCN (5.0 mL) was added, resulting in a blue suspension. Trifluoroacetic acid (17 µL, 25 mg, 0.22 mmol, 4.4 mol%) was added, resulting in a grey suspension.

Separately, a solution of methyl 4-bromo-3-methylbenzoate (1.145 g, 5.00 mmol, 1.00 eq) in MeCN (3.0 mL) was prepared under nitrogen in a 10 mL recovery flask. A small amount of the aryl bromide solution (about 0.2 mL) was added to the zinc suspension and allowed to stir for 25 minutes at room temperature. The remainder of the aryl bromide solution was then added (dropwise over five minutes at room temperature, with rinsing 2x0.5 mL of MeCN for quantitative transfer).

After stirring at room temperature for 1 hour and 35 minutes, the reaction was monitored by NMR. A small aliquot (0.2 mL) was removed via needle and added to a solution of iodine in pentane. The vial was capped and shaken, and then 3 mL of saturated $Na_2S_2O_3$ was added. The organic layer was removed, concentrated, and analyzed by ^{1}H-NMR in C_6D_6. No aryl bromide remained (complete conversion). There was approximately 75% of the aryl iodide and 25% protodehalogenated side product.

The arylzinc solution was filtered by the following procedure. First, a dry, stir bar-equipped 100 mL Schlenk flask was put under nitrogen. The arylzinc was drawn into a 24 mL syringe through a needle.

The needle was then quickly removed and replaced with a dry 0.2 micron HPLC filter with a needle on the end. The arylzinc was pushed through the filter and the needle into the receiving Schlenk flask.

To this room temperature stirring solution was added phosphorus trichloride (110 µL, 0.173 g, 1.25 mmol, 0.33 eq relative to the arylzinc as read out by NMR yield of the aryl iodide). During the addition, the reaction changed from an orange homogeneous solution to a yellow cloudy suspension. After the addition was complete, the reaction was heated to 45°C with vigorous stirring.

After one hour, the reaction was monitored by NMR. A small aliquot was removed, quenched by 1 M HCl, extracted with DCM, and concentrated. Only traces of product had formed, with most material converted to the protodehalogenated side product. The reaction was then heated to 65°C and allowed to stir overnight.

After 13 hours, the reaction was again monitored by NMR. Two aliquots were removed. One was quenched with 1 M HCl (to check for product formation), and the other was quenched with iodine (to check for consumption of the arylzinc reagent). By NMR analysis, there was a 5:5:1 ratio of arylzinc / protodehalogenated side product / triarylphosphine product. To accelerate the reaction by enhancing the nucleophilicity of the arylzinc reagent,[10,11] anhydrous lithium bromide beads (0.436 g, 5.00 mmol, 1.00 eq relative to original ArBr) in THF was added in one portion. The reaction changed from a pale, cream-colored suspension to an opaque, green/blue suspension. After another hour, the reaction was monitored by NMR using the same dual aliquot procedure. It was approximately 3:3:1 arylzinc / protodehalogenated side product / triarylphosphine product. The reaction was allowed to continue stirring at 65°C for another three hours and then cooled to room temperature. The reaction was quenched with 1 Molar HCl (30 mL) and extracted with DCM (3x30 mL). The combined organics were washed with H_2O (100 mL) and brine (100 mL), dried with Na_2SO_4, decanted, and concentrated. The crude material was dried on high vac overnight.

The next day, the crude reaction was purified by normal phase column chromatography (6 cm diameter, 200 mL silica gel, isocratic 6/1 Hex/EtOAc, R_f = 0.16), giving **L16** as a white solid (64.6 mg, 0.135 mmol, 11% yield).

^{1}H NMR (500 MHz, C_6D_6) δ 8.00 (ddd, *J* = 4.7, 1.1, 0.5 Hz, 3H), 7.81 (ddd, *J* = 7.9, 1.2, 0.4 Hz, 3H), 6.86 (dd, J = 7.9, 3.9 Hz, 3H), 3.50 (s, 9H), 2.24 (s, 9H).

^{13}C NMR (126 MHz, $CDCl_3$) δ 167.10, 143.19 (d, $J_{C\text{-}P}$ = 26.5 Hz), 139.42 (d, $J_{C\text{-}P}$ = 12.8 Hz), 133.11, 131.18 (d, $J_{C\text{-}P}$ = 4.9 Hz), 131.00, 127.34, 52.37, 21.30 (d, $J_{C\text{-}P}$ = 21.1 Hz).

^{31}P NMR (202 MHz, C_6D_6 referenced to H_3PO_4 in D_2O) δ -27.25.

HRMS (ESI$^+$) Calculated for $C_{27}H_{28}O_6P$ $(M+H)^+$: 479.1624, Found: 479.1614

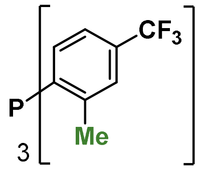

L17, σ_{para} = +0.54

Tris(2-methyl-4-(trifluoromethyl)phenyl)phosphine L17. Caution: the synthesis of these ligands through the arylmagnesium species is potentially hazardous.[8] A dry, stir bar-equipped, 50 mL 3-neck round bottom flask was put under nitrogen before adding 1-bromo-2-methyl-4-(trifluoromethyl)benzene (1.21 g, 5.00 mmol, 1.00 eq) and dry THF (8.3 mL). The stirring solution as cooled to -78°C in a dry ice / acetone bath. *n*butyllithium (1.6 Molar in hexanes, 3.1 mL, 4.96 mmol, 0.99 eq) was added dropwise over 10 minutes, resulting in a green/grey solution. Over the next hour of stirring at -78°C, the reaction color changed from green to yellow, to orange, and finally to dark red. After one hour and 15 minutes, a solution of phosphorus trichloride (0.131 mL, 0.206 g, 1.50 mmol, 0.30 eq) in THF (2.5 mL) was added dropwise over five minutes. The resulting dark red-colored reaction was allowed to stir and gradually warm to room temperature over the next seven hours.

The reaction was cooled to 0°C and quenched by addition of H_2O (0.8 mL) while under nitrogen. The septum was then removed, and saturated NH_4Cl (10 mL) was added. The reaction was diluted using H_2O (20 mL) and toluene (20 mL). After extraction of the aqueous layer (3x30 mL toluene), the combined organics were washed with brine (100 mL), dried ($MgSO_4$), filtered, and concentrated to afford a mixture of a yellow oil and white crystalline product. The crude reaction was purified by normal phase column chromatography (150 mL silica gel, isocratic 100% hexanes). The ligand was further purified via recrystallization by dissolving in a minimal amount of boiling MeOH, cooling to 0°C, and filtering, giving **L17** a white crystalline product (0.2615 g, 0.5144 mmol, 34% yield).

^{1}H NMR (500 MHz, C_6D_6) δ 7.27 (d, *J* = 3.9 Hz, 3H), 7.01 (d, *J* = 8.2 Hz, 3H), 6.54 (dd, *J* = 8.0, 3.8 Hz, 3H), 2.05 (s, 9H).

^{13}C NMR (126 MHz, $CDCl_3$) δ 143.80 (d, $J_{C\text{-}P}$ = 27.3 Hz), 137.83 (d, $J_{C\text{-}P}$ = 12.9 Hz), 133.39, 131.64 (q, $J_{C\text{-}F}$ = 32.3 Hz), 127.12 app. quint, *J* = 3.9 Hz), 124.12 (q, $J_{C\text{-}F}$ = 272.8 Hz), 123.30 (q, $J_{C\text{-}F}$ = 3.8 Hz), 21.39 (d, *J* = 21.3 Hz).

^{31}P NMR (202 MHz, C_6D_6 referenced to H_3PO_4 in D_2O) δ -28.47.

^{19}F NMR (470 MHz, C_6D_6 referenced to $CFCl_3$ in $CDCl_3$) δ -62.99.

HRMS (EI$^+$) Calculated for $C_{24}H_{18}F_9P$ $(M)^+$: 508.10021, Found: 508.09941

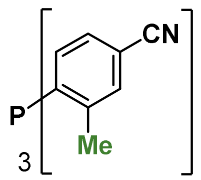

L18, σ_{para} = +0.660

Tris(2-methyl-4-cyanophenyl)phosphine L18. To a dry 50 mL, 3-neck, stir bar-equipped round bottom flask was added 4-bromo-3-methylbenzonitrile (0.9803 g, 5.00 mmol, 1.00 eq). The aryl bromide was put under nitrogen, dry THF (5.0 mL) was added, and the stirring solution was cooled to 0°C in an ice/water bath. *i*PrMgCl•LiCl (1.3 Molar in THF, 3.85 mL, 5.00 mmol, 1.00 eq) was added (dropwise over 20 minutes), causing the reaction appearance to change to a cloudy, dark yellow. The reaction was allowed to continue stirring at 0°C. The reaction was monitored by NMR by removing a small aliquot and reacting with a mixture of iodine in pentane. After quenching with saturated $Na_2S_2O_4$, the organic layer was separated and concentrated. ^{1}H NMR was used to quantify the aryl iodide (as a readout for the reactive organometallic reagent), remaining aryl bromide, and protodehalogenated side product.

1 hour and 40 minutes: 10% ArI, 58% ArBr, 32% ArH.
3 hours and 55 minutes: 28% ArI, 40% ArBr, 32% ArH.
6 hours and 50 minutes: 48% ArI, 31% ArBr, 21% ArH.

After seven hours, the reaction was cooled to -78°C in a dry ice / acetone bath. Phosphorus trichloride (50 µL, 0.0785 g, 0.572 mmol, 0.24 eq relative to aryl Grignard as readout by NMR yield of corresponding aryl iodide) was added (neat, dropwise over 2 minutes). The reaction changed from an orange cloudy appearance to a yellow cloudy appearance. The reaction was allowed to warm to room temperature and stir overnight. Nine hours later, the crude reaction was filtered through celite (20 mL), rinsing with DCM (100 mL). The filrate was concentrated to give a viscous orange oil. The crude product was purified by normal phase column chromatography (5 cm diameter, 300 mL silica gel, isocratic 8/1 Hex), giving **L18** as a white powder (40.4 mg, 0.106 mmol, 19% yield).

^{1}H NMR (500 MHz, C_6D_6) δ 6.80-6.76 (m, 6H), 6.27 (dd, *J* = 8.0, 3.8 Hz, 3H), 1.86 (s, 9H).

^{13}C NMR (126 MHz, $CDCl_3$) δ 144.23 (d, J_{C-P} = 27.5 Hz), 138.77 (d, J_{C-P} = 14.5 Hz), 133.71 (d, J_{C-P} = 4.7 Hz), 133.43, 130.11, 118.40, 113.81, 21.20 (d, J_{C-P} = 21.4 Hz).

^{31}P NMR (202 MHz, C_6D_6 referenced to H_3PO_4 in D_2O) δ -26.84.

HRMS (ESI$^+$) Calculated for $C_{24}H_{19}N_3P$ $(M+H)^+$: 380.1317, Found: 380.1301

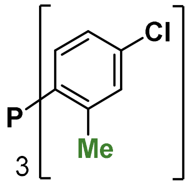 **SI-7**

Tris(4-chloro-2-methylphenyl)phosphine SI-7. A dry, stir bar-equipped 50 mL Schlenk flask was sealed with a rubber septum and vac-filled with nitrogen three times. In a separate dry 40 mL vial, 2-bromo-5-chlorotoluene (2.055 g, 10.0 mmol, 1.00 eq) was massed out. The vial was likewise put under nitrogen, and dry THF (16.7 mL total, 0.60 Molar) was used to transfer the aryl bromide to the Schlenk flask. The solution was cooled with stirring to -78°C in a dry ice / acetone bath. To this stirring solution was added n-butyllithium (1.6 Molar in hexanes, 5.6 mL, 9.0 mmol, 0.90 eq, dropwise over 10 minutes), causing a change in the reaction appearance from clear / colorless to opaque / cream-colored. After additional stirring at -78°C for 1 hour and 50 minutes, PCl_3 (0.218 mL, 0.343 g, 2.50 mmol, 2.50 eq) was added (neat, dropwise over 3-4 minutes). The now opaque orange suspension was allowed to gradually warm to room temperature with stirring.

Two days later, the reaction was quenched by addition of saturated NH_4Cl. The mixture was diluted with H_2O and extracted with DCM three times. Combined organics were washed with brine, dried with Na_2SO_4, and concentrated by rotary evaporation. The crude black oil was filtered through a pad of silica gel rinsing with 5/1 Hex/DCM, giving a light yellow oil. This material was purified by column chromatography (4 cm diameter, 120 mL SiO_2, isocratic 100% hexanes, R_f = 0.27) giving **SI-7** as a fluffy white solid (276.0 mg = 0.6781 mmol = 27% yield).

^{1}H NMR (500 MHz, $CDCl_3$) δ 7.24 (dd, *J* = 4.1, 2.0, 3H), 7.07 (dd, *J* = 8.2, 2.2 Hz, 3H), 6.59 (dd, *J* = 8.2, 3.8 Hz, 3H), 2.34 (s, 9H).

^{13}C NMR (126 MHz, $CDCl_3$) δ 144.58 (d, *J* = 27.8 Hz), 135.34, 134.27, 132.17 (d, *J* = 11.4 Hz), 130.42 (d, *J* = 4.9 Hz), 126.68, 21.13 (d, *J* = 21.5 Hz).

^{31}P NMR (202 MHz, C_6D_6 referenced to H_3PO_4 in D_2O) δ -31.64.

HRMS (EI^+) Calculated for $C_{21}H_{18}Cl_3P$ $(M)^+$: 406.02121, Found: 406.02040

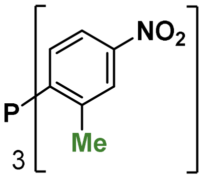

L19, σ_{para} = +0.778

Tris(2-methyl-4-nitrophenyl)phosphine L19. This synthesis was based on a reported conversion of aryl chlorides to nitroaromatics.[12] Sodium nitrite was dried under high vacuum in 80 °C sand bath, and *tert*-amyl alcohol was degassed by bubbling nitrogen through for 20 min. A dry, stir bar-equipped, 15 mL pressure tube was brought into an argon-filled glovebox and charged with sodium nitrite (0.24877 g, 3.606 mmol, 6.00 eq), tris(4-chloro-2-methylphenyl)phosphine (0.2450 g, 0.6009 mmol, 1.00 eq),

Pd_2dba_3 (41.1 mg, 0.0449 mmol, 7.5 mol%), and *t*BuBrettPhos (52.2 mg, 0.108 mmol, 18 mol%). *tert*-amyl alcohol (4.0 mL) and tris[2-(2-methoxyethoxy)ethyl]amine (28.8 µL, 29.1 mg, 0.0900 mmol, 0.15 eq) were added, and the pressure tube was tightly capped. The reaction was brought out of the glovebox and into a fume hood, where it was stirred for 72 hours at 140 °C.

After cooling to room temperature, the catalyst was removed by silica gel filtration (diluting with 100 mL EtOAc). The crude product was then purified by normal phase column chromatography (70 g silica gel, 3.5x15cm, dry loading on celite, 50:1 to 20:1 Hex/EtOAc), giving **L19** as a yellow powder (74.1 mg, 0.169 mmol, 28% yield).

^{1}H NMR (500 MHz, $CDCl_3$) δ 8.16 (dd, *J* = 4.4, 2.4 Hz, 3H), 7.97 (dd, *J* = 8.4, 2.3 Hz, 3H), 6.84 (dd, *J* = 8.4, 3.5 Hz, 3H), 2.50 (s, 9H).

^{13}C NMR (126 MHz, $CDCl_3$) δ 149.12, 144.88 (d, $J_{C\text{-}P}$ = 28.2 Hz), 140.68 (d, $J_{C\text{-}P}$ = 14.5 Hz), 133.82, 125.23 (d, $J_{C\text{-}P}$ = 4.9 Hz), 121.51, 21.55 (d, $J_{C\text{-}P}$ = 21.2 Hz).

^{31}P NMR (202 MHz, $CDCl_3$ referenced to H_3PO_4 in D_2O) δ -26.72.

HRMS (ESI$^+$) Calculated for $C_{21}H_{18}N_3O_6P$ (M)$^+$: 439.0933, Found: 439.0932

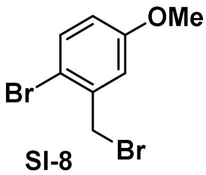

SI-8

1-bromo-2-(bromomethyl)-4-methoxybenzene SI-8. A dry, stir bar-equipped, 3-neck 250 mL round bottom flask was fitted with a reflux condenser and put under nitrogen. 1-bromo-4-methoxy-2-methylbenzene (1.39 mL, 2.011 g, 10.0 mmol, 1.00 eq), CCl_4 (20.0 mL, 0.50 Molar, not degassed), *N*-bromosuccinimide (recrystallized, 2.67 g, 15.0 mmol, 1.50 eq), and benzoyl peroxide (0.121 g, 0.50 mmol, 5 mol%) were added by briefly removing a septum while under positive nitrogen pressure. The reaction was heated to reflux for 18 hours.

The next day, the reaction was filtered through a silica gel plug, rinsing with 10/1 Hex/Et_2O. The crude product was purified by normal phase column chromatography (5 cm diameter, 300 mL silica gel, isocratic 4/1 Hex/DCM), giving **SI-8** as a fluffy, slightly off-white powder (1.404 g, 5.015 mmol, 50% yield).

^{1}H NMR (500 MHz, $CDCl_3$) δ 7.45 (d, *J* = 8.8 Hz, 1H), 6.99 (d, *J* = 3.0 Hz, 1H), 6.74 (dd, *J* = 8.8, 3.0 Hz, 1H), 4.56 (s, 2H), 3.80 (s, 3H).

^{13}C NMR (126 MHz, $CDCl_3$) δ 159.28, 137.90, 134.06, 116.67, 116.27, 114.85, 55.71, 33.61.

HRMS (EI$^+$) Calculated for $C_8H_8O^{79}Br_2$ (M)$^+$: 277.89422, Found: 277.89402

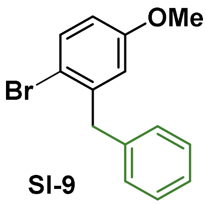

SI-9

2-benzyl-1-bromo-4-methoxybenzene SI-9. This procedure was based on a previous report of the selective coupling of benzylic bromides.[4] A stir bar-equipped, 3-neck 250 mL round bottom flask was sealed with two septa and equipped with a reflux condenser attached to a nitrogen inlet. After the system was put under nitrogen, reagents and solvents were added by briefly removing a septum while under a positive nitrogen pressure. In this manner, phenylboronic acid (0.5990 g, 4.913 mmol, 1.00 eq), ethanol (7.8 mL, 0.63 Molar), water (2.1 mL, 2.3 Molar), 1-bromo-2-(bromomethyl)-4-methoxybenzene **SI-3** (1.376 g, 4.913 mmol, 1.00 eq) as a solution in toluene (9.5 mL, 0.52 Molar), aqueous sodium carbonate (5.53 mL, 1.00 Molar, 5.53 mmol, 1.13 eq), and $Pd(PPh_3)_4$ (0.1135 g, 0.09826 mmol, 2 mol%) were added to the reaction.

The reaction was heated to 80°C for 12 hours and then filtered through celite, rinsing with Et_2O. After concentration, the crude material was diluted with H_2O (30 mL) and extracted with Et_2O (3x30 mL). Combined organics were washed with brine (100 mL), dried with Na_2SO_4, decanted, and reconcentrated. The crude product was purified by normal phase column chromatography (5 cm diameter, 300 mL silica gel, isocratic 4/1 Hex/DCM, R_f = 0.27), giving **SI-9** as a clear colorless oil (0.7136 g, 2.575 mmol, 52% yield).

^{1}H NMR (500 MHz, $CDCl_3$) δ 7.45 (d, *J* = 8.6 Hz, 1H), 7.30 (t, *J*= 7.4 Hz, 2H), 7.24-7.18 (m, 3H), 6.68 (d, *J* = 3.0 Hz, 1H), 6.66 (dd, *J* = 8.6, 3.1 Hz, 1H), 4.07 (s, 2H), 3.73 (s, 3H).

^{13}C NMR (126 MHz, $CDCl_3$) δ 159.06, 141.46, 139.43, 133.42, 129.09, 128.61, 126.42, 117.07, 115.44, 113.44, 55.49, 42.01.

HRMS (EI$^+$) Calculated for $C_{14}H_{13}O^{79}Br$ $(M)^+$: 276.01498, Found: 276.01453

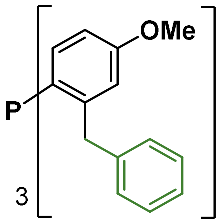

L20, σ_{para} = -0.268

Tris(2-benzyl-4-methoxyphenyl)phosphine L20. A flame dried, 25 mL recovery flask was equipped with a stir bar, sealed with a septum, and put under nitrogen. To a separate, flame-dried 40 mL vial was added 2-benzyl-1-bromo-4-methoxybenzene (0.6762 g, 2.44 mmol, 1.00 eq). The vial put under nitrogen, and THF (4.1 mL total) was used to transfer the aryl bromide to the reaction flask, with rinsing for quantitative transfer. The solution was cooled to -78°C by submerging in a dry ice / acetone bath, and then *n*butyllithium (1.6 Molar in hexanes, 1.37 mL, 2.20 mmol, 0.90 eq) was added dropwise over 5-

10 minutes. The reaction was allowed to stir for 1.5 hours at -78°C. Phosphorus trichloride (53.2 µL, 83.8 mg, 0.610 mmol, 0.25 eq) was added neat in a dropwise manner over 2-3 minutes. The reaction was allowed to gradually warm to room temperature and stirred overnight.

The next day, the reaction was quenched with NH_4Cl (10 mL). Water (20 mL) and DCM (30 mL) were added, and the aqueous layer was extracted (3 x 30 mL DCM). The combined organics were washed with brine (100 mL), dried (Na_2SO_4), decanted, and concentrated. The crude product was purified by normal phase column chromatography (3 cm diameter, 120 mL SiO_2, isocratic 1/1 Hex/DCM), giving **L20** as a white powder (0.2932 g, 0.471 mmol, 77% yield).

^1H NMR (500 MHz, $CDCl_3$) δ 7.14 (t, J = 7.2 Hz, 6H), 7.08 (t, J = 7.2 Hz, 3H), 7.02 (d, J = 7.0 Hz, 6H), 6.74 (dd, J = 8.8, 3.6 Hz, 3H), 6.65-6.61 (m, 6H), 4.05 (s, 6H), 3.72 (s, 9H).

^{13}C NMR (126 MHz, $CDCl_3$) δ 160.26, 147.19 (d, J_{C-P} = 27.8 Hz), 140.58, 135.56, 129.39, 128.26, 126.62 (d, J_{C-P} = 9.3 Hz), 125.91, 116.13 (d, J_{C-P} = 5.3 Hz), 111.82, 55.16, 40.21 (d, J_{C-P} = 22.2 Hz).

^{31}P NMR (202 MHz, $CDCl_3$, referenced to H_3PO_4 in D_2O) δ -35.94.

HRMS (EI^+) Calculated for $C_{42}H_{39}O_3P$ $(M)^+$: 622.2637, Found: 622.2643

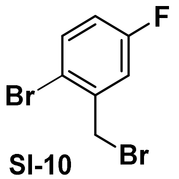

SI-10

1-bromo-2-(bromomethyl)-4-fluorobenzene SI-10. A dry, stir bar-equipped, 3-neck 300 mL round bottom flask was fitted with a reflux condenser and put under nitrogen. 1-bromo-4-fluoro-2-methylbenzene (1.26 mL, 1.890 g, 10.0 mmol, 1.00 eq), CCl_4 (20.0 mL, 0.50 Molar, not degassed), N-bromosuccinimide (recrystallized, 2.67 g, 15.0 mmol, 1.50 eq), and benzoyl peroxide (0.121 g, 0.50 mmol, 5 mol%) were added by briefly removing a septum while under positive nitrogen pressure. The reaction was heated to reflux for 18 hours.

The next day, the reaction was filtered through a silica gel plug, rinsing with hexanes. The crude product was purified by normal phase column chromatography (5 cm diameter, 300 mL silica gel, isocratic 100% hexanes), giving **SI-10** (1.219 g, 4.55 mmol, 45% yield).

^1H NMR (500 MHz, $CDCl_3$) δ 7.53 (dd, J = 8.8, 5.2 Hz, 1H), 7.20 (dd, J = 8.8, 3.0 Hz, 1H), 6.92 (ddd, J = 8.8, 7.8, 3.0 Hz, 1H), 4.54 (s, 2H).

^{13}C NMR (126 MHz, $CDCl_3$) δ 161.94 (d, J_{C-F} = 248.1 Hz), 138.96 (d, J_{C-F} = 7.6 Hz), 134.67 (d, J_{C-F} = 7.8 Hz), 118.62 (d, J_{C-F} = 3.4 Hz), 118.27 (d, J_{C-F} = 23.6 Hz), 117.48 (d, J_{C-F} = 22.3 Hz), 32.59 (d, J_{C-F} = 1.6 Hz).

^{19}F NMR (470 MHz, $CDCl_3$, referenced to $CFCl_3$ in $CDCl_3$) δ -114.28

HRMS (EI^+) Calculated for $C_7H_5{}^{79}Br_2F$ $(M)^+$: 265.87423, Found: 265.87446

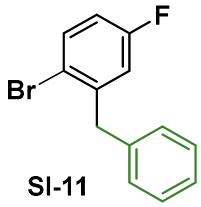

SI-11

2-benzyl-1-bromo-4-fluorobenzene SI-11. This procedure was based on a previous report of the selective coupling of benzylic bromides.[4] A stir bar-equipped, 3-neck 300 mL round bottom flask was sealed with two septa and equipped with a reflux condenser attached to a nitrogen inlet. After the system was put under nitrogen, reagents and solvents were added by briefly removing a septum while under a positive nitrogen pressure. In this manner, phenylboronic acid (0.5548 g, 4.550 mmol, 1.00 eq), ethanol (7.1 mL, 0.63 Molar), water (2.0 mL, 2.3 Molar), 1-bromo-2-(bromomethyl)-4-fluorobenzene **SI-5** (1.219 g, 4.550 mmol, 1.00 eq) as a solution in toluene (8.75 mL, 0.52 Molar), aqueous sodium carbonate (5.12 mL, 1.00 Molar, 5.12 mmol, 1.13 eq), and $Pd(PPh_3)_4$ (0.1052 g, 0.0910 mmol, 2 mol%) were added to the reaction.

The reaction was heated to 80°C for 12 hours and then filtered through celite, rinsing with Et_2O. After concentration, the crude material was diluted with H_2O (30 mL) and extracted with Et_2O (3x30 mL). Combined organics were washed with brine (100 mL), dried with Na_2SO_4, decanted, and reconcentrated. The crude product was purified by normal phase column chromatography (5 cm diameter, 300 mL silica gel, isocratic 100% hexanes) followed by reverse phase column chromatography (3 cm diameter, 50 mL C18 silica gel, isocratic 3/1 MeCN/H_2O, R_f = 0.25, extracting with 4x100 mL pentane and drying with Na_2SO_4), giving **SI-11** as a clear colorless oil (0.5966 g, 2.250 mmol, 49% yield).

^{1}H NMR (500 MHz, $CDCl_3$) δ 7.52 (m, 1H), 7.32 (m, 2H), 7.25 (m, 1H), 7.19 (d, J = 7.4 Hz, 2H), 6.85-6.79 (m, 2H), 4.08 (s, 2H).

^{13}C NMR (126 MHz, $CDCl_3$) δ 162.11 (d, J_{C-F} = 246.5 Hz), 142.75 (d, J_{C-F} = 7.1 Hz), 138.73, 133.97 (d, J_{C-F} = 7.9 Hz), 129.18, 128.78, 126.72, 118.93 (d, J_{C-F} = 3.3 Hz), 118.01 (d, J_{C-F} = 23.0 Hz), 115.16 (d, J_{C-F} = 22.5 Hz), 41.94 (d, J_{C-F} = 1.4 Hz).

^{19}F NMR (470 MHz, $CDCl_3$, referenced to $CFCl_3$ in $CDCl_3$) δ -115.30

HRMS (EI$^+$) Calculated for $C_{13}H_{10}{}^{79}BrF$ $(M)^+$: 263.99498, Found: 263.99517

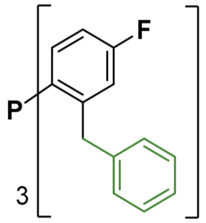

L21, σ_{para} = +0.062

Tris(2-benzyl-4-fluorophenyl)phosphine L21. A flame dried, 25 mL recovery flask was equipped with a stir bar, sealed with a septum, and put under nitrogen. To a separate, flame-dried 40 mL vial was added

2-benzyl-1-bromo-4-fluorobenzene (0.5583 g, 2.106 mmol, 1.00 eq). The vial put under nitrogen, and THF (3.5 mL total) was used to transfer the aryl bromide to the reaction flask, with rinsing for quantitative transfer. The solution was cooled by submerging in a dry ice / acetone bath. To the -78°C mixture was added *n*butyllithium (1.6 Molar in hexanes, 1.18 mL, 1.895 mmol, 0.90 eq) dropwise over 5-10 minutes. The reaction was allowed to stir for 1.5 hours at -78°C. Phosphorus trichloride (45.9 µL, 72.3 mg, 0.527 mmol, 0.25 eq) was added neat in a dropwise manner over 2-3 minutes. The reaction was allowed to gradually warm to room temperature and stirred overnight.

The next day, the reaction was quenched with NH_4Cl (10 mL). Water (20 mL) and DCM (30 mL) were added, and the aqueous layer was extracted (3x30 mL DCM). The combined organics were washed with brine (100 mL), dried (Na_2SO_4), decanted, and concentrated. The crude product was purified by normal phase column chromatography (3 cm diameter, 120 mL SiO_2, isocratic 5/1 Hex/DCM, Rf = 0.20), giving **L21** as a white powder (0.1394 g, 0.2376 mmol, 45% yield).

^{1}H NMR (500 MHz, $CDCl_3$) δ 7.18-7.14 (m, 6H), 7.13-7.09 (m, 3H), 6.99 (d, *J* = 7.0 Hz, 6H), 6.84-6.76 (m, 6H), 6.71 (ddd, *J* = 8.4, 6.2, 3.5 Hz, 3H), 4.06 (s, 6H)

^{13}C NMR (126 MHz, $CDCl_3$) δ 163.75 (d, *J* = 249.6 Hz), 148.35 (dd, *J* = 28.5, 7.3 Hz), 139.53 (d, *J* = 1.5 Hz), 135.93 (d, *J* = 7.9 Hz), 129.85 (dd, *J* = 11.1, 3.3 Hz), 129.33, 128.48, 126.36, 117.35 (dd, *J* = 21.3, 5.4 Hz), 113.88 (d, *J* = 20.4 Hz), 40.23 (dd, *J* = 22.6, 1.3 Hz).

^{31}P NMR (202 MHz, $CDCl_3$, referenced to H_3PO_4 in D_2O) δ -39.71 (q, *J* = 2.3 Hz)

^{19}F NMR (470 MHz, $CDCl_3$, referenced to $CFCl_3$ in $CDCl_3$) δ -112.80 (q, *J* = 10.2 Hz)

HRMS (EI^+) Calculated for $C_{39}H_{30}F_3P$ $(M)^+$: 586.2037, Found: 586.2039

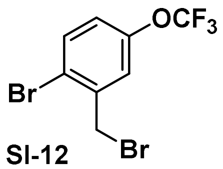

SI-12

1-bromo-2-(bromomethyl)-4-(trifluoromethoxy)benzene SI-12. A dry, stir bar-equipped, 3-neck 250 mL round bottom flask was fitted with a reflux condenser and put under nitrogen. 1-bromo-2-methyl-4-(trifluoromethoxy)benzene (2.550 g, 10.0 mmol, 1.00 eq), CCl_4 (20.0 mL, 0.50 Molar, not degassed), N-bromosuccinimide (recrystallized, 1.958 g, 11.0 mmol, 1.10 eq), and benzoyl peroxide (48.4 mg, 0.20 mmol, 2 mol%) were added by briefly removing a septum while under positive nitrogen pressure. After refluxing for 2.5 hours, monitoring of the reaction by NMR showed no conversion to product. Additional benzoyl peroxide (100 mg, 0.41 mmol, 4.1 mol%) was added, and the reaction was allowed to reflux for 24 hours.

The reaction was filtered through a silica gel plug (rinsing with 5:1 Hex/Et_2O), and concentrated *in vacuo*. The crude product was purified by normal phase column chromatography (5 cm diameter, 350 mL silica gel, isocratic 100% hexanes), giving **SI-12** (1.548 g, 4.64 mmol, 46% yield).

^{1}H NMR (500 MHz, $CDCl_3$) δ 7.60 (d, *J* = 8.8 Hz, 1H), 7.33 (d, *J* = 2.7 Hz, 1H), 7.06 (ddq, *J* = 8.8, 3.0, 1.0 Hz, 1H), 4.56 (s, 2H).

^{13}C NMR (126 MHz, $CDCl_3$) δ 148.65 (q, J = 1.9 Hz), 139.08, 134.69, 123.75, 122.61, 122.09, 120.39 (q, J = 258.7 Hz), 32.29.

^{19}F NMR (470 MHz, $CDCl_3$, referenced to $CFCl_3$ in $CDCl_3$) δ -58.51.

HRMS (EI^+) Calculated for $C_8H_5OF_3Br_2$ $(M)^+$: 331.86596, Found: 331.86479

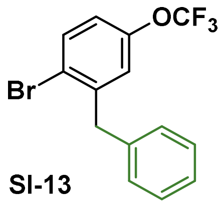

SI-13

2-benzyl-1-bromo-4-(trifluoromethoxy)benzene SI-13. This procedure was based on a previous report of the selective coupling of benzylic bromides.[4] A stir bar-equipped, 3-neck 250 mL round bottom flask was sealed with two septa and equipped with a reflux condenser attached to a nitrogen inlet. After the system was put under nitrogen, reagents and solvents were added by briefly removing a septum while under a positive nitrogen pressure. In this manner, phenylboronic acid (0.4016 g, 3.294 mmol, 1.00 eq), ethanol (5.2 mL, 0.63 Molar), water (1.43 mL, 2.3 Molar), 1-bromo-2-(bromomethyl)-4-(trifluoromethoxy)benzene **SI-7** (1.10 g, 3.294 mmol, 1.00 eq) as a solution in toluene (6.3 mL, 0.52 Molar), aqueous sodium carbonate (3.7 mL, 1.00 Molar, 3.7 mmol, 1.13 eq), and $Pd(PPh_3)_4$ (0.1142 g, 0.0988 mmol, 3 mol%) were added to the reaction.

The reaction was heated to 80°C for 15 hours and then filtered through celite, rinsing with Et_2O. After concentration, the crude material was diluted with H_2O and extracted with Et_2O. Combined organics were washed with brine, dried with Na_2SO_4, decanted, and reconcentrated. The crude product was purified by normal phase column chromatography (isocratic 100% hexanes), giving **SI-13** as a clear colorless oil (0.3895 g, 1.176 mmol, 36% yield).

^1H NMR (500 MHz, $CDCl_3$) δ 7.52 (m, 1H), 7.32 (m, 2H), 7.25 (m, 1H), 7.19 (d, J = 7.4 Hz, 2H), 6.85-6.79 (m, 2H), 4.08 (s, 2H).

^{13}C NMR (126 MHz, $CDCl_3$) δ 162.11 (d, $J_{C\text{-}F}$ = 246.5 Hz), 142.75 (d, $J_{C\text{-}F}$ = 7.1 Hz), 138.73, 133.97 (d, $J_{C\text{-}F}$ = 7.9 Hz), 129.18, 128.78, 126.72, 118.93 (d, $J_{C\text{-}F}$ = 3.3 Hz), 118.01 (d, $J_{C\text{-}F}$ = 23.0 Hz), 115.16 (d, $J_{C\text{-}F}$ = 22.5 Hz), 41.94 (d, $J_{C\text{-}F}$ = 1.4 Hz).

^{19}F NMR (470 MHz, $CDCl_3$, referenced to $CFCl_3$ in $CDCl_3$) δ -115.30

R_f = 0.25 on reverse phase TLC in 3/1 MeCN/H_2O

HRMS (EI^+)
Calculated for $C_{14}H_{10}OF_3Br$ $(M)^+$: 329.98671
Found: 329.98647

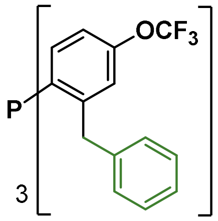

L22, σ_{para} = +0.35

Tris(2-benzyl-4-(trifluoromethoxy)phenyl)phosphine L22. A flame dried, 25 mL recovery flask was equipped with a stir bar, sealed with a septum, and put under nitrogen. To a separate, flame-dried 40 mL vial was added 2-benzyl-1-bromo-4-(trifluoromethoxy)benzene (0.4368 g, 1.319 mmol, 1.00 eq). The vial put under nitrogen, and THF (2.2 mL total) was used to transfer the aryl bromide to the reaction flask, with rinsing for quantitative transfer. The solution was cooled by submerging in a dry ice / acetone bath. To the -78°C mixture was added *n*butyllithium (1.6 Molar in hexanes, 0.74 mL, 1.19 mmol, 0.90 eq) dropwise over 5-10 minutes. The reaction was allowed to stir for 1.5 hours at -78°C. Phosphorus trichloride (28.8 µL, 45.3 mg, 0.330 mmol, 0.25 eq) was added neat in a dropwise manner over 2-3 minutes. The reaction was allowed to gradually warm to room temperature and stirred overnight.

The next day, the reaction was quenched with NH_4Cl (5 mL). Water (10 mL) and DCM (15 mL) were added, and the aqueous layer was extracted (3x15 mL DCM). The combined organics were washed with brine (50 mL), dried (Na_2SO_4), decanted, and concentrated. The crude product was purified by normal phase column chromatography (isocratic 100% hexanes), giving **L22** as a white powder (0.1394 g, 0.2376 mmol, 45% yield).

^{1}H NMR (500 MHz, $CDCl_3$) δ 7.13-7.04 (m, 9H), 6.97-6.88 (m, 12H), 6.68 (dd, *J* = 8.4, 3.6 Hz, 3H), 4.06 (s, 6H).

^{13}C NMR (126 MHz, $CDCl_3$) δ 150.29 (d, *J* = 1.6 Hz), 147.87 (d, *J* = 28.3 Hz), 139.05 (d, *J* = 1.1 Hz), 135.82, 132.55 (d, *J* = 12.1 Hz), 129.19, 128.45, 126.44, 122.59 (d, *J* = 5.4 Hz), 120.53 (q, *J* = 258 Hz), 118.65, 40.48 (d, *J* = 21.8 Hz).

^{31}P NMR (202 MHz, $CDCl_3$, referenced to H_3PO_4 in D_2O) δ -35.83

^{19}F NMR (470 MHz, $CDCl_3$, referenced to $CFCl_3$ in $CDCl_3$) δ -58.04

HRMS (ESI+) Calculated for $C_{42}H_{31}O_3F_9P$ $(M+H)^+$: 785.1867, Found: 785.1862

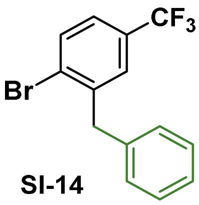

SI-14

2-benzyl-1-bromo-4-(trifluoromethyl)benzene SI-14. In an argon-filled glovebox, combined (2-bromo-5-(trifluoromethyl)phenyl)methanol (25.0 g, 98.0 mmol, 1.00 eq), $FeCl_3$ (3.24 g, 20.0 mmol, 0.20 eq), and dry benzene (100 mL) in a stir bar-equipped 200 mL high pressure round bottom flask. The reaction was sealed, brought into a fume hood, and stirred at 100°C for two hours. Monitoring the reaction by TLC (10% EtOAc in hexanes) showed low conversion. Additional $FeCl_3$ (4.00 g, 24.7 mmol, 0.25 eq) was added, and the reaction was stirred at 100°C for 15 more hours.

At this point, TLC indicated that the reaction had gone to full conversion. The mixture was then filtered through celite (rinsing with hexanes) and concentrated *in vacuo*. The crude product was purified by normal phase column chromatography (isocratic 100% hexanes), giving **SI-14** as a white solid (24.32 g, 77.2 mmol, 79% yield).

^{1}H NMR (500 MHz, $CDCl_3$) δ 7.70 (d, J = 8.3 Hz, 1H), 7.39 (s, 1H), 7.36-7.29 (m, 3H), 7.25 (m, 1H), 7.18 (d, J = 7.3 Hz, 2H), 4.16 (s, 2H).

^{13}C NMR (126 MHz, $CDCl_3$) δ 141.62, 138.44, 133.59, 130.14 (q, $J_{C\text{-}F}$ = 32.7 Hz), 129.05, 128.90 (q, $J_{C\text{-}F}$ = 5.9 Hz), 128.84, 127.78 (q, $J_{C\text{-}F}$ = 3.8 Hz), 126.82, 124.75 (q, $J_{C\text{-}F}$ = 3.8 Hz). 123.93 (q, $J_{C\text{-}F}$ = 272.6 Hz), 41.87.

^{19}F NMR (470 MHz, $CDCl_3$, referenced to $CFCl_3$ in $CDCl_3$) δ -65.23.

HRMS (EI$^+$) Calculated for $C_{14}H_{10}BrF_3$ $(M)^+$: 313.99179, Found: 313.99135

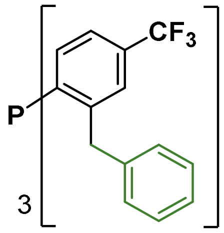

L23, σ_{para} = +0.54

Tris(2-benzyl-4-(trifluoromethyl)phenyl)phosphine L23. To a suspension of magnesium powder (0.170 g, 7.00 mmol, 1.02 eq) in THF (14 mL) in a dry 40 mL vial was added 2-benzyl-1-bromo-4-(trifluoromethyl)benzene. After addition of catalytic iodine and brief heating, the Grignard reaction initiated. The reaction was allowed to stir overnight at room temperature. The next day, a small aliquot was quenched with H_2O and analyzed by TLC (100% hexanes), showing complete consumption of the aryl bromide. The reaction was submerged in a dry ice / acetone bath, and upon cooling to -78°C, phosphorus trichloride (0.198 mL, 0.3109 g, 2.20 mmol, 0.32 eq) was added dropwise. The reaction was stirred for one hour at -78°C and then at room temperature overnight.

The next day, an orange solid was visible in the reaction. TLC of a quenched aliquot (5% EtOAc in hexanes) showed complete consumption of the dehalogenated intermediate. Saturated NH_4Cl and H_2O were added, and the crude mixture was extracted with EtOAc twice. The combined organic layers were dried with Na_2SO_4 and concentrated to afford a red oil. This material was filtered through a plug of silica gel, rinsing with 5% EtOAc in hexanes to remove the red baseline side products. After concentration to an oil and application of high vac to remove solvent residue, the crude product was recrystallized from hot methanol (7 mL). After cooling for 30 minutes in an ice bath, the mixture was filtered through a medium porosity glass frit to give **L23** as a white solid (0.530 grams, 0.720 mmol, 33% yield).

^{1}H NMR (500 MHz, $CDCl_3$) δ 7.39 (dd, *J* = 4.5, 1.9 Hz, 3H), 7.26 (dd, *J* = 7.8, 1.1 Hz, 3H), 7.11-7.03 (m, 9H), 6.93-6.88 (m, 6H), 6.73 (dd, *J* = 8.0, 3.7 Hz, 3H), 4.09 (s, 6H).

^{13}C NMR (126 MHz, $CDCl_3$) δ 146.40 (d, *J* = 27.5 Hz), 138.78 (d, *J* = 0.8 Hz), 138.25 (d, *J* = 13.6 Hz), 134.80, 131.65 (d, *J* = 32.4 Hz), 129.11, 128.50, 127.05 (dq, J = 3.7, 1.1 Hz), 126.56, 124.01 (q, *J* = 272.9 Hz), 123.50 (q, *J* = 3.7 Hz), 40.61 (d, *J* = 21.1 Hz).

^{31}P NMR (202 MHz, $CDCl_3$, referenced to H_3PO_4 in D_2O) δ -31.72.

^{19}F NMR (470 MHz, $CDCl_3$, referenced to $CFCl_3$ in $CDCl_3$) δ -63.23.

HRMS (ESI+) Calculated for $C_{42}H_{31}F_9P$ $(M+H)^+$: 737.2020, Found: 737.2012

Ligand Testing Data

Determination of Retention Times

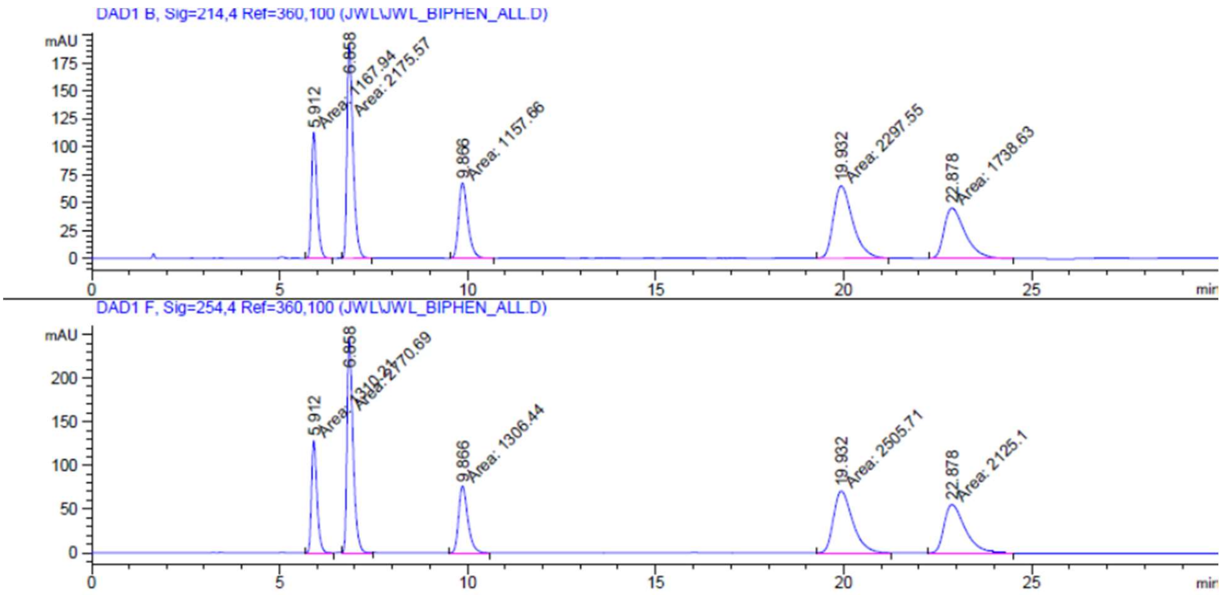

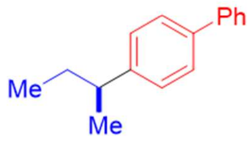

(+)-3a, Branched Product, Major enantiomer: 5.9 minutes

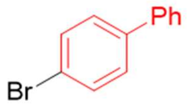

2a, Starting Aryl Bromide: 6.9 minutes

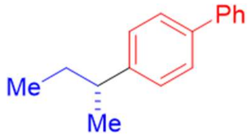

(-)-3a, Branched Product, Minor enantiomer: 9.9 minutes

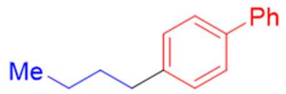

4a, Linear Product: 19.9 minutes

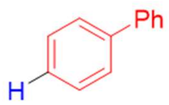

Protodehalogenated Side Product: 22.9 minutes

Conditions: OD-H chiral column, 2.0 mL/min, isocratic 100% hexanes

Compound characterization: **(+)-3a**: Characterization: **(-)-3a**: identical to **(+)-3a**; **4a** matched previous characterization.[13]

Determination of Response Factors

All standard solutions were prepared by dissolving the compounds in HPLC hexanes in 25 mL volumetric flasks.

Compound	Amount (µmol)	Injection Volume (µL)	Absorbance Area (mAU at 214 nm)	µmol / mAU (Normalizing to 5 µL injection)
**(±)-3a**	83.2	10	14338.73	0.01160
**(±)-3a**	79.9	5	6909.31	0.01156
**(±)-3a**	79.9	2.5	3463.31	0.01153
Average				0.01156
**2a**	78.9	5	7193.07	0.01097
**2a**	78.9	2.5	3559.17	0.01108
Average				0.01103
Biphenyl	90.1	5	5986	0.01505

General Procedure for Testing Ligands:

Cross-coupling reactions were assembled per general procedure D, using boronic acid **(*S*)-1a** in ≥99:1 e.r prepared by general procedure C. After 24 hours, the reactions were cooled to room temperature and filtered through a plug of silica gel in a glass pipet, rinsing with HPLC grade hexanes. The filtrate was collected in 25 mL volumetric flasks and further diluted with HPLC grade hexanes to the 25 mL mark. After thorough mixing, an aliquot of this solution was transferred to an HPLC vial and immediately subjected to HPLC analysis using the same conditions as above (OD-H chiral column, 2.0 mL/min, isocratic 100% hexanes, 214.4 nm absorbance). On each new day of HPLC analysis, a standard solution of the branched product standard was analyzed in duplicate to confirm the bulb brightness and to adjust response factors if necessary. If peak retention times drifted, standards were repeated as necessary to confirm the identity of the peaks.

***Ortho*-substituted Ligands:**

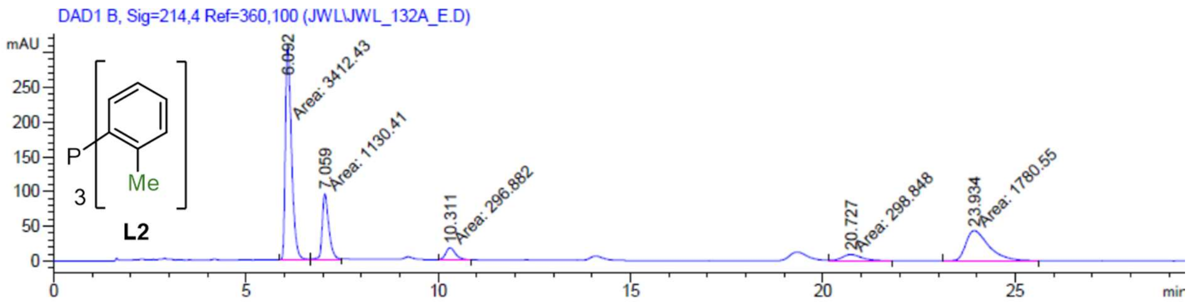

Response Factors Used
Branched Product = 0.01156 µmol / mAU, Linear Product = 0.01156 µmol / mAU, Aryl Bromide = 0.01103 µmol / mAU, Protodehalogenated Product = 0.01505 µmol / mAU

Quantity of 4-bromobiphenyl used = 24.1 mg = 0.1034 mmol

Branched Product = 42%
Conversion = 88%
Protodehalogenated = 26%
Branched / Linear Ratio = 12.4/1
e.r. = 92.00 : 8.00; Enantiospecificity = 85.7 %ES

A duplicate run gave:
Branched Product = 43%
Conversion = 87%
Protodehalogenated = 27%
Branched / Linear Ratio = 13.0/1
e.r. = 92.08 : 7.92
Enantiospecificity = 85.9 %ES

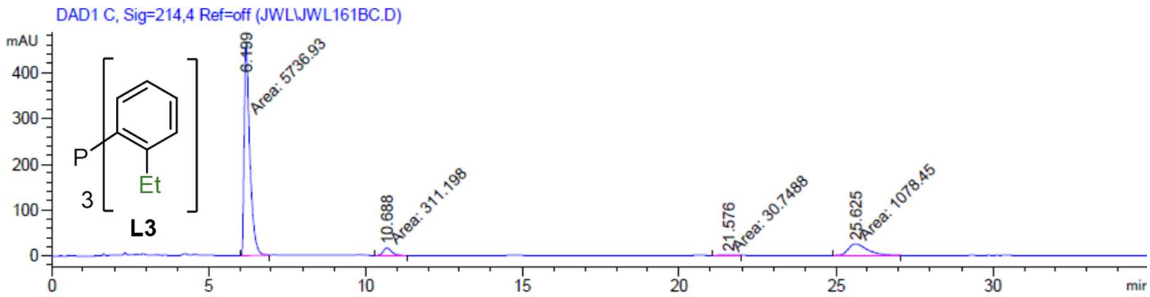

Response Factors Used
Branched Product = 0.1134 µmol / mAU, Linear Product = 0.1134 µmol / mAU, Aryl Bromide = 0.01081 µmol / mAU, Protodehalogenated Product = 0.01464 µmol / mAU

Quantity of 4-bromobiphenyl used = 23.1 mg = 0.0991 mmol
Branched Product = 69% yield
Conversion = 100%
Protodehalogenated = 16%
Branched/Linear Ratio = 197/1
e.r. = 94.85 : 5.15 ; Enantiospecificity = 91.5%

A duplicate run gave:
Branched Product = 67%
Conversion = 100%
Protodehalogenated = 18%
Branched / Linear Ratio = 126/1
Enantiospecificity = 91.2 %ES

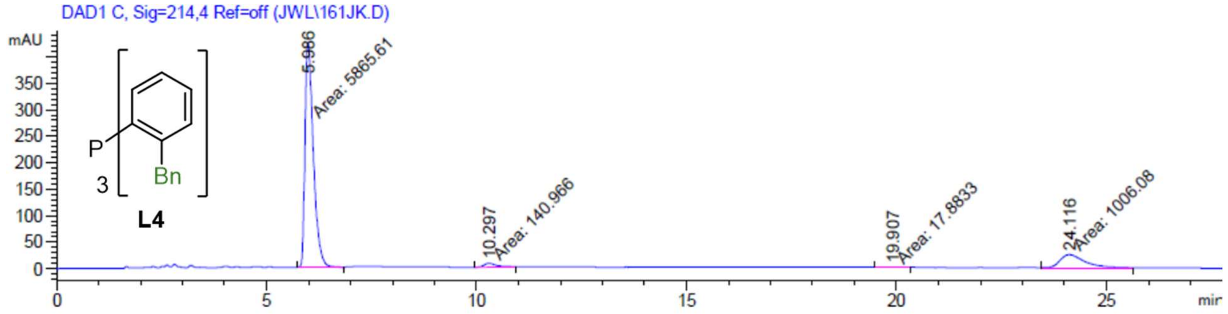

Response Factors Used
Branched Product = 0.01176 µmol / mAU, Linear Product = 0.01176 µmol / mAU, Aryl Bromide = 0.01122 µmol / mAU, Protodehalogenated Product = 0.01518 µmol / mAU

Quantity of 4-bromobiphenyl used = 23.6 mg = 0.1012 mmol
Branched Product = 70% yield
Conversion = 100%
Protodehalogenated = 15%
Branched/Linear Ratio = 336/1
e.r. = 97.65 : 2.35 ; Enantiospecificity = 97.3%

A duplicate run gave:
Branched Product = 73%
Conversion = 100%
Protodehalogenated = 14%
Branched / Linear Ratio = 371/1
Enantiospecificity = 98.0 %ES

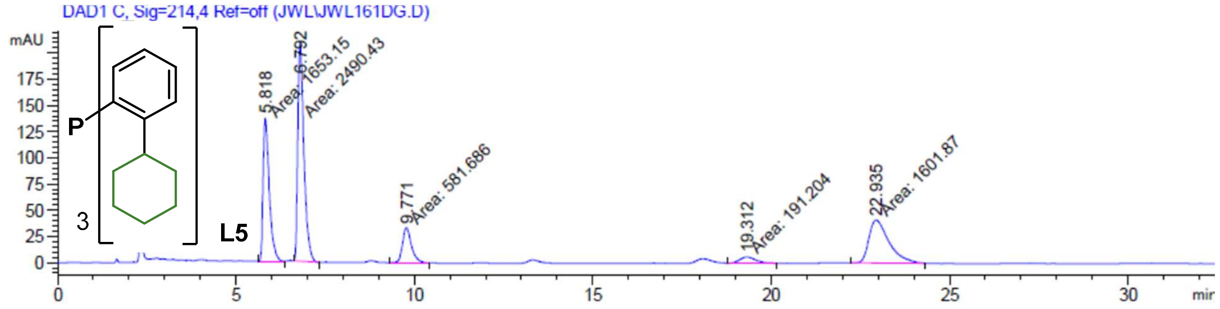

Response Factors Used
Branched Product = 0.01136 µmol / mAU, Linear Product = 0.01136 µmol / mAU, Aryl Bromide = 0.01093 µmol / mAU, Protodehalogenated Product = 0.01467 µmol / mAU

Quantity of 4-bromobiphenyl used = 23.7 mg = 0.1017 mmol
Branched Product = 25% yield
Conversion = 73%
Protodehalogenated = 23%
Branched/Linear Ratio = 11.7/1
e.r. = 73.97 : 26.03 ; Enantiospecificity = 48.9%

A duplicate run gave:
Branched Product = 19%
Conversion = 87%
Protodehalogenated = 31%
Branched / Linear Ratio = 14.6/1
Enantiospecificity = 49.1 %ES

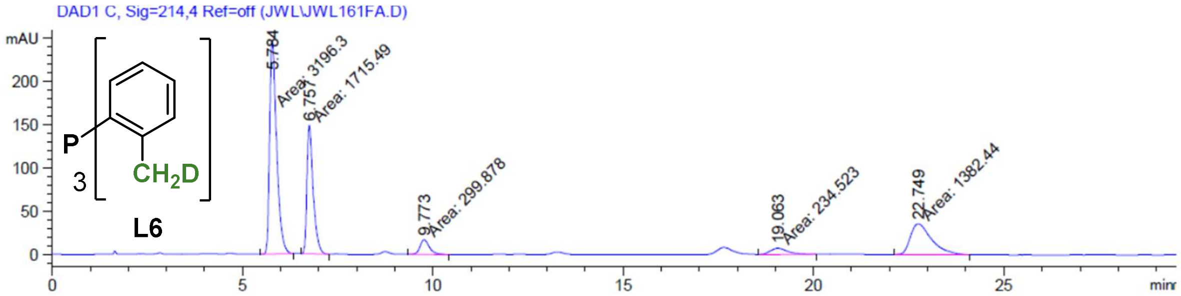

Response Factors Used
Branched Product = 0.01155 µmol / mAU, Linear Product = 0.01155 µmol / mAU, Aryl Bromide = 0.01101 µmol / mAU, Protodehalogenated Product = 0.01491 µmol / mAU

Quantity of 4-bromobiphenyl used = 23.5 mg = 0.1008 mmol
Branched Product = 40% yield
Conversion = 81%
Protodehalogenated = 20%
Branched/Linear Ratio = 14.9/1
e.r. = 91.42 : 8.58 ; Enantiospecificity = 84.5%

A duplicate run gave:
Branched Product = 35%
Conversion = 80%
Protodehalogenated = 21%
Branched / Linear Ratio = 15.2/1
Enantiospecificity = 84.9 %ES

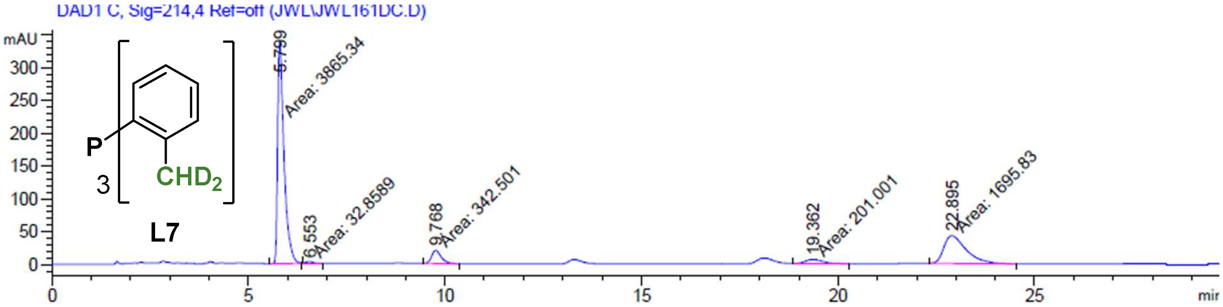

Response Factors Used
Branched Product = 0.01136 µmol / mAU, Linear Product = 0.01136 µmol / mAU, Aryl Bromide = 0.01093 µmol / mAU, Protodehalogenated Product = 0.01467 µmol / mAU

Quantity of 4-bromobiphenyl used = 23.6 mg = 0.1012 mmol
Branched Product = 47% yield
Conversion = 100%
Protodehalogenated = 25%
Branched/Linear Ratio = 20.9/1
e.r. = 91.86 : 8.14 ; Enantiospecificity = 85.4%

A duplicate run gave:
Branched Product = 44%
Conversion = 100%
Protodehalogenated = 22%
Branched / Linear Ratio = 24.8/1
Enantiospecificity = 85.3 %ES

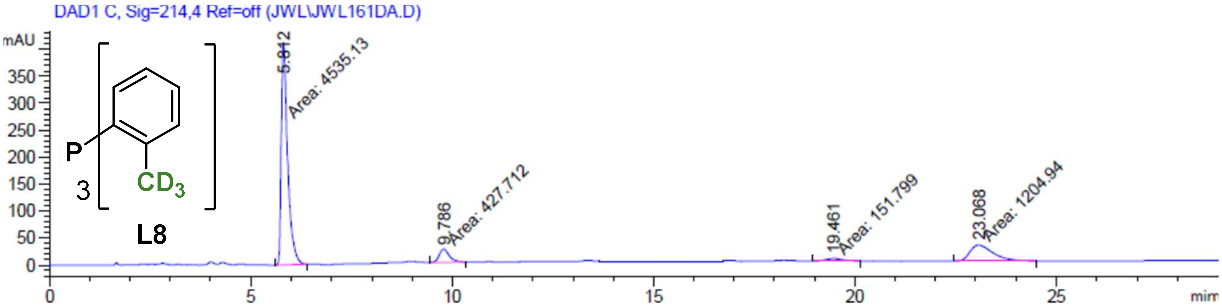

Response Factors Used
Branched Product = 0.01136 µmol / mAU, Linear Product = 0.01136 µmol / mAU, Aryl Bromide = 0.01093 µmol / mAU, Protodehalogenated Product = 0.01467 µmol / mAU

Quantity of 4-bromobiphenyl used = 22.9 mg = 0.09824 mmol
Branched Product = 57% yield
Conversion = 100%
Protodehalogenated = 18%
Branched/Linear Ratio = 32.7/1
e.r. = 91.38 : 8.62 ; Enantiospecificity = 84.4%

A duplicate run gave:
Branched Product = 55%
Conversion = 100%
Protodehalogenated = 16%
Branched / Linear Ratio = 41.1/1
Enantiospecificity = 83.9 %ES

Testing Ligand Electronics

P(*t*Bu)$_3$; Anhydrous Conditions

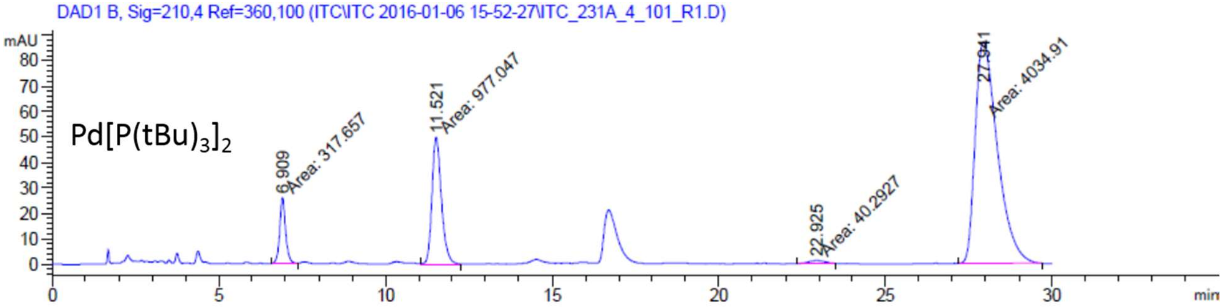

Response factor at 210.4 nm
Branched Product = Linear Product = 0.00542 μmol / mAU
Yield = 7.0%
B/L ratio = 32/1
e.r. = 75.46 : 24.54; % Enantiospecificity = 52.0%

P(2-Me-4-X-Ph)$_3$ Derivatives; Anhydrous Conditions

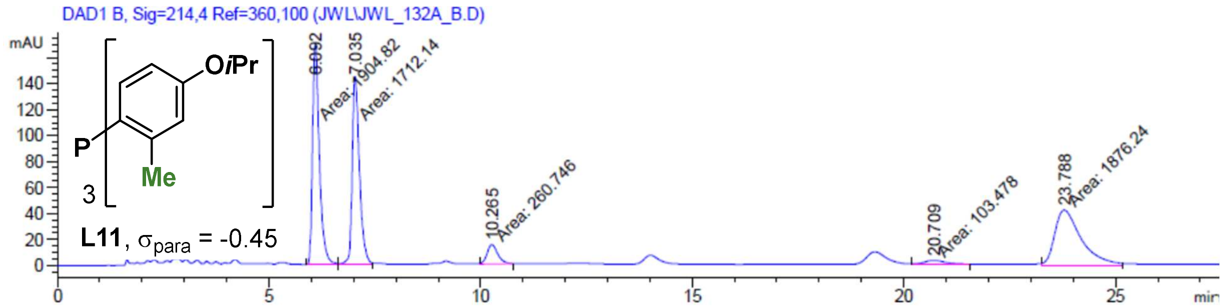

Response Factors Used
Branched Product = 0.01156 μmol / mAU, Linear Product = 0.01156 μmol / mAU, Aryl Bromide = 0.01103 μmol / mAU, Protodehalogenated Product = 0.01505 μmol / mAU

Quantity of 4-bromobiphenyl used = 23.2 mg = 0.0996 mmol
Branched Product = 25% yield
Conversion = 81%
Protodehalogenated = 28%
Branched/Linear Ratio = 20.9/1
e.r. = 87.96 : 12.04 ; Enantiospecificity = 77.5%

A duplicate run gave:
Branched Product = 26%
Conversion = 77%
Protodehalogenated = 24%
Branched / Linear Ratio = 20.7/1
Enantiospecificity = 78.1 %ES

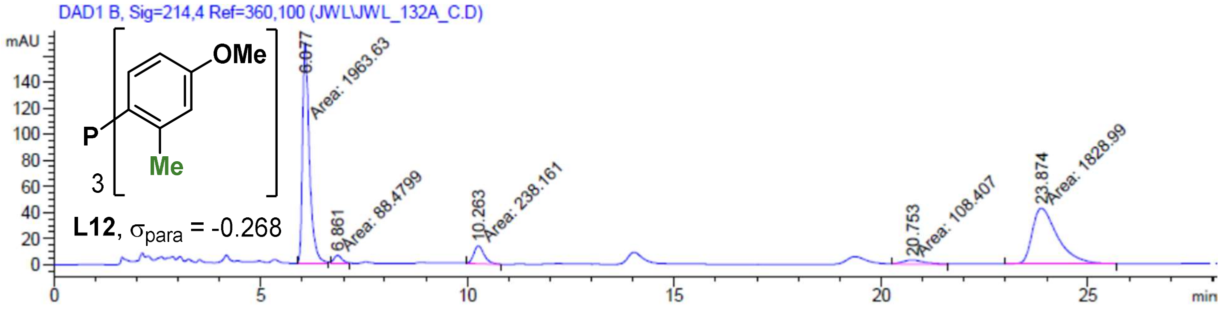

Response Factors Used
Branched Product = 0.01156 μmol / mAU, Linear Product = 0.01156 μmol / mAU, Aryl Bromide = 0.01103 μmol / mAU, Protodehalogenated Product = 0.01505 μmol / mAU

Quantity of 4-bromobiphenyl used = 23.2 mg = 0.0996 mmol
Branched Product = 26% yield
Conversion = 99%
Protodehalogenated = 28%
Branched/Linear Ratio = 20.3/1
e.r. = 89.18 : 10.82 ; Enantiospecificity = 80.0%

A duplicate run gave:
Branched Product = 26%
Conversion = 70%
Protodehalogenated = 16%
Branched / Linear Ratio = 15.7/1
Enantiospecificity = 79.8%

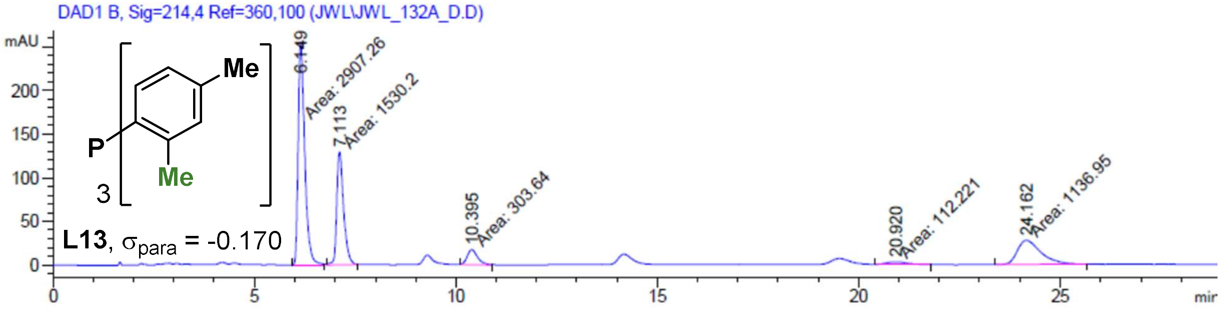

Response Factors Used
Branched Product = 0.01156 μmol / mAU, Linear Product = 0.01156 μmol / mAU, Aryl Bromide = 0.01103 μmol / mAU, Protodehalogenated Product = 0.01505 μmol / mAU

Quantity of 4-bromobiphenyl used = 23.5 mg = 0.1009 mmol
Branched Product = 37% yield
Conversion = 83%
Protodehalogenated = 17%
Branched/Linear Ratio = 28.6/1
e.r. = 90.54 : 9.46 ; Enantiospecificity = 82.7%

A duplicate run gave:
Branched Product = 38%
Conversion = 83%
Protodehalogenated = 18%
Branched / Linear Ratio = 29.0/1
Enantiospecificity = 82.5%

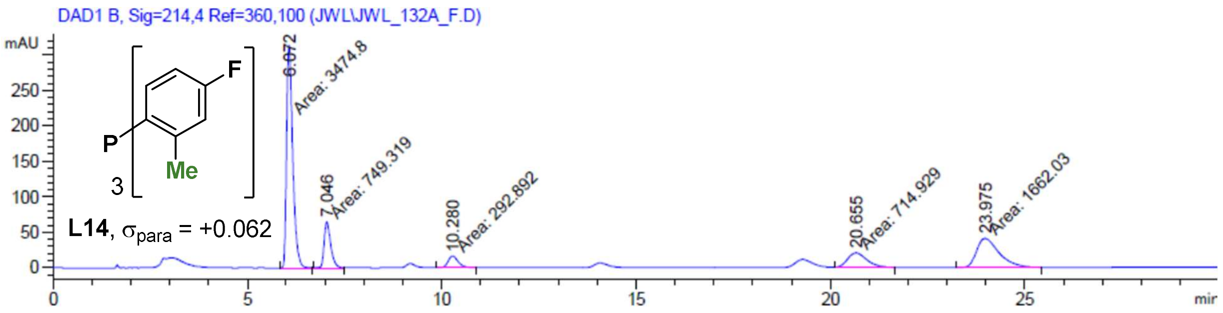

Response Factors Used
Branched Product = 0.01156 µmol / mAU, Linear Product = 0.01156 µmol / mAU, Aryl Bromide = 0.01103 µmol / mAU, Protodehalogenated Product = 0.01505 µmol / mAU

Quantity of 4-bromobiphenyl used = 23.3 mg = 0.1000 mmol
Branched Product = 44% yield
Conversion = 92%
Protodehalogenated = 25%
Branched/Linear Ratio = 5.3/1
e.r. = 92.23 : 7.77; Enantiospecificity = 86.2%

A duplicate run gave:
Branched Product = 39%
Conversion = 90%
Protodehalogenated = 27%
Branched / Linear Ratio = 5.4/1
Enantiospecificity = 86.4%

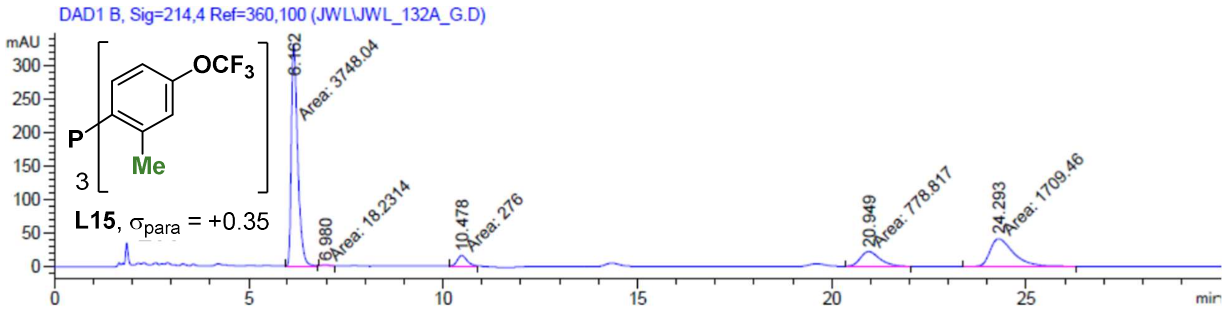

Response Factors Used
Branched Product = 0.01156 µmol / mAU, Linear Product = 0.01156 µmol / mAU, Aryl Bromide = 0.01103 µmol / mAU, Protodehalogenated Product = 0.01505 µmol / mAU

Quantity of 4-bromobiphenyl used = 23.9 mg = 0.1026 mmol
Branched Product = 45% yield
Conversion = 100%
Protodehalogenated = 25%
Branched/Linear Ratio = 5.2/1
e.r. = 93.14 : 6.86; Enantiospecificity = 88.0%

A duplicate run gave:
Branched Product = 46%
Conversion = 100%
Protodehalogenated = 24%
Branched / Linear Ratio = 5.9/1
Enantiospecificity = 88.2%

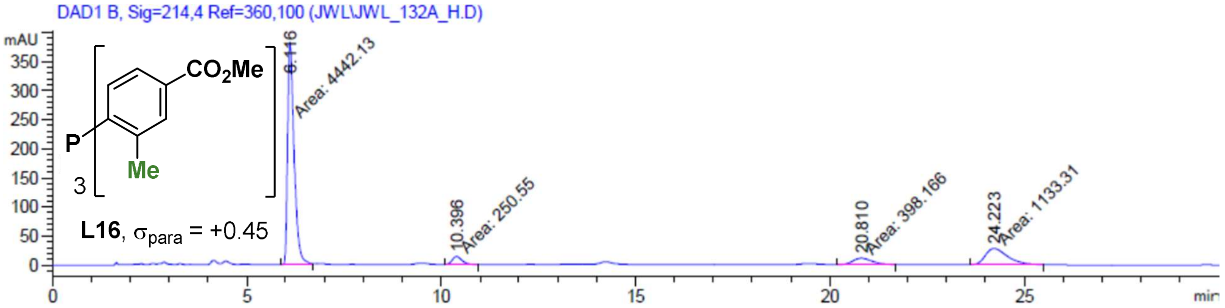

Response Factors Used
Branched Product = 0.01156 µmol / mAU, Linear Product = 0.01156 µmol / mAU, Aryl Bromide = 0.01103 µmol / mAU, Protodehalogenated Product = 0.01505 µmol / mAU

Quantity of 4-bromobiphenyl used = 23.4 mg = 0.1004 mmol
Branched Product = 54% yield
Conversion = 100%
Protodehalogenated = 17%
Branched/Linear Ratio = 11.8/1
e.r. = 94.66 : 5.34; Enantiospecificity = 91.1 %

A duplicate run gave:
Branched Product = 54%
Conversion = 100%
Protodehalogenated = 18%
Branched / Linear Ratio = 10.5/1
Enantiospecificity = 91.0%

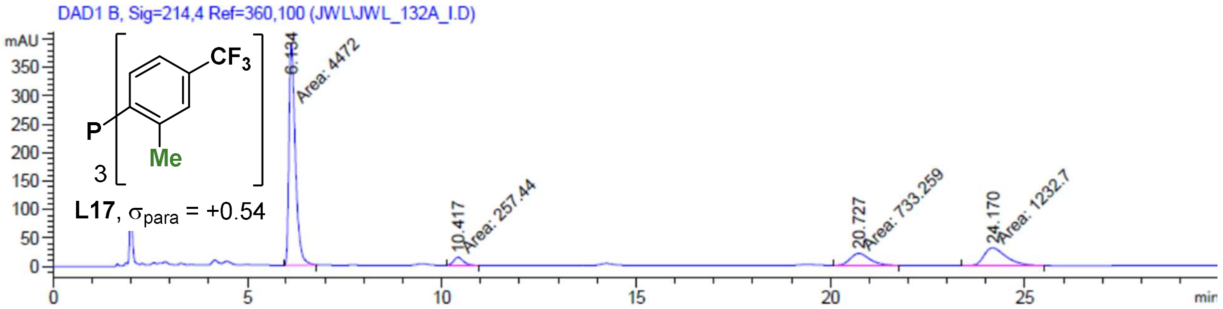

Response Factors Used
Branched Product = 0.01156 µmol / mAU, Linear Product = 0.01156 µmol / mAU, Aryl Bromide = 0.01103 µmol / mAU, Protodehalogenated Product = 0.01505 µmol / mAU

Quantity of 4-bromobiphenyl used = 23.8 mg = 0.1021 mmol
Branched Product = 54% yield
Conversion = 100%
Protodehalogenated = 18%
Branched/Linear Ratio = 6.4/1
e.r. = 94.56 : 5.44; Enantiospecificity = 90.9%

A duplicate run gave:
Branched Product = 51%
Conversion = 100%
Protodehalogenated = 17%
Branched / Linear Ratio = 6.2/1
Enantiospecificity = 90.6%

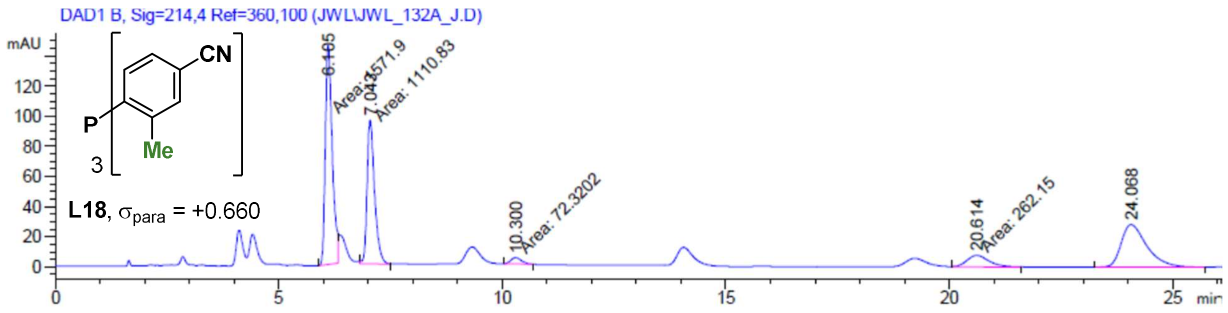

Response Factors Used
Branched Product = 0.01156 µmol / mAU, Linear Product = 0.01156 µmol / mAU, Aryl Bromide = 0.01103 µmol / mAU, Protodehalogenated Product = 0.01505 µmol / mAU

Quantity of 4-bromobiphenyl used = 23.8 mg = 0.1000 mmol
Branched Product = 19% yield
Conversion = 88%
Protodehalogenated = 17%
Branched/Linear Ratio = 6.3/1
e.r. = 95.60 : 4.40; Enantiospecificity = 93.1%

A duplicate run gave:
Branched Product = 19%
Conversion = 80%
Protodehalogenated = 15%
Branched / Linear Ratio = 7.3/1
Enantiospecificity = 93.5%

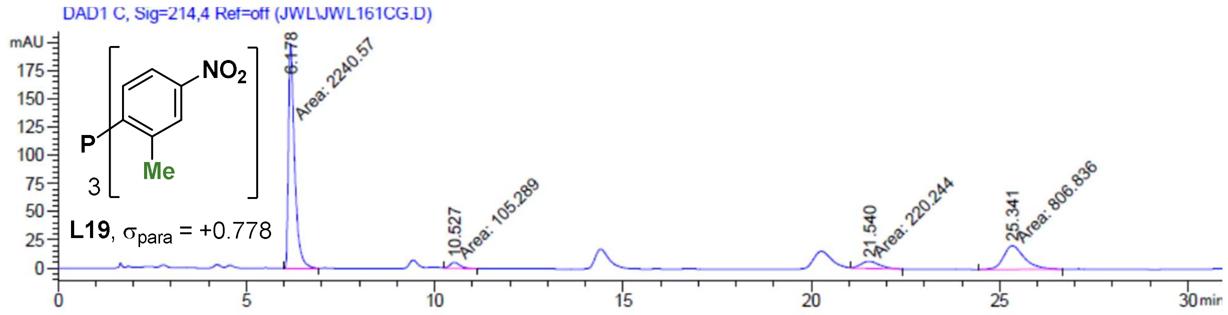

Response Factors Used
Branched Product = 0.01157 µmol / mAU, Linear Product = 0.01157 µmol / mAU, Aryl Bromide = 0.01113 µmol / mAU, Protodehalogenated Product = 0.01493 µmol / mAU

Quantity of 4-bromobiphenyl used = 25.6 mg = 0.1098 mmol
Branched Product = 25% yield
Conversion = 100%
Protodehalogenated = 11%
Branched/Linear Ratio = 10.7/1
e.r. = 95.51 : 4.49; Enantiospecificity = 92.9%

A duplicate run gave:
Branched Product = 28%
Conversion = 100%
Protodehalogenated = 12%
Branched / Linear Ratio = 11.2/1
Enantiospecificity = 92.9%

P(2-Bn-4-X-Ph)$_3$ Derivatives; Anhydrous Conditions

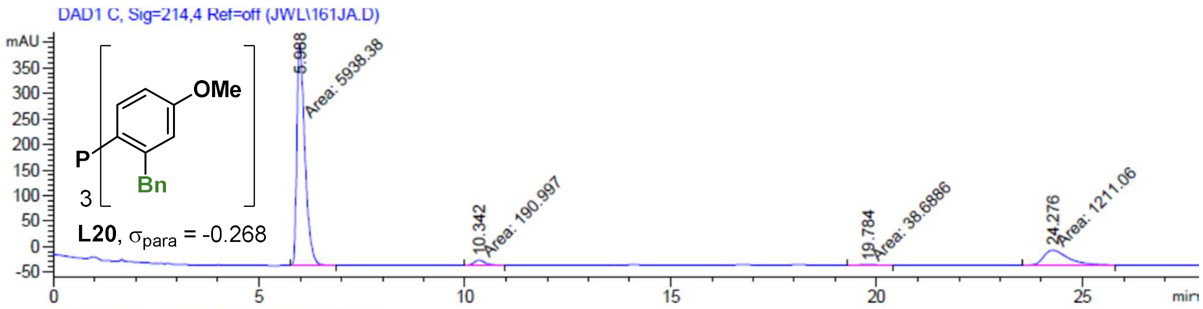

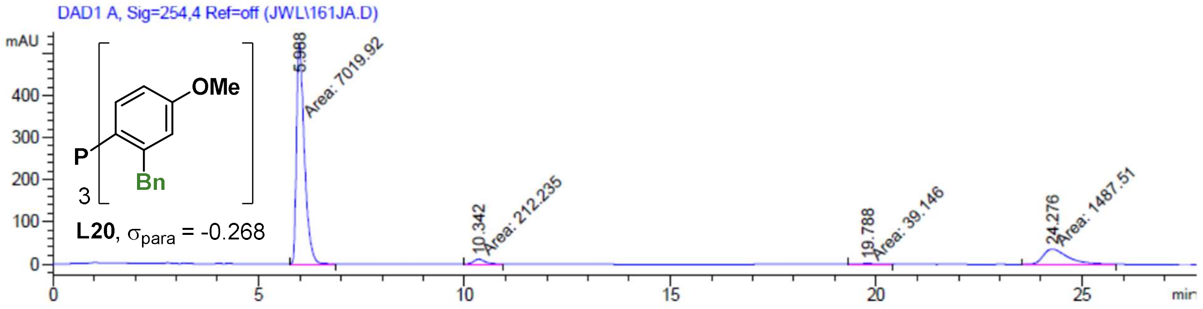

Response Factors Used
Branched Product = 0.01176 μmol / mAU, Linear Product = 0.01176 μmol / mAU, Aryl Bromide = 0.01122 μmol / mAU, Protodehalogenated Product = 0.01518 μmol / mAU

Quantity of 4-bromobiphenyl used = 23.4 mg = 0.1004 mmol
Branched Product = 72% yield
Conversion = 100%
Protodehalogenated = 18%
Branched/Linear Ratio = 158/1
e.r. = 97.07 : 2.93 (determined from cleaner 254.4 nm trace)
Enantiospecificity = 96.1%

A duplicate run gave:
Branched Product = 73%
Conversion = 100%
Protodehalogenated = 17%
Branched/Linear Ratio = 181/1
Enantiospecificity = 96.0%

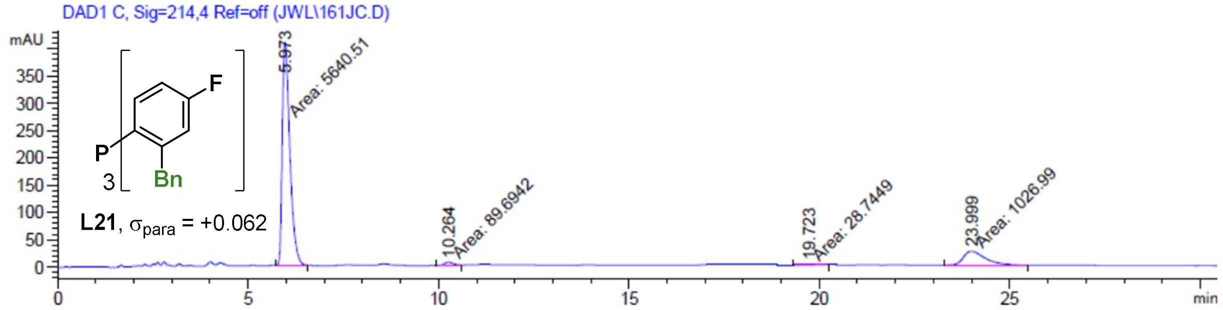

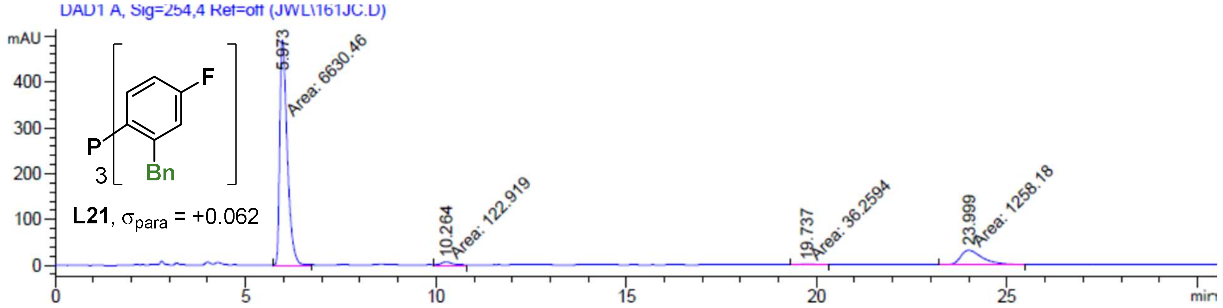

Response Factors Used
Branched Product = 0.01176 µmol / mAU, Linear Product = 0.01176 µmol / mAU, Aryl Bromide = 0.01122 µmol / mAU, Protodehalogenated Product = 0.01518 µmol / mAU

Quantity of 4-bromobiphenyl used = 23.7 mg = 0.1017 mmol
Branched Product = 66% yield
Conversion = 100%
Protodehalogenated = 15%
Branched/Linear Ratio = 199/1
e.r. = 98.18 : 1.82 (determined from cleaner 254.4 nm trace)
Enantiospecificity = 98.3%

A duplicate run gave:
Branched Product = 66%
Conversion = 100%
Protodehalogenated = 13%
Branched/Linear Ratio = 205/1
Enantiospecificity = 98.4%

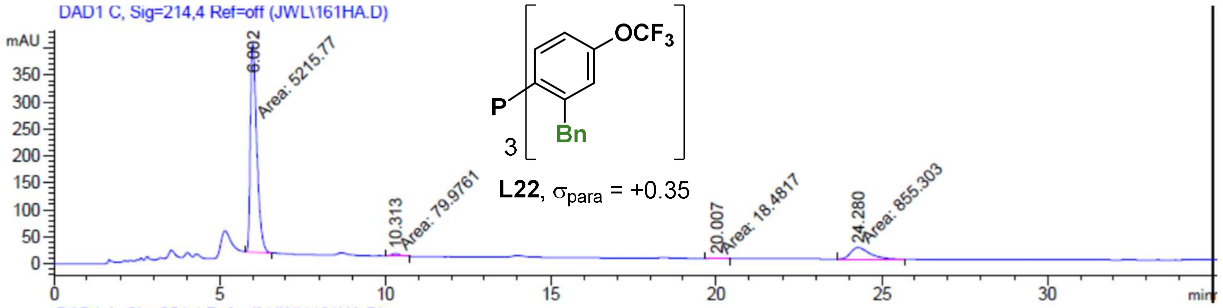

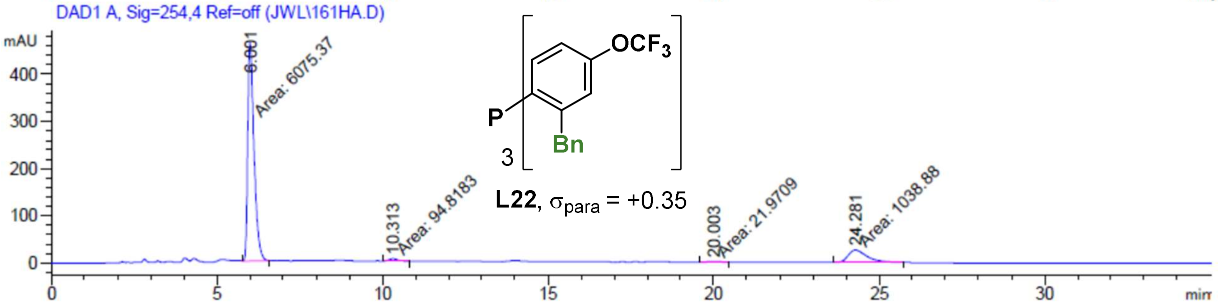

Response Factors Used
Branched Product = 0.01131 µmol / mAU, Linear Product = 0.01131 µmol / mAU, Aryl Bromide = 0.01078 µmol / mAU, Protodehalogenated Product = 0.01459 µmol / mAU

Quantity of 4-bromobiphenyl used = 23.7 mg = 0.1017 mmol
Branched Product = 59% yield
Conversion = 100%
Protodehalogenated = 12%
Branched/Linear Ratio = 287/1
e.r. = 98.46 : 1.54 (determined from cleaner 254.4 nm trace)
Enantiospecificity = 98.9%

A duplicate run gave:
Branched Product = 56%
Conversion = 100%
Protodehalogenated = 13%
Branched/Linear Ratio = 359/1
Enantiospecificity = 98.8%

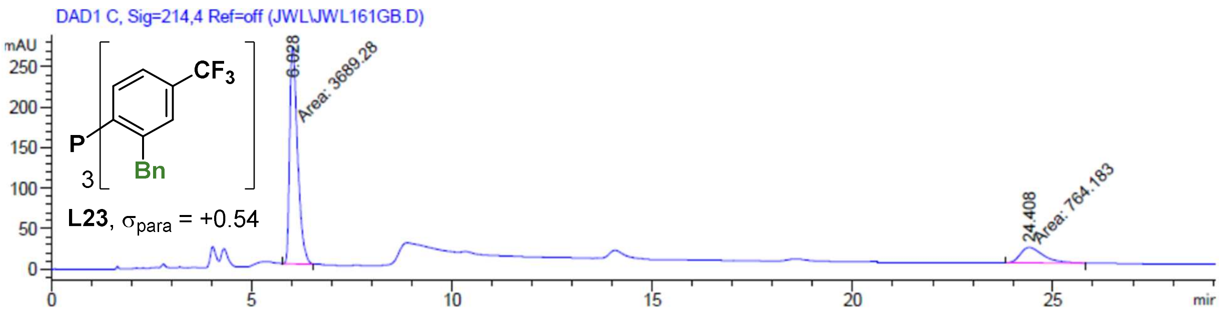

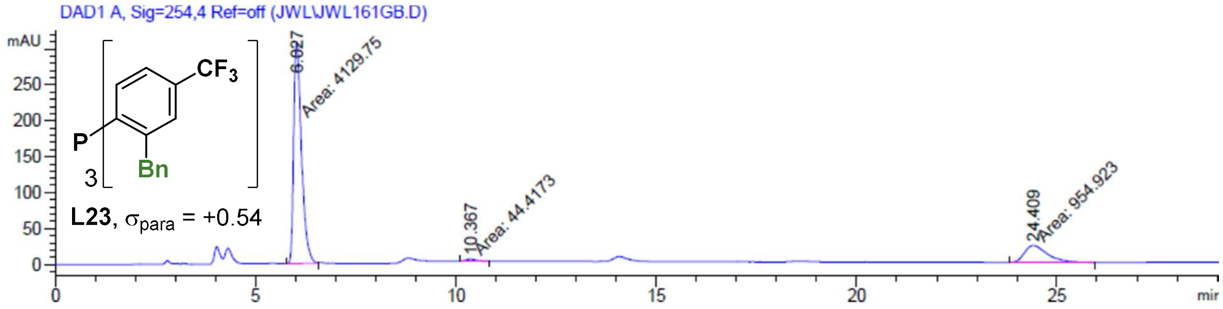

Response Factors Used
Branched Product = 0.01174 µmol / mAU, Linear Product = 0.01174 µmol / mAU, Aryl Bromide = 0.01119 µmol / mAU, Protodehalogenated Product = 0.01515 µmol / mAU

Quantity of 4-bromobiphenyl used = 24.2 mg = 0.1038 mmol
Branched Product = 42% yield
Conversion = 100%
Protodehalogenated = 11%
No linear product detectable
e.r. = 98.94 : 1.06 (determined from cleaner 254.4 nm trace)
Enantiospecificity = 99.9%

A duplicate run gave:
Branched Product = 51%
Conversion = 100%
Protodehalogenated = 8%
No linear product detectable
Enantiospecificity = 99.4%

P(2-Me-4-X-Ph)$_3$ Derivatives; Aqueous Biphasic Conditions

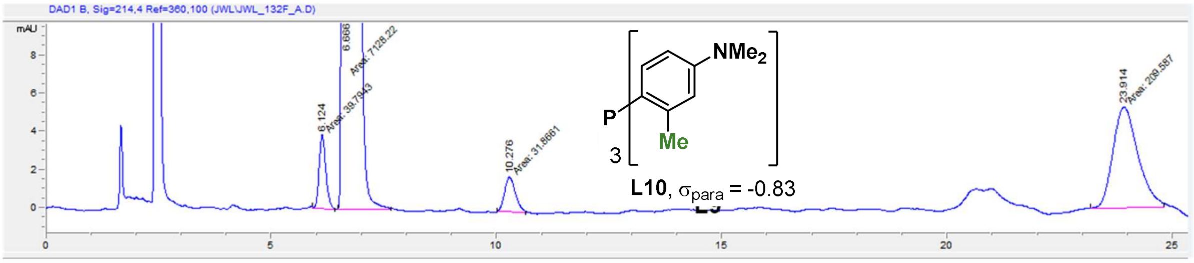

Response Factors Used
Branched Product = 0.01146 µmol / mAU, Linear Product = 0.01146 µmol / mAU, Aryl Chloride = 0.01223 µmol / mAU, Protodehalogenated Product = 0.01479 µmol / mAU

Quantity of 4-chlorobiphenyl used = 18.2 mg = 0.09647 mmol
Branched Product = 0.8% yield
Conversion = 10%
Protodehalogenated = 3%
Branched/Linear Ratio Not Determined
e.r. = 55.53 : 44.47 ; Enantiospecificity = 11.3%

A duplicate run gave:
Branched Product = 0.8%
Conversion = 10%
Protodehalogenated = 2%
Branched / Linear Ratio Not Determined
Enantiospecificity = 6.1 %ES

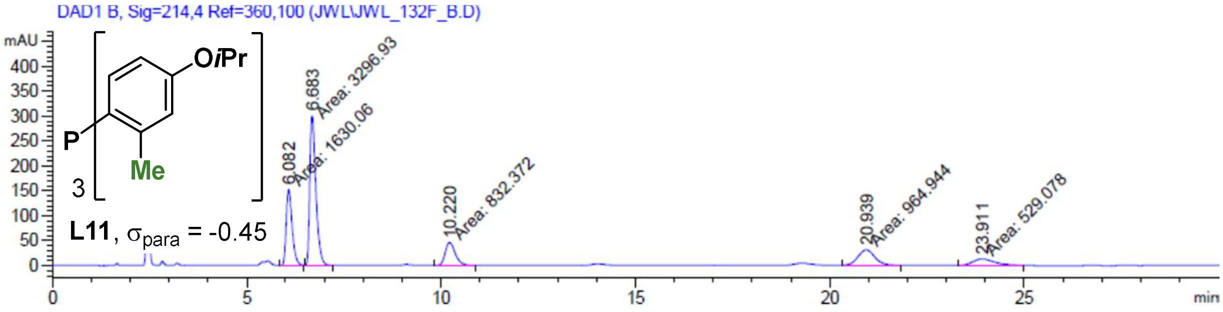

Response Factors Used
Branched Product = 0.01146 µmol / mAU, Linear Product = 0.01146 µmol / mAU, Aryl Chloride = 0.01223 µmol / mAU, Protodehalogenated Product = 0.01479 µmol / mAU

Quantity of 4-chlorobiphenyl used = 19.1 mg = 0.1012 mmol
Branched Product = 28% yield
Conversion = 60%
Protodehalogenated = 7%
Branched/Linear Ratio = 2.6/1
e.r. = 66.20 : 33.80; Enantiospecificity = 33.1%

A duplicate run gave:
Branched Product = 22%
Conversion = 50%
Protodehalogenated = 6%
Branched / Linear Ratio = 2.6/1
Enantiospecificity = 32.3 %ES

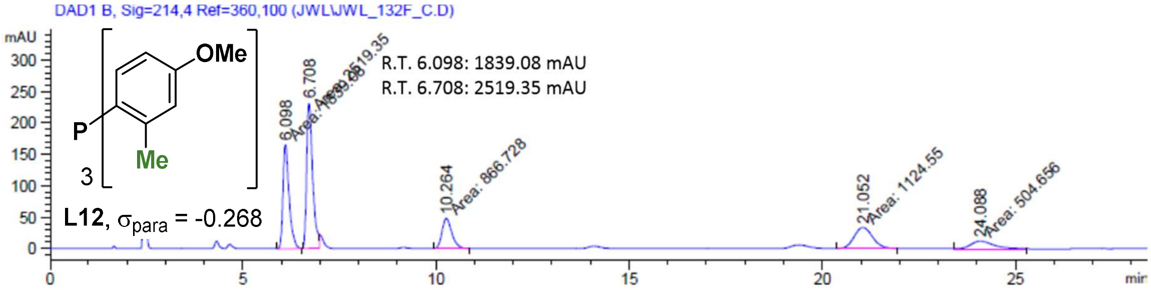

Response Factors Used
Branched Product = 0.01146 µmol / mAU, Linear Product = 0.01146 µmol / mAU, Aryl Chloride = 0.01223 µmol / mAU, Protodehalogenated Product = 0.01479 µmol / mAU

Quantity of 4-chlorobiphenyl used = 18.7 mg = 0.09912 mmol
Branched Product = 31% yield
Conversion = 69%
Protodehalogenated = 8%
Branched/Linear Ratio = 2.4/1
e.r. = 67.97 : 32.03; Enantiospecificity = 36.7%

A duplicate run gave:
Branched Product = 27%
Conversion = 59%
Protodehalogenated = 5%
Branched / Linear Ratio = 2.2/1
Enantiospecificity = 33.5 %ES

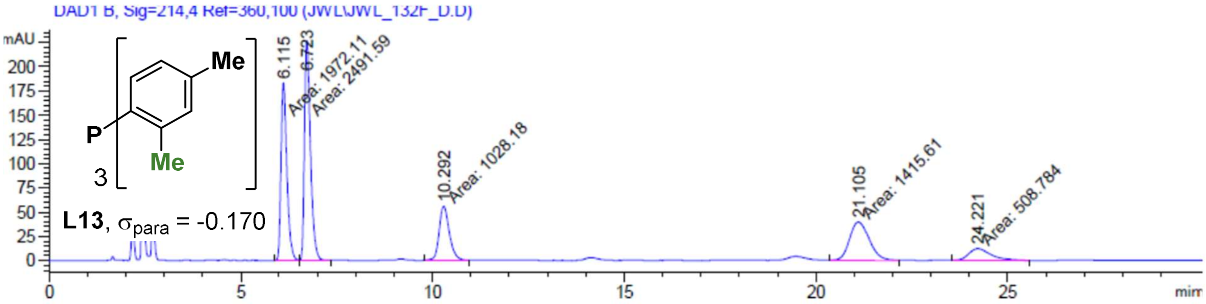

Response Factors Used
Branched Product = 0.01146 µmol / mAU, Linear Product = 0.01146 µmol / mAU, Aryl Chloride = 0.01223 µmol / mAU, Protodehalogenated Product = 0.01479 µmol / mAU

Quantity of 4-chlorobiphenyl used = 19.1 mg = 0.1012 mmol
Branched Product = 34% yield
Conversion = 70%
Protodehalogenated = 7%
Branched/Linear Ratio = 2.1/1
e.r. = 65.73 : 34.27; Enantiospecificity = 32.1%

A duplicate run gave:
Branched Product = 32%
Conversion = 68%
Protodehalogenated = 4%
Branched / Linear Ratio = 2.6/1
Enantiospecificity = 34.5 %ES

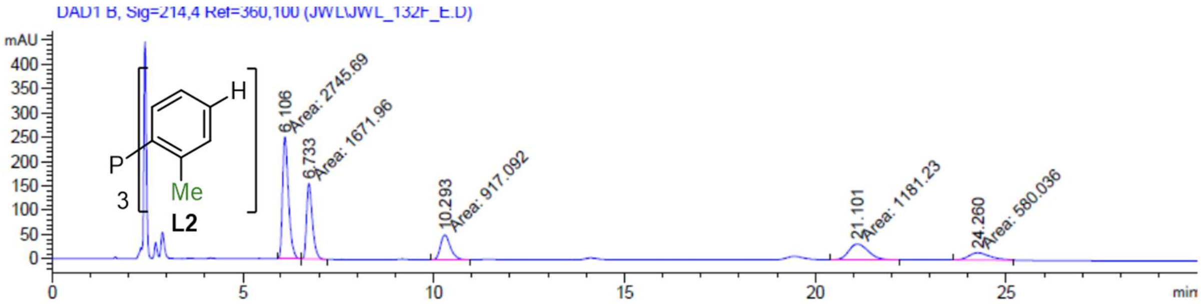

Response Factors Used
Branched Product = 0.01146 µmol / mAU, Linear Product = 0.01146 µmol / mAU, Aryl Chloride = 0.01223 µmol / mAU, Protodehalogenated Product = 0.01479 µmol / mAU

Quantity of 4-chlorobiphenyl used = 18.8 mg = 0.09965 mmol
Branched Product = 42% yield
Conversion = 79%
Protodehalogenated = 9%
Branched/Linear Ratio = 3.1/1
e.r. = 74.96 : 25.04; Enantiospecificity = 50.9%

A duplicate run gave:
Branched Product = 31%
Conversion = 67%
Protodehalogenated = 5%
Branched / Linear Ratio = 2.4/1
Enantiospecificity = 43.2 %ES

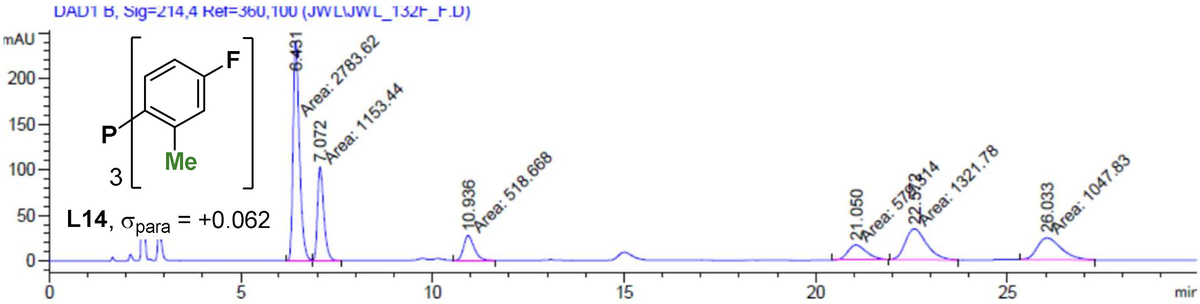

Response Factors Used
Branched Product = 0.01146 µmol / mAU, Linear Product = 0.01146 µmol / mAU, Aryl Chloride = 0.01223 µmol / mAU, Protodehalogenated Product = 0.01479 µmol / mAU

Quantity of 4-chlorobiphenyl used = 19.8 mg = 0.1050 mmol
Branched Product = 36% yield
Conversion = 87%
Protodehalogenated = 15%
Branched/Linear Ratio = 2.5/1
e.r. = 84.29 : 15.71; Enantiospecificity = 70.0%

A duplicate run gave:
Branched Product = 35%
Conversion = 82%
Protodehalogenated = 11%
Branched / Linear Ratio = 2.1/1
Enantiospecificity = 64.2 %ES

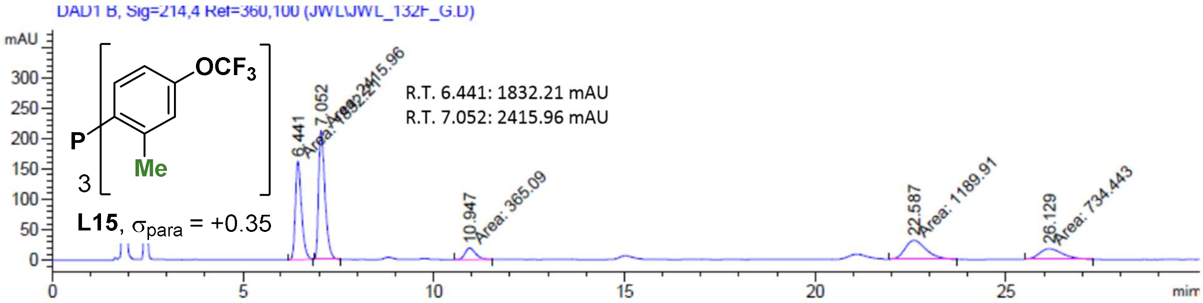

Response Factors Used
Branched Product = 0.01146 μmol / mAU, Linear Product = 0.01146 μmol / mAU, Aryl Chloride = 0.01223 μmol / mAU, Protodehalogenated Product = 0.01479 μmol / mAU

Quantity of 4-chlorobiphenyl used = 18.7 mg = 0.09912 mmol
Branched Product = 25% yield
Conversion = 70%
Protodehalogenated = 11%
Branched/Linear Ratio = 1.85/1
e.r. = 83.38 : 16.62; Enantiospecificity = 68.1%

A duplicate run gave:
Branched Product = 27%
Conversion = 72%
Protodehalogenated = 10%
Branched / Linear Ratio = 1.96/1
Enantiospecificity = 68.8 %ES

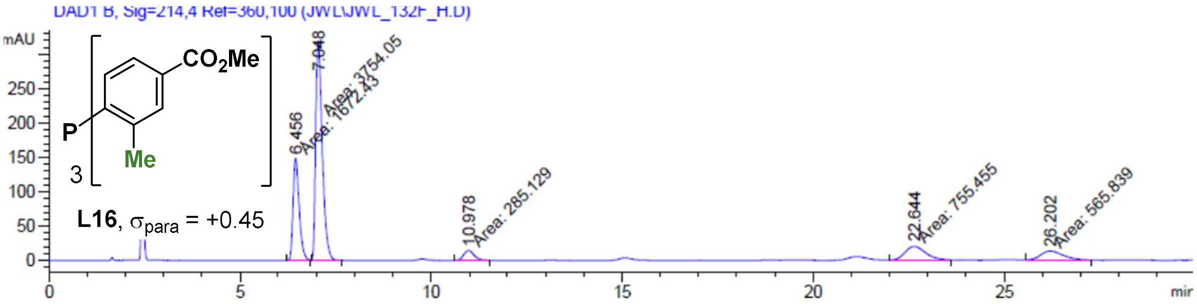

Response Factors Used
Branched Product = 0.01146 μmol / mAU, Linear Product = 0.01146 μmol / mAU, Aryl Chloride = 0.01223 μmol / mAU, Protodehalogenated Product = 0.01479 μmol / mAU

Quantity of 4-chlorobiphenyl used = 18.9 mg = 0.1002 mmol
Branched Product = 22% yield
Conversion = 54%
Protodehalogenated = 8%
Branched/Linear Ratio = 2.6/1
e.r. = 85.43 : 14.57; Enantiospecificity = 72.3%

A duplicate run gave:
Branched Product = 23%
Conversion = 51%
Protodehalogenated = 8%
Branched / Linear Ratio = 2.6/1
Enantiospecificity = 74.5 %ES

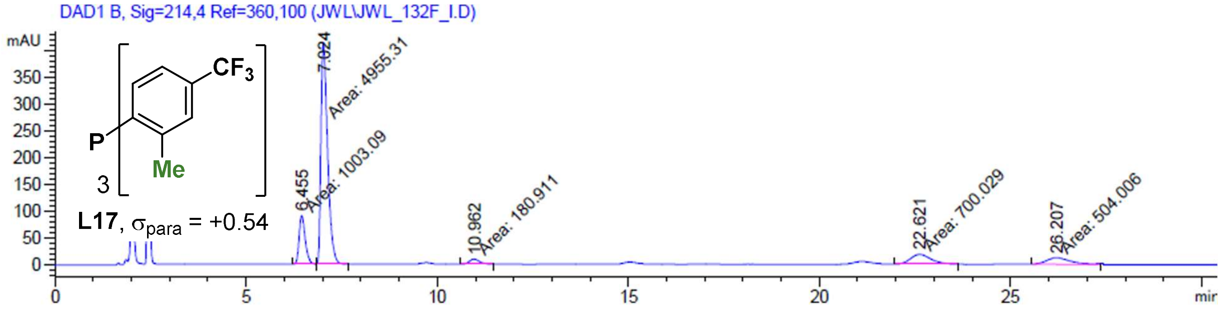

Response Factors Used
Branched Product = 0.01146 µmol / mAU, Linear Product = 0.01146 µmol / mAU, Aryl Chloride = 0.01223 µmol / mAU, Protodehalogenated Product = 0.01479 µmol / mAU

Quantity of 4-chlorobiphenyl used = 18.8 mg = 0.09965 mmol
Branched Product = 14% yield
Conversion = 39%
Protodehalogenated = 7%
Branched/Linear Ratio = 1.69/1
e.r. = 84.72 : 15.28; Enantiospecificity = 70.9%

A duplicate run gave:
Branched Product = 14%
Conversion = 42%
Protodehalogenated = 7%
Branched / Linear Ratio = 1.85/1
Enantiospecificity = 73.2 %ES

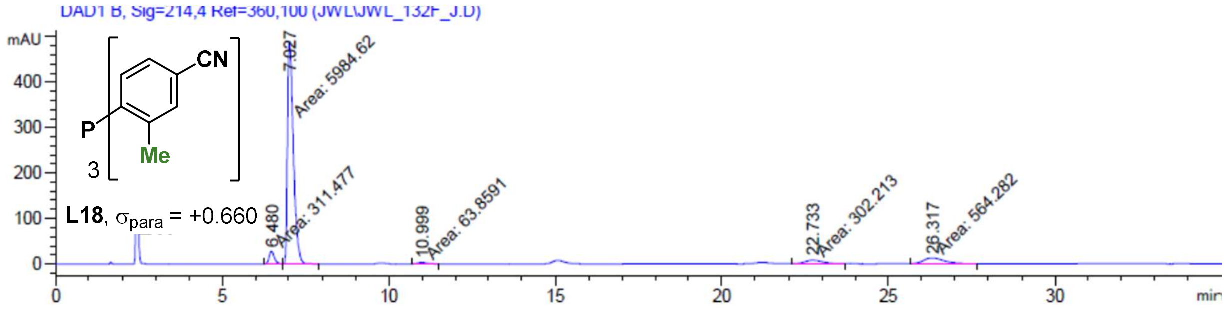

Response Factors Used
Branched Product = 0.01146 µmol / mAU, Linear Product = 0.01146 µmol / mAU, Aryl Chloride = 0.01223 µmol / mAU, Protodehalogenated Product = 0.01479 µmol / mAU

Quantity of 4-chlorobiphenyl used = 19.2 mg = 0.1018 mmol
Branched Product = 4.2% yield
Conversion = 28%
Protodehalogenated = 8%
Branched/Linear Ratio = 1.24/1
e.r. = 82.99 : 17.01; Enantiospecificity = 67.3%

A duplicate run gave:
Branched Product = 3.9%
Conversion = 28%
Protodehalogenated = 7%
Branched / Linear Ratio = 1.45/1
Enantiospecificity = 73.5 %ES

P(2-Bn-4-X-Ph)$_3$ Derivatives; Aqueous Biphasic Conditions

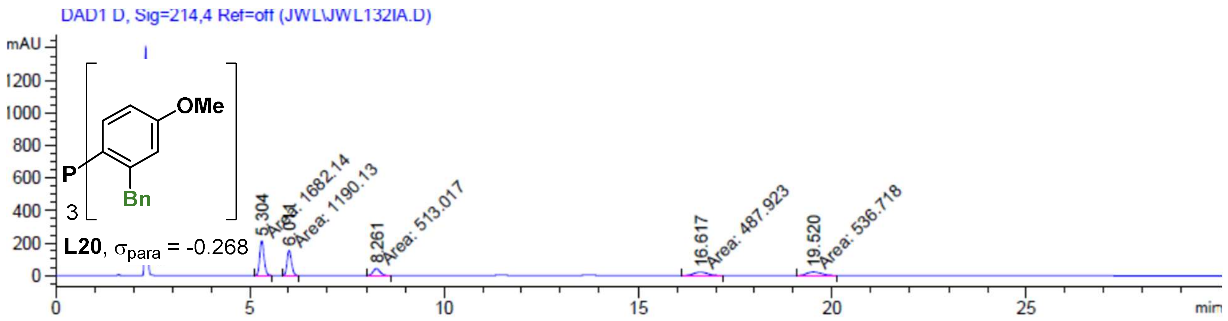

Response Factors Used
Branched Product = 0.01156 μmol / mAU, Linear Product = 0.01156 μmol / mAU, Aryl Chloride = 0.01223 μmol / mAU, Protodehalogenated Product = 0.01505 μmol / mAU

Quantity of 4-chlorobiphenyl used = 19.5 mg = 0.1034 mmol
Branched Product = 25% yield
Conversion = 86%
Protodehalogenated = 8%
Branched/Linear Ratio = 4.50/1
e.r. = 76.63 : 23.37; Enantiospecificity = 54.3%

A duplicate run gave:
Branched Product = 42%
Conversion = 82%
Protodehalogenated = 13%
Branched / Linear Ratio = 6.02/1
Enantiospecificity = 59.0 %ES

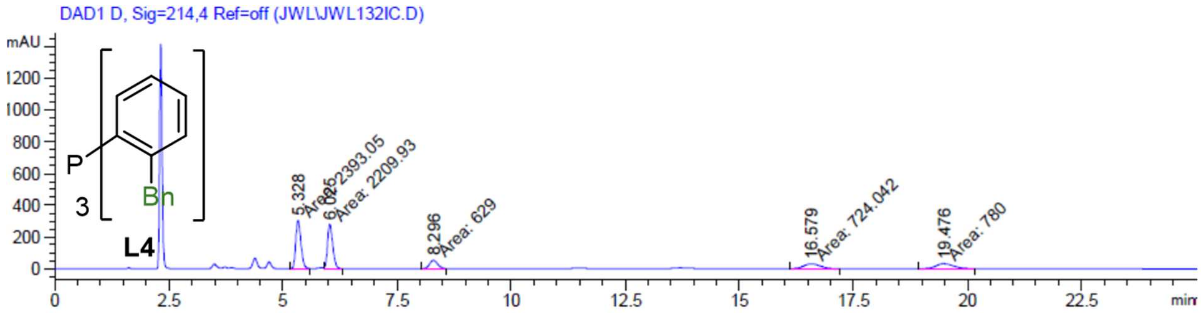

Response Factors Used
Branched Product = 0.01156 μmol / mAU, Linear Product = 0.01156 μmol / mAU, Aryl Chloride = 0.01223 μmol / mAU, Protodehalogenated Product = 0.01505 μmol / mAU

Quantity of 4-chlorobiphenyl used = 19.2 mg = 0.1018 mmol
Branched Product = 34% yield
Conversion = 73%
Protodehalogenated = 12%
Branched/Linear Ratio = 4.17/1
e.r. = 79.19 : 20.81; Enantiospecificity = 59.6%

A duplicate run gave:
Branched Product = 38%
Conversion = 77%
Protodehalogenated = 12%
Branched / Linear Ratio = 4.52/1
Enantiospecificity = 59.5 %ES

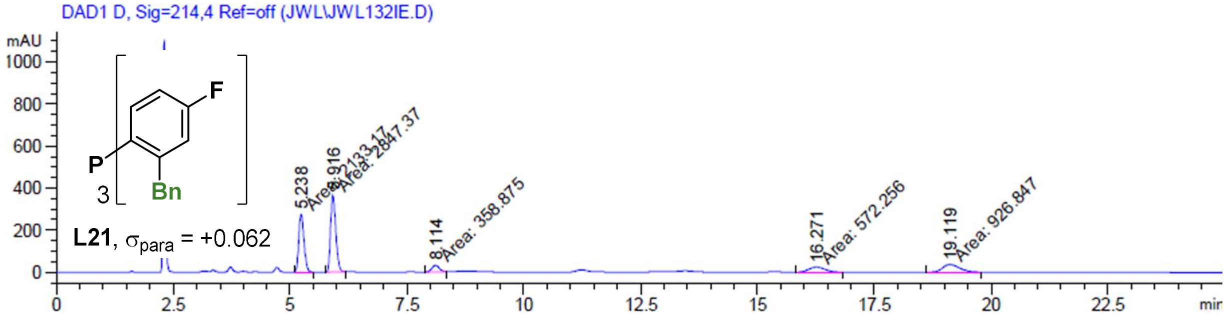

Response Factors Used
Branched Product = 0.01156 µmol / mAU, Linear Product = 0.01156 µmol / mAU, Aryl Chloride = 0.01223 µmol / mAU, Protodehalogenated Product = 0.01505 µmol / mAU

Quantity of 4-chlorobiphenyl used = 18.6 mg = 0.09860 mmol
Branched Product = 29% yield
Conversion = 65%
Protodehalogenated = 14%
Branched/Linear Ratio = 4.35/1
e.r. = 85.60 : 14.40; Enantiospecificity = 72.7%

A duplicate run gave:
Branched Product = 31%
Conversion = 69%
Protodehalogenated = 16%
Branched / Linear Ratio = 4.51/1
Enantiospecificity = 73.4 %ES

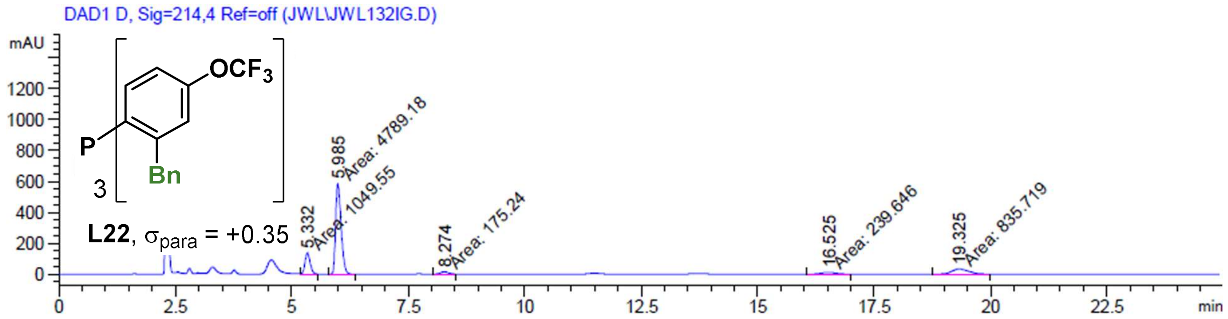

Response Factors Used
Branched Product = 0.01156 µmol / mAU, Linear Product = 0.01156 µmol / mAU, Aryl Chloride = 0.01223 µmol / mAU, Protodehalogenated Product = 0.01505 µmol / mAU

Quantity of 4-chlorobiphenyl used = 19.2 mg = 0.1018 mmol
Branched Product = 14% yield
Conversion = 42%
Protodehalogenated = 12%
Branched/Linear Ratio = 5.11/1
e.r. = 85.69 : 14.31; Enantiospecificity = 72.8%

A duplicate run gave:
Branched Product = 15%
Conversion = 42%
Protodehalogenated = 13%
Branched / Linear Ratio = 5.73/1
Enantiospecificity = 75.7 %ES

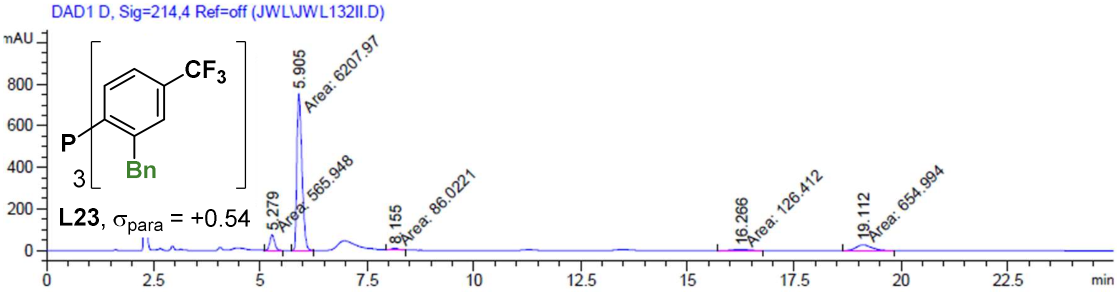

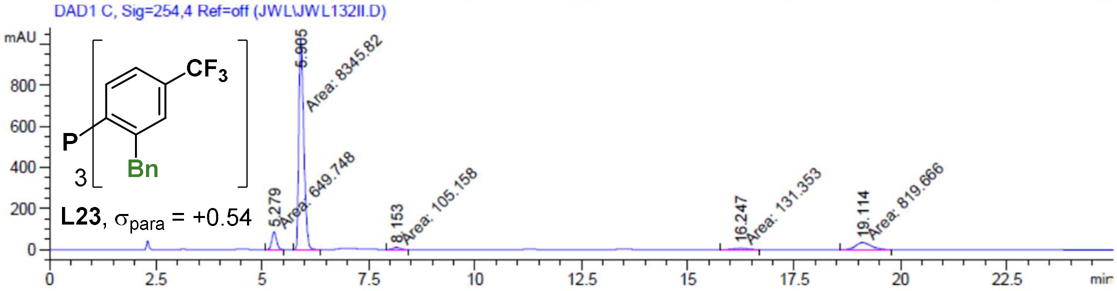

Response Factors Used
Branched Product = 0.01156 μmol / mAU, Linear Product = 0.01156 μmol / mAU, Aryl Chloride = 0.01223 μmol / mAU, Protodehalogenated Product = 0.01505 μmol / mAU

Quantity of 4-chlorobiphenyl used = 19.4 mg = 0.1028 mmol
Branched Product = 7% yield
Conversion = 26%
Protodehalogenated = 10%
Branched/Linear Ratio = 5.75/1 (determined from cleaner 254.4 nm trace)
e.r. = 86.07 : 13.93; Enantiospecificity = 73.6% (254.4 nm trace)

A duplicate run gave:
Branched Product = 7%
Conversion = 27%
Protodehalogenated = 9%
Branched / Linear Ratio = 5.85/1
Enantiospecificity = 73.8 %ES

P*t*Bu$_3$ Under Aqueous Biphasic Conditions

The BIDA boronate **5a**, 99:1 d.r., was hydrolyzed to the boronic acid **(*S*)-1a** as in general procedure B. This boronic acid was then converted to the potassium (*S*)-2-butyltrifluoroborate **(*S*)-1a'** by a reported method.[14] The chiral trifluoroborate salt was cross-coupled using the reaction conditions recently reported to proceed with stereoinversion.[15] The trifluoroborate salt (94 mg, 0.57 mmol, 1.5 eq), 4-chlorobiphenyl (72 mg, 0.38 mmol, 1.0 eq), the palladacycle[16] (11 mg, 0.019 mmol, 5 mol%), toluene (0.76 mL, 0.5 M), H_2O (0.38 mL, 1.0 M), and K_2CO_3 (157 mg, 1.14 mmol, 3.0 eq) were combined under argon in a 7 mL screw-cap vial with a stir bar. The vial was capped and stirred at 100°C for 24 hours. TLC (100% hexanes) showed complete conversion of the aryl chloride. The aqueous layer was extracted twice with hexanes. The combined organic layers were dried with sodium sulfate and concentrated under vacuum. The crude product was purified by silica column using 100% pentane, giving **(*R*)-3a** (65 mg, 0.309 mmol, 81% yield). NMR spectra were identical to those of the (*S*) enantiomer.

Using the potassium trifluoroborate salt made from BIDA boronate **5a** of 99:1 d.r., the coupling product **(*R*)-3a** had an e.r. of 96:4 (94% enantiospecificity), as determined by Chiralcel OD-H column, 100% hexanes, 2.0mL/min., 210nm absorbance. The absolute configuration of the major enantiomer was identified as (*R*), having identified the retention time of the (*S*) enantiomer by independent synthesis. Minor: 5.7, Major: 9.3. This independently confirms that the cross-coupling conditions developed by Biscoe results in *stereoinversion*.

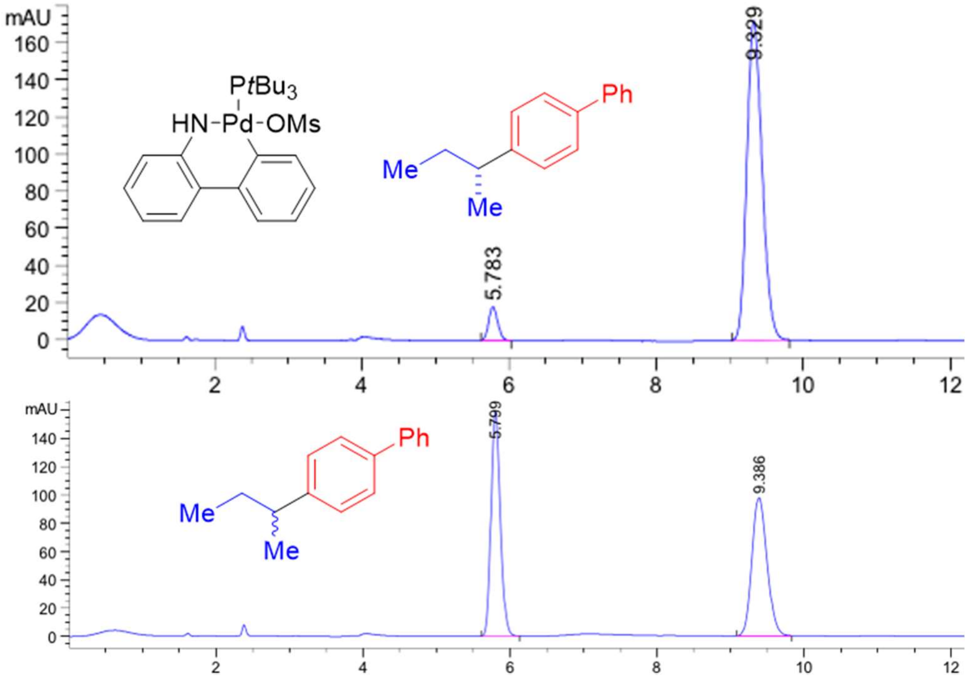

Synthesis and Characterization of Boron-Containing Compounds

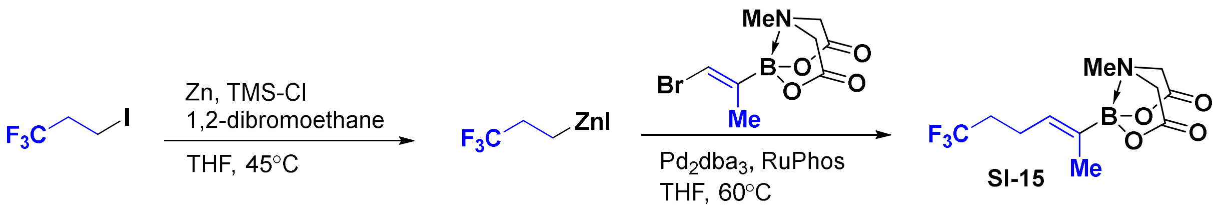

MIDA Boronate SI-15. To zinc dust (3.24 g, 49.5 mmol, 3.41 eq) in a nitrogen-purged 40 mL vial with stir bar were added trimethylsilyl chloride (100 µL, 120 mg, 1.1 mmol, 8 mol%) and 1,2-dibromoethane (100 µL, 220 mg, 1.2 mmol, 8 mol%) by syringe. Anhydrous THF (16 mL) was added, followed by 1-iodo-3,3,3-trifluoropropane (3.8 g, 17.0 mmol, 1.17 eq) portionwise at RT with stirring over 20 min, keeping the exotherm below 50 °C. The suspension was then stirred for 1 h at 50 °C. To a separate 100 mL round bottom flask purged with nitrogen containing a stir bar were added RuPhos (655 mg, 1.40 mmol, 10 mol%), Pd_2dba_3 (629 mg, 0.687 mmol, 5 mol%), and DMF (20 mL). This was stirred for 30 min at 23 °C. *Trans*-2-bromo-1-methylvinyl MIDA boronate (Aldrich cat. no. 763853, 3.79 g, 14.5 mmol, 1.00 eq) was then added to this solution in 13 mL DMF. The alkyl zinc suspension was filtered through a syringe filter and the filtrate was added to the DMF solution. The remaining alkylzinc solution was washed over with additional THF (2 mL). The reaction was then stirred for 24 h at 50 °C, after which time nearly complete conversion of the vinyl bromide was observed by TLC (C_{18} plate, 2:1 H_2O:MeCN, $KMnO_4$ stain). In a separatory funnel, the reaction was diluted with EtOAc and saturated aqueous NH_4Cl was added. The aqueous phase was extracted twice with EtOAc. The combined organic phase was washed three times with H_2O, each time adding a few mL of brine to break the emulsion. The organic phase was dried with Na_2SO_4, concentrated to a dark orange foam, then dissolved in DCM and adsorbed onto celite. The celite pad was loaded onto a C_{18} silica column of 100 mL volume, eluting with a gradient of 30% to 60% MeCN in H_2O. The product-containing fractions were combined and solid NaCl was added to induce phase separation. The organic phase was separated and the aqueous layer extracted with EtOAc (×2). The combined organic phases were dried and concentrated to give **SI-15** as a yellow solid (2.91 g, 9.93 mmol 68% yield).

^{1}H NMR (500 MHz, $CDCl_3$) δ 5.87 (t, J = 7.2 Hz, 1H), 3.81 (d, J = 16.3 Hz, 2H), 3.69 (d, J = 16.3 Hz, 2H), 2.79 (s, 3H), 2.41 (q, J = 7.3 Hz, 2H), 2.27 – 2.14 (m, 2H), 1.68 (dt, J = 1.6, 0.9 Hz, 3H).

^{13}C NMR (126 MHz, $CDCl_3$) δ166.90, 136.96, 127.02 (q, $J_{C\text{-}F}$ = 277.6 Hz), 61.77, 46.25, 33.06 (q, $J_{C\text{-}F}$ = 27.7 Hz), 21.23 (q, $J_{C\text{-}F}$ = 2.5 Hz), 14.39.

^{19}F NMR (470 MHz, $CDCl_3$) -66.33 (t, J = 11.0 Hz).

^{11}B NMR (128 MHz, $CDCl_3$) δ 11.02

HRMS (ESI+) Calculated for $C_{11}H_{16}NO_4BF_3$ $(M+H)^+$: 294.1124, Found: 294.1121

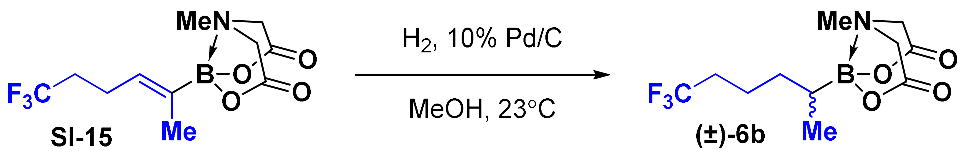

MIDA Boronate (±)-6b. A 200 mL round bottom flask was charged with a stir bar, **SI-15** (2.59 g, 8.84 mmol), and 10% Pd/C (1.3 g). The flask was sealed with a rubber septum and purged with nitrogen. Methanol (30 mL) was added via syringe. A balloon of hydrogen was affixed by needle and the headspace was purged with hydrogen. The balloon was refilled with hydrogen and affixed to the reaction again. The black suspension was stirred for two hours at 23°C. Monitoring the reaction by TLC (100% EtOAc, $KMnO_4$) showed complete conversion. The headspace was purged with nitrogen and the reaction was filtered through a silica plug twice, washing with EtOAc. The flow-through was concentrated to give **6b** as a white solid (2.34 g, 7.93 mmol, 90% yield).

^{1}H NMR (500 MHz, acetone-d_6) δ 4.20 (dd, J = 17.0, 4.5 Hz, 2H), 4.03 (dd, J = 17.0, 4.1 Hz, 2H), 3.16 (s, 3H), 2.28 – 2.07 (m, 2H), 1.81 – 1.45 (m, 3H), 1.27 (ddt, J = 12.1, 8.7, 4.9 Hz, 1H), 0.93 (m, 4H).

^{13}C NMR (126 MHz, acetone-d_6) δ 168.85, 168.69, 128.72 (q, J_{C-F} = 276.3 Hz), 63.47, 63.29, 45.20, 34.23 (q, J_{C-F} = 27.7 Hz), 32.15, 21.17 (q, J_{C-F} = 2.85 Hz), 14.53.

^{19}F NMR (470 MHz, acetone-d_6) δ -66.32 (t, J = 11.1 Hz).

^{11}B NMR (128 MHz, acetone-d_6) δ 13.81.

HRMS (ESI+) Calculated for $C_{11}H_{18}NO_4BF_3$ $(M+H)^+$: 296.1281, Found: 296.1277

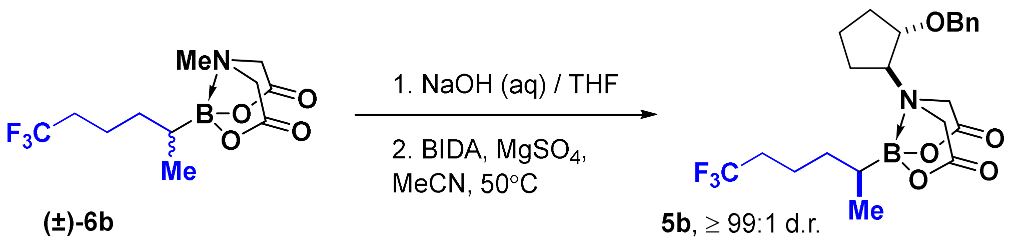

BIDA Boronate 5b. To a 100-mL round-bottom flask with a stir bar was added MIDA boronate **(±)-6b** (2.07 g, 7.01 mmol), THF (35 mL, 0.20 Molar) and freshly prepared 1M NaOH (35 mL, 5.0 eq). The mixture was stirred at 23 °C until complete conversion was confirmed by TLC (100% EtOAc, $KMnO_4$). THF was removed under rotary evaporation (bath temperature 40°C). When most of the THF was removed, the receiving flask was emptied and dried and rotary evaporation was then continued until water condensation began to collect in the receiving flask. Saturated NH_4Cl (35 mL) was added to the resulting aqueous solution and this was extracted with MTBE (4x35 mL) in a separatory funnel. The organic phase was dried over Na_2SO_4, filtered, and concentrated to an oil. The oil was combined with BIDA (2.15 g, 7.04 mmol, 1.0 eq), $MgSO_4$ (2.0 g, 16.6 mmol, 2.4 eq), and anhydrous MeCN (14 mL, 0.50 Molar) in a 40 mL vial and stirred at 50 °C overnight. The reaction was filtered through fluorosil in a glass frit, rinsing with EtOAc. The filtrate was concentrate to a white foam. The two diastereomers were

resolved by silica gel column (125 g silica, 1:1 Hex/EtOAc). The diastereomer with the higher R_f was isolated as **5b** (1.19 g, 2.61 mmol, 37%). The stereochemistry of the C2 center of **5b** was assigned by analogy to the other BIDA boronates resolved by silica gel column.

^{1}H-NMR in $CDCl_3$ showed a diastereomeric ratio of ≥99:1 by integrating the methyl doublets of **5b** and ***epi*-5b** at 0.87 and 0.98 ppm, respectively. The absolute stereochemistry at the boron-bearing carbon was tentatively assigned based on analogy to other BIDA boronates.

^{1}H NMR (500 MHz, acetone-d_6) δ 7.44 – 7.29 (m, 5H), 4.65 (d, J = 10 Hz, 1H), 4.58 (d, J = 10 Hz, 1H), 4.41 (q, J = 6.5 Hz, 1H), 4.13 (d, J = 17.6 Hz, 1H), 4.10, (s, 2H), 3.97 (d, J = 17.6 Hz, 1H), 3.77 (td, J = 8.7, 6.2 Hz, 1H), 2.34 – 2.08 (m, 4H), 1.89 – 1.76 (m, 3H), 1.76 – 1.47 (m, 4H), 1.28 (qd, J = 9.1, 4.4 Hz, 1H), 1.11 – 0.90 (m, 1H), 0.87 (d, J = 7.0 Hz, 3H).

^{13}C NMR (126 MHz, acetone-d_6) δ169.97, 168.15, 138.95, 129.20, 128.84, 128.71 (q, $J_{C\text{-}F}$ = 276.3 Hz), 128.57, 81.11, 73.37, 72.33, 61.04, 57.08, 34.19 (q, $J_{C\text{-}F}$ = 27.8 Hz), 32.49, 30.49, 27.31, 22.07, 20.98 (q, $J_{C\text{-}F}$ = 2.7 Hz), 14.98.

^{19}F NMR (470 MHz, acetone-d_6) δ -66.27 (t, J = 11.6 Hz).

^{11}B NMR (128 MHz, acetone-d_6) δ 14.13.

$[\alpha]^{20}_{D}$ = -6.4 (c 1.46, $CHCl_3$)

HRMS (ESI+) Calculated for $C_{22}H_{30}NO_5BF_3$ $(M+H)^+$: 456.2169, Found: 456.2170

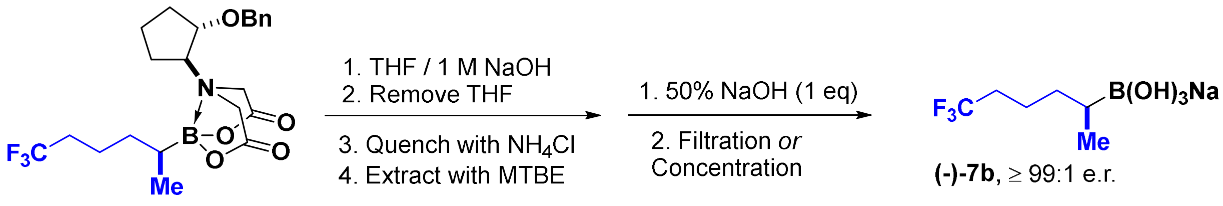

5b, ≥ 99:1 d.r.

Sodium alkyltrihydroxyborate (-)-7b was made from BIDA boronate **5b** according to general procedure B. The product was isolated by concentration *in vacuo* (0.268 g, 1.20 mmol, quantitative yield, white solid).

^{1}H NMR (500 MHz, CD_3OD) 2.12 (dtd, J = 14.6, 11.4, 5.1 Hz, 1H), 2.06 – 1.91 (m, 1H), 1.66 (ddt, J = 16.0, 10.7, 5.2 Hz, 1H), 1.60 – 1.47 (m, 1H), 1.39 (ddq, J = 17.2, 11.5, 5.7 Hz, 1H), 1.15 – 1.04 (m, 1H), 0.82 (d, J = 7.1 Hz, 3H), 0.46 (s, 1H).

^{13}C NMR (126 MHz, CD_3OD) δ 129.34 (q, $J_{C\text{-}F}$ = 275.7 Hz), 35.26 (q, $J_{C\text{-}F}$ = 27.7 Hz), 34.20, 22.87 (q, $J_{C\text{-}F}$ = 2.5 Hz), 16.18.

^{11}B NMR (128 MHz, CD_3OD) δ 6.66.

^{19}F NMR (470 MHz, CD_3OD) δ -67.49 (t, J = 11.0 Hz)

$[\alpha]^{20}_{D}$ = -15.9 (c = 1.53, CD3OD)

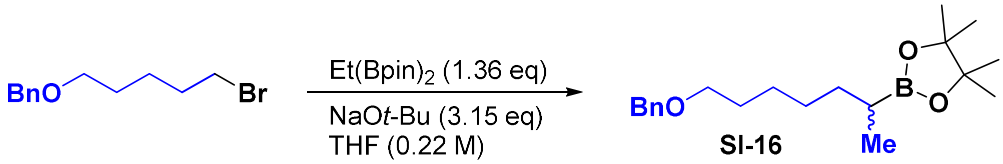

Pinacol boronic ester SI-16. The pinacol boronic ester was synthesized according to a modified literature procedure.[17] In an argon-filled glovebox, sodium *tert*-butoxide (9.652 g, 100.4 mmol, 3.15 eq) was added to a dry, stir bar-equipped 500 mL round bottom flask. In a separate dry 300 mL round bottom flask, a mixture was prepared containing $Et(Bpin)_2$ (12.23 g, 43.37 mmol, 1.36 eq) and 5-benzyloxypentyl bromide[18] (8.20 g, 31.9 mmol, 1.00 eq). Both flasks were sealed with rubber septa, brought out into a fume hood, and connected to nitrogen lines. To the flask containing NaO*t*-Bu was added anhydrous THF (97 mL), and the suspension was cooled to 0°C in an ice / water bath. Additional THF (25 mL) was added to the $Et(Bpin)_2$ / alkyl bromide mixture, and the resulting solution was transferred (gradually over six minutes, using an additional 25 mL THF for quantitative transfer) into the flask containing NaO*t*-Bu. During this time, a precipitate began to form. The reaction was allowed to stir overnight, gradually warming to room temperature.

The next day, the reaction was diluted with Et_2O (250 mL) and filtered through a pad of celite to remove salts. The filtrate was concentrated thoroughly *in vacuo*, giving a viscous orange oil. This crude product was purified by column chromatography (1 Liter silica gel, 10 cm diameter, isocratic 30:10:2 Hex/DCM/Et_2O, R_f = 0.25), affording **SI-16** as a clear colorless oil (7.68 g, 23.1 mmol, 72% yield).

^1H NMR (500 MHz, $CDCl_3$) δ 7.36-7.31 (m, 4H), 7.27 (m, 1H), 4.49 (s, 2H), 3.46 (t, J = 6.7 Hz, 2H), 1.65-1.58 (m, 2H), 1.45 (m, 1H), 1.39-1.25 (m, 5H), 1.23 (s, 12H), 1.03-0.96 (m, 1H), 0.95 (d, J = 5.7 Hz, 3H).

^{13}C NMR (126 MHz, $CDCl_3$) δ 138.88, 128.47, 127.75, 127.57, 82.91, 73.00, 70.72, 33.30, 29.91, 28.96, 26.54, 24.90, 24.87, 15.65.

^{11}B NMR (128 Hz, $CDCl_3$) δ 34.37.

HRMS (EI^+) Calculated for $C_{20}H_{33}O_3B$ $(M)^+$: 332.25229, Found: 332.25227

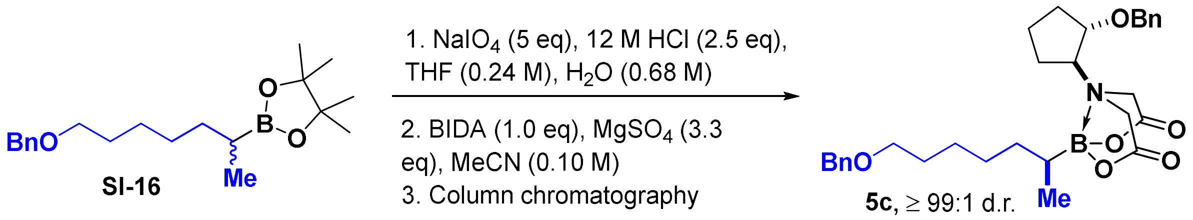

BIDA Boronate 5c. The pinacol boronic ester **SI-16** (6.68 g, 20.1 mmol, 1.00 eq) was added to a stir bar-equipped 1 Liter round bottom flask. THF (84 mL, 0.24 M), H_2O (30 mL, 0.68 M), and sodium periodate (21.5 g, 100.5 mmol, 5.00 eq) were added. To the resulting stirring mixture was added concentrated HCl (12 Molar, 4.2 mL, 50.4 mmol, 2.5 eq). The reaction was allowed to stir at room temperature for two hours. Monitoring the reaction by TLC showed the formation of boronic acid (with 1:1 Hex/EtOAc) and consumption of starting material (with 5/1 Hex/EtOAc).

Solvent was removed by rotary evaporation. The aqueous mixture was diluted with addition water and extracted with methyl *tert*-butyl ether three times. Combined organic layers were washed with water six times to remove any traces of the oxidant before finally drying with Na_2SO_4 and performing a solvet switch to dry acetonitrile (200 mL, 0.10 Molar). To this solution was added a stir bar, magnesium sulfate (8.04 g, 66.8 mmol, 3.3 eq), and BIDA (6.18 g, 20.2 mmol, 1.00 eq). The reaction was sealed with a rubber septum and vac-filled with nitrogen (using brief cycles to avoid solvent evaporation). The reaction was stirred overnight at 60°C.

The next day, the reaction was filtered through a pad of silica gel, rinsing with EtOAc. The crude product was resolved by normal phase column chromatography (1/1.3 Hex/EtOAc), giving a fraction of mostly the first diastereomer and another fraction of mostly the second diastereomer. The first diastereomer was repurified with two more columns (1/1.2 Hex/EtOAc and 1/1.1 Hex/EtOAc, R_f = 0.30), giving the pure product as a sticky foam (3.29 g, 6.31 mmol, 31% yield). ^{1}H-NMR in $CDCl_3$ showed a diastereomeric ratio of ≥99:1 by integrating the methyl signals of **5c** and ***epi*-5c** at 0.86 and 0.98 ppm, respectively. The absolute stereochemistry at the boron-bearing carbon was tentatively assigned based on analogy to other BIDA boronates.

^{1}H NMR (500 MHz, $CDCl_3$) δ 7.43-7.25 (m, 10H), 4.65 (d, *J* = 11.5 Hz, 1H), 4.49 (s, 2H), 4.38 (d, *J* = 11.5 Hz, 1H), 4.00 (d, *J* = 16.7 Hz, 1H), 3.90 (q, *J* = 6.7 Hz, 1H), 3.62 (m, 1H), 3.61 (d, *J* = 16.8 Hz, 1H), 3.46 (t, *J* = 6.6 Hz, 2H), 3.43 (d, *J* = 16.4 Hz, 1H), 3.32 (d, *J* = 16.9 Hz, 1H), 2.21 (m, 1H), 2.06 (m, 1H), 1.88-1.68 (m, 3H), 1.65-1.57 (m, 3H), 1.53-1.43 (m, 2H), 1.40-1.19 (m, 4H), 0.86 (d, *J* = 6.1 Hz, 3H), 0.83 (m, 1H).

^{13}C NMR (126 MHz, $CDCl_3$) δ 168.93, 167.39, 138.84, 136.38, 128.97, 128.74, 128.45, 128.43, 127.74, 127.54, 79.12, 72.90, 72.05, 70.65, 61.03, 56.14, 32.23, 29.85, 29.58, 27.86, 26.57, 26.52, 21.41, 14.72,

^{11}B NMR (128 MHz, $CDCl_3$) δ 13.67.

$[\alpha]^{20}_D$ = +31.9 (c = 1.0, acetone)

HRMS (ESI+) Calculated for $C_{30}H_{41}BNO_6$ $(M+H)^+$: 522.3027, Found: 522.3028

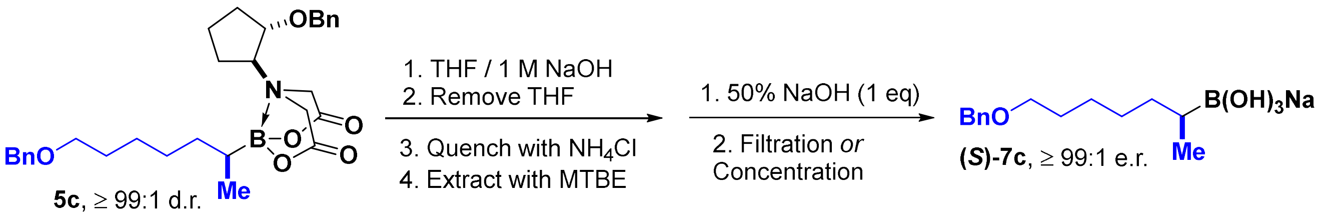

Sodium alkyltrihydroxyborate (*S*)-7c was made from BIDA boronate **5c** according to general procedure B. The product was isolated by concentration *in vacuo* (1.64 g, 5.65 mmol, 90% yield).

^{1}H NMR (500 MHz, CD_3OD) δ 7.32 (d, *J* = 4.3 Hz, 4H), 7.26 (ddd, *J* = 8.8, 4.9, 3.8 Hz, 1H), 4.48 (s, 2H), 3.48 (t, *J* = 6.7 Hz, 2H), 1.65-1.57 (m, 2H), 1.55-1.26 (m, 4H), 1.17 (m, 1H), 1.04 (m, 1H), 0.83 (d, *J* = 7.1 Hz, 3H), 0.58 (b. s, 1H).

^{13}C NMR (126 MHz, CD_3OD) δ 139.86, 129.33, 128.84, 128.59, 73.80, 71.74, 34.87, 31.02, 30.82, 27.97,

16.41.

^{11}B NMR (128 MHz, CD_3OD) δ 6.83.

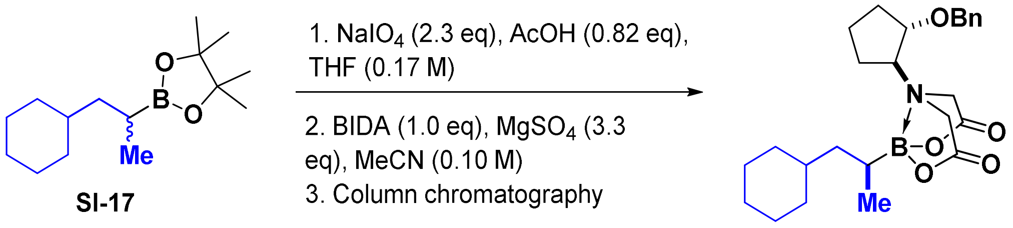

SI-17

5d, ≥99:1 d.r.

BIDA Boronate 5d. AcOH (11.7 mL, 12.3 g, 204 mmol, 0.82 eq.) was added as a single portion to a slurry of $NaIO_4$ (125 g, 584 mmol, 2.3 eq.) and pinacol boronic ester **SI-17**[19] (62.8 g, 249 mmol, 1.00 eq.) in THF (1.5 L, 0.17 Molar). The reaction mixture was stirred at room temperature, filtered to remove salts and the filter cake washed with Et_2O (200 mL). The filtrate was concentrated *in vacuo* and then partitioned between H_2O (500 mL) and methyl *tert*-butyl ether (1 L). The organic layer was separated, and then the aqueous phase was extracted with a MTBE (1 L). The combined organics were washed with water (5x300 mL) until free of peroxide (as determined by peroxide test strips). The organics were diluted with DMSO (196 mL, 1.3 Molar) and concentrated *in vacuo* to afford a DMSO solution of boronic acid. The DMSO solution was diluted with toluene (2.1 L, 0.12 Molar) and BIDA (50 g, 163 mmol, 0.65 eq.) was added. The reaction mixture was then heated at reflux with a Dean-Stark Trap for 3 hours. The reaction mixture was concentrated *in vacuo* to afford 90 g of crude material which was purified with the following gradient elution of hexanes/EtOAc (80:20 3L, 70:30 2L, 60:40 1L, 55:45 1L, 50:50 1L, 45:55 1L, 30:70 1L), affording the mostly resolved BIDA boronate (12.5 g, 28.3 mmol 17% yield, ~97:3 dr) after 5 columns. This material was then suspended in boiling hexanes (3 L), and a minimal amount of EtOAc (~90 mL) was added to effect dissolution. The mixture was cooled to room temperature, cooled in an ice bath for one hour, and then filtered to give the BIDA boronate. After a second crop was recrystallized, the product was isolated as a white powder (8.66 g, 20.1 mmol, 12% yield). ^{1}H-NMR in DMSO-*d6* showed a diastereomeric ratio of ≥99:1 by integrating the methyl signals of **5d** and ***epi*-5d** at 0.72 and 0.80 ppm, respectively.

^{1}H NMR (500 MHz, DMSO-*d6*, 40°C) δ 7.40 – 7.26 (m, 5H), 4.53 (d, *J* = 11.3 Hz, 1H), 4.46 (d, *J* = 11.3 Hz, 1H), 4.18 – 4.09 (m, 3H), 4.06 (d, *J* = 17.7 Hz, 1H), 3.93 (d, *J* = 17.0 Hz, 1H), 3.56 (td, *J* = 8.7, 6.1 Hz, 1H), 2.13 – 1.95 (m, 2H), 1.76 – 1.52 (m, 8H), 1.50 – 1.39 (m, 1H), 1.36 – 0.84 (m, 8H), 0.74 (d, *J* = 6.7 Hz, 3H), 0.65 (q, *J* = 12.5, 11.8 Hz, 1H).

^{13}C NMR (126 MHz, $CDCl_3$) δ 169.24, 167.66, 136.67, 128.85, 128.52, 128.32, 79.50, 72.21, 72.07, 60.85, 56.29, 39.55, 34.94, 34.52, 31.97, 29.76, 26.91, 26.68, 26.66, 26.42, 21.52, 14.59.

^{11}B NMR (128 MHz, $CDCl_3$) δ 14.48.

$[\alpha]^{20}_D$ = +2.25 (c 1.0, acetone)

HRMS (ESI+) Calculated for $C_{25}H_{37}BNO_5$ $(M+H)^+$: 442.2765, Found: 442.2760

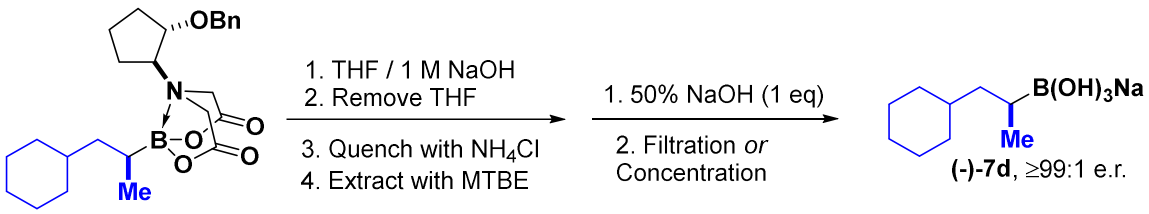

5d, ≥99:1 d.r.

(-)-7d, ≥99:1 e.r.

Sodium alkyltrihydroxyborate (-)-7d was made from BIDA boronate **5d** according to general procedure B. The product was isolated by concentration *in vacuo* (3.9 g, 18.5 mmol, 92% yield, white solid).

^{1}H NMR (400 MHz, D_2O) δ 1.71 (d, J = 13.4 Hz, 1H), 1.65 – 1.45 (m, 4H), 1.29 – 0.99 (m, 5H), 0.92 – 0.75 (m, 2H), 0.70-0.56 (m, 4H), 0.38 (b. s, 1H).

^{13}C NMR (101 MHz, D_2O) δ 42.20, 36.28, 36.01, 32.93, 27.58, 27.26, 27.06, 16.22.

^{11}B NMR (128 MHz, D_2O) δ 8.71.

$[\alpha]^{20}_{D}$ = -16.9 (c 0.7, H_2O)

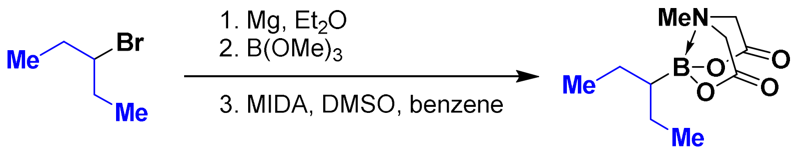

6e

MIDA Boronate 6e. 3-bromopentane (4.53 g, 30 mmol) was added dropwise to Mg turnings (840 mg, 35 mmol) in Et_2O (30 mL). After 1 h the reaction mixture was cooled to -78 C and $B(OMe)_3$ (3.87L, 35 mmol) was added dropwise, the reaction mixture was warmed to room temperature and stirred fro 30 mins at which point 300 mL 1M HCl was added. The organic layer was separated, and the aqueous layer extracted with Et_2O (2 x 100 mL), the organics were combined, dried (Na_2SO_4) and decanted into a 500 mL RBF. DMSO (40 mL) was added to the flask and the Et_2O was removed in vacuo. MIDA (1.47 g, 10 mmol) and benzene (95 mL) were added and the reaction mixture heated under dean-stark conditions for 3h. The reaction mixture was diluted with EtOAc (200 mL) and washed with water (4 x 50 mL), dried over $MgSO_4$, filtered and concentrated to dryness. The residue was dissolved in the minimum amount of acetone and Et2O/Hexanes (1:1, 300 mL) was added causing precipitation of **6e** which was collected by vacuum filtration on a fine porosity fritted glass funnel as a white crystalline solid (530 mg, 2.33 mmol, 23% yield).

^{1}H NMR (500 MHz, CD_3CN) δ 3.90 (d, J = 17.0 Hz, 2H), 3.77 (d, J = 17.0 Hz, 2H), 2.88 (s, 3H), 1.45 – 1.29 (m, 4H), 0.90 (t, J = 7.4 Hz, 6H), 0.67-0.65 (m, 1H).

^{13}C NMR (126 MHz, CD_3CN) δ 169.23, 63.31, 46.56, 21.71, 12.88.

^{11}B NMR (128 MHz, CD_3CN) δ 13.18.

HRMS (ESI^+) Calculated for $C_{10}H_{19}O_2BN$ $(M)^+$: 228.1407, Found: 228.1399

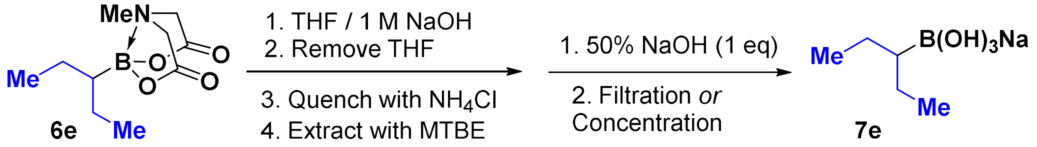

Sodium alkyltrihydroxyborate 7e was made from BIDA boronate **5e** according to general procedure B. The product was isolated by concentration in vacuo (0.23 g, 1.50 mmol, 85% yield, white solid).

^{1}H NMR (500 MHz, DMSO-*d6*) δ 1.35 – 1.27 (m, 2H), 1.15 (bs, 2H), 0.78 (t, J = 7.2 Hz, 6H), 0.36 (bs, 1H).

^{13}C NMR (126 MHz, DMSO-*d6*) δ 24.87, 14.30.

^{11}B NMR (128 MHz, DMSO-*d6*) δ 3.92.

The BIDA boronates corresponding to boronic acid **1g** were not readily separable. Boronic acid **1g** was prepared in non-racemic form through the following process.

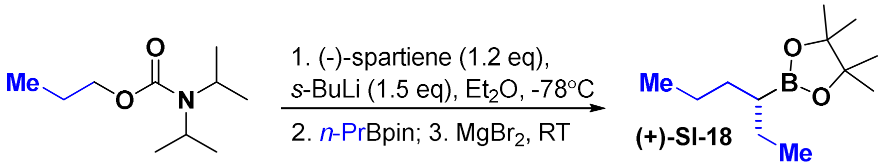

Pinacol boronic ester (+)-SI-18. According to the procedure of Aggarwal and co-workers,[19] *n*-propyl carbamate[19] (3.29 g, 17.6 mmol, 1.2 eq.) and (-)-spartiene (4.20 mL, 17.6 mmol, 1.2 eq.) were dissolved in Et_2O (86 mL) and *s*-BuLi (13.5 mL, 1.6M in cyclohexane, 21.6 mmol, 1.5 eq) was added dropwise at −78 °C. After five hours, *n*-PrBpin (2.50 g, 14.7 mmol, 1.0 eq.) was added dropwise. The reaction mixture was stirred for one more hour at −78 °C before warming to room temperature, and then a biphasic solution of $MgBr_2$•Et_2O was added as a single portion. [$MgBr_2$•Et_2O was prepared in a separate flask from 1,2-dibromoethane (1.91 mL, 22 mmol, 1.5 eq.) and Mg turnings (528 mg, 22 mmol, 1.5 eq.) in Et_2O (22 mL).] The reaction mixture was heated at reflux overnight, cooled to room temperature, and quenched with water (200 mL). The organic layer was separated, and the aqueous phase extracted with Et_2O (3 x 200 mL). The organics were combined, dried over $MgSO_4$, filtered and concentrated *in vacuo*. Subsequent purification by column chromatography (1% Et_2O/hexane) gave **SI-18** as a colourless oil (1.39 g, 6.55 mmol, 45% yield).

^{1}H NMR (500 MHz, $CDCl_3$) δ 1.46-1.35 (m, 3H), 1.34-1.26 (m, 3H), 1.24 (s, 12H), 0.94-0.85 (m, 7H)

^{13}C NMR (126 MHz, $CDCl_3$) δ 82.91, 33.60, 24.97, 24.95, 24.40, 22.51, 14.59, 13.86.

^{11}B NMR (128 MHz, $CDCl_3$) δ 34.40

$[\alpha]^{20}_D$ = +0.9 (c 1.69, $CHCl_3$)

HRMS (CI^+) Calculated for $C_{12}H_{25}O_2B$ $(M)^+$: 212.1948, Found: 212.1949

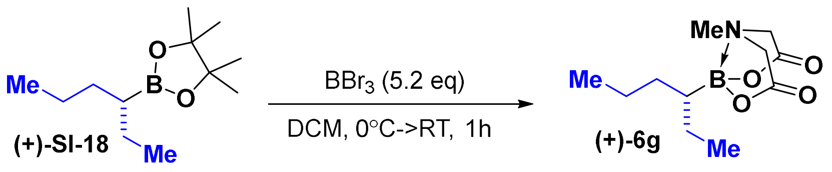

MIDA boronate (+)-6g. A flame dried, stir bar-equipped 3-neck 500mL RBF was sealed with septa, fitted with a Schlenk adaptor, and put under nitrogen via three vac-fill cycles. Separately, pinacol boronic ester **(+)-SI-18** (0.849 g, 4.00 mmol, 1.00 eq) was massed out into a dry 40 mL vial and likewise put under nitrogen. **(+)-SI-18** was transferred to the RBF using DCM, and then additional DCM was added to reach 51 mL. The stirring solution was cooled to 0°C with an ice/water bath, and then BBr_3 (1.0 M in DCM, 20.8 mL, 20.8 mmol, 5.2 eq) was added (dropwise over 10 minutes). The reaction was stirred for another 10 minutes a 0°C and then allowed to warm to room temperature and stirred for an additional hour.

At this point, TLC confirmed that the starting material was consumed (4/1 Hex/DCM) and that boronic acid was present (1/1 Hex/EtOAc). The reaction was cooled to 0°C and quenched with H_2O (120 mL). Additional H_2O (80 mL) and methyl tert-butyl ether (200 mL) were added, and the layers were mixed and separated. The organic layer was washed with 0.1 Molar HCl (200 mL) and then H_2O (200 mL). The organic layer was then dried with Na_2SO_4, decanted, and partially concentrated to a volume of 20 mL. Toluene (21 mL) and DMSO (2 mL) were added, and then the remaining MTBE and DCM were removed by rotary evaporation. The resulting solution (in a 100 mL recovery flask) was charged with a stir bar and MIDA. The flask was then equipped with a Dean Stark trap and reflux condenser. After refluxing for 1.5 hours, TLC confirmed that the boronic acid was gone (1/1 Hex/EtOAc) and that MIDA boronate was present (100% EtOAc). The stir bar was removed, and then toluene was removed by rotary evaporation using the pump cart.

The resulting DMSO solution was diluted with H2O (60 mL) and extracted with EtOAc (3x60mL EtOAc). Combined organics were repeatedly washed with H_2O (5x180 mL), washed with brine (1x180 mL), dried with Na_2SO_4, decanted and concentrated in vacuo. The resulting yellow solid was purified by column chromatography (5 cm diameter, 250 mL SiO2, isocratic 1/1 Hex/acetone, Rf = 0.35), affording the pure product as a fluffy white powder (680 mg, 2.82 mmol, 71% yield).

^{1}H NMR (500 MHz, DMSO-*d6*) δ 4.15 (d, *J* = 17.2 Hz, 2H), 3.98 (d, *J* = 17.2 Hz, 2H), 2.87 (s, 3H), 1.42-1.30 (m, 2H), 1.29-1.14 (m, 4H), 0.88-0.82 (m, 6H), 0.67 (m, 1H).

^{13}C NMR (126 MHz, DMSO-*d6*) δ 169.07, 169.03, 62.23, 62.21, 45.53, 30.31, 21.12, 20.77, 14.54, 12.49.

^{11}B NMR (128 MHz, acetone-*d6*) δ 18.39.

$[\alpha]^{20}_D$ = +6.1 (c 1.05, acetone)

HRMS (CI$^+$) Calculated for $C_{11}H_{21}O_4NB$ $(M+H)^+$: 242.15637, Found: 242.15626

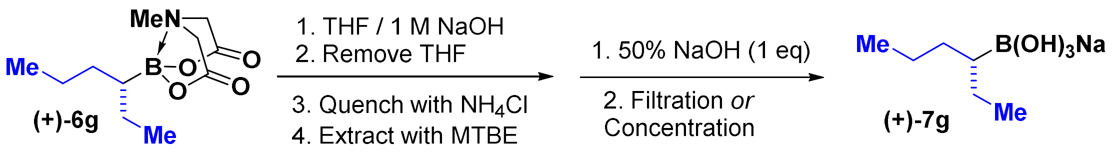

Sodium alkyltrihydroxyborate (+)-7g was made from MIDA boronate **(+)-6g** according to general procedure B. The product was isolated by concentration *in vacuo* (311 mg, 1.828 mmol, 91% yield).

^{1}H NMR (500 MHz, CD_3OD) δ 1.52-1.31 (m, 3H), 1.31-1.16 (m, 6H), 0.92-0.84 (m, 6H), 0.84-0.59 (b. s, 1H).

^{13}C NMR (126 MHz, CD_3OD) δ 35.99, 30.77, 26.04, 24.79, 15.46.

^{11}B NMR (128 MHz, CD_3OD) δ 6.70.

$[\alpha]^{20}_{D}$ = +4.4 (c 1.05, methanol)

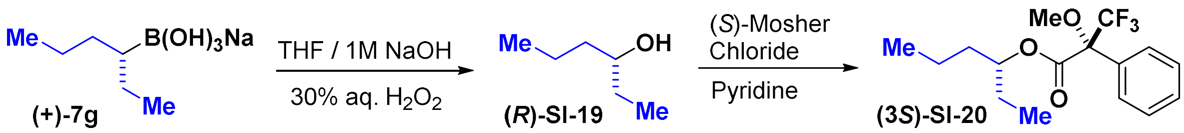

SI-20. To a stir bar-equipped 7 mL vial were added trihydroxyborate **(+)-7g** (4.2 mg, 0.025 mmol, 1.0 eq), THF (0.25 mL, 0.10 Molar), 1M NaOH (0.25 mL, 0.25 mmol, 10 eq), and 30% aqueous H_2O_2 (0.025 mL). The mixture was stirred at room temperature. After one hour, TLC (2:1 Hex/EtOAc, $KMnO_4$) indicated full conversion of the boronic acid. The reaction was quenched by treatment with saturated aqueous $Na_2S_2O_3$ (0.5 mL) and then extracted with Et_2O (3x0.5 mL Et_2O). The combined organic layers were passed through a short (0.5 cm) plug of silica gel in a glass pipet, rinsing with Et_2O. The filtrate was collected in a flame-dried 7 mL vial and then subjected to rotary evaporation using mild vacuum, giving crude **(*R*)-SI-19**.

To the vial containing **(*R*)-SI-19** were added a dry stir bar, anhydrous pyridine (0.10 mL, 0.098 g, 1.24 mmol, 50 eq), and (*S*) -(+)-α-Methoxy-α-(trifluoromethyl)phenylacetyl chloride (6.1 µL, 8.2 mg, 0.035 mmol, 1.4 eq). The reaction was capped and stirred at room temperature overnight. The next day, the reaction was quenched with 1M HCl (2 mL), extracted with Et_2O (3x1.5 mL Et2O), dried with Na_2SO_4, and concentrated. The crude product **(3*S*)-SI-20** was collected by passing through a plug of silica gel, rinsing with Et_2O.

This crude ester **(3*S*)-SI-20** was determined to be 97.2:2.8 d.r. by chiral HPLC (OD-H chiral column, isocratic 100% hexanes, 1.0 mL/min, 215.4 nm absorbance). Major = 9.7 minutes, minor = 10.4 minutes. A standard of **SI-20** in 1:1 d.r. was prepared from 3-hexanol, following the same procedure as for **(3*S*)-SI-20**.

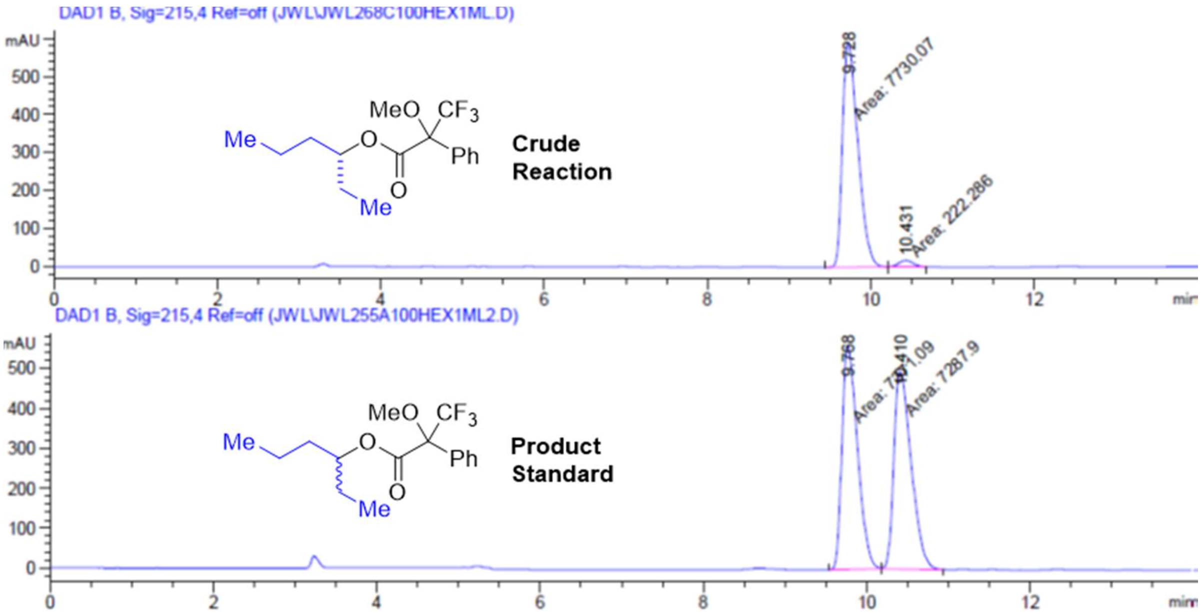

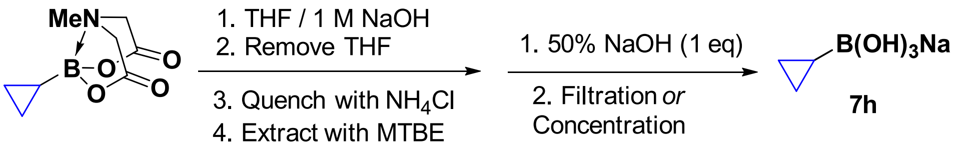

Sodium alkyltrihydroxyborate 7h was made from the commercially available MIDA boronate by general procedure B in 84% yield as a white solid.

^{1}H NMR (400 MHz, D_2O) δ 0.44 – 0.40 (m, 2H), 0.18 (s, 2H), -0.31 – -0.35 (m, 1H).

^{13}C NMR (126 MHz, D_2O) δ 0.76.

^{11}B NMR (128 MHz, D_2O) δ 5.63.

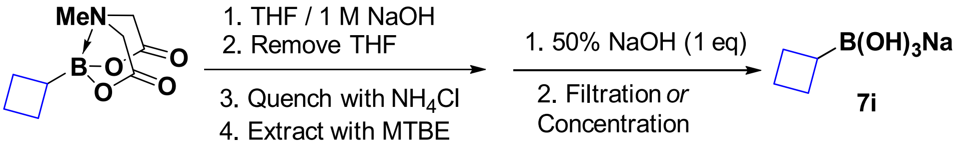

Sodium alkyltrihydroxyborate 7i was made from the commercially available MIDA boronate by general procedure B in 80% yield as a white solid.

^1H NMR (400 MHz, D_2O) δ 1.22 – 2.14 (m, 1H), 2.10 – 1.94 (m, 5H), 1.77 – 1.71 (m, 1H).

^{13}C NMR (126 MHz, D_2O) δ 24.45, 21.72.

^{11}B NMR (128 MHz, D_2O) δ 7.02.

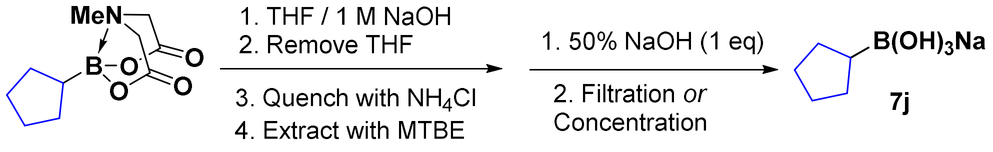

Sodium alkyltrihydroxyborate 7j was made from the commercially available MIDA boronate by general procedure B in 86% yield as a white solid.

^1H NMR (400 MHz, D_2O) δ 1.85 – 1.62 (m, 6H), 1.36 (td, J = 11.8, 6.0 Hz, 2H), 0.91 – 0.82 (m, 1H).

^{13}C NMR (126 MHz, D_2O) δ 29.70, 27.44.

^{11}B NMR (128 MHz, D_2O) δ 7.03.

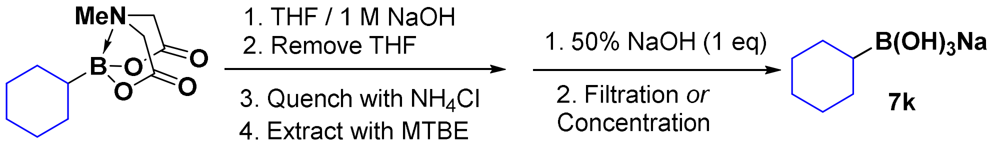

Sodium alkyltrihydroxyborate 7k was made from the commercially available MIDA boronate by general procedure B in 96% yield as a white solid.

^1H NMR (400 MHz, D_2O) δ 1.88 – 1.81 (m, 5H), 1.38 (q, J = 3.7 Hz, 3H), 1.17 (q, J = 11.9, 11.5 Hz, 2H), 0.43 (bs, 1H).

^{13}C NMR (126 MHz, D_2O) δ 29.81, 28.87, 27.75.

^{11}B NMR (128 MHz, D_2O) δ 7.10.

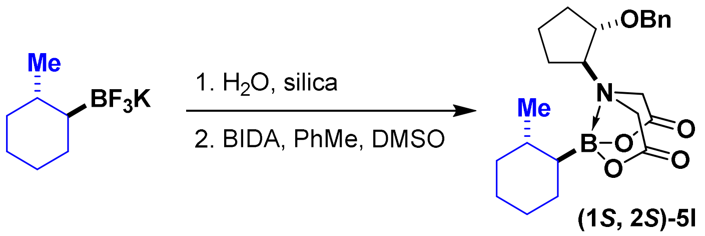

BIDA boronate (1*S*, 2*S*)-5l. 1.07 g (5 mmol) of racemic potassium *trans*-2-methyl-cyclohexyltrifluoroborate (95% purity, Frontier Scientific) was combined with 25 mL of H_2O and 1.07 g of silica gel. The suspension was stirred at 23 °C for 20 min. 10mL of MTBE was added and the mixture was

filtered through a pad of Celite with a washing of MTBE to remove the silica. The aqueous layer was extracted with MTBE and the combined organic layers were dried over sodium sulfate and concentrated under vacuum to 5 mL. This crude solution of boronic acid was used directly in the next step, forming **7l** of ~1:1 d.r. by general procedure A. After work up, the crude material was subjected to resolution by silica column using a gradient of 10% to 70% EtOAc in hexanes. Fractions were analyzed by TLC using 1:1 EtOAc:hexanes and $KMnO_4$ stain. 620 mg (1.5 mmol) of the top diastereomer was isolated as a white foam in 30% yield. ^{1}H-NMR integration of the signals at 0.71 and 0.45 ppm showed none of the bottom diastereomer. The absolute configuration of the C1 and C2 stereocenters was assigned by x-ray crystal structure of the corresponding MIDA boronate.

^{1}H NMR (500 MHz, $CDCl_3$) δ 7.45 – 7.34 (m, 3H), 7.30 (d, *J* = 6.3 Hz, 2H), 4.65 (d, *J* = 11.6 Hz, 1H), 4.38 (d, *J* = 11.5 Hz, 1H), 4.00 (d, *J* = 16.7 Hz, 1H), 3.92 (q, *J* = 6.8 Hz, 1H), 3.69 (q, *J* = 8.5 Hz, 1H), 3.57 (d, *J* = 17.1 Hz, 1H), 3.43 (d, *J* = 16.6 Hz, 1H), 3.24 (d, *J* = 17.0 Hz, 1H), 2.24 (tt, *J* = 12.1, 6.0 Hz, 1H), 2.06 (dt, *J* = 15.7, 5.9 Hz, 1H), 1.92 – 1.59 (m, 7H), 1.58 – 1.42 (m, 3H), 1.35 – 1.09 (m, 4H), 1.06 – 1.02 (m, 3H), 1.02 – 0.94 (m, 1H), 0.49 – 0.39 (m, 1H).

^{13}C NMR (126 MHz, $CDCl_3$) δ 169.19, 167.54, 136.40, 129.01, 128.80, 128.52, 79.07, 72.15, 71.57, 61.27, 55.68, 37.74, 33.33, 29.49, 28.28, 26.89, 26.53, 26.29, 23.20, 21.30.

^{11}B NMR (128 MHz, $CDCl_3$) δ 14.0.

$[\alpha]^{20}_D$ = +21.2 (c 1, acetone)

HRMS (ESI$^+$) Calculated for $C_{23}H_{33}BNO_5$: 414.2452 Found: 414.2450

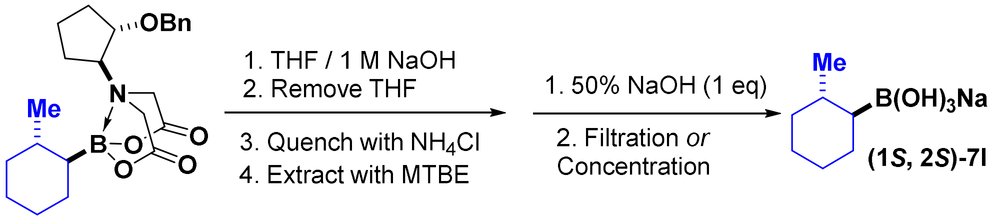

Sodium alkyltrihydroxyborate 7l was made from BIDA boronate **(1*S*, 2*S*)-5l** by general procedure B in 71% yield as a white solid.

^{1}H NMR (400 MHz, D_2O) δ 1.92 – 1.85 (m, 3H), 1.53 – 1.45 (m, 2H), 1.39 – 1.31 (m, 2H), 1.07 (d, *J* = 6.6 Hz, 3H), 0.56 (bs, 1H).

^{13}C NMR (126 MHz, D_2O) δ 37.18, 34.42, 29.64, 27.79, 27.32, 23.31.

^{11}B NMR (128 MHz, D_2O) δ 7.10.

$[\alpha]^{20}_D$ = +12.4 (c. 1, MeOH)

Synthesis and Characterization of Cross-Coupled Products

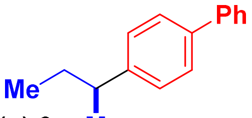

(+)-3a

Boronic acid ***(S)*-1a** (≥99:1 e.r.) was prepared in 77% yield by general procedure C and coupled to organohalide **2a** to give product **(+)-3a** by general procedure D. The branched/linear product ratio of the crude reaction was determined to be 268/1 by HPLC (OD-H chiral column, isocratic 100% hexanes, 2.0 mL/min, 214.4 nm absorbance). Branched = 6.0 and 10.3 minutes; linear = 19.9 minutes. The product was isolated in 81% yield (17.0 mg) by purification with reverse phase flash chromatography (6/1 $MeCN/H_2O$). The e.r. of the purified product was determined to be 98.02 : 1.98. (98% es) using chiral HPLC (OD-H chiral column, isocratic 100% hexanes, 2.0 mL/min, 254.4 nm absorance). Major = 6.0 minutes; minor = 10.3 minutes.

A duplicate run of the reaction gave a branched/linear product ratio of 301/1, isolated yield of 83%, and enantiospecificity of 98%.

^{1}H NMR (500 MHz, $CDCl_3$) δ 7.60 (d, *J* = 7.3 Hz, 2H), 7.53 (d, *J* = 8.2 Hz, 2H), 7.43 (t, *J* = 7.6 Hz, 2H), 7.33 (t, *J* = 7.3 Hz, 1H), 7.26 (d, *J* = 8.1 Hz, 2H), 2.65 (sext, *J* = 7.0 Hz, 1H), 1.64 (m, 2H), 1.28 (d, *J* = 6.9 Hz, 3H), 0.87 (t, *J* = 7.4 Hz, 3H).

^{13}C NMR (126 MHz, $CDCl_3$) δ 146.95, 141.30, 138.81, 128.81, 127.60, 127.12, 127.11, 127.06, 41.48, 31.32, 21.98, 12.46.

$[\alpha]^{20}_D$ = +23.2 (c 1.0, $CHCl_3$)

HRMS (EI^+) Calculated for $C_{16}H_{18}$ $(M)^+$: 210.14085, Found: 210.14096

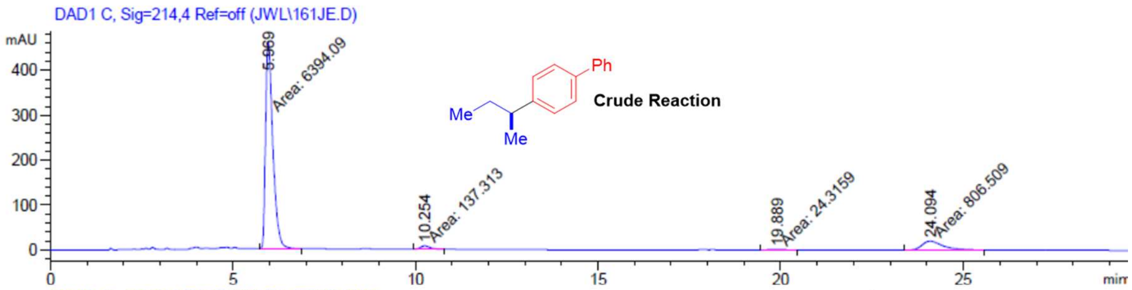
DAD1 C, Sig=214,4 Ref=off (JWL\161JE.D)
mAU
5.969
Area: 6394.09
10.254
Area: 137.313
19.889
Area: 24.3159
24.094
Area: 806.509
Crude Reaction
Me
Me
Ph
min

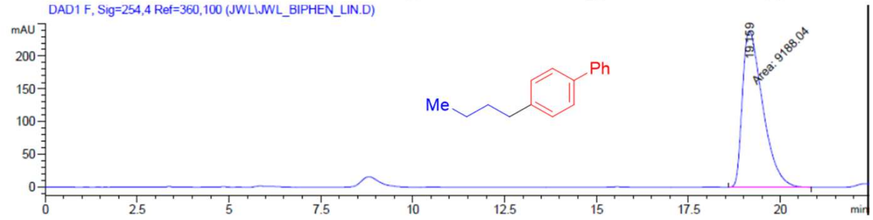
DAD1 F, Sig=254,4 Ref=360,100 (JWL\JWL_BIPHEN_LIN.D)
mAU
19.159
Area: 9188.04
Me
Ph
min

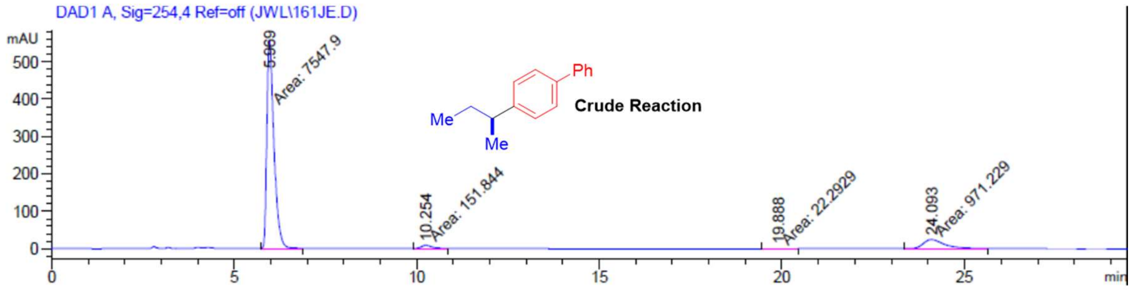
DAD1 A, Sig=254,4 Ref=off (JWL\161JE.D)
mAU
5.969
Area: 7547.9
10.254
Area: 151.844
19.888
Area: 22.2929
24.093
Area: 971.229
Crude Reaction
Me
Me
Ph
min

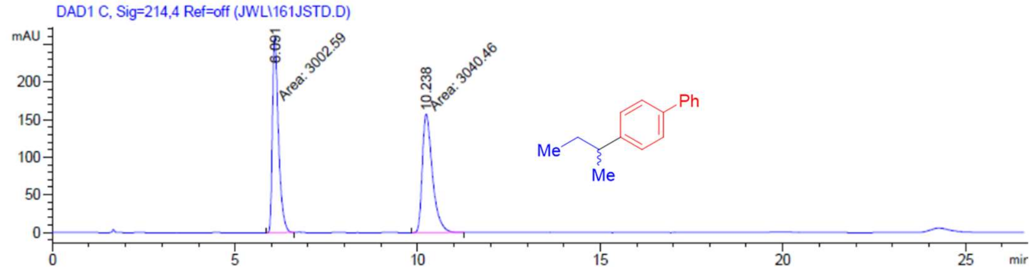
DAD1 C, Sig=214,4 Ref=off (JWL\161JSTD.D)
mAU
6.091
Area: 3002.59
10.238
Area: 3040.46
Me
Me
Ph
min

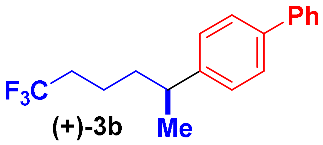

(+)-3b

Boronic acid **(*S*)-1b** (≥99:1 e.r.) was prepared in 66% yield by general procedure C and coupled to organohalide **2a** to give product **(+)-3b** by general procedure D. The branched/linear product ratio of the crude reaction was determined to be 243/1 by HPLC (Eclipse XDB-C8 column, isocratic 68/32 MeCN/H_2O, 1.2 mL/min, 254.4 nm absorbance). Branched = 11.2 minutes; linear = 12.0 minutes. The product was isolated in 59% yield (17.2 mg) by purification with reverse phase flash chromatography (5/1 MeCN/H_2O, Rf = 0.29). The e.r. of the purified product was determined to be 99.12 : 0.88. (>99% es) using chiral HPLC (AD-RH chiral column, isocratic 75/25 MeCN/H_2O, 0.5 mL/min, 254.4 nm absorance). Major = 8.9 minutes; minor = 14.6 minutes.

A duplicate run of the reaction gave a branched/linear product ratio of 283/1, isolated yield of 61%, and enantiospecificity of >99%.

^{1}H NMR (500 MHz, $CDCl_3$) δ 7.59 (dd, *J* = 8.2, 1.4 Hz, 2H), 7.54 (d, *J* = 8.1 Hz, 2H), 7.43 (t, *J* = 7.7 Hz, 2H), 7.33 (t, *J* = 7.5 Hz, 1H), 7.25 (d, *J* = 8.3 Hz, 2H), 2.75 (sext, *J* = 7.1 Hz, 1H), 2.06 (dddd, *J* = 19.0, 17.3, 9.3, 5.4 Hz, 2H), 1.67 (ddd, *J* = 9.7, 7.4, 3.6 Hz, 2H), 1.61 – 1.39 (m, 2H), 1.30 (d, *J* = 7.0 Hz, 3H).

^{13}C NMR (126 MHz, $CDCl_3$) δ 145.98, 141.15, 139.24, 128.86, 127.43, 127.35, 127.30 (q, *J* = 277 Hz), 127.14, 39.54, 37.47, 33.92 (q, *J* = 29 Hz), 22.34, 20.32 (q, *J* = 2.9 Hz).

^{19}F NMR (470 MHz, $CDCl_3$) δ -66.88 (t, *J* = 11.0 Hz).

$[\alpha]^{20}_{D}$ = +15.0 (c 1.0, $CHCl_3$)

HRMS (EI^+) Calculated for $C_{18}H_{19}F_3$ $(M)^+$: 292.14389, Found: 292.14326

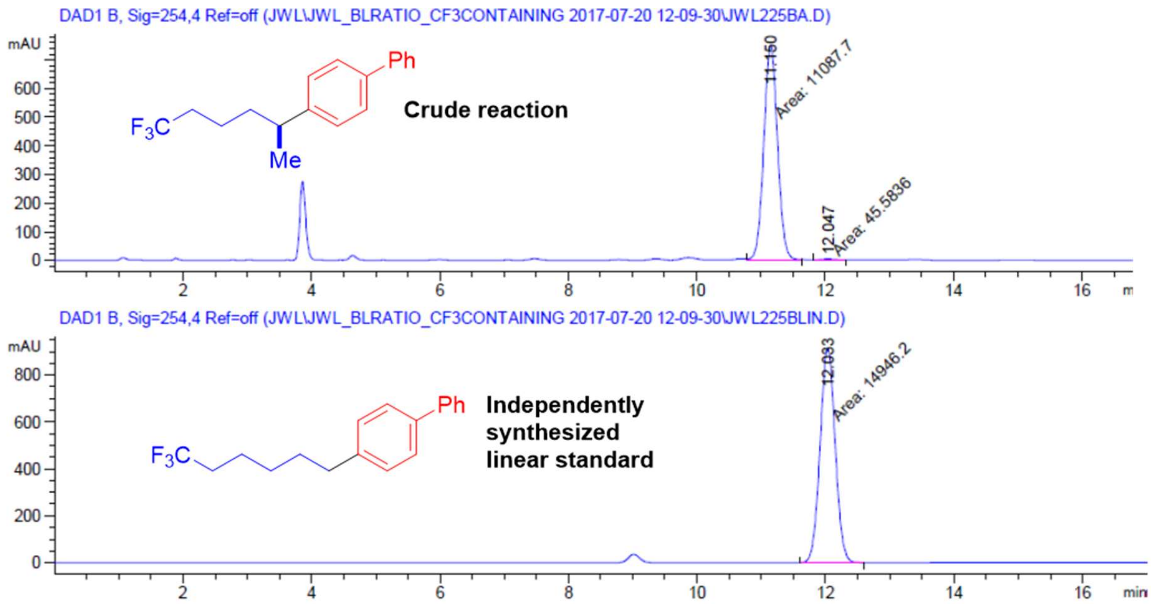

DAD1 B, Sig=254,4 Ref=off (JWL\JWL_BLRATIO_CF3CONTAINING 2017-07-20 12-09-30\JWL225BA.D)
Crude reaction
11.150
Area: 11087.7
12.047
Area: 45.5836
DAD1 B, Sig=254,4 Ref=off (JWL\JWL_BLRATIO_CF3CONTAINING 2017-07-20 12-09-30\JWL225BLIN.D)
Independently synthesized linear standard
12.033
Area: 14946.2

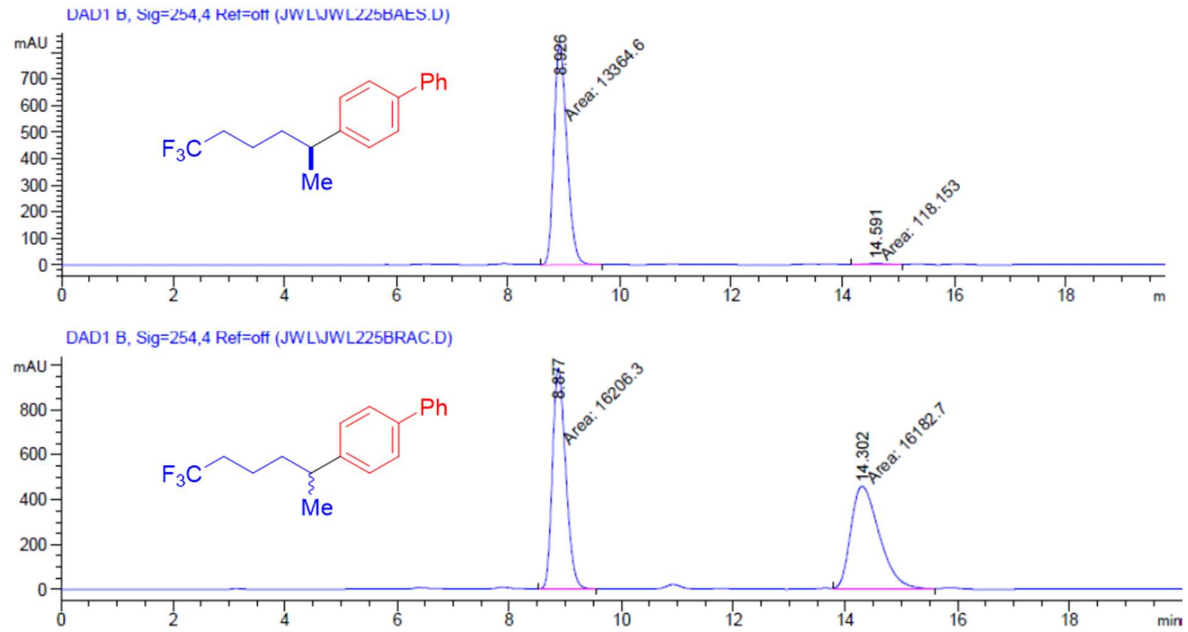

DAD1 B, Sig=254,4 Ref=off (JWL\JWL225BAES.D)
8.926
Area: 13364.6
14.591
Area: 118.153
DAD1 B, Sig=254,4 Ref=off (JWL\JWL225BRAC.D)
8.877
Area: 16206.3
14.302
Area: 16182.7

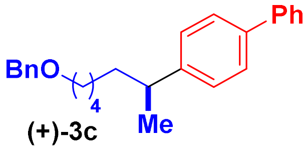

(+)-3c

Boronic acid ***(S)*-1c** (≥99:1 e.r.) was prepared in 71% yield by general procedure C and coupled to organohalide **2a** to give product **(+)-3c** by general procedure D. The branched/linear product ratio of the crude reaction was determined to be 702/1 by HPLC (Eclipse XDB-C8 column, isocratic 70/30 MeCN/H_2O, 1.2 mL/min, 254.4 nm absorbance). Branched = 24.8 minutes; linear = 26.8 minutes. The product was isolated in 69% yield (24.7 mg) by purification with normal phase flash chromatography (20/1 Hex/Et_2O, Rf = 0.27). The e.r. of the purified product was determined to be 99.05 : 0.95 (>99% es) using chiral HPLC (AD-RH chiral column, isocratic 94/6 MeCN/H_2O, 0.5 mL/min, 254.4 nm absorance). Major = 10.8 minutes; minor = 19.0 minutes.

A duplicate run of the reaction gave a branched/linear product ratio of 550/1, isolated yield of 72%, and enantiospecificity of >99%.

^{1}H NMR (500 MHz, $CDCl_3$) δ 7.61 (d, *J* = 7.7 Hz, 2H), 7.54 (d, *J* = 8.1 Hz, 2H), 7.45 (t, *J* = 7.7 Hz, 2H), 7.38-7.27 (m, 8H), 4.51 (s, 2H), 3.46 (t, *J* = 6.6 Hz, 2H), 2.74 (sext, *J* = 7.0 Hz, 1H), 1.69-1.58 (m, 4H), 1.43-1.19 (m, 4H), 1.28 (d, *J* = 6.9 Hz, 3H).

^{13}C NMR (126 MHz, $CDCl_3$) δ 147.12, 141.30, 138.84, 138.83, 128.81, 128.47, 127.73, 127.59, 127.52, 127.15, 127.13, 127.07, 72.98, 70.56, 39.69, 38.48, 29.82, 27.69, 26.42, 22.47.

$[\alpha]^{20}_D$ = +30.8 (c 1.0, $CHCl_3$)

HRMS (EI^+) Calculated for $C_{26}H_{30}O$ $(M)^+$: 358.2297, Found: 358.2299

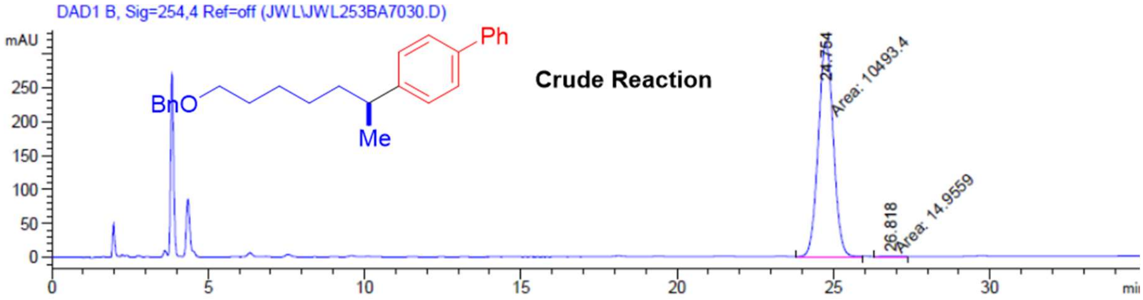
DAD1 B, Sig=254,4 Ref=off (JWL\JWL253BA7030.D)
Crude Reaction
BnO
Ph
Me
24.754
Area: 10493.4
26.818
Area: 14.9559

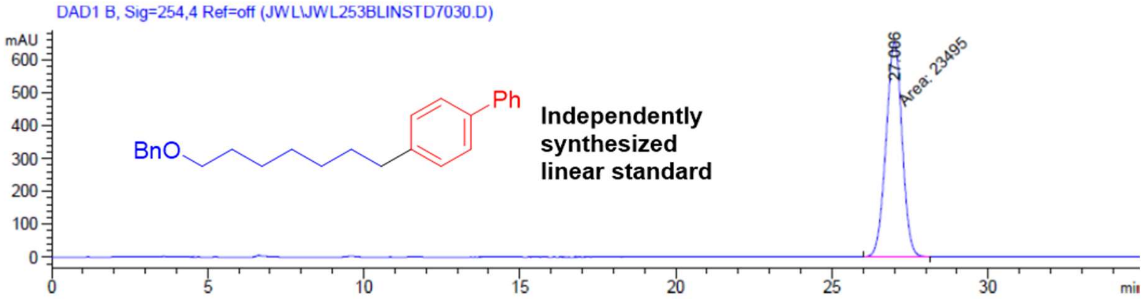
DAD1 B, Sig=254,4 Ref=off (JWL\JWL253BLINSTD7030.D)
Independently synthesized linear standard
BnO
Ph
27.006
Area: 23495

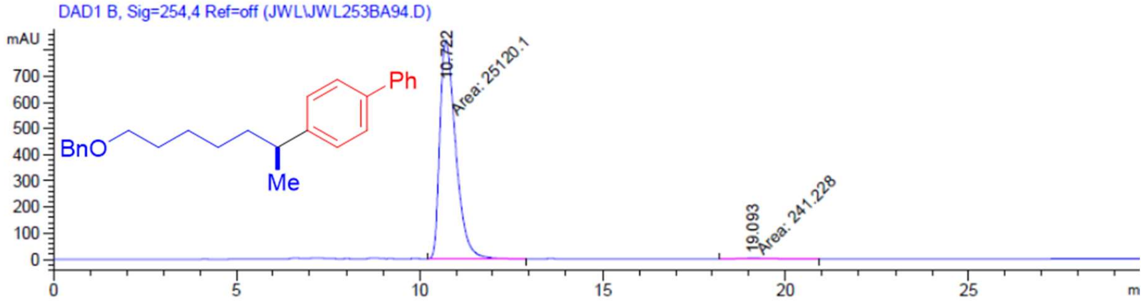
DAD1 B, Sig=254,4 Ref=off (JWL\JWL253BA94.D)
BnO
Ph
Me
10.722
Area: 25120.1
19.093
Area: 241.228

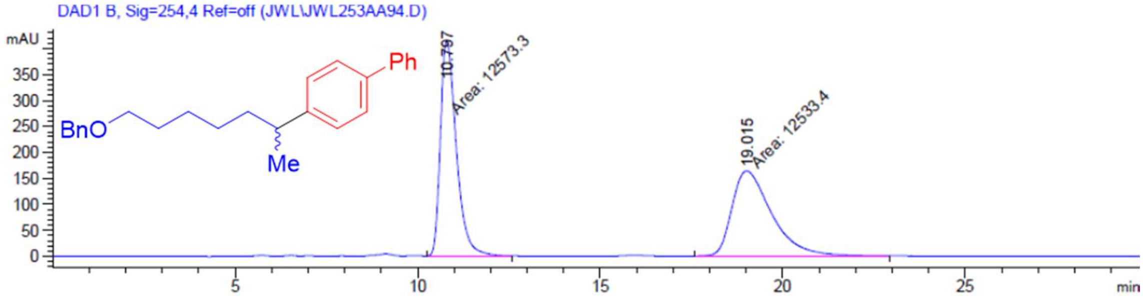
DAD1 B, Sig=254,4 Ref=off (JWL\JWL253AA94.D)
BnO
Ph
Me
10.797
Area: 12573.3
19.015
Area: 12533.4

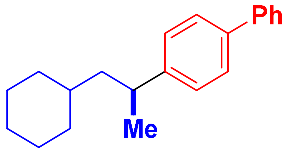

(+)-3d

Boronic acid **(*S*)-1d** (≥99:1 e.r.) was prepared in 67% yield by general procedure C and coupled to organohalide **2a** to give product **(+)-3d** by general procedure D. The branched/linear product ratio of the crude reaction was determined to be 276/1 by HPLC (Eclipse XDB-C8 column, isocratic 85/15 MeCN/H_2O, 1.2 mL/min, 254 nm absorbance). Branched = 9.3 minutes; linear = 10.2 minutes. The product was isolated in 74% yield (20.3 mg) by purification with reverse phase flash chromatography (20/1 MeCN/H_2O, R_f = 0.26). The e.r. of the purified product was determined to be 98.89 : 1.11 (>99% es) using chiral HPLC (OD-H chiral column, isocratic 100% hexanes, 2.0 mL/min, 254 nm absorance). Major = 6.2 minutes; minor = 10.2 minutes.

A duplicate run of the reaction gave a branched/linear product ratio of 279/1, isolated yield of 72%, and enantiospecificity of >99%.

^{1}H NMR (500 MHz, $CDCl_3$) δ 7.63 – 7.56 (m, 2H), 7.52 (d, *J* = 8.1 Hz, 2H), 7.42 (t, *J* = 7.7 Hz, 2H), 7.32 (t, *J* = 7.4 Hz, 1H), 7.28 – 7.23 (m, 2H), 2.87 (sext, *J* = 7.0 Hz, 1H), 1.78 (d, *J* = 12.9 Hz, 1H), 1.72 – 1.58 (m, 4H), 1.58 – 1.49 (m, 1H), 1.42 (td, *J* = 13.8, 7.2 Hz, 1H), 1.24 (d, *J* = 6.9 Hz, 3H), 1.22 – 1.09 (m, 4H), 0.89 (q, *J* = 11.1 Hz, 2H).

^{13}C NMR (126 MHz, $CDCl_3$) δ 147.50, 141.31, 138.73, 128.82, 127.51, 127.16, 127.12, 127.06, 46.51, 36.44, 35.22, 33.79, 33.55, 26.86, 26.42, 22.90.

$[\alpha]^{20}_D$ = +37.6 (c 1.0, $CHCl_3$)

HRMS (EI$^+$) Calculated for $C_{21}H_{26}(M)^+$: 278.20345, Found: 278.20395

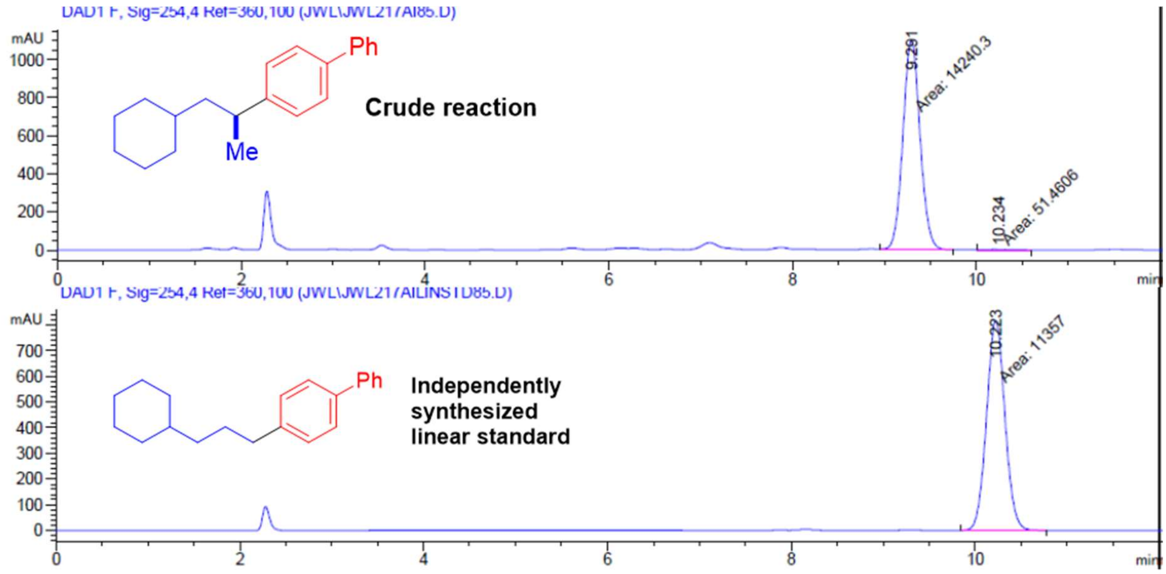
DAD1 F, Sig=254,4 Ref=360,100 (JWL\JWL217AI85.D)
mAU
Ph
Me
Crude reaction
9.291
Area: 14240.3
10.234
Area: 51.4606
min
DAD1 F, Sig=254,4 Ref=360,100 (JWL\JWL217AILINSTD85.D)
mAU
Ph
Independently
synthesized
linear standard
10.223
Area: 11357
min

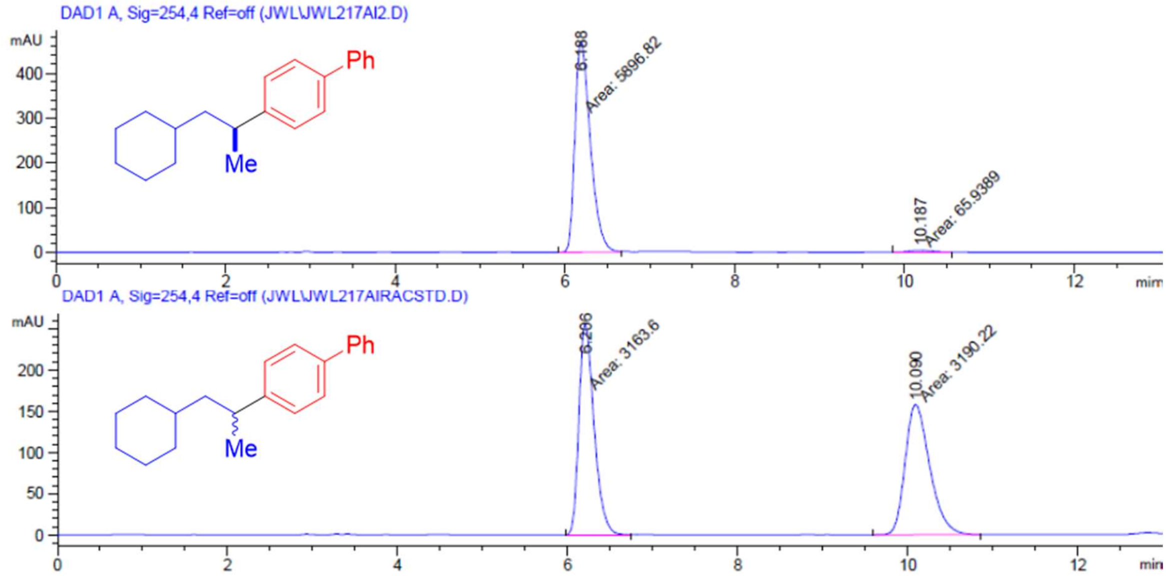
DAD1 A, Sig=254,4 Ref=off (JWL\JWL217AI2.D)
mAU
Ph
Me
6.188
Area: 5896.82
10.187
Area: 65.9389
min
DAD1 A, Sig=254,4 Ref=off (JWL\JWL217AIRACSTD.D)
mAU
Ph
Me
6.206
Area: 3163.6
10.090
Area: 3190.22
min

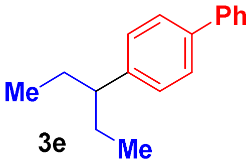

3e

Boronic acid **1e** was prepared by general procedure C and coupled to organohalide **2a** to give product **3e** by general procedure D. The product was isolated in 25% yield (11 mg) by purification with normal phase flash chromatography (hexanes) as a mixture with unreacted aryl halide.

A duplicate run of the reaction gave an isolated yield of 30%.

^{1}H NMR (500 MHz, $CDCl_3$) δ 7.55 (d, J = 8.2 Hz, 2H), 7.50-7.46 (Obscured 2H), 7.42-7.34 (3H, m), 7.24 (d, J = 8.1 Hz, 2H), 2.39 (td, J = 9.1, 4.7 Hz, 1H), 1.72 (m, 2H), 1.62 (m, 2H) 0.84 (t, J = 7.4 Hz, 6H).

^{13}C NMR (126 MHz, $CDCl_3$) 145.13, 141.31, 138.72, 128.81, 128.34, 127.78, 127.08, 127.03, 126.97, 49.48, 29.39, 12.41.

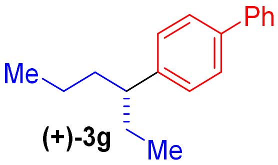

(+)-3g

Boronic acid **1g** (97.2:2.8 e.r.) was prepared in 76% yield by general procedure C and coupled to organohalide **2a** to give product **(+)-3g** by general procedure D. The regioisomeric product ratio of the crude reaction (3-hexyl : 2-hexyl : 1-hexyl) was determined to be 220/4.7/1 by GC (Cyclodex-B column, isothermal 170°C). 3-hexyl = 13.5 minutes; 2-hexyl = 15.0 minutes; 1-hexyl = 20.3 minutes). The product was isolated in 54% yield (12.7 mg) by purification with reverse phase flash chromatography (8/1 $MeCN/H_2O$, Rf = 0.26). The e.r. of the purified product was determined to be 96.31 : 3.69 (98.1% es) using chiral HPLC (OD-H chiral column, isocratic 100% hexanes, 1.0 mL/min, 254.4 nm absorance). Major = 7.4 minutes; minor = 8.0 minutes.

A duplicate run of the reaction gave a regioisomeric product ratio of 86/2.4/1, an isolated yield of 58%, and an enantiospecificity of 98.0%.

^{1}H NMR (500 MHz, $CDCl_3$) δ 7.60 (d, J = 7.6 Hz, 2H), 7.53 (d, J = 8.2 Hz, 2H), 7.43 (t, J = 7.7 Hz, 2H), 7.32 (t, J = 7.4 Hz, 1H), 7.21 (d, J = 8.1 Hz, 2H), 2.47 (m, 1H), 1.75-1.52 (m, 4H), 1.30-1.15 (m, 2H), 0.87 (t, J = 7.3 Hz, 3H), 0.81 (t, J = 7.4 Hz, 3H).

^{13}C NMR (126 MHz, $CDCl_3$) δ 145.40, 141.32, 138.70, 128.81, 128.28, 127.09, 127.03, 126.98, 47.41, 38.93, 29.82, 20.91, 14.35, 12.41.

$[\alpha]^{20}_D$ = +1.8 (c 0.64, $CHCl_3$)

HRMS (EI^+) Calculated for $C_{18}H_{22}$ $(M)^+$: 238.17215, Found: 238.17177

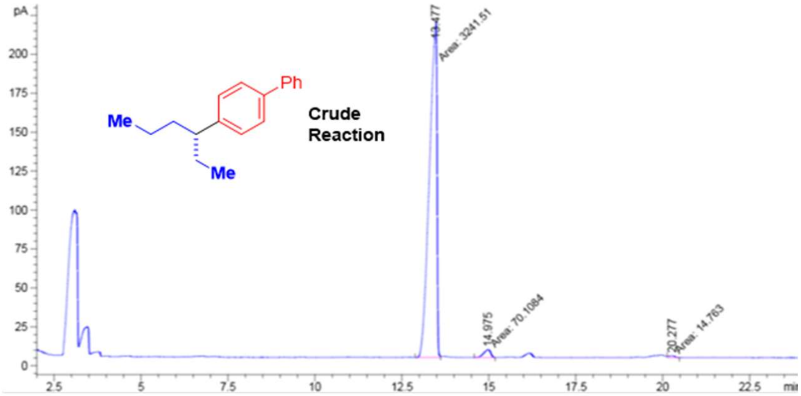
pA
13.477
Area: 3241.51
14.975
Area: 70.1084
20.277
Area: 14.763
Ph
Me
Me
Crude
Reaction
min

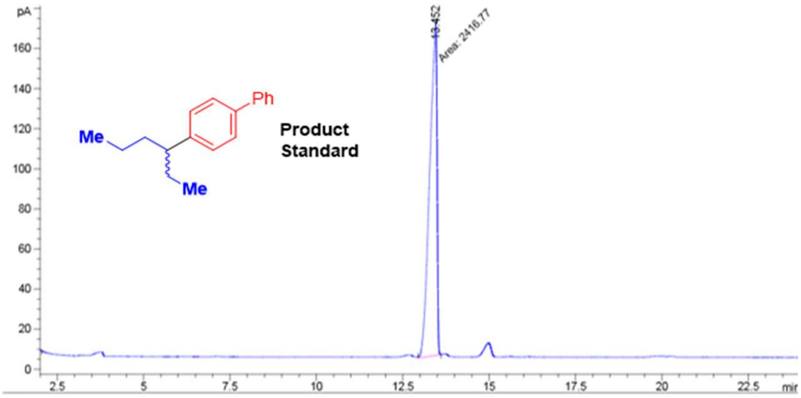
pA
13.452
Area: 2416.77
Ph
Me
Me
Product
Standard
min

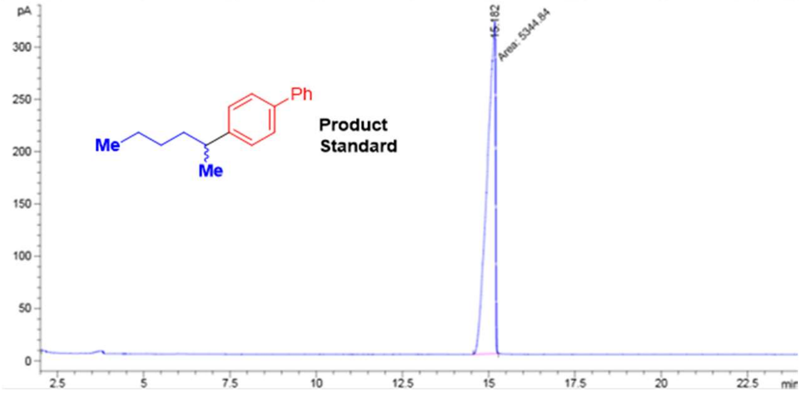
pA
15.182
Area: 5344.84
Ph
Me
Me
Product
Standard
min

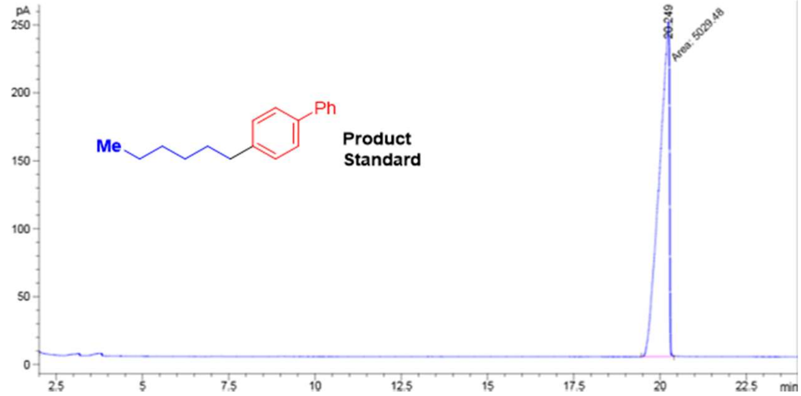
pA
20.249
Area: 5029.48
Ph
Me
Product
Standard
min

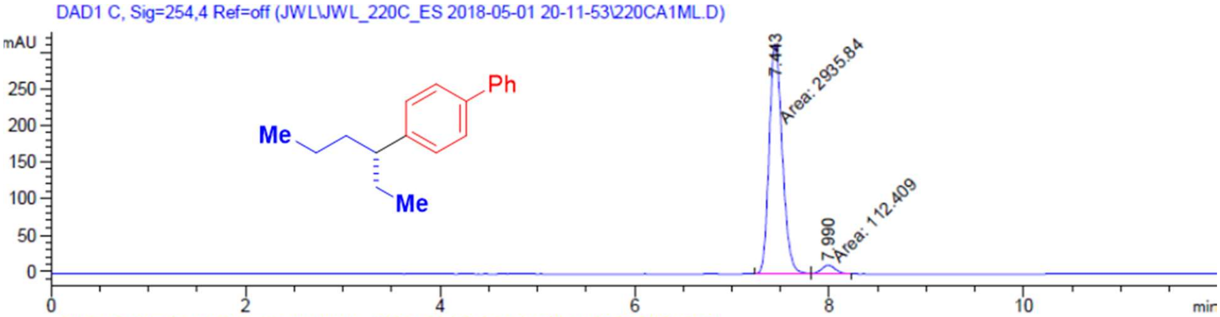

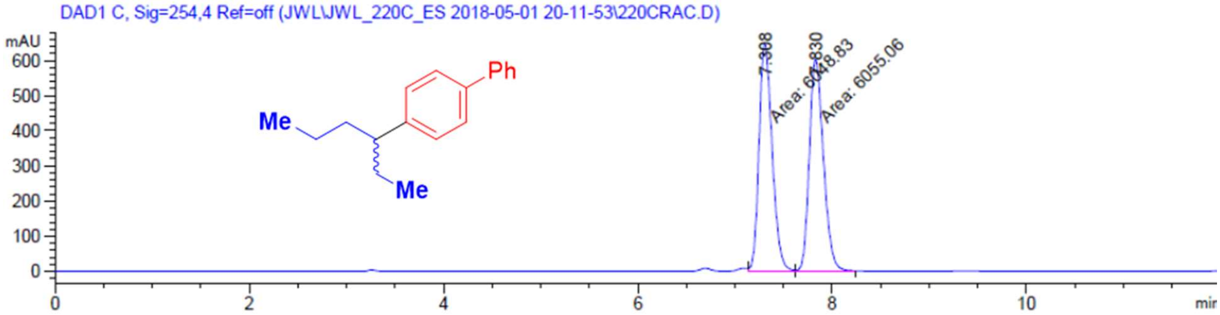

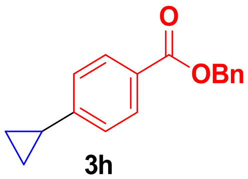

3h

3h was made from **1h** (prepared by general procedure C) and **2b** by general procedure D in 96% yield (24.2 mg). The product was isolated by silica column, eluting with 99:1 hexanes:EtOAc. A 1 mmol scale reaction gave 3s in 94% yield.

^{1}H NMR (500 MHz, $CDCl_3$) δ 7.96 (d, J = 8.4 Hz, 2H), 7.44 (d, J = 6.7 Hz, 2H), 7.36 (dt, J = 24.3, 7.2 Hz, 3H), 7.10 (d, J = 8.3 Hz, 2H), 5.35 (s, 2H), 1.94 (tt, J = 8.4, 5.0 Hz, 1H), 1.09 – 1.01 (m, 2H), 0.76 (dt, J = 6.6, 4.8 Hz, 2H).

^{13}C NMR (126 MHz, $CDCl_3$) δ 166.57, 150.27, 136.35, 129.89, 128.68, 128.27, 128.21, 127.27, 125.44, 66.57, 15.85, 10.47.

HRMS (EI+) Calculated for C17H16O2: 252.1150, Found: 252.1149

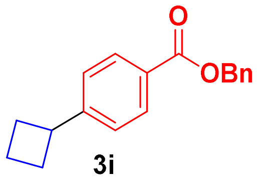

3i

3i was made from **1i** (prepared by general procedure C) and **2b** by general procedure D in 44% yield (11.7 mg). The product was isolated by reverse-phase MPLC with 13 g of C18 silica using a gradient of 1:1 MeCN:H_2O to 100% MeCN.

^{1}H NMR (500 MHz, $CDCl_3$) δ 8.00 (d, *J* = 8.0 Hz, 2H), 7.49 – 7.31 (m, 5H), 7.27 (d, *J* = 8.0 Hz, 2H), 5.36 (s, 2H), 3.60 (p, *J* = 8.6 Hz, 1H), 2.44 – 2.32 (m, 2H), 2.22 – 2.10 (m, 2H), 2.10 – 1.99 (m, 1H), 1.92 – 1.82 (m, 1H).

^{13}C NMR (126 MHz, $CDCl_3$) δ 166.63, 151.98, 136.36, 129.86, 128.70, 128.29, 128.23, 127.65, 126.43, 66.62, 40.38, 29.69, 18.43.

HRMS (EI$^+$) Calculated for $C_{18}H_{18}O_2$: 266.1307, Found: 266.1306

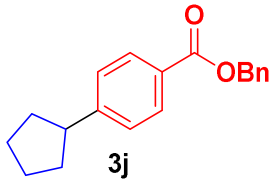

3j

3j was made from **1j** (prepared by general procedure C) and **2b** by general procedure D in 61% yield (17.1 mg). The product was isolated by reverse-phase MPLC with 13 g of C18 silica using a gradient of 1:1 MeCN:H_2O to 100% MeCN. A 1 mmol scale reaction gave **3j** in 42% NMR yield (118 mg) after partial purification using reverse-phase MPLC, which gave a mixture of the product with a small amounts of unidentified side products. The NMR yield of this partially purified product was obtained using 1,4-dimethoxybenzene as an internal standard.

^{1}H NMR (500 MHz, $CDCl_3$) δ 8.00 (d, *J* = 8.3 Hz, 2H), 7.48 – 7.42 (m, 2H), 7.42 – 7.32 (m, 3H), 7.31 (d, *J* = 8.1 Hz, 2H), 5.36 (s, 2H), 3.05 (ddd, *J* = 17.2, 9.6, 7.6 Hz, 1H), 2.15 – 2.03 (m, 2H), 1.88 – 1.76 (m, 2H), 1.76 – 1.66 (m, 2H), 1.66 – 1.53 (m, 2H).

^{13}C NMR (126 MHz, $CDCl_3$) δ 166.64, 152.53, 136.39, 129.89, 128.70, 128.28, 128.22, 127.73, 127.28, 66.60, 46.14, 34.67, 25.71.

HRMS (EI$^+$) Calculated for $C_{19}H_{20}O_2$: 280.1463, Found: 280.1463

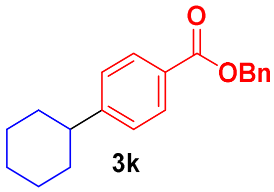

3k

3k was made from **1k** (prepared by general procedure C) and **2b** by general procedure D in 68% yield (20.0 mg). The product was isolated by reverse-phase MPLC with 13 g of C18 silica using a gradient of 1:1 MeCN:H_2O to 100% MeCN. A 1 mmol scale reaction gave **3k** in 79% yield (233 mg).

^{1}H NMR (500 MHz, $CDCl_3$) δ 8.00 (d, *J* = 8.3 Hz, 2H), 7.48 – 7.42 (m, 2H), 7.42 – 7.31 (m, 3H), 7.27 (d, *J* = 8.2 Hz, 2H), 5.35 (s, 2H), 2.62 – 2.50 (m, 1H), 1.94 – 1.80 (m, 4H), 1.76 (d, *J* = 12.7 Hz, 1H), 1.41 (h, *J* = 12.3 Hz, 4H), 1.33 – 1.19 (m, 1H).

^{13}C NMR (126 MHz, $CDCl_3$) δ 166.63, 153.77, 136.37, 129.97, 128.69, 128.28, 128.23, 127.82, 127.02, 66.59, 44.84, 34.27, 26.87, 26.17.

HRMS (EI$^+$) Calculated for $C_{20}H_{22}O_2$: 294.1620, Found: 294.1623

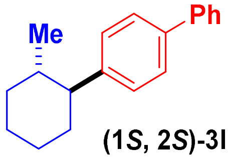

(1*S*, 2*S*)-3l

(1*S*, 2*S*)-3l was made from **(1*S*, 2*S*)-1l** and **2a** by general procedure D in 28% yield (7.0 mg). The product was isolated by preparative HPLC using 80:20 MeOH:H2O with a 25 mL/minute flow rate. The retention time of the desired product was 30 minutes. The isomeric product was isolated in 9% yield. These were the two major coupling products seen in the reaction mixture. Their NMR spectra matched those previously reported.[14] When the crude reaction mixture was subjected to reverse-phase MPLC, a 42% yield of product isomers resulted. This mixture showed 5 peaks with the mass of the coupled product. In addition, 31% yield of biphenyl was detected by quantitative HPLC assay. This side product presumably arises from β-hydride elimination of the L_nPd^{II} (Alkyl)(Ar) followed by reductive elimination of the resulting L_nPd^{II} (Alkyl)(Ar).

^{1}H NMR (500 MHz, CDCl3) δ 7.59 (d, *J* = 7.3 Hz, 2H), 7.52 (d, *J* = 8.1 Hz, 2H), 7.42 (t, *J* = 7.7 Hz, 2H), 7.32 (t, *J* = 7.4 Hz, 1H), 7.23 (d, *J* = 8.2 Hz, 2H), 2.12 (td, *J* = 11.6, 3.2 Hz, 1H), 1.89 – 1.74 (m, 4H), 1.68 – 1.56 (m, 1H), 1.53 – 1.31 (m, 3H), 1.11 (qd, *J* = 12.8, 3.3 Hz, 1H), 0.70 (d, *J* = 6.5 Hz, 3H).

^{13}C NMR (126 MHz, CDCl3) δ 146.21, 141.33, 138.75, 128.81, 128.07, 127.10, 127.07, 127.03, 52.29, 37.83, 35.94, 35.81, 27.13, 26.83, 21.02.

HRMS (EI$^+$) Calculated for C19H22: 250.1722, Found: 250.1728

Using boronic acid **(1*S*, 2*S*)-1l** made from diasteromerically pure BIDA boronate **(1*S*, 2*S*)-5l**, the coupling product **(1*S*, 2*S*)-3l** had an e.r. of > 99.5:0.5 (100% enantiospecificity), as determined by Chiralcel OD-H column, 100% hexanes, 2.0 mL/min., 254nm absorbance. Major: 3.7; Minor: 5.4.

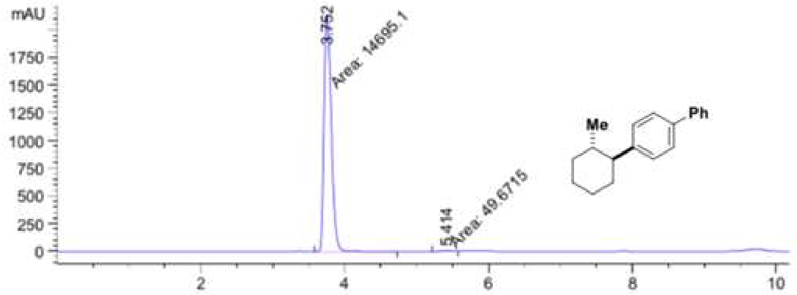

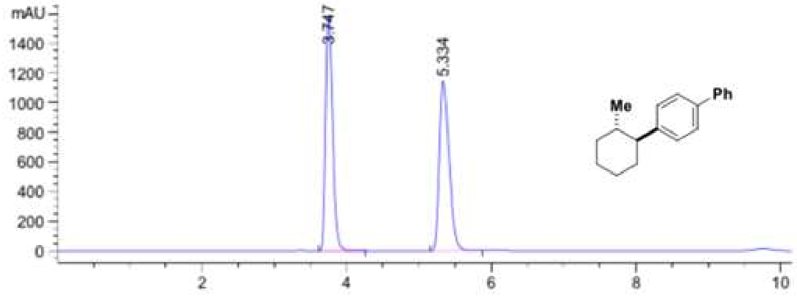

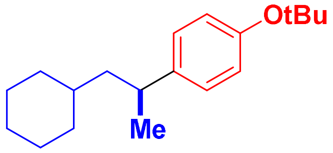

(+)-3m

Boronic acid ***(S)*-1d** (≥99:1 e.r.) was prepared in 67% yield by general procedure C and coupled to organohalide **2c** to give product **(+)-3m** by general procedure D. The branched/linear product ratio of the crude reaction was determined to be 187/1 by HPLC (Eclipse XDB-C8 column, isocratic 85/15 MeCN/H_2O, 1.2 mL/min, 215.4 nm absorbance). Branched = 9.4 minutes; linear = 10.2 minutes. The product was isolated in 76% yield (20.4 mg) by purification with reverse phase flash chromatography (15/1 MeCN/H_2O, R_f = 0.26). The e.r. of the purified product was determined to be 99.32 : 0.68 (>99% es) using chiral HPLC (OD-H chiral column, isocratic 100% hexanes, 2.0 mL/min, 214.4 nm absorance). Major = 3.07 minutes; minor = 3.82 minutes.

A duplicate run of the reaction gave a branched/linear product ratio of 160/1, isolated yield of 70%, and enantiospecificity of >99%.

^{1}H NMR (500 MHz, $CDCl_3$) δ 7.04 (d, *J* = 8.4 Hz, 2H), 6.89 (d, *J* = 8.4 Hz, 2H), 2.77 (sext, *J* = 7.3 Hz, 1H), 1.73 (d, *J* = 13.0 Hz, 1H), 1.70-1.55 (m, 4H), 1.44 (ddd, J = 14.3, 8.2, 6.6 Hz, 1H), 1.39-1.34 (m, 1H), 1.33 (s, 9H), 1.18 (d, J = 6.9 Hz, 3H), 1.16-1.08 (m, 4H), 0.90-0.80 (m, 2H).

^{13}C NMR (126 MHz, $CDCl_3$) δ 153.18, 143.17, 127.25, 124.09, 78.11, 46.68, 36.11, 35.26, 33.69, 33.62, 29.02, 26.87, 26.46, 22.84.

$[\alpha]^{20}_D$ = +19.3 (c 1.9, $CHCl_3$)

HRMS (EI^+) Calculated for $C_{19}H_{30}O$ $(M)^+$ 274.22967, Found: 274.22963

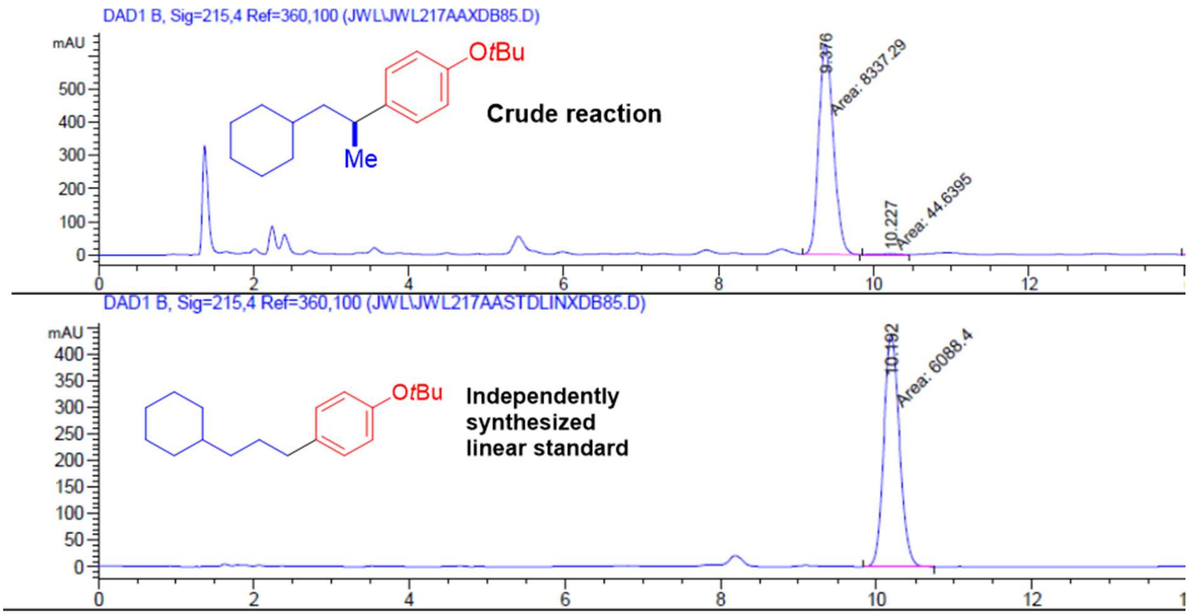

DAD1 B, Sig=215,4 Ref=360,100 (JWL\JWL217AAXDB85.D)
mAU
OtBu
Me
Crude reaction
9.376
Area: 8337.29
10.227
Area: 44.6395
DAD1 B, Sig=215,4 Ref=360,100 (JWL\JWL217AASTDLINXDB85.D)
mAU
OtBu
Independently synthesized linear standard
10.192
Area: 6088.4

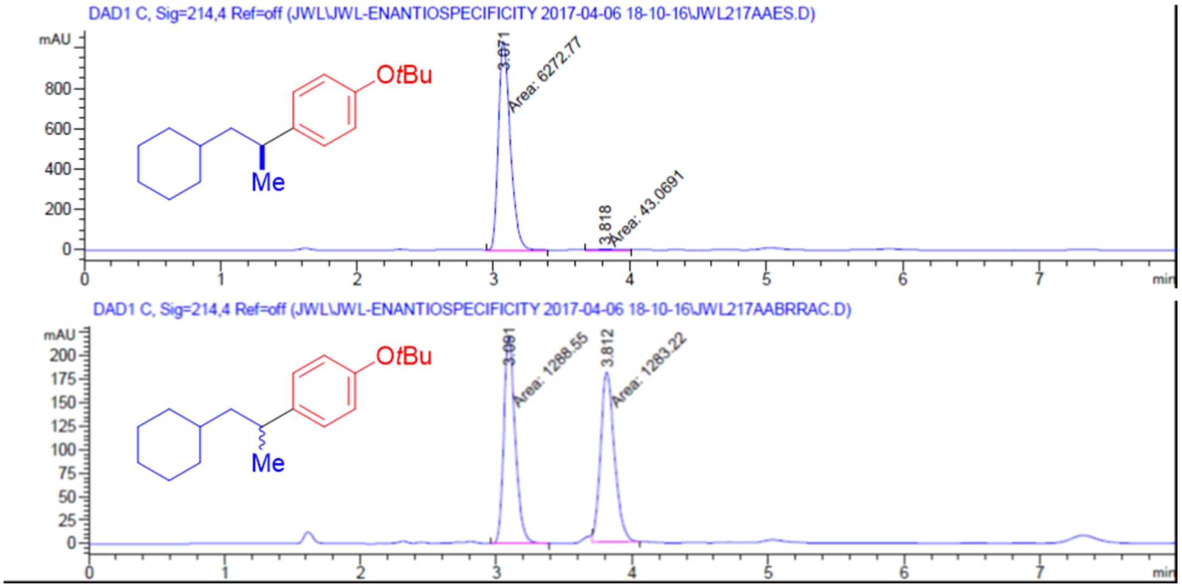

DAD1 C, Sig=214,4 Ref=off (JWL\JWL-ENANTIOSPECIFICITY 2017-04-06 18-10-16\JWL217AAES.D)
mAU
OtBu
Me
3.071
Area: 6272.77
3.818
Area: 43.0691
min
DAD1 C, Sig=214,4 Ref=off (JWL\JWL-ENANTIOSPECIFICITY 2017-04-06 18-10-16\JWL217AABRRAC.D)
mAU
OtBu
Me
3.091
Area: 1288.55
3.812
Area: 1283.22
min

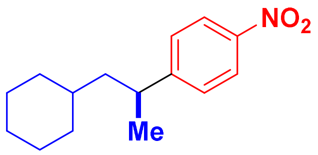

(+)-3n

Boronic acid **(*S*)-1d** (≥99:1 e.r.) was prepared in 66% yield by general procedure C and coupled to organohalide **2d** to give product **(+)-3n** by general procedure D. The branched/linear product ratio of the crude reaction was determined to be 108/1 by HPLC (Eclipse XDB-C8 column, isocratic 70/30 MeCN/H_2O, 1.2 mL/min, 215.4 nm absorbance). Branched = 12.7 minutes; linear = 14.2 minutes. The product was isolated in 59% yield (14.3 mg) by purification with reverse phase flash chromatography (8/1 MeCN/H_2O, R_f = 0.30) followed by normal phase flash chromatography (4/1 Hex/DCM, R_f = 0.27). The e.r. of the purified product was determined to be 98.65 : 1.35 (>99% es) using chiral HPLC (AD-RH chiral column, isocratic 75/25 MeCN/H_2O, 0.5 mL/min, 214 nm absorance). Major = 22.1 minutes; minor = 27.1 minutes.

A duplicate run of the reaction gave a branched/linear product ratio of 106/1, isolated yield of 62%, and enantiospecificity of >99% es.

^{1}H NMR (500 MHz, $CDCl_3$) δ 8.15 (d, *J* = 8.7 Hz, 2H), 7.33 (d, *J* = 8.7 Hz, 2H), 2.95 (sext, *J* = 8.2 Hz, 1H), 1.73 (d, *J* = 13.1 Hz, 1H), 1.70-1.58 (m, 4H), 1.51 (ddd, *J* = 14.4 Hz, 8.5 Hz, 6.2 Hz, 1H) 1.43 (dt, *J* = 13.9 Hz, 7.1 Hz, 1H), 1.23 Hz (d, *J* = 6.9 Hz, 3H), 1.18-1.03 (m, 4H), 0.93-0.82 (m, 2H)

^{13}C NMR (126 MHz, $CDCl_3$) δ 156.19, 146.40, 127.91, 123.85, 46.07, 37.04, 35.23, 33.75, 33.33, 26.69, 26.31, 22.63.

$[\alpha]^{20}_D$ = +56.5 (c 1.0, $CDCl_3$)

HRMS (EI$^+$) Calculated for $C_{15}H_{22}NO_2$ $(M+H)^+$: 248.1651, Found: 248.1660

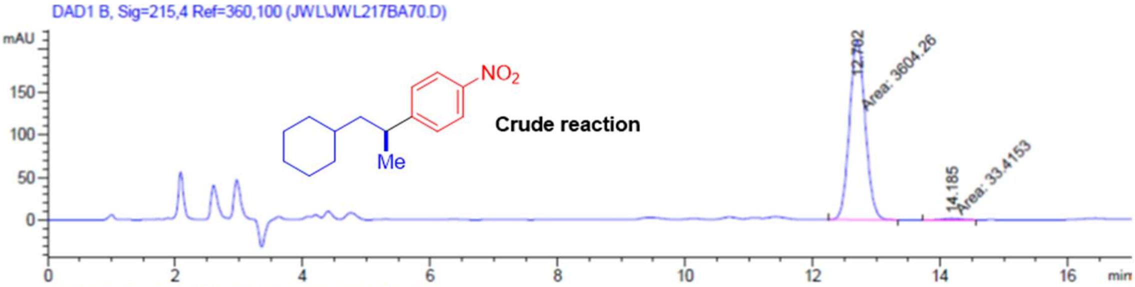
DAD1 B, Sig=215,4 Ref=360,100 (JWL\JWL217BA70.D)
mAU
NO2
Me
Crude reaction
12.702
Area: 3604.26
14.185
Area: 33.4153
min

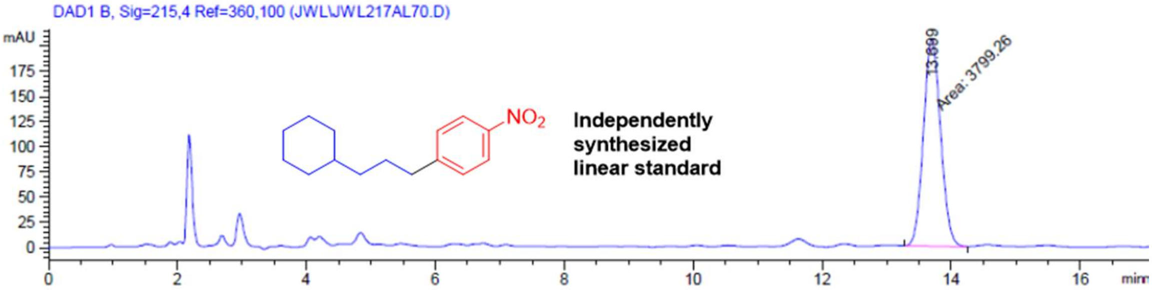
DAD1 B, Sig=215,4 Ref=360,100 (JWL\JWL217AL70.D)
mAU
NO2
Independently
synthesized
linear standard
13.699
Area: 3799.26
min

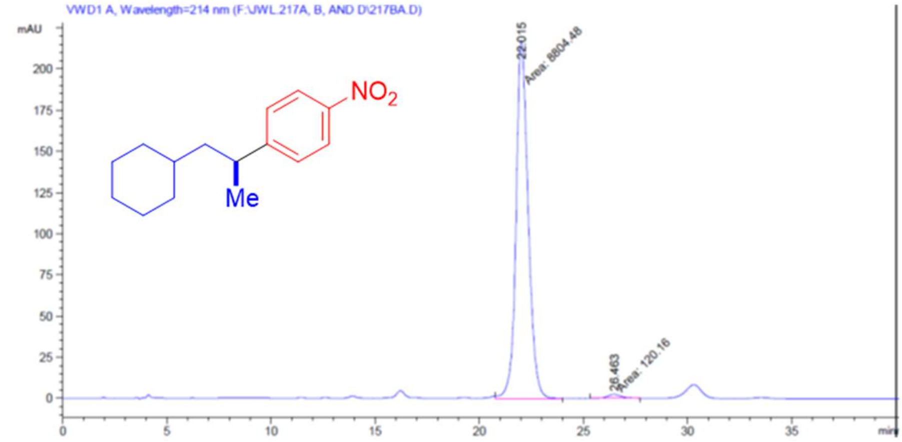
VWD1 A, Wavelength=214 nm (F:\JWL217A, B, AND D\217BA.D)
mAU
NO2
Me
22.015
Area: 8804.48
26.463
Area: 120.16
min

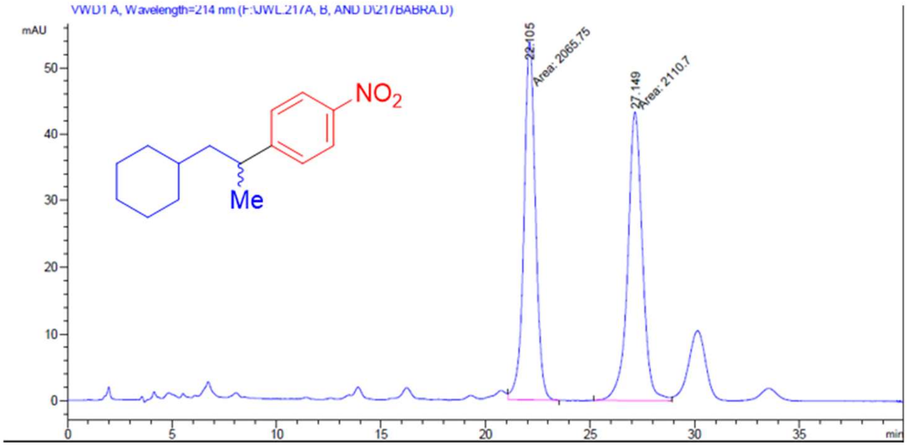
VWD1 A, Wavelength=214 nm (F:\JWL217A, B, AND D\217BABRA.D)
mAU
NO2
Me
22.105
Area: 2065.75
27.149
Area: 2110.7
min

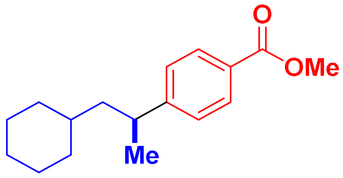

(+)-3o

Boronic acid ***(S)*-1d** (≥99:1 e.r.) was prepared in 67% yield by general procedure C and coupled to organohalide **2e** to give product **(+)-3o** by general procedure D. The branched/linear product ratio of the crude reaction was determined to be 220/1 by HPLC (Eclipse XDB-C8 column, isocratic 70/30 MeCN/H_2O, 1.2 mL/min, 254.4 nm absorbance). Branched = 13.7 minutes; linear = 15.5 minutes. The product was isolated in 84% yield (21.7 mg) by purification with reverse phase flash chromatography (10/1 MeCN/H_2O, R_f = 0.27). The e.r. of the purified product was determined to be 99.53 : 0.47 (>99% es) using chiral HPLC (OD-H chiral column, isocratic 100% hexanes, 2.0 mL/min, 214.4 nm absorance). Major = 6.4 minutes; minor = 8.3 minutes.

A duplicate run of the reaction gave a branched/linear product ratio of 207/1, isolated yield of 88%, and enantiospecificity of >99%.

^{1}H NMR (500 MHz, $CDCl_3$) δ 7.96 (d, *J* = 8.3 Hz, 2H), 7.24 (d, *J* = 8.3 Hz, 2H), 3.90 (s, 3H), 2.88 (h, *J* = 8.6 Hz, 1H), 1.74 (d, *J* = 11.3 Hz, 1H), 1.68-1.54 (m, 4H), 1.50 (ddd, *J* = 14.3, 8.6, 6.1 Hz, 1H), 1.40 (dt, *J* = 13.9, 7.1 Hz, 1H), 1.21 (d, *J* = 6.9 Hz, 3H), 1.17-1.04 (m, 4H), 0.91-0.80 (m, 2H).

^{13}C NMR (126 MHz, $CDCl_3$) δ 167.34, 153.85, 129.86, 127.89, 127.17, 52.06, 46.19, 36.98, 35.22, 33.81, 33.38, 26.77, 26.37, 22.73.

$[\alpha]^{20}_D$ = +39.1 (c 1.7, $CDCl_3$)

HRMS (AP^+) Calculated for $C_{17}H_{25}O_2$ $(M+H)^+$: 261.1855, Found: 261.1851

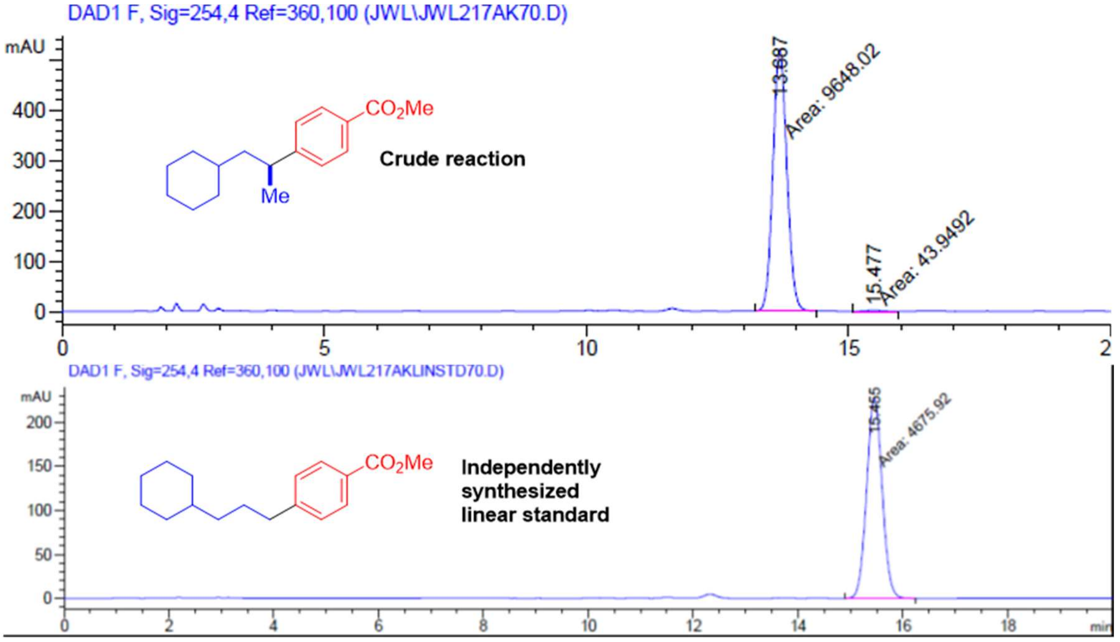
DAD1 F, Sig=254,4 Ref=360,100 (JWL\JWL217AK70.D)
mAU
13.687
Area: 9648.02
15.477
Area: 43.9492
CO2Me
Me
Crude reaction
DAD1 F, Sig=254,4 Ref=360,100 (JWL\JWL217AKLINSTD70.D)
15.455
Area: 4675.92
Independently
synthesized
linear standard

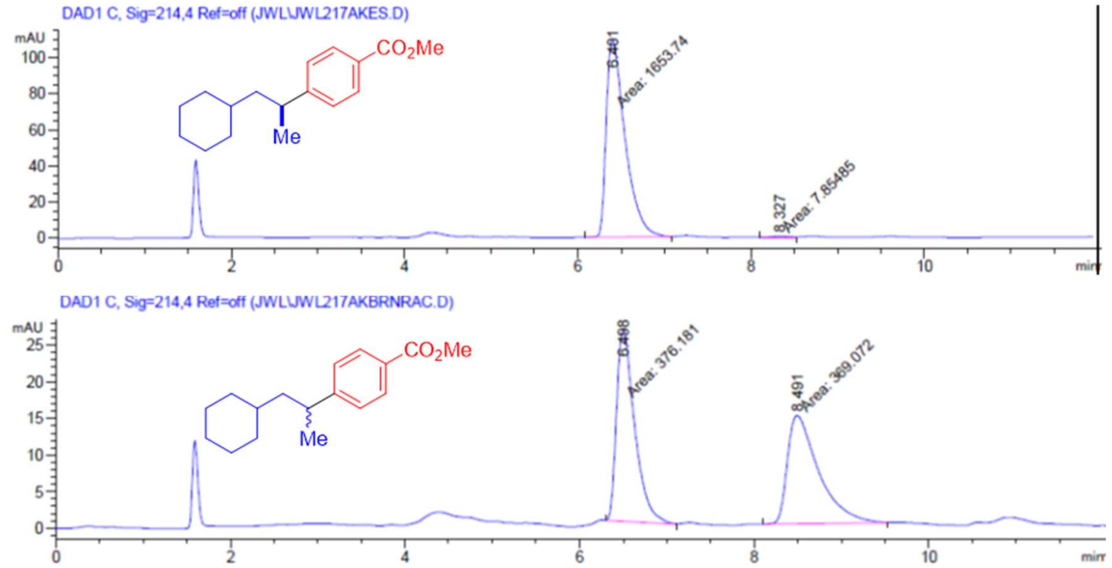
DAD1 C, Sig=214,4 Ref=off (JWL\JWL217AKES.D)
mAU
Area: 1653.74
8.327
Area: 7.85485
CO2Me
Me
DAD1 C, Sig=214,4 Ref=off (JWL\JWL217AKBRNRAC.D)
Area: 376.181
8.491
Area: 369.072

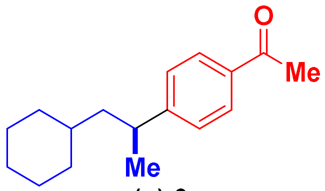

(+)-3p

Boronic acid ***(S)*-1d** (≥99:1 e.r.) was prepared in 66% yield by general procedure C and coupled to organohalide **2f** to give product **(+)-3p** by general procedure D. The branched/linear product ratio of the crude reaction was determined to be 274/1 by HPLC (Eclipse XDB-C8 column, isocratic 70/30 MeCN/H_2O, 1.2 mL/min, 254.4 nm absorbance). Branched = 9.8 minutes; linear = 11.1 minutes. The product was isolated in 54% yield (13.6 mg) by purification with reverse phase flash chromatography (6/1 MeCN/H_2O, R_f = 0.23) followed by normal phase flash chromatography (1/2 Hex/DCM, R_f = 0.29). The e.r. of the purified product was determined to be 99.64 : 0.36 (>99% es) using chiral HPLC (AD-RH chiral column, isocratic 72/28 MeCN/H_2O, 0.5 mL/min, 214 nm absorance). Major = 27.4 minutes; minor = 33.5 minutes.

A duplicate run of the reaction gave a branched/linear product ratio of 285/1, isolated yield of 61%, and enantiospecificity of >99%.

^{1}H NMR (500 MHz, $CDCl_3$) δ 7.89 (d, J = 8.3 Hz, 2H), 7.27 (d, J = 8.3 Hz, 2H), 2.89 (sext, *J* = 8.3 Hz, 1H), 2.59 (s, 3H), 1.74 (d, *J* = 12.6 Hz, 1H), 1.68-1.58 (m, 4H), 1.51 (ddd, *J* = 14.3, 8.6, 6.1 Hz, 1H), 1.41 (m, 1H), 1.21 (d, *J* = 6.9 Hz, 3H), 1.18-1.05 (m, 4H), 0.91-0.82 (m, 2H).

^{13}C NMR (126 MHz, $CDCl_3$) δ 198.04, 154.17, 135.21, 128.71, 127.33, 46.14, 37.00, 35.22, 33.80, 33.36, 26.76, 26.69, 26.36, 26.34, 22.71

$[\alpha]^{20}_D$ = +24.2 (c 1.0, $CHCl_3$)

HRMS (EI^+) Calculated for $C_{17}H_{24}O$ $(M)^+$: 244.18272, Found: 244.18204

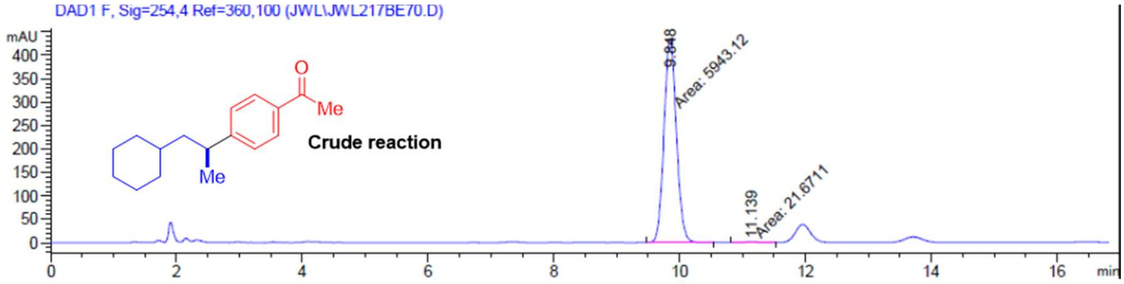
DAD1 F, Sig=254,4 Ref=360,100 (JWL\JWL217BE70.D)
mAU
9.848
Area: 5943.12
11.139
Area: 21.6711
Crude reaction
Me
Me
min

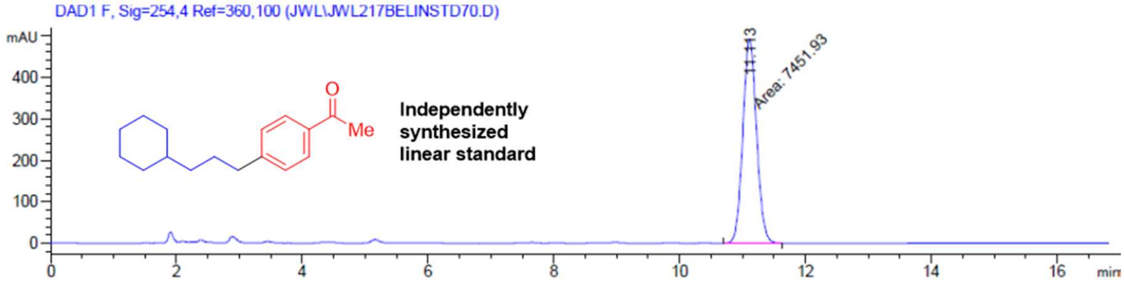
DAD1 F, Sig=254,4 Ref=360,100 (JWL\JWL217BELINSTD70.D)
mAU
11.113
Area: 7451.93
Independently
synthesized
linear standard
Me
min

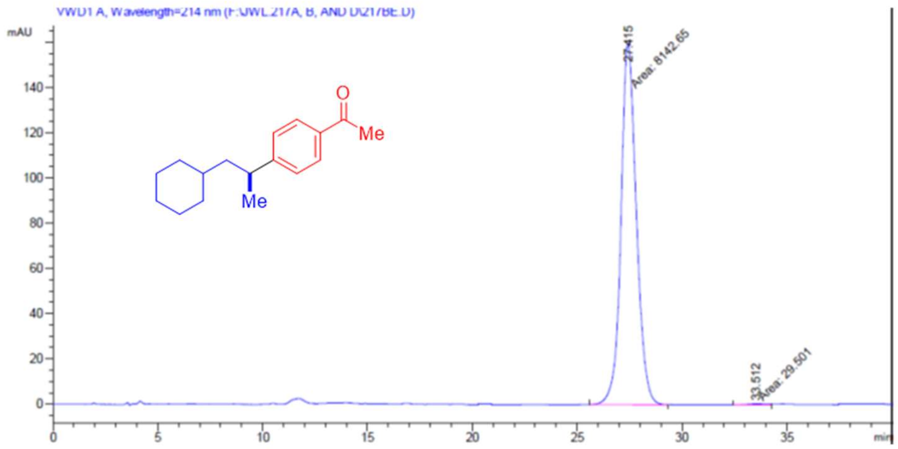
VWD1 A, Wavelength=214 nm (F:\JWL.217A, B, AND D\217BE.D)
mAU
27.415
Area: 8142.65
33.512
Area: 29.501
Me
Me
min

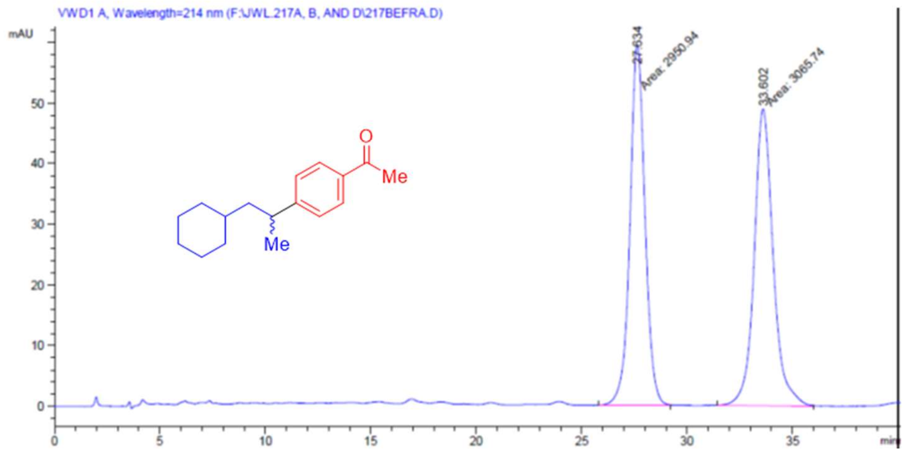
VWD1 A, Wavelength=214 nm (F:\JWL.217A, B, AND D\217BEFRA.D)
mAU
27.634
Area: 2950.94
33.602
Area: 3065.74
Me
Me
min

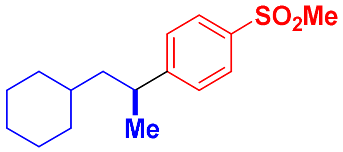

(-)-3q

Boronic acid **(*S*)-1d** (≥99:1 e.r.) was prepared in 65% yield by general procedure C and coupled to organohalide **2g** to give product **(-)-3q** by general procedure D. The branched/linear product ratio of the crude reaction was determined to be 118/1 by HPLC (Eclipse XDB-C8 column, isocratic 68/32 MeCN/H_2O, 1.2 mL/min, 230 nm absorbance). Branched = 6.3 minutes; linear = 7.0 minutes. The product was isolated in 78% yield (21.8 mg) by purification with reverse phase flash chromatography (4/1 MeCN/H_2O, R_f = 0.33) as a clear oil. The e.r. of the purified product was determined to be 99.74 : 0.26 (>99% es) using chiral HPLC (AD-RH chiral column, isocratic 75/25 MeCN/H_2O, 0.5 mL/min, 214 nm absorance). Major = 11.9 minutes; minor = 17.2 minutes.

A duplicate run of the reaction gave a branched/linear product ratio of 119/1, isolated yield of 82%, and enantiospecificity of >99%.

^{1}H NMR (500 MHz, $CDCl_3$) δ 7.85 (d, J = 8.3 Hz, 2H), 7.37 (d, J = 8.3 Hz, 2H), 3.06 (s, 3H), 2.93 (sext, *J* = 8.0 Hz, 1H), 1.73 (d, 12.8 Hz, 1H), 1.69-1.58 (m, 4H), 1.51 (ddd, J = 14.3, 8.5, 6.2 Hz, 1H), 1.43 (dt, J = 13.9, 7.1 Hz, 1H), 1.22 (d, J = 6.9 Hz, 3H), 1.19-1.04 (m, 4H), 0.92-0.82 (m, 2H).

^{13}C NMR (126 MHz, $CDCl_3$) δ 154.92, 138.02, 128.09, 127.62, 46.08, 44.70, 36.99, 35.13, 33.72, 33.31, 26.69, 26.30, 26.28, 22.65.

$[\alpha]^{20}_D$ = -79.6 (c 1.0, $CHCl_3$)

HRMS (EI^+) Calculated for $C_{16}H_{24}O_2S$ $(M)^+$: 280.14970, Found: 280.14856

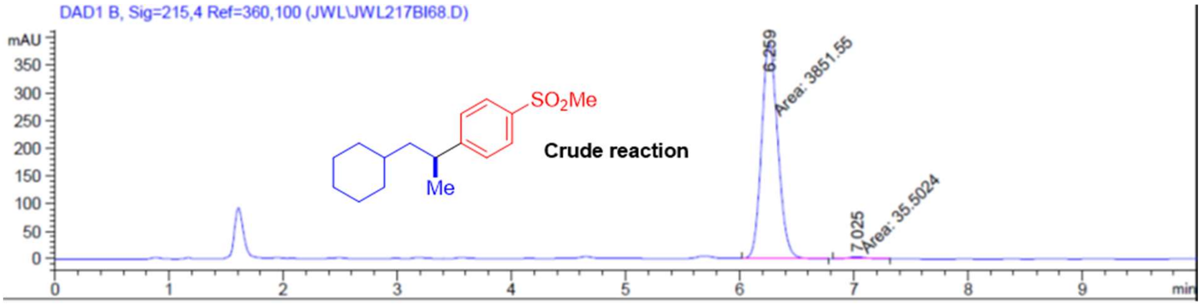
DAD1 B, Sig=215,4 Ref=360,100 (JWL\JWL217BI68.D)
mAU
SO2Me
Me
Crude reaction
6.259
Area: 3851.55
7.025
Area: 35.5024
min

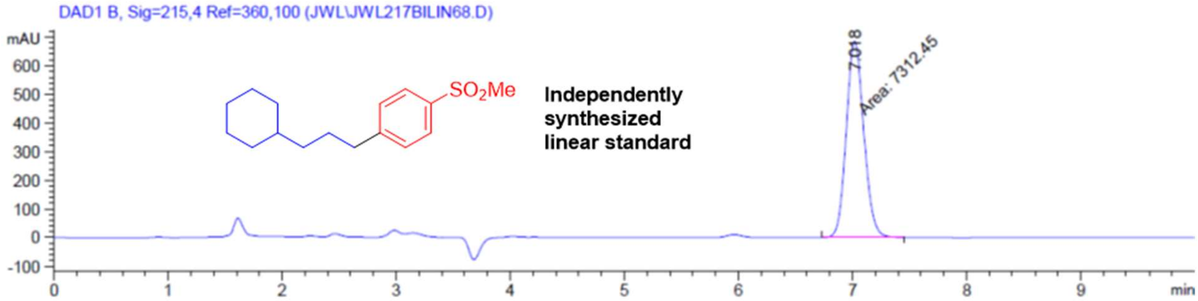
DAD1 B, Sig=215,4 Ref=360,100 (JWL\JWL217BILIN68.D)
mAU
SO2Me
Independently synthesized linear standard
7.018
Area: 7312.45
min

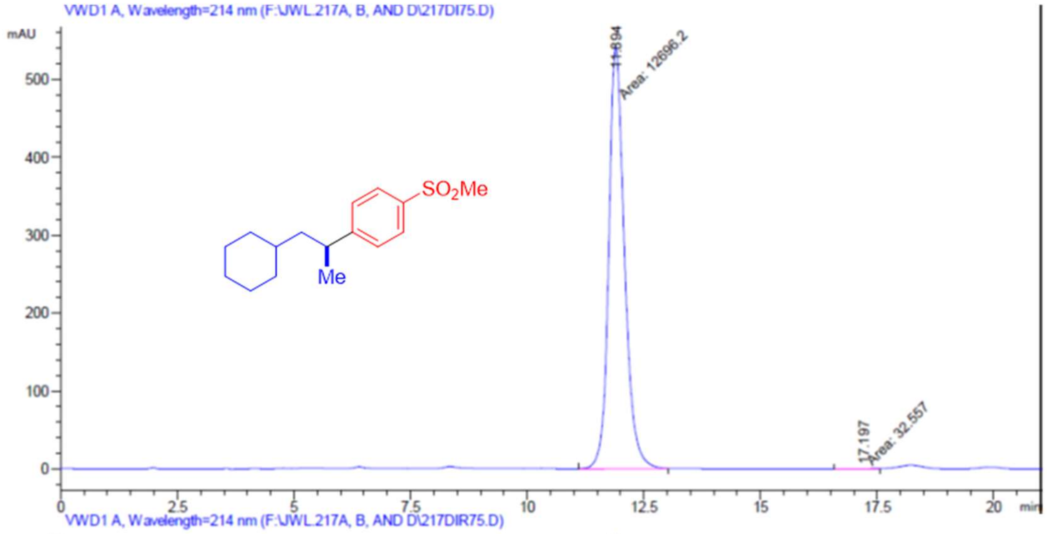
VWD1 A, Wavelength=214 nm (F:\JWL.217A, B, AND D\217DI75.D)
mAU
SO2Me
Me
11.894
Area: 12696.2
17.197
Area: 32.557
min

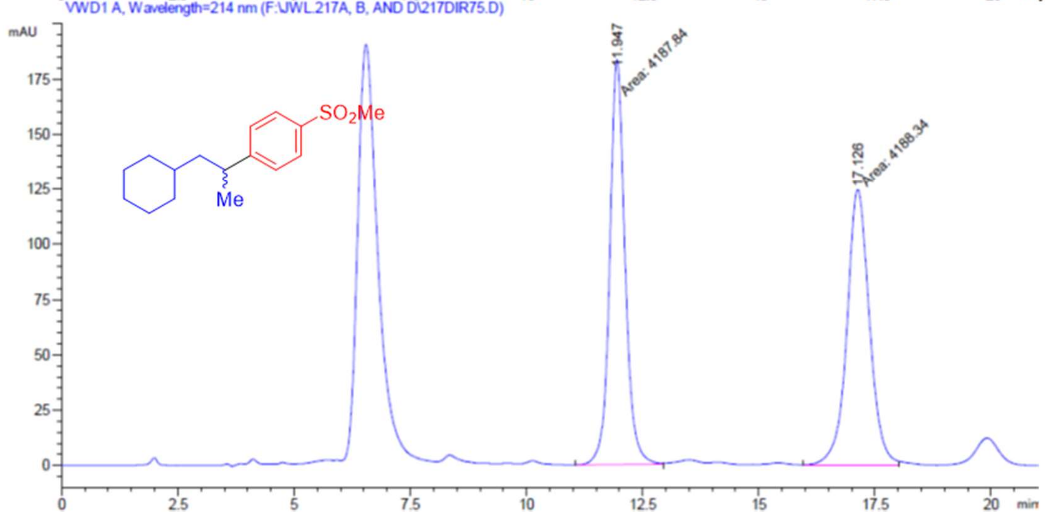
VWD1 A, Wavelength=214 nm (F:\JWL.217A, B, AND D\217DIR75.D)
mAU
SO2Me
Me
11.947
Area: 4187.84
17.126
Area: 4188.34
min

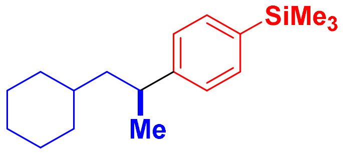

(-)-3r

Boronic acid ***(S)*-1d** (≥99:1 e.r.) was prepared in 65% yield by general procedure C and coupled to organohalide **2h** to give product **(-)-3r** by general procedure D. The branched/linear product ratio of the crude reaction was determined to be 596/1 by HPLC (Eclipse XDB-C8 column, isocratic 85/15 MeCN/H_2O, 1.2 mL/min, 214.4 nm absorbance). Branched = 14.6 minutes; linear = 16.5 minutes. The product was isolated in 67% yield (19.8 mg) by purification with reverse phase flash chromatography (20/1 MeCN/H_2O, R_f = 0.21) as clear oil. The e.r. of the purified product was determined to be 99.67 : 0.33 (>99% es) using chiral HPLC (AD-RH chiral column, isocratic 80/20 MeCN/H_2O, 0.5 mL/min, 214.4 nm absorance). Major = 19.3 minutes; minor = 21.6 minutes.

A duplicate run of the reaction gave a branched/linear product ratio of 624/1, isolated yield of 78%, and enantiospecificity of >99%.

^{1}H NMR (500 MHz, $CDCl_3$) δ 7.44 (d, *J* = 7.9 Hz, 2H), 7.17 (d, *J* = 7.9 Hz, 2H), 2.81 (sext, *J* = 7.1 Hz, 1H), 1.75 (d, *J* = 13.0 Hz, 1H), 1.69-1.58 (m, 4H), 1.50 (ddd, *J* = 14.1, 7.9, 6.5 Hz, 1H), 1.38 (dt, *J* = 13.8, 7.2 Hz, 1H), 1.20 (d, J = 6.9 Hz, 3H), 1.19-1.06 (m, 4H), 0.91-0.81 (m, 2H), 0.26 (s, 9H).

^{13}C NMR (126 MHz, $CDCl_3$) δ 149.03, 137.30, 133.53, 126.58, 46.45, 36.64, 35.14, 33.68, 33.63, 26.87, 26.40, 22.66, -0.85.

$[\alpha]^{20}_D$ = -110.0 (c 1.0, $CDCl_3$)

HRMS (EI^+) Calculated for $C_{18}H_{30}Si$ $(M)^+$: 274.21168, Found: 274.21214

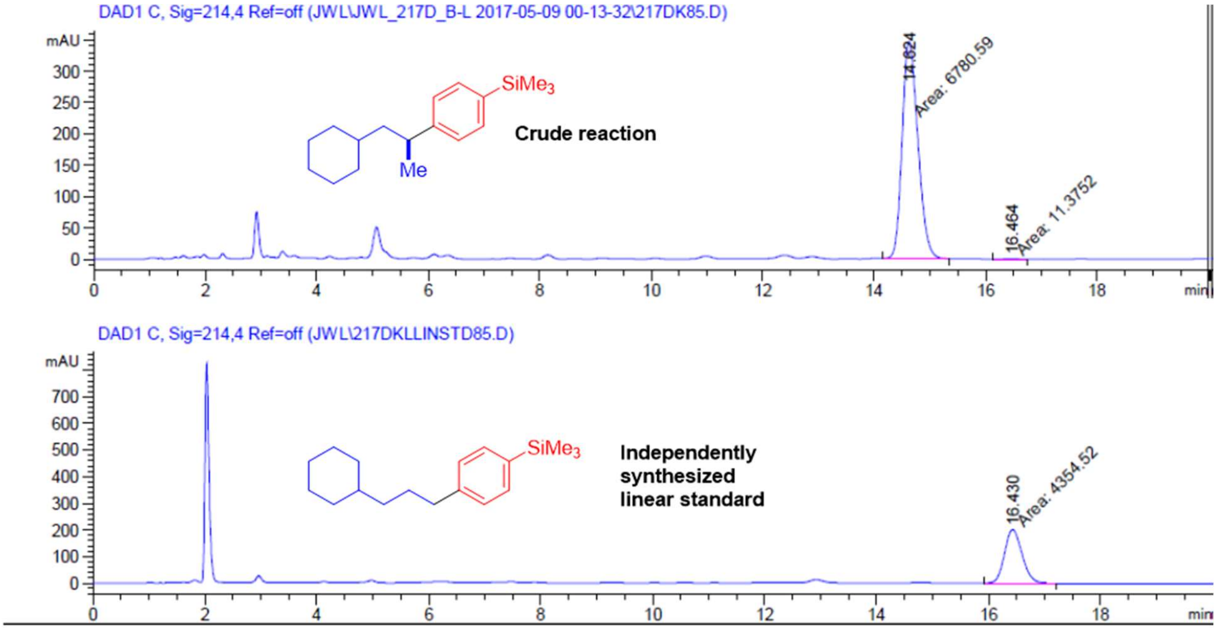

DAD1 C, Sig=214,4 Ref=off (JWL\JWL_217D_B-L 2017-05-09 00-13-32\217DK85.D)
mAU
SiMe3
Me
Crude reaction
14.624
Area: 6780.59
16.464
Area: 11.3752
min
DAD1 C, Sig=214,4 Ref=off (JWL\217DKLLINSTD85.D)
mAU
SiMe3
Independently
synthesized
linear standard
16.430
Area: 4354.52
min

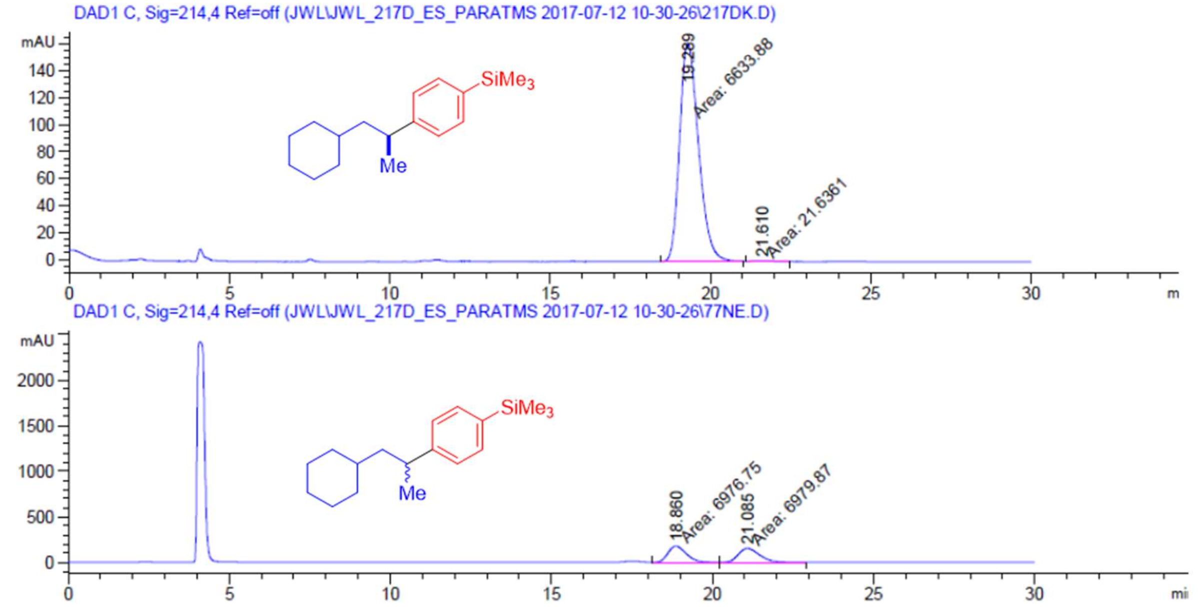

DAD1 C, Sig=214,4 Ref=off (JWL\JWL_217D_ES_PARATMS 2017-07-12 10-30-26\217DK.D)
mAU
SiMe3
Me
19.289
Area: 6633.88
21.610
Area: 21.6361
DAD1 C, Sig=214,4 Ref=off (JWL\JWL_217D_ES_PARATMS 2017-07-12 10-30-26\77NE.D)
mAU
SiMe3
Me
18.860
Area: 6976.75
21.085
Area: 6979.87

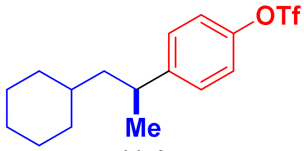

(-)-3s

Boronic acid **(*S*)-1d** (≥99:1 e.r.) was prepared in 65% yield by general procedure C and coupled to organohalide **2i** to give product **(-)-3s** by general procedure D. The branched/linear product ratio of the crude reaction was determined to be 74/1 by HPLC (Eclipse XDB-C8 column, isocratic 80/20 MeCN/H_2O, 1.2 mL/min, 220.4 nm absorbance). Branched = 9.8 minutes; linear = 10.9 minutes. The product was isolated in 81% yield (29.5 mg) by purification with reverse phase flash chromatography (8/1 MeCN/H_2O, R_f = 0.30) as clear oil. The e.r. of the purified product was determined to be 99.41 : 0.59 (>99% es) using chiral HPLC (AD-RH chiral column, isocratic 75/25 MeCN/H_2O, 0.5 mL/min, 214.4 nm absorance). Major = 9.1 minutes; minor = 10.6 minutes.

A duplicate run of the reaction gave a branched/linear product ratio of 76/1, isolated yield of 76%, and enantiospecificity of >99%.

^1H NMR (500 MHz, $CDCl_3$) δ 7.24 (d, J = 8.7 Hz, 2H), 7.17 (d, J = 8.7 Hz, 2H), 2.86 (sext, J = 7.1 Hz, 1H), 1.72 (d, J = 12.4 Hz, 1H), 1.69-1.58 (m, 4H), 1.46 (ddd, J = 14.3, 8.1, 6.4 Hz, 1H), 1.39 (dt, J = 13.9, 7.2 Hz, 1H), 1.19 (d, J = 6.9 Hz, 3H), 1.18-1.06 (m, 4H), 0.91-0.82 (m, 2H).

^{13}C NMR (126 MHz, $CDCl_3$) δ 148.86, 147.72, 128.76, 121.20, 118.9 (q, *J* = 319 Hz), 46.35, 36.37, 35.16, 33.67, 33.47, 26.76, 26.35, 22.75.

^{19}F NMR (470 Hz, $CDCl_3$) δ -75.06

$[\alpha]^{20}_D$ = -62.6 (c 1.0, $CHCl_3$)

HRMS (EI^+) Calculated for $C_{16}H_{21}O_3F_3S$ $(M)^+$: 350.11636, Found: 350.11738

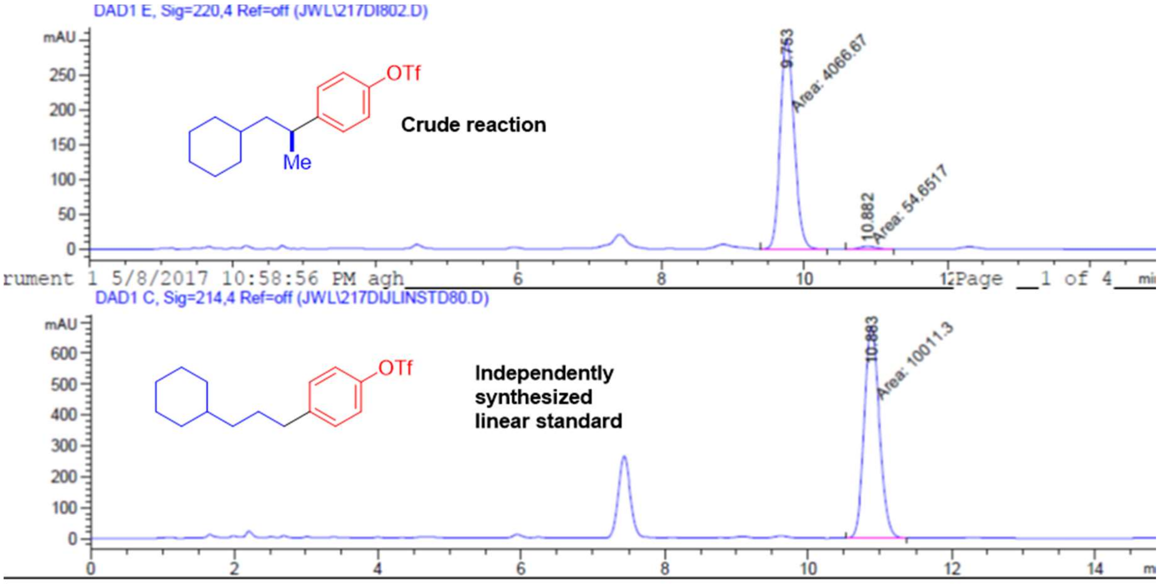
DAD1 E, Sig=220,4 Ref=off (JWL\217DI802.D)
mAU
OTf
Me
Crude reaction
9.753
Area: 4066.67
10.882
Area: 54.6517
rument 1 5/8/2017 10:58:56 PM agh
Page 1 of 4
DAD1 C, Sig=214,4 Ref=off (JWL\217DIJLINSTD80.D)
OTf
Independently synthesized linear standard
10.883
Area: 10011.3

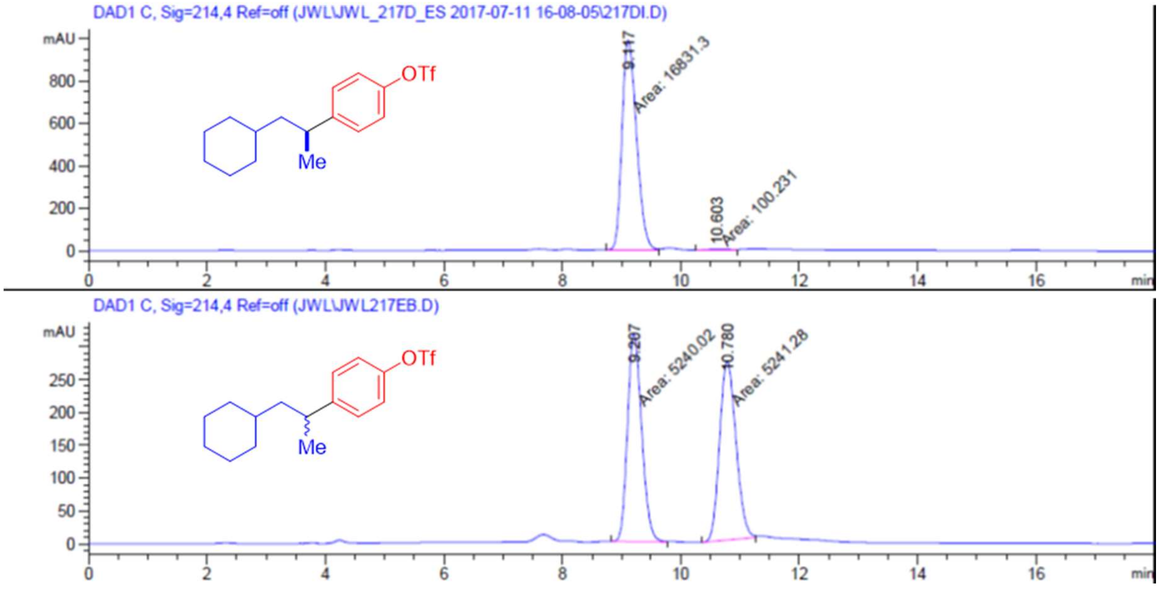
DAD1 C, Sig=214,4 Ref=off (JWL\JWL_217D_ES 2017-07-11 16-08-05\217DI.D)
mAU
OTf
Me
9.117
Area: 16831.3
10.603
Area: 100.231
DAD1 C, Sig=214,4 Ref=off (JWL\JWL217EB.D)
OTf
Me
9.267
Area: 5240.02
10.780
Area: 5241.28
min

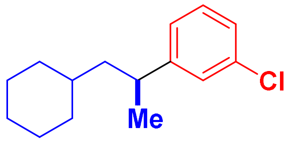

(+)-3t

Boronic acid **(*S*)-1d** (≥99:1 e.r.) was prepared in 66% yield by general procedure C and coupled to organohalide **2j** to give product **(+)-3t** by general procedure D. The branched/linear product ratio of the crude reaction was determined to be 94/1 by HPLC (Eclipse XDB-C8 column, isocratic 83/17 MeCN/H_2O, 1.2 mL/min, 215.4 nm absorbance). Branched = 8.5 minutes; linear = 9.6 minutes. The product was isolated in 78% yield (20.6 mg) by purification with reverse phase flash chromatography (15/1 MeCN/H_2O, R_f = 0.25). The e.r. of the purified product was determined to be 99.18 : 0.82 (100% es) using chiral HPLC (AD-RH chiral column, isocratic 75/25 MeCN/H_2O, 0.5 mL/min, 214 nm absorance). Major = 13.2 minutes; minor = 11.6 minutes.

A duplicate run of the reaction gave a branched/linear product ratio of 86/1, isolated yield of 81%, and enantiospecificity of 100%.

^{1}H NMR (500 MHz, $CDCl_3$) δ 7.20 (td, *J* = 7.6, 0.9 Hz, 1H), 7.17-7.13 (m, 2H), 7.05 (dt, *J* = 7.6, 1.4 Hz, 1H), 2.80 (sext, *J* = 7.7 Hz, 1H), 1.73 (d, *J* = 12.9 Hz, 1H), 1.69-1.58 (m, 4H), 1.47 (m, 1H), 1.37 (dt, *J* = 13.9, 7.2 Hz, 1H), 1.18 (d, *J* = 6.9 Hz, 3H), 1.18-1.07 (m, 4H), 0.92-0.81 (m, 2H).

^{13}C NMR (126 MHz, $CDCl_3$) δ 150.45, 134.18, 129.68, 127.24, 126.02, 125.40, 46.25, 36.70, 35.15, 33.73, 33.45, 26.80, 26.37, 22.78

$[\alpha]^{20}_D$ = +6.7 (c 1.0, $CHCl_3$)

HRMS (EI^+) Calculated for $C_{15}H_{21}Cl$ $(M)^+$: 236.13318, Found: 236.13205

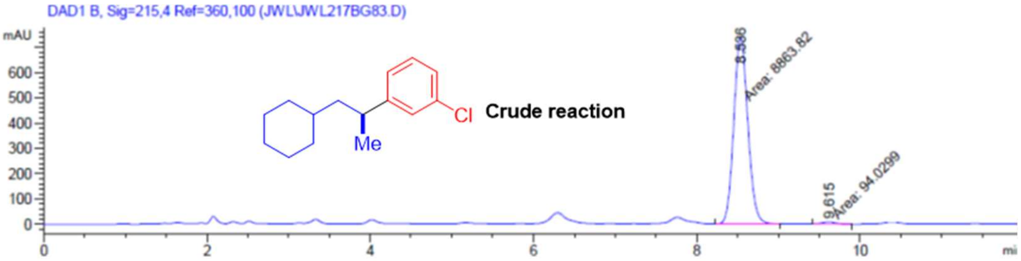
DAD1 B, Sig=215,4 Ref=360,100 (JWL\JWL217BG83.D)
mAU
Cl
Me
Crude reaction
8.536
Area: 8863.82
9.615
Area: 94.0299
min

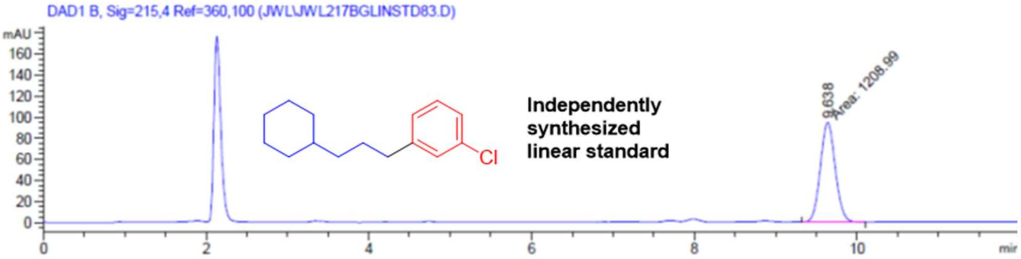
DAD1 B, Sig=215,4 Ref=360,100 (JWL\JWL217BGLINSTD83.D)
mAU
Cl
Independently
synthesized
linear standard
9.638
Area: 1208.99
min

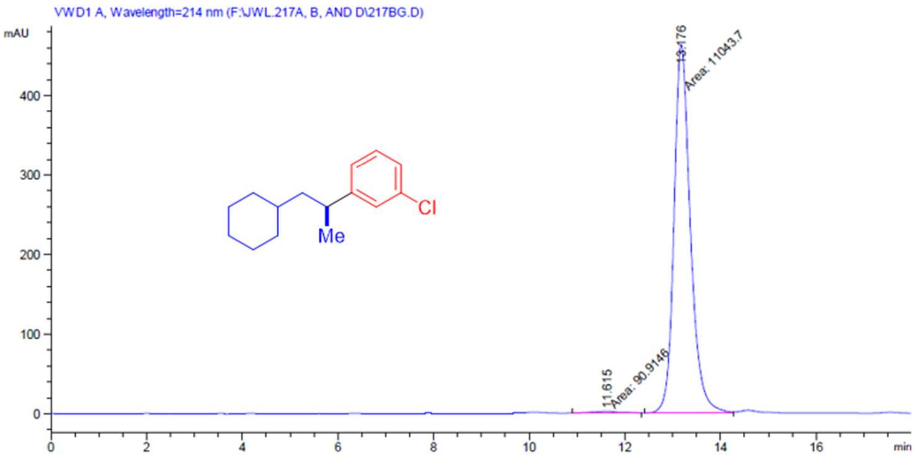
VWD1 A, Wavelength=214 nm (F:\JWL.217A, B, AND D\217BG.D)
mAU
Cl
Me
11.615
Area: 90.9146
13.176
Area: 11043.7
min

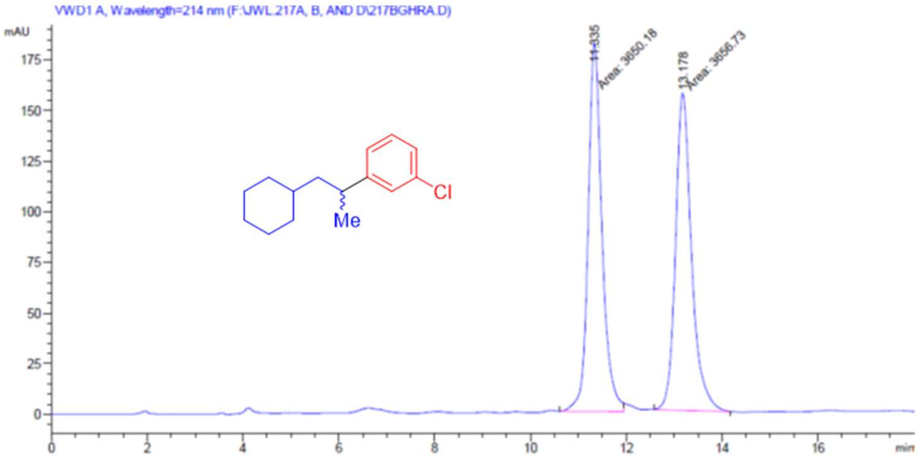
VWD1 A, Wavelength=214 nm (F:\JWL.217A, B, AND D\217BGHRA.D)
mAU
Cl
Me
11.335
Area: 3650.18
13.178
Area: 3656.73
min

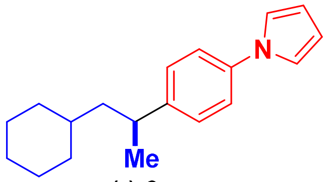

(-)-3u

Boronic acid **(*S*)-1d** (≥99:1 e.r.) was prepared in 65% yield by general procedure C and coupled to organohalide **2k** to give product **(-)-3u** by general procedure D. The branched/linear product ratio of the crude reaction was determined to be 266/1 by HPLC (Eclipse XDB-C8 column, isocratic 75/25 MeCN/H_2O, 1.2 mL/min, 250 nm absorbance). Branched = 14.6 minutes; linear = 16.4 minutes. The product was isolated in 68% yield (18.3 mg) by purification with reverse phase flash chromatography (9/1 MeCN/H_2O, R_f = 0.26) as an off-white solid. The e.r. of the purified product was determined to be 99.70 : 0.30 (>99% es) using chiral HPLC (AD-RH chiral column, isocratic 85/15 MeCN/H_2O, 0.5 mL/min, 214 nm absorance). Major = 14.7 minutes; minor = 24.7 minutes.

A duplicate run of the reaction gave a branched/linear product ratio of 246/1, isolated yield of 62%, and enantiospecificity of >99%.

^{1}H NMR (500 MHz, $CDCl_3$) δ 7.31 (d, *J* = 8.4 Hz, 2H), 7.22 (d, *J* = 8.5 Hz, 2H), 7.07 (t, *J* = 2.1 Hz, 2H), 6.33 (t, *J* = 2.1 Hz, 2H), 2.85 (sext, *J* = 7.9 Hz, 1H), 1.77 (d, *J* = 13.1 Hz, 1H), 1.70-1.59 (m, 4H), 1.50 (m, 1H), 1.41 (dt, *J* = 13.9, 7.1 Hz, 1H), 1.22 (d, *J* = 6.8 Hz, 3H), 1.19-1.10 (m, 4H), 0.93-0.83 (m, 2H).

^{13}C NMR (126 MHz, $CDCl_3$) δ 145.84, 138.79, 128.07, 120.73, 119.52, 110.14, 46.48, 36.28, 35.22, 33.80, 33.46, 26.82, 26.41, 26.41, 22.96

$[\alpha]^{20}_D$ = -112.8 (c 1.0, $CHCl_3$)

HRMS (EI^+) Calculated for $C_{19}H_{25}N$ $(M)^+$: 267.19870, Found: 267.19823

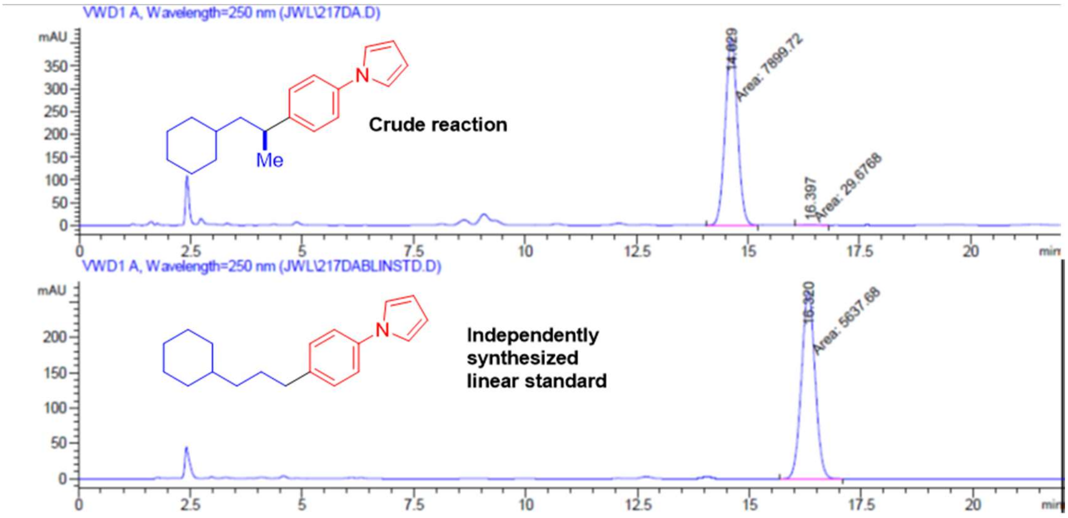
VWD1 A, Wavelength=250 nm (JWL\217DA.D)
Crude reaction
N
Me
14.629
Area: 7899.72
16.397
Area: 29.6768
VWD1 A, Wavelength=250 nm (JWL\217DABLINSTD.D)
N
Independently synthesized linear standard
16.320
Area: 5637.68

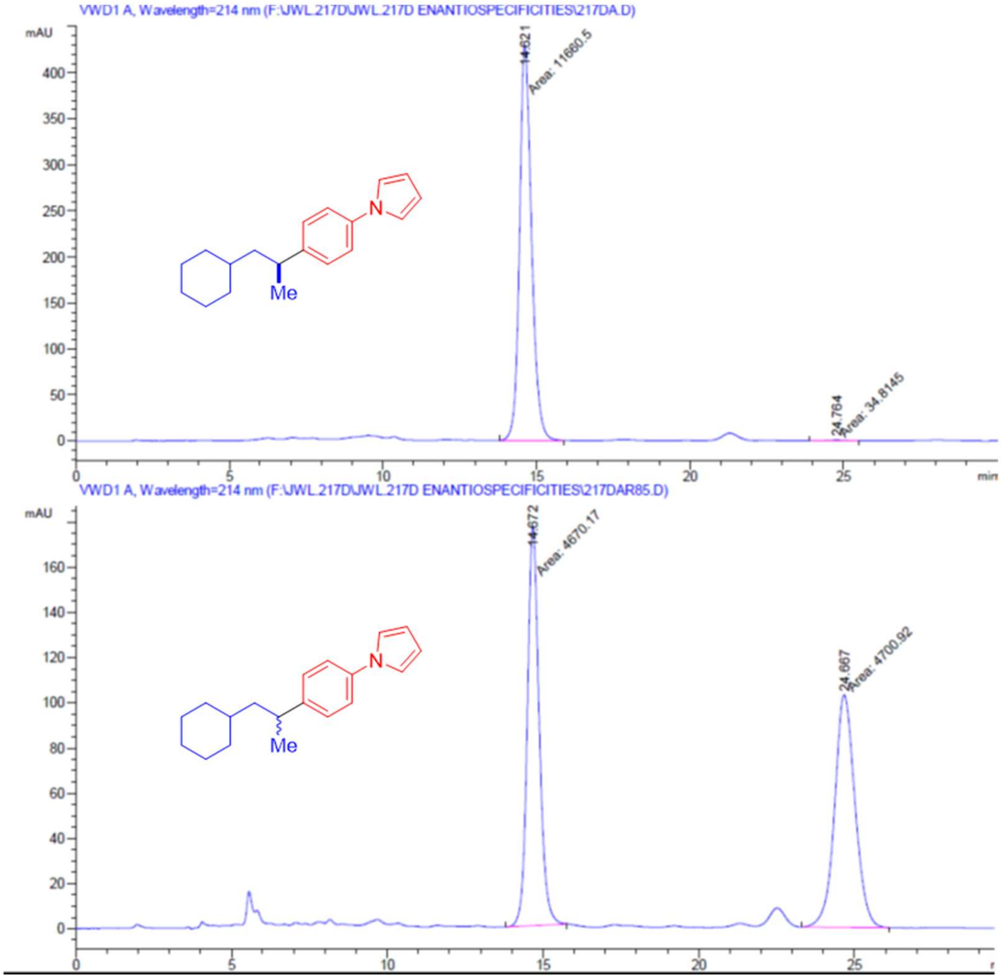
VWD1 A, Wavelength=214 nm (F:\JWL.217D\JWL.217D ENANTIOSPECIFICITIES\217DA.D)
N
Me
14.621
Area: 11660.5
24.764
Area: 34.8145
VWD1 A, Wavelength=214 nm (F:\JWL.217D\JWL.217D ENANTIOSPECIFICITIES\217DAR85.D)
N
Me
14.672
Area: 4670.17
24.667
Area: 4700.92

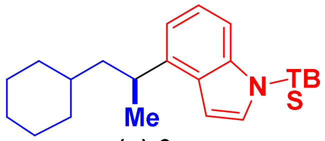

(+)-3v

Boronic acid ***(S)*-1d** (≥99:1 e.r.) was prepared in 67% yield by general procedure C and coupled to organohalide **2l** to give product **(+)-3v** by general procedure D. The branched/linear product ratio of the crude reaction was determined to be 250/1 by HPLC (Eclipse XDB-C8 column, isocratic 90/10 MeCN/H_2O, 1.2 mL/min, 215.4 nm absorbance). Branched = 12.0 minutes; linear = 13.4 minutes. The product was isolated in 68% yield (24.7 mg) by purification with reverse phase flash chromatography (20/1 MeCN/H_2O, R_f = 0.19). The e.r. of the purified product was determined to be 99.43 : 0.57 (>99% es) using chiral HPLC (AD-RH chiral column, isocratic 72/28 MeCN/H_2O, 0.4 mL/min, 214 nm absorance). Major = 22.7 minutes; minor = 21.2 minutes.

A duplicate run of the reaction gave a branched/linear product ratio of 226/1, isolated yield of 75%, and enantiospecificity of >99%.

^{1}H NMR (500 MHz, $CDCl_3$) δ 7.35 (d, J = 8.4 Hz, 1H), 7.16 (d, J = 3.3 Hz, 1H), 7.10 (dd, J = 8.3, 7.3 Hz, 1H), 6.95 (d, J = 7.2 Hz, 1H), 6.68 (d, *J* = 3.0 Hz, 1H), 3.29 (sext, J = 7.0 Hz, 1H), 1.81 (d, *J* = 12.8 Hz, 1H), 1.77-1.58 (m, 5H), 1.48 (ddd, J = 13.8, 8.1, 6.2 Hz, 1H), 1.32 (d, J = 6.9 Hz, 3H), 1.35-1.27 (m, 1H), 1.23-1.09 (m, 3H), 0.94 (s, 9H), 0.97-0.86 (m, 2H), 0.60 (s, 3H), 0.59 (s, 3H).

^{13}C NMR (126 MHz, $CDCl_3$) δ 141.17, 140.64, 130.29, 121.59, 116.24, 111.56, 103.20, 45.82, 35.46, 34.19, 33.69, 33.37, 26.92, 26.53, 26.50, 26.47, 21.49, 19.65, -3.75.

$[\alpha]^{20}_D$ = +26.3 (c 1.0, $CHCl_3$)

HRMS (EI^+) Calculated for $C_{23}H_{38}NSi$ $(M+H)^+$: 356.2774, Found: 356.2769

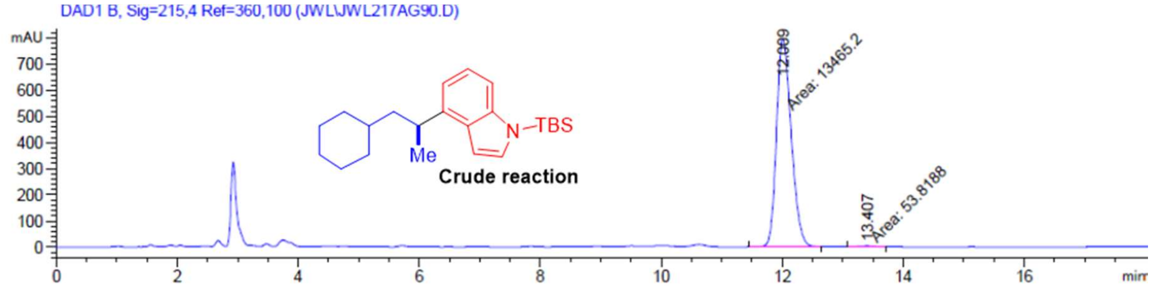
DAD1 B, Sig=215,4 Ref=360,100 (JWL\JWL217AG90.D)
mAU
12.009
Area: 13465.2
13.407
Area: 53.8188
N–TBS
Me
Crude reaction
min

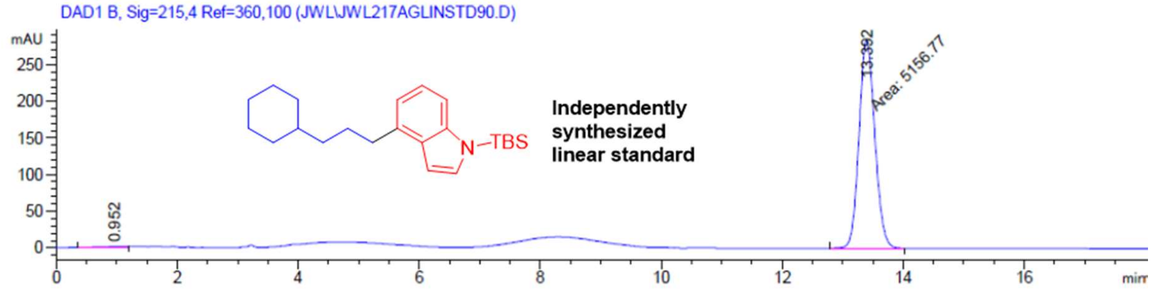
DAD1 B, Sig=215,4 Ref=360,100 (JWL\JWL217AGLINSTD90.D)
mAU
0.952
13.392
Area: 5156.77
N–TBS
Independently
synthesized
linear standard
min

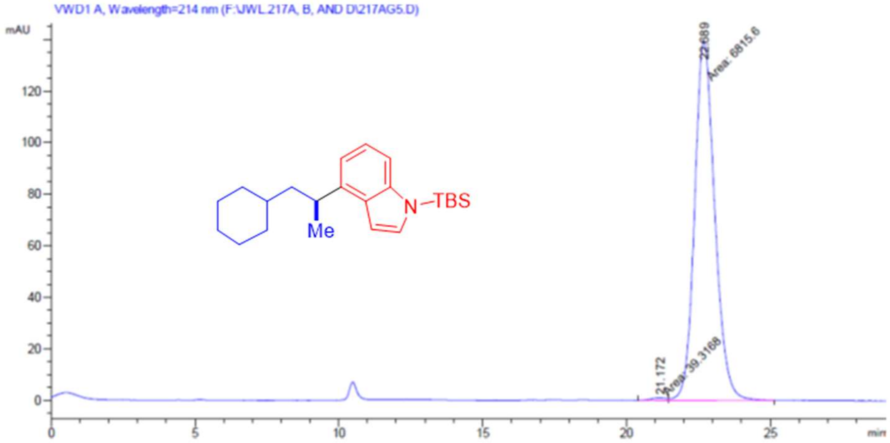
VWD1 A, Wavelength=214 nm (F:\JWL.217A, B, AND D\217AG5.D)
mAU
22.689
Area: 6815.6
21.172
Area: 39.3168
N–TBS
Me
min

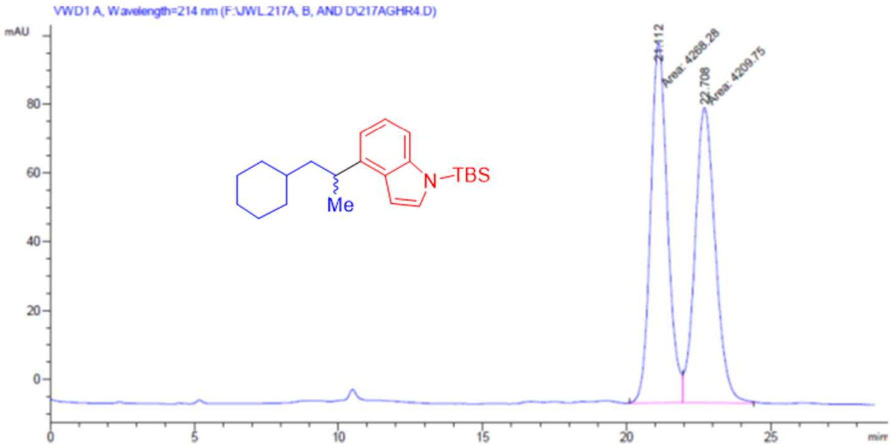
VWD1 A, Wavelength=214 nm (F:\JWL.217A, B, AND D\217AGHR4.D)
mAU
21.112
Area: 4268.28
22.708
Area: 4209.75
N–TBS
Me
min

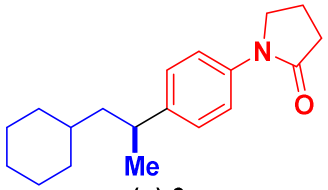

(+)-3w

Boronic acid ***(S)*-1d** (≥99:1 e.r.) was prepared in 65% yield by general procedure C and coupled to organohalide **2m** to give product **(+)-3w** by general procedure D. The branched/linear product ratio of the crude reaction was determined to be 307/1 by HPLC (Eclipse XDB-C8 column, isocratic 65/35 MeCN/H_2O, 1.2 mL/min, 250 nm absorbance). Branched = 9.6 minutes; linear = 10.9 minutes. The product was isolated in 74% yield (21.4 mg) by purification with reverse phase flash chromatography (7/1 MeCN/H_2O, R_f = 0.22) followed by normal phase flash chromatography (2/1 Hex/EtOAc, R_f = 0.24). The e.r. of the purified product was determined to be 99.56 : 0.44 (>99% es) using chiral HPLC (AD-RH chiral column, isocratic 45/45/10 MeCN/MeOH/H_2O, 0.5 mL/min, 214 nm absorance). Major = 24.2 minutes; minor = 21.3 minutes.

A duplicate run of the reaction gave a branched/linear product ratio of 254/1, isolated yield of 61%, and enantiospecificity of >99%.

^{1}H NMR (500 MHz, $CDCl_3$) δ 7.50 (d, J = 8.6 Hz, 2H), 7.17 (d, J = 8.5 Hz, 2H), 3.86 (t, J = 7.0 Hz, 2H), 2.80 (app. sext, *J* = 8.3 Hz, 1H), 2.60 (t, J = 8.1 Hz, 2H), 2.15 (app. quint, *J* = 7.5 Hz, 2H), 1.75 (d, *J* = 13.0 Hz, 1H), 1.67-1.55 (m, 4H), 1.48 (ddd, J = 14.2, 8.7, 5.9 Hz, 1H), 1.36 (ddd, J = 13.8, 7.9, 6.5 Hz, 1H), 1.17 (d, J = 6.9 Hz, 3H), 1.16-1.06 (m, 4H), 0.91-0.79 (m, 2H),

^{13}C NMR (126 MHz, $CDCl_3$) δ 174.17, 144.56, 137.19, 127.41, 120.19, 49.05, 46.40, 36.26, 35.10, 33.88, 33.32, 32.84, 26.82, 26.38, 26.35, 23.12, 18.25.

$[\alpha]^{20}_D$ = +38.2 (c 1.0, $CHCl_3$)

HRMS (EI^+) Calculated for $C_{19}H_{27}ON$ $(M)^+$: 285.20927, Found: 285.20903

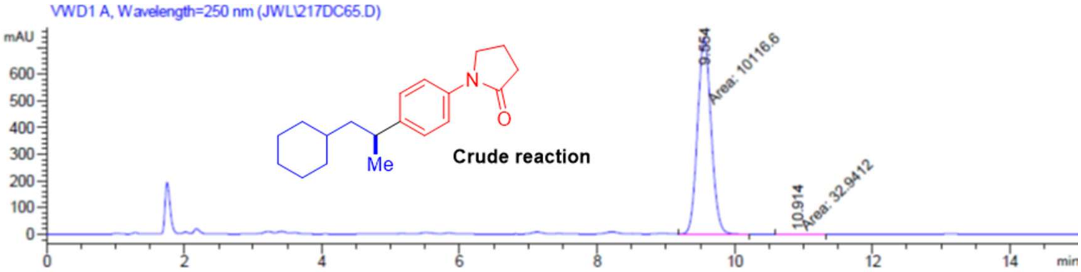
VWD1 A, Wavelength=250 nm (JWL\217DC65.D)
mAU
Crude reaction
Me
9.554
Area: 10116.6
10.914
Area: 32.9412
min

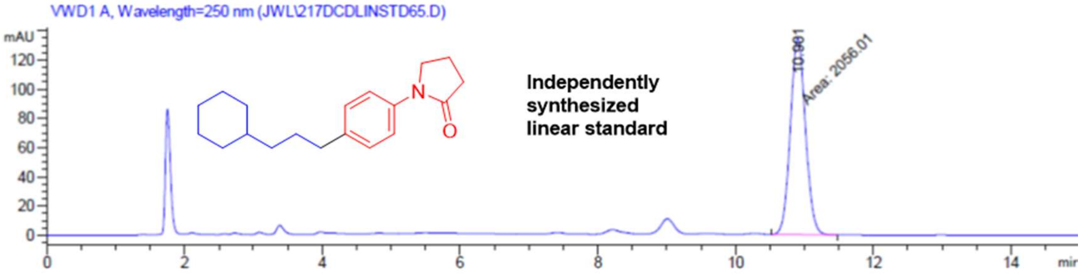
VWD1 A, Wavelength=250 nm (JWL\217DCDLINSTD65.D)
mAU
Independently synthesized linear standard
10.901
Area: 2056.01
min

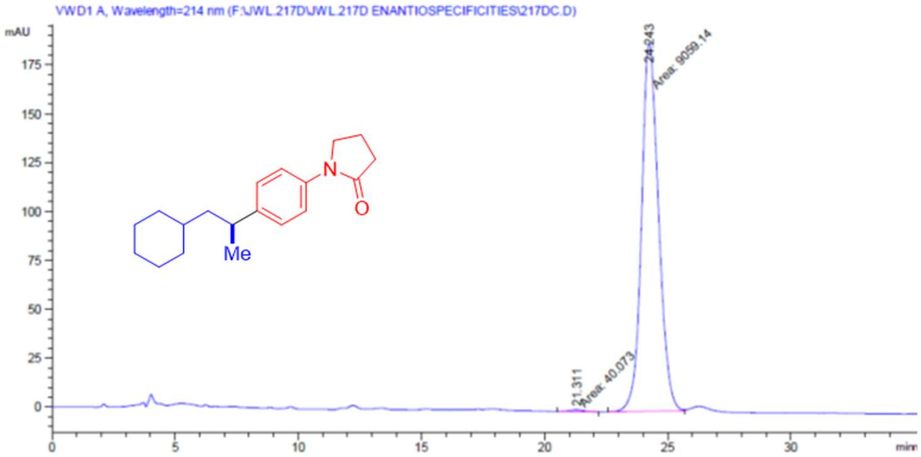
VWD1 A, Wavelength=214 nm (F:\JWL.217D\JWL.217D ENANTIOSPECIFICITIES\217DC.D)
mAU
Me
21.311
Area: 40.073
24.243
Area: 9059.14

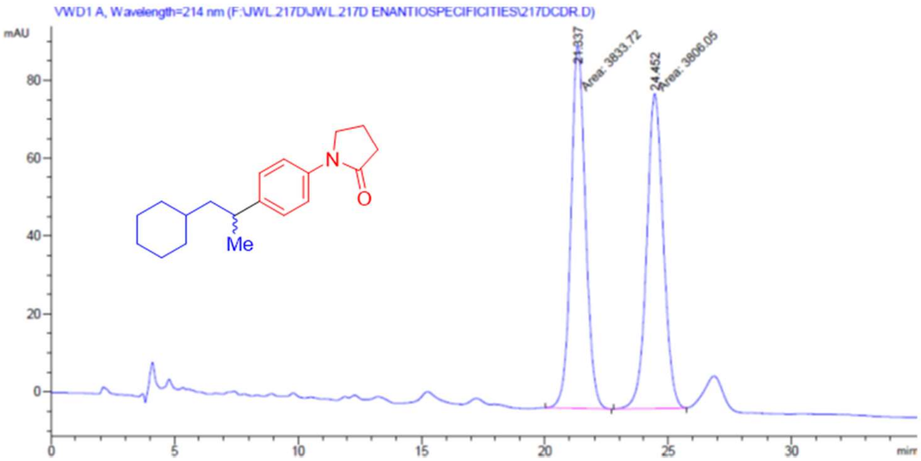
VWD1 A, Wavelength=214 nm (F:\JWL.217D\JWL.217D ENANTIOSPECIFICITIES\217DCDR.D)
mAU
Me
21.337
Area: 3833.72
24.452
Area: 3806.05

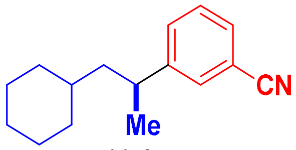

(-)-3x

Boronic acid ***(S)*-1d** (≥99:1 e.r.) was prepared in 66% yield by general procedure C and coupled to organohalide **2n** to give product **(-)-3x** by general procedure D. The branched/linear product ratio of the crude reaction was determined to be 44/1 by HPLC (Eclipse XDB-C8 column, isocratic 70/30 MeCN/H_2O, 1.2 mL/min, 215.4 nm absorbance). Branched = 10.8 minutes; linear = 12.3 minutes. The product was isolated in 56% yield (12.9 mg) by purification with reverse phase flash chromatography (6/1 MeCN/H_2O, R_f = 0.26) followed by normal phase flash chromatography (2/1 Hex/DCM, R_f = 0.26). The e.r. of the purified product was determined to be 99.15 : 0.85 (>99% es) using chiral HPLC (AD-RH chiral column, isocratic 75/25 MeCN/H_2O, 0.5 mL/min, 214.4 nm absorance). Major = 10.0 minutes; minor = 7.4 minutes.

A duplicate run of the reaction gave a branched/linear product ratio of 42/1, isolated yield of 55%, and enantiospecificity of >99%.

^{1}H NMR (500 MHz, $CDCl_3$) δ 7.49-7.45 (m, 2H), 7.43-7.36 (m, 2H), 2.86 (sext, *J* = 8.1 Hz, 1H), 1.75-1.58 (m, 5H), 1.47 (ddd, *J* = 14.4, 8.4, 6.3 Hz, 1H), 1.40 (dt, *J* = 13.9, 7.2 Hz, 1H), 1.20 (d, *J* = 6.9 Hz, 3H), 1.17-1.03 (m, 4H), 0.92-0.82 (m, 2H).

^{13}C NMR (126 MHz, $CDCl_3$) δ 149.63, 131.83, 130.81, 129.72, 129.24, 119.39, 112.43, 46.10, 36.65, 35.15, 33.70, 33.35, 26.71, 26.32, 22.68.

$[\alpha]^{20}_D$ = -74.4 (c 1.0, $CHCl_3$)

HRMS (EI^+) Calculated for $C_{16}H_{21}N$ $(M)^+$: 227.16740, Found: 227.16723

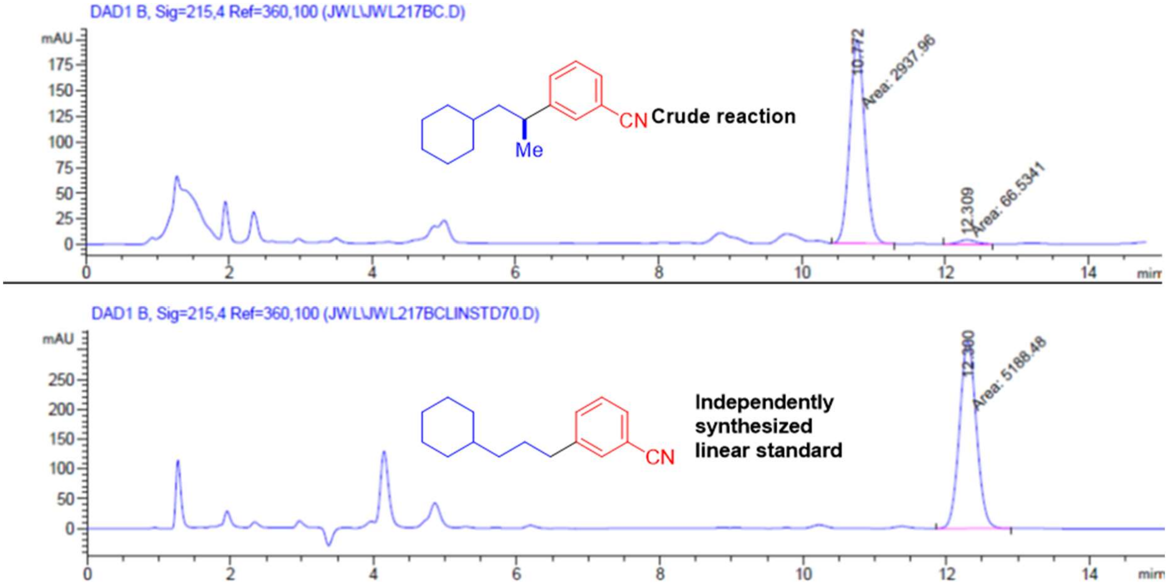
DAD1 B, Sig=215,4 Ref=360,100 (JWL\JWL217BC.D)
mAU
Me
CN
Crude reaction
10.772
Area: 2937.96
12.309
Area: 66.5341
DAD1 B, Sig=215,4 Ref=360,100 (JWL\JWL217BCLINSTD70.D)
CN
Independently
synthesized
linear standard
12.300
Area: 5168.48

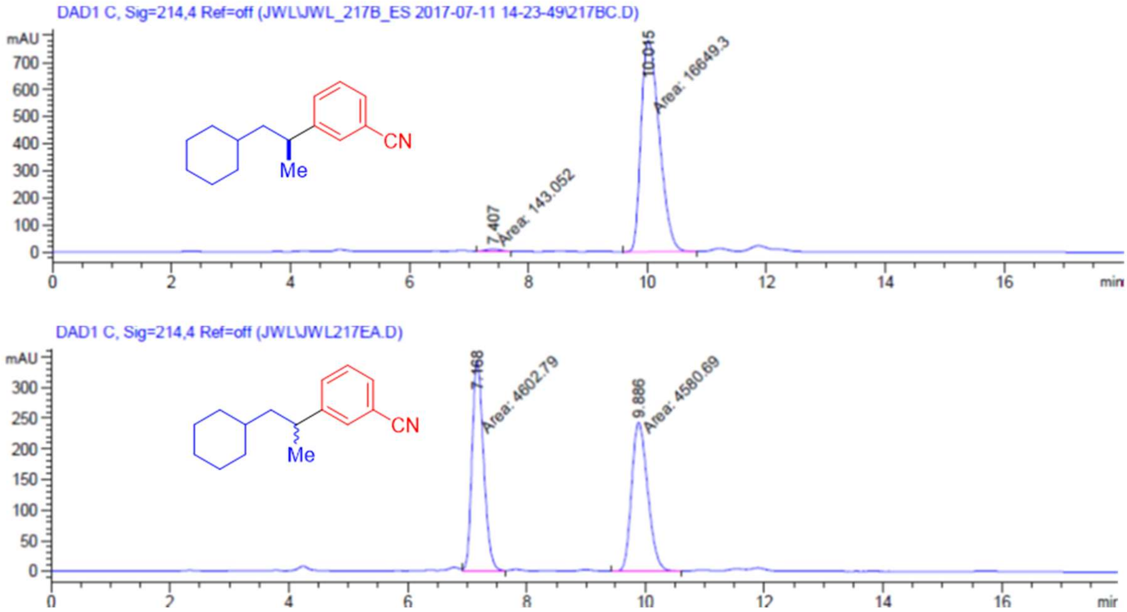
DAD1 C, Sig=214,4 Ref=off (JWL\JWL_217B_ES 2017-07-11 14-23-49\217BC.D)
mAU
CN
Me
7.407
Area: 143.052
10.015
Area: 16649.3
DAD1 C, Sig=214,4 Ref=off (JWL\JWL217EA.D)
CN
Me
7.168
Area: 4602.79
9.886
Area: 4580.69

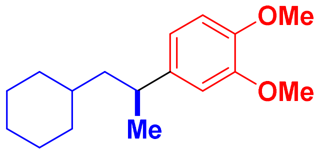

(+)-3y

Boronic acid **(*S*)-1d** (≥99:1 e.r.) was prepared in 67% yield by general procedure C and coupled to organohalide **2o** to give product **(+)-3y** by general procedure D. The branched/linear product ratio of the crude reaction was determined to be 266/1 by HPLC (Eclipse XDB-C8 column, isocratic 70/30 MeCN/H_2O, 1.2 mL/min, 215.4 nm absorbance). Branched = 9.8 minutes; linear = 11.1 minutes. The product was isolated in 62% yield (15.0 mg) by purification with reverse phase flash chromatography (6/1 MeCN/H_2O). The e.r. of the purified product was determined to be 99.52 : 0.48 (>99% es) using chiral HPLC (OD-H chiral column, isocratic 99.8/0.2 Hex/IPA, 2.0 mL/min, 210 nm absorance). Major = 7.3 minutes; minor = 8.8 minutes.

A duplicate run of the reaction gave a branched/linear product ratio of 256/1, isolated yield of 55%, and enantiospecificity of >99%.

^{1}H NMR (500 MHz, $CDCl_3$) δ 6.80 (d, *J* = 8.1 Hz, 1H), 6.74-6.69 (m, 2H), 3.88 (s, 3H), 3.86 (s, 3H), 2.76 (sext, *J* = 7.8 Hz, 1H), 1.75 (d, *J* = 12.7 Hz, 1H), 1.68-1.56 (m, 4H), 1.45 (ddd, *J* = 14.3, 8.3, 6.3 Hz, 1H), 1.36 (dt, *J* = 13.8, 7.1 Hz, 1H), 1.18 (d, *J* = 6.9 Hz, 3H), 1.18-1.09 (m, 4H), 0.91-0.81 (m, 2H).

^{13}C NMR (126 MHz, $CDCl_3$) δ 148.88, 147.12, 141.07, 118.81, 111.25, 110.42, 56.01, 55.98, 46.65, 36.43, 35.24, 33.80, 33.54, 26.85, 26.43, 23.09.

$[\alpha]^{20}_D$ = +19.8 (c 1.2, $CHCl_3$)

HRMS (EI^+) Calculated for $C_{17}H_{26}O_2$ $(M)^+$: 262.19328, Found: 262.19324

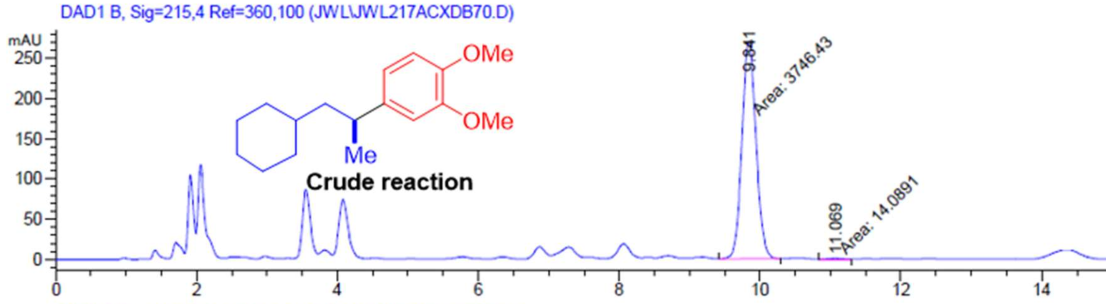
DAD1 B, Sig=215,4 Ref=360,100 (JWL\JWL217ACXDB70.D)
OMe
OMe
Me
Crude reaction
9.841
Area: 3746.43
11.069
Area: 14.0891

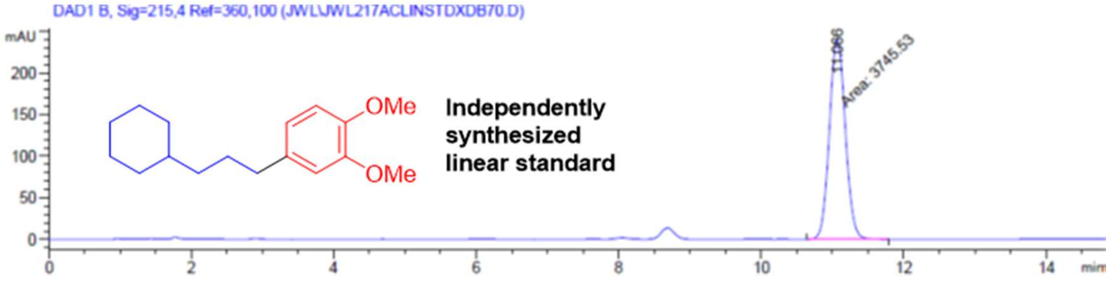
DAD1 B, Sig=215,4 Ref=360,100 (JWL\JWL217ACLINSTDXDB70.D)
OMe
OMe
Independently
synthesized
linear standard
11.066
Area: 3745.53

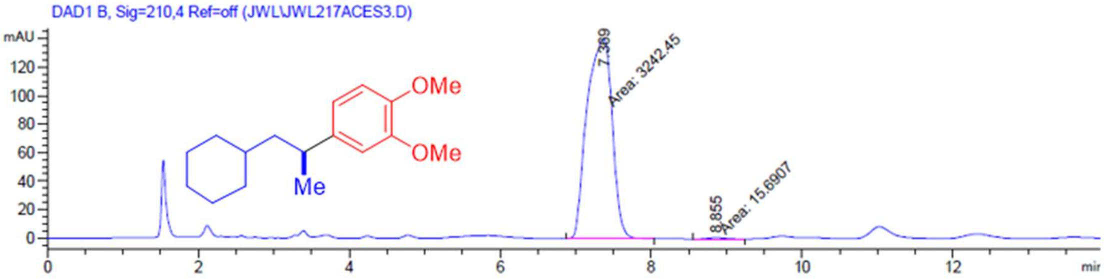
DAD1 B, Sig=210,4 Ref=off (JWL\JWL217ACES3.D)
OMe
OMe
Me
7.369
Area: 3242.45
8.855
Area: 15.6907

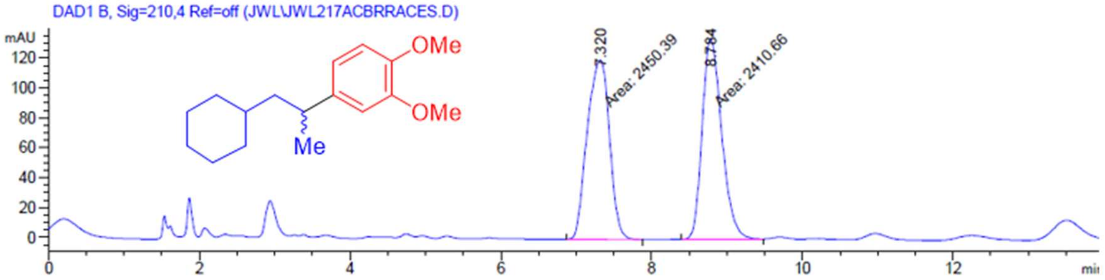
DAD1 B, Sig=210,4 Ref=off (JWL\JWL217ACBRRACES.D)
OMe
OMe
Me
7.320
Area: 2450.39
8.784
Area: 2410.66

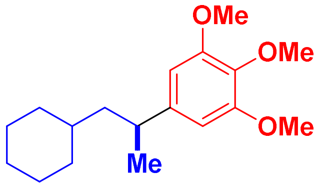

(+)-3z

Boronic acid **(*S*)-1d** (≥99:1 e.r.) was prepared in 67% yield by general procedure C and coupled to organohalide **2p** to give product **(+)-3z** by general procedure D. The branched/linear product ratio of the crude reaction was determined to be 100/1 by HPLC (Eclipse XDB-C8 column, isocratic 65/35 MeCN/H_2O, 1.2 mL/min, 215.4 nm absorbance). Branched = 13.0 minutes; linear = 15.1 minutes. The product was isolated in 71% yield (20.3 mg) by purification with reverse phase flash chromatography (5/1 MeCN/H_2O, R_f = 0.24). The e.r. of the purified product was determined to be 99.57 : 0.43 (>99% es) using chiral HPLC (OD-H chiral column, isocratic 99.5/0.5 Hex/IPA, 2.0 mL/min, 214.4 nm absorance). Major = 4.69 minutes; minor = 5.43 minutes.

A duplicate run of the reaction gave a branched/linear product ratio of 128/1, isolated yield of 74%, and enantiospecificity of >99%.

^{1}H NMR (500 MHz, $CDCl_3$) δ 6.39 (s, 2H), 3.86 (s, 6H), 3.83 (s, 3H), 2.75 (sext, *J* = 7.1 Hz, 1H), 1.76 (d, *J* = 13.1 Hz, 1H), 1.70-1.58 (m, 4H), 1.45 (dt, *J* = 14.2, 7.2 Hz, 1H), 1.37 (dt, *J* = 13.8, 7.1 Hz, 1H), 1.19 (d, J = 6.8 Hz, 3H), 1.22-1.11 (m, 4H), 0.92-0.82 (m, 2H).

^{13}C NMR (126 MHz, $CDCl_3$) δ 153.15, 144.26, 136.07, 103.91, 60.97, 56.21, 46.56, 37.23, 35.23, 33.68, 26.83, 26.42, 22.80.

$[\alpha]^{20}_D$ = +17.1 (c 1.8, $CHCl_3$)

HRMS (EI^+) Calculated for $C_{18}H_{28}O_3$ $(M)^+$: 292.20385, Found: 292.20380

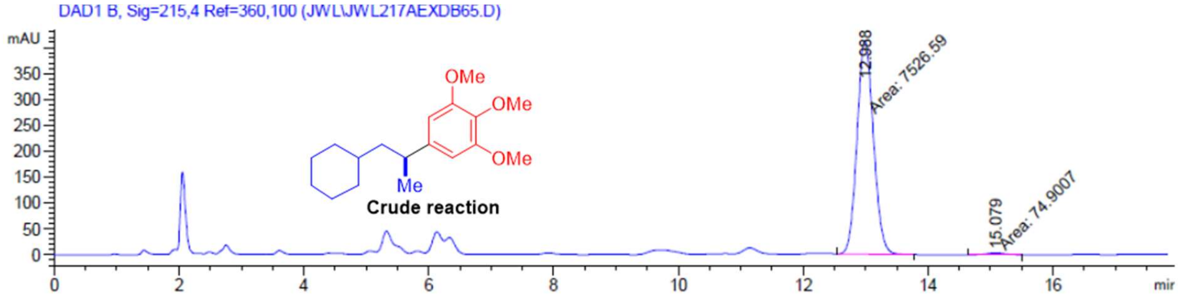
DAD1 B, Sig=215,4 Ref=360,100 (JWL\JWL217AEXDB65.D)
mAU
12.988
Area: 7526.59
15.079
Area: 74.9007
OMe
OMe
OMe
Me
Crude reaction
min

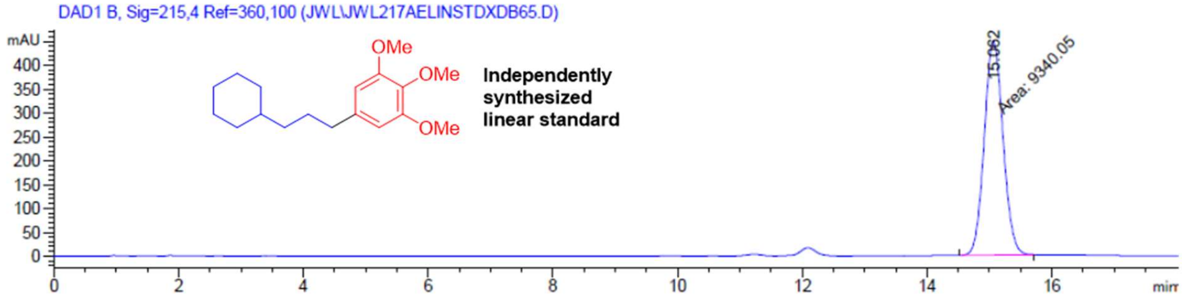
DAD1 B, Sig=215,4 Ref=360,100 (JWL\JWL217AELINSTDXDB65.D)
mAU
15.062
Area: 9340.05
OMe
OMe
OMe
Independently
synthesized
linear standard
min

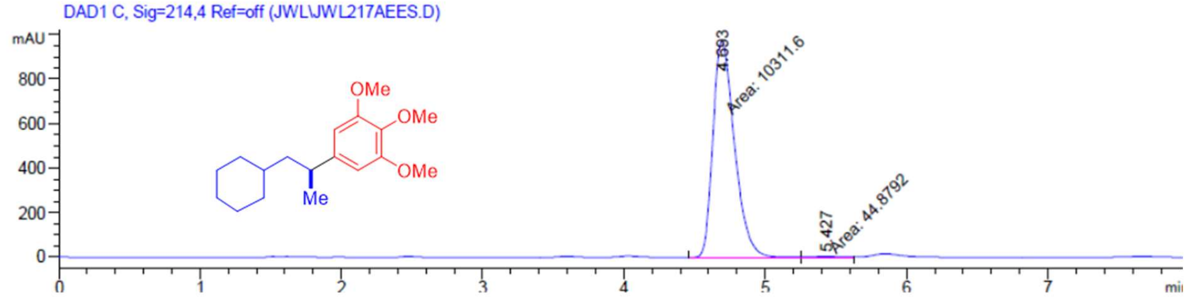
DAD1 C, Sig=214,4 Ref=off (JWL\JWL217AEES.D)
mAU
4.693
Area: 10311.6
5.427
Area: 44.8792
OMe
OMe
OMe
Me

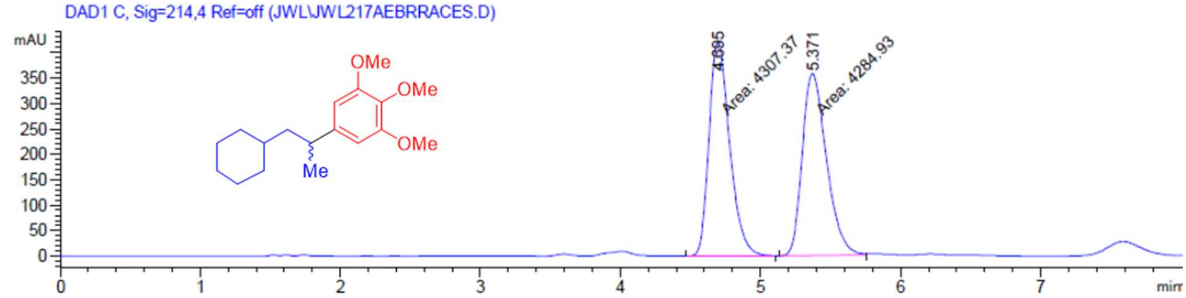
DAD1 C, Sig=214,4 Ref=off (JWL\JWL217AEBRRACES.D)
mAU
4.695
Area: 4307.37
5.371
Area: 4284.93
OMe
OMe
OMe
Me
min

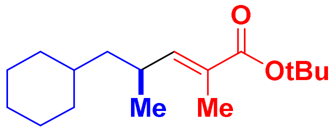

(+)-3aa

Boronic acid **(*S*)-1d** (≥99:1 e.r.) was prepared in 65% yield by general procedure C and coupled to organohalide **2q** to give product **(+)-3aa** by general procedure D. The branched/linear product ratio of the crude reaction was determined to be 888/1 by HPLC (Eclipse XDB-C8 column, isocratic 85/15 MeCN/H_2O, 1.2 mL/min, 214.4 nm absorbance). Branched = 8.2 minutes; linear = 8.9 minutes. The product was isolated in 64% yield (17.9 mg) by purification with normal phase phase flash chromatography (3/1 Hex/DCM, R_f = 0.25). The e.r. of the purified product 50/50 MeCN/H_2O, 0.5 mL/min, 214.4 nm absorance). Major = 24.3 minutes; minor = 26.5 minutes.

A duplicate run of the reaction gave a branched/linear product ratio of 1303/1, isolated yield of 68%, and enantiospecificity of 100%.

^{1}H NMR (500 MHz, $CDCl_3$) δ 6.41 (dd, *J* = 10.0, 1.5 Hz, 1H), 2.57 (dq, *J* = 10.3, 6.7 Hz, 1H), 1.78 (d, *J* = 1.4 Hz, 3H), 1.71-1.60 (m, 5H), 1.49 (s, 9H), 1.26-1.07 (m, 6H), 0.95 (d, *J* = 6.6 Hz, 3H), 0.90-0.78 (m, 2H).

^{13}C NMR (126 MHz, $CDCl_3$) δ 168.07, 147.62, 127.37, 107.71, 80.01, 44.93, 35.34, 33.85, 33.59, 30.34, 28.31, 26.81, 26.43, 26.42, 20.38, 12.63.

$[\alpha]^{20}_D$ = +28.7 (c 1.0, $CHCl_3$)

HRMS (ESI+) Calculated for $C_{17}H_{30}O_2Na$ $(M+Na)^+$: 289.2144, Found: 289.2136

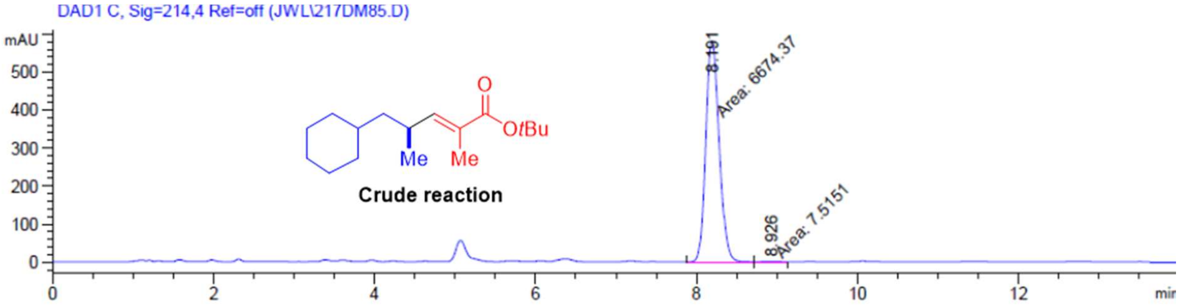
DAD1 C, Sig=214,4 Ref=off (JWL\217DM85.D)
mAU
OtBu
Me
Me
Crude reaction
8.191
Area: 6674.37
8.926
Area: 7.5151
min

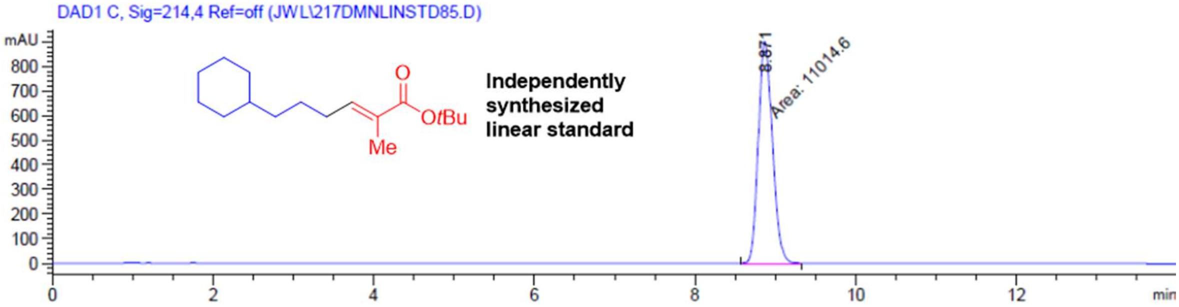
DAD1 C, Sig=214,4 Ref=off (JWL\217DMNLINSTD85.D)
mAU
OtBu
Me
Independently synthesized linear standard
8.871
Area: 11014.6
min

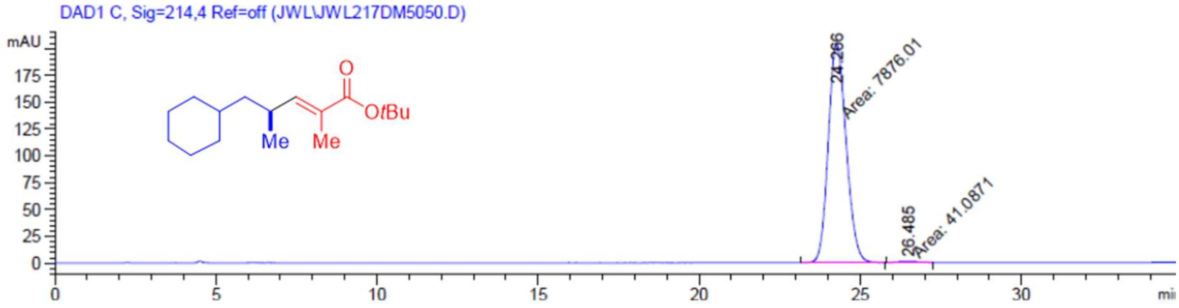
DAD1 C, Sig=214,4 Ref=off (JWL\JWL217DM5050.D)
mAU
OtBu
Me
Me
24.266
Area: 7876.01
26.485
Area: 41.0871

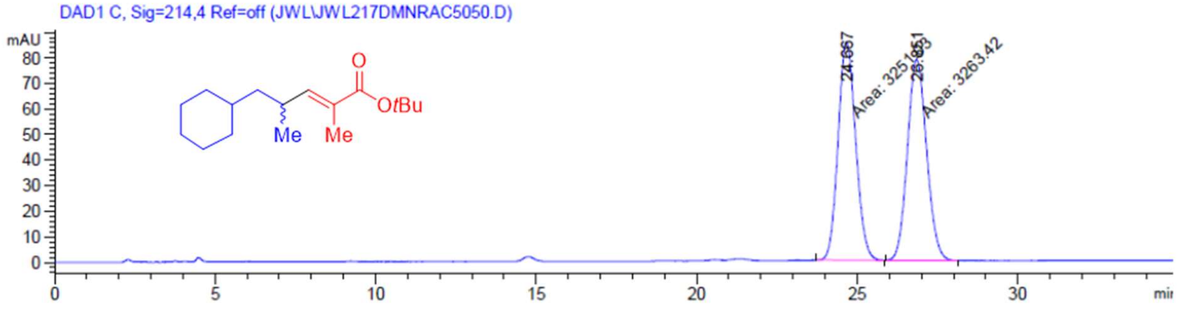
DAD1 C, Sig=214,4 Ref=off (JWL\JWL217DMNRAC5050.D)
mAU
OtBu
Me
Me
24.667
Area: 3263.42

Synthesis and Characterization of Xylarinic Acid B

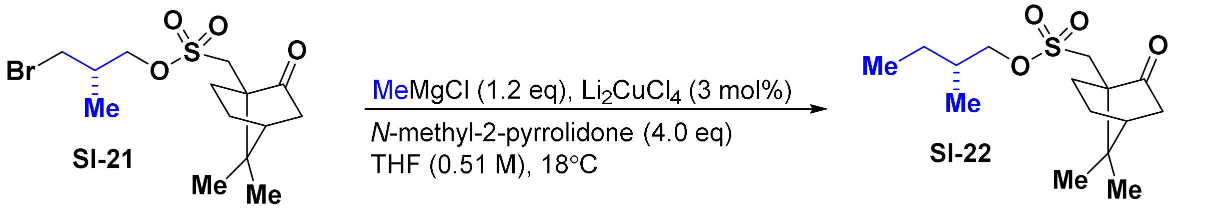

SI-22. A flame-dried Schlenk flask was charged with alkyl bromide **SI-21**[20] (5.47 g, 14.89 mmol, 1.0 eq). The flask was sealed, evacuated, and vac-filled with nitrogen three times. Anhydrous THF (14 mL), Li_2CuCl_4 (0.1M in THF, 4.5 mL, 0.45 mmol, 3 mol%), and *N*-methyl-2-pyrrolidone (5.7 mL, 59.23 mmol, 4.0 eq) was added via syringe under N_2. The reaction was cooled in an 18 °C water bath, and MeMgCl solution (1.7M in THF, 10.5 mL, 17.85 mmol, 1.2 eq) was added dropwise over 10 minutes. The reaction was stirred for 1 hour and 45 minutes. The reaction was then cooled to 0 °C, then 1N HCl (50 mL) was added in one portion. The mixture was stirred until the solids dissolved and transferred to a separatory funnel with Et_2O (10 mL). After phase separation, the aqueous layer was extracted with Et_2O (2x30 mL). The organics were washed with H_2O (30 mL), brine, dried over $MgSO_4$, filtered, and concentrated *in vacuo*. The crude product was purified on a silica gel column (isocratic 15% EtOAc in hexanes). Mixed fractions were combined for a second silica gel column purification, affording the product **SI-22** as a colorless oil (2.92 g, 9.65 mmol, 65% yield).

^{1}H NMR (500 MHz, $CDCl_3$) δ 4.17 (dd, *J* = 9.4, 5.8 Hz, 1H), 4.05 (dd, *J* = 9.4, 6.6 Hz, 1H), 3.60 (d, *J* = 15.1 Hz, 1H), 2.98 (d, *J* = 15.1 Hz, 1H), 2.50 (ddd, *J* = 14.7, 11.8, 3.9 Hz, 1H), 2.39 (m, 1H), 2.12, (t, *J* = 4.5 Hz, 1H), 2.06 (m, 1H), 1.95 (d, *J* = 18.5 Hz, 1H), 1.79 (m, 1H), 1.65 (ddd, *J* = 14.1, 9.4, 4.7 Hz, 1H), 1.52 – 1.41 (m, 2H), 1.22 (dquint, *J* = 13.5, 7.6 Hz, 1H), 1.12 (s, 3H), 0.97 (d, *J* = 6.8 Hz, 3H), 0.92 (t, *J* = 7.5 Hz, 3H), 0.88 (s, 3H).

^{13}C NMR (126 MHz, $CDCl_3$) δ 214.71, 74.90, 58.08, 48.09, 46.64, 42.88, 42.66, 34.75, 27.02, 25.67, 25.01, 19.98, 19.84, 16.19, 11.23.

HRMS (ESI$^+$) Calculated for $C_{15}H_{26}O_4NaS$ $(M+Na)^+$: 325.1449, Found: 325.1450

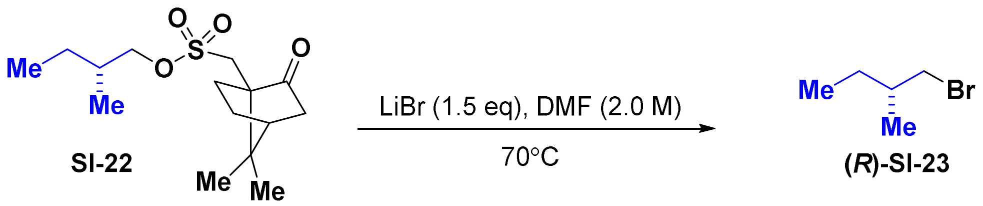

***(R)*-SI-23.** A solution of **SI-22** (4.60 g, 15.2 mmol) in anhydrous DMF (6.5 mL) was prepared in a dry, stir bar-equipped 40 mL vial. This solution was then added to LiBr (1.98 g, 22.8 mmol, 1.5 eq) in another dry, stir bar-equipped 40 mL vial via syringe under nitrogen, rinsing with DMF (2x0.5 mL, total DMF = 7.5 mL = 2.0 Molar) for quantitative transfer. The reaction was stirred at 70 °C for one hour, then cooled to room temperature and transferred to two 50 mL centrifuge tubes, rinsing with DMF (3×0.5 mL). To each centrifuge tube was added H_2O (40 mL). After shaking, the phases were separated by centrifugation (3000 rpm for three minutes, then 4000 rpm for three minutes). In both centrifuge tubes, the aqueous

layer on top was removed by pipet and fresh H_2O was added to the 40 mL mark. After mixing, the phases were again separated by centrifugation by the same procedure. The oil at the bottom of each centrifuge tube was removed and passed through a short pad of a mixture consisting of celite and Na_2SO_4 in a Pasteur pipette into a tared 7 mL vial, giving the **(*R*)-SI-23** as a clear colorless oil (1.76 g, 11.6 mmol, 76% yield). NMR matches that reported in the literature.[21]

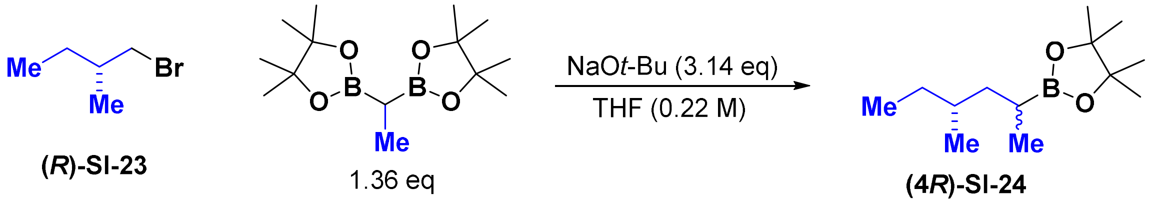

(4*R*)-SI-24. The pinacol boronic ester was synthesized by a modified literature procedure. In an argon-filled glovebox, a stir bar-equipped 250 mL Schlenk flask was charged with NaO*t*-Bu (2.87 g, 29.8 mmol, 3.15 eq). A separate 40 mL vial was charged with (R)-1-bromo-2-methylbutane **(*R*)-SI-23** (1.43 g, 9.47 mmol, 1.00 eq) and Et(Bpin)$_2$[17] (3.63 g, 12.9 mmol, 1.36 eq). The Schlenk flask was sealed with a rubber septum, and the 40 mL was sealed with a septa cap. Both vessels were brought out of the glovebox and into a fume hood and connected to nitrogen lines. Anhydrous THF (29 mL) was added to the Schlenk flask, resulting in a cloudy, light yellow suspension. The mixture of alkyl bromide and Et(Bpin)$_2$ was then added (at room temperature, dropwise over five minutes) to the Schlenk flask, using additional THF (2x7 mL, total THF = 43 mL = 0.22 Molar) for quantitative transfer. The reaction was stirred efficiently at room temperature overnight.

The next day, the reaction was diluted with Et_2O (75 mL) and filtered through a pad of silica gel in a coarse glass frit. The filtrate was concentrated by rotary evaporation, giving a colorless oil of low viscosity. The crude product was purified by normal phase column chromatography (6 cm diameter, 250 mL silica gel, isocratic 4/1 Hex/DCM), giving **(4*R*)-SI-24** as a colorless oil (1.34 g, 5.94 mmol, 63% yield).

^{1}H NMR (500 MHz, $CDCl_3$) δ 1.47 (ddd, *J* = 12.9, 8.9, 5.4 Hz, 1H), 1.40 – 1.27 (m, 4H), 1.22 (d, *J* = 2.2 Hz, 26H), 1.15 – 1.00 (m, 5H), 0.93 (dd, *J* = 8.9, 7.3 Hz, 6H), 0.89 – 0.78 (m, 12H).

^{13}C NMR (126 MHz, $CDCl_3$) δ 82.72, 82.70, 40.53, 39.87, 33.62, 32.96, 29.68, 29.51, 24.74, 24.70, 24.69, 24.65, 19.45, 18.88, 16.08, 15.35, 11.43.

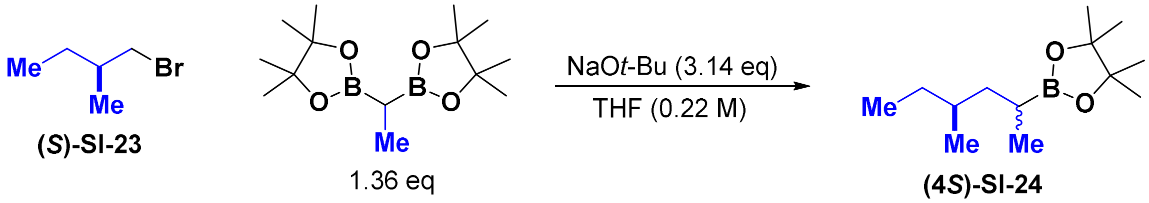

(4*S*)-SI-24. was synthesized from **(*S*)-SI-23**[22] by the same procedure as **(4*R*)-SI-24**, giving the product as a colorless oil (1.869 g, 8.22 mmol, 62% yield).

NMR of **(4*S*)-SI-24** matches that of **(4*R*)-SI-24**.

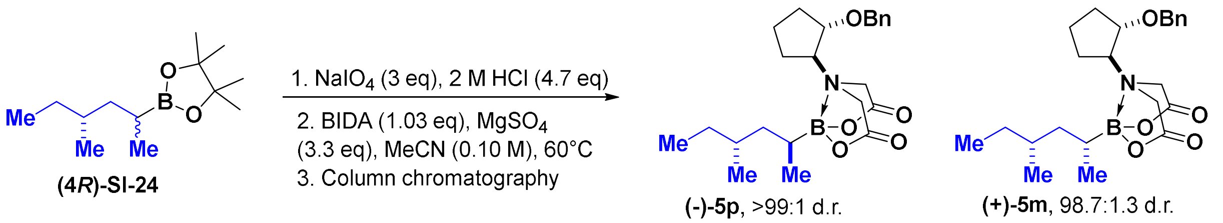

BIDA Boronates 5p and 5m. To a stir bar-equipped 300 mL round bottom flask was added the pinacol boronic ester **(4*R*)-SI-24** (1.442 g, 6.37 mmol, 1.00 eq) followed by THF (43 mL, 0.15 M), H_2O (6.1 mL), $NaIO_4$ (6.02 g, 28.1 mmol, 3.00 eq), and 1 Molar HCl (4.3 mL, 4.3 mmol, 0.68 eq). After five hours, monitoring of the reaction by TLC (3/1 Hex/EtOAc) still showed a substantial amount of the starting boronic ester. Additional 5.5 Molar HCl (4.6 mL, 25.3 mmol, 4.0 eq) was added to increase the total amount of HCl (2 Molar, 4.7 eq). The reaction was stirred for another hour and 15 minutes, at which point TLC indicated full consumption of the pinacol boronic ester. The stir bar was removed, additional H_2O (15 mL) was added, and THF was removed by rotary evaporation. The reaction was diluted with additional H_2O (15 mL) and extracted with methyl *tert*-butyl ether (2x40 mL). Combined organics were washed repeatedly with H_2O (10x35 mL) to remove any remaining oxidant and then once with brine (35 mL). After drying with $MgSO_4$ and filtering through a glass frit into a 500 mL round bottom flask, the solution was partially concentrated (remaining volume of 5-10 mL) and then the solvent was switched to anhydrous MeCN (64 mL, 0.10 Molar). BIDA (2.01 g, 6.54 mmol, 1.03 eq) and $MgSO_4$ (2.55 g, 21.2 mmol, 3.3 eq) were added, along with a stir bar. The reaction was sealed with a rubber septum and connected to a nitrogen line before stirring overnight at 60°C. The reaction mixture was filtered through a plug of silica gel, rinsing with EtOAc. The filtrate was concentrated by rotary evaporation, giving a red foam (2.06 g, 4.97 mmol, 78% crude yield). This material was subjected to normal phase column chromatography (15/1 MTBE/EtOAc or 1/1 Hex/EtOAc). Mixed fractions were repurified until the d.r. of both diastereomers was ≥99:1 as determined by a sequence of stereospecific oxidation to the alcohol and derivatization to the para-nitrobenzoate ester (described below). The higher R_f diastereomer (**5p**, *R*,*S*; 0.549 g, 1.32 mmol, 21% yield) was isolated as a white powder, and so was the lower R_f diastereomer (**5m**, *R*,*R*; 0.498 g, 1.20 mmol, 19% yield).

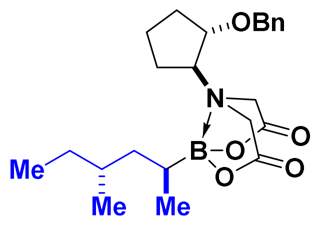

(-)-5p, Higher Rf, >99:1 d.r.

^{1}H NMR (500 MHz, acetone-*d6*) δ 7.43 – 7.39 (m, 2H), 7.38 – 7.34 (m, 2H), 7.33 – 7.27 (m, 1H), 4.65 (d, *J* = 11.3 Hz, 1H), 4.59 (d, *J* = 11.2 Hz, 1H), 4.41 (m, 1H), 4.16 – 4.04 (m, 3H), 3.95 (d, *J* = 17.5 Hz, 1H), 3.79 (td, *J* = 8.7, 6.4 Hz, 1H), 2.28 (m, 1H), 2.19 (m, 1H), 1.88-1.77 (m 3H), 1.62 (m, 1H), 1.45 (dtd, J = 13.5, 6.6, 3.4 Hz, 1H), 1.33 – 1.13 (m, 4H), 1.08 (dqd, *J* = 10.1, 6.6, 3.3 Hz, 1H), 0.87 (t, *J* = 7.4 Hz, 3H), 0.81 (dd, J = 6.7, 1.4 Hz, 6H).

^{13}C NMR (126 MHz, acetone-*d6*) δ 169.70, 167.90, 138.63, 128.85, 128.49, 128.20, 80.79, 72.88, 71.98, 60.70, 56.80, 39.57, 31.94, 31.30, 30.17, 27.01, 21.74, 18.32, 14.44, 11.65.

^{11}B NMR (128 MHz, acetone-*d6*) δ 14.48.

$[\alpha]^{20}_{D}$ = -0.5 (c = 1.0, acetone)

HRMS (ESI+) Calculated for $C_{23}H_{35}BNO_5$ $(M+H)^+$: 416.2608, Found: 416.2608

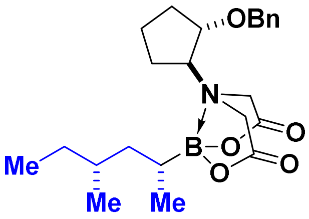

(+)-5m, Lower Rf, 98.7:1.3 d.r.

^{1}H NMR (500 MHz, acetone-*d6*) δ 7.41 – 7.34 (m, 4H), 7.30 (m, 1H), 4.65 (d, *J* = 11.5 Hz, 1H), 4.58 (d, *J* = 11.5 Hz, 1H), 4.37 (dt, *J* = 7.5, 5.0 Hz, 1H), 4.13 (dd, J = 17.2, 2.8 Hz, 2H), 4.05 (d, *J* = 16.9 Hz, 1H), 3.97 (d, *J* = 17.4 Hz, 1H), 3.77 (td, *J* = 8.7, 6.0 Hz, 1H), 2.28 (m, 1H), 2.14 (m, 1H), 1.88 – 1.74 (m, 3H), 1.68 (ddt, *J* = 13.3, 9.2, 7.9 Hz, 1H), 1.52 (ddtd, *J* = 18.2, 14.7, 7.0, 3.6 Hz, 2H), 1.22 (m, 1H), 1.12 – 1.02 (m, 2H), 1.01 – 0.93 (m, 1H), 0.93 (d, *J* = 6.4 Hz, 3H), 0.86 – 0.81 (m, 6H).

^{13}C NMR (126 MHz, acetone-*d6*) δ 169.30, 168.02, 138.68, 128.82, 128.17, 128.13, 80.63, 73.12, 71.79, 60.28, 57.54, 39.87, 31.80, 30.34, 27.56, 27.24, 21.90, 20.54, 14.82, 11.02.

^{11}B NMR (128 MHz, acetone-*d*6) δ 13.67.

$[\alpha]^{20}_{D}$ = +55.6 (c 1.0, acetone)

HRMS (ESI+) Calculated for $C_{23}H_{35}BNO_5$ $(M+H)^+$: 416.2608, Found: 416.2608

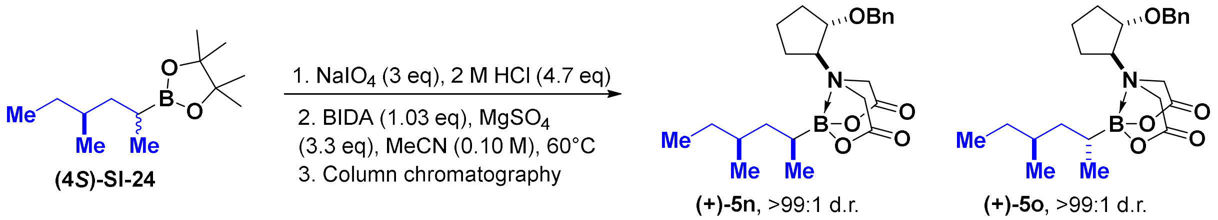

(4*S*)-SI-24 **(+)-5n**, >99:1 d.r. **(+)-5o**, >99:1 d.r.

BIDA Boronates 5n and 5o. To a stir bar-equipped 300 mL round bottom flask was added the pinacol boronic ester **(4*S*)-SI-24** (1.859 g, 8.22 mmol, 1.00 eq) followed by THF (55 mL, 0.15 M), $NaIO_4$ (5.28 g, 24.7 mmol, 3.00 eq), and 2 Molar HCl (19 mL, 38 mmol, 6.0 eq). Once the reaction was complete by TLC (3/1 Hex/EtOAc), the stir bar was removed, additional H_2O (20 mL) was added, and THF was removed by rotary evaporation. The reaction was diluted with more H_2O (20 mL) and extracted with methyl *tert*-butyl ether (2x50 mL). Combined organics were washed repeatedly with H_2O (10x45 mL) to remove any remaining oxidant and then once with brine (50 mL). After drying with $MgSO_4$ and filtering through a

glass frit into a 500 mL round bottom flask, the solution was partially concentrated (remaining volume of 5-10 mL) and then the solvent was switched to anhydrous MeCN (84 mL, 0.10 Molar). BIDA (2.53 g, 8.22 mmol, 1.00 eq) and $MgSO_4$ (3.28 g, 27.3 mmol, 3.3 eq) were added, along with a stir bar. The reaction was sealed with a rubber septum and connected to a nitrogen line before stirring overnight at 60°C.

The next day, the reaction mixture was filtered through a plug of silica gel, rinsing with EtOAc. The filtrate was concentrated by rotary evaporation, giving a red foam. This material was subjected to normal phase column chromatography (gradient 1.2/1 Hex/EtOAc to 1/1.2 Hex/EtOAc). Mixed fractions were repurified until the d.r. of both diastereomers was ≥99:1 as determined by a sequence of stereospecific oxidation to the alcohol and derivatization to the para-nitrobenzoate ester (described below). The higher R_f diastereomer (**5n**, *S*,*S*; 0.8222 g, 1.98 mmol, 24% yield) was isolated as white powder, and so was the lower R_f diastereomer (**5o**, *S*,*R*; 1.2124 g, 2.92 mmol, 36% yield).

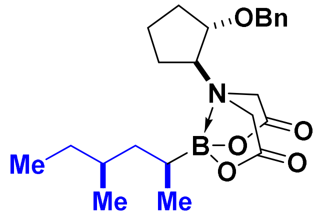

(+)-5n, Higher Rf, >99:1 d.r.

^{1}H NMR (500 MHz, $CDCl_3$) δ 7.43-7.35 (m, 3H), 7.31-7.28 (2H), 4.65 (d, *J* = 11.5 Hz, 1H), 4.38 (d, *J* = 11.5 Hz, 1H), 4.02 (d, *J* = 16.7 Hz, 1H), 3.91 (q, *J* = 6.8 Hz, 1H), 3.65 (m, 1H), 3.61 (d, *J* = 17.0 Hz, 1H), 3.44 (d, *J* = 16.6 Hz, 1H), 3.32 (d, *J* = 16.9 Hz, 1H), 2.21 (m, 1H), 2.08 (m, 1H), 1.88 (m, 1H), 1.82-1.69 (m, 2H), 1.50 (m, 3H), 1.40 (dqd, *J* = 14.8, 7.5, 3.2 Hz, 1H), 1.09 (app. quint, *J* = 9.1 Hz, 1H), 1.02-0.89 (m, 2H), 0.88-0.82 (m, 9H).

^{13}C NMR (126 MHz, $CDCl_3$) δ 169.02, 167.43, 136.44, 128.73, 128.43, 128.19, 79.27, 71.98, 71.92, 60.79, 56.05, 39.35, 31.16, 29.56, 27.55, 26.45, 21.36, 20.16, 14.86, 10.99.

^{11}B NMR (128 MHz, $CDCl_3$) δ 14.14.

$[\alpha]^{20}_D$ = +6.5 (c 1.09, $CDCl_3$)

HRMS (ESI+) Calculated for $C_{23}H_{33}BNO_5$ $(M-H)^+$: 414.2452, Found: 414.2448

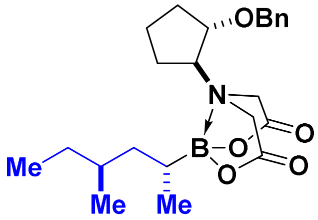

(+)-5o, Lower Rf, >99:1 d.r.

^1H NMR (500 MHz, $CDCl_3$) δ 7.43-7.35 (m, 3H), 7.31-7.28 (m, 2H), 4.66 (d, *J* = 11.7 Hz, 1H), 4.39 (d, *J* = 11.6 Hz, 1H), 4.04 (d, *J* = 16.7 Hz, 1H), 3.89 (q, *J* = 6.6 Hz, 1H), 3.65 (q, *J* = 8.1 Hz, 1H), 3.63 (dd, *J* = 17.0 Hz, 1H), 3.41 (d, *J* = 16.7 Hz, 1H), 3.33 (d, *J* = 16.9 Hz, 1H), 2.23 (m, 1H), 2.05 (m, 1H), 1.88-1.69 (m, 3H), 1.53-1.44 (m, 2H), 1.33-1.13 (m, 4H), 0.99-0.92 (m, 4H), 0.87 (t, *J* = 7.4 Hz, 3H), 0.83 (d, *J* = 6.5 Hz, 3H), 0.79 (m, 1H).

^{13}C NMR (126 MHz, $CDCl_3$) δ 168.96, 167.56, 136.62, 128.89, 128.58, 128.23, 79.17, 71.92, 61.00, 56.66, 38.96, 31.61, 31.19, 29.66, 26.75, 21.48, 18.54, 14.35, 11.83.

$[\alpha]^{20}_D$ = +32.4 (c 1.09, CDCl3)

HRMS (ESI+) Calculated for $C_{23}H_{34}BNO_5Na$ $(M+Na)^+$: 438.2428, Found: 438.2423

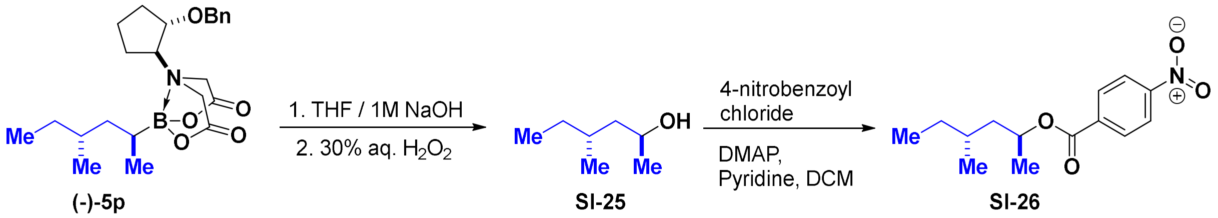

SI-26. BIDA boronate **(-)-5p** (10.4 mg, 0.025 mmol, 1.0 eq) was added to a stir bar-equipped 7 mL vial. THF (0.25 mL) and 1 M NaOH (0.25 mL, 10 eq) were added. The reaction was stirred at room temperature until full conversion to the boronic acid was observed by TLC (1:1 Hex/EtOAc, $KMnO_4$ stain). To the reaction was added 30% aqueous H_2O_2 (0.05 mL, 0.5 mmol, 20 eq), and the reaction was allowed to stir at room temperature until full conversion of the alcohol was observed by TLC (1:1 Hex/EtOAc, $KMnO_4$ stain). The reaction was diluted with Et_2O (15 mL) and quenched with saturated aqueous $Na_2S_2O_3$ (15 mL). After the organic layer was mixed and separated, it was washed with brine (15 mL), dried with Na_2SO_4, and concentrated under mild vacuum to afford (2*S*,4*R*)-4-methylhexan-2-ol (**SI-25**).

To a solution of the crude **SI-25** in anhydrous DCM (0.5 mL, 0.05 Molar) in a stir bar-equipped 7 mL vial was added anhydrous pyridine (7 µL, 7 mg, 0.09 mmol, 1.8 eq), 4-nitrobenzoyl chloride (13 mg, 0.07 mmol, 1.4 eq) and 4-(dimethylamino)pyridine (0.6 mg, 0.005 mmol, 10 mol%). The vial was capped and stirred at room temperature overnight. The next day, the reaction mixture was passed through a pad of $MgSO_4$ in a cotton-plugged glass pipet, and filtrate was concentrated *in vacuo*. The crude product was purified by normal phase column chromatography (1:1 Hex/DCM), giving the pure 4-nitrobenzoate ester **SI-26**.

The product was analyzed by reverse phase HPLC (AD-RH column, isocratic 62:38 MeCN/H_2O, 0.5 mL/min, 214.4 nm), giving a d.r. of 99.9:0.1.

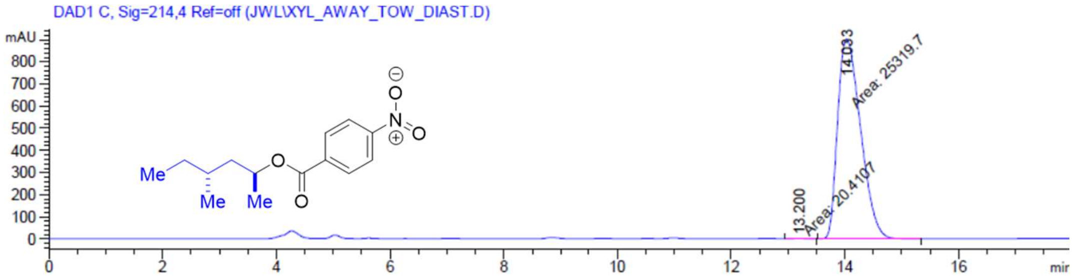

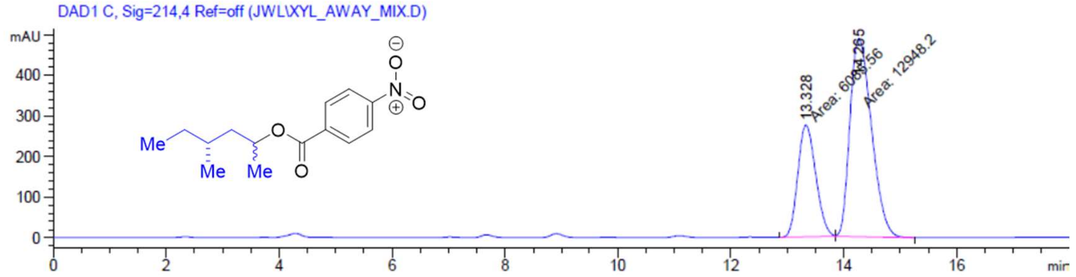

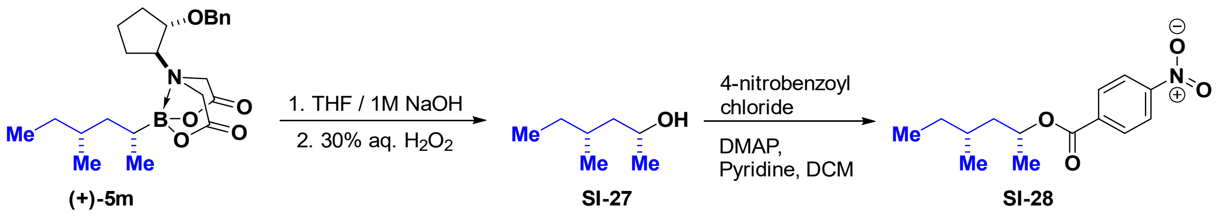

SI-28 was prepared from **(+)-5m** following the same procedure as for **SI-26**. Using the same HPLC conditions as for **SI-26**, the d.r. was determined to be 98.7:1.3.

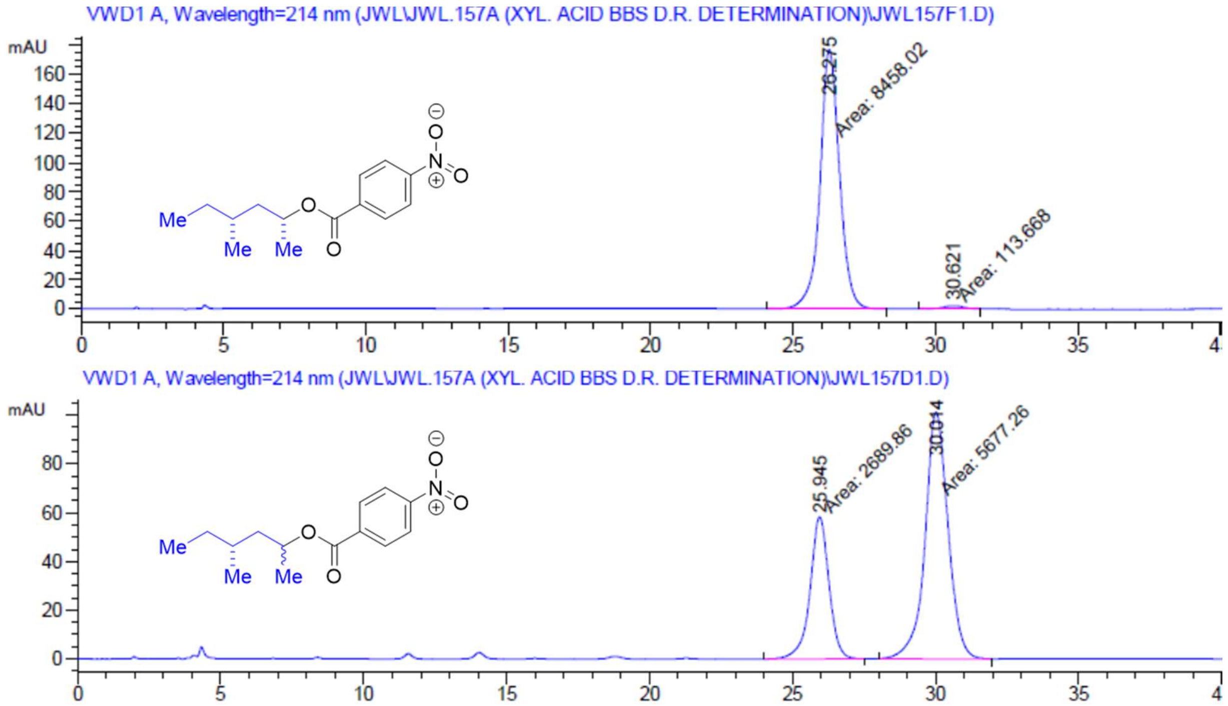

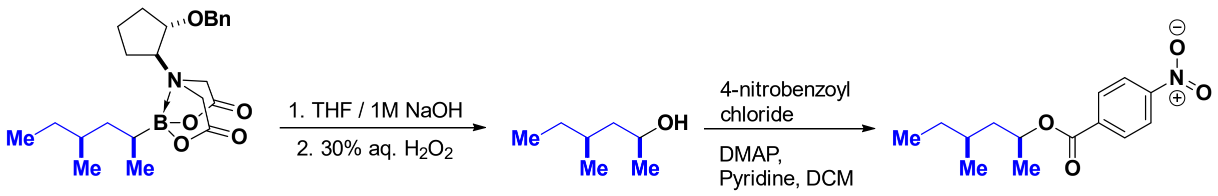

SI-30 was prepared from **(+)-5n** following the same procedure as for **SI-26**. Using the same HPLC conditions as for **SI-26**, the d.r. was determined to be 99.9:0.1.

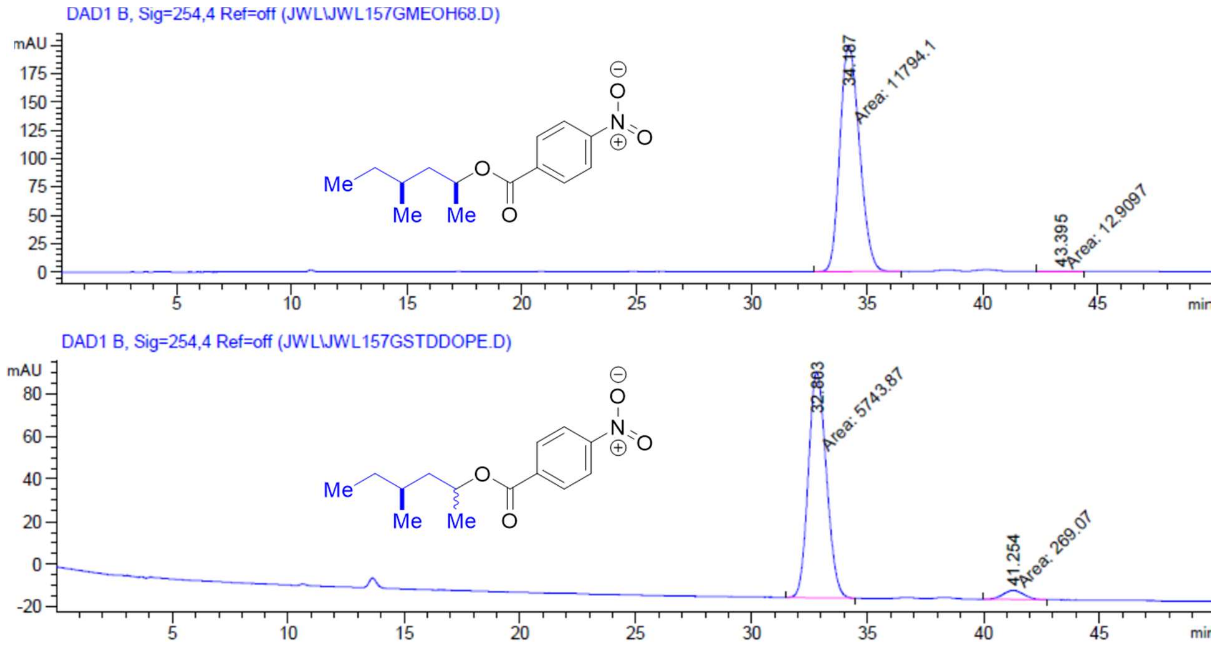

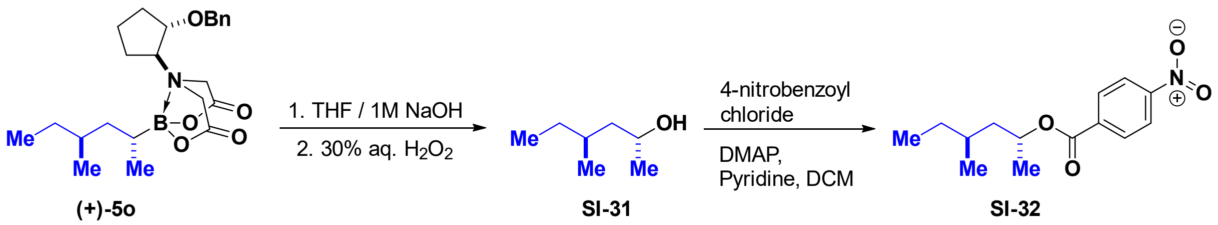

SI-32 was prepared following the same procedure as for **SI-26**. Using the same HPLC conditions as for **SI-26**, the d.r. was determined to be 99.3:0.7.

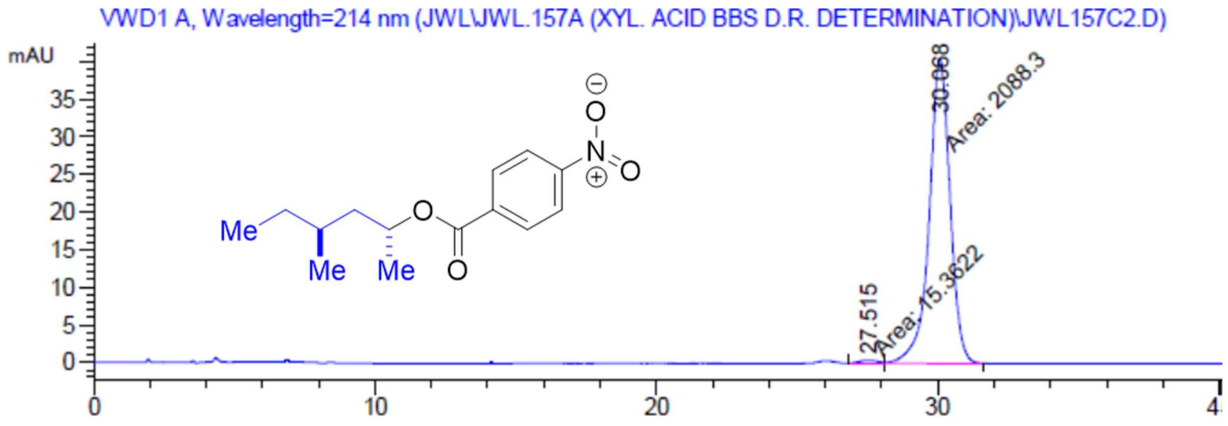

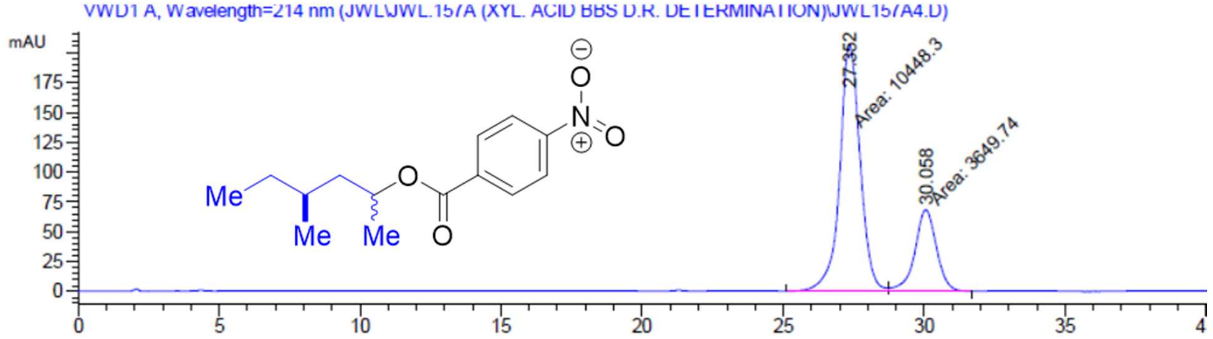

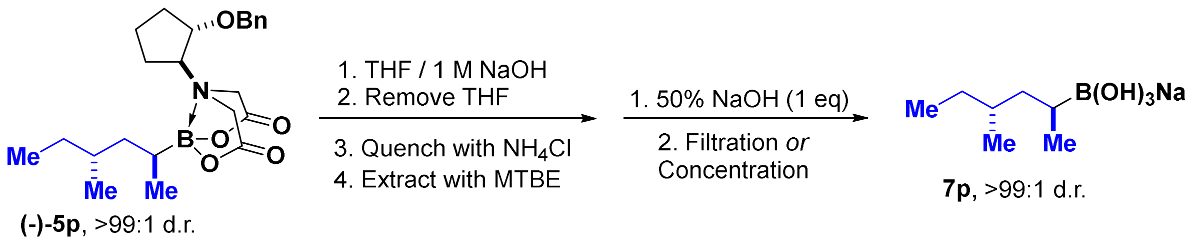

(-)-5p, >99:1 d.r.; **7p**, >99:1 d.r.

Sodium alkyltrihydroxyborate 7p was made from BIDA boronate **(-)-5p** in quantitative yield as a white solid using general procedure B by directly concentrating the suspension without filtration.

^{1}H NMR (500 MHz, CD_3OD) 1.38 (dq, J = 12.8, 6.2 Hz, 1H), 1.26 (ddd, J = 13.5, 7.6, 5.9 Hz, 1H), 1.21 – 1.10 (m, 3H), 0.88 (t, J = 7.4 Hz, 3H), 0.81 (d, J = 6.5 Hz, 3H), 0.78 (d, J = 7.1 Hz, 3H), 0.66 (d, J = 10.7 Hz, 1H).

^{13}C NMR (126 MHz, CD_3OD) δ 41.78, 33.89, 32.73, 19.00, 16.08, 12.26.

^{11}B NMR (128 MHz, CD_3OD) δ 7.47

$[\alpha]^{20}_D$ = -32.8 (c 1.03, CD_3OD)

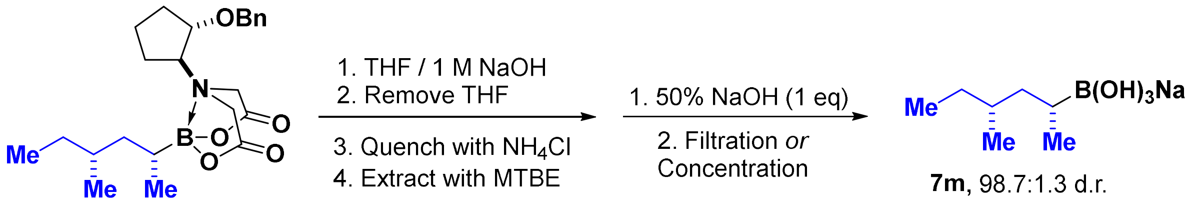

(+)-5m, 98.7:1.3 d.r.; **7m**, 98.7:1.3 d.r.

Sodium alkyltrihydroxyborate 7m was made from BIDA boronate **(+)-5m** in quantitative yield as a white solid using general procedure B by directly concentrating the suspension without filtration.

^{1}H NMR (500 MHz, CD_3OD) 1.54 (m, 1H), 1.45-1.32 (m, 2H), 1.02 – 0.88 (m, 2H), 0.88-0.83 (m, 6H), 0.80 (d, J = 7.1 Hz, 3H), 0.68 (br s, 1H).

^{13}C NMR (126 MHz, CD_3OD) δ 42.60, 34.06, 28.99, 21.36, 16.59, 11.91.

^{11}B NMR (128 MHz, CD_3OD) δ 7.74

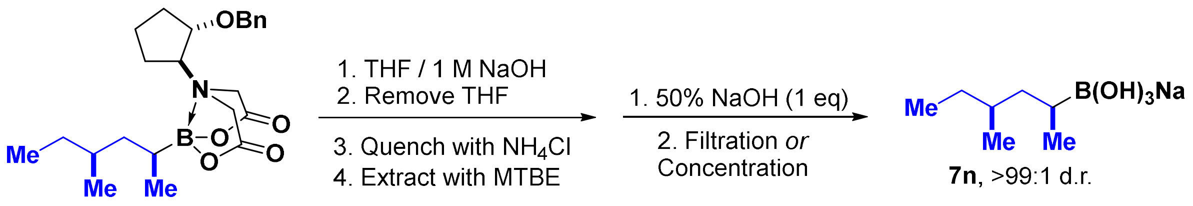

(+)-5n, >99:1 d.r.

7n, >99:1 d.r.

Sodium alkyltrihydroxyborate 7n was made from BIDA boronate **(+)-5n** in quantitative yield as a white solid using general procedure B by directly concentrating the suspension without filtration. ^{1}H NMR matches that of **7m**.

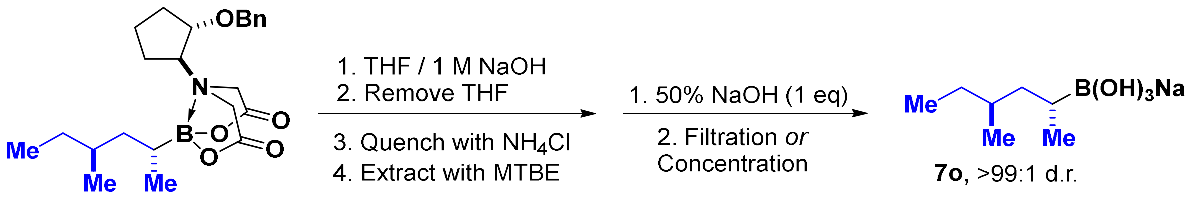

(+)-5o, >99:1 d.r.

7o, >99:1 d.r.

Sodium alkyltrihydroxyborate 7o was made from BIDA boronate **(+)-5o** in quantitative yield as a white solid using general procedure B by directly concentrating the suspension without filtration. NMR matches that of **7p**.

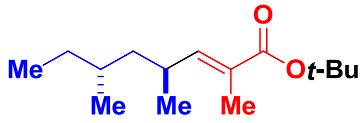

(+)-3ab

Boronic acid **1p** (99.9:0.1 e.r.) was prepared in 73% yield by general procedure C and coupled to organohalide **2p** to give product **(+)-3ab** by general procedure D. The branched/linear product ratio of the crude reaction was determined to be 396/1 by HPLC (Eclipse XDB-C8 column, isocratic 75/25 MeCN/H_2O, 1.2 mL/min, 214.4 nm absorbance). Branched = 10.8 minutes; linear = 11.7 minutes. The product was isolated in 69% yield (16.6 mg) by purification with normal phase flash chromatography (2/1 Hex/DCM). The d.r. of the purified product was determined by a sequence of deprotection and derivatization to the corresponding phenyl amide (**SI-33**) to be 99.6:0.4 (99.4%DS).

A duplicate run of the reaction gave a branched/linear product ratio of 516/1, isolated yield of 65%, and diastereospecificity of 99.4%.

^{1}H NMR (500 MHz, $CDCl_3$) δ 6.43 (dq, *J* = 9.8, 1.4 Hz, 1H), 2.55 (dquint, *J* = 10.0, 6.7 Hz, 1H), 1.78 (d, *J* = 1.5 Hz, 3H), 1.48 (s, 9H), 1.38 – 1.24 (m, 3H), 1.16 – 1.04 (m, 2H), 0.95 (d, *J* = 6.6 Hz, 3H), 0.88 – 0.80 (m, 6H).

^{13}C NMR (126 MHz, $CDCl_3$) δ 168.07, 147.64, 127.21, 80.01, 43.96, 32.05, 30.85, 29.50, 28.30, 19.94, 19.51, 12.60, 11.35.

[▯]$^{22}_{D}$ = +13.3 (c. 0.43, $CDCl_3$)

HRMS (ESI+) Calculated for $C_{15}H_{28}O_2Na$ $(M+Na)^+$: 263.1987, Found: 263.1980

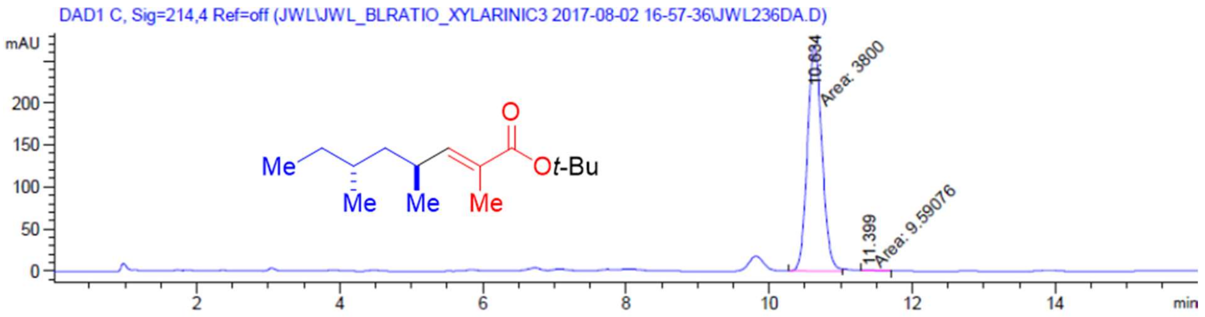

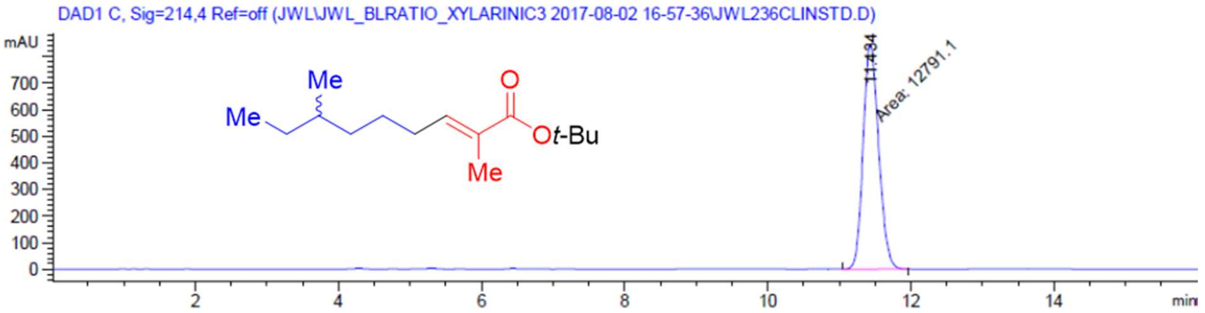

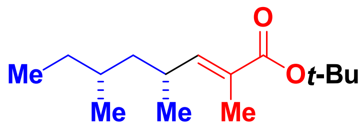

(-)-3ac

Boronic acid **1m** (98.7:1.3 e.r.) was prepared in 56% yield by general procedure C and coupled to organohalide **2p** to give product **(-)-3ac** by general procedure D. The branched/linear product ratio of the crude reaction was determined to be 402/1 by HPLC (Eclipse XDB-C8 column, isocratic 75/25 MeCN/H_2O, 1.2 mL/min, 214.4 nm absorbance). Branched = 11.0 minutes; linear = 11.9 minutes. The product was isolated in 57% yield (14.0 mg) by purification with normal phase flash chromatography (2/1 Hex/DCM). The d.r. of the purified product was determined by a sequence of deprotection and derivatization to the corresponding phenyl amide (**SI-34**) to be 98.0:2.0 (98.6%DS).

A duplicate run of the reaction gave a branched/linear product ratio of 381/1, isolated yield of 57%, and diastereospecificity of 98.5%.

^{1}H NMR (500 MHz, $CDCl_3$) δ 6.39 (dq, *J* = 10.2, 1.5 Hz, 1H), 2.57 (dddd, *J* = 16.2, 11.8, 8.2, 5.6 Hz, 1H), 1.79 (d, *J* = 1.5 Hz, 3H), 1.48 (s, 9H), 1.37 – 1.20 (m, 3H), 1.17 – 1.06 (m, 2H), 0.97 (d, *J* = 6.6 Hz, 3H), 0.85 (t, *J* = 7.4 Hz, 3H), 0.82 (d, *J* = 6.4 Hz, 3H).

^{13}C NMR (126 MHz, $CDCl_3$) δ 168.02, 147.41, 127.59, 80.00, 44.34, 32.33, 30.95, 30.09, 28.30, 20.72, 19.26, 12.67, 11.35.

[▯]$^{22}{}_{D}$ = -39.1 (c. 0.99, $CDCl_3$)

HRMS (ESI+) Calculated for $C_{15}H_{28}O_2Na$ $(M+Na)^+$: 263.1987, Found: 263.1978

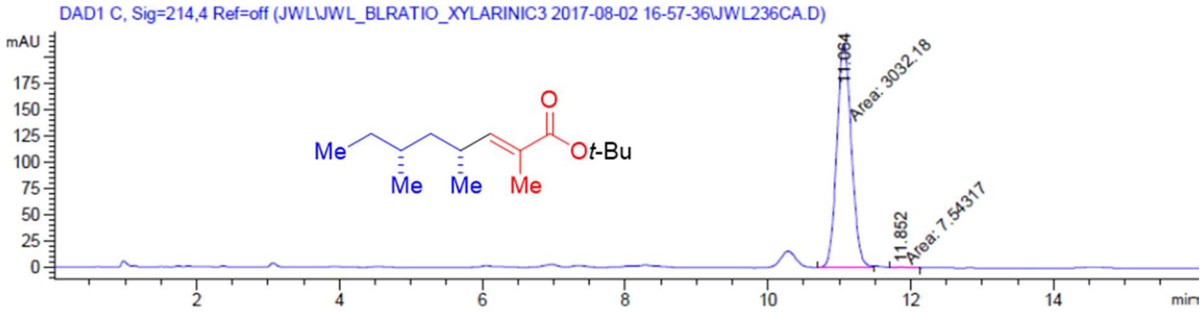

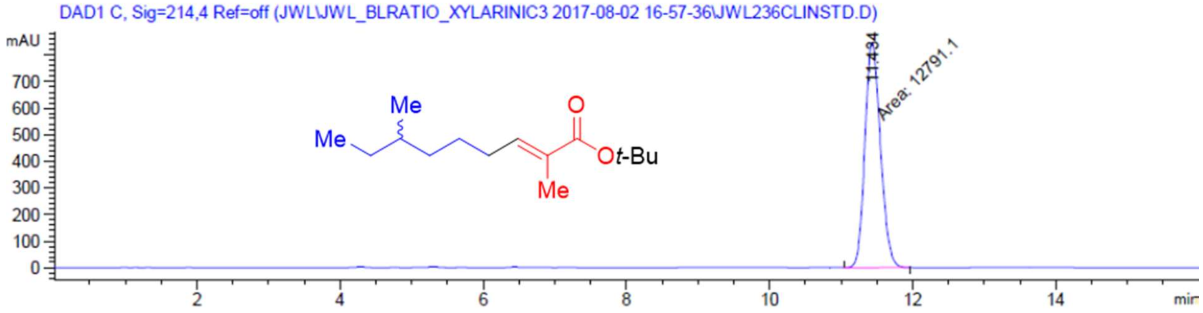

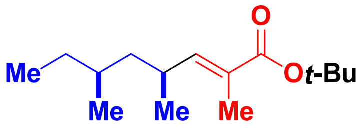

(-)-3ad

Boronic acid **1m** (99.9:0.1 e.r.) was prepared in 65% yield by general procedure C and coupled to organohalide **2p** to give product **(-)-3ad** by general procedure D. The branched/linear product ratio of the crude reaction was determined to be 181/1 by HPLC (Eclipse XDB-C8 column, isocratic 75/25 MeCN/H_2O, 1.2 mL/min, 214.4 nm absorbance). Branched = 10.7 minutes; linear = 11.7 minutes. The product was isolated in 46% yield (11.9 mg) by purification with normal phase flash chromatography (2/1 Hex/DCM). The d.r. of the purified product was determined by a sequence of deprotection and derivatization to the corresponding phenyl amide (**SI-35**) to be 99.2:0.4 (98.6%DS).

A duplicate run of the reaction gave a branched/linear product ratio of 600/1, isolated yield of 54%, and diastereospecificity of 99.0%.

^{1}H NMR matches that of **(-)-3ac**.

^{13}C NMR matches that of **(-)-3ac**.

[?]$^{22}_{D}$ = -39.1 (c. 0.99, $CDCl_3$)

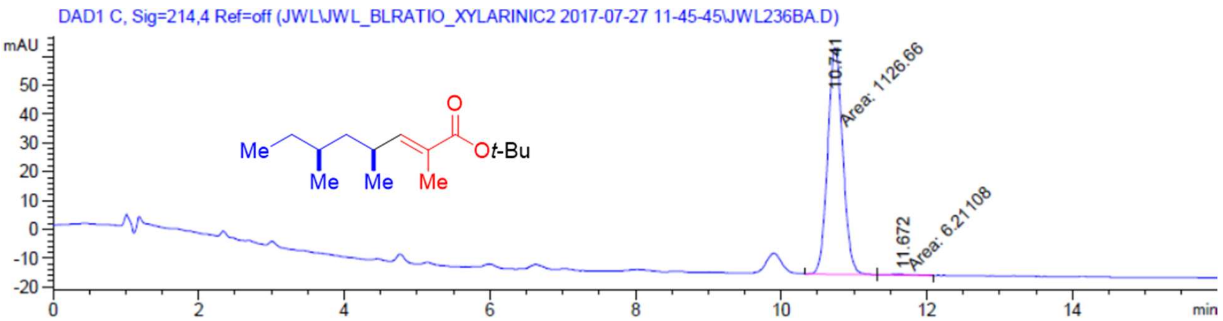

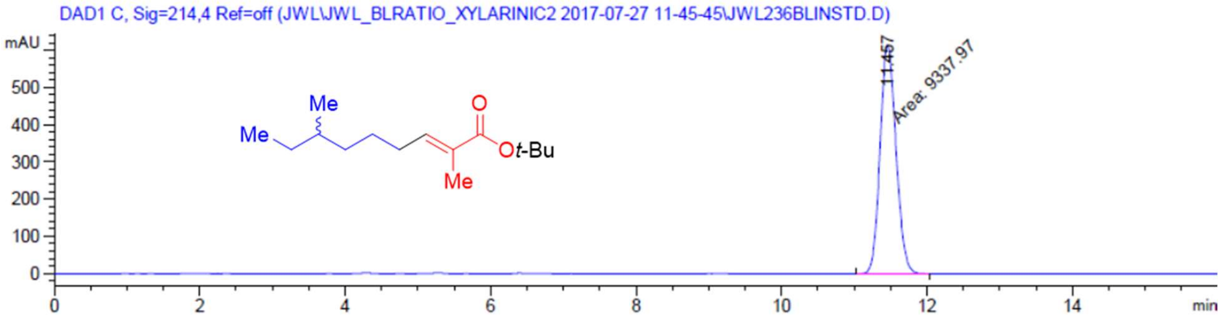

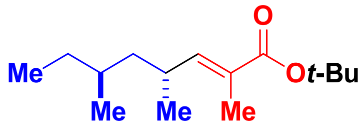

(-)-3ae

Boronic acid **1n** (99.3:0.7 e.r.) was prepared in 73% yield by general procedure C and coupled to organohalide **2p** to give product **(-)-3ae** by general procedure D. The branched/linear product ratio of the crude reaction was determined to be 618/1 by HPLC (Eclipse XDB-C8 column, isocratic 75/25 MeCN/H_2O, 1.2 mL/min, 214.4 nm absorbance). Branched = 10.8 minutes; linear = 11.7 minutes. The product was isolated in 69% yield (17.0 mg) by purification with normal phase flash chromatography (2/1 Hex/DCM). The d.r. of the purified product was determined by a sequence of deprotection and derivatization to the corresponding phenyl amide (**SI-36**) to be 99.4:0.6 (100%DS).

A duplicate run of the reaction gave a branched/linear product ratio of 669/1, isolated yield of 64%, and diastereospecificity of 99.8%.

^{1}H NMR matches that of **(+)-3ab**.

^{13}C NMR matches that of **(+)-3ab**.

[?]$^{23}_{D}$ = -26.3 (c. 0.58, MeOH)

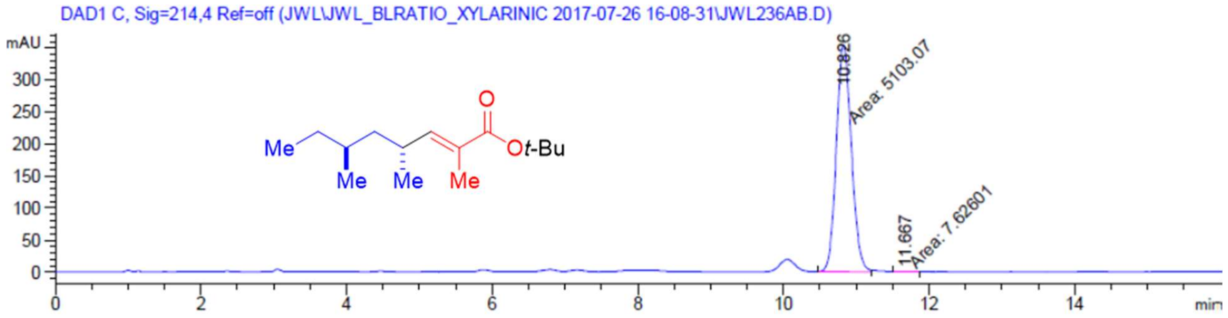

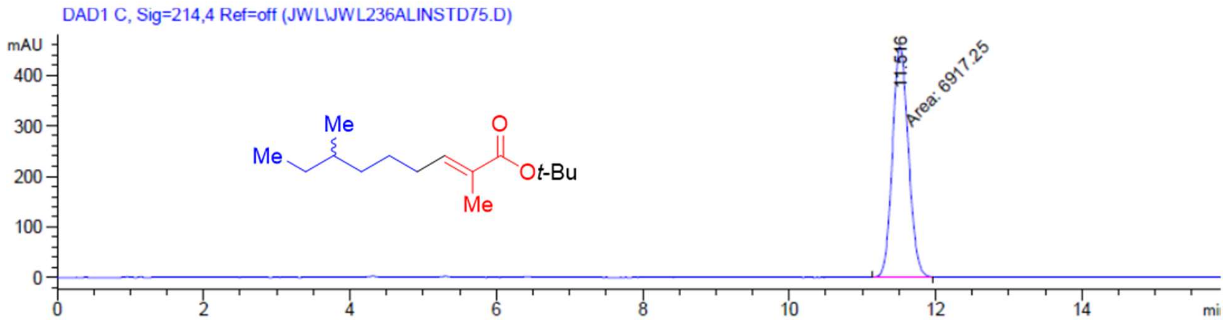

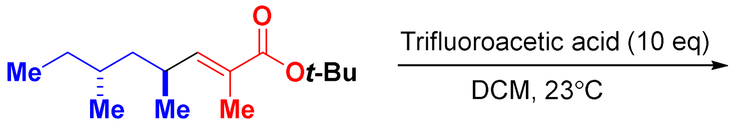

(+)-3ab **4-epi-xylarinic acid B**

4-*epi*-xylarinic acid B. A stir bar-equipped, 2 mL screw-cap vial was charged with the *tert*-butyl ester **(+)-3ab** (12.1 mg, 0.0503 mmol). DCM (0.50 mL, 0.10 Molar) was added, followed by trifluoroacetic acid (dropwise over 1 minute at room temperature, 39 µL, 58 mg, 0.51 mmol, 10 eq). The vial was capped and stirred at room temperature for four hours. At this time, TLC (10% EtOAc in hexanes, $KMnO_4$ stain) showed complete conversion to a more polar spot. The solvent and trifluoroacetic acid were removed by rotary evaporation. Toluene was added and evaporated three times to remove residual trifluoroacetic acid. The carboxylic acid was obtained as a colorless oil (9.55 mg, 0.518 mmol, quantitative yield) without purification.

^{1}H NMR (500 MHz, $CDCl_3$) δ 6.70 (dq, *J* = 10.2, 1.4 Hz, 1H), 2.61 (dsext, *J* = 9.4, 6.7 Hz, 1H), 1.84 (d, *J* = 1.4 Hz, 3H), 1.40 – 1.28 (m, 3H), 1.20 – 1.04 (m, 2H), 0.98 (d, *J* = 6.6 Hz, 3H), 0.89 – 0.81 (m, 6H).

^{13}C NMR (126 MHz, $CDCl_3$) δ 173.71, 151.55, 125.13, 43.86, 32.20, 31.21, 29.38, 19.83, 19.57, 12.18, 11.34.

$[?]^{23}_{D}$ = +30.6 (c. 0.55, MeOH)

HRMS (ESI+) Calculated for $C_{11}H_{20}O_2$ $(M)^+$: 184.1463, Found: 184.1465

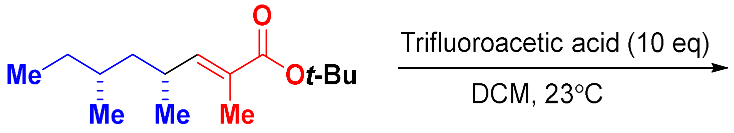

(-)-3ac **xylarinic acid B**

Xylarinic acid B was prepared (colorless oil, 6.18 mg, 0.0335 mmol, 99% yield) using the same procedure as for 4-*epi*-xylarinic acid B.

^{1}H NMR (500 MHz, $CDCl_3$) δ 6.66 (dq, *J* = 10.2, 1.4 Hz Hz, 1H), 2.63 (tdd, *J* = 13.4, 9.1, 5.7 Hz, 1H), 1.86 (d, *J* = 1.4 Hz, 3H), 1.37 (ddd, *J* = 13.5, 9.6, 4.2 Hz, 1H) 1.33 – 1.19 (m, 2H), 1.18 – 1.08 (m, 2H), 0.99 (d, *J* = 6.6 Hz, 3H), 0.85 (t, *J* = 7.2 Hz, 3H), 0.83 (d, *J* = 6.3 Hz, 3H).

^{13}C NMR (126 MHz, $CDCl_3$) δ 173.48, 151.27, 125.51, 44.21, 32.51, 31.28, 30.20, 20.55, 19.18, 12.27, 11.39.

$[?]^{23}_{D}$ = -41.0 (c. 0.10, MeOH)

HRMS (ESI+) Calculated for $C_{11}H_{20}O_2$ $(M)^+$: 184.1463, Found: 184.1465

Comparison of ^{13}C-NMR chemical shifts for xylarinic acid B with the literature.[23]

Observed	Reported
173.48	172.7
151.27	151.3
125.51	125.4
44.21	44.2
32.51	32.5
31.28	31.3
30.20	30.2
20.55	20.6
19.18	19.2
12.27	12.3
11.39	11.4

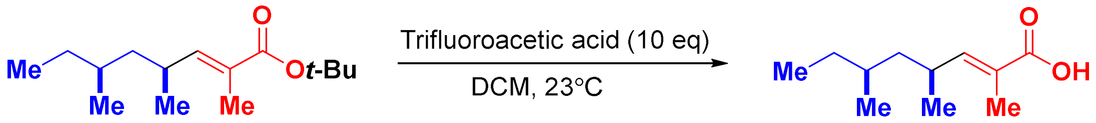

(-)-3ad → ***ent*-xylarinic acid B**

ent-xylarinic acid B was prepared (colorless oil, 5.08 mg, 0.0276 mmol, quantitative yield) using the same procedure as for 4-*epi*-xylarinic acid B.

^{1}H NMR matches that of xylarinic acid B.

^{13}C NMR matches that of xylarinic acid B.

$[\alpha]^{23}_{D}$ = +52.5 (c. 0.40, MeOH)

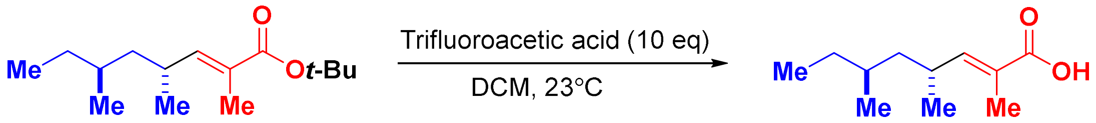

(-)-3ae → **6-*epi*-xylarinic acid B**

6-*epi*-xylarinic acid B was prepared (colorless oil, 5.04 mg, 0.0273 mmol, 92% yield) using the same procedure as for 4-*epi*-xylarinic acid B.

^{1}H NMR matches that of 4-*epi*-xylarinic acid B.

^{13}C NMR matches that of 4-*epi*-xylarinic acid B.

$[\alpha]^{23}_{D}$ = -26.3 (c. 0.58, MeOH)

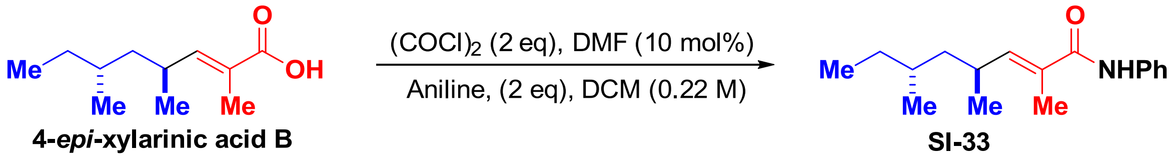

Phenyl amide SI-33. **4-*epi*-xylarinic acid B** (6.49 mg, 0.0352 mmol, 1.00 eq) was massed out in a stir bar-equipped 2 mL screw cap vial. Under nitrogen, anhydrous DCM (0.100 mL) was added, followed by a solution of oxalyl chloride in DCM (1.17 Molar, 60 µL, 0.0070 mmol, 2.0 eq) and a solution of DMF in DCM (1.3 Molar, 2.7 µL, 3.5 µmol, 10 mol%). The mixture was stirred under nitrogen for 20 minutes at room temperature, and then the volatiles (DCM and excess oxalyl chloride) were removed by a stream of nitrogen. Additional DCM (100 µL) was added, followed by a solution of aniline in DCM (0.548 Molar, 129 µL, 0.71 mmol, 2.0 eq). The reaction was stirred for 10 more minutes and then quenched by addition of 1M HCl (0.50 mL). The aqueous layer was extracted twice with DCM, and the extracts were passed through silica gel in a cotton-plugged glass pipet, rinsing with 10% EtOAc/hexanes. The filtrate was concentrated *in vacuo*. The d.r. of the crude phenyl amide was determined to be 99.6:0.4 (99.4 %DS) by HPLC (OD-H chiral column, 1% IPA in hexanes, 2.0 mL/min, 254.4 nm absorbance). Major = 17.7 minutes; minor = 15.5 minutes. The second of the run reaction also gave 99.4%DS.

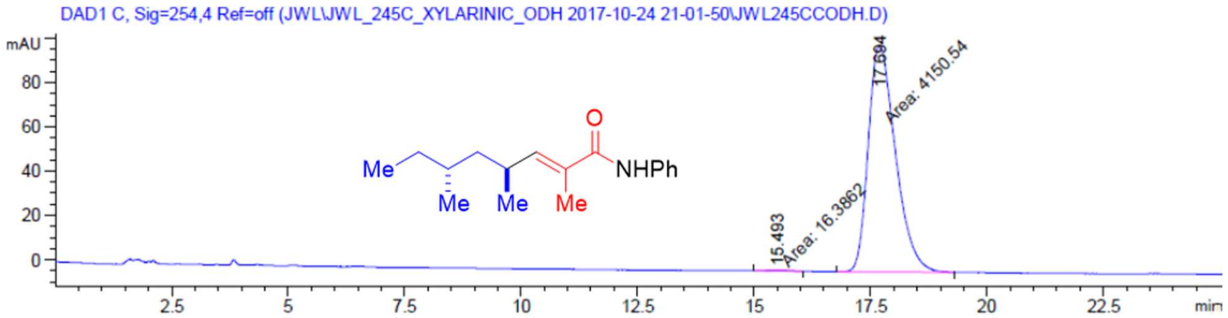

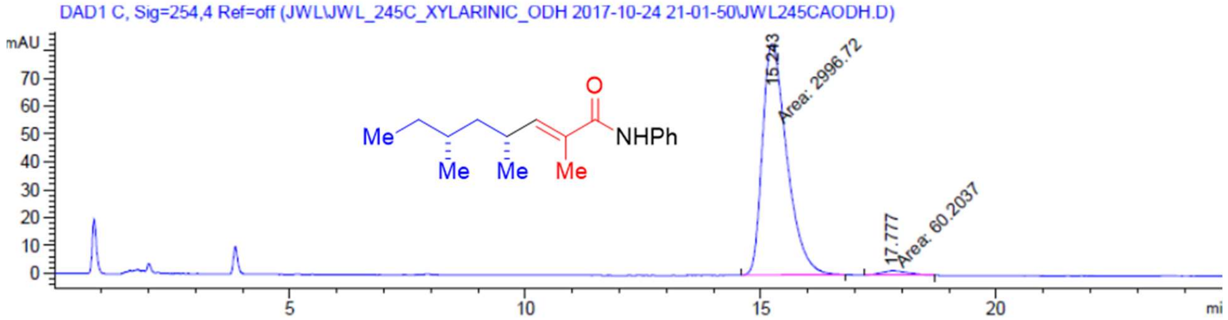

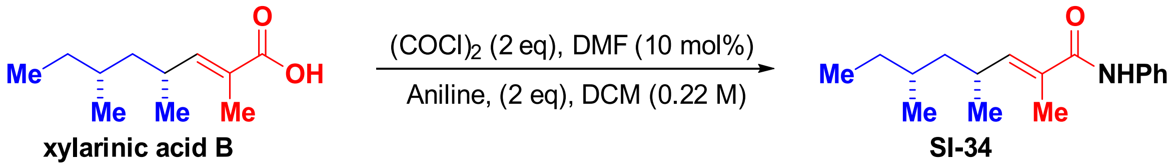

Phenyl Amide SI-34 was prepared from **xylarinic acid B** using the same procedure for phenyl amide **SI-33**. The d.r. of the crude phenyl amide was determined to be 98.0:2.0 (98.6%DS) by HPLC (OD-H chiral column, 1% IPA in hexanes, 2.0 mL/min, 254.4 nm absorbance). Major = 15.2 minutes; minor = 17.8 minutes. The second of the run reaction gave 98.5%DS.

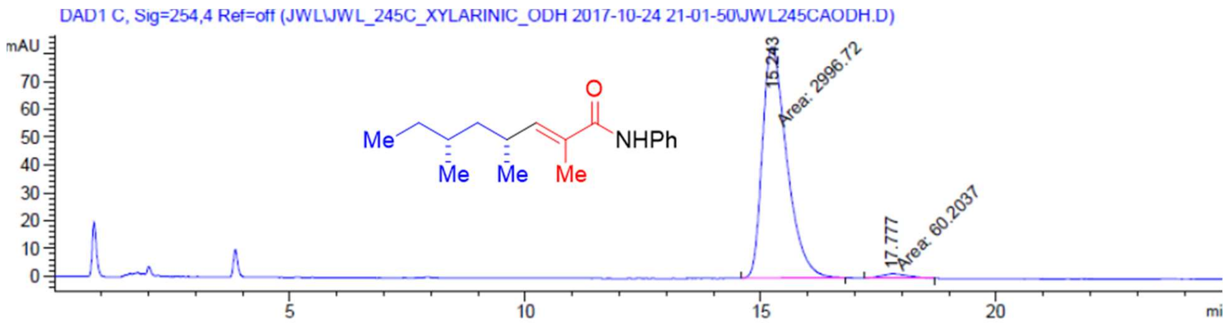

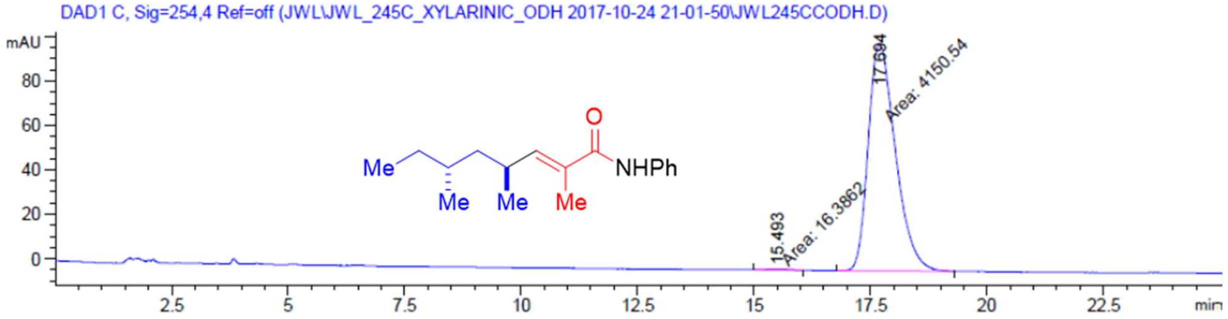

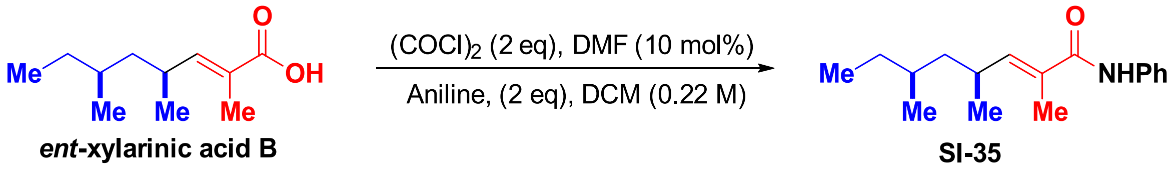

Phenyl Amide SI-35 was prepared from ***ent*-xylarinic acid B** using the same procedure for phenyl amide **SI-33**. The d.r. of the crude phenyl amide was determined to be 99.2:0.8 (98.6%DS) by HPLC (OD-H chiral column, 1% IPA in hexanes, 2.0 mL/min, 254.4 nm absorbance). Major = 21.7 minutes; minor = 17.8 minutes. The second of the run reaction gave 99.0%DS.

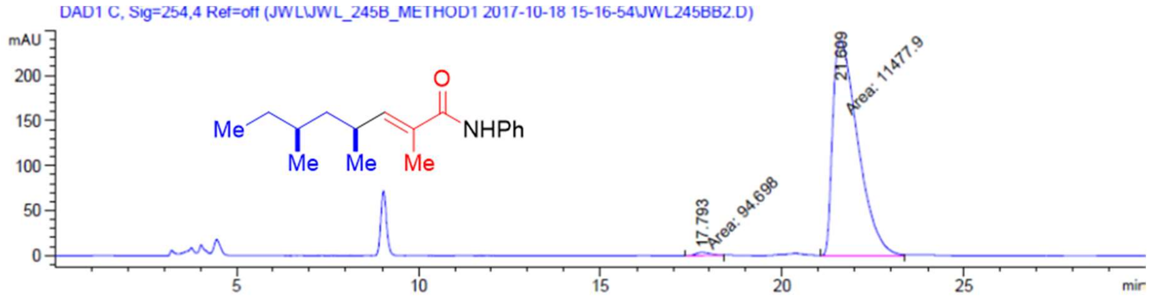

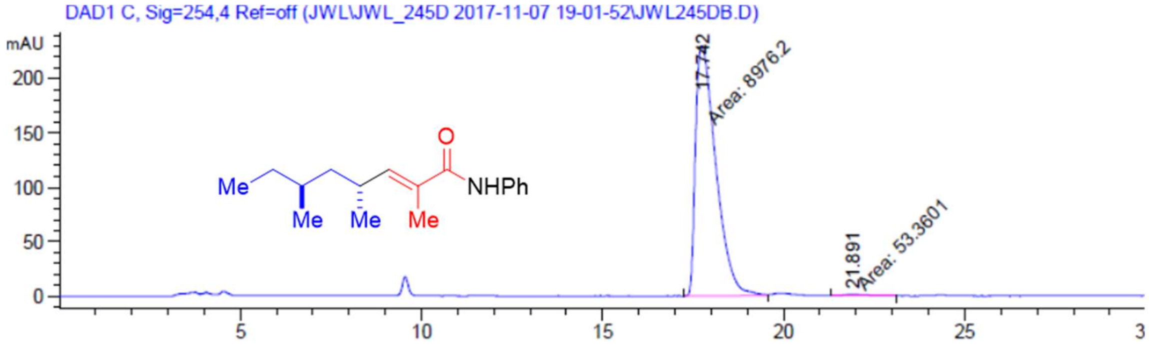

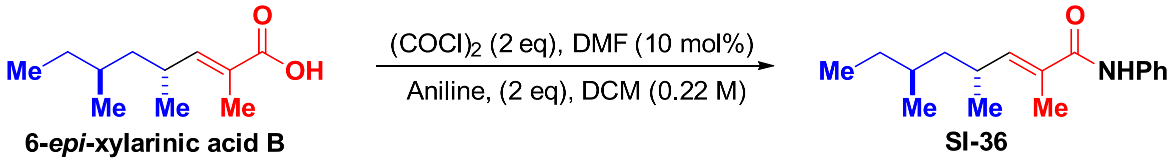

Phenyl Amide SI-36 was prepared from **6-*epi*-xylarinic acid B** using the same procedure for phenyl amide **SI-33**. The d.r. of the crude phenyl amide was determined to be 99.4:0.6 (100%DS) by HPLC (OD-H chiral column, 1% IPA in hexanes, 2.0 mL/min, 254.4 nm absorbance). Major = 17.7 minutes; minor = 21.9 minutes. The second of the run reaction gave 99.8%DS.

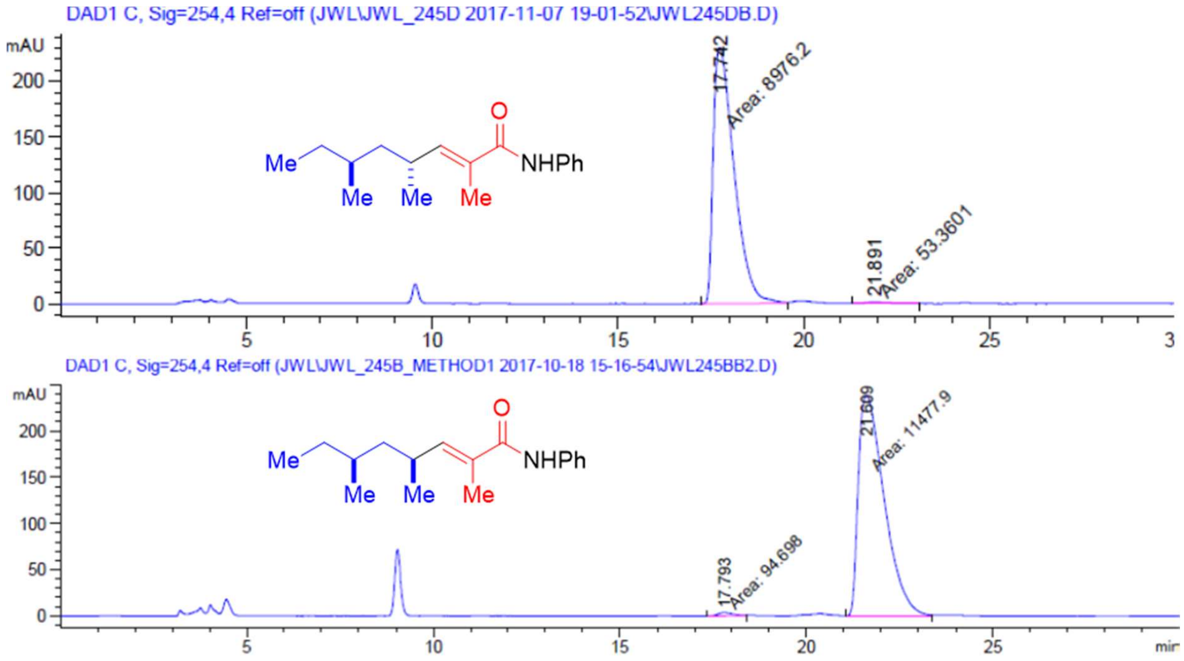

Absolute Configuration of BIDA Boronates

Determining the absolute configuration of the C2 stereocenter of **5a**:

The absolute stereochemistry of the 2-butyl stereocenter was determined by x-ray crystallography of crystals grown by slow diffusion of Et_2O into an acetone solution of **5a** at 23°C using the known stereocenters of the cyclopentyl ring as reference.

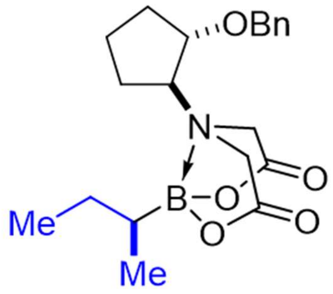

5a, ≥99:1 d.r.

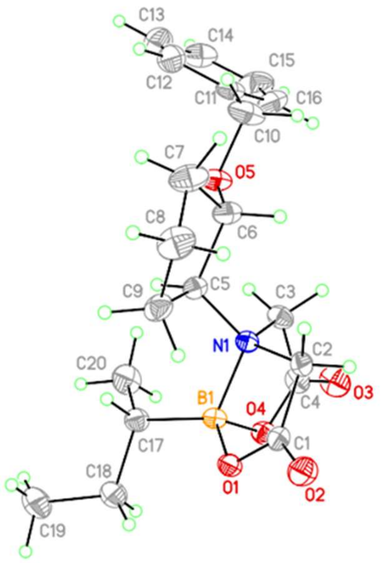

Determining the absolute configuration of the C2 stereocenter of **7d**:

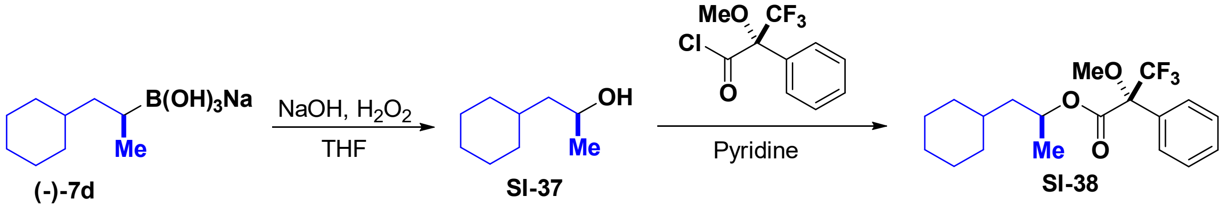

The trihydroxyborate salt **(-)-7d** (synthesized from **5d** using general procedure B) was treated with 1M NaOH (5 equiv) followed by 30% H_2O_2 (3 eq) dropwise, causing the product to oil out. The mixture was stirred for 1 h at 23 °C, then quenched with saturated aqueous sodium thiosulfate (10 equiv). The solution was extracted with DCM, dried with Na_2SO_4, and concentrated to give **SI-37** an oil. The crude alcohol (4.0 mg, 0.028 mmol) thus obtained was dissolved in pyridine (50 µL, 0.056 Molar) and treated with (*S*)-(+)-α-methoxy-α-trifluoromethylphenylacetyl chloride (10 mg, 0.04 mmol, 1.4 eq) at 23 °C and stirred for three hours. 2M HCl was added and the mixture extracted with EtOAc twice. The organic phase was dried with Na_2SO_4 and concentrated under vacuum. The product **SI-38** (7 mg, 0.020 mmol 70%) was obtained after purification by silica gel chromatography (2% EtOAc/hexanes). The ^1H NMR and ^{13}C NMR matched that of **SI-38** independently synthesized from (*S*)-propylene oxide as described below, thus confirming that the configuration of the C2 stereocenter is (*S*).

Independent synthesis of **SI-37** and **SI-38**:

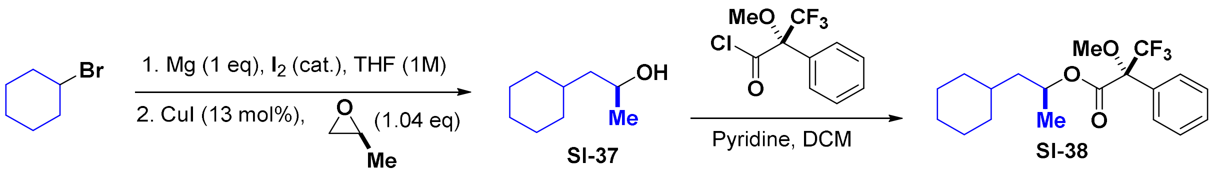

Following a literature procedure,[24] a 7-mL vial was charged with Mg turnings (24 mg, 1 mmol, 1 eq) and I_2 (1 mg, catalytic), followed by anhydrous THF (1 mL, 1 Molar) and bromocyclohexane (163 mg, 1 mmol, 1 eq) under nitrogen. The mixture was stirred at 50 °C until most of the Mg dissolved. CuI (24 mg, 0.13 mmol) was added. (*S*)-propylene oxide (Aldrich # 540021, 73 µL, 1.04 mmol) was then added dropwise at 23 °C. After stirring for 1 h, the black suspension was quenched with saturated aqueous NH_4Cl, and the mixture extracted with EtOAc. The organics were dried with Na_2SO_4, filtered and concentrated. The crude product was purified by silica gel column (10% to 15% EtOAc/hexanes) to give **SI-37**[25] as a colorless oil. Spectra matched those reported previously.

A 7 mL vial equipped with a stir bar was charged with **SI-37** (15.2 mg, 0.107 mmol, 1.0 eq), DCM (0.5 mL, 0.21 Molar) and pyridine (30 µL, 0.37 mmol, 3.5 eq). (*S*)-(+)-α-Methoxy-α-trifluoromethylphenylacetyl chloride (20 µL, 0.107 mmol, 1.0 eq) was added at 23 °C and the reaction stirred at the same temperature overnight. The solvent was removed under a stream of nitrogen and the crude product loaded onto a silica gel column with 4% Et_2O/pentane. After silica gel purification (4% Et_2O/pentane), a white crystalline solid was obtained as the desired product **SI-38** (34.9 mg, 0.0974 mmol, 91%).

^{1}H NMR (500 MHz, $CDCl_3$) δ 7.57 – 7.51 (m, 2H), 7.43 – 7.36 (m, 3H), 5.26 (ddt, *J* = 11.1, 4.9, 3.1 Hz, 1H), 3.58 (q, *J* = 1.3 Hz, 3H), 1.73 – 1.48 (m, 6H), 1.33 (d, *J* = 6.2 Hz, 3H), 1.28 (ddd, *J* = 14.1, 8.7, 4.4 Hz, 1H), 1.16-1.02 (m, 4H), 0.90 – 0.74 (m, 2H).

^{13}C NMR (126 MHz, $CDCl_3$) δ 166.27, 132.80, 129.60, 128.48, 127.30, 123.37 (q, $J_{C\text{-}F}$ = 289.4 Hz), 71.89, 55.57, 43.52, 33.79, 33.77, 32.65, 26.56, 26.31, 26.16, 20.61.

$[\alpha]^{20}_D$ = +68.4 (c 1.74, $CHCl_3$)

HRMS (ESI+) Calculated for $C_{19}H_{25}O_3F_3Na$ $(M+Na)^+$: 381.1653, Found: 381.1648

Determination of the absolute configuration of **(1*S*, 2*S*)-5l**:

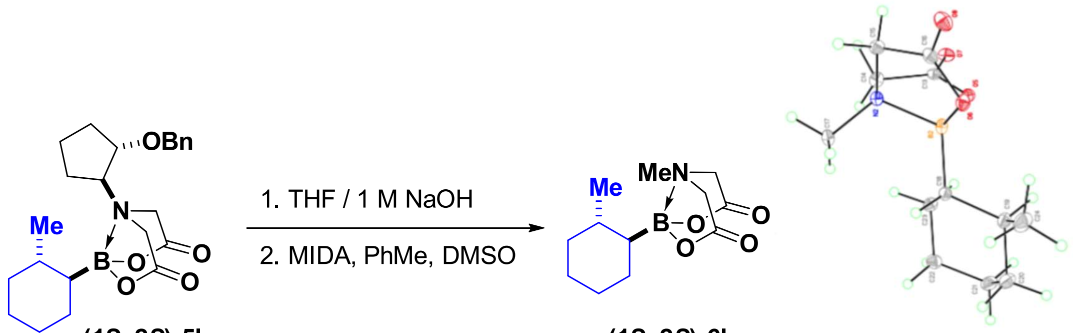

(1*S*, 2*S*)-5l **(1*S*, 2*S*)-6l**

248 mg (0.6 mmol) of **(1*S*, 2*S*)-5l** was hydrolyzed and the boronic acid was obtained as a solution in MTBE after workup as described in general procedure B. The boronic acid solution was used directly in the complexation with MIDA ligand by general procedure A. 100 mg (0.4 mmol) of **(1*S*, 2*S*)-6l** was obtained as a white crystalline solid in 66% overall yield. X-ray crystals were grown by solvent layering of hexanes over a homogeneous solution of **6l** in EtOAc. The absolute configuration was assigned by x-ray crystallography.

^{1}H NMR (500 MHz, DMSO-*d6*) δ 4.19 (d, *J* = 17.2 Hz, 1H), 4.15 (d, *J* = 17.1 Hz, 1H), 3.98 (d, *J* = 17.0 Hz, 1H), 3.93 (d, *J* = 17.3 Hz, 1H), 2.85 (s, 3H), 1.83 – 1.76 (m, 1H), 1.74 – 1.65 (m, 2H), 1.57 – 1.40 (m, 2H), 1.26 – 1.19 (m, 1H), 1.11 – 1.04 (m, 1H), 1.00 (d, *J* = 6.5 Hz, 3H), 0.62 (q, *J* = 8.8 Hz, 1H).

^{13}C NMR (126 MHz, DMSO-*d6*) δ 169.16, 169.08, 61.93, 45.84, 36.65, 36.16, 32.31, 28.17, 25.87, 22.19.

^{11}B NMR (128 MHz, DMSO-*d6*) δ 12.34.

$[\alpha]^{20}$D = -7.6 (c 1, acetone)

HRMS (ESI^{+}) Calculated for $C_{12}H_{21}BNO_4$: 254.1564, Found: 254.1561

Determining the absolute configuration of the C2 stereocenter of **5m** for xylarinic acid and 4-*epi*-xylarinic acid:

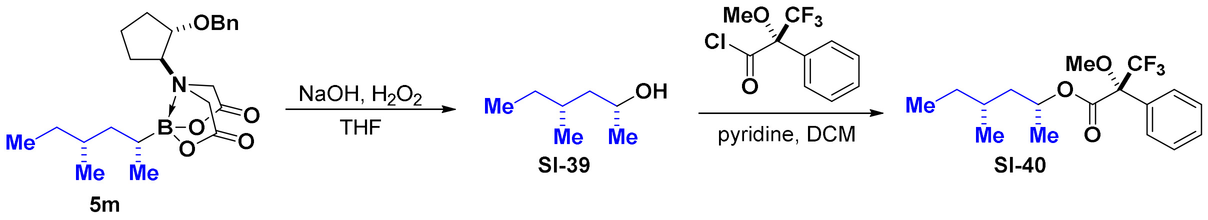

To a solution of BIDA boronate **5m** (20 mg, 0.048 mmol) in THF (0.24 mL) was added 1N NaOH (0.24 mL, 0.24 mmol, 5.0 eq). The mixture was stirred for 5 minutes, and then 30% H_2O_2 (25 µL, 0.24 mmol, 5.0 eq) was added. The resulting mixture was stirred for 15 min, then diluted with Et_2O. The organic layer was washed with saturated aqueous $Na_2S_2O_3$, washed with brine, dried over Na_2SO_4, filtered, and concentrated under vacuum to give alcohol **SI-39**. The alcohol was dissolved in DCM (0.48 mL, 0.1 Molar), and then pyridine (12 µL, 0.144 mmol, 3.0 eq) and (*S*)-(+)-α-Methoxy-α-trifluoromethylphenylacetyl chloride (10 µL, 0.053 mmol, 1.1 eq) were added at 23 °C. The reaction was stirred overnight and then quenched with the addition of H_2O. The product was extracted with DCM, dried over Na_2SO_4, filtered and concentrated. After silica gel purification (20% Et_2O/pentane), the Mosher ester **SI-40**, was obtained as a colorless oil. The NMR data matches that of **SI-40** below, thus confirming that the C2 stereocenter is (*R*).

^{1}H NMR (500 MHz, $CDCl_3$) δ 7.55 – 7.50 (m, 2H), 7.42-7.36 (dd, *J* = 5.0, 2.0 Hz, 3H), 5.25 (m, 1H), 3.55 (d, *J* = 1.5 Hz, 3H), 1.75 (ddd, *J* = 14.0, 9.3, 4.6 Hz, 1H), 1.39 (dt, *J* = 12.9, 6.8 Hz, 1H), 1.34 – 1.13 (m, 6H), 0.89 (d, *J* = 6.6 Hz, 3H), 0.85 (t, *J* = 7.4 Hz, 3H).

^{13}C NMR (126 MHz, $CDCl_3$) δ 166.38, 132.55, 129.63, 128.47, 127.52, 123.51 (q, J_{C-F} = 289.4 Hz), 72.58, 55.53, 42.86, 31.02, 29.90, 20.39, 18.99, 11.34.

HRMS (ESI$^+$) Calculated for $C_{17}H_{23}O_3F_3Na$ $(M+Na)^+$: 355.1497, Found: 355.1497

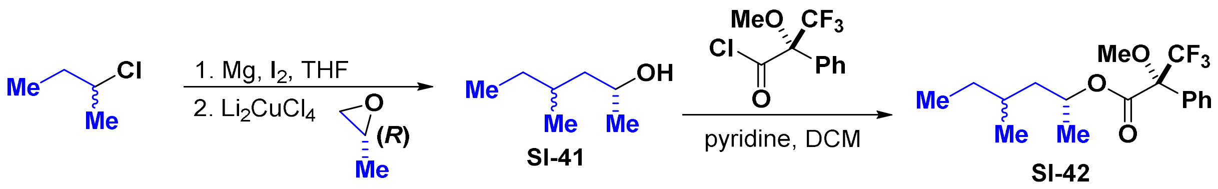

In an unoptimized procedure, 2-chlorobutane (0.635 mL, 6 mmol, 1.0 eq) was added to a mixture of Mg (146 mg, 6 mmol, 1.0 eq), I_2 (one crystal) and Et_2O (6 mL, 1.0 Molar) dropwise at 23 °C. The reaction was stirred for 2.5 hours after the addition. The solution of the Grignard reagent was then added dropwise to a solution of (*R*)-propylene oxide (0.14 mL, 2 mmol, 0.33 eq) and Li_2CuCl_4 (1M in THF, 2 mL, 0.2 mmol, 0.033 eq) in THF (6 mL) at -50 °C. The reaction was warmed to room temperature and stirred overnight, then cooled to 0 °C. Saturated aqueous NH_4Cl (12 mL) was added, and the mixture was stirred until most of the brown solids dissolved. The mixture was transferred to a separatory funnel with H_2O and Et_2O (10 mL). After mixing and phase separation, the aqueous layer was extracted with Et_2O (10 mL). The combined organics were washed with brine, dried over $MgSO_4$, filtered, and concentrated. The residue was purified by silica gel chromatography (30 to 40% Et_2O/pentane) to give alcohol **SI-41** (130 mg, 37%). The Mosher ester **SI-42**, which is a mixture of diastereomers with a stereodefined C2 stereocenter was synthesized from **SI-41** using the procedure described above for the synthesis **SI-40** from **SI-39**.

^1H NMR (500 MHz, $CDCl_3$) δ 7.56 – 7.50 (m, 4H), 7.42 – 7.37 (m, 6H), 5.36 – 5.14 (m, 2H), 3.55 (dq, *J* = 2.5, 1.3 Hz, 6H), 1.75 (ddd, *J* = 14.0, 9.3, 4.6 Hz, 1H), 1.61 – 1.53 (m, 1H), 1.51 – 1.36 (m, 4H), 1.34 – 1.08 (m, 10H), 0.91 – 0.80 (m, 12H).

^{13}C NMR (126 MHz, $CDCl_3$) δ 166.38, 166.29, 132.56, 129.64, 128.49, 127.52, 123.51 (q, J_{C-F} = 289.2 Hz), 72.83, 72.58, 55.52, 42.86, 42.74, 31.10, 31.02, 29.90, 29.11, 20.38, 19.83, 19.36, 18.99, 11.34, 11.23.

Absolute Configuration of Coupled Products

To determine the absolute stereochemistry of **(+)-3a** obtained from the coupling reaction of **(*S*)-1a** and **2a**, **(*S*)-3a** was independently synthesized from (*S*)-3-phenylbutyric acid:

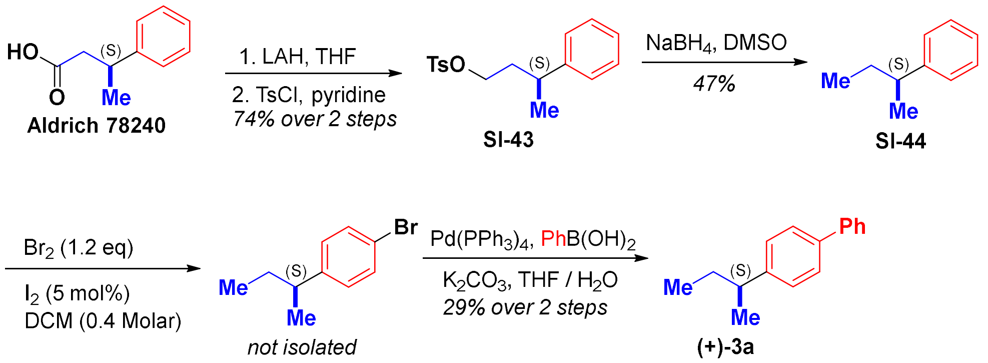

SI-43. In a glovebox, lithium aluminum hydride powder (88 mg, 2.2 mmol) was added to a dry 3-neck flask. This was fitted with a reflux condenser and 2 septa. In a fume hood, dry THF (10 mL) was added and the mixture was stirred at 23°C. (+)-(*S*)-3-phenylbutyric acid (Sigma Aldrich 78240, Lot # BCBF9385V), was added dropwise as a solution in THF (3.5 mL) and the mixture was stirred at reflux for 12 hours. TLC (3:1 Hex/EtOAc, $KMnO_4$) showed complete conversion. The reaction was quenched with 1M aqueous Rochelle salt and extracted three times with DCM. The combined DCM phase was dried over sodium sulfate and concentrated under vacuum. The crude material (310 mg) was used directly in the next step.

The crude alcohol (310 mg) was combined in a 7 mL vial with distilled pyridine (1.0 mL) and toluenesulfonyl chloride (0.419 g, 2.2 mmol) that had been recrystallized from hot hexanes. The reaction was capped and stirred at 23 °C until complete conversion of the alcohol as seen by TLC. At 5 h, 1M HCl was added and the mixture was extracted three times with Et_2O. The combined Et_2O phase was washed with 1M HCl, then H_2O, then saturated $NaHCO_3$. The solution was dried over sodium sulfate and concentrated under vacuum. The crude was purified by silica column using a gradient of 5% to 15% EtOAc in hexanes, giving the product **SI-43** as a colorless oil (0.460 g, 1.51 mmol, 74% yield from (S)-3-phenylbutyric acid).

^{1}H NMR (400 MHz, $CDCl_3$) δ 7.74 (d, *J* = 8.3 Hz, 2H), 7.32 (d, *J* = 8.0 Hz, 2H), 7.25 – 7.13 (m, 3H), 7.04 (d, *J* = 7.1 Hz, 2H), 3.97 (dt, *J* = 9.8, 5.9 Hz, 1H), 3.82 (ddd, *J* = 9.8, 7.9, 5.8 Hz, 1H), 2.81 (m, 1H), 2.45 (s, 3H), 2.00 – 1.80 (m, 2H), 1.21 (d, *J* = 7.0 Hz, 3H).

^{13}C NMR (101 MHz, $CDCl_3$) δ 145.46, 144.78, 133.16, 129.93, 128.66, 128.02, 127.01, 126.47, 68.98, 37.17, 35.97, 22.15, 21.79.

$[\alpha]^{20}_D$ = +97.1 (c 1.0, $CDCl_3$)

HRMS (EI^+) Calculated for $C_{17}H_{20}O_3S$ $(M)^+$: 304.1133, Found: 304.1132

SI-44. To a 20 mL vial with a septum cap and stir bar were added (*S*)-3-phenylbutyl 4-methylbenzenesulfonate **SI-45** (0.450 g, 1.45 mmol, 1.00 eq), followed by DMSO (8 mL) and $NaBH_4$ (0.281 g, 7.43 mmol, 5.1 eq). The headspace was purged with nitrogen the reaction was stirred at 70°C, during

which time the reaction became homogeneous. Monitoring of the reaction by TLC (4:1 Hex/EtOAc, $KMnO_4$) showed complete conversion of the substrate at 20 hours. H_2O (8 mL) was added and the solution was extracted four times with pentane. The combined pentane phase was washed twice with H_2O, then with 3% H_2O_2, and again with H_2O. The solution was dried over sodium sulfate and concentrated under light vacuum to give **SI-44** as an oil (0.128 g, 0.770 mmol, 47% yield). This was used in the next step without purification. The spectral properties of *sec*-butylbenzene were identical to those reported previously.[26] Comparison of the optical rotation to the literature value showed a high level of enantiopurity.

$[\alpha]^{20}_{D}$ = +27.4 (c 1.0, $CHCl_3$)

Lit:[27] $[\alpha]^{20}_{D}$ = +25.0 (c 1.0, $CHCl_3$)

(*S*)-3a. Elemental bromine (12.4 µl, 38.5 mg, 0.241 mmol) was added dropwise to a solution of *sec*-butylbenzene **SI-44** (27 mg, 0.16 mmol, 1.0 eq) and iodine (2.5 mg, 0.010 mmol, 6 mol%) in DCM (0.5 mL, 0.3 Molar) in an ice bath under nitrogen. The reaction was stirred at 23 °C for 2 hours. The conversion could not be determined by TLC (100% pentane). The reaction was quenched with 0.5M KOH and extracted twice with DCM. The DCM phase was dried over sodium sulfate and concentrated under vacuum. This material was filtered through a silica plug with pentane and concentrated to afford an oil (23mg). ^{1}H-NMR shows a 1:1 ratio of starting material (0.061 mmol, 10.1 mg) to product (0.061 mmol, 12.9 mg). This mixture was taken on to the coupling reaction without purification.

To the crude product from the above reaction in a 7 mL vial in a glove box were added $Pd(PPh_3)_4$ (1.7 mg, 1.5 µmol, 2.5 mol%), phenylboronic acid (12.1 mg, 0.10 mmol, 1.6 eq), K_2CO_3 (0.236 g, 1.9 mmol, 32 eq), and THF (0.8 mL, 0.08 Molar). In a fume hood, H_2O (0.57 mL, 0.11 Molar) was added. The headspace was purged with nitrogen, capped and stirred 10 hours at 75°C. TLC (100% pentane, UV) showed product. The THF was removed under vacuum. The solution was then extracted three times with pentane. The pentane phase was dried over sodium sulfate, concentrated under vacuum, and purified by silica column with 100% pentane, giving **(*S*)-3a** as a colorless oil (12.3 mg, 0.0507 mmol, 32% yield).

^{1}H NMR (500 MHz, $CDCl_3$) δ 7.61 (d, *J* = 7.1 Hz, 2H), 7.55 (d, *J* = 8.2 Hz, 2H), 7.45 (t, *J* = 7.7 Hz, 2H), 7.34 (t, *J* = 7.4 Hz, 1H), 7.28 (d, *J* = 8.3 Hz, 2H), 2.66 (sext, *J* = 7.0 Hz, 1H), 1.65 (pd, *J* = 7.3, 2.2 Hz, 2H), 1.30 (d, *J* = 7.0 Hz, 3H), 0.88 (t, *J* = 7.4 Hz, 3H).

^{13}C NMR (101 MHz, $CDCl_3$) δ 146.95, 141.30, 138.81, 128.81, 127.60, 127.12, 127.11, 127.06, 41.48, 31.32, 21.98, 12.46.

The product **(*S*)-3a** had an e.r. of > 99.5:0.5 as determined using a Chiralcel OD-H column of 4.6 mm x 250 mm, hexanes, 2.0 mL/min., 210 nm absorbance. Major: 5.7, Minor: 9.3. The retention time of the **(*S*)-3a** obtained here matches that of the coupling product **(+)-3a**, thus confirming that the coupling reaction went with *stereoretention*.

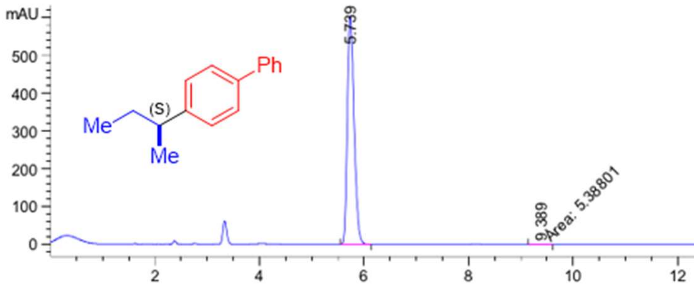

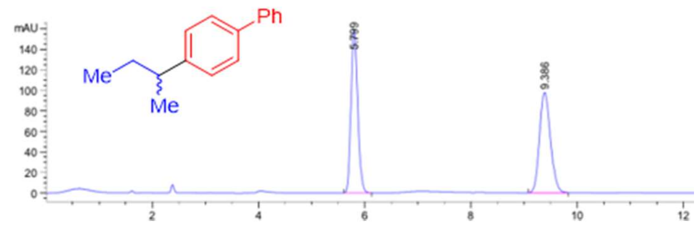

Crystal Structures

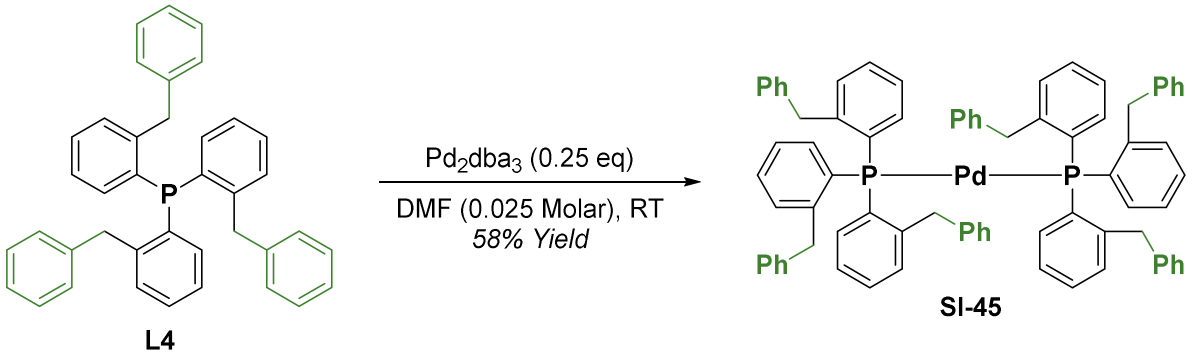

Bis[tris(2-benzyl-phenyl)phosphine] SI-45. A solution of P(2-benzyl-phenyl)$_3$ (0.533 g, 1.00 mmol, 1.00 eq) and Pd_2dba_3 (0.229 g, 0.25 mmol, 0.50 eq) was prepared in anhydrous DMF (40 mL, 0.025 Molar) under nitrogen. The reaction was stirred for 10 hours at room temperature, during which time a yellow-green precipitate formed and the dark red color of Pd_2dba_3 faded. The DMF was removed by filteration with by syringe. The filtered solid was washed with Et_2O (5 mL), and the color began to change. The solid was dissolved in THF, causing a color change to dark orange. The solution was drawn portionwise into a 20 mL syringe. A disc-shaped filter was fitted to the syringe, and then it was equipped with a needle. After filtration, solution was recrystallized from DCM (20 mL) and pentane (60 mL) with overnight stirring at 0°C. Crystals of **SI-45** were collected by filtration (340 mg, 0.290 mmol, 58% yield).

X-ray crystals were prepared by dissolving the product in benzene with slow diffusion into Et_2O.

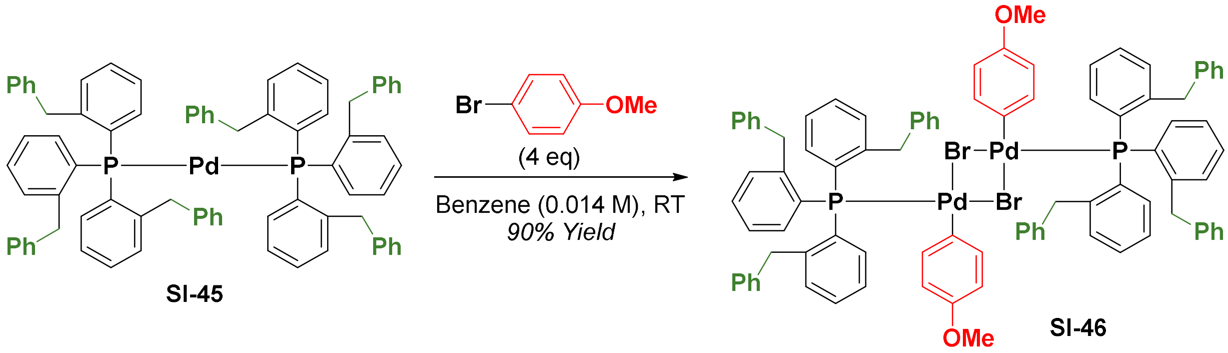

{[(2-Benzyl-phenyl)$_3$P]Pd(4-OMe-phenyl)(Br)}$_2$ SI-46. Bis[tris(2-benzyl-phenyl)phosphine]Pd(0) **SI-45** (50 mg, 0.0427 mmol, 1.00 eq) was combined with 4-bromoanisole (32 mg, 0.171 mmol, 4.0 eq). Dry benzene (3.0 mL, 0.014 Molar) was added under a nitrogen stream, and a solid cap was fitted on the vial. The yellow suspension was stirred for 24 hours at room temperature. Solvent was removed by rotary evaporation, and then the product **SI-46** was washed with Et_2O (2x2.5 mL) using a centrifuge.

X-ray crystals were obtained by dissolving the dimeric complex in a minimal sufficient amount of DCM. This solution was passed through a syringe filter into a 7 mL vial containing Et_2O (approximately 6 mL) using a needle to slowly load the solution into a bottom layer. The vial was filled to the top with Et_2O and fitted with a solid screw cap, allowing crystals to form by slow diffusion.

Crystallography

SI-45

Intensity data were collected on a Bruker D8 Venture kappa diffractometer equipped with a Photon 100 CMOS detector. An Iμs microfocus Mo source (λ = 0.71073 Å) coupled with a multi-layer mirror monochromator provided the incident beam. The sample was mounted on a 0.3 mm loop with the minimal amount of Paratone-N oil. Data was collected as a series of ϕ and/or ω scans. Data was collected at 100 K using a cold stream of $N_{2(g)}$. The collection, cell refinement, and integration of intensity data was carried out with the APEX2 software.[28] A semi-empirical absorption correction was performed with SADABS.[29] The structure was phased with direct methods using SHELXS and refined with the full-matrix least-squares program SHELXL.[30]

A structural model consisting of the host plus one highly disordered diethyl ether solvate molecule was developed; however, positions for the idealized solvate molecules were poorly determined. This model converged with wR2 = 0.1715 and R1 = 0.687 for 496 parameters with 645 restraints against 6325 data. Since positions for the solvate molecule were poorly determined a second structural model was refined with contributions from the solvate molecule removed from the diffraction data using the bypass procedure in PLATON.[31] No positions for the host network differed by more than two su's between these two refined models. The electron count from the "squeeze" model converged in good agreement with the number of solvate molecules predicted by the complete refinement.

One of the ligands was modeled as disordered over 2 sites. The C27/C27a pivot atom positions of bonded to the P atom were constrained to have the same position and same displacement parameters. The disordered ligands were restrained to have the same geometries (esd 0.01 Å). The terminal phenyl rings on the disordered ligands were also constrained to be perfect hexagons. Rigid-bond restraints (esd 0.006) were imposed on displacement parameters for all disordered sites.

H atom treatment - H atoms were included as riding idealized contributors and their U's were assigned as 1.2 times carrier U_{eq}.

3 low angle reflections were omited from the final refinements.

CCDC: 1838473

SI-46

Intensity data were collected on a Bruker D8 kappa diffractometer equipped with an APEXII CCD detector. An fine-focus Mo source (λ = 0.71073 Å) coupled with a graphite monochromator provided the

incident beam. The sample was mounted on a 0.3 mm loop with the minimal amount of Paratone-N oil. Data was collected as a series of ϕ and/or ω scans. Data was collected at 100 K using a cold stream of $N_{2(g)}$. The collection, cell refinement, and integration of intensity data was carried out with the APEX2 software.[28] A semi-empirical absorption correction was performed with SADABS.[29] The structure was phased by intrinsic methods using SHELXS and refined with the full-matrix least-squares program SHELXL.[30]

A structural model consisting of the target molecule, one ordered dichloromethane solvate molecule, and one disordered solvate molecule position in the asymmetric unit was developed; however, positions for the disordered solvate molecule was poorly determined. This model converged with wR2 = 0.2636 and R1 = 0.0783 for 956 parameters with 956 restraints against 14637 data. Since positions for the solvate molecules were poorly determined a second structural model was refined with contributions from the solvate molecules removed from the diffraction data using the bypass procedure in PLATON.[32] No positions for the host network differed by more than two su's between these two refined models. The electron count from the "squeeze" model converged in good agreement with the number of solvate molecules predicted by the complete refinement.

One of the phenyl rings of one phosphine ligand was found to be disordered over two orientations. Both disordered phenyl rings were constrained to be perfect hexagons. Similar displacement amplitudes (esd 0.01) were imposed on disordered sites overlapping by less than the sum of van der Waals radii. Similarity restraints (esd 0.01) were imposed on the C---C bonds joining the disordered phenyl ring to the ordered ligand carbon atom C73. The site occupancy of the two orientations was allowed to freely refine; at convergence the site occupancy ratio was 0.717(8):0.283(8).

H atom treatment - Methyl H atom positions, $R-CH_3$, were optimized by rotation about R-C bonds with idealized C-H, R--H and H--H distances. Remaining H atoms were included as riding idealized contributors. Methyl H atom U's were assigned as 1.5 times U_{eq} of the carrier atom; remaining H atom U's were assigned as 1.2 times carrier U_{eq}.

Due to the large unit cell and limitations on the instrument configuration, several reflections were completely or partially obscured by the beam stop in some orientations. These reflections were omitted from the final refinement.

CCDC: 1838474

5a

Intensity data were collected on a Bruker D8 Venture kappa diffractometer equipped with a Photon 100 CMOS detector. An Iμs microfocus Cu source (λ = 1.54178 Å) coupled with a multi-layer mirror monochromator provided the incident beam. The sample was mounted on a 0.3 mm loop with the minimal amount of Paratone-N oil. Data was collected as a series of ϕ and/or ω scans. Data was collected at 100 K using a cold stream of $N_{2(g)}$. The collection, cell refinement, and integration of intensity data was carried out with the APEX2 software.[28] A semi-empirical absorption correction was performed with SADABS.[29] The structure was phased with direct methods using SHELXS[30] and refined with the full-matrix least-squares program SHELXL.[30]

A structural model consisting of the target molecule was developed. There is no disorder in the structure.

Methyl H atom positions, R-CH_3, were optimized by rotation about R-C bonds with idealized C-H, R--H and H--H distances. Remaining H atoms were included as riding idealized contributors. Methyl H atom U's were assigned as 1.5 times U_{eq} of the carrier atom; remaining H atom U's were assigned as 1.2 times carrier U_{eq}.

On the basis of 1548 unmerged Friedel opposites, the fractional contribution of the racemic twin was negligible.[33,34] The absolute structure parameter y was calculated using PLATON.[32] The resulting value was y=0.00(5) indicating that the absolute structure has probably been determined correctly.[35]

CCDC: 1838475

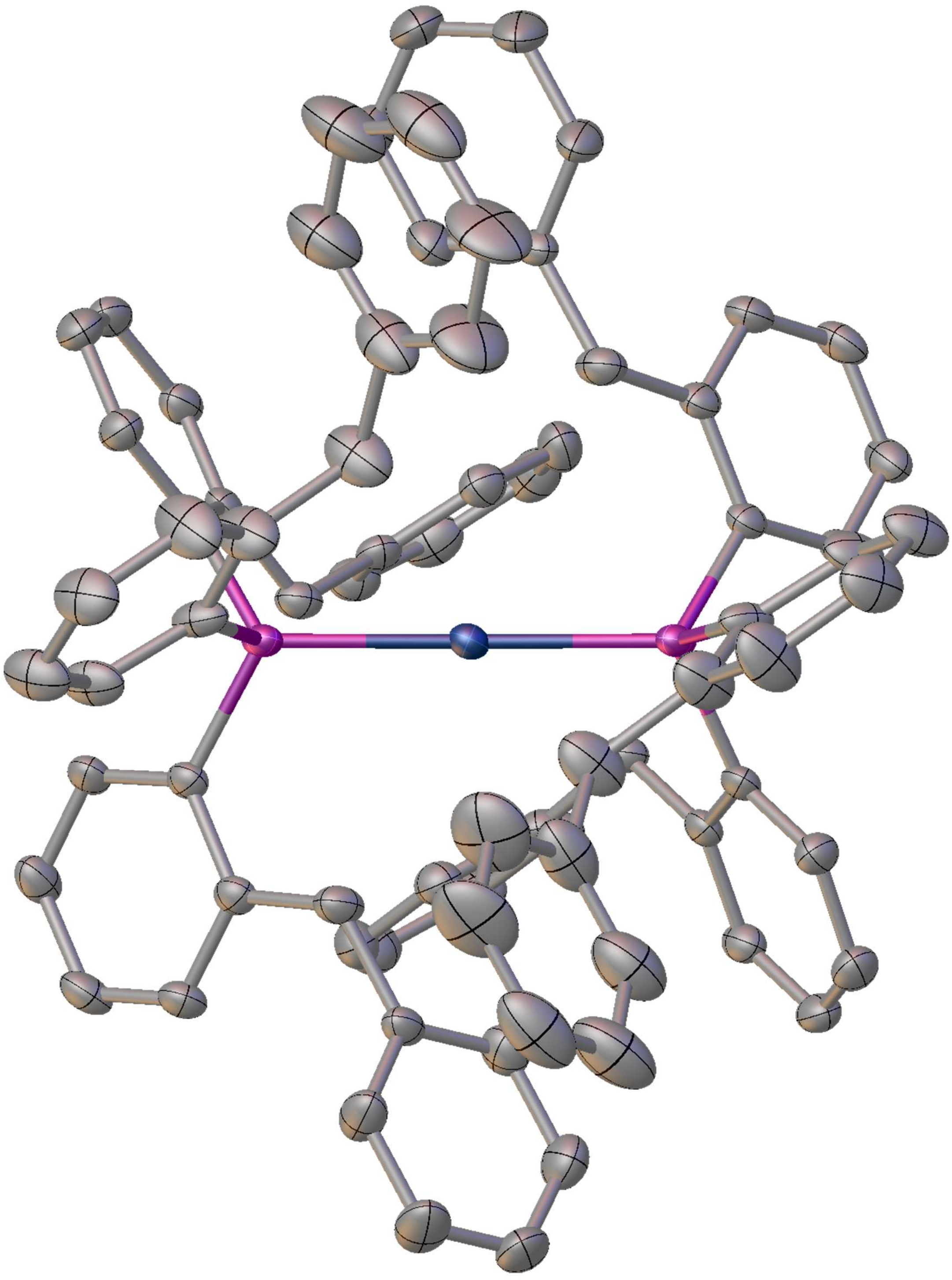

Supplementary Figure 7| X-ray structure of compound **SI-45**.

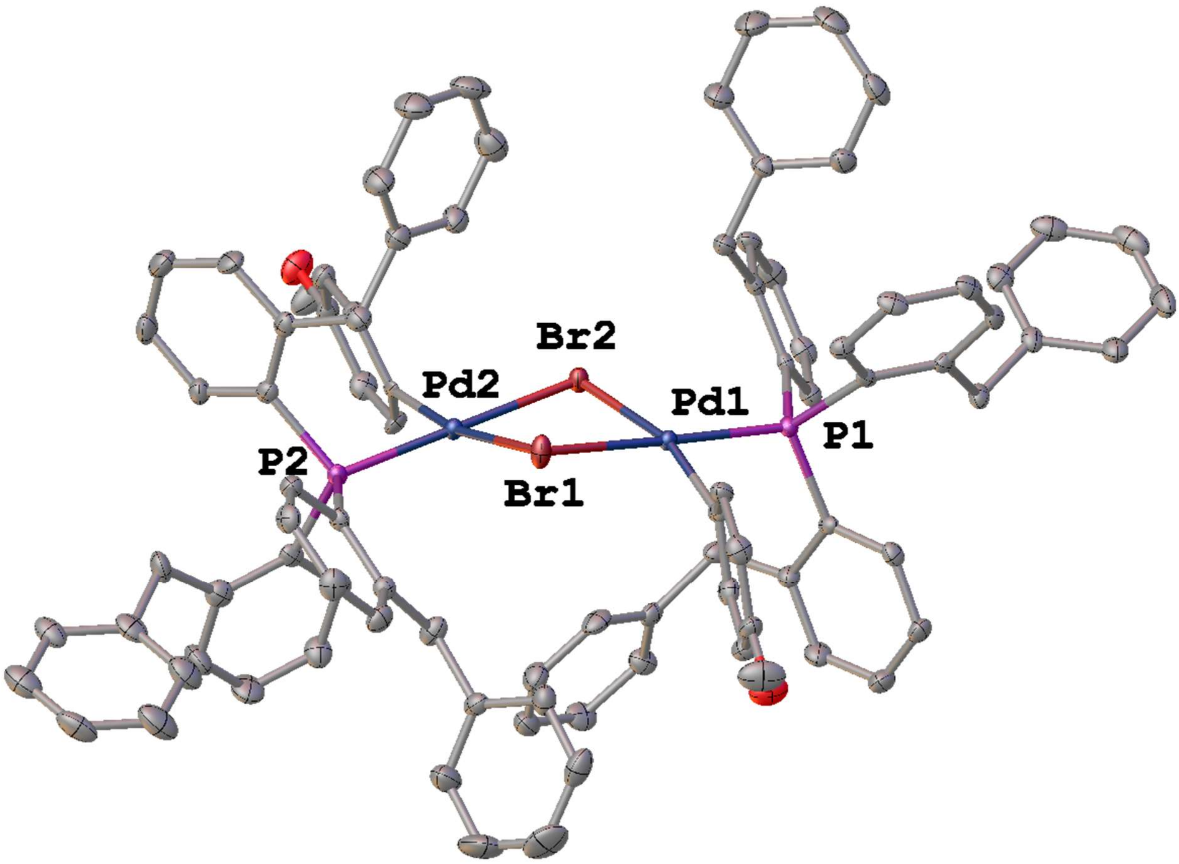

Supplementary Figure 8| X-ray structure of compound **SI-46**.

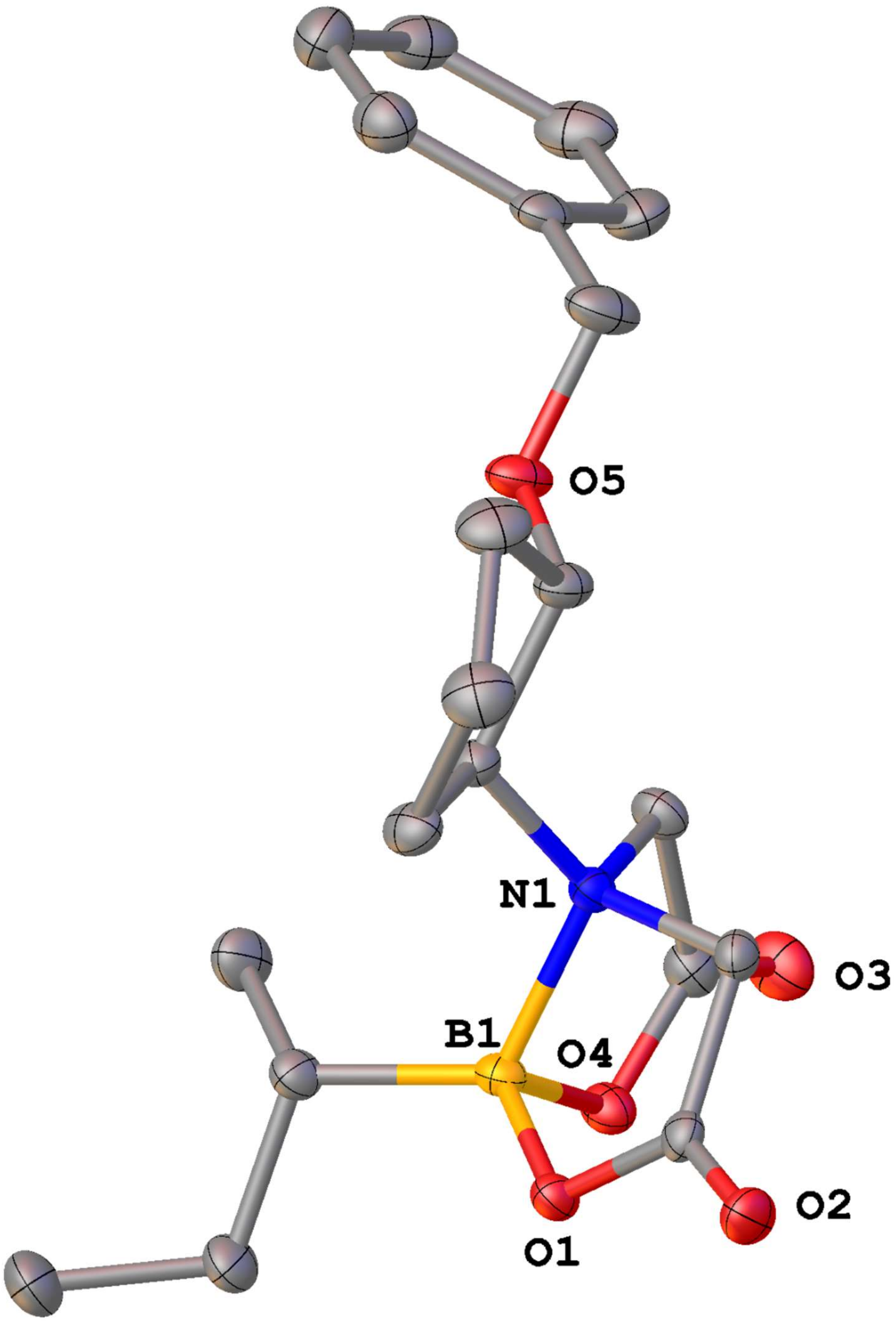

Supplementary Figure 9| X-ray structure of compound **5a**.

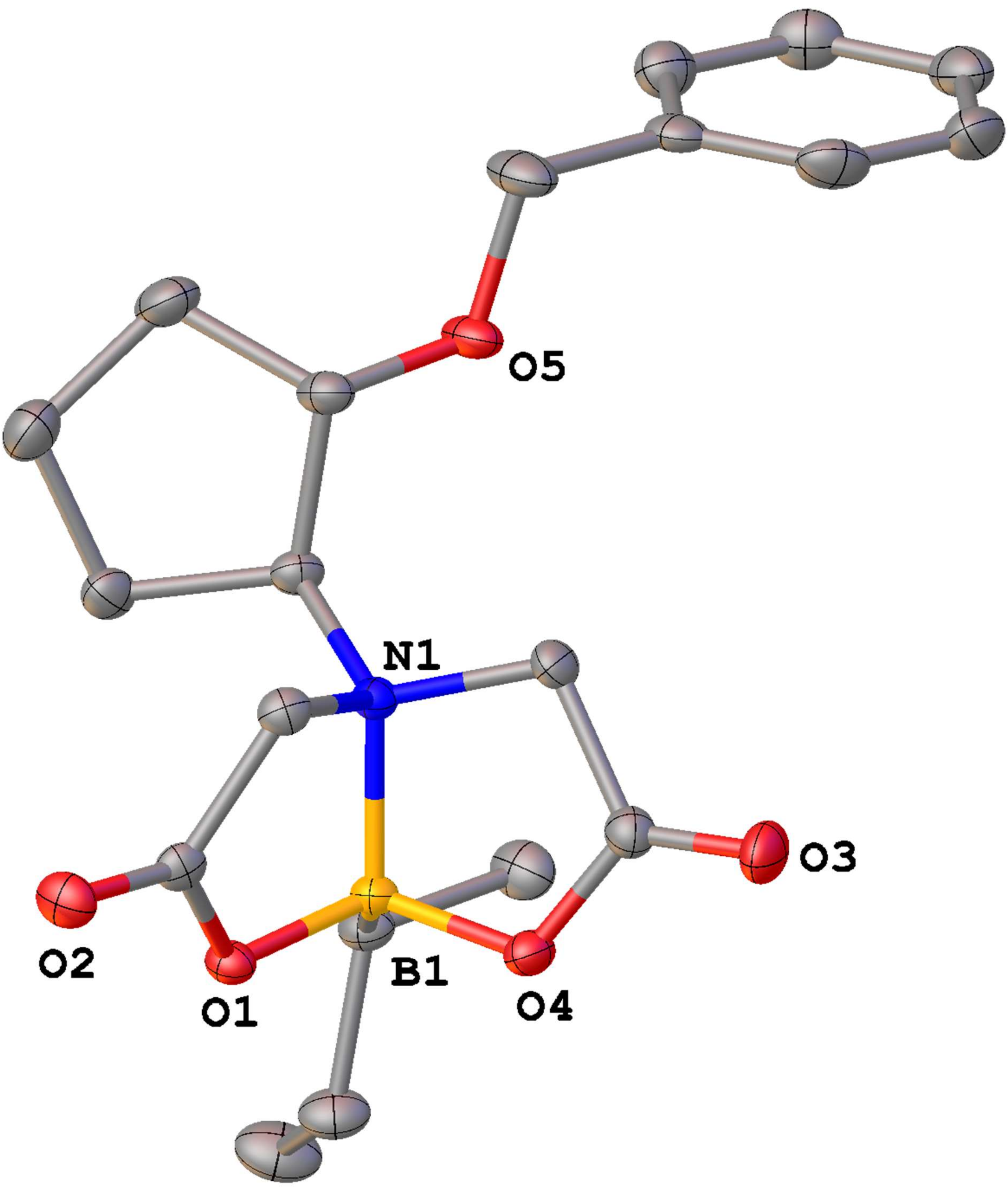

Supplementary Figure 10| X-ray structure of compound **5a** (alternate angle).

XI. Assay to Test for Racemization of Boronic Acids

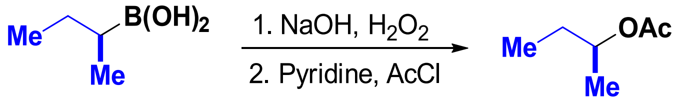

(*S*)-1a **SI-47**

A solution of 1 mmol of **(*S*)-1a**, produced by hydrolysis of BIDA boronate **5a** of ≥99:1 d.r. following general procedures B and C, was diluted to 0.5 Molar in dioxane in a 7 mL vial in an ice bath. To this was added 30% H_2O_2 (0.227 g, 2.0 mmol, 2.0 eq) and 1 Molar NaOH (2.0 mL, 2.0 mmol, 2.0 eq). The mixture was stirred until complete conversion of the boronic acid was seen by TLC (1:1 Hex/EtOAc, $KMnO_4$). 1 Molar HCl (5 mL) and Et_2O (5 mL) were then added, and the organic layer was washed with saturated sodium bisulfate, H_2O, and brine. It was dried with sodium sulfate and concentrated to a 2mL volume (a small aliquot was removed, the Et_2O was removed under an air stream, and the sample was analyzed by ^{1}H-NMR, showing the presence of 2-butanol). To this solution was then added pyridine (0.242 mL, 0.238 g, 3.00 mmol, 3.00 eq) followed by the dropwise addition of acetyl chloride (0.213 mL, 0.234 g, 3.00 mmol, 3.00 eq). The heterogeneous mixture was capped and stirred 8 hours at 23 °C. The mixture was washed three times with 1 Molar HCl, once with saturated $NaHCO_3$, and once with brine. The solvent was removed under light vacuum to yield 25 mg of 2-butylacetate (**SI-47**), confirmed by 1H NMR) as a colorless oil. This 2-butylacetate (**SI-47**) was determined by chiral GC to be of ≥99:1 e.r. (Agilent chiral G-TA column was used, with 0.8mL/minute gas flow, 24°C to 55°C at 1°C/minute. Major: 20.6, Minor: 22.1), indicating that no racemization took place during the synthesis of **(*S*)-1a**.

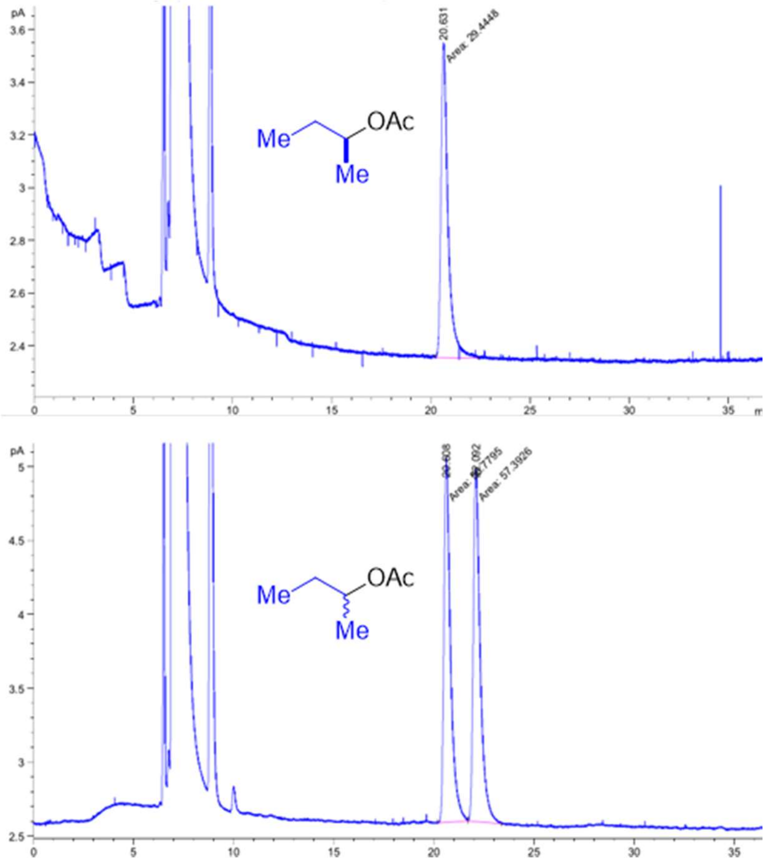

XII. Stability Tests

The long-term bench top stability of these compounds was determined as follows. Two 2 mL Teflon-lined screw-cap vials were each filled with 10 mg of MIDA boronate **(±)-6a**. Similarly, two of these vials were filled with 15 mg of BIDA boronate **5a** of 99:1 dr and two were filled with 10 mg of trihydroxyborate salt **(±)-7a**. 0.5 mL of a solution of 1.15M **(±)-1a** in dioxane, prepared by general procedure C, was added to one vial. Each compound was initially quantified by adding 0.5 mL of DMSO-d_6 with 0.10 Molar 1,4-dimethoxybenzene standard to one of the vials containing **(±)-6a**, **5a** or **(±)-7a**. The solutions were transferred to NMR tubes. 50 µl of the solution of **(±)-1a** was also added to an NMR tube with 0.5 mL of DMSO-d_6 with 0.1 Molar 1,4-dimethoxybenzene standard. Mmol of compound was determined by ^{1}H-NMR integration with a relaxation delay of 10 seconds. The remaining vials were tightly capped under air and stored on the bench top for 4 months. Then, the NMR quantification was repeated. All three solid compounds showed no decomposition. The concentration of **(±)-1a** decreased by <10% (1.05M), with small amounts of decomposition products present in the spectrum.

Compound	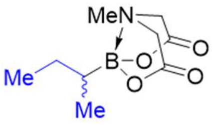 **(±)-6a**	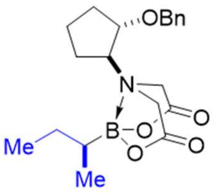 **5a**, ≥99:1 d.r.	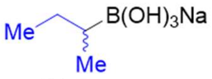 **(±)-7a**	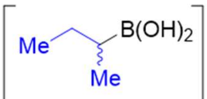 **(±)-1a** 1.16 Molar in dioxane
Result at 4 months	No decomposition	No decomposition	No decomposition	Concentration decreased to 1.06 Molar

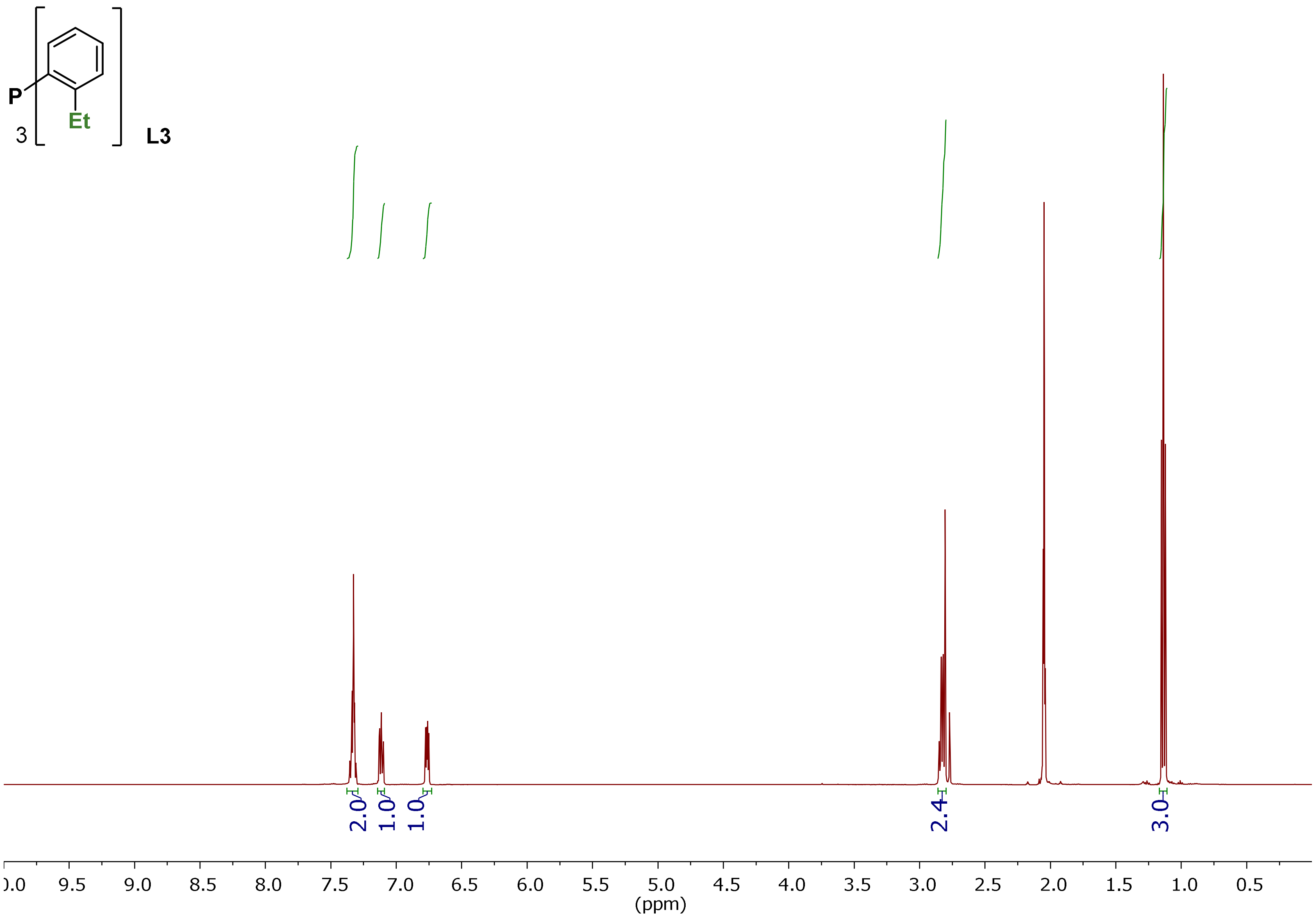

Supplementary Figure 11| ^{1}H-NMR spectrum (500 MHz, acetone-*d6*) for **L3**.

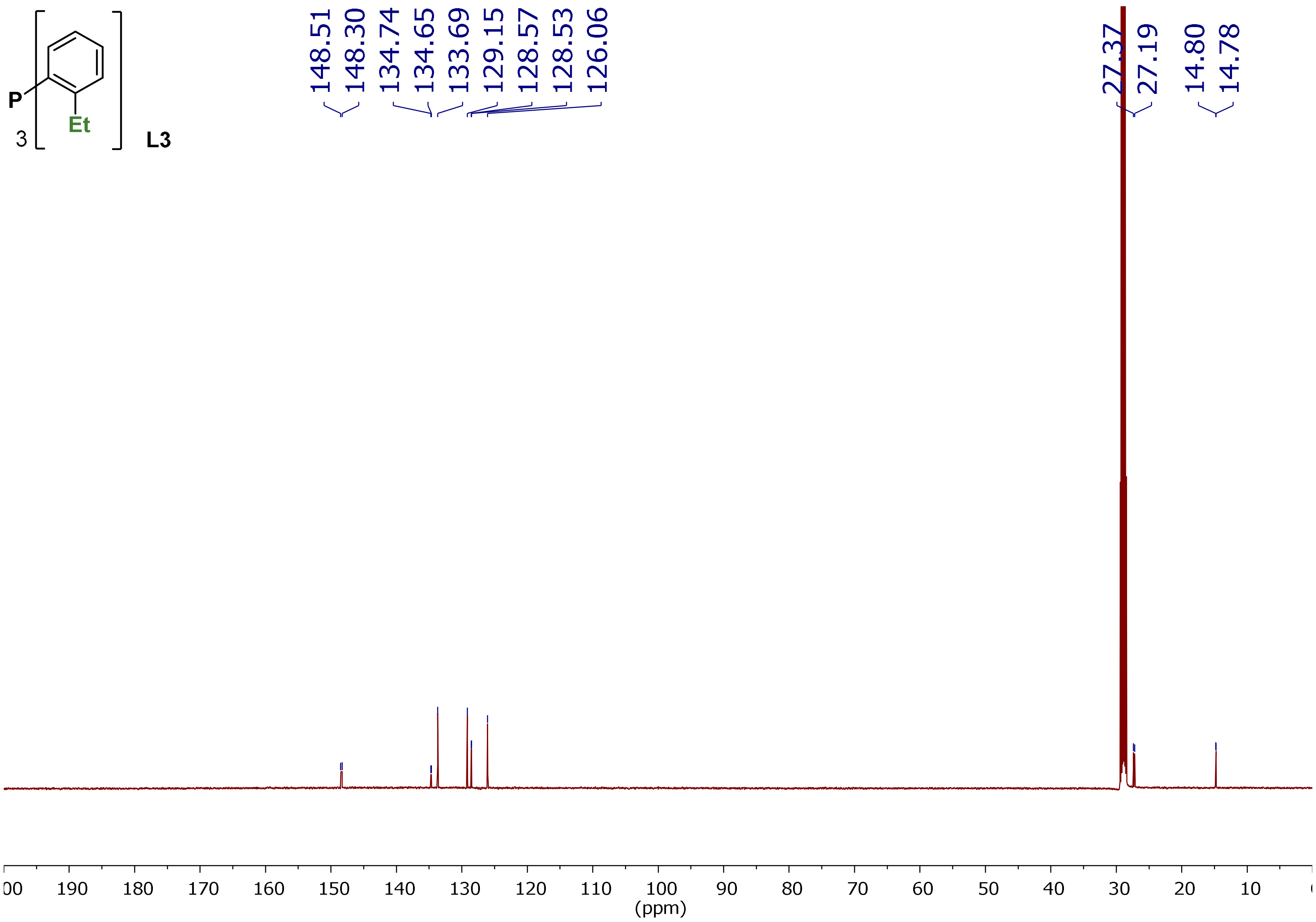

Supplementary Figure 12| ^{13}C-NMR spectrum (126 MHz, acetone-*d6*) for **L3**.

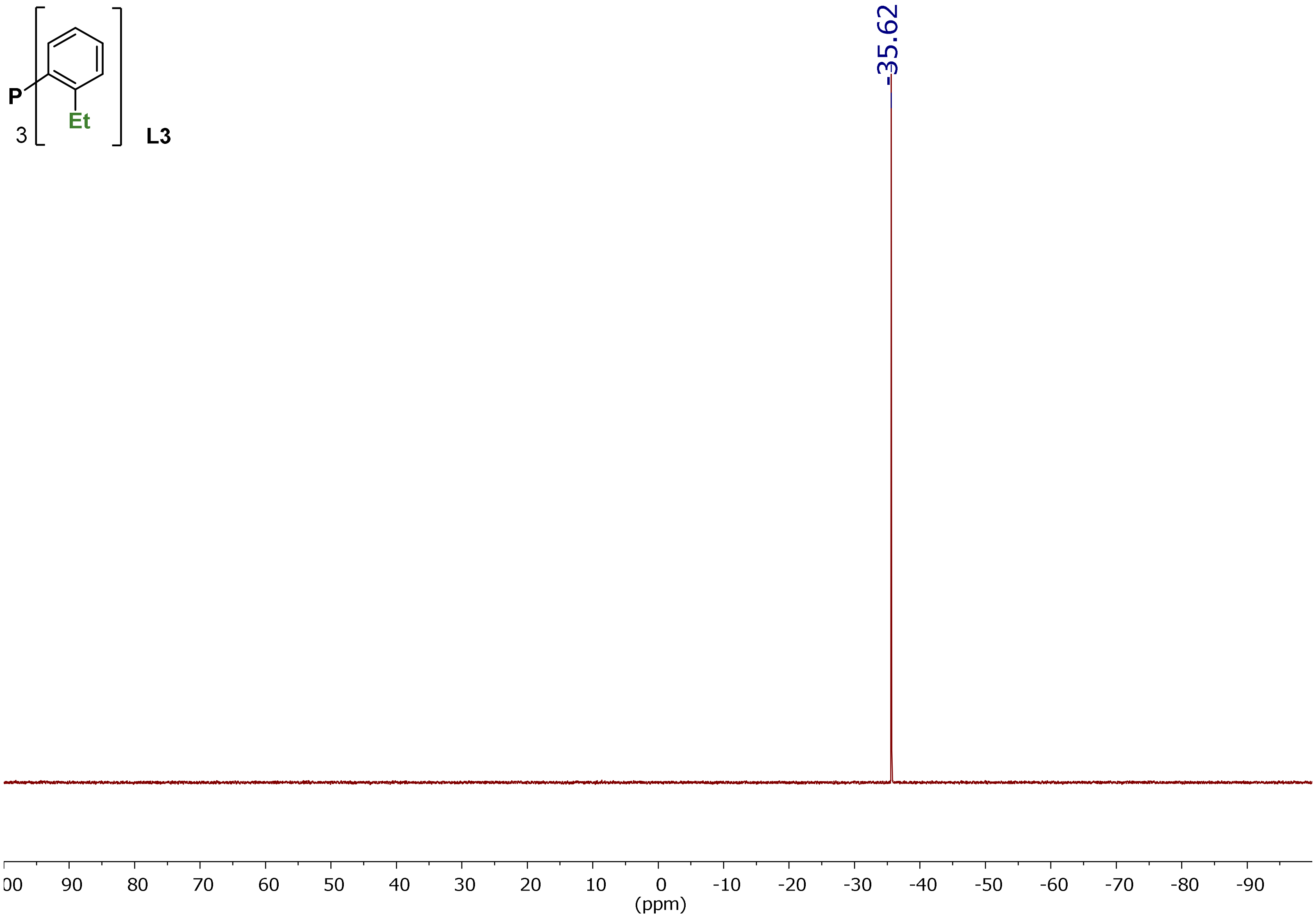

Supplementary Figure 13| ^{31}P-NMR spectrum (202 MHz, acetone-*d6*) for **L3**.

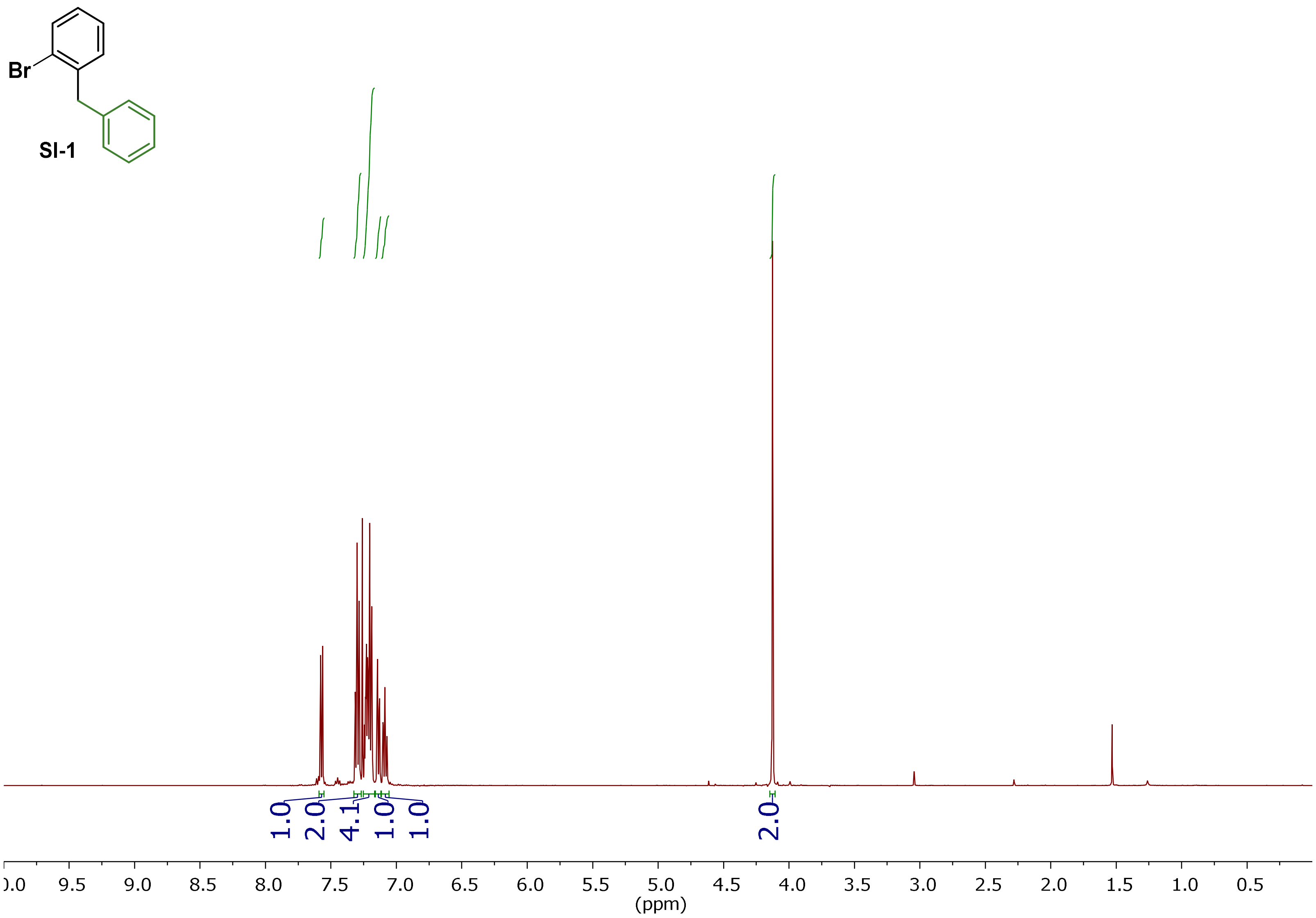

Supplementary Figure 14| ^{1}H-NMR spectrum (500 MHz, $CDCl_3$) for **SI-1**.

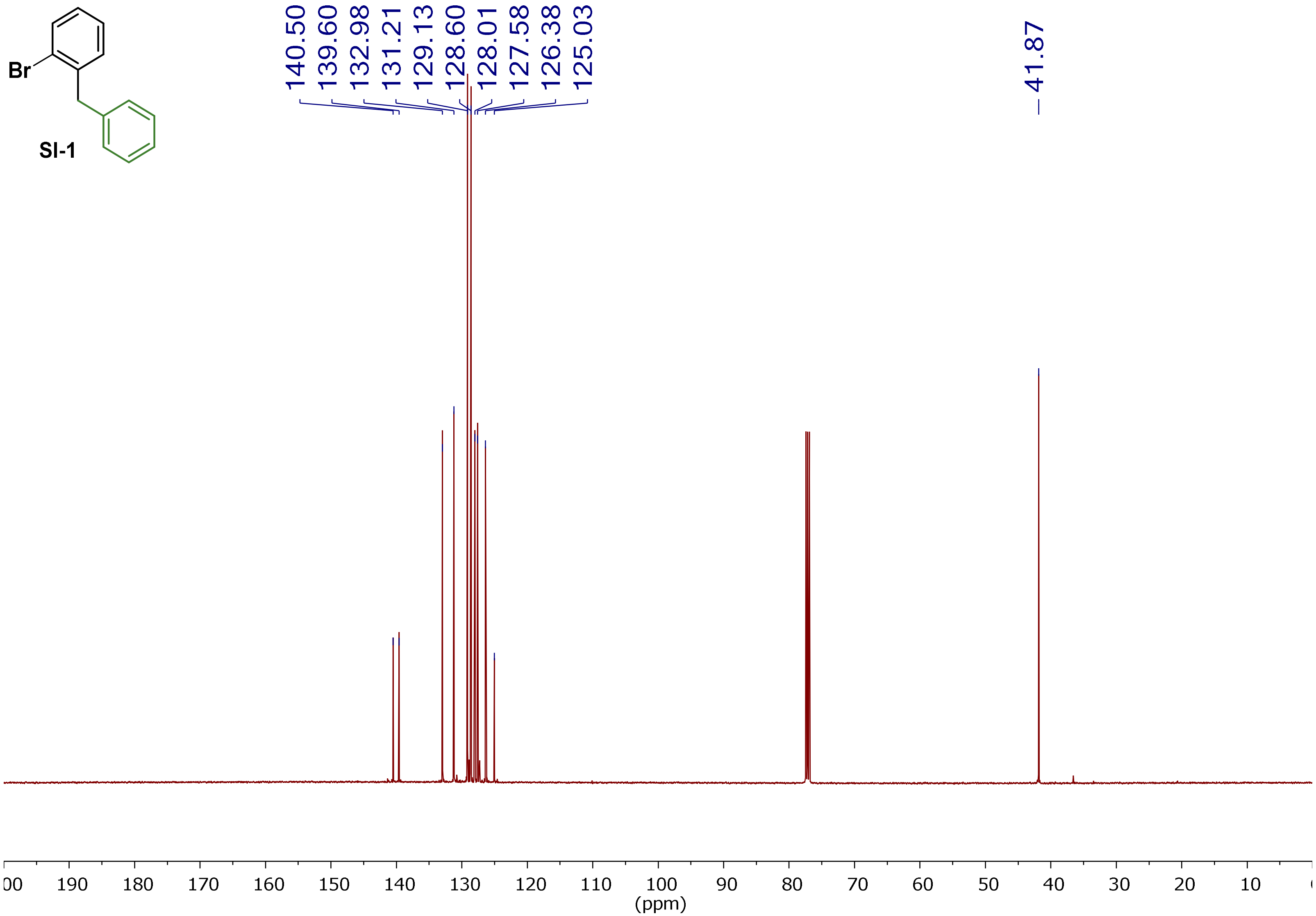

Supplementary Figure 15| ^{13}C-NMR spectrum (126 MHz, $CDCl_3$) for **SI-1**.

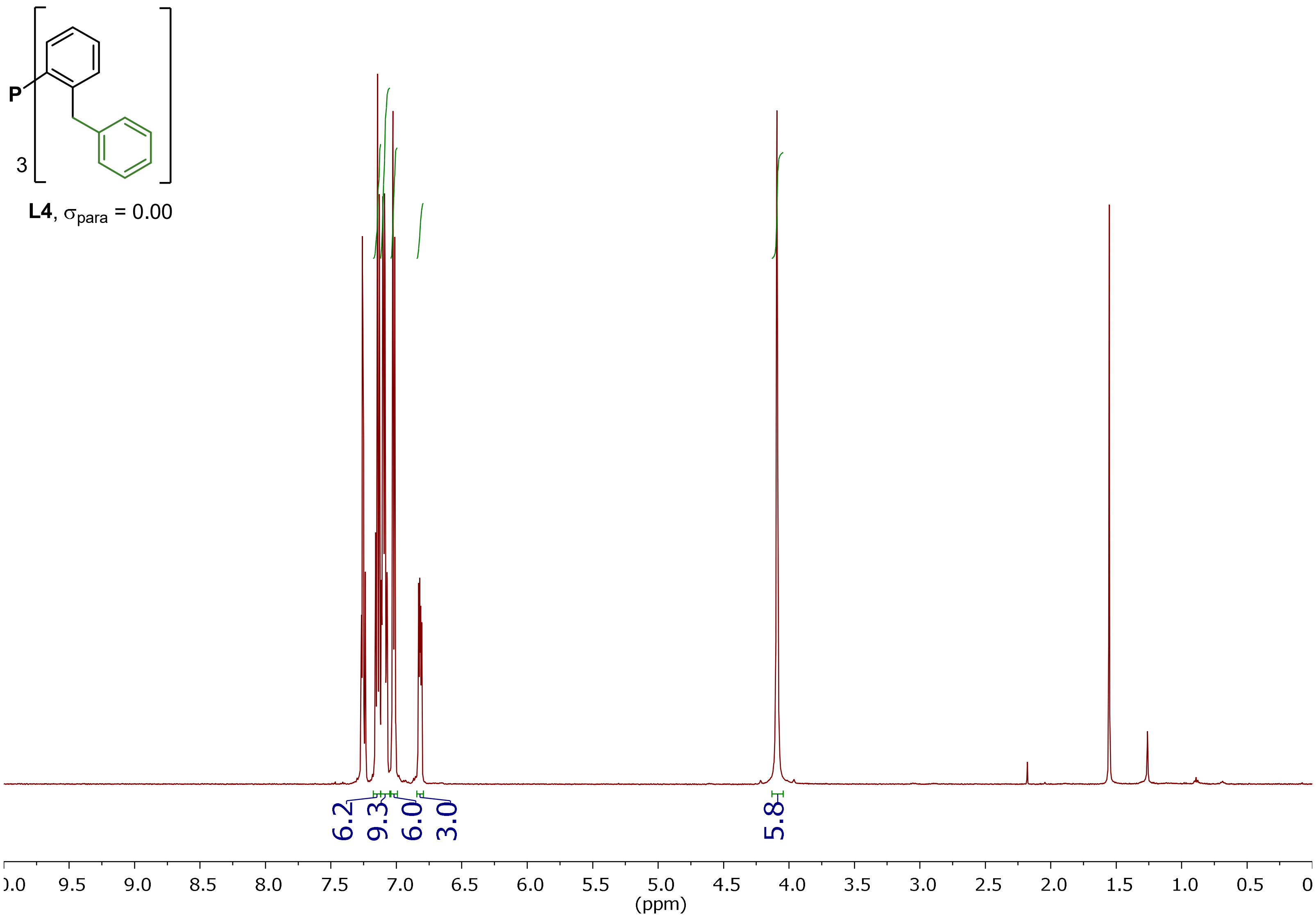

Supplementary Figure 16| ^{1}H-NMR spectrum (500 MHz, $CDCl_3$) for **L4**.

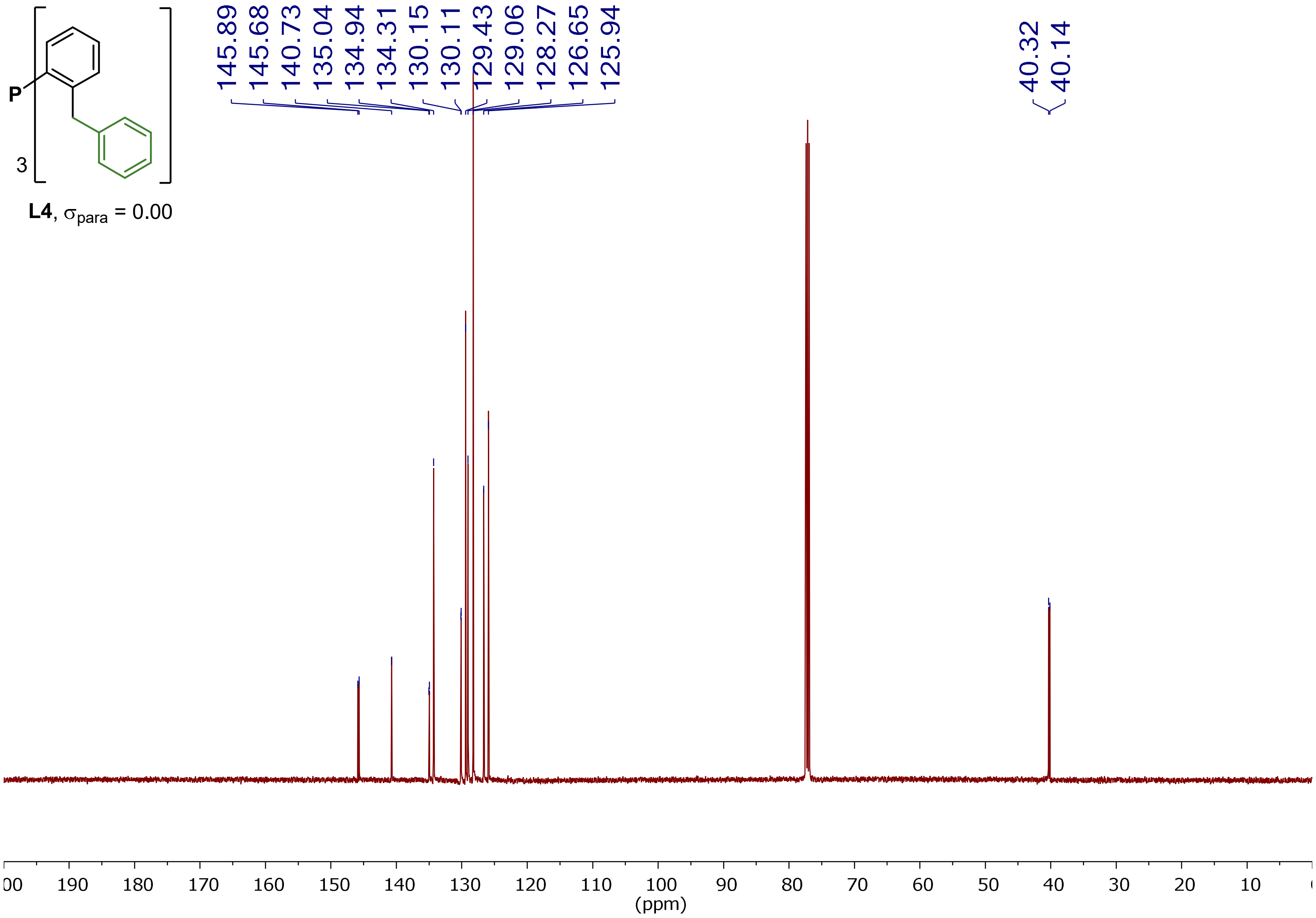

Supplementary Figure 17| ^{13}C-NMR spectrum (126 MHz, $CDCl_3$) for **L4**.

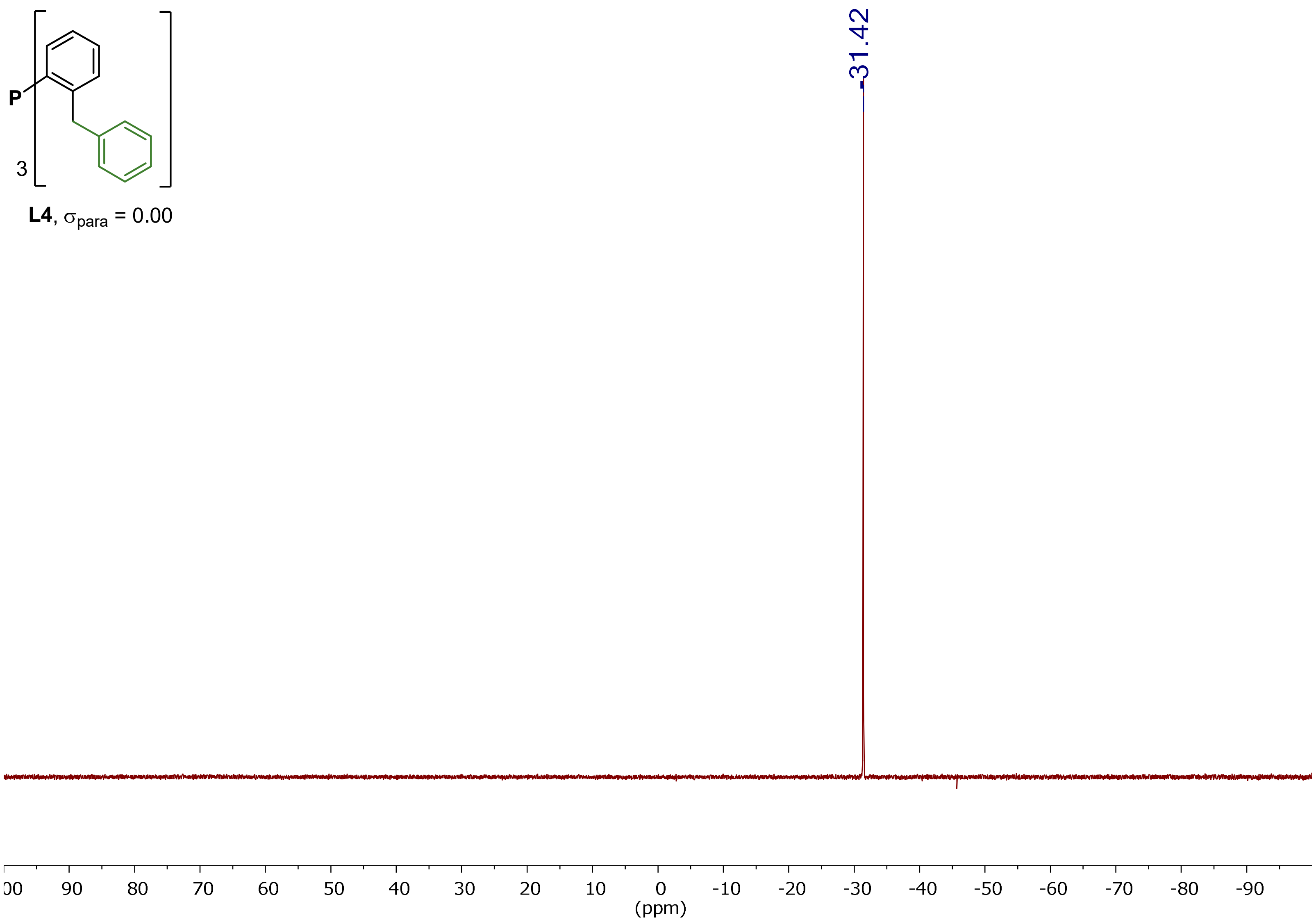

Supplementary Figure 18| ^{31}P-NMR spectrum (202 MHz, $CDCl_3$) for **L4**.

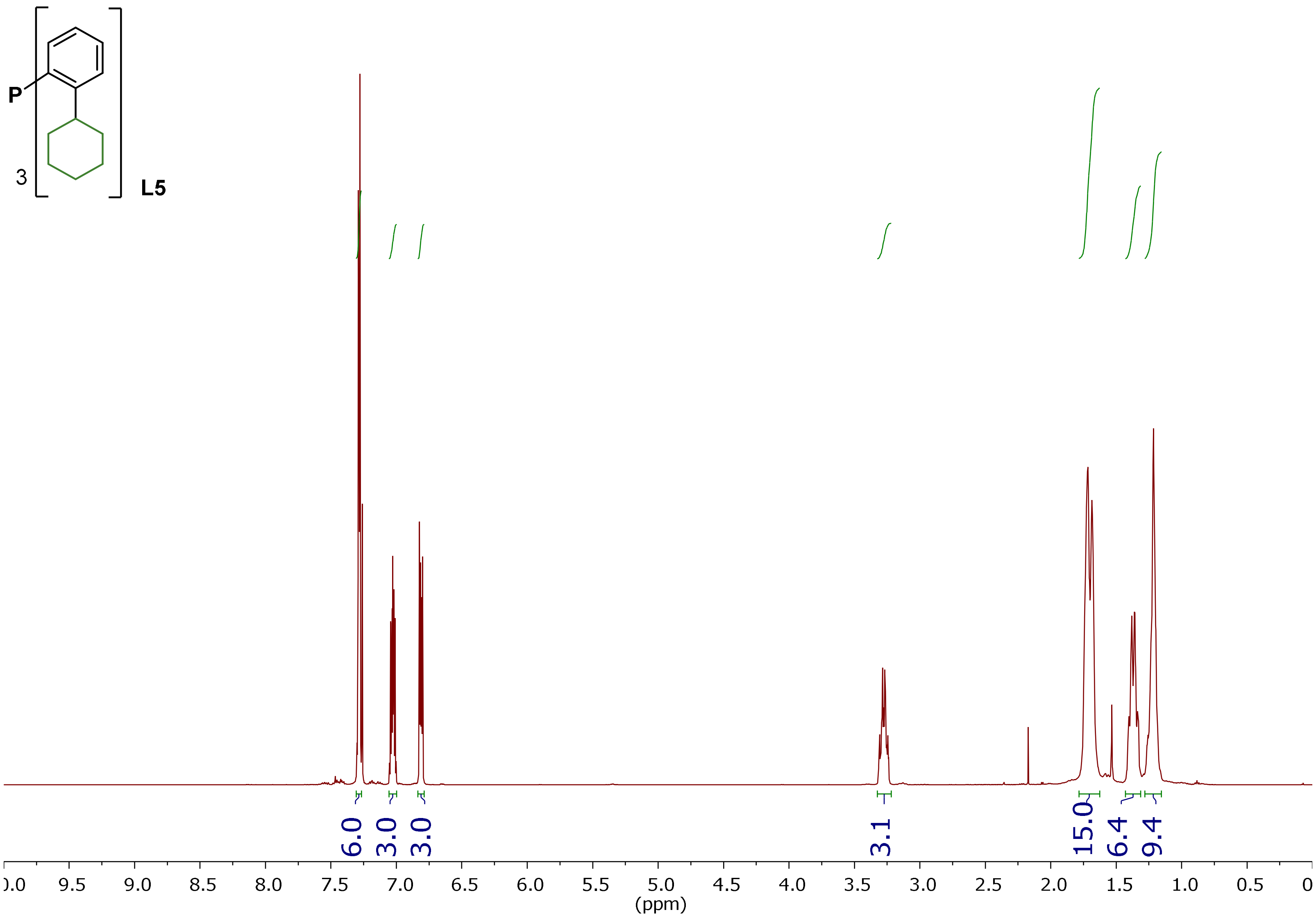

Supplementary Figure 19| ^{1}H-NMR spectrum (500 MHz, $CDCl_3$) for **L5**.

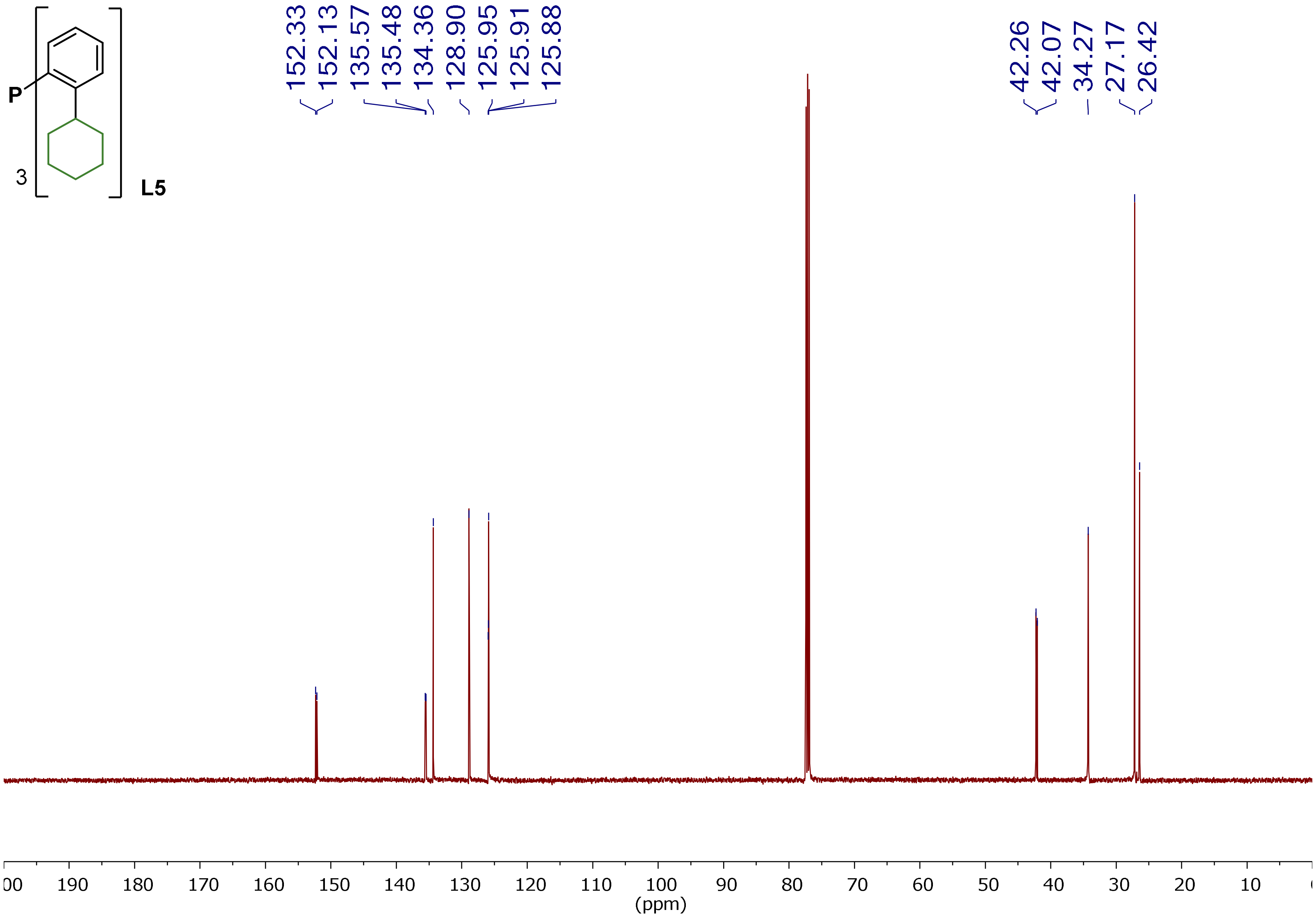

Supplementary Figure 20| ^{13}C-NMR spectrum (126 MHz, $CDCl_3$) for **L5**.

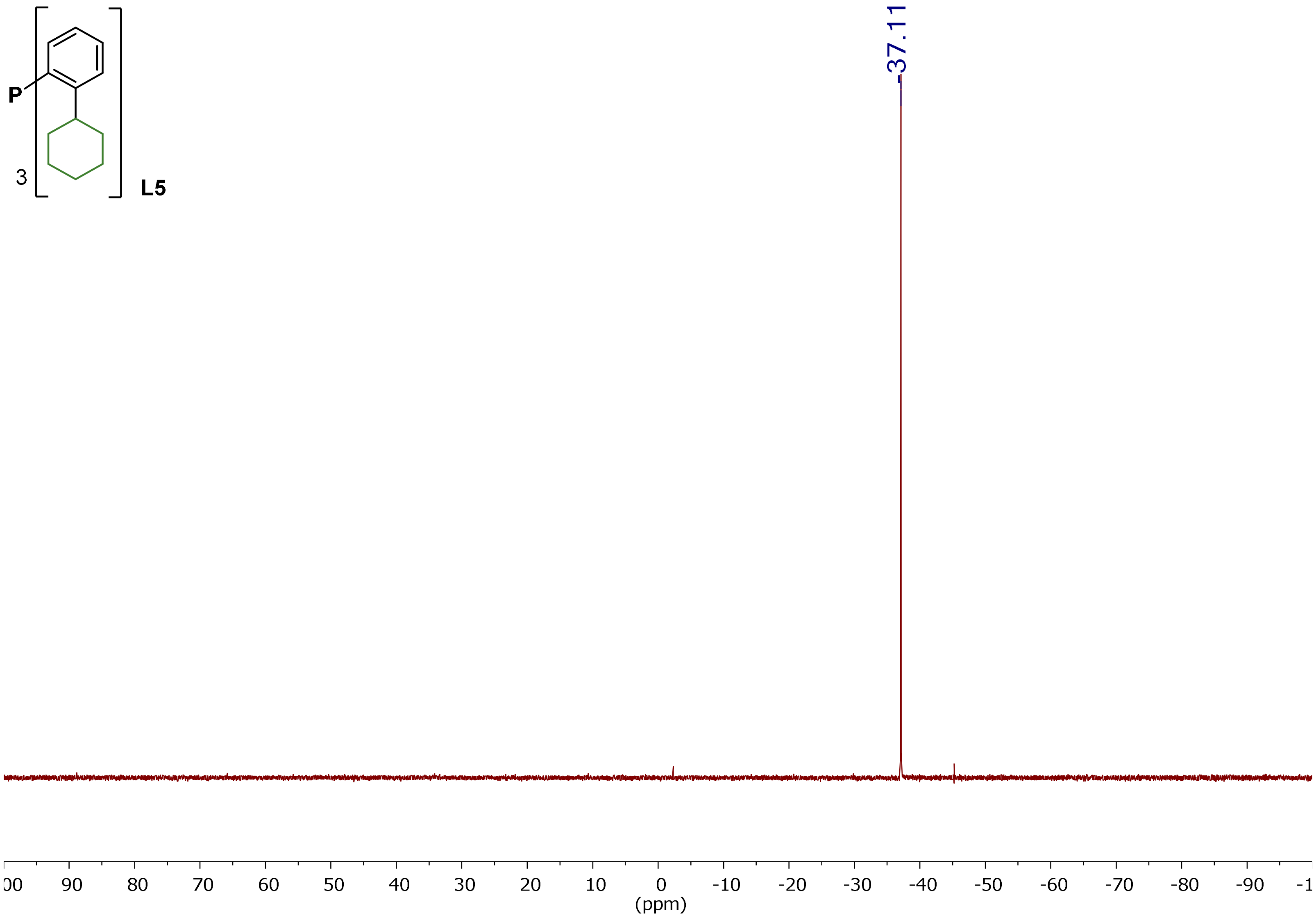

Supplementary Figure 21| ^{31}P-NMR spectrum (202 MHz, $CDCl_3$) for **L5**.

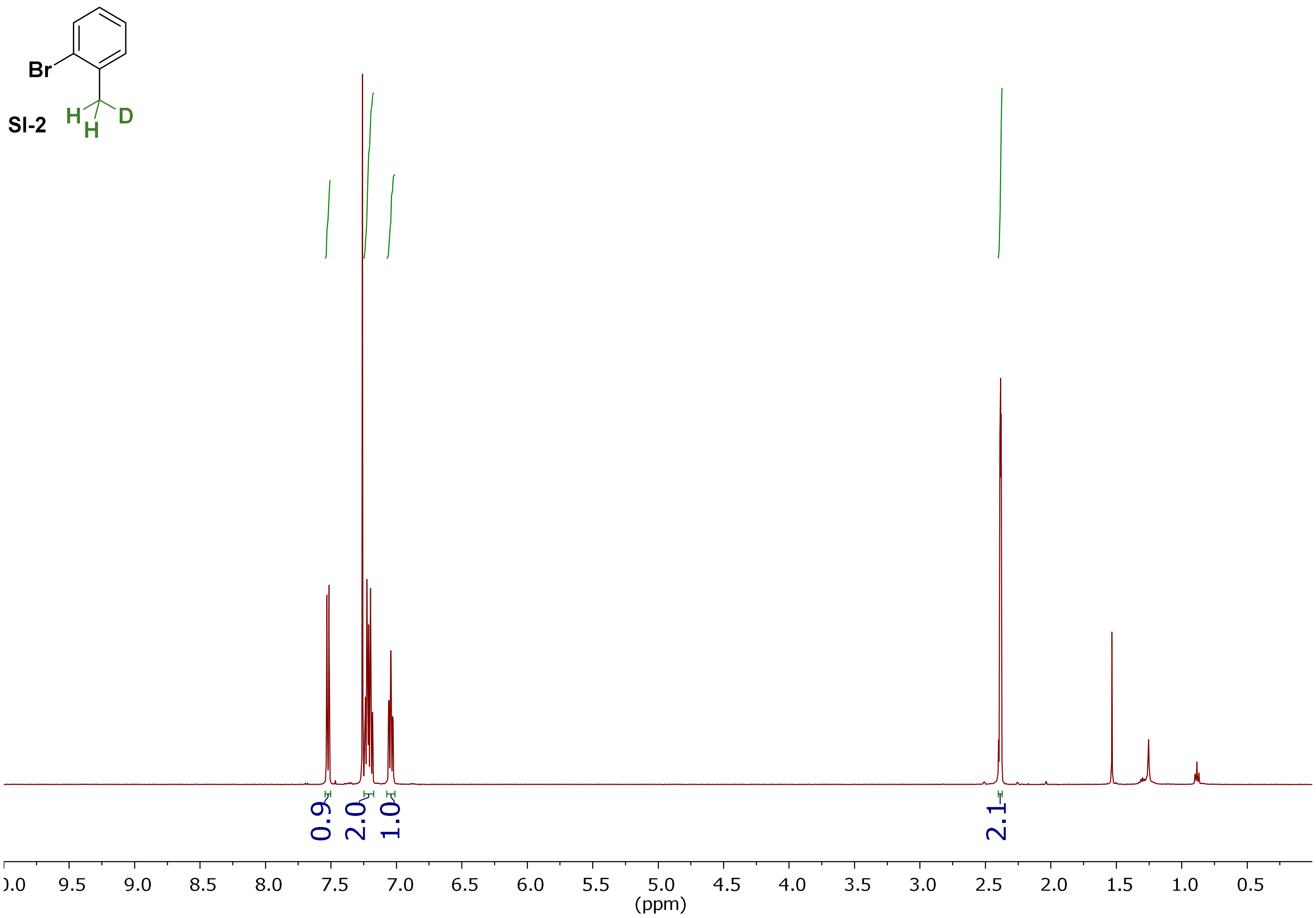

Supplementary Figure 22| ^{1}H-NMR spectrum (500 MHz, $CDCl_3$) for **SI-2**.

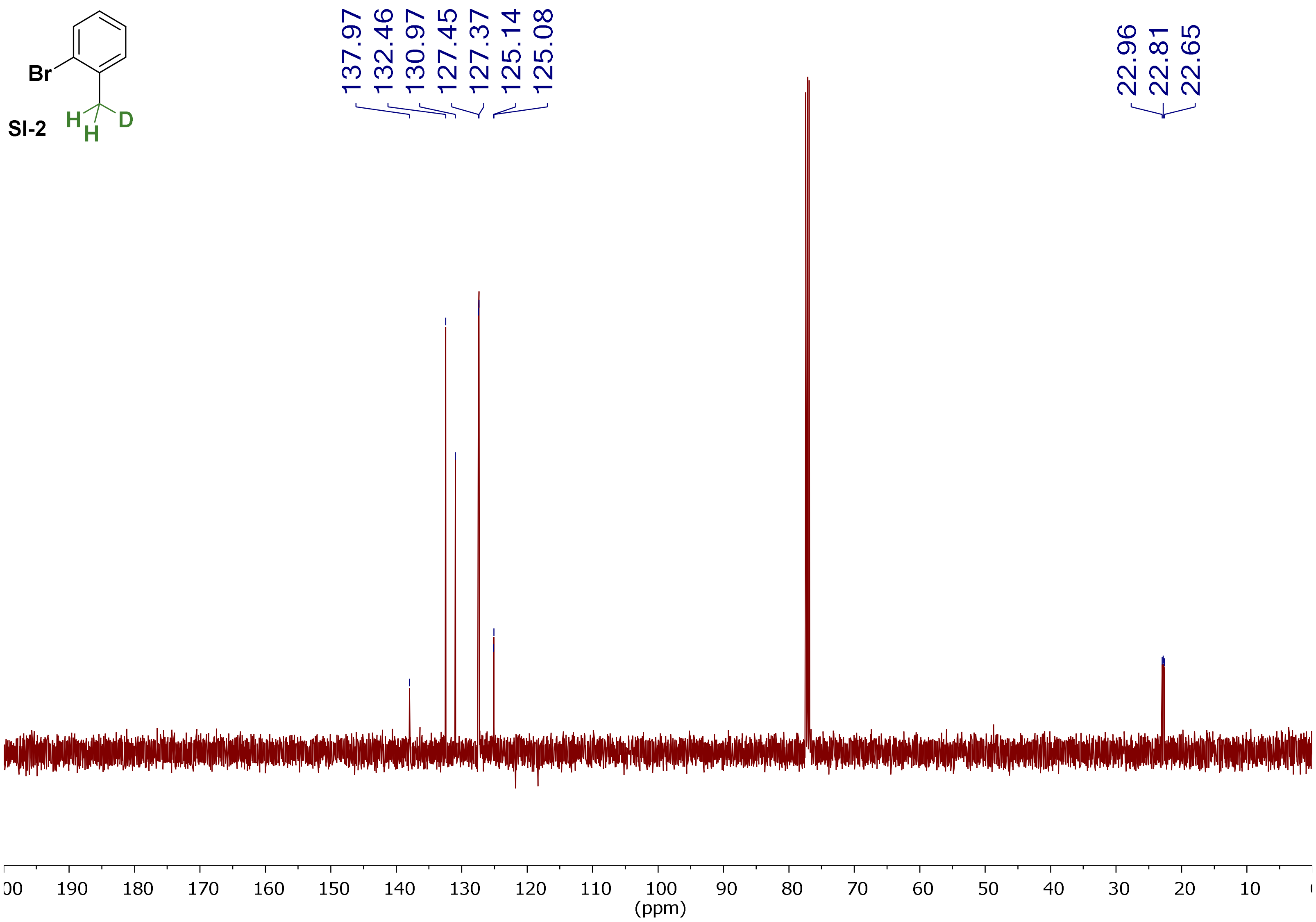

Supplementary Figure 23| ^{13}C-NMR spectrum (126 MHz, $CDCl_3$) for **SI-2**.

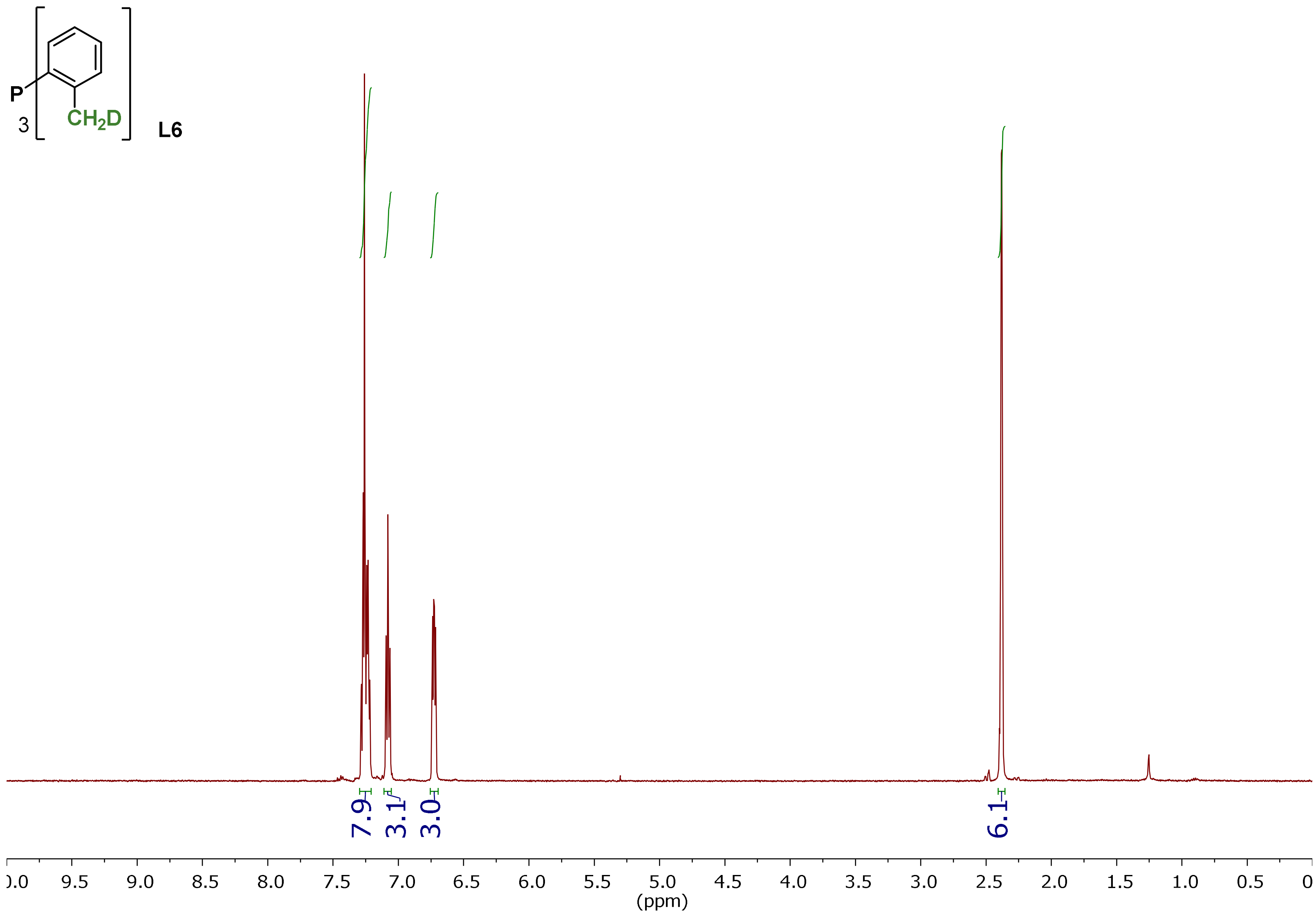

Supplementary Figure 24| ^{1}H-NMR spectrum (500 MHz, $CDCl_3$) for **L6**.

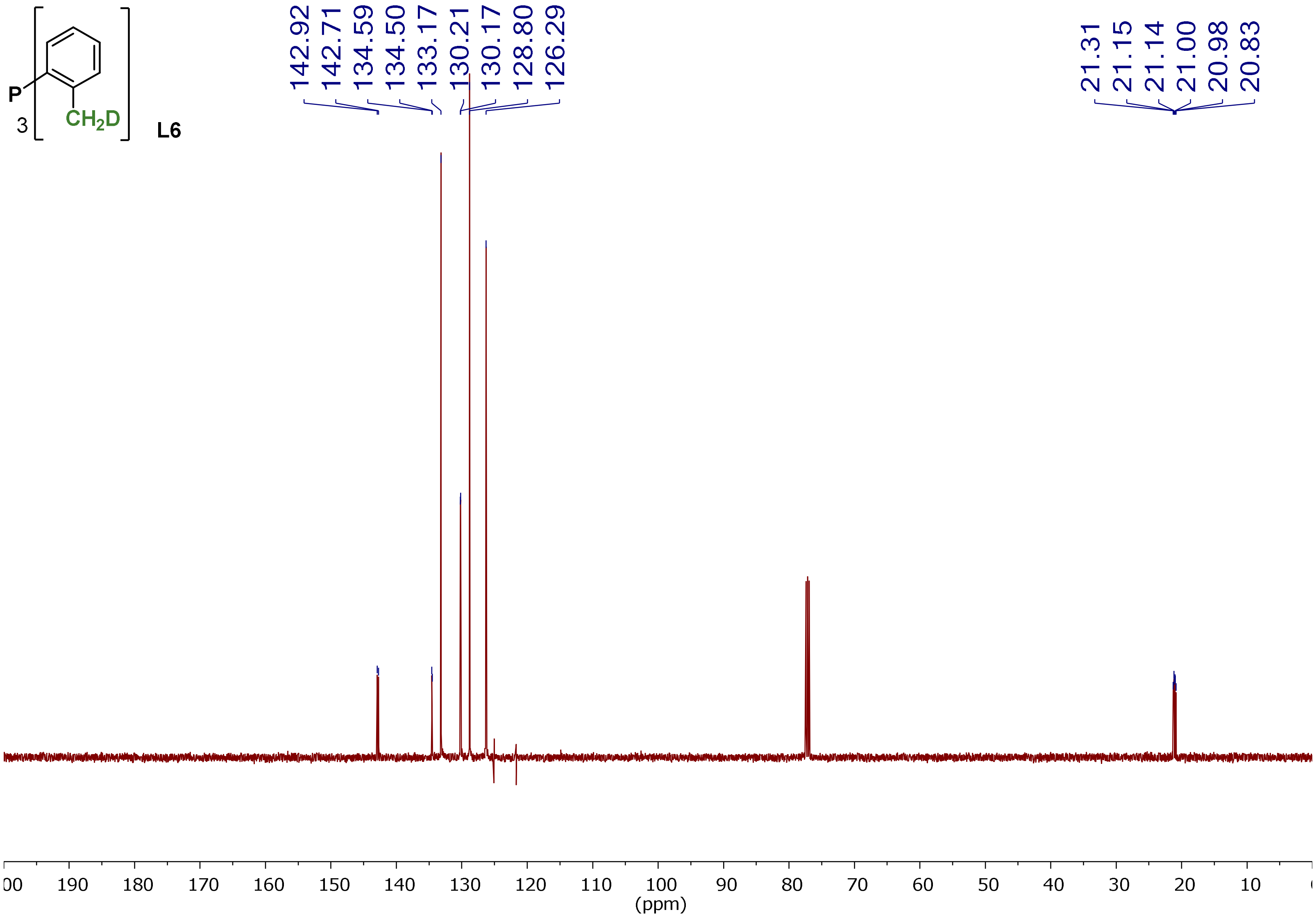

Supplementary Figure 25| ^{13}C-NMR spectrum (126 MHz, $CDCl_3$) for **L6**.

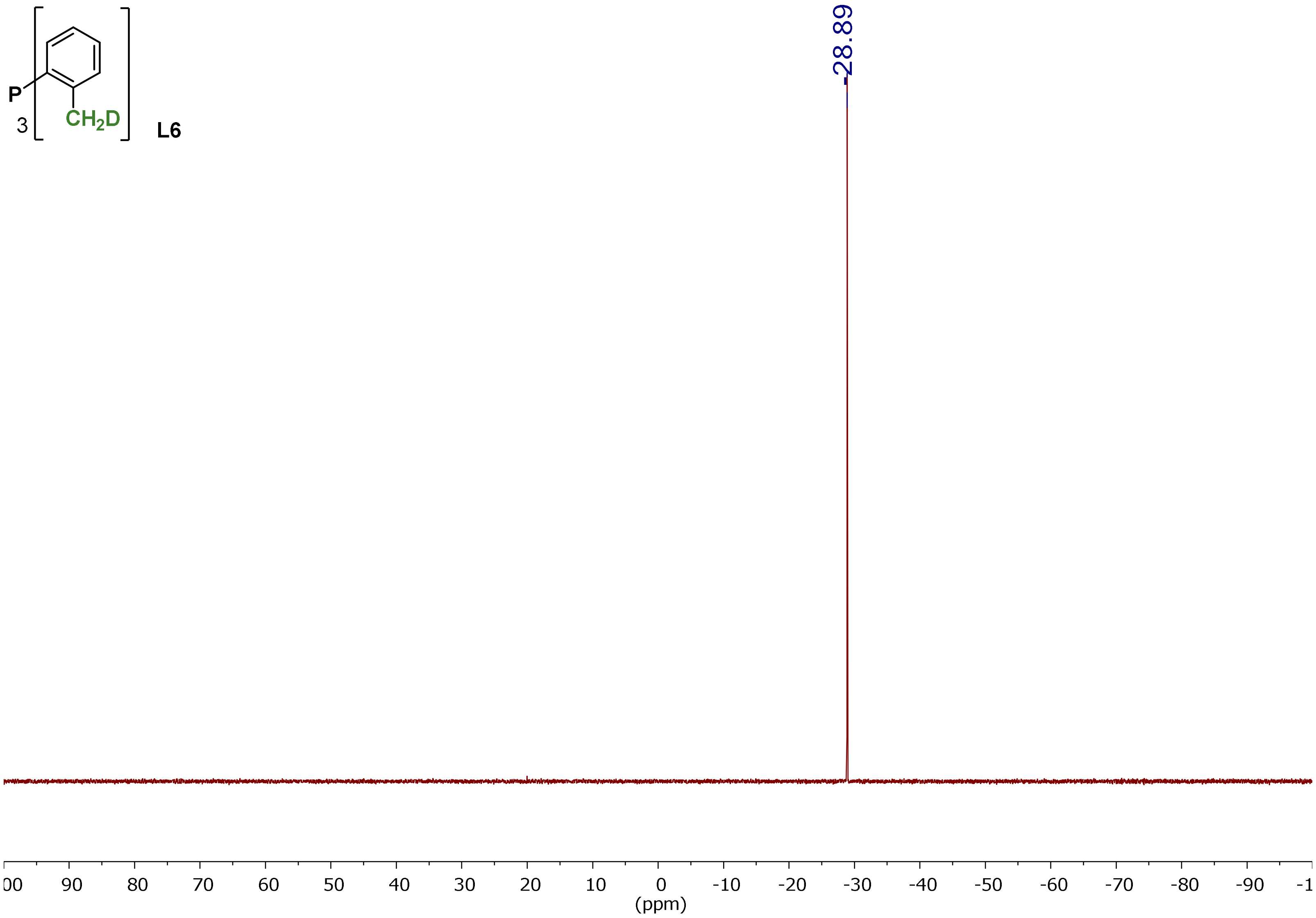

Supplementary Figure 26| ^{31}P-NMR spectrum (202 MHz, $CDCl_3$) for **L6**.

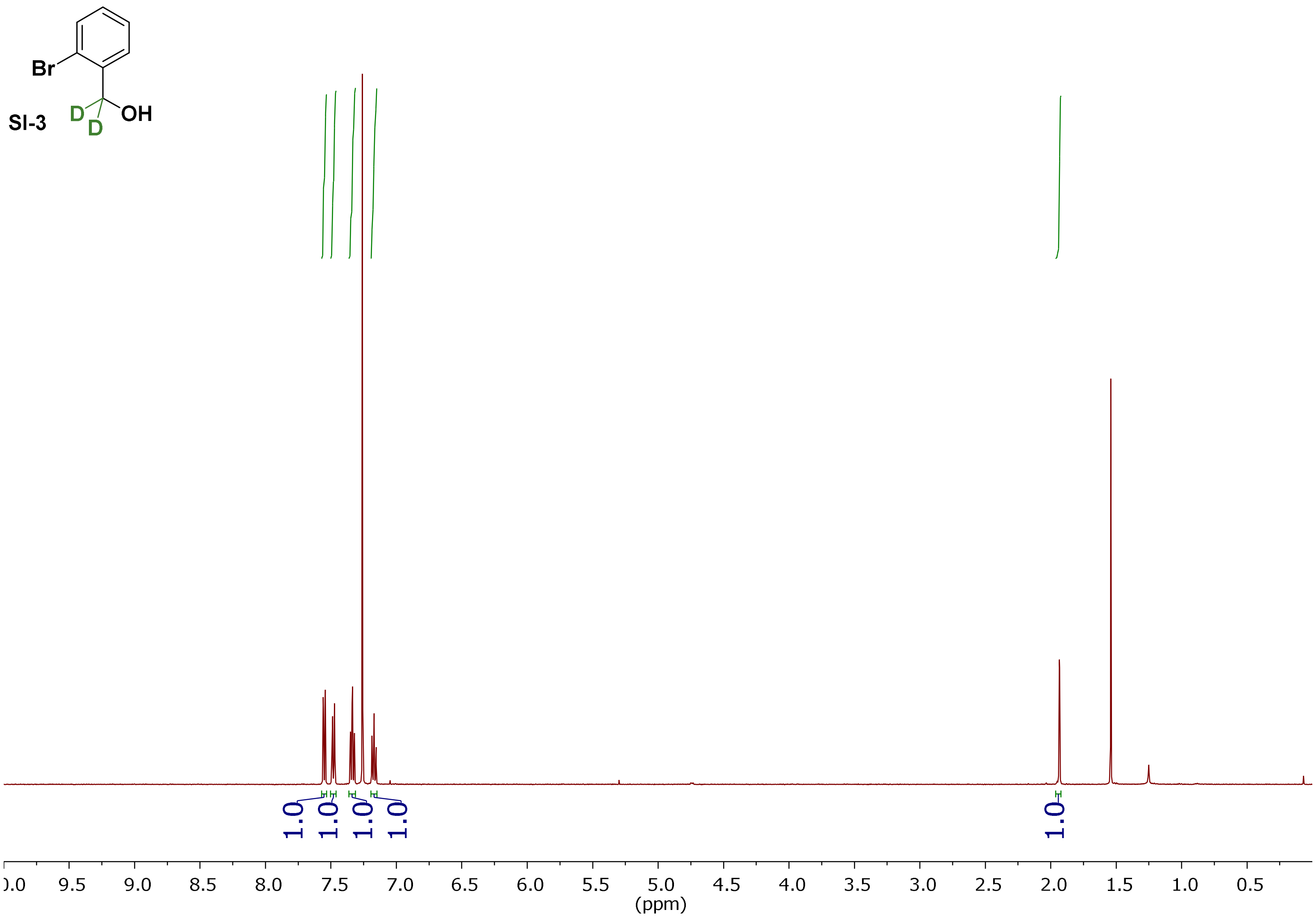

Supplementary Figure 27| ^{1}H-NMR spectrum (500 MHz, $CDCl_3$) for **SI-3**.

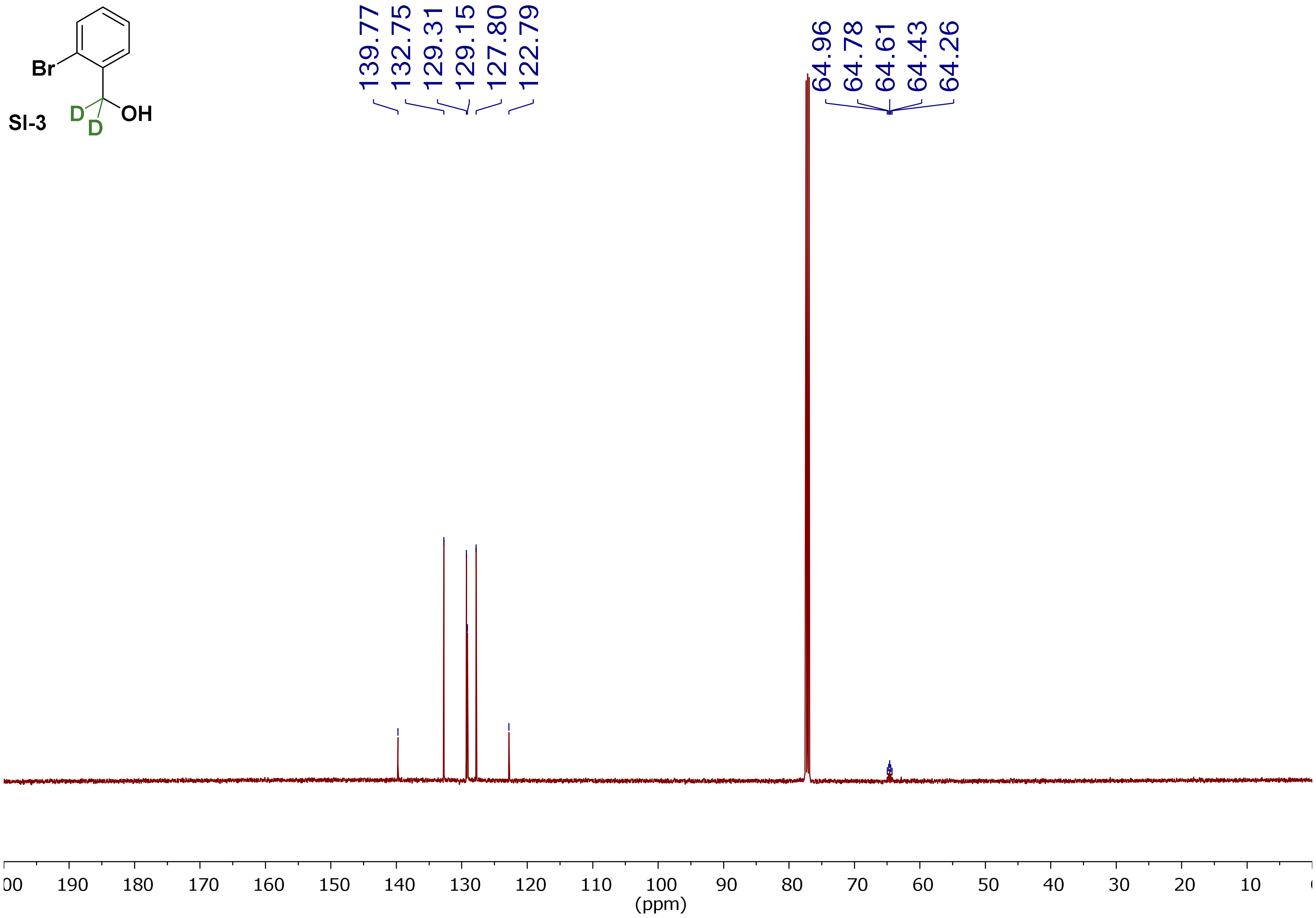

Supplementary Figure 28| ^{13}C-NMR spectrum (126 MHz, $CDCl_3$) for **SI-3**.

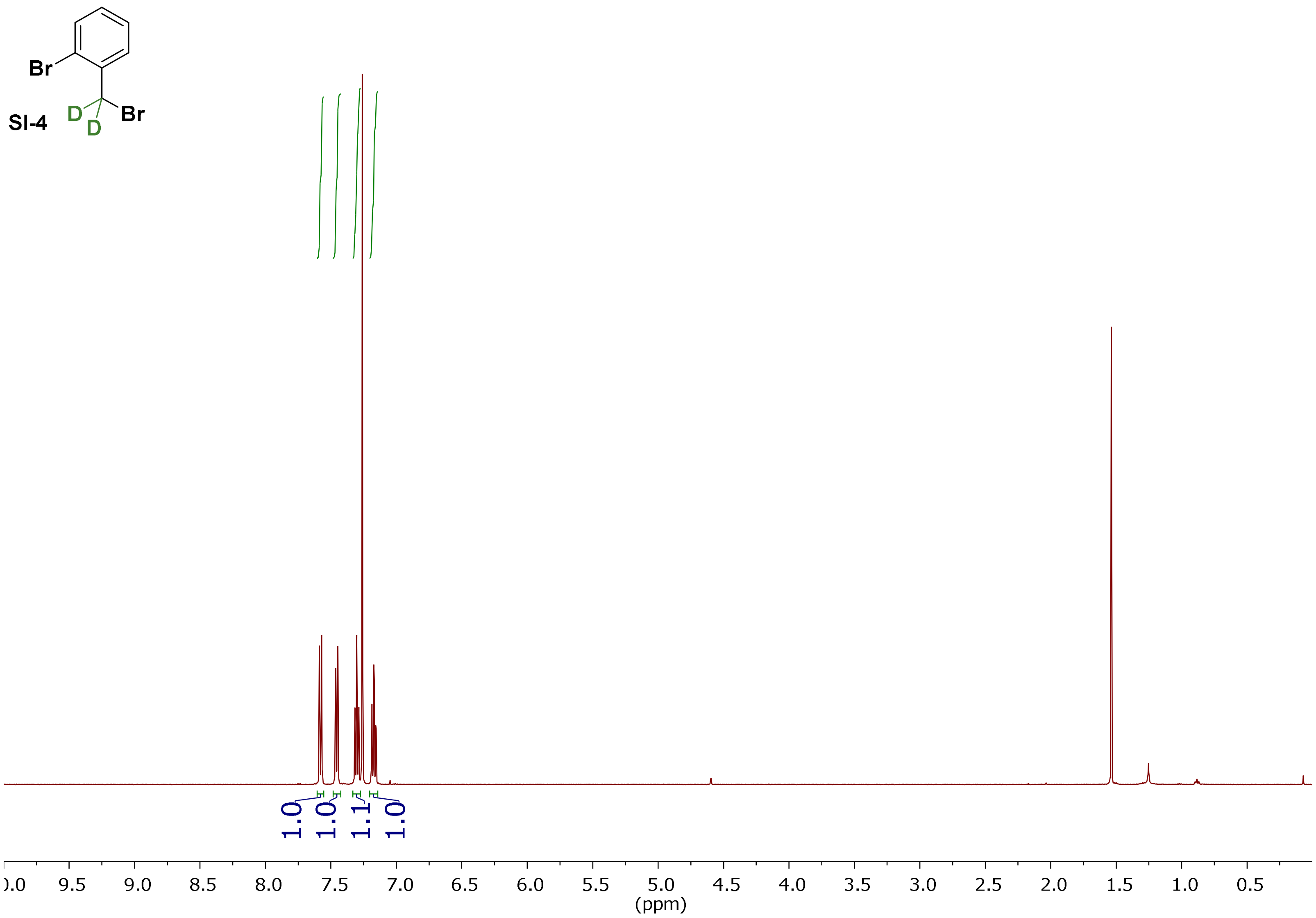

Supplementary Figure 29| ^{1}H-NMR spectrum (500 MHz, $CDCl_3$) for **SI-4**.

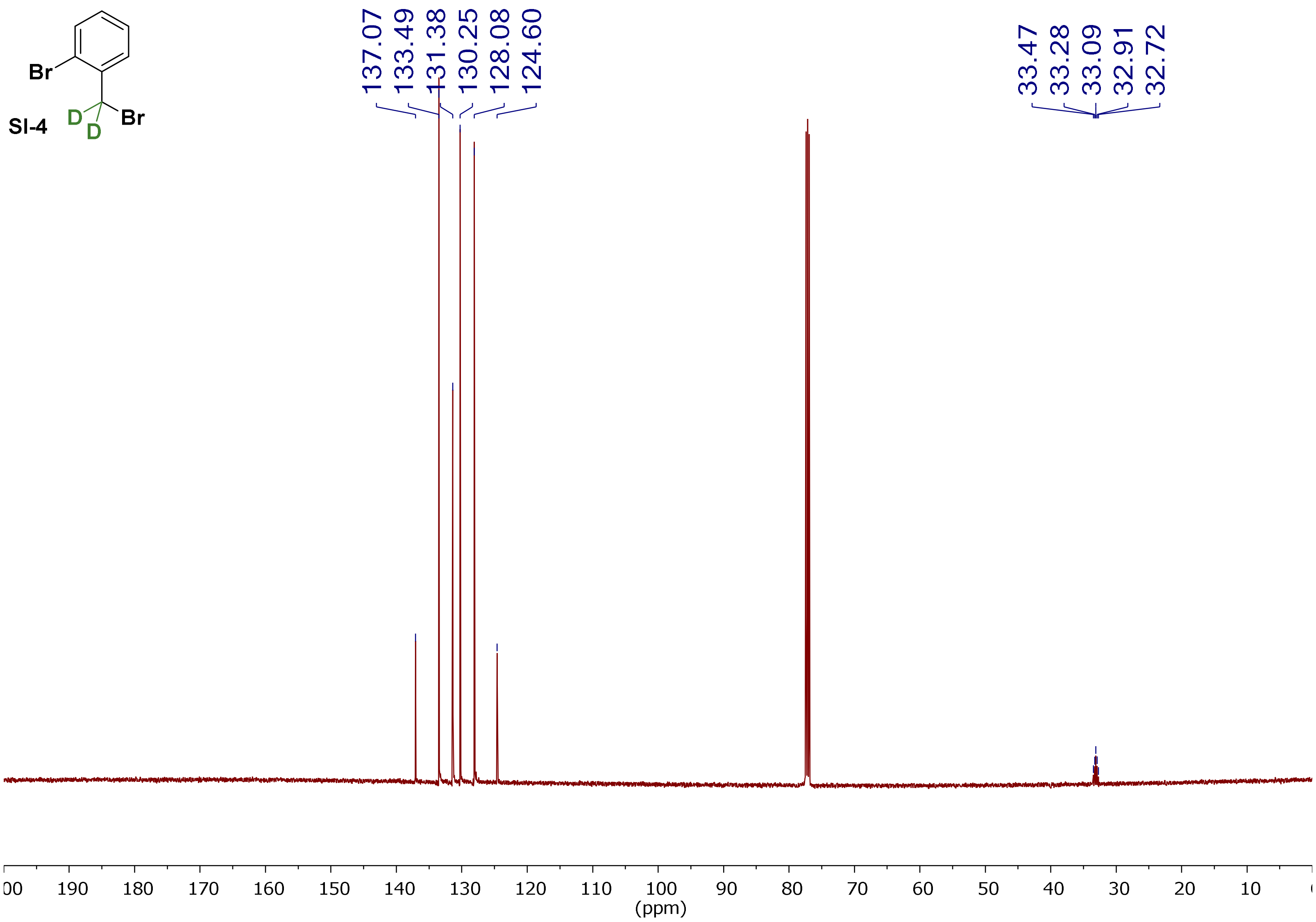

Supplementary Figure 30| ^{13}C-NMR spectrum (126 MHz, $CDCl_3$) for **SI-4**.

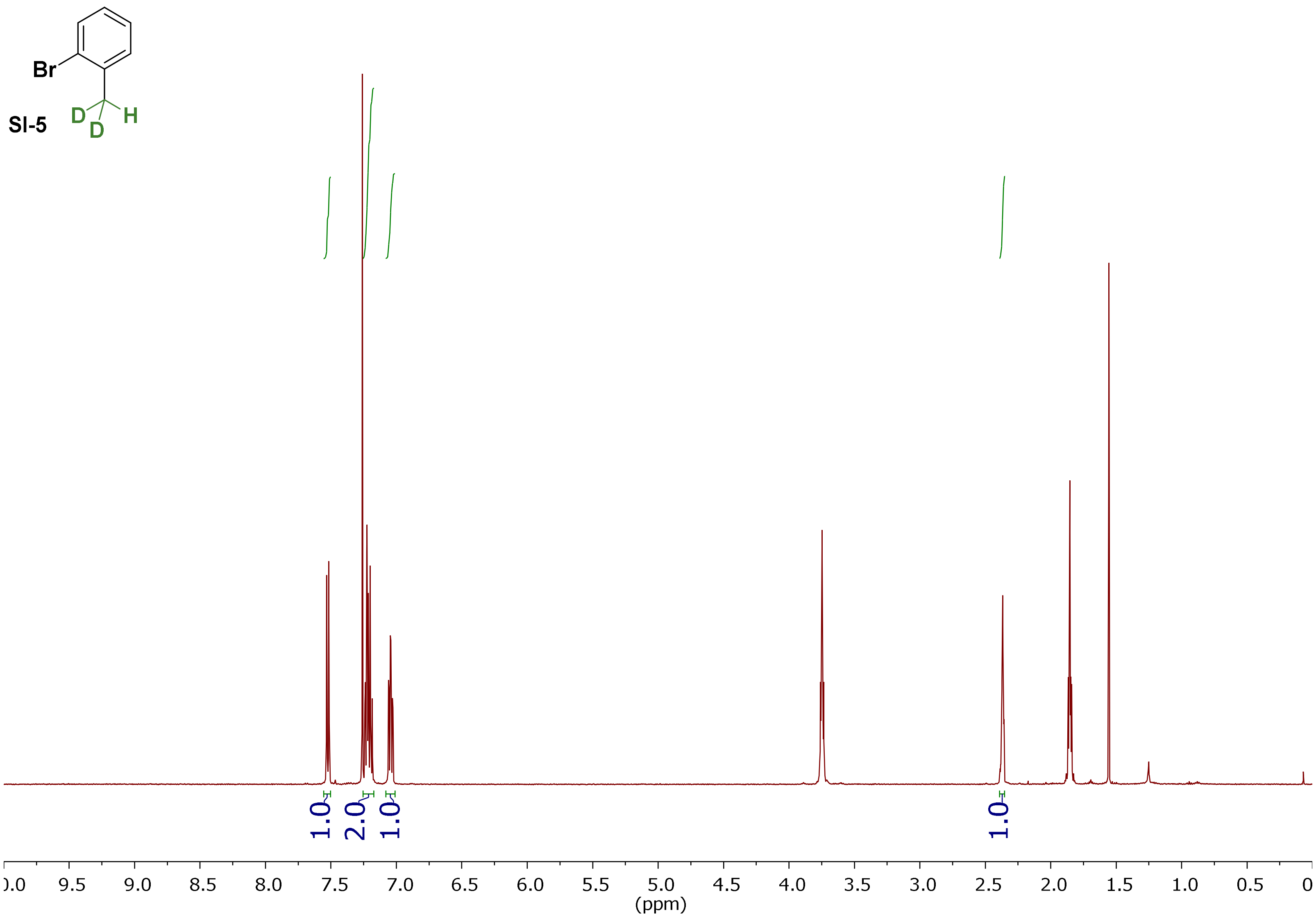

Supplementary Figure 31| ^{1}H-NMR spectrum (500 MHz, $CDCl_3$) for **SI-5**.

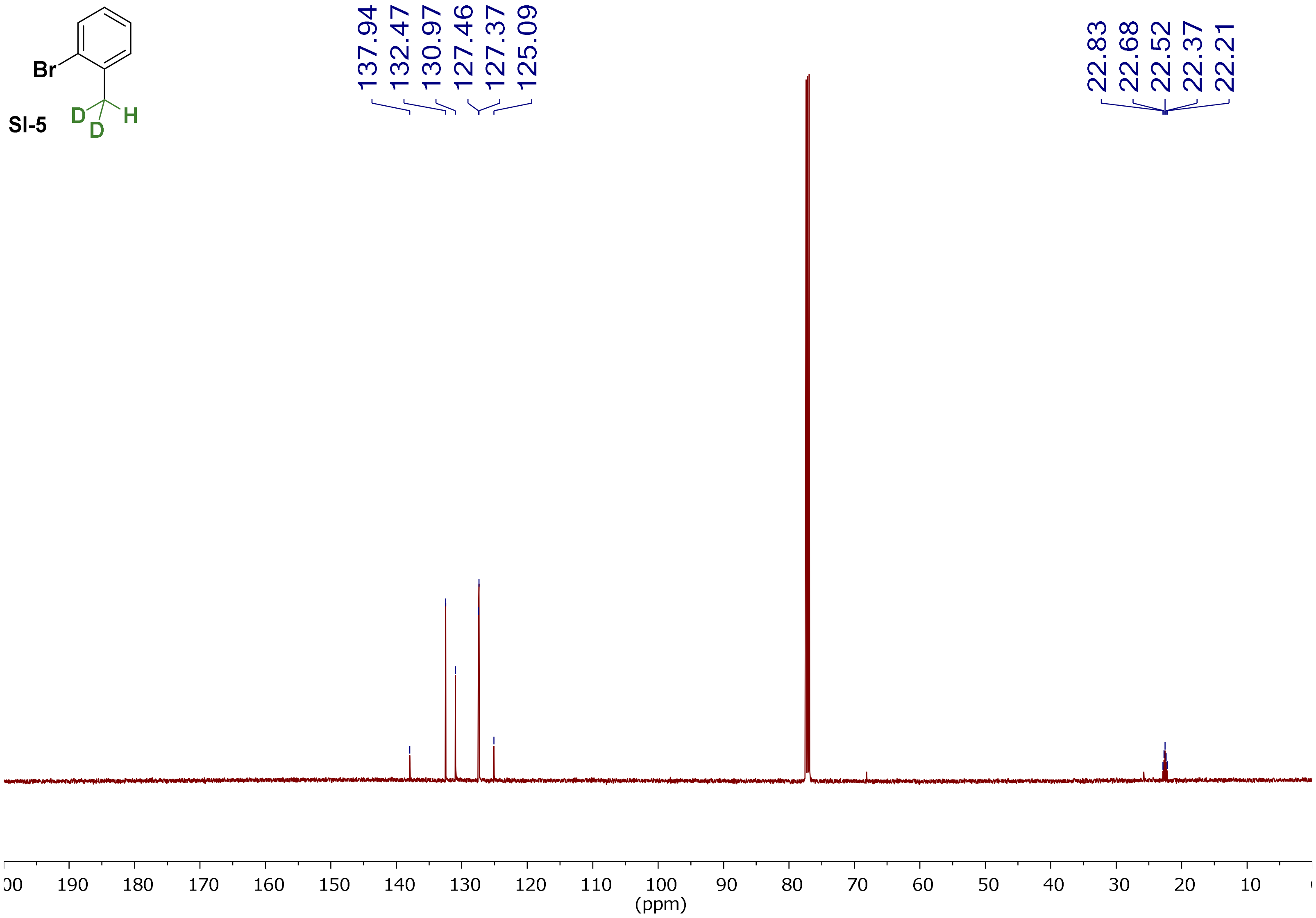

Supplementary Figure 32| ^{13}C-NMR spectrum (126 MHz, $CDCl_3$) for **SI-5**.

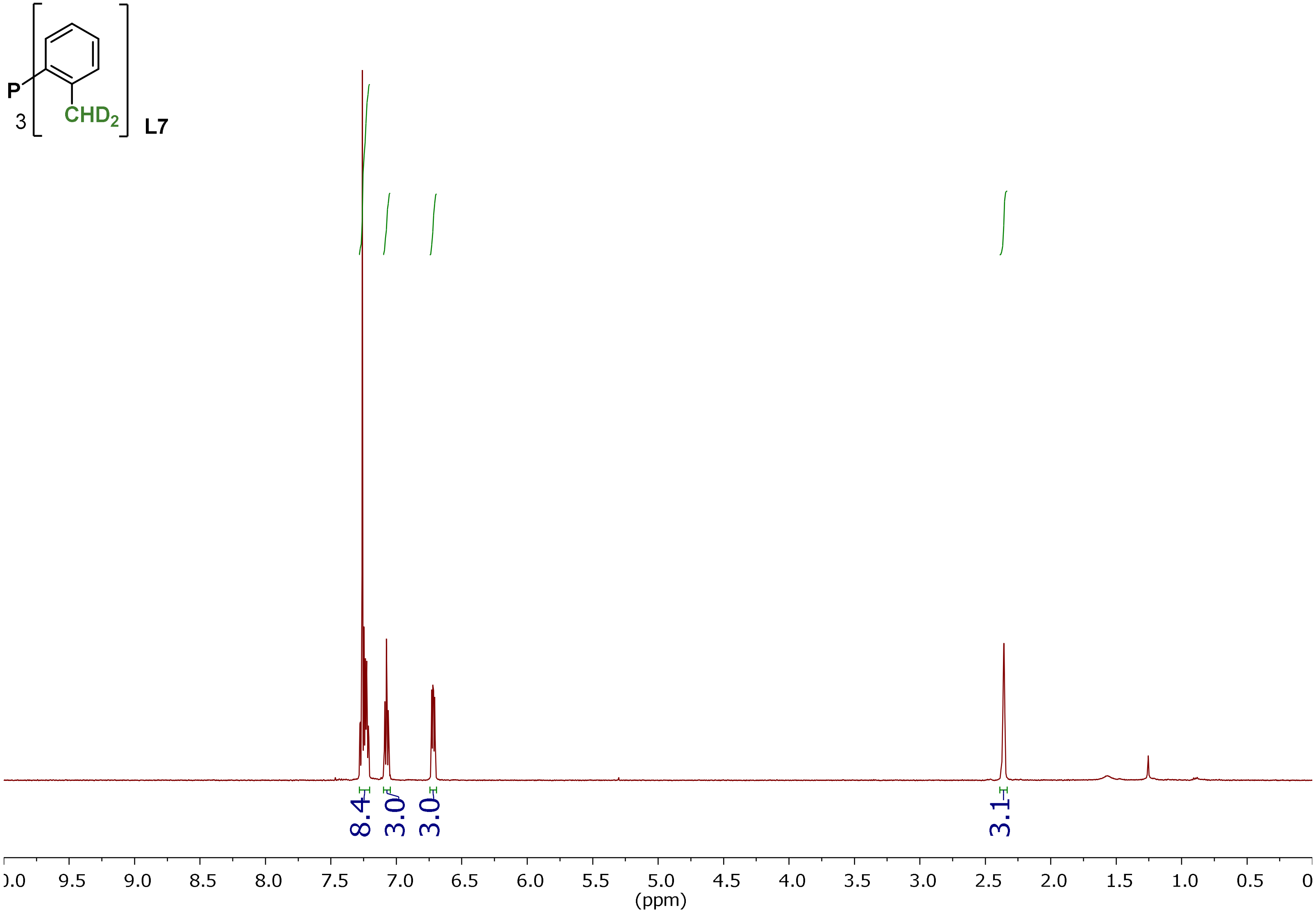

Supplementary Figure 33| ^{1}H-NMR spectrum (500 MHz, $CDCl_3$) for **L7**.

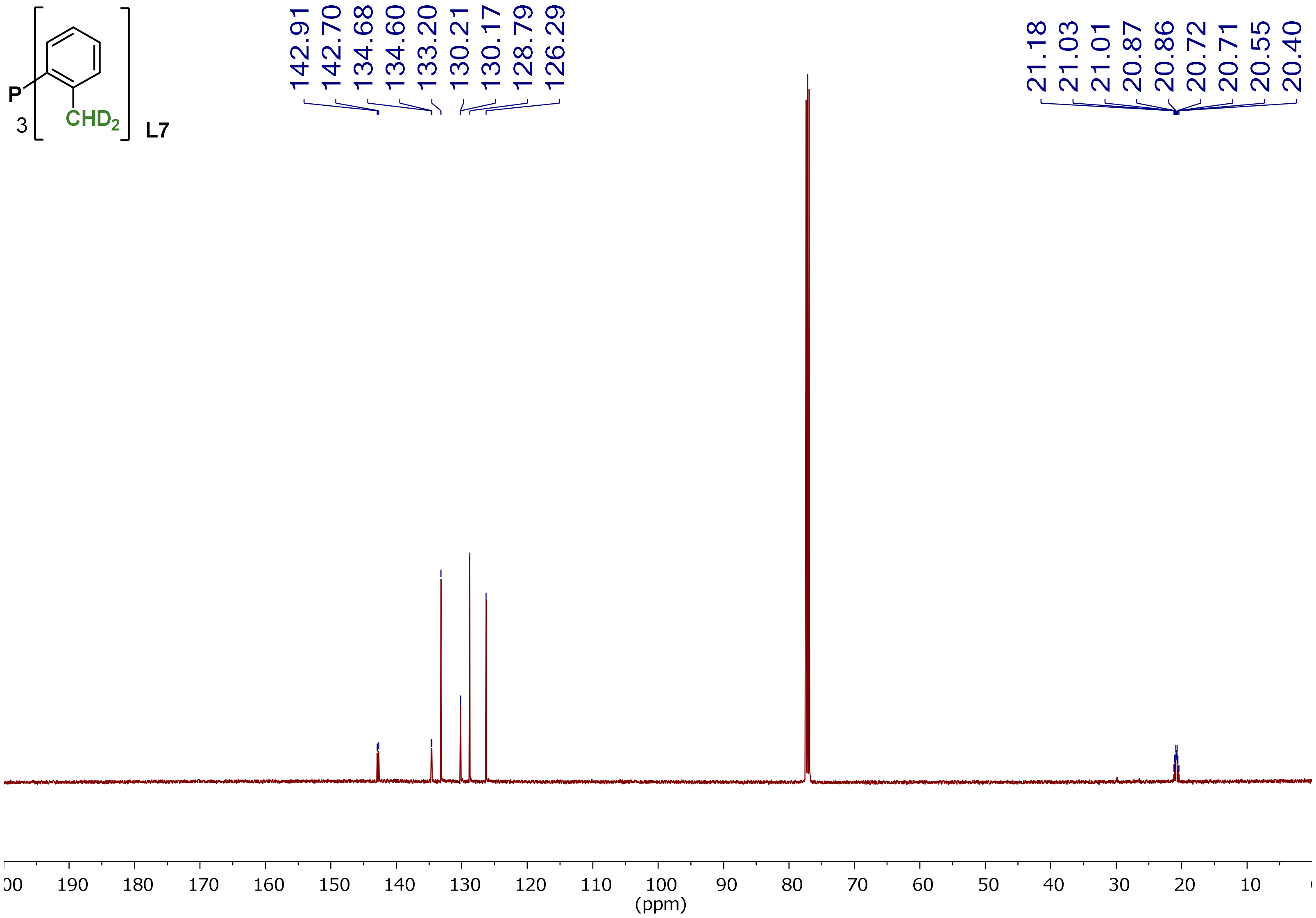

Supplementary Figure 34| ^{13}C-NMR spectrum (126 MHz, $CDCl_3$) for **L7**.

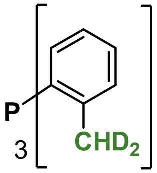

L7

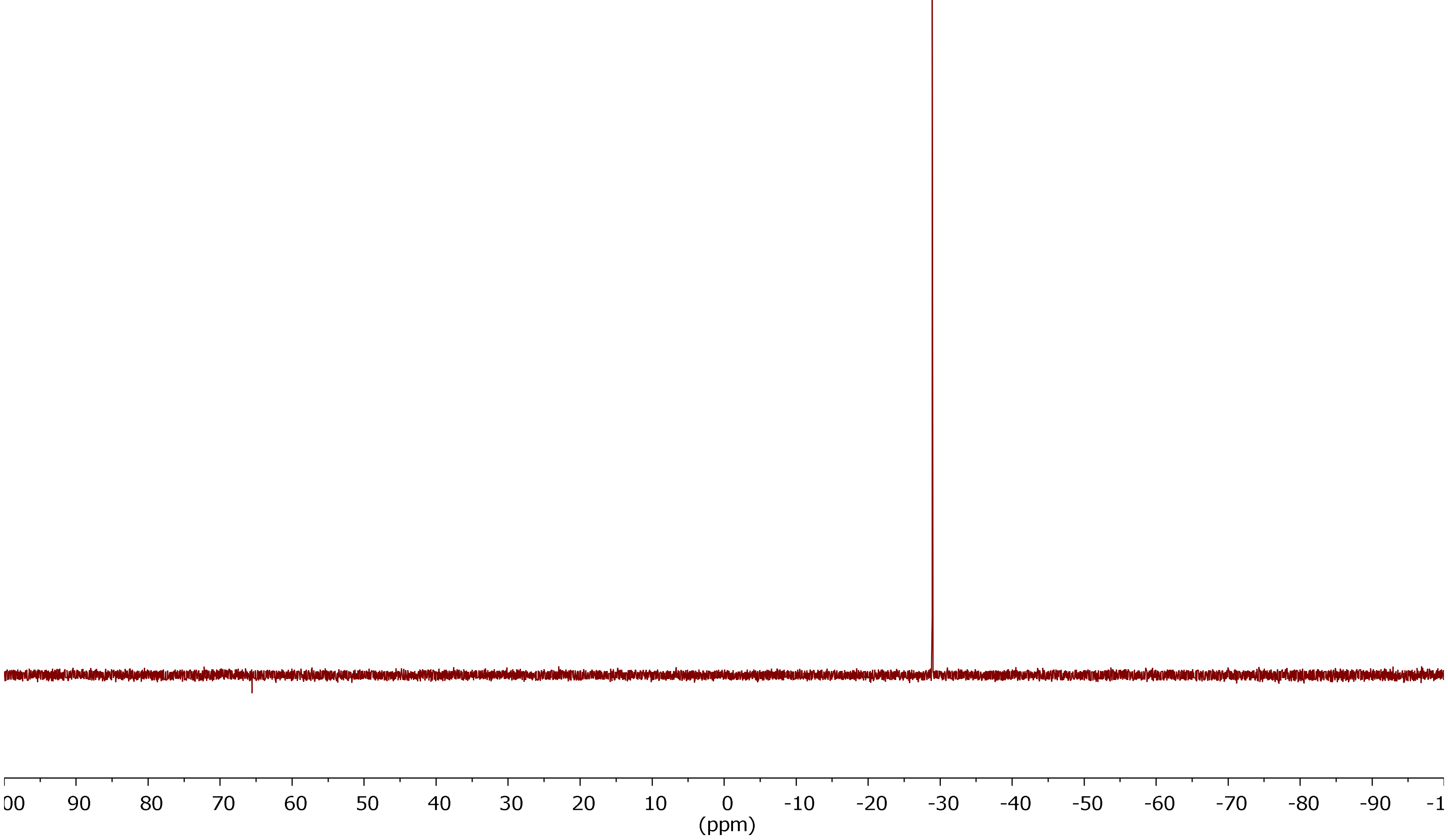

Supplementary Figure 35| ^{31}P-NMR spectrum (202 MHz, $CDCl_3$) for **L7**.

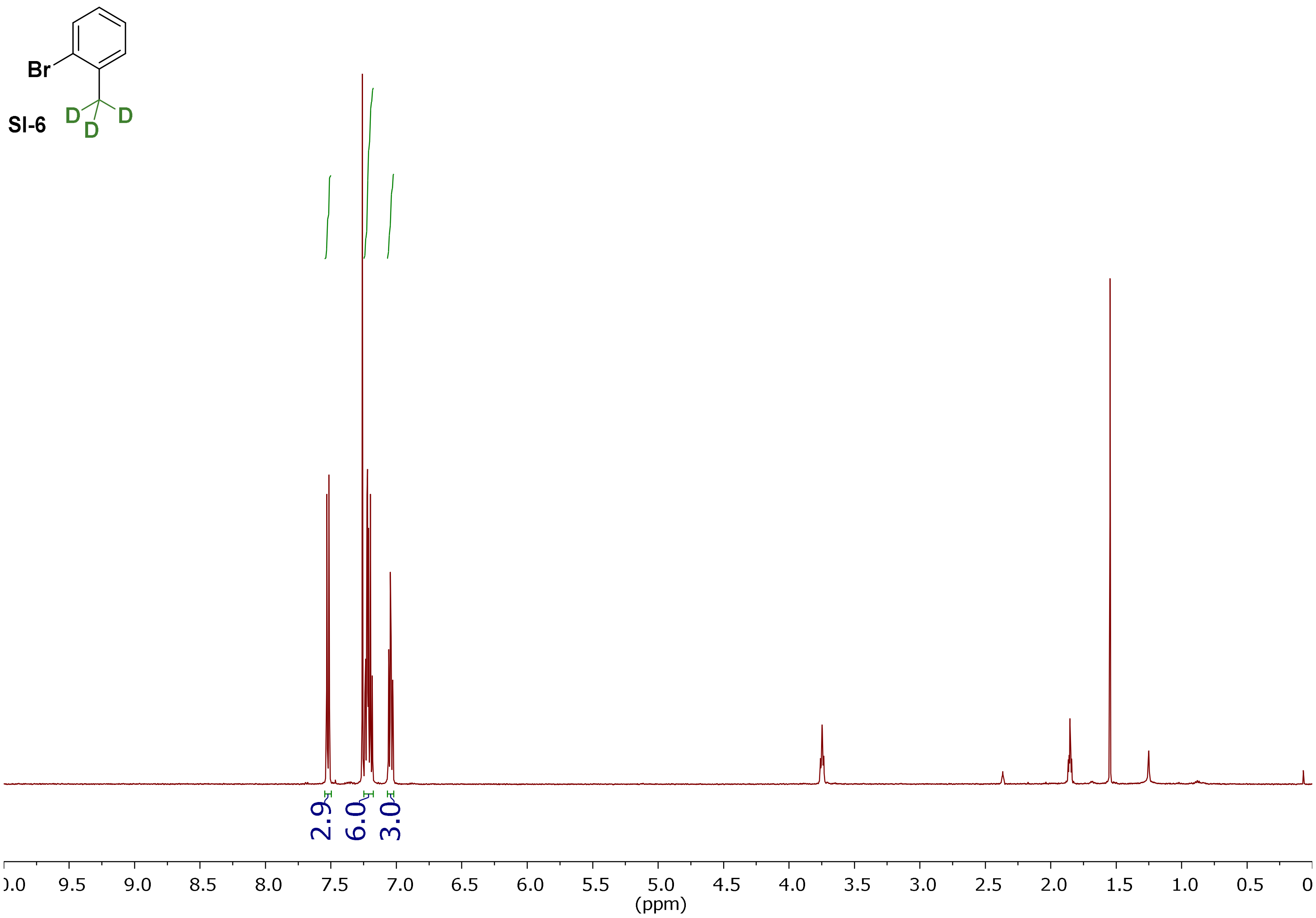

Supplementary Figure 36| ^{1}H-NMR spectrum (500 MHz, $CDCl_3$) for **SI-6**.

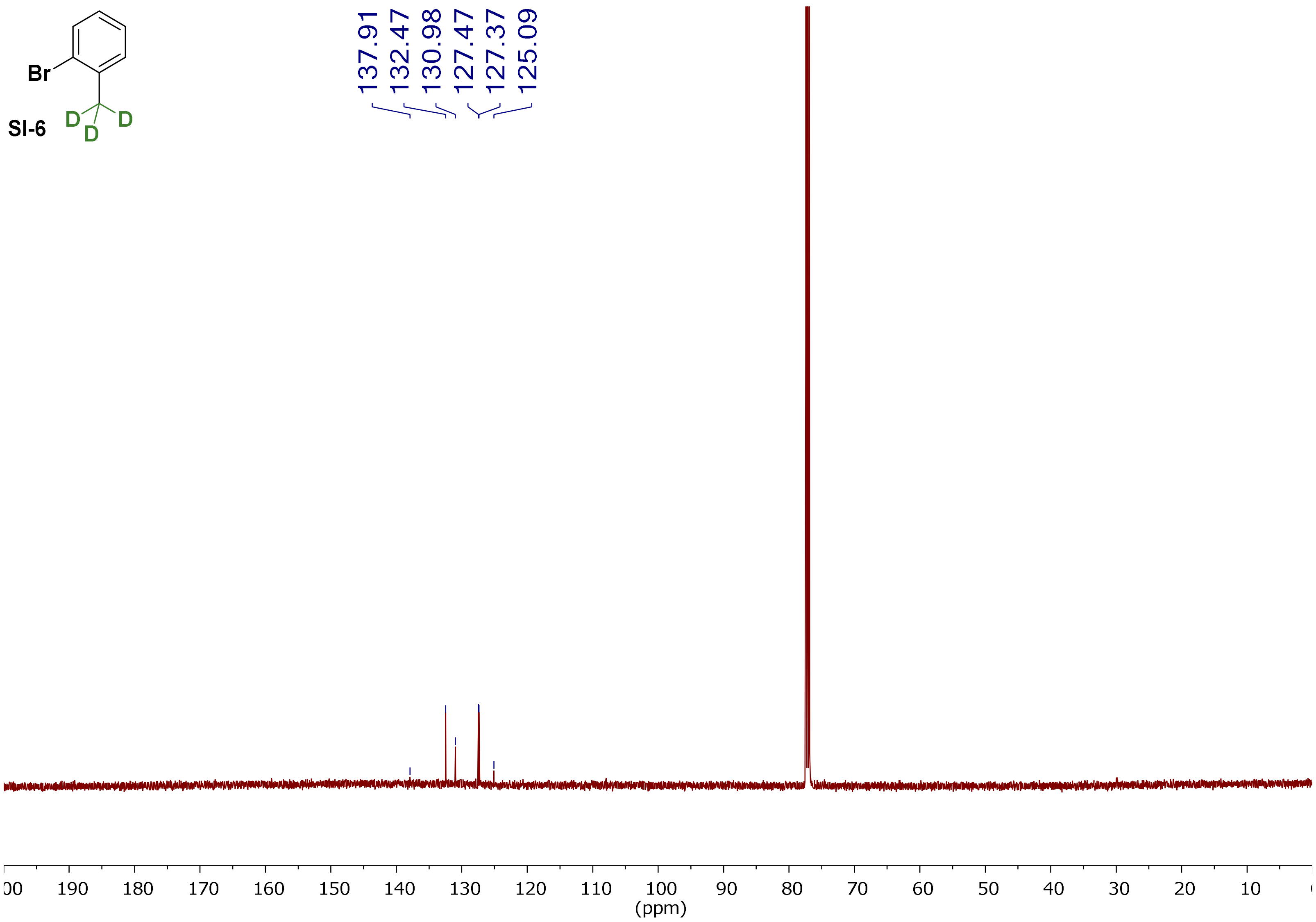

Supplementary Figure 37| ^{13}C-NMR spectrum (126 MHz, $CDCl_3$) for **SI-6**.

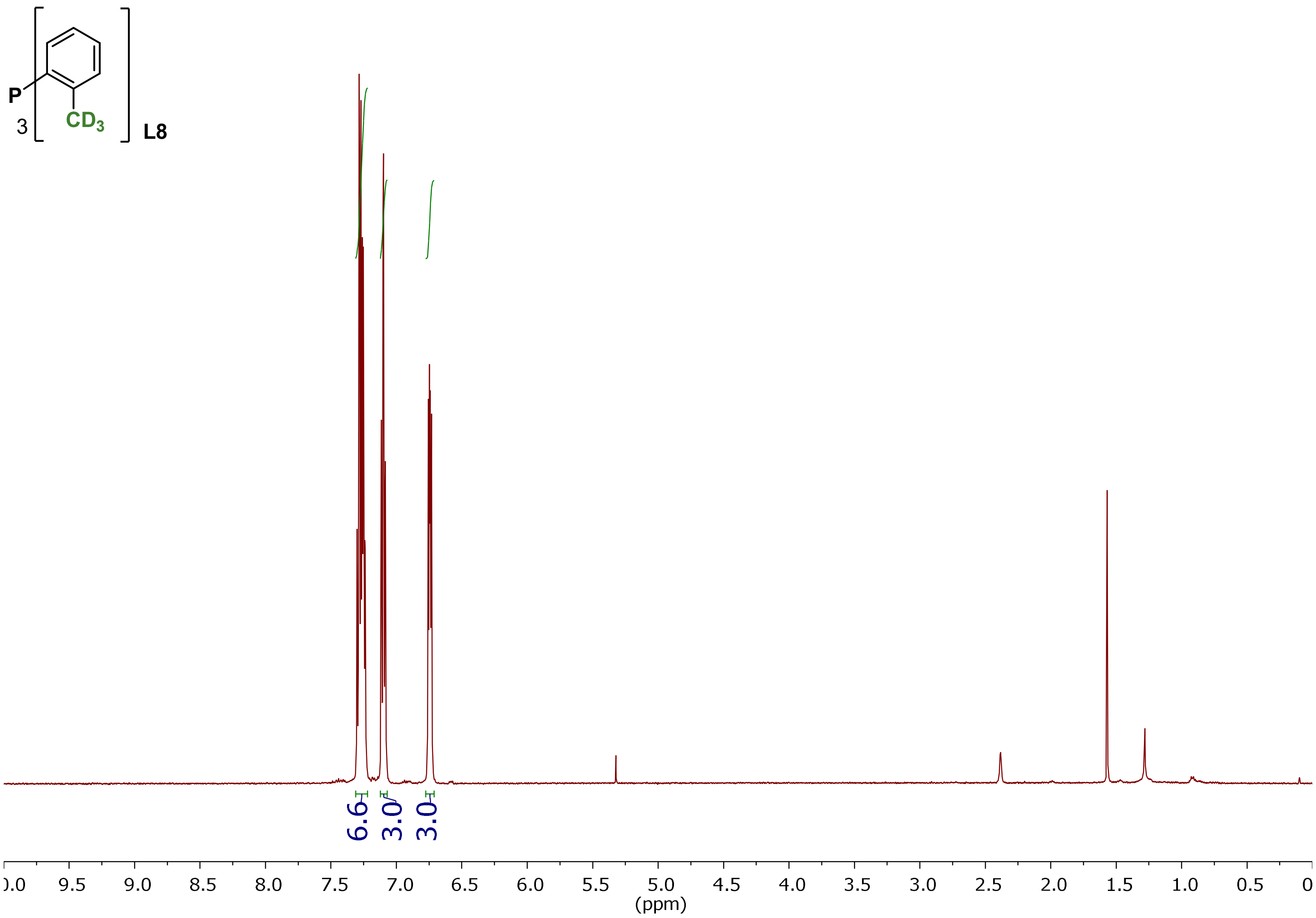

Supplementary Figure 38| ^{1}H-NMR spectrum (500 MHz, $CDCl_3$) for **L8**.

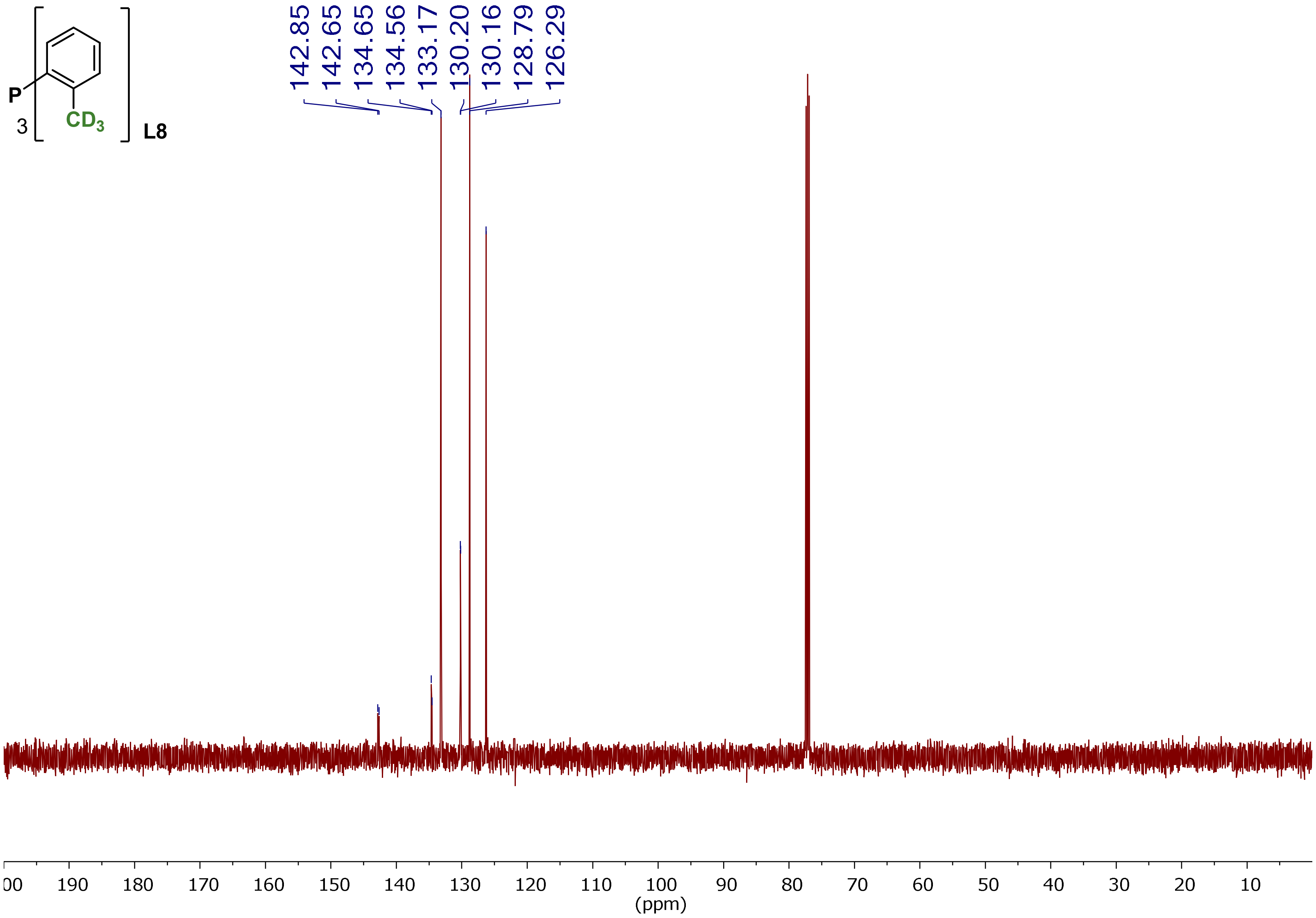

Supplementary Figure 39| ^{13}C-NMR spectrum (126 MHz, $CDCl_3$) for **L8**.

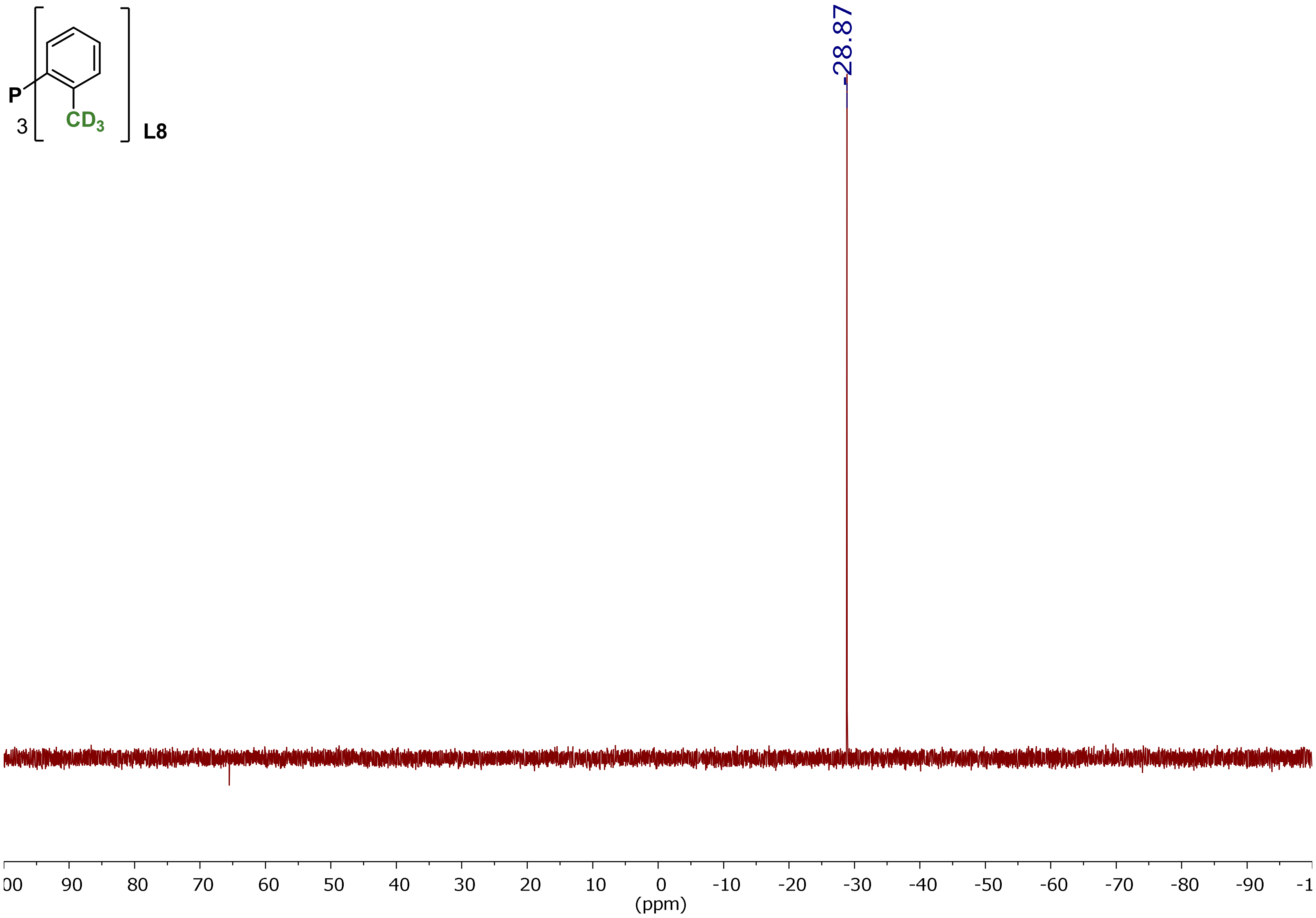

Supplementary Figure 40| ^{31}P-NMR spectrum (202 MHz, $CDCl_3$) for **L8**.

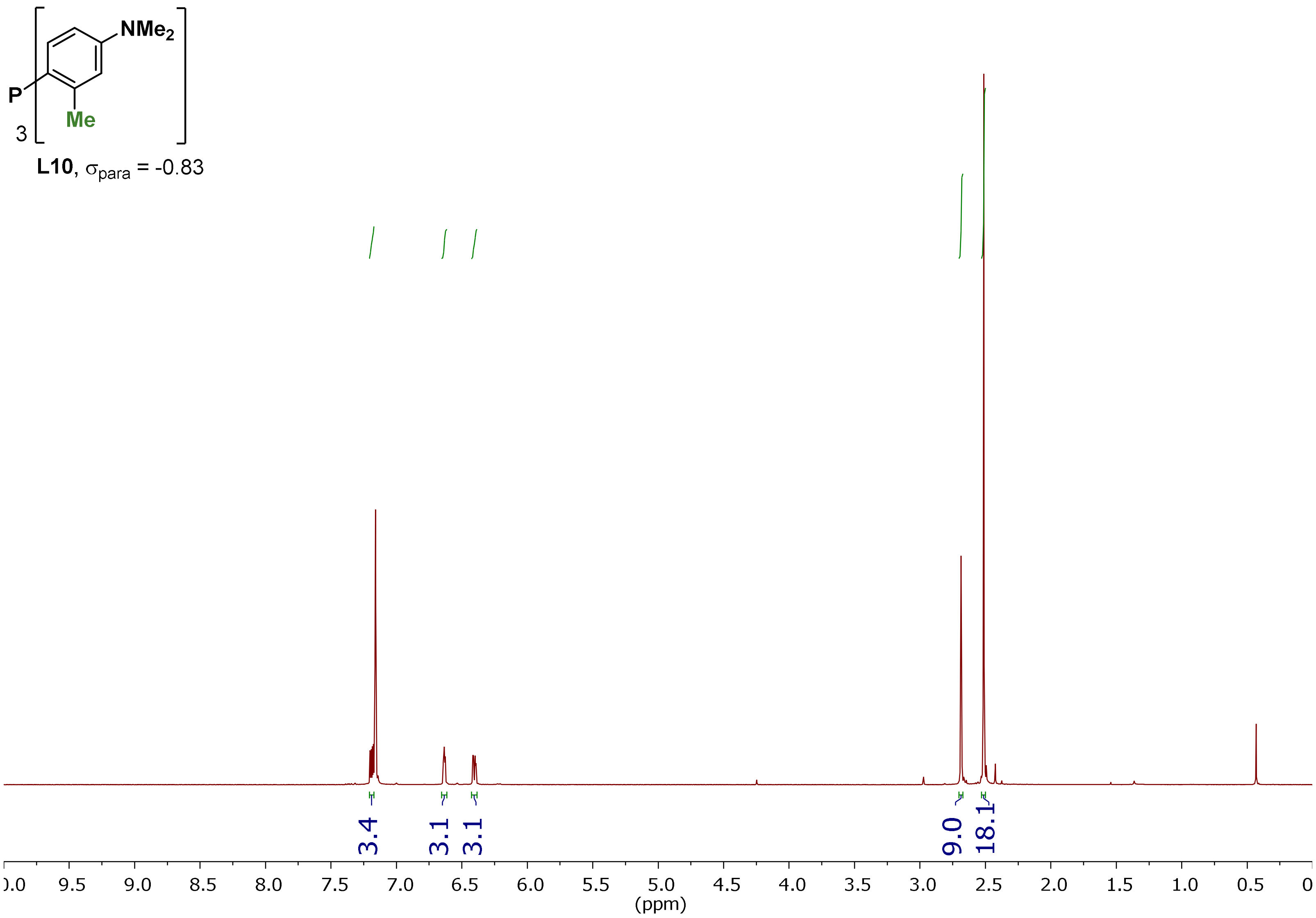

Supplementary Figure 41| ^{1}H-NMR spectrum (500 MHz, C_6D_6) for **L10**.

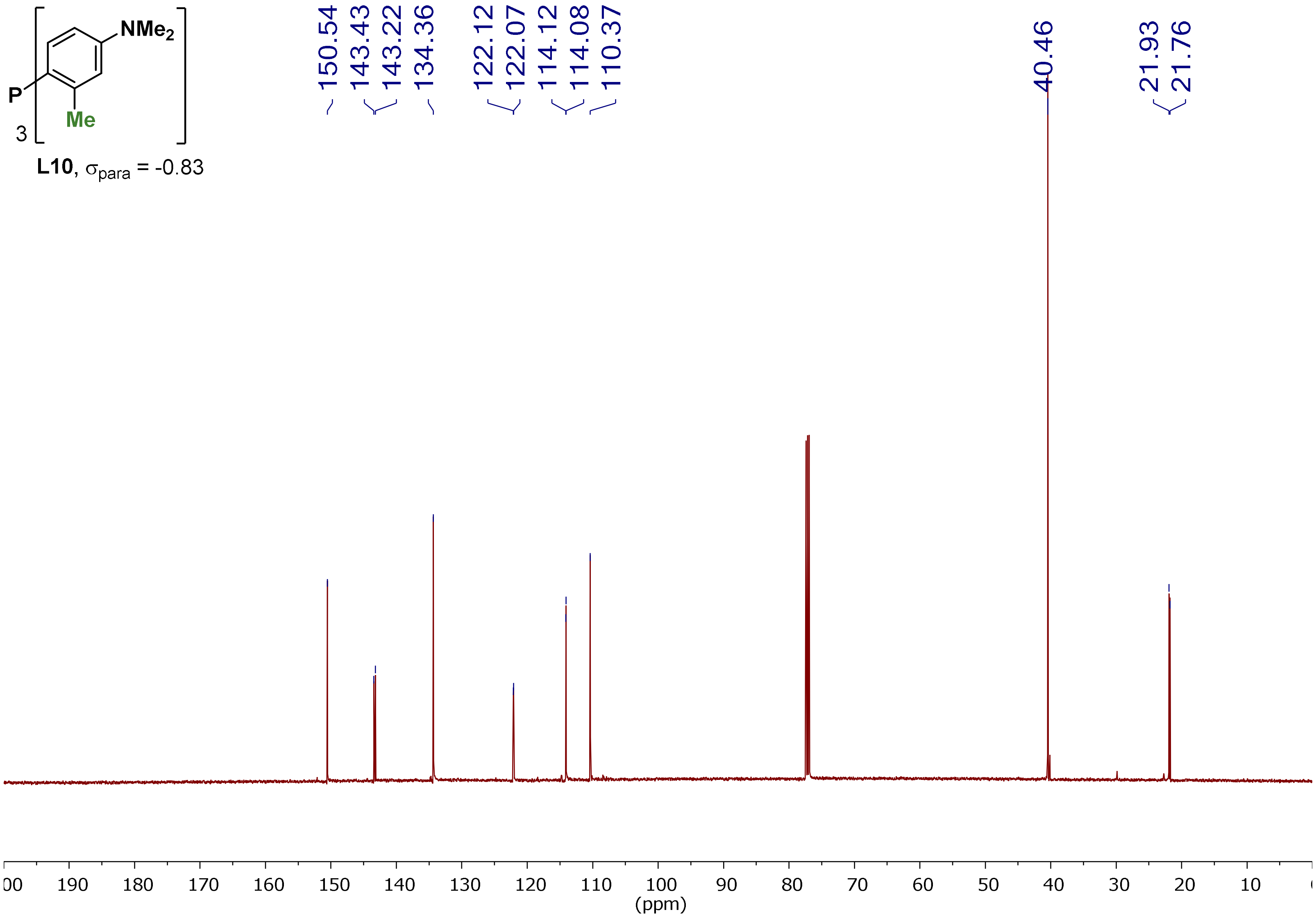

Supplementary Figure 42| ^{13}C-NMR spectrum (126 MHz, $CDCl_3$) for **L10**.

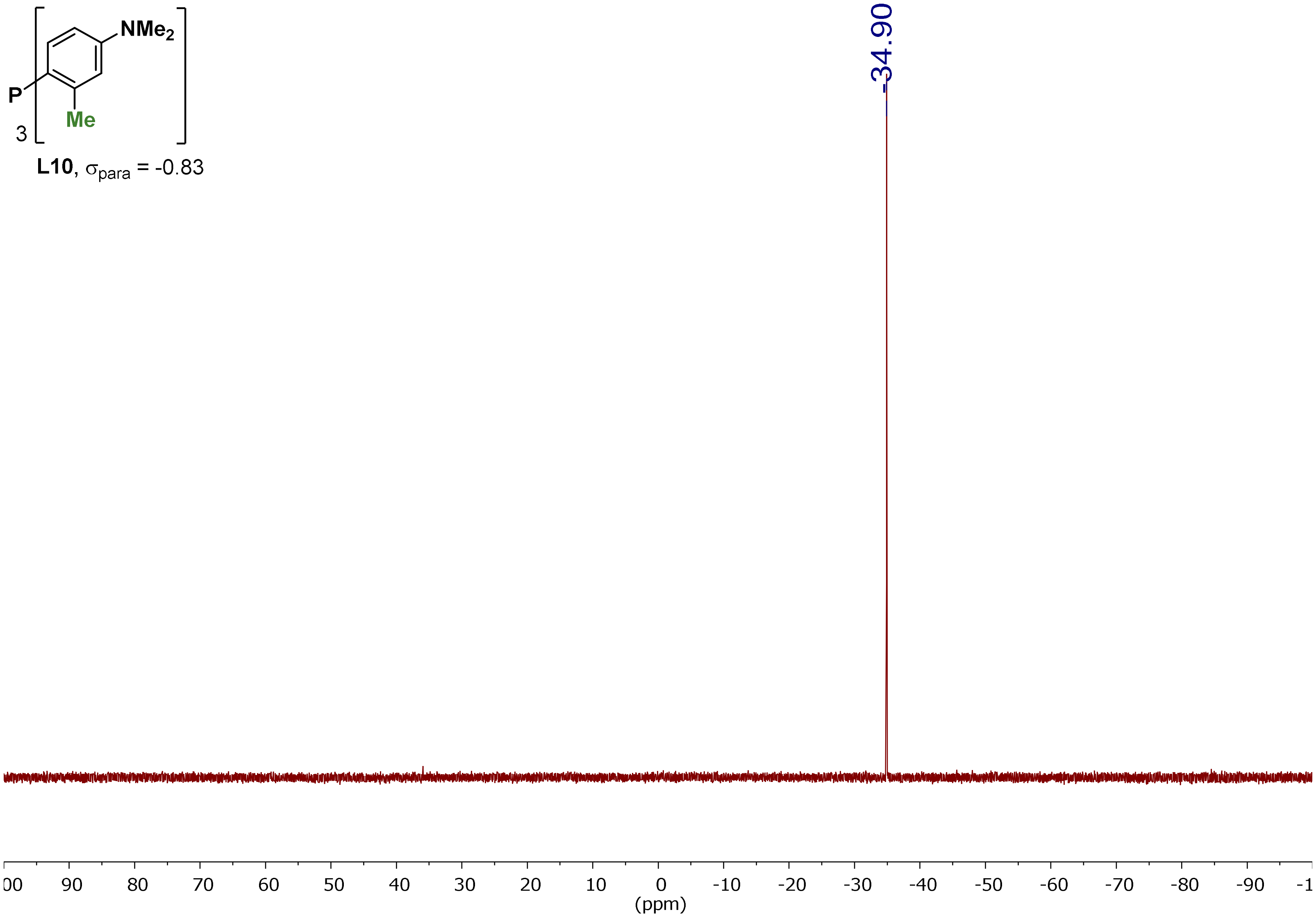

Supplementary Figure 43| ^{31}P-NMR spectrum (202 MHz, C_6D_6) for **L10**.

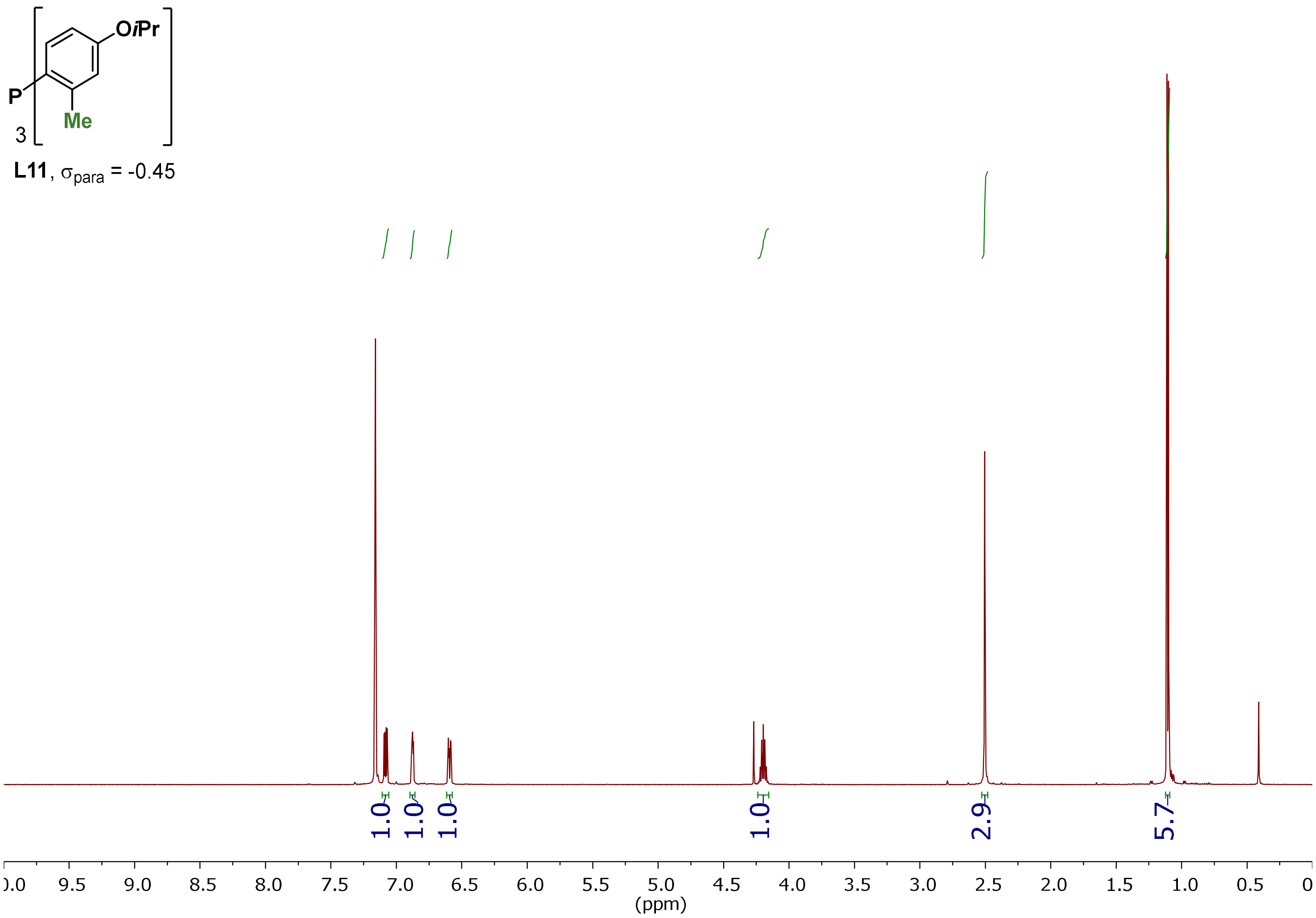

Supplementary Figure 44| ^{1}H-NMR spectrum (500 MHz, C_6D_6) for **L11**.

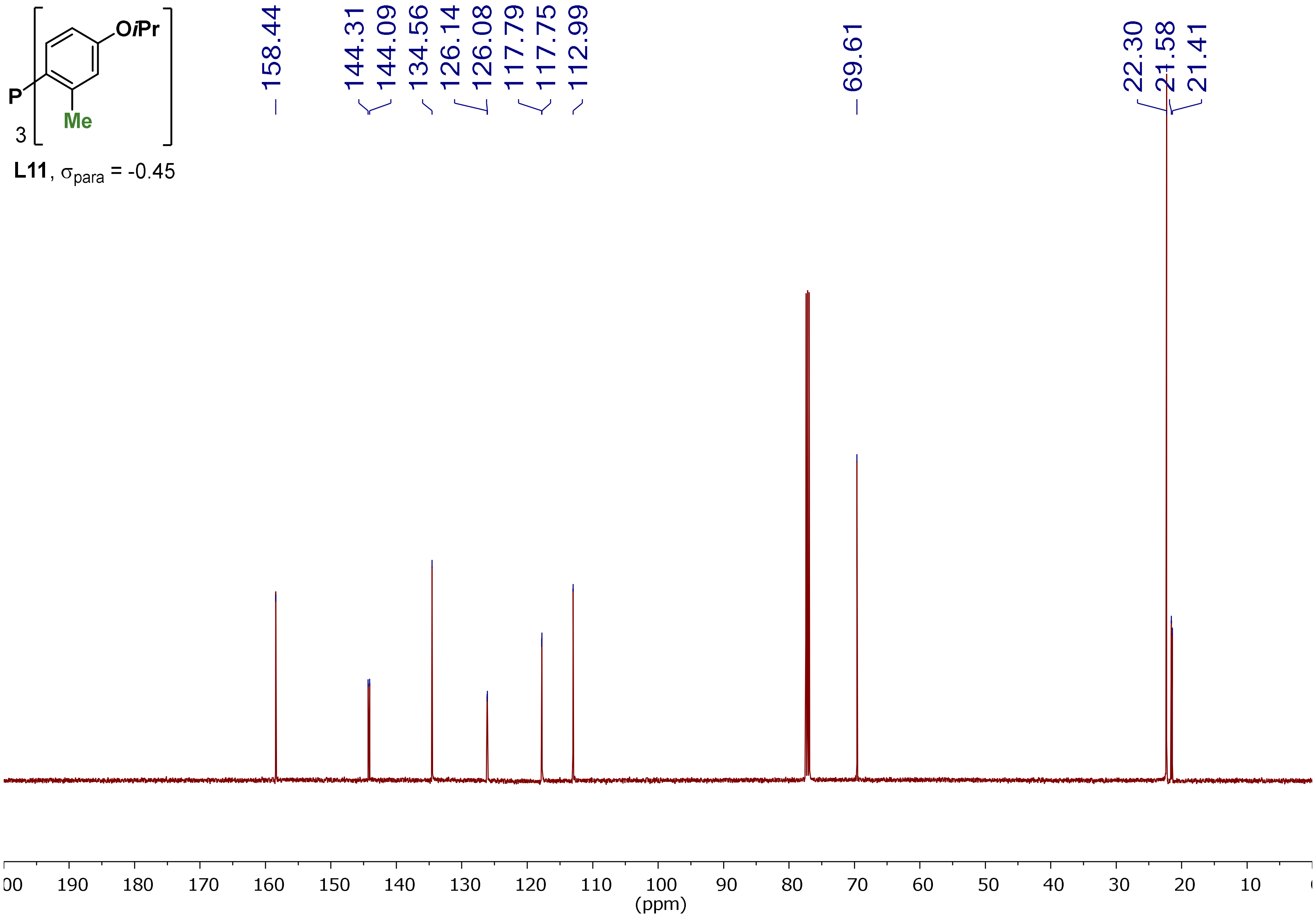

Supplementary Figure 45| ^{13}C-NMR spectrum (126 MHz, $CDCl_3$) for **L11**.

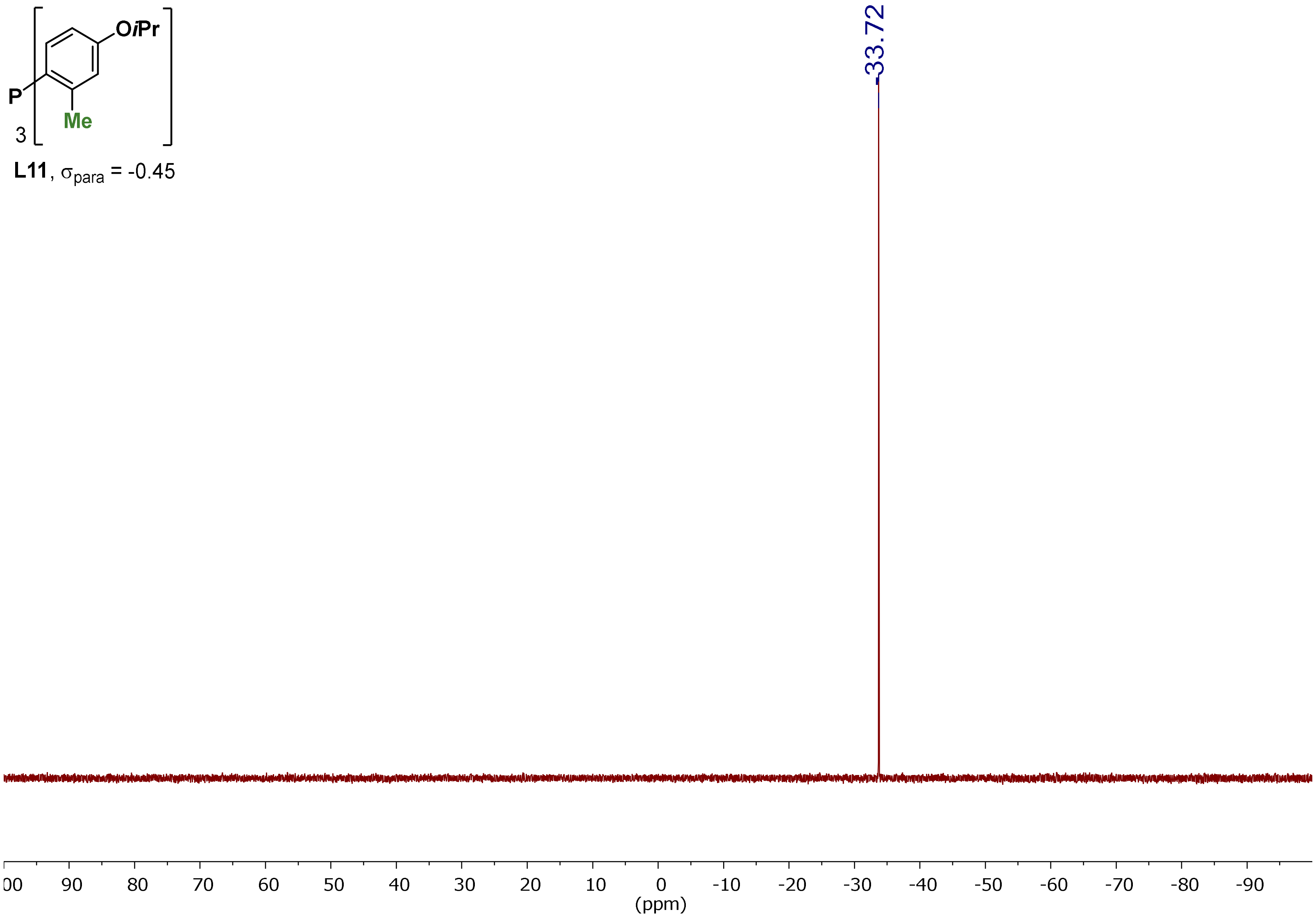

Supplementary Figure 46| ^{31}P-NMR spectrum (202 MHz, C_6D_6) for **L11**.

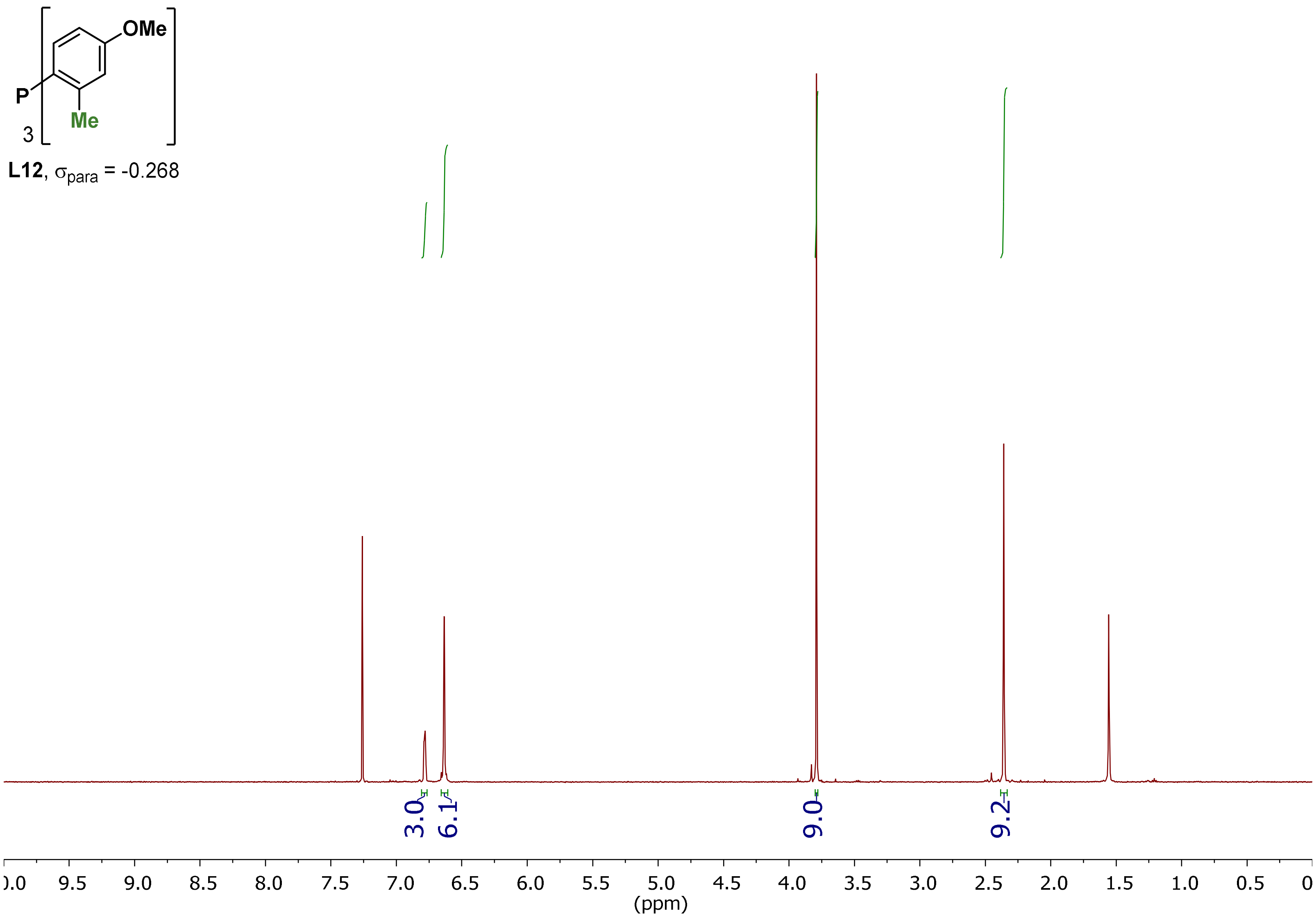

Supplementary Figure 47| ^{1}H-NMR spectrum (500 MHz, $CDCl_3$) for **L12**.

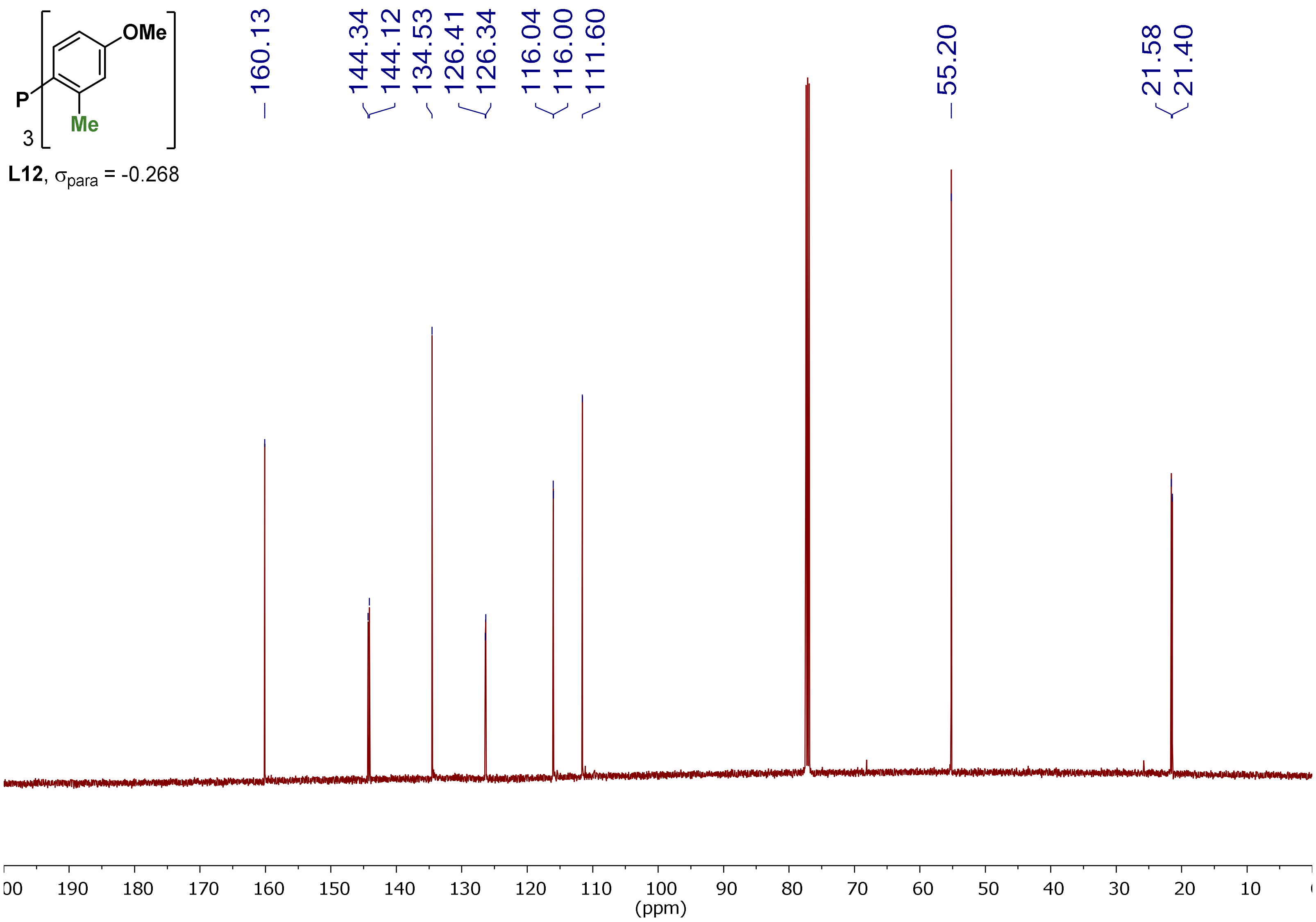

Supplementary Figure 48| ^{13}C-NMR spectrum (126 MHz, $CDCl_3$) for **L12**.

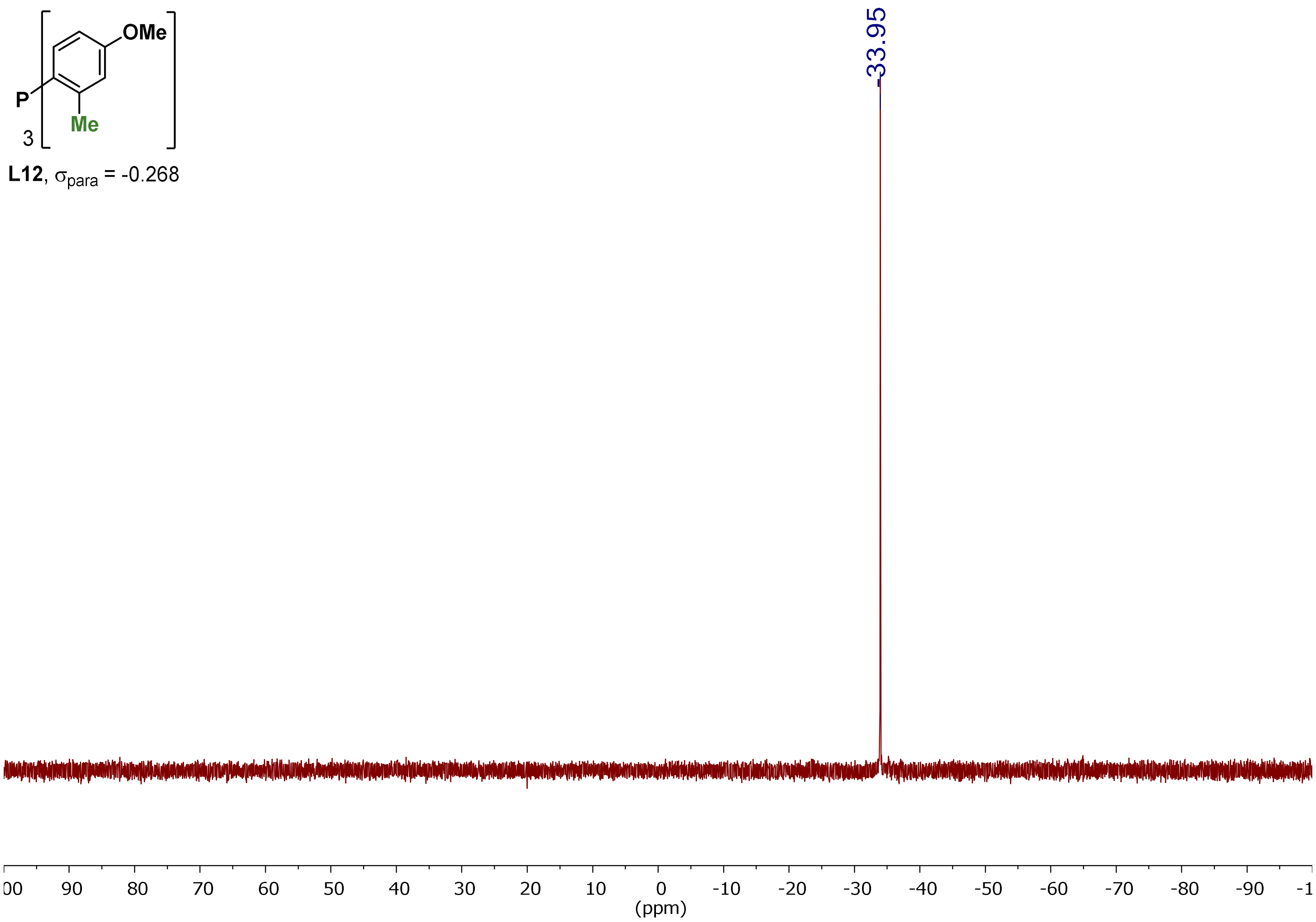

Supplementary Figure 49| ^{31}P-NMR spectrum (202 MHz, C_6D_6) for **L12**.

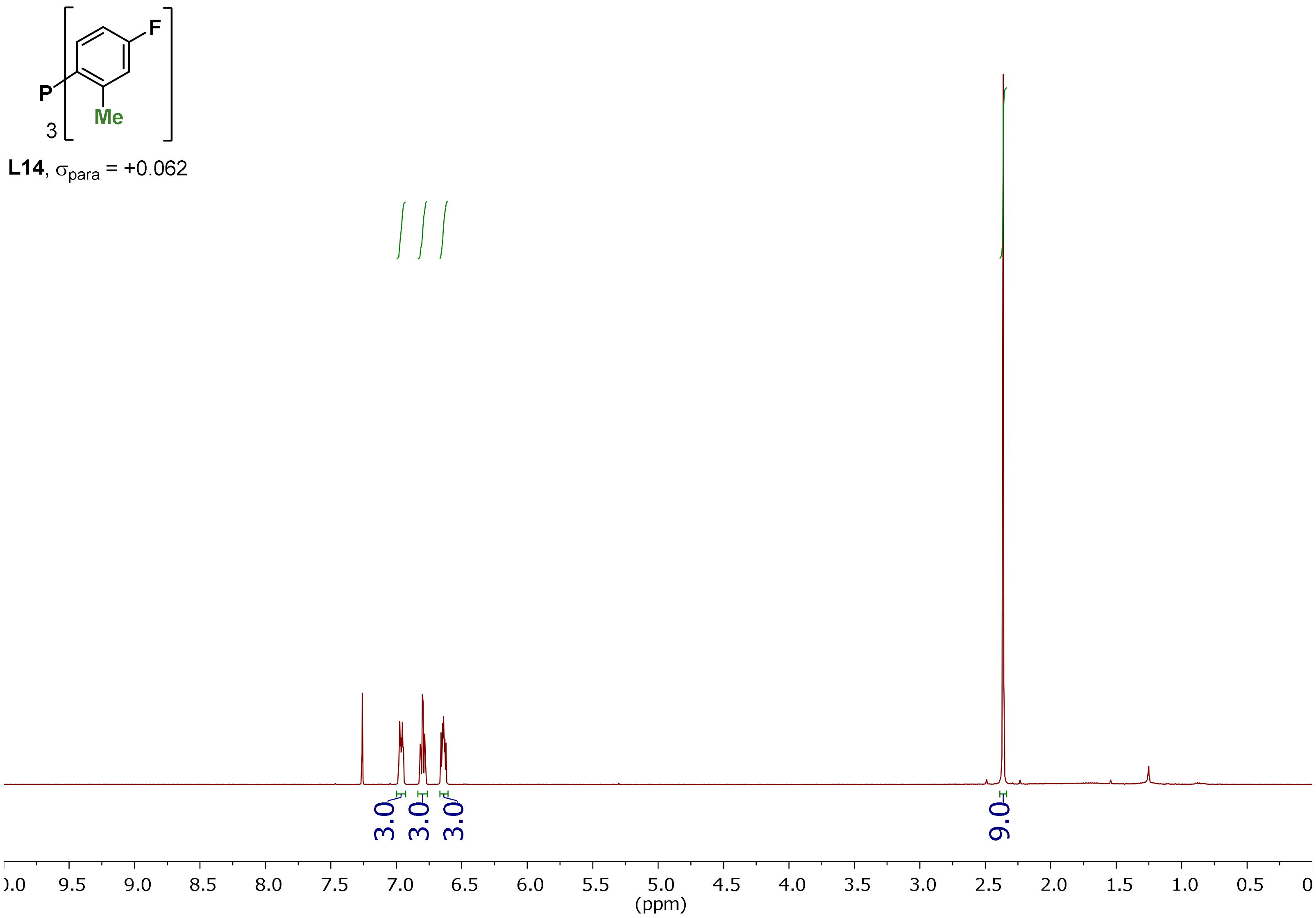

Supplementary Figure 50| ^{1}H-NMR spectrum (500 MHz, $CDCl_3$) for **L14**.

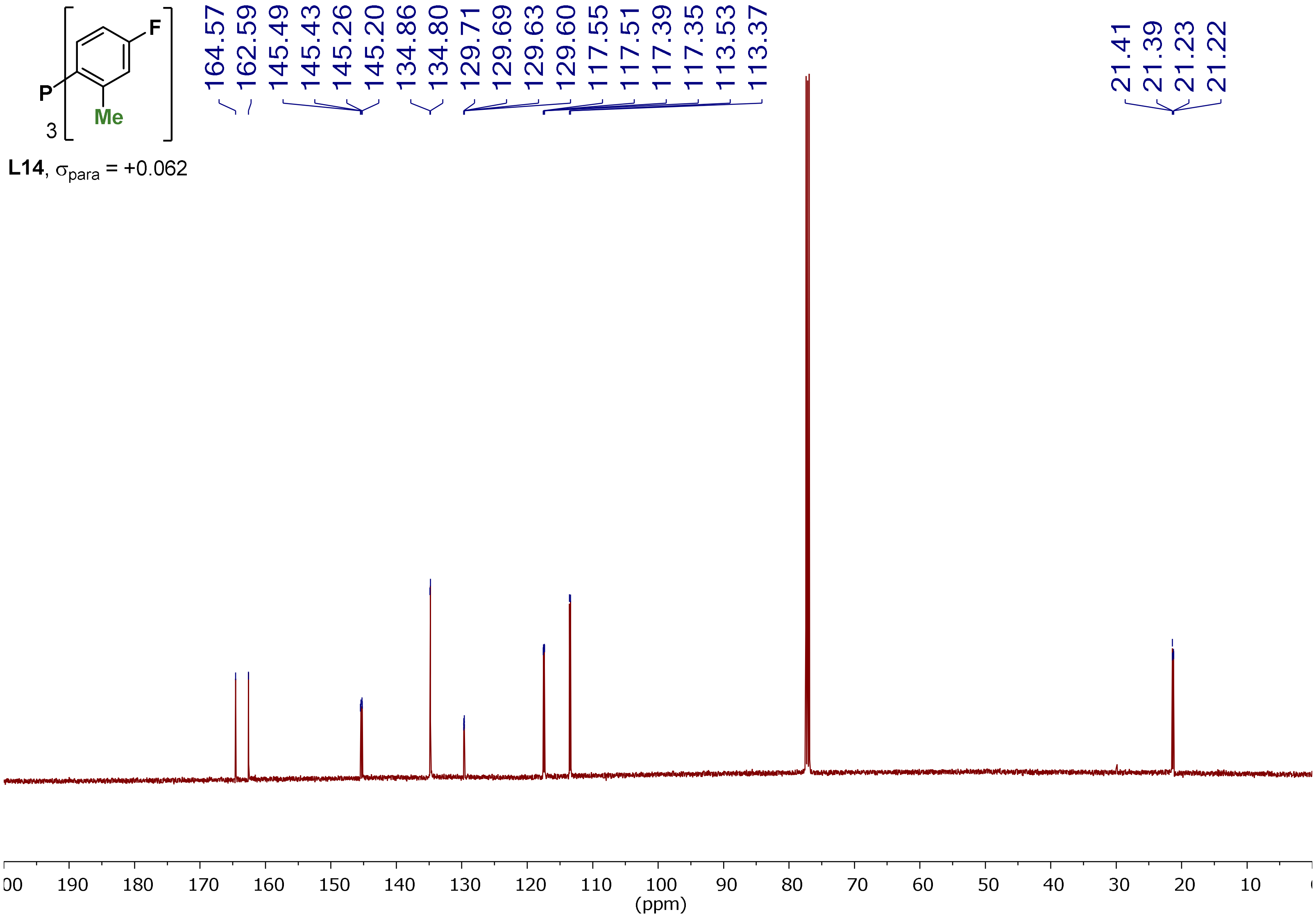

Supplementary Figure 51| ^{13}C-NMR spectrum (126 MHz, $CDCl_3$) for **L14**.

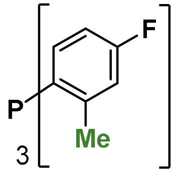

L14, σ_{para} = +0.062

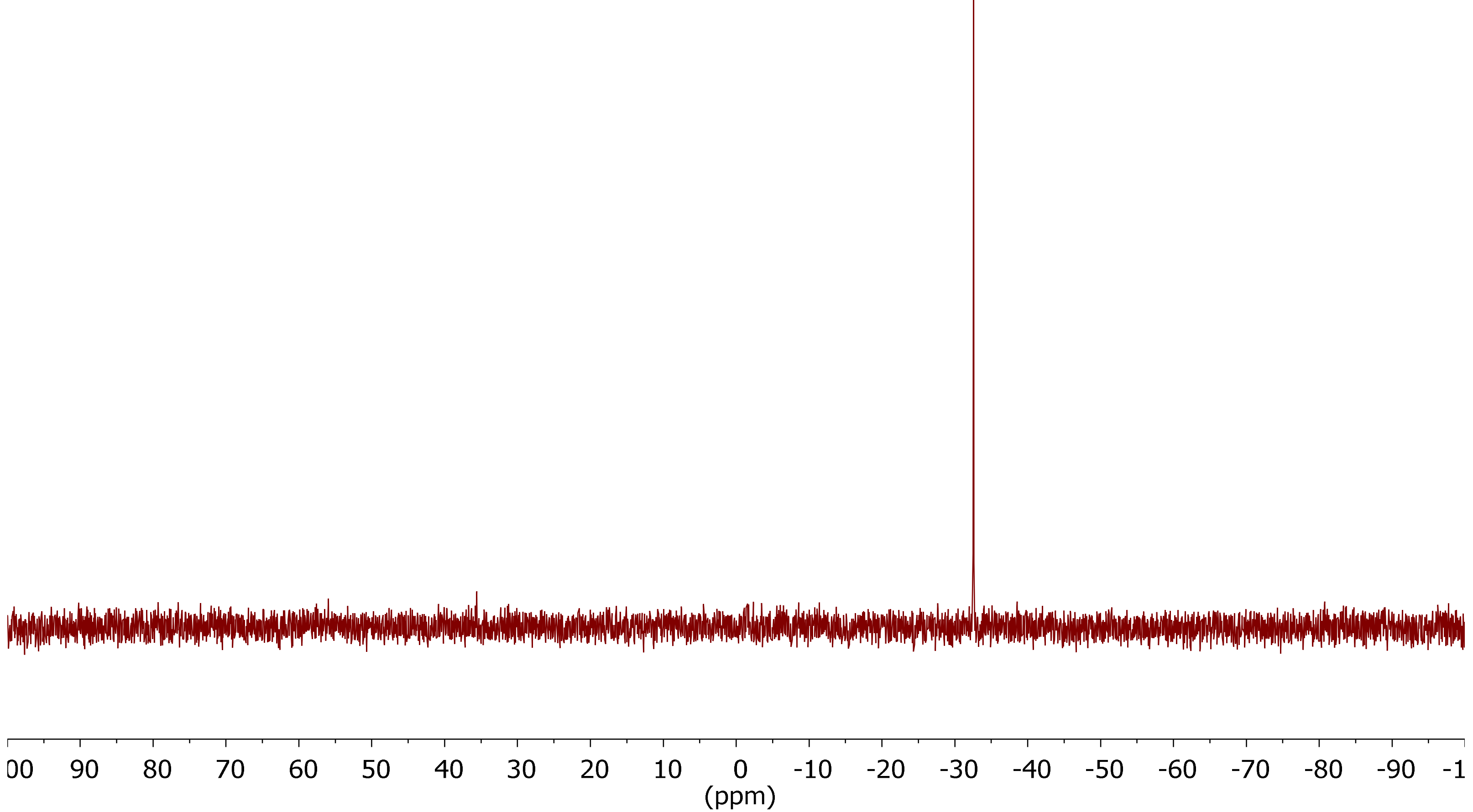

Supplementary Figure 52| ^{31}P-NMR spectrum (202 MHz, C_6D_6) for **L14**.

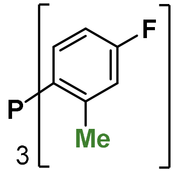

L14, σ_{para} = +0.062

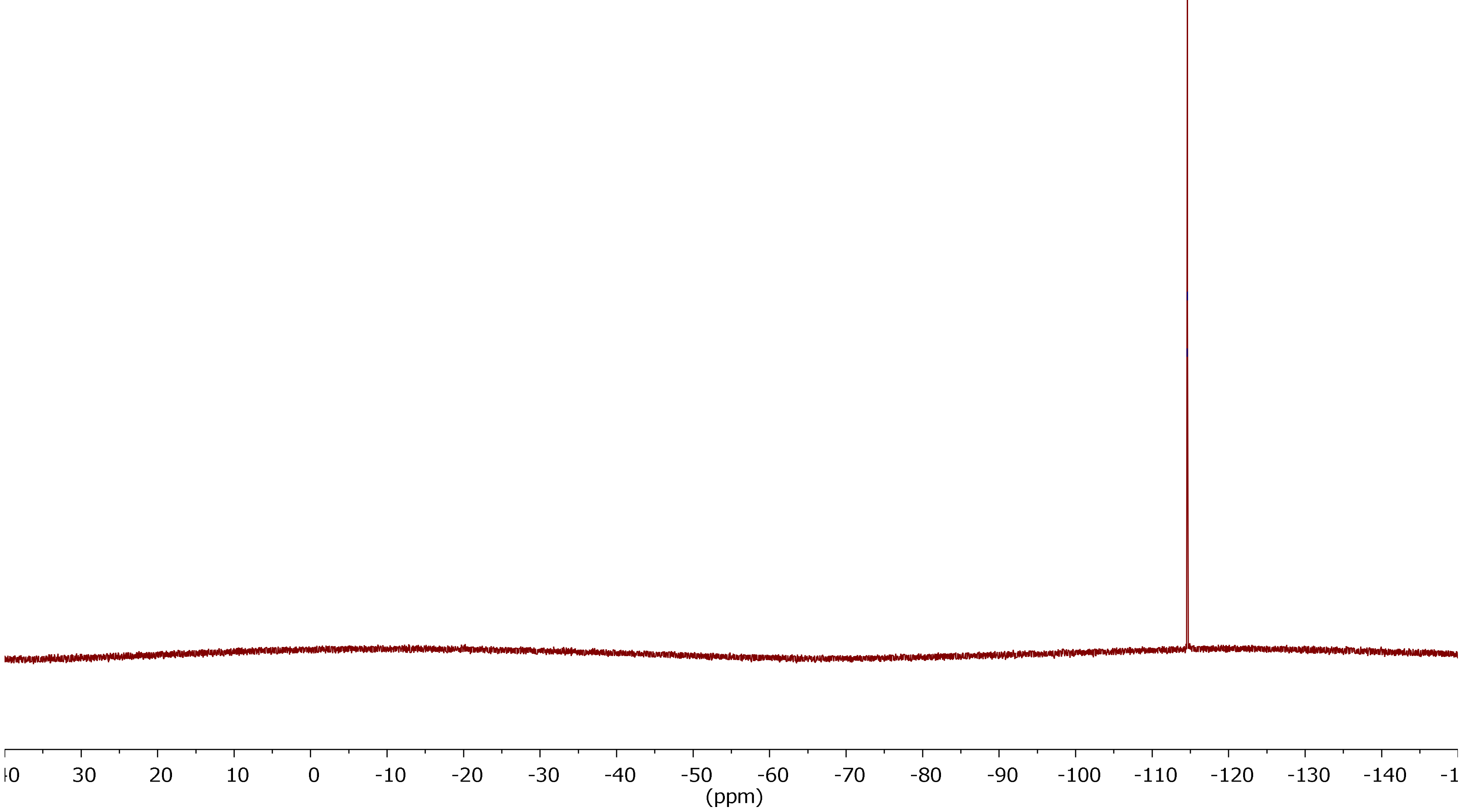

Supplementary Figure 53| ^{19}F-NMR spectrum (470 MHz, $CDCl_3$) for **L14**.

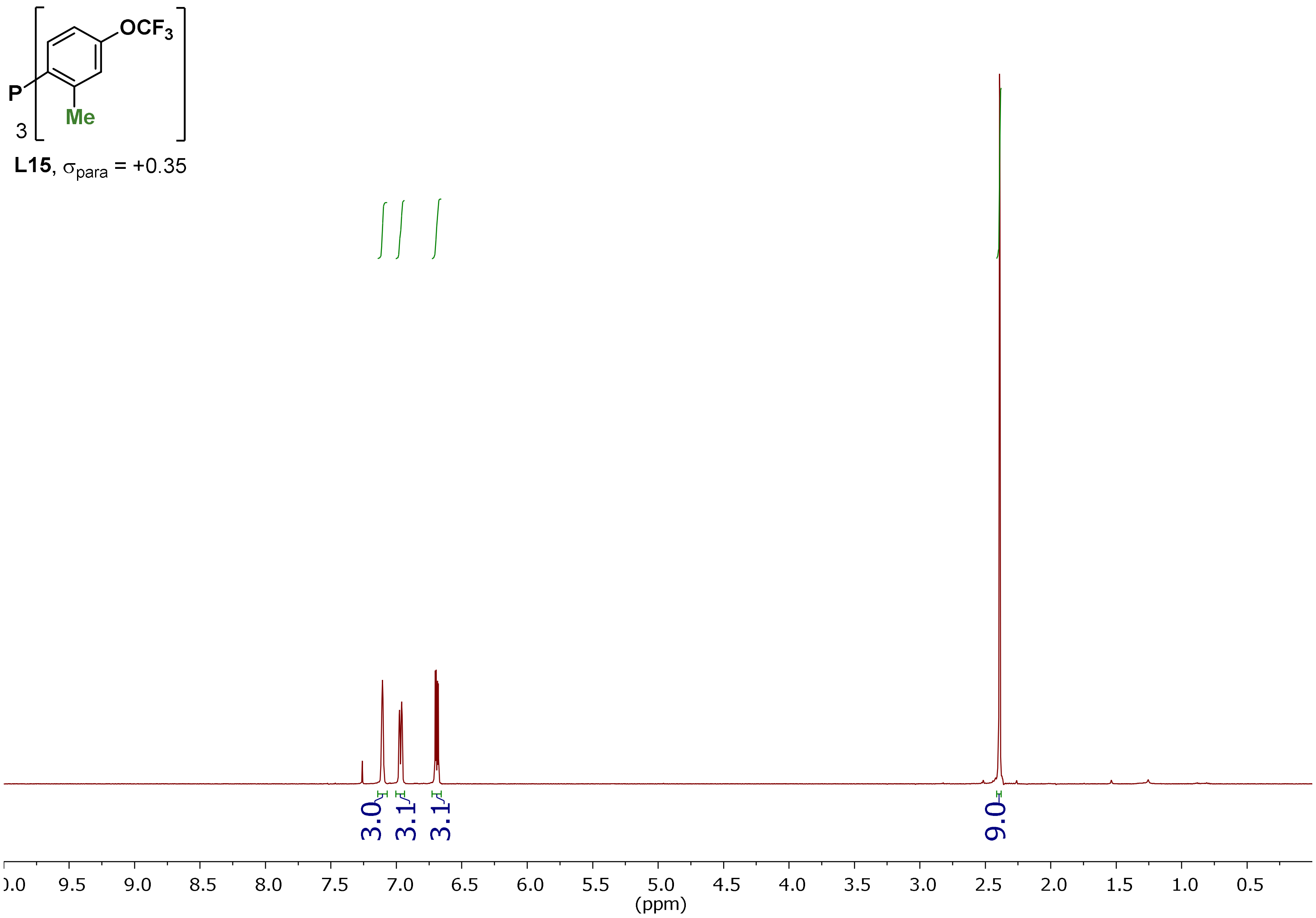

Supplementary Figure 54| ^{1}H-NMR spectrum (500 MHz, $CDCl_3$) for **L15**.

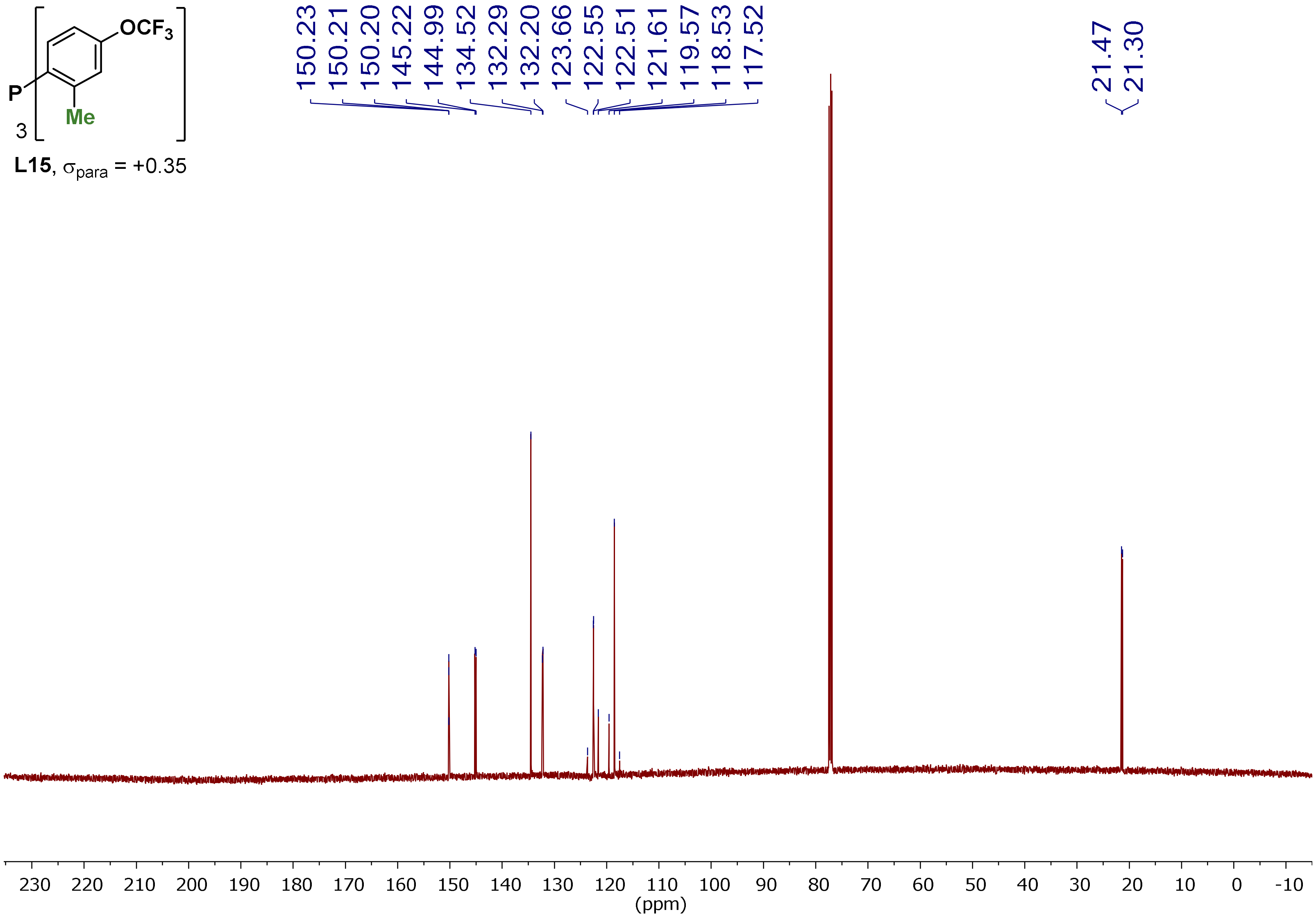

Supplementary Figure 55| ^{13}C-NMR spectrum (126 MHz, $CDCl_3$) for **L15**.

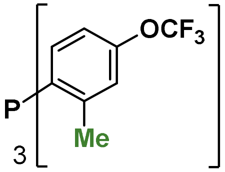

L15, σ_{para} = +0.35

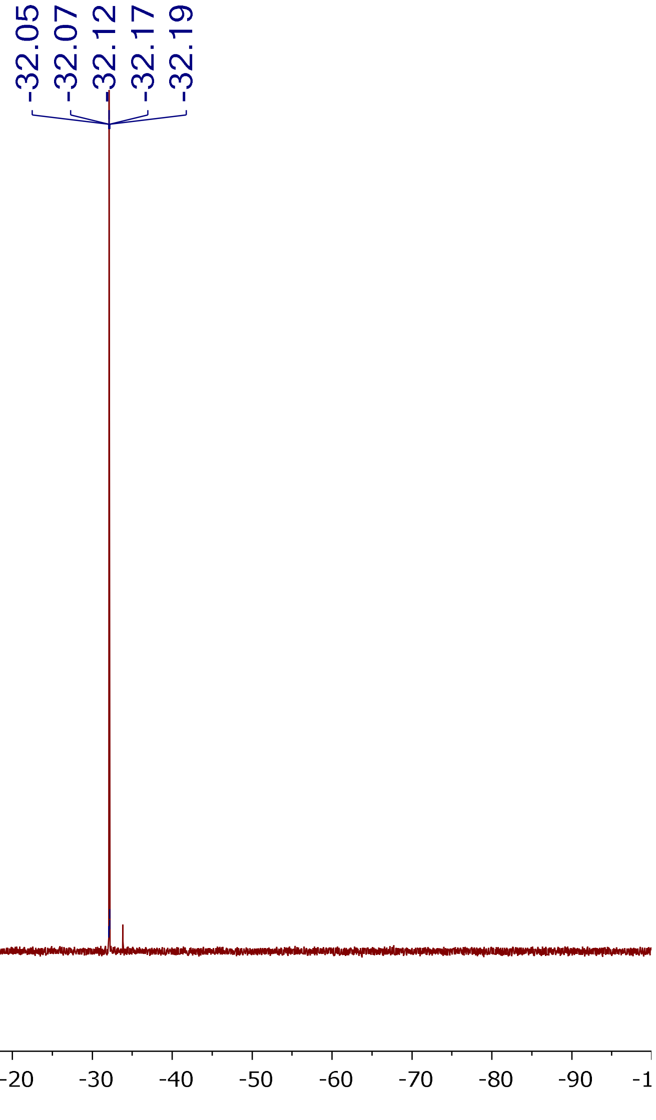

Supplementary Figure 56| ^{31}P-NMR spectrum (202 MHz, C_6D_6) for **L15**.

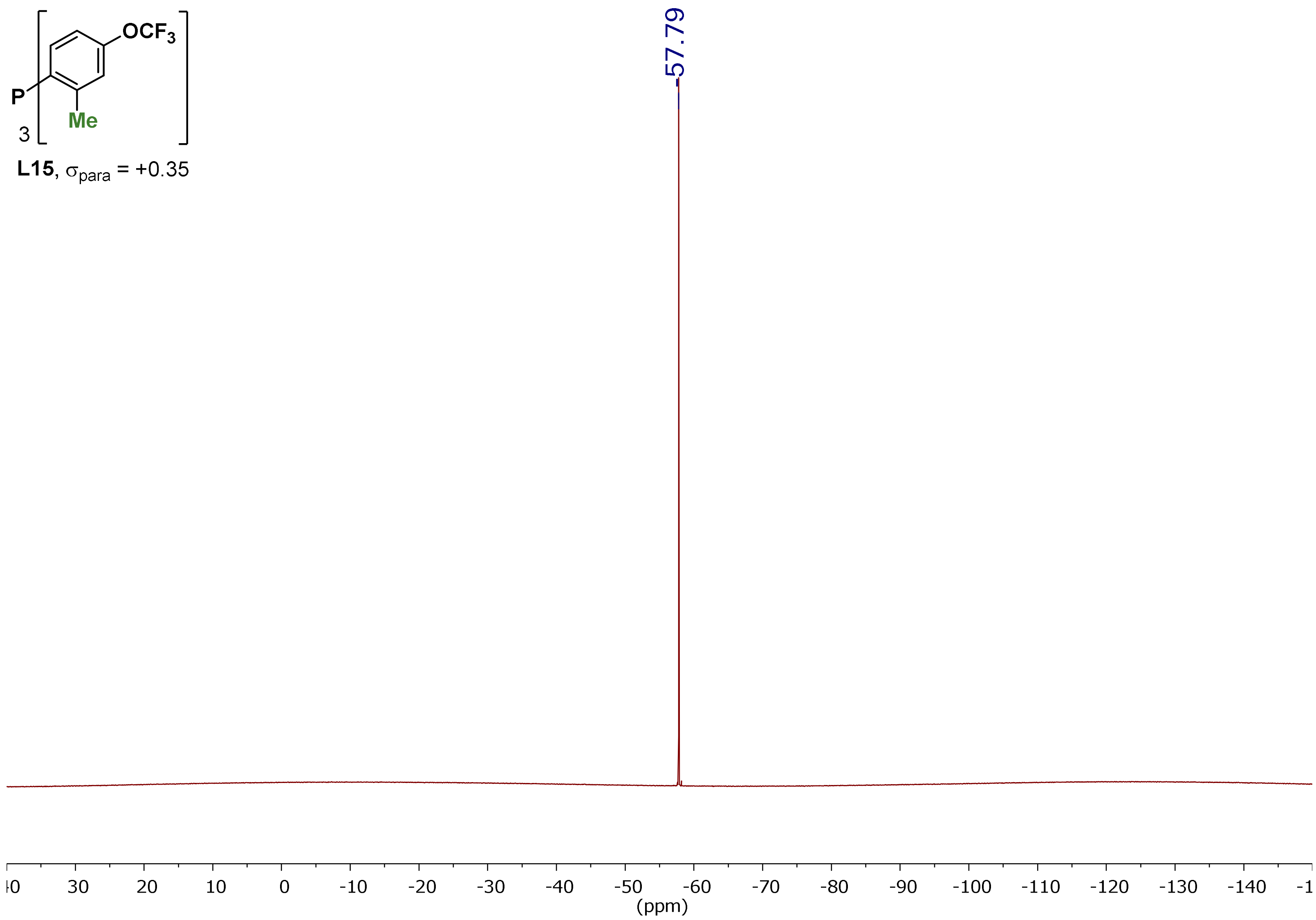

Supplementary Figure 57| ^{19}F-NMR spectrum (470 MHz, C_6D_6) for **L15**.

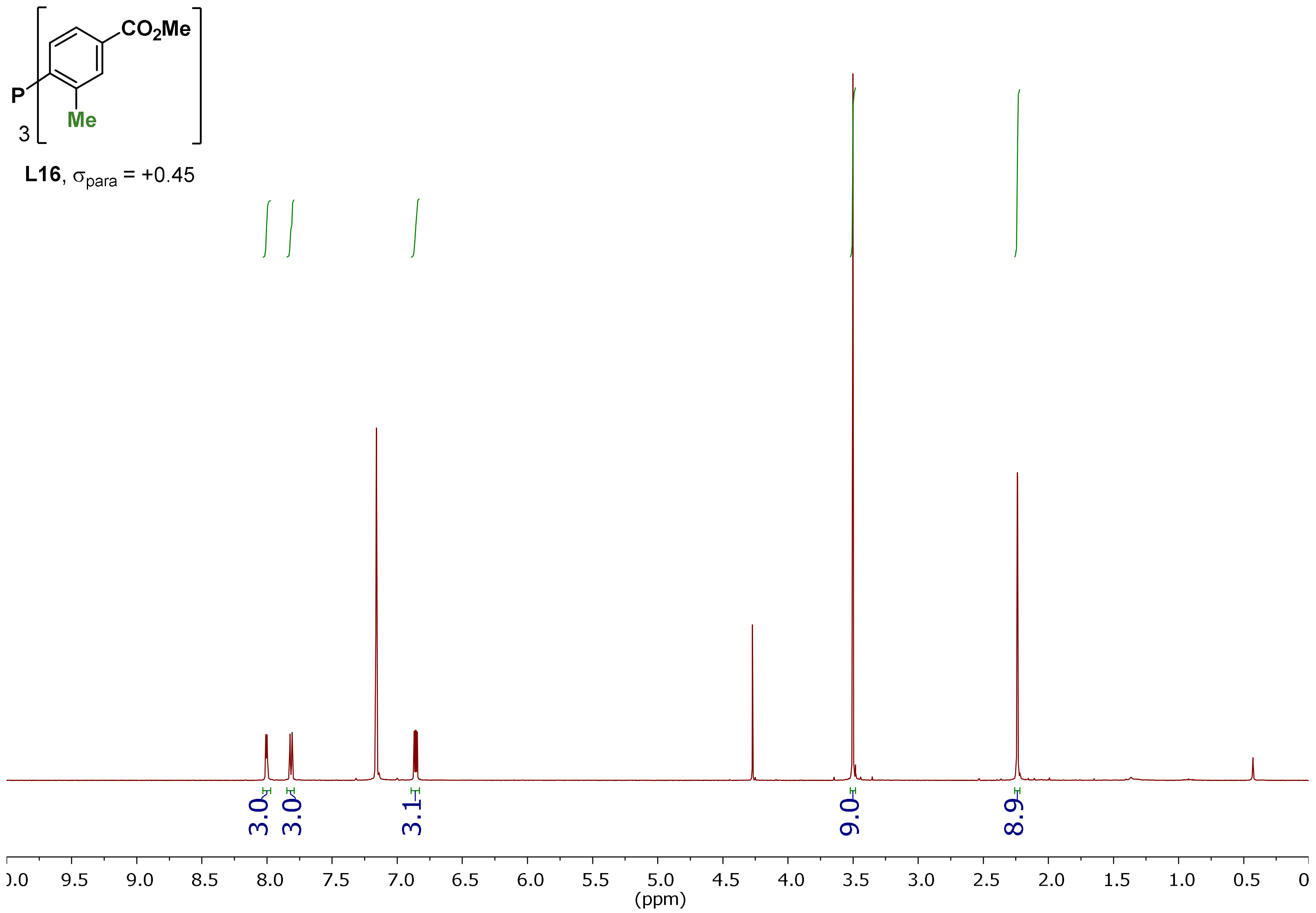

Supplementary Figure 58| ^{1}H-NMR spectrum (500 MHz, C_6D_6) for **L16**.

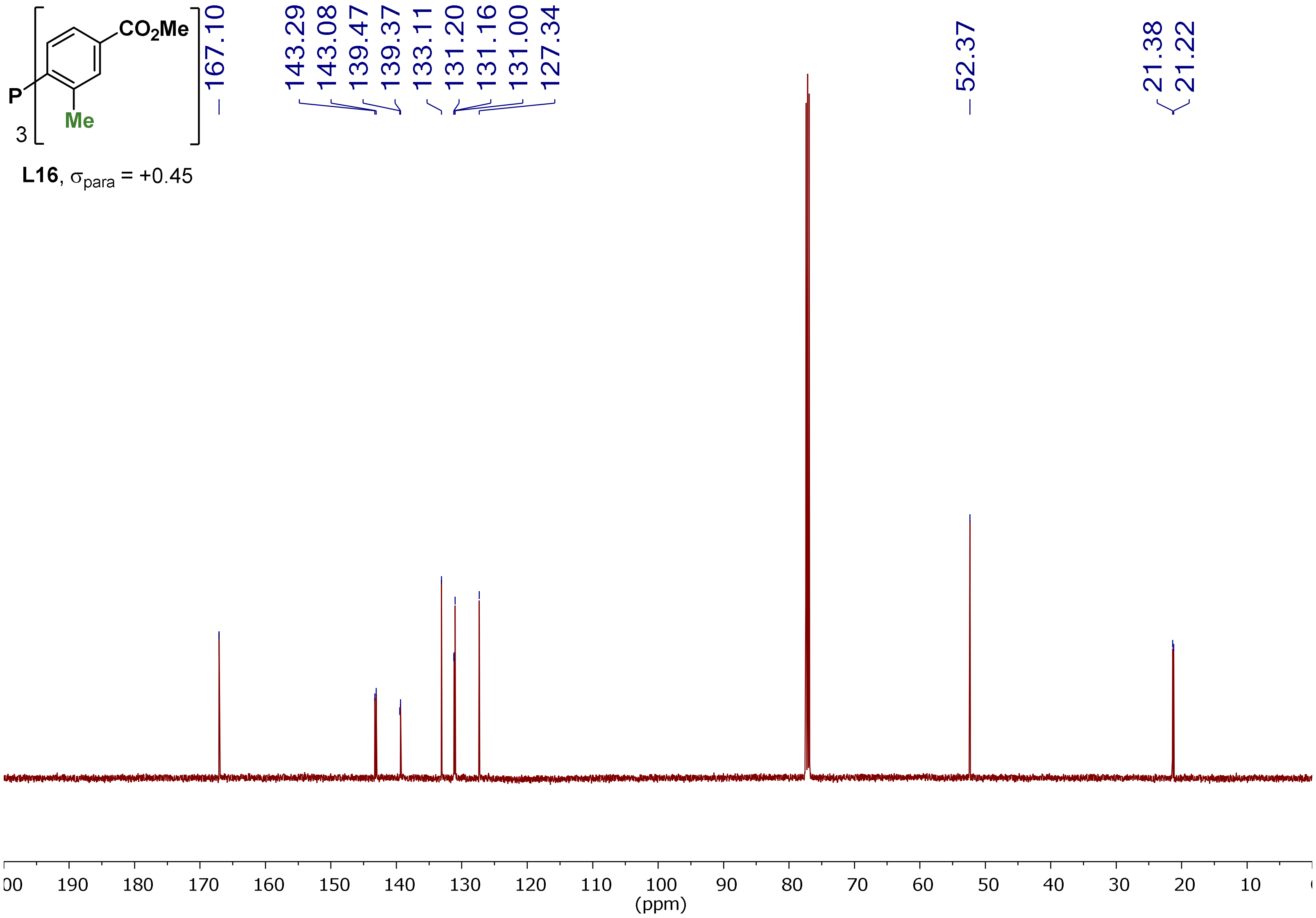

Supplementary Figure 59| ^{13}C-NMR spectrum (126 MHz, $CDCl_3$) for **L16**.

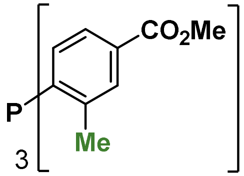

L16, σ_{para} = +0.45

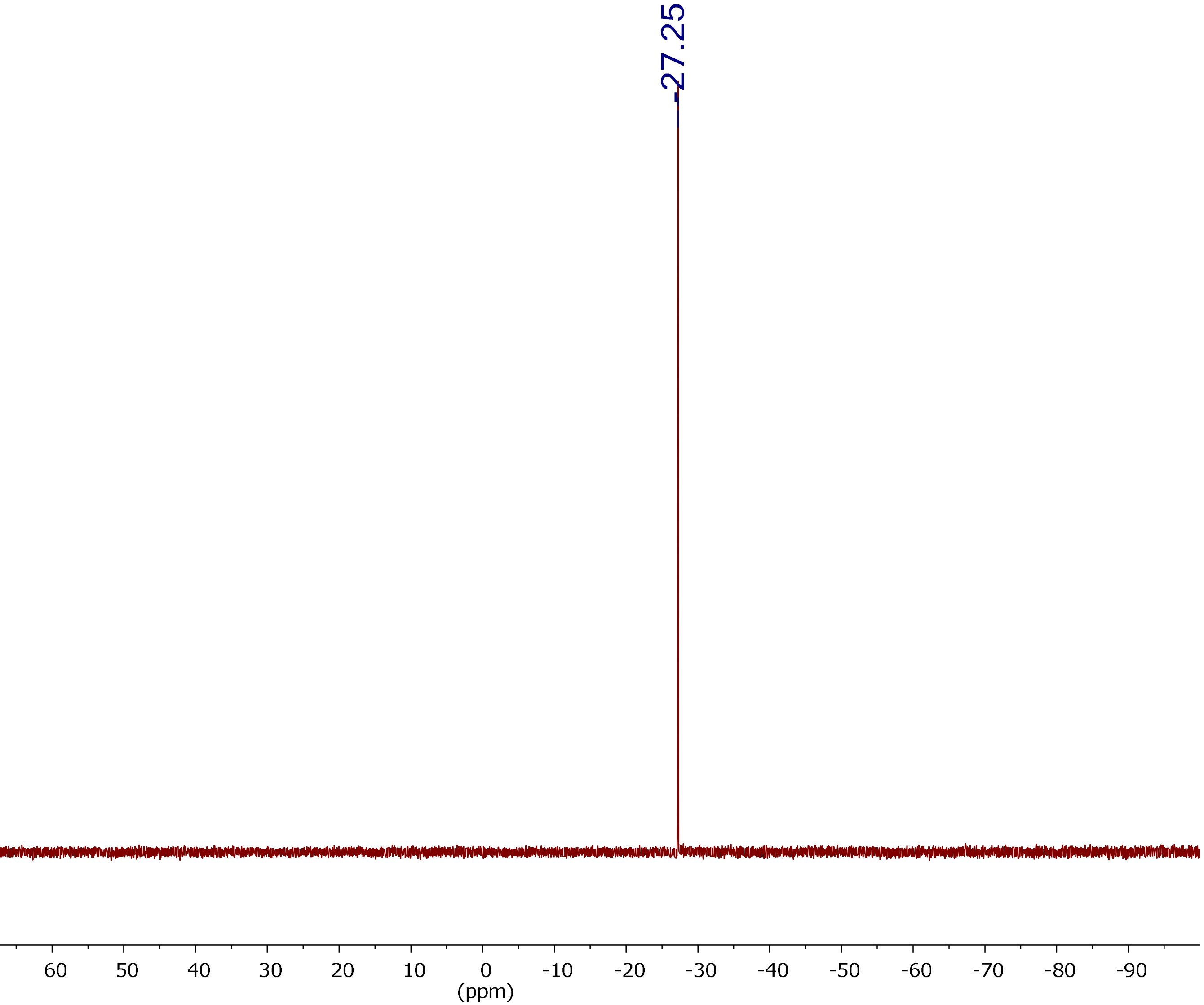

Supplementary Figure 60| ^{31}P-NMR spectrum (202 MHz, C_6D_6) for **L16**.

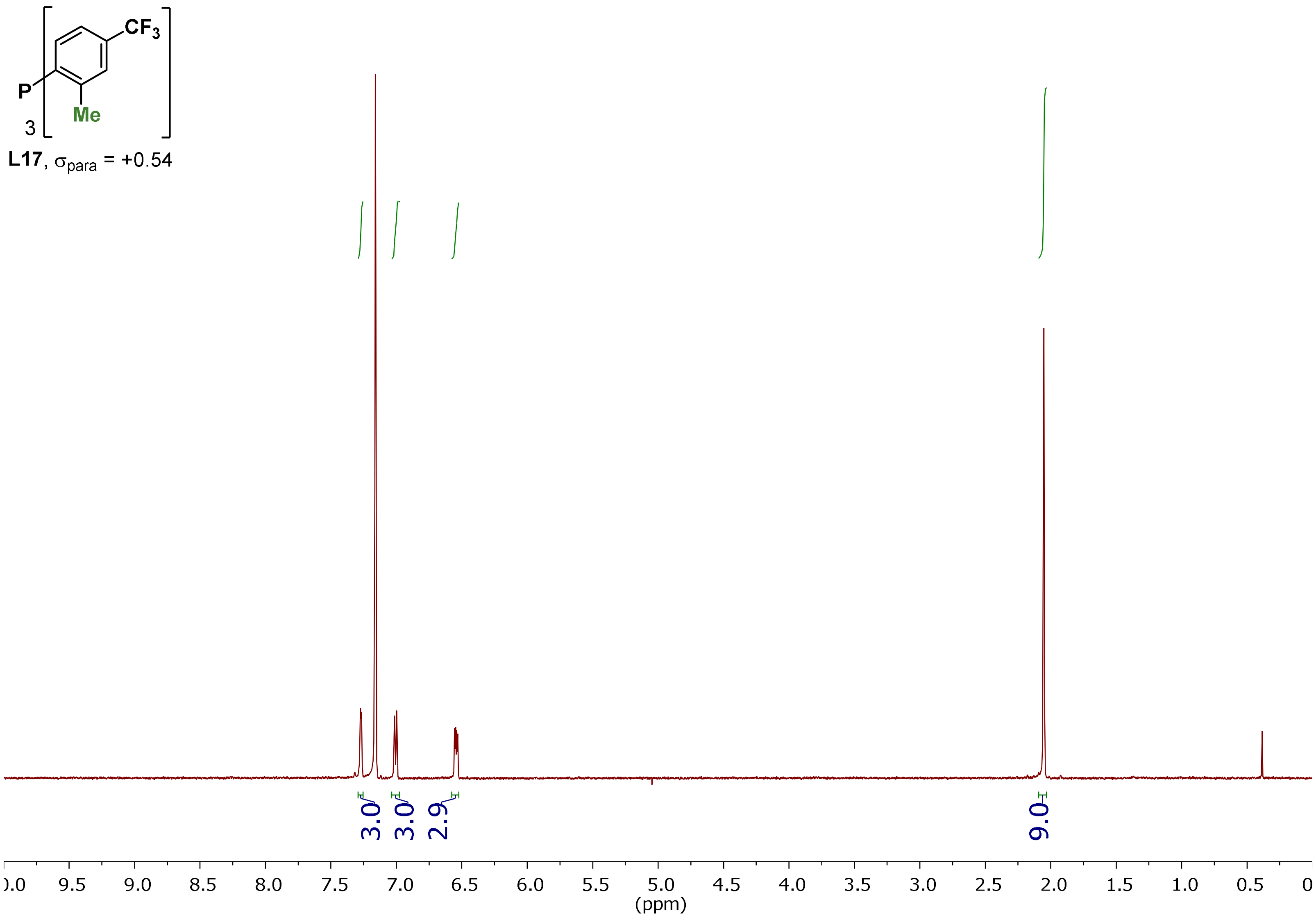

Supplementary Figure 61| ^{1}H-NMR spectrum (500 MHz, C_6D_6) for **L17**.

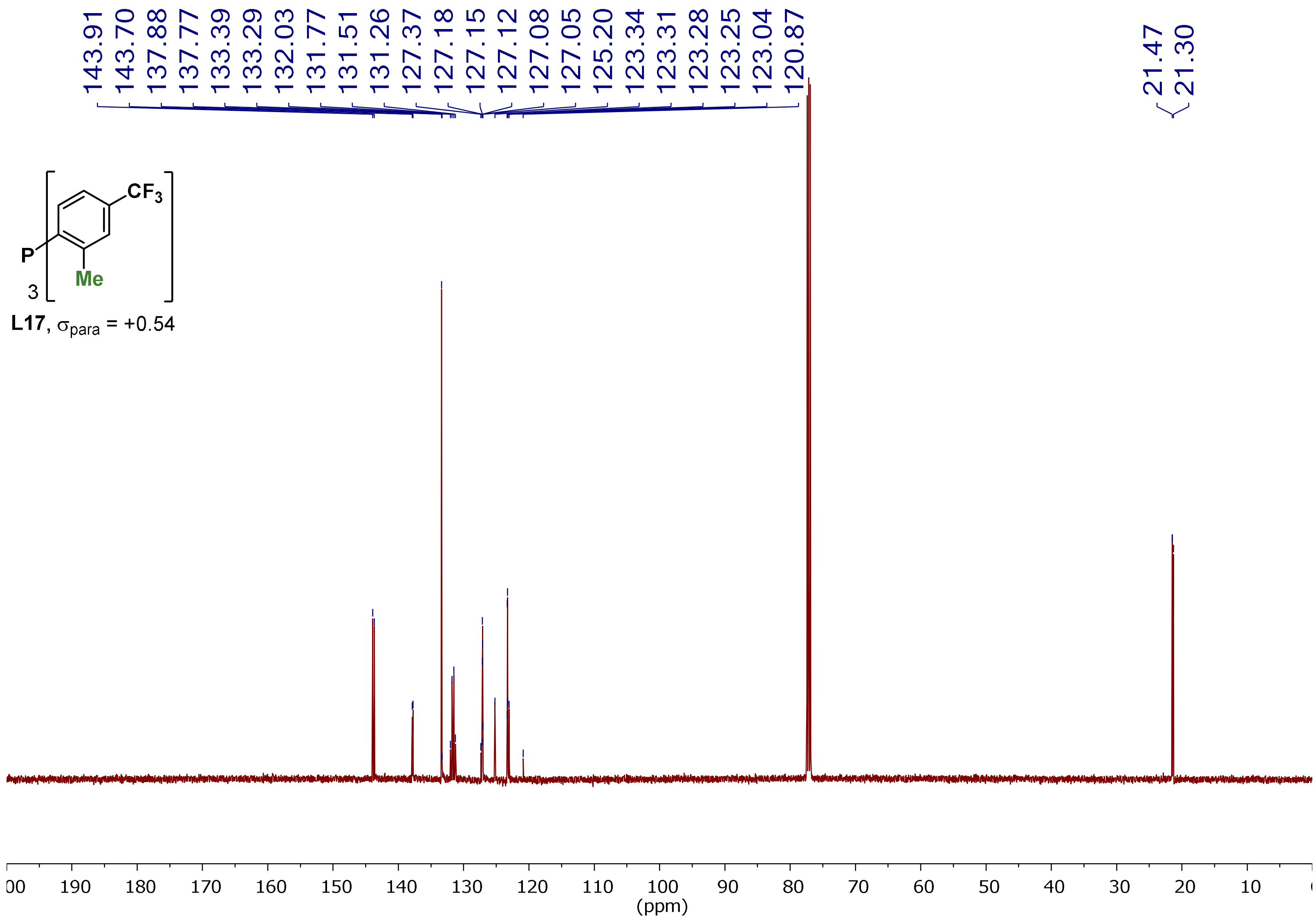

Supplementary Figure 62| ^{13}C-NMR spectrum (126 MHz, $CDCl_3$) for **L17**.

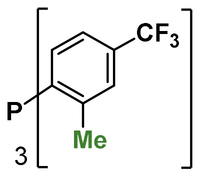

L17, σ_{para} = +0.54

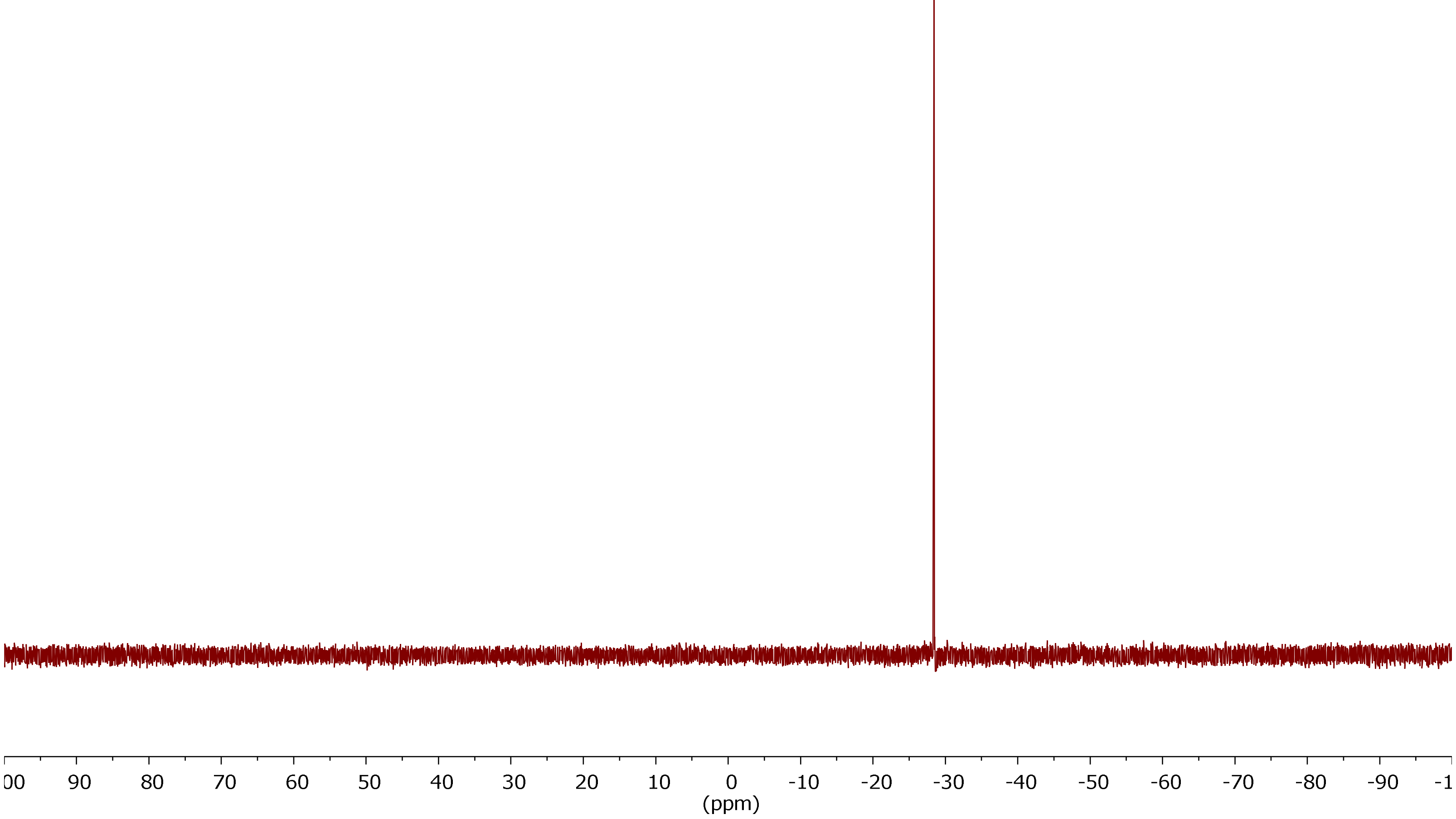

Supplementary Figure 63| ^{31}P-NMR spectrum (202 MHz, C_6D_6) for **L17**.

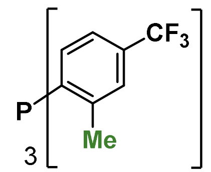
L17, σ_{para} = +0.54

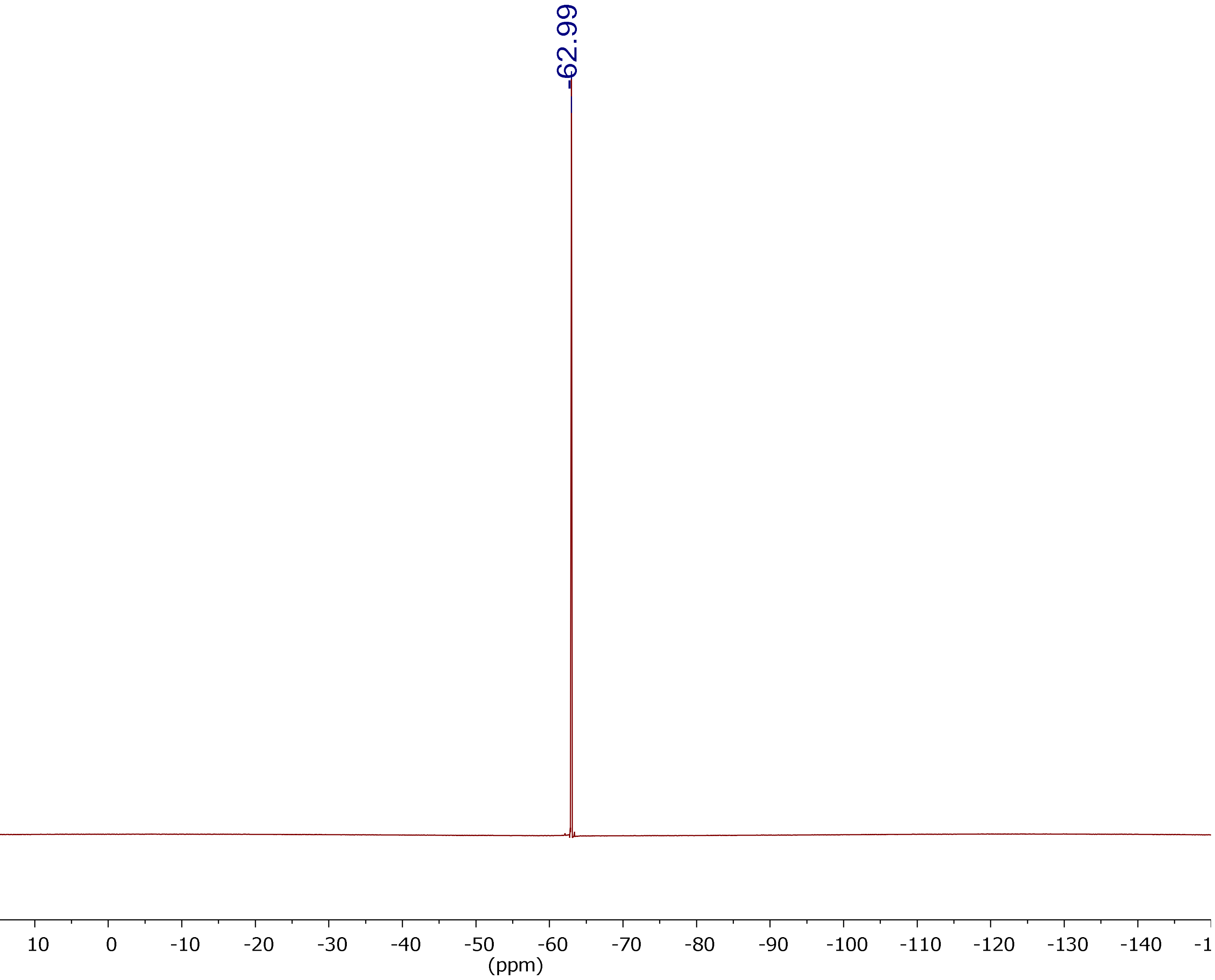

Supplementary Figure 64| ^{19}F-NMR spectrum (470 MHz, C_6D_6) for **L17**.

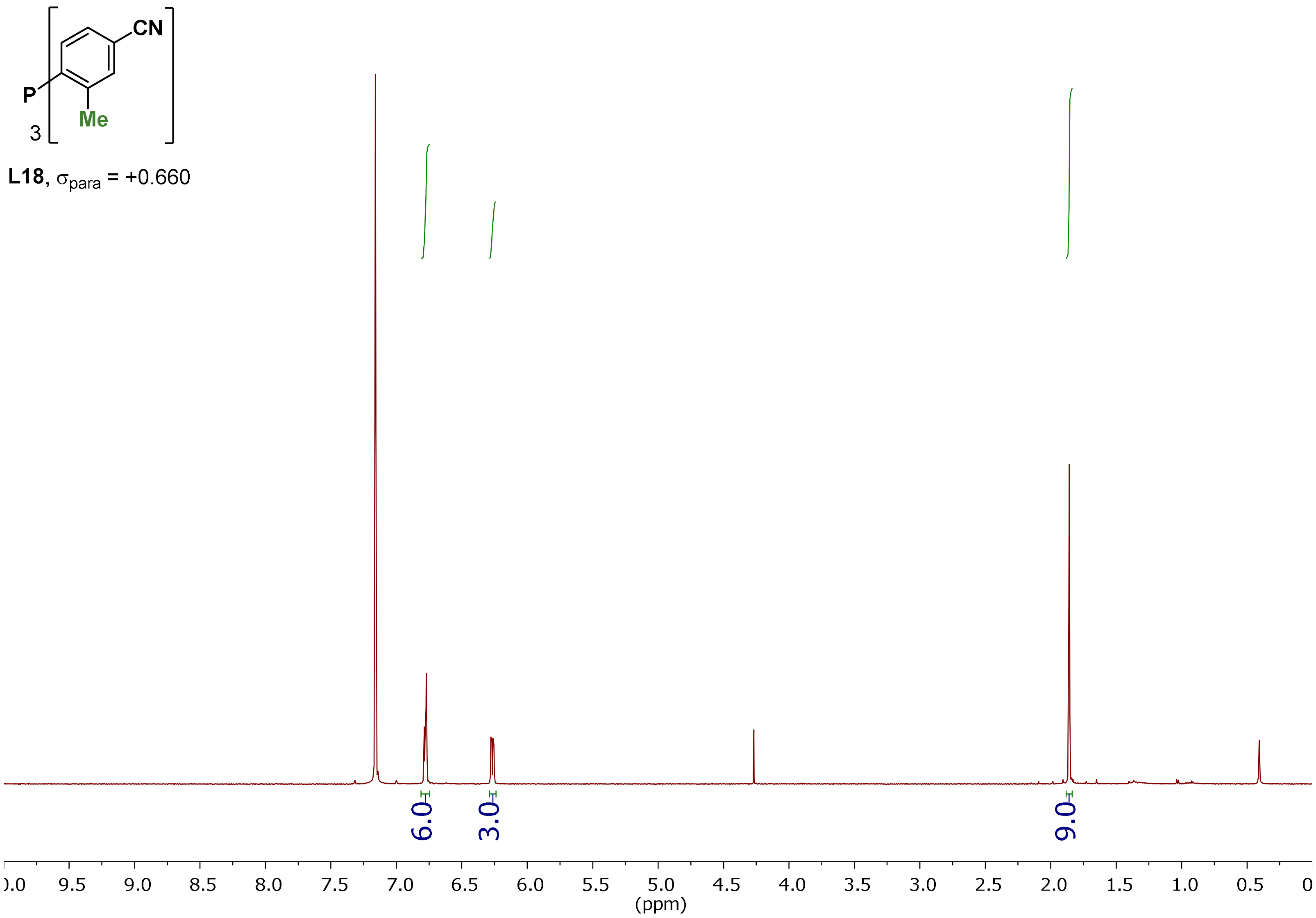

Supplementary Figure 65| ^{1}H-NMR spectrum (500 MHz, C_6D_6) for **L18**.

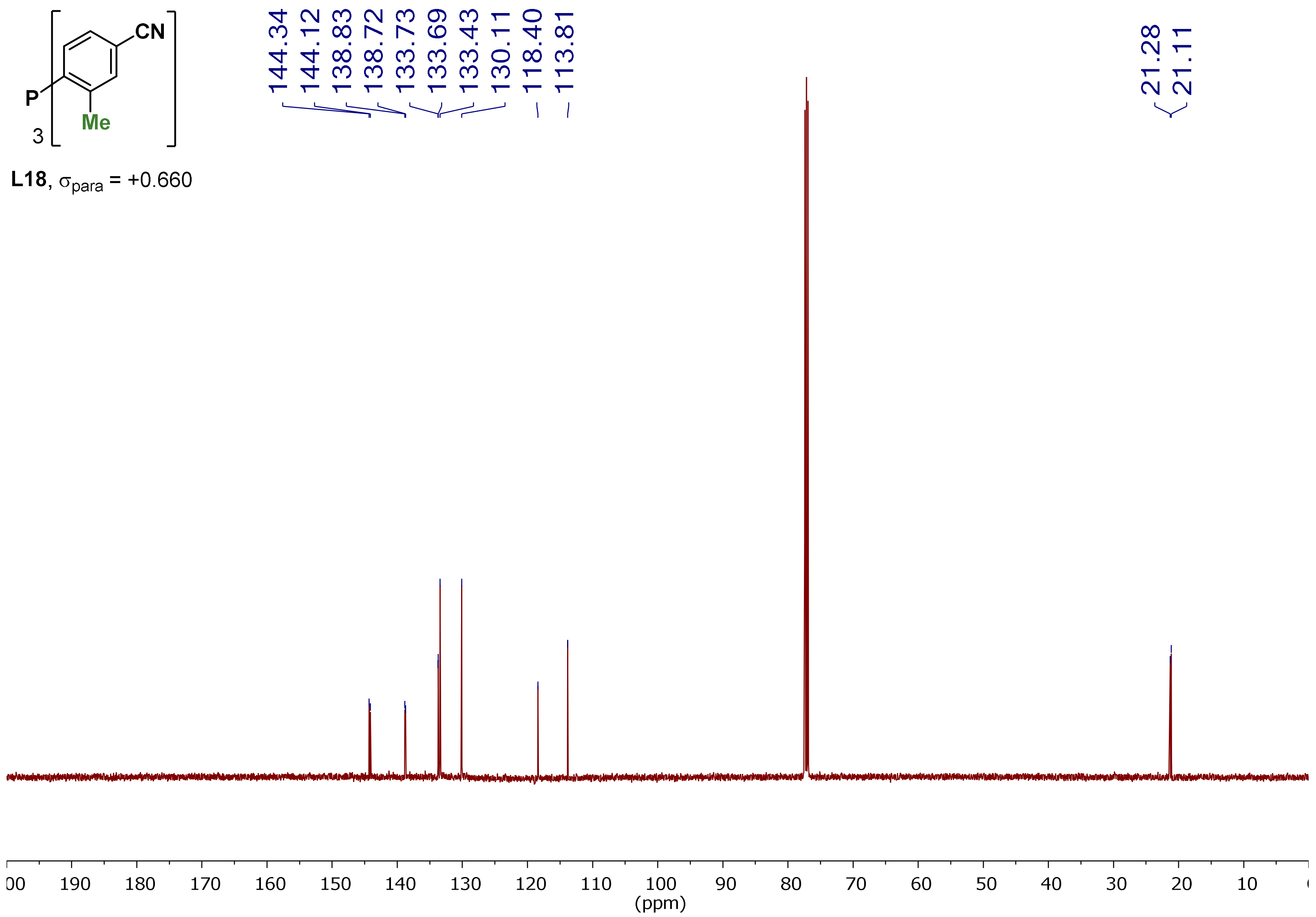

Supplementary Figure 66| ^{13}C-NMR spectrum (126 MHz, $CDCl_3$) for **L18**.

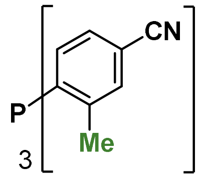

L18, σ_{para} = +0.660

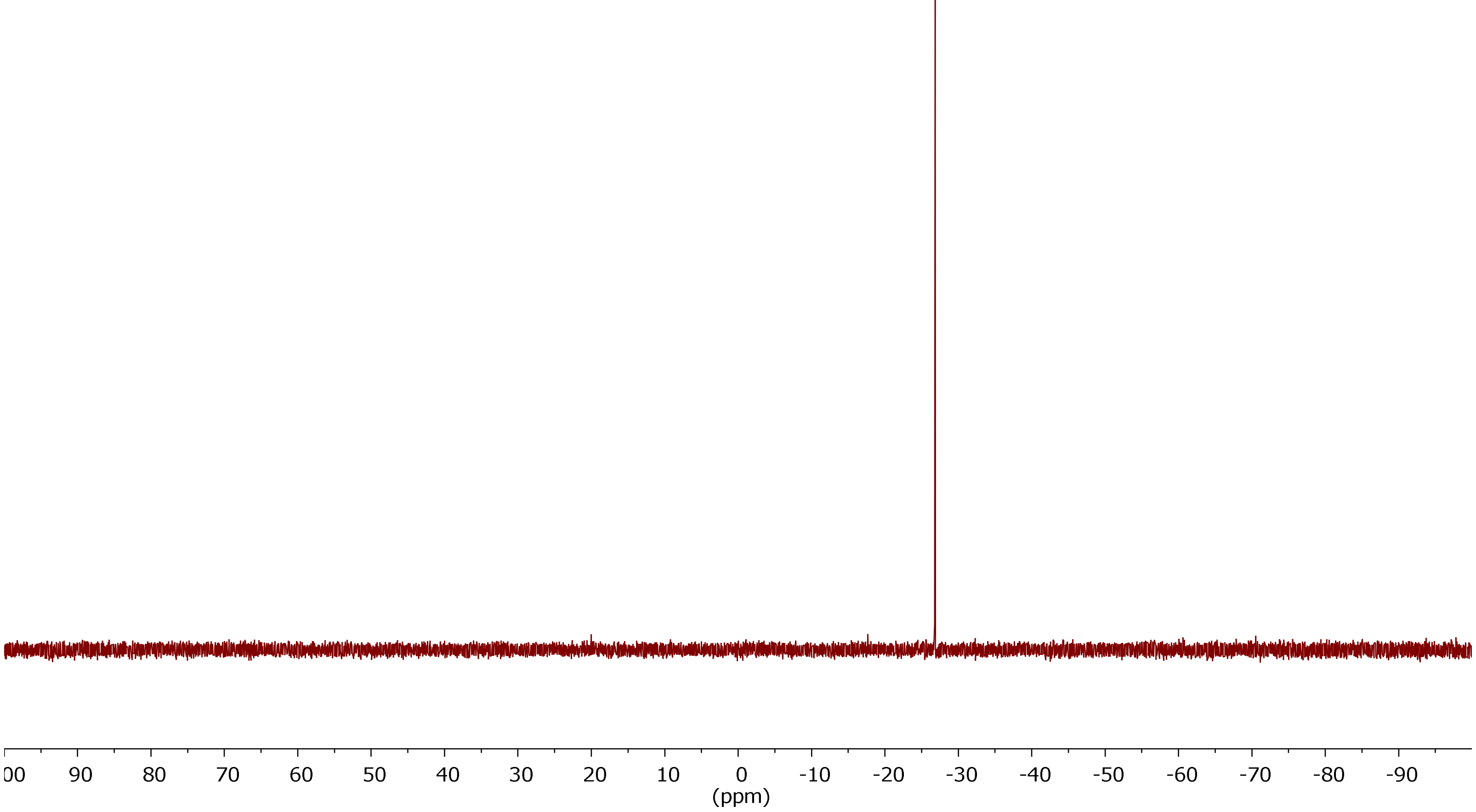

Supplementary Figure 67| ^{31}P-NMR spectrum (202 MHz, C_6D_6) for **L18**.

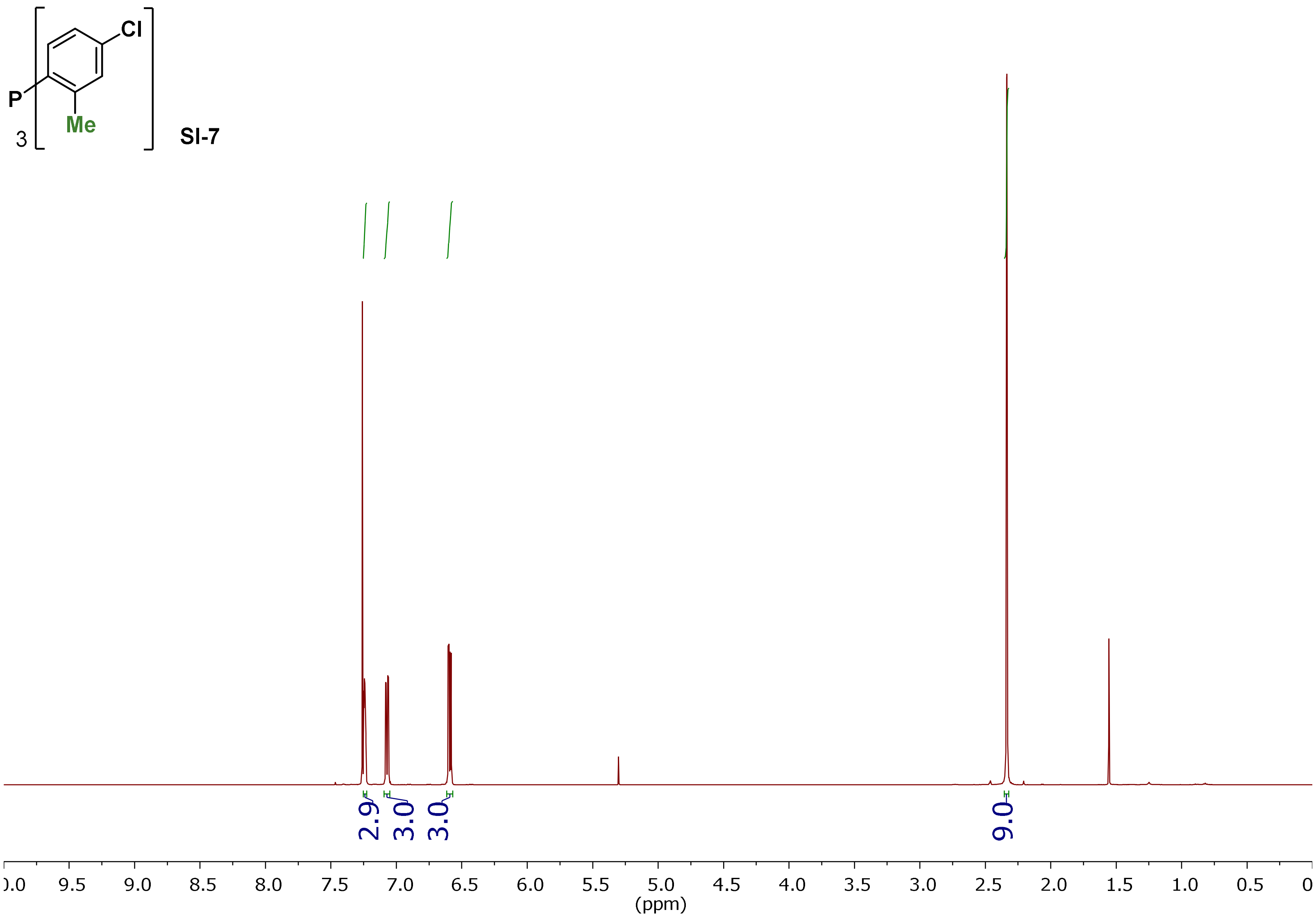

Supplementary Figure 68| ^{1}H-NMR spectrum (500 MHz, $CDCl_3$) for **SI-7**.

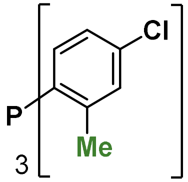

SI-7

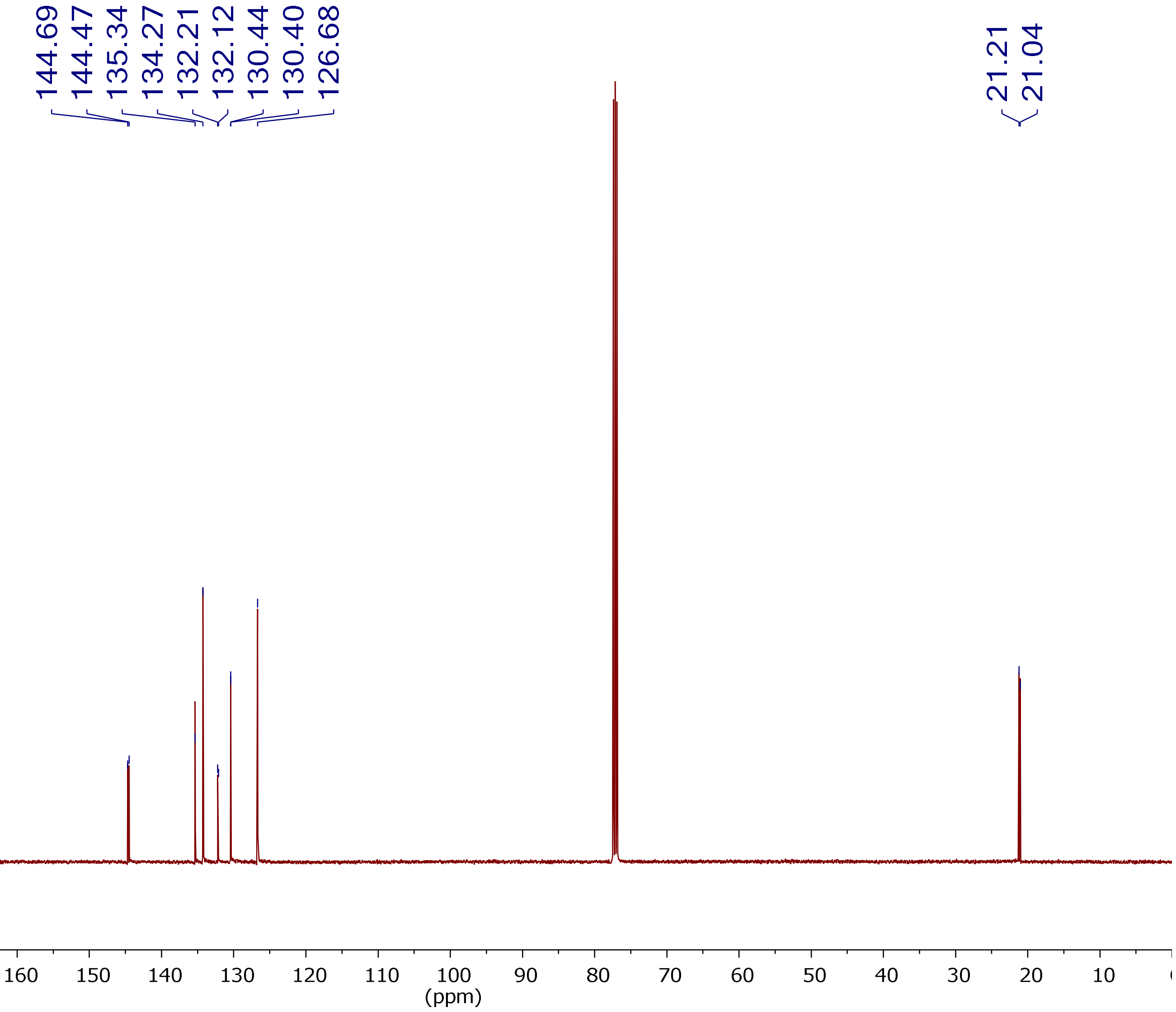

Supplementary Figure 69| ^{13}C-NMR spectrum (126 MHz, $CDCl_3$) for **SI-7**.

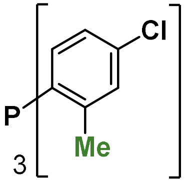

SI-7

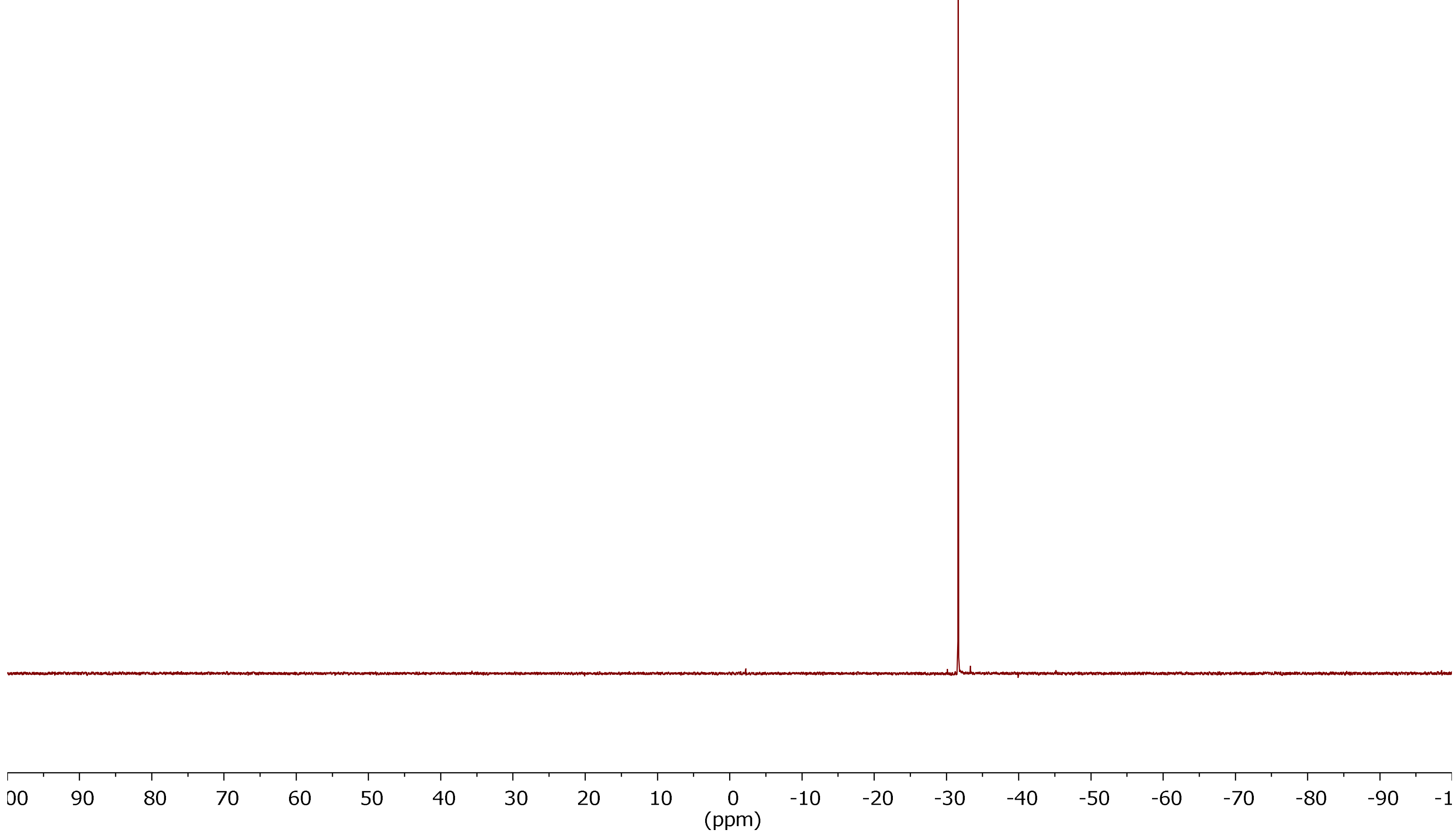

Supplementary Figure 70| ^{31}P-NMR spectrum (202 MHz, C_6D_6) for **SI-7**.

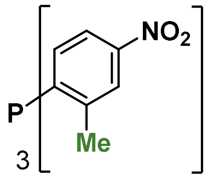

L19, σ_{para} = +0.778

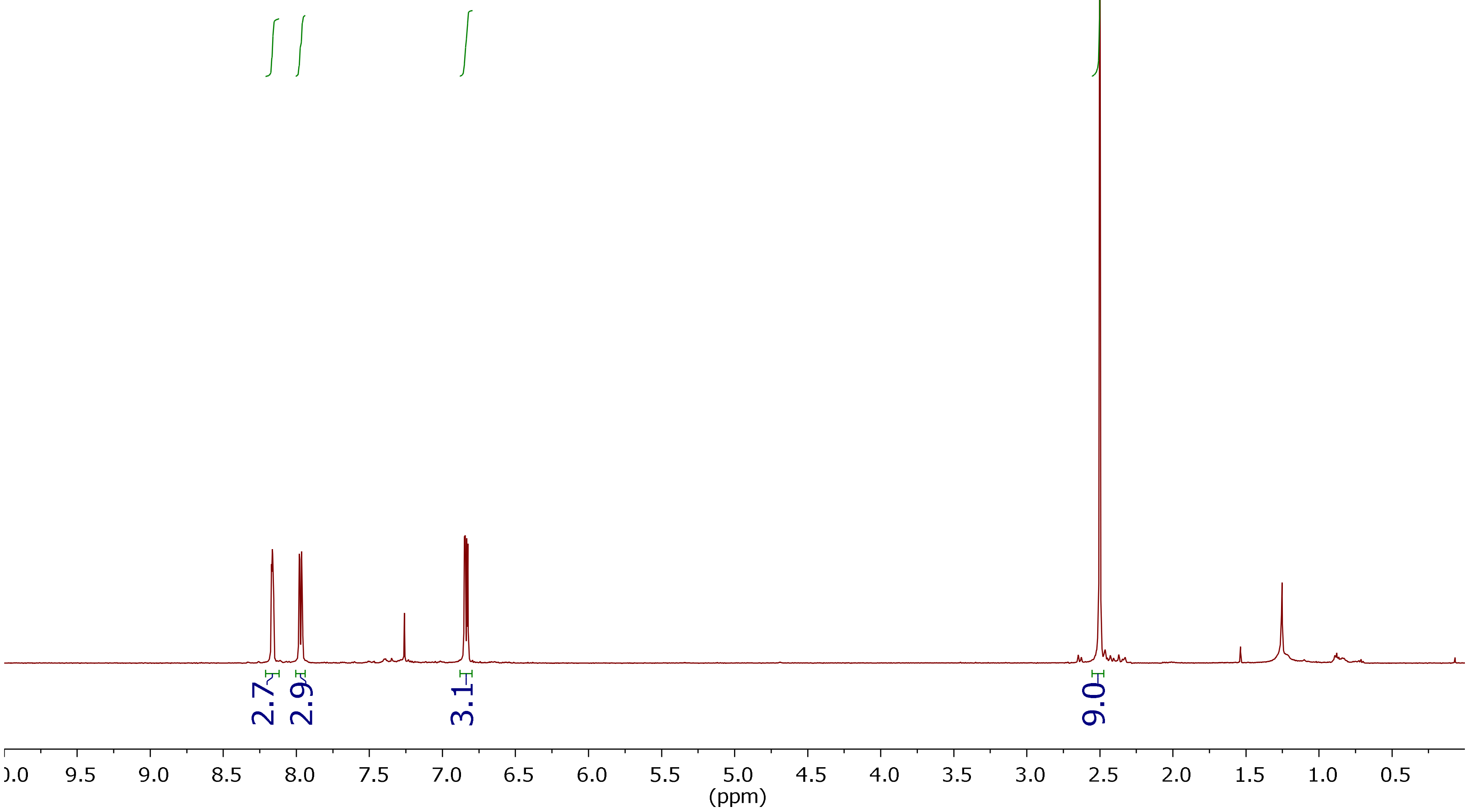

Supplementary Figure 71| ^{1}H-NMR spectrum (500 MHz, $CDCl_3$) for **L19**.

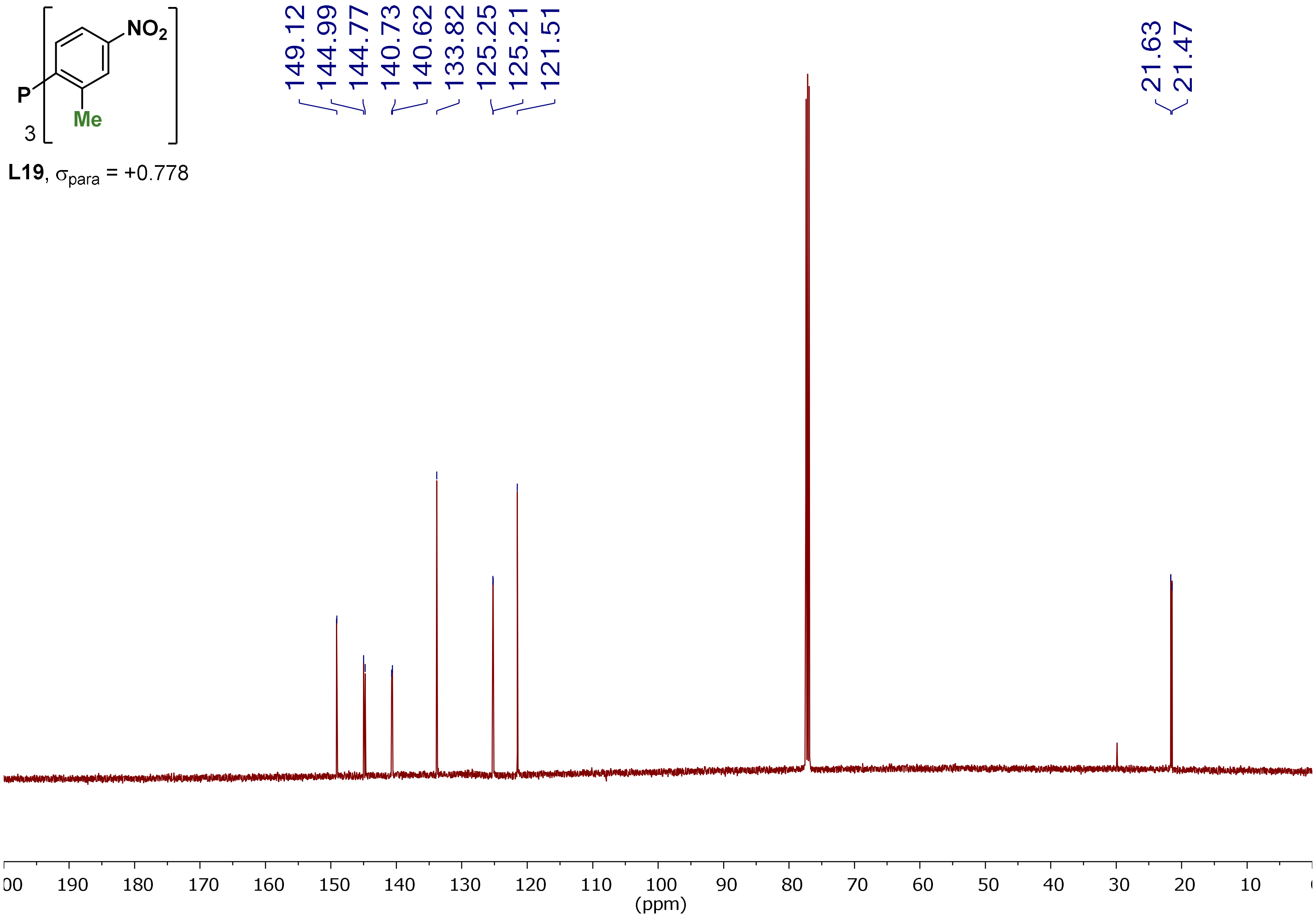

Supplementary Figure 72| ^{13}C-NMR spectrum (126 MHz, $CDCl_3$) for **L19**.

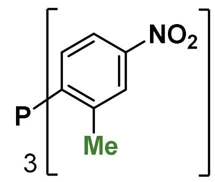

L19, σ_{para} = +0.778

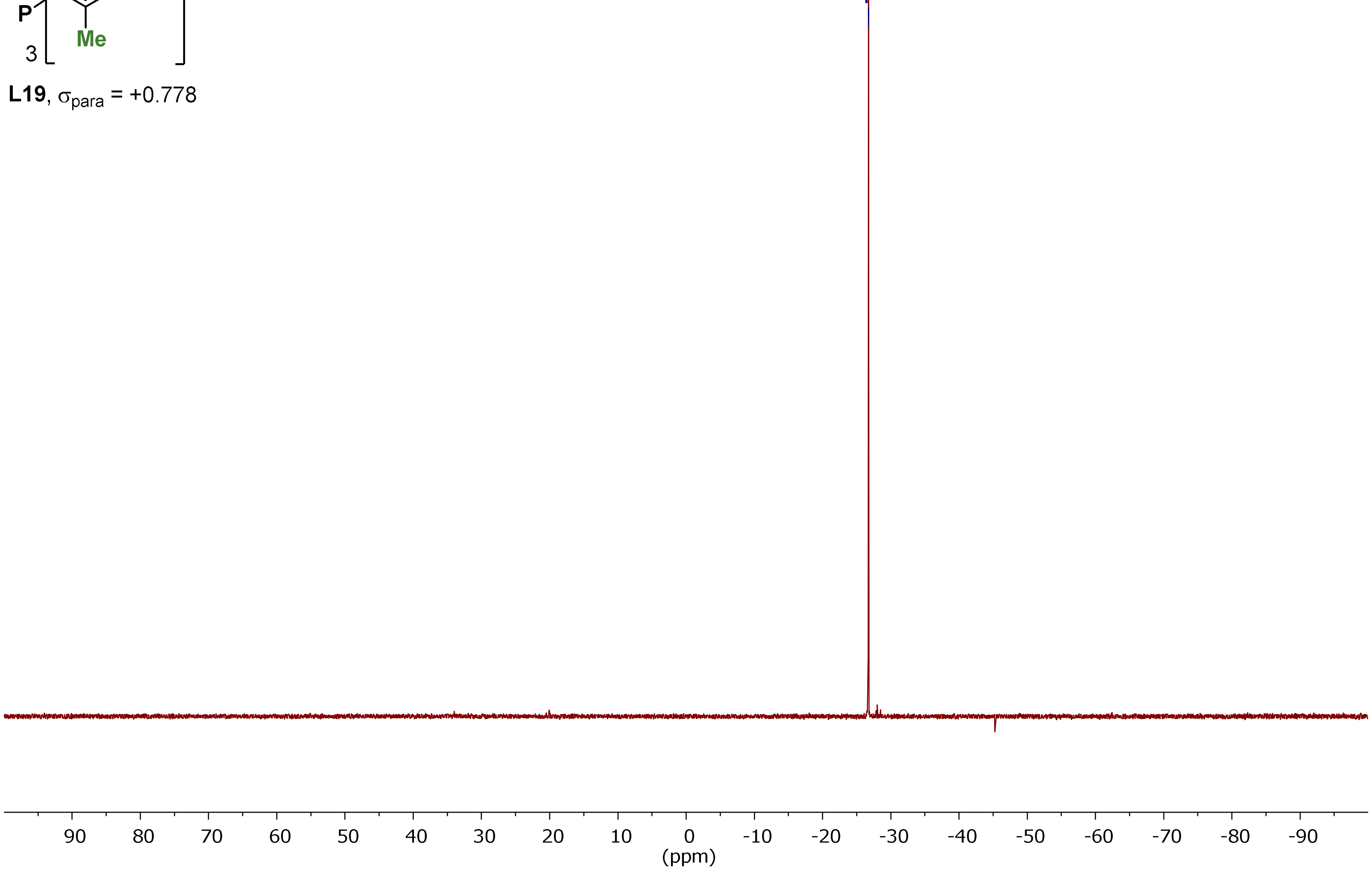

Supplementary Figure 73| ^{31}P-NMR spectrum (202 MHz, $CDCl_3$) for **L19**.

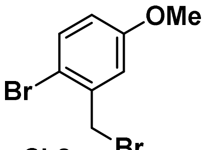

SI-8

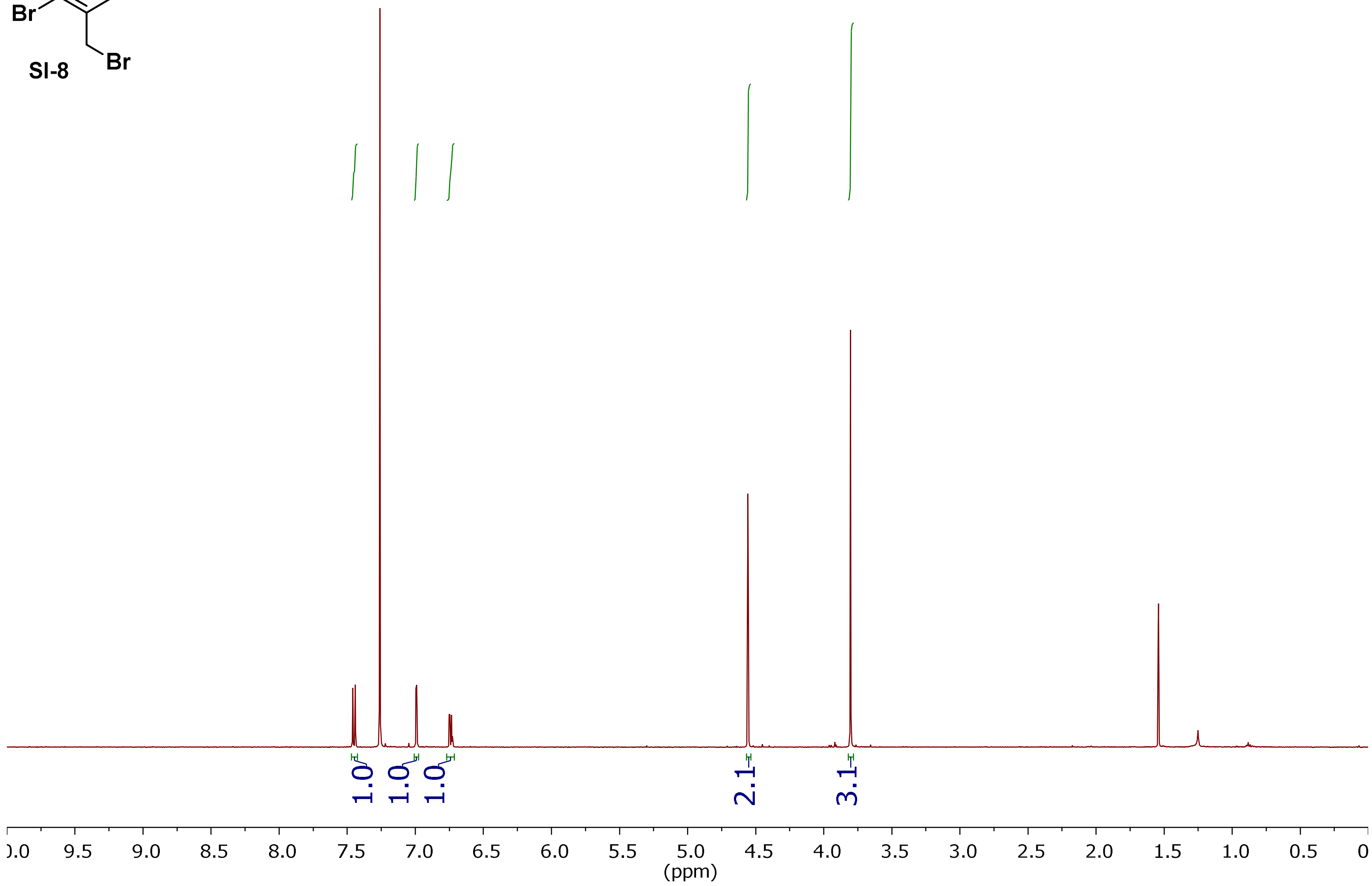

Supplementary Figure 74| ^{1}H-NMR spectrum (500 MHz, $CDCl_3$) for **SI-8**.

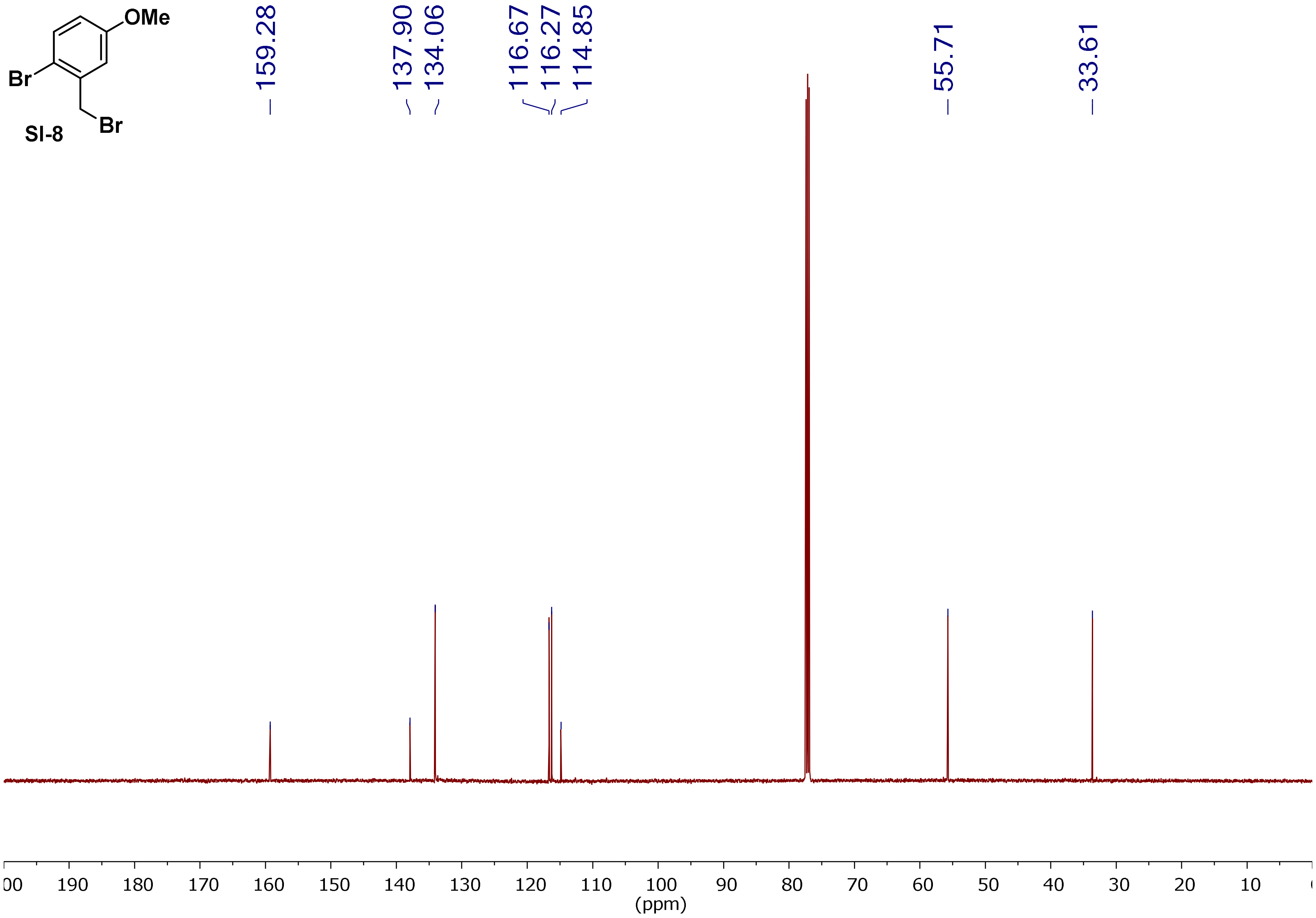

Supplementary Figure 75| ^{13}C-NMR spectrum (126 MHz, $CDCl_3$) for **SI-8**.

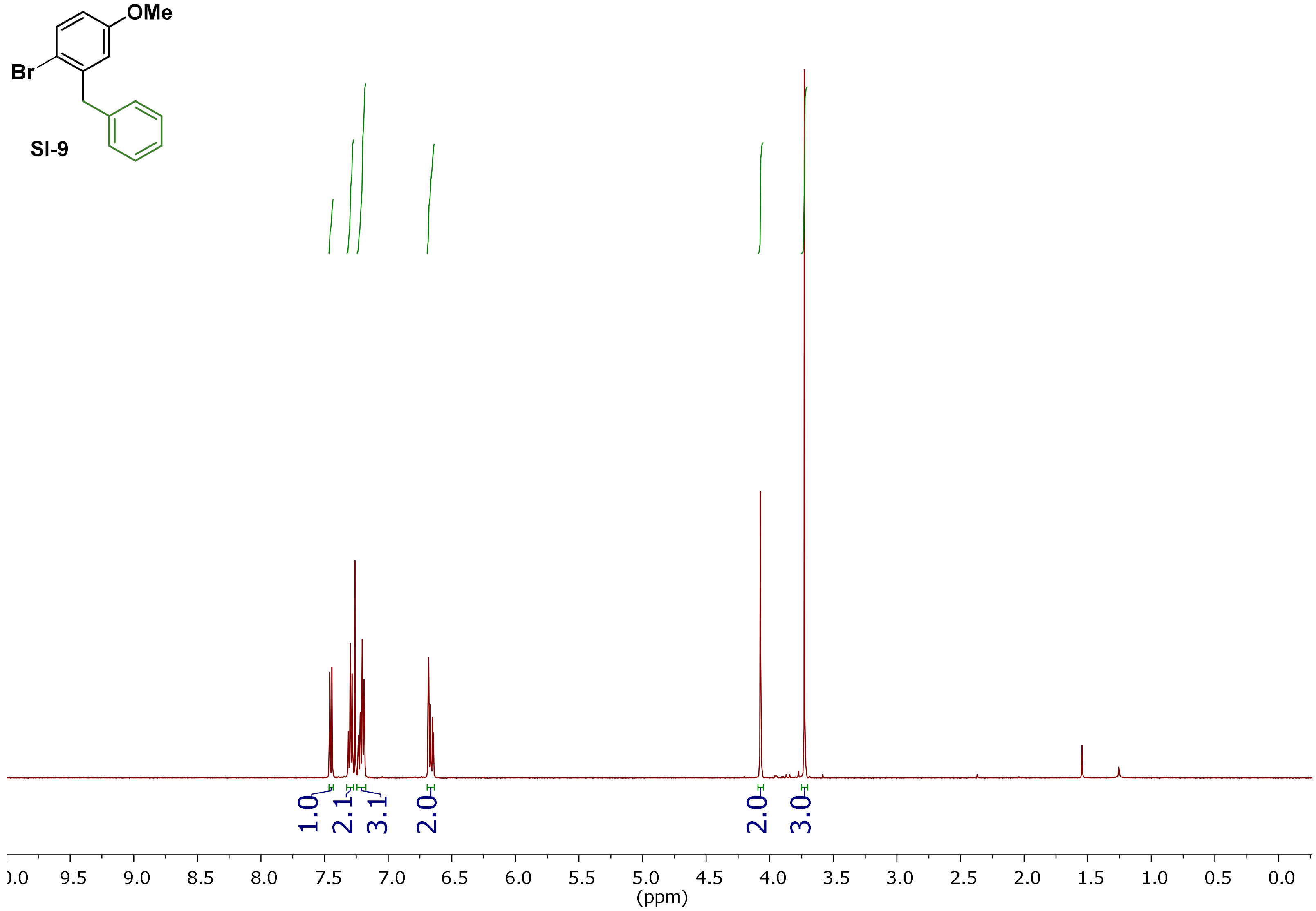

Supplementary Figure 76| ^{1}H-NMR spectrum (500 MHz, $CDCl_3$) for **SI-9**.

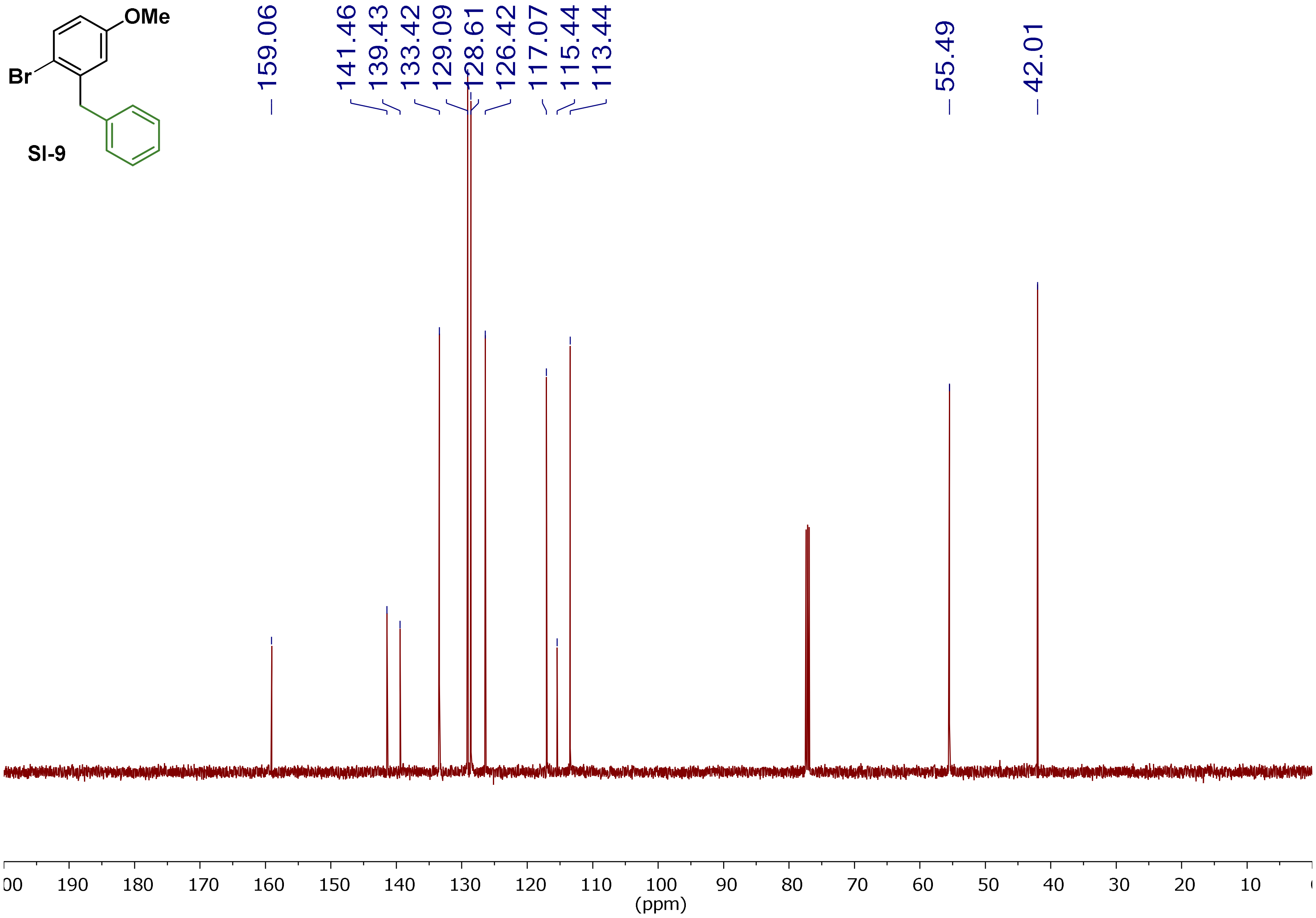

Supplementary Figure 77| ^{13}C-NMR spectrum (126 MHz, $CDCl_3$) for **SI-9**.

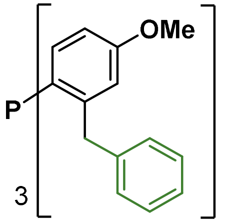

L20, σ_{para} = -0.268

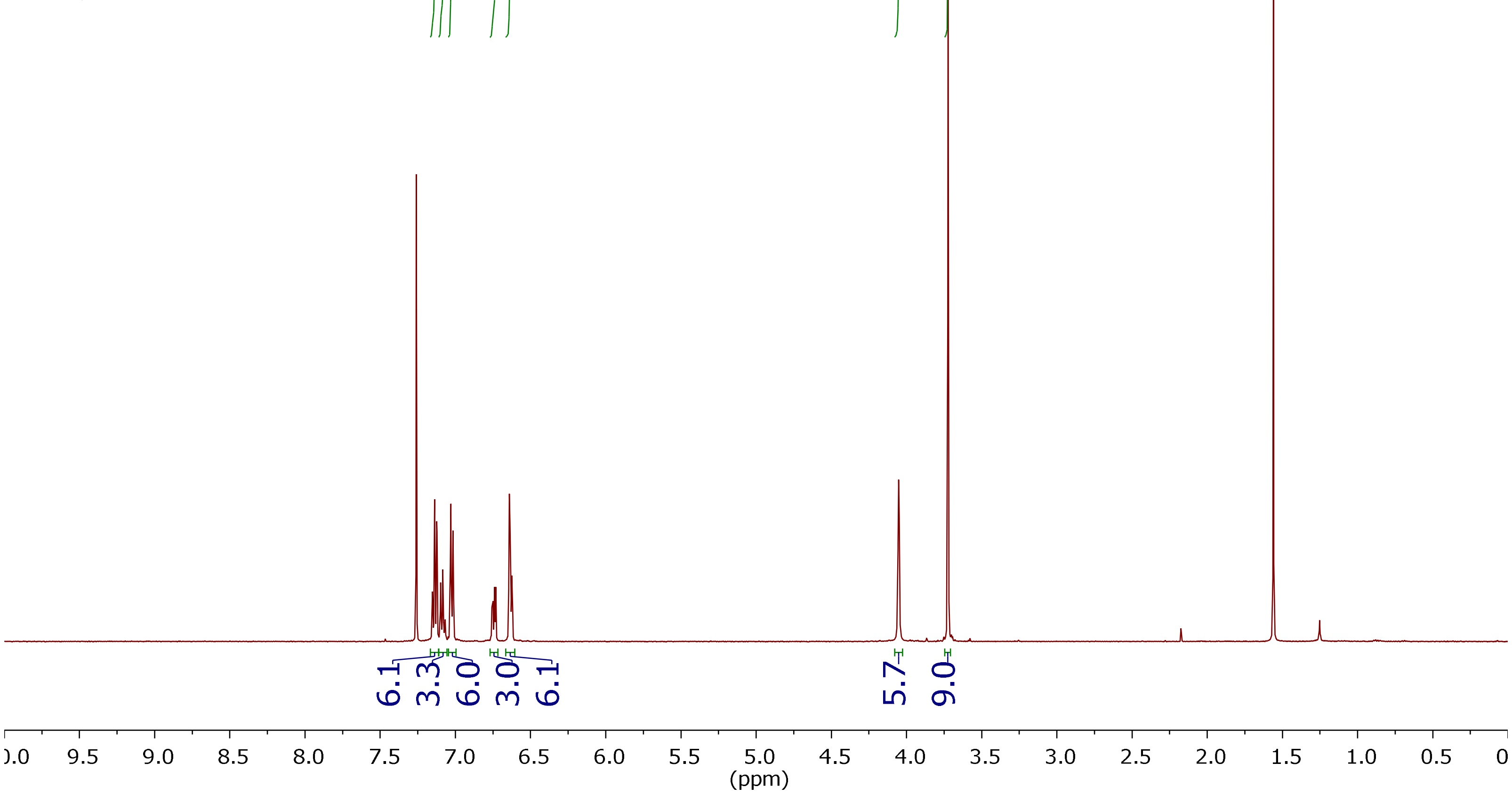

Supplementary Figure 78| ^{1}H-NMR spectrum (500 MHz, $CDCl_3$) for **L20**.

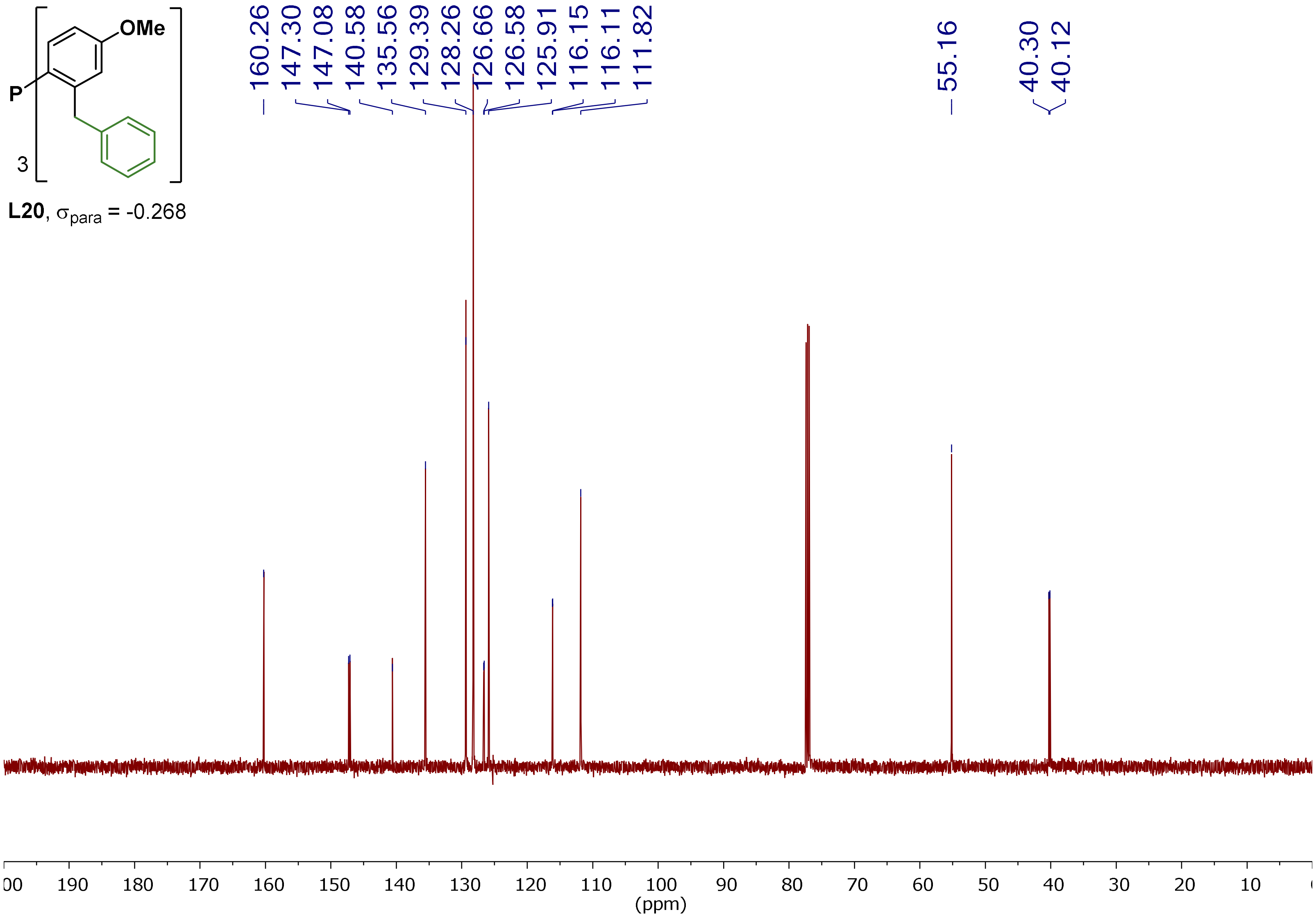

Supplementary Figure 79| ^{13}C-NMR spectrum (126 MHz, $CDCl_3$) for **L20**.

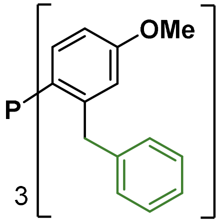

L20, σ_{para} = -0.268

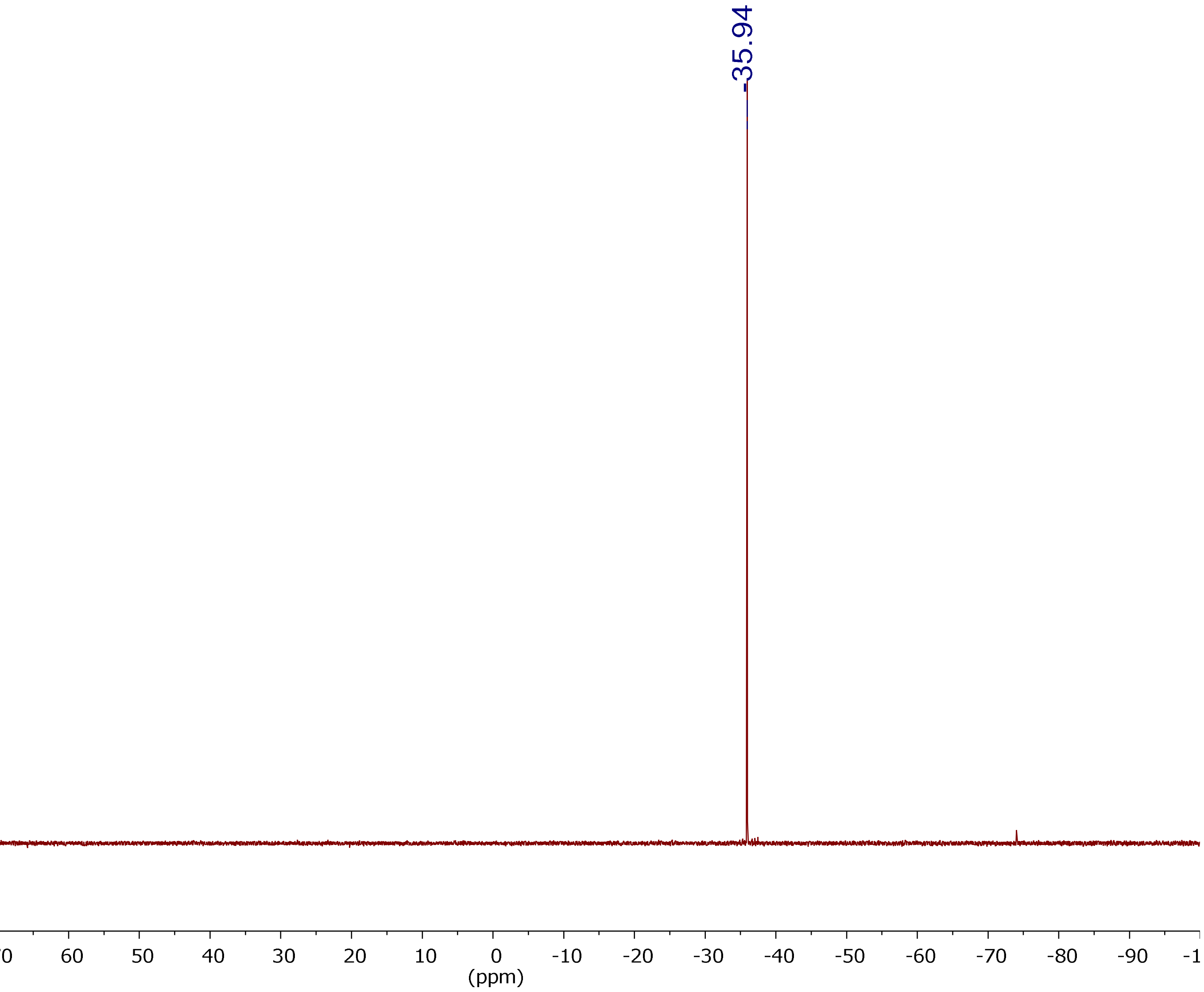

Supplementary Figure 80| ^{1}H-NMR spectrum (500 MHz, $CDCl_3$) for **L20**.

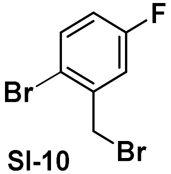

SI-10

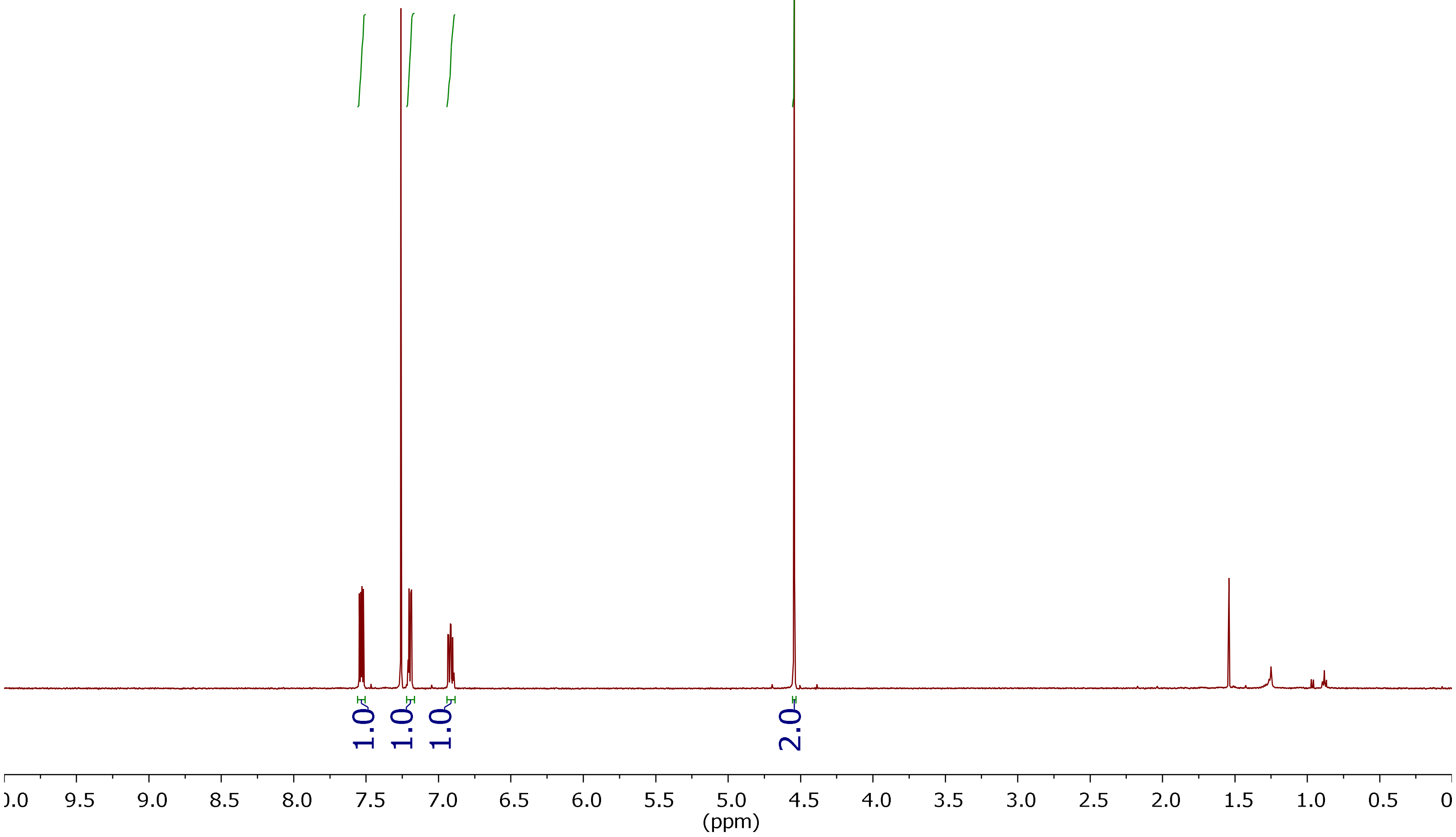

Supplementary Figure 81| ^{1}H-NMR spectrum (500 MHz, $CDCl_3$) for **SI-10**.

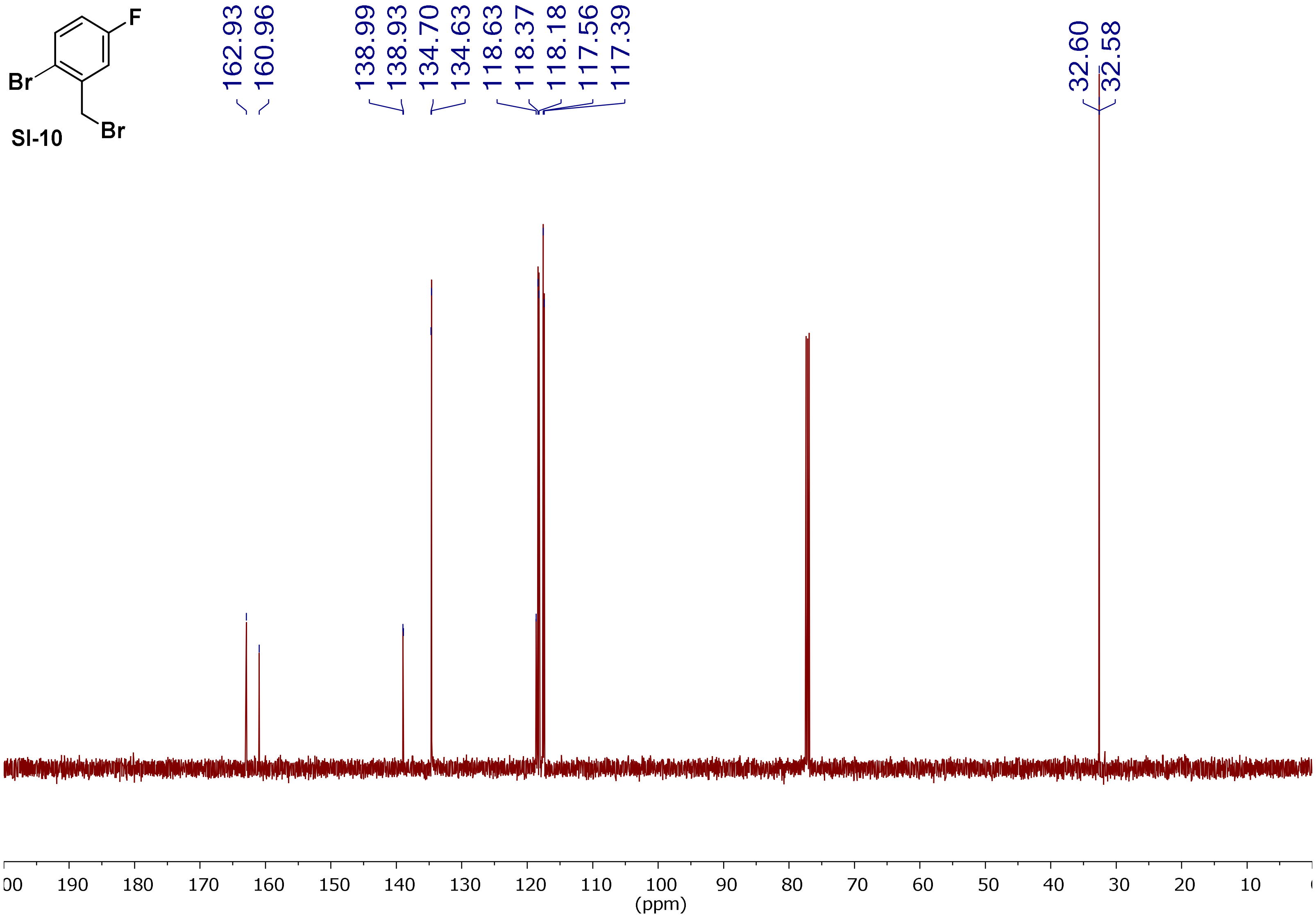

Supplementary Figure 82| ^{13}H-NMR spectrum (126 MHz, $CDCl_3$) for **SI-10**.

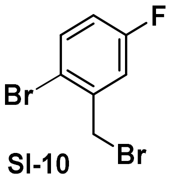
SI-10

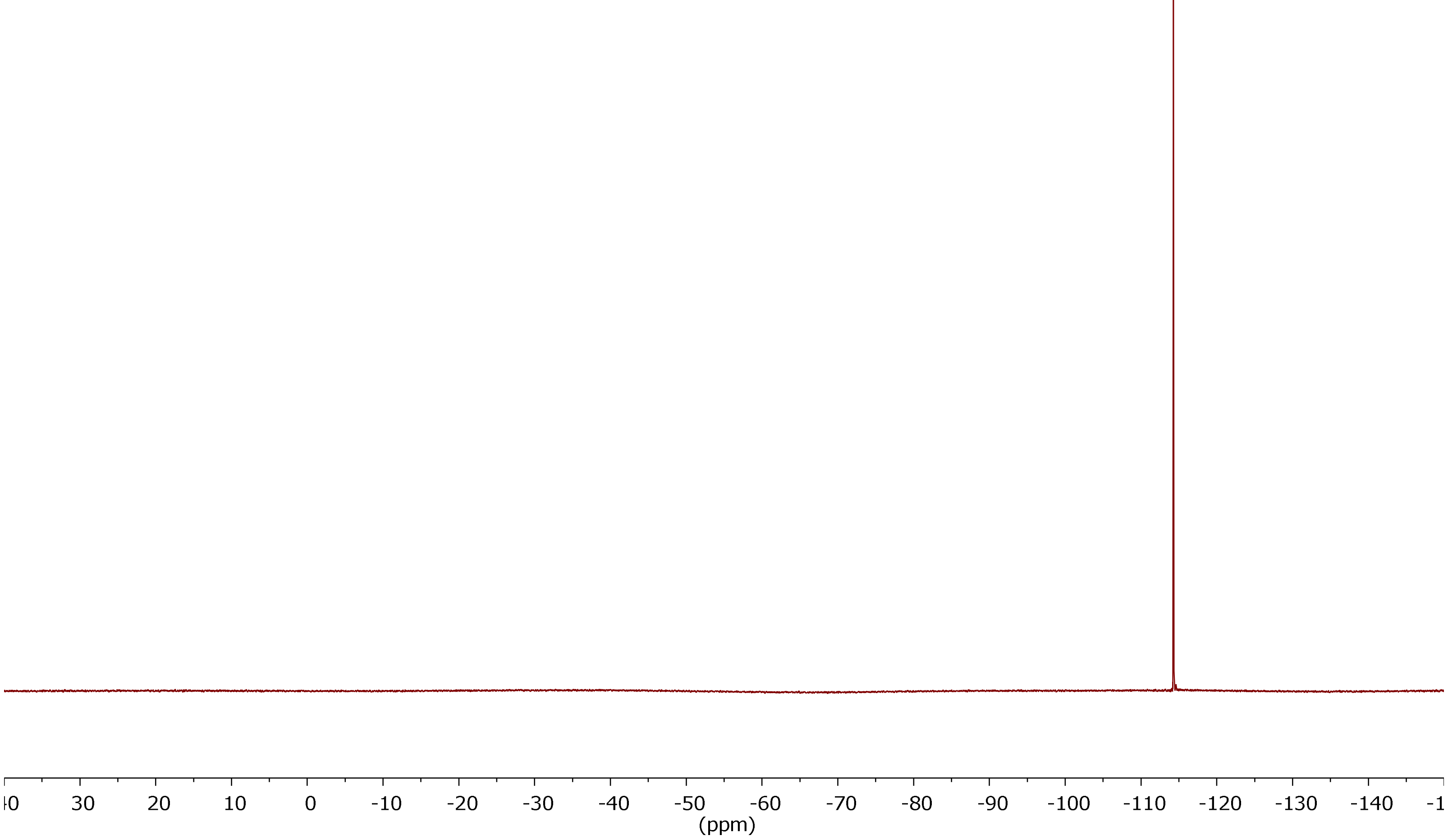

Supplementary Figure 83| ^{19}F-NMR spectrum (470 MHz, $CDCl_3$) for **SI-10**.

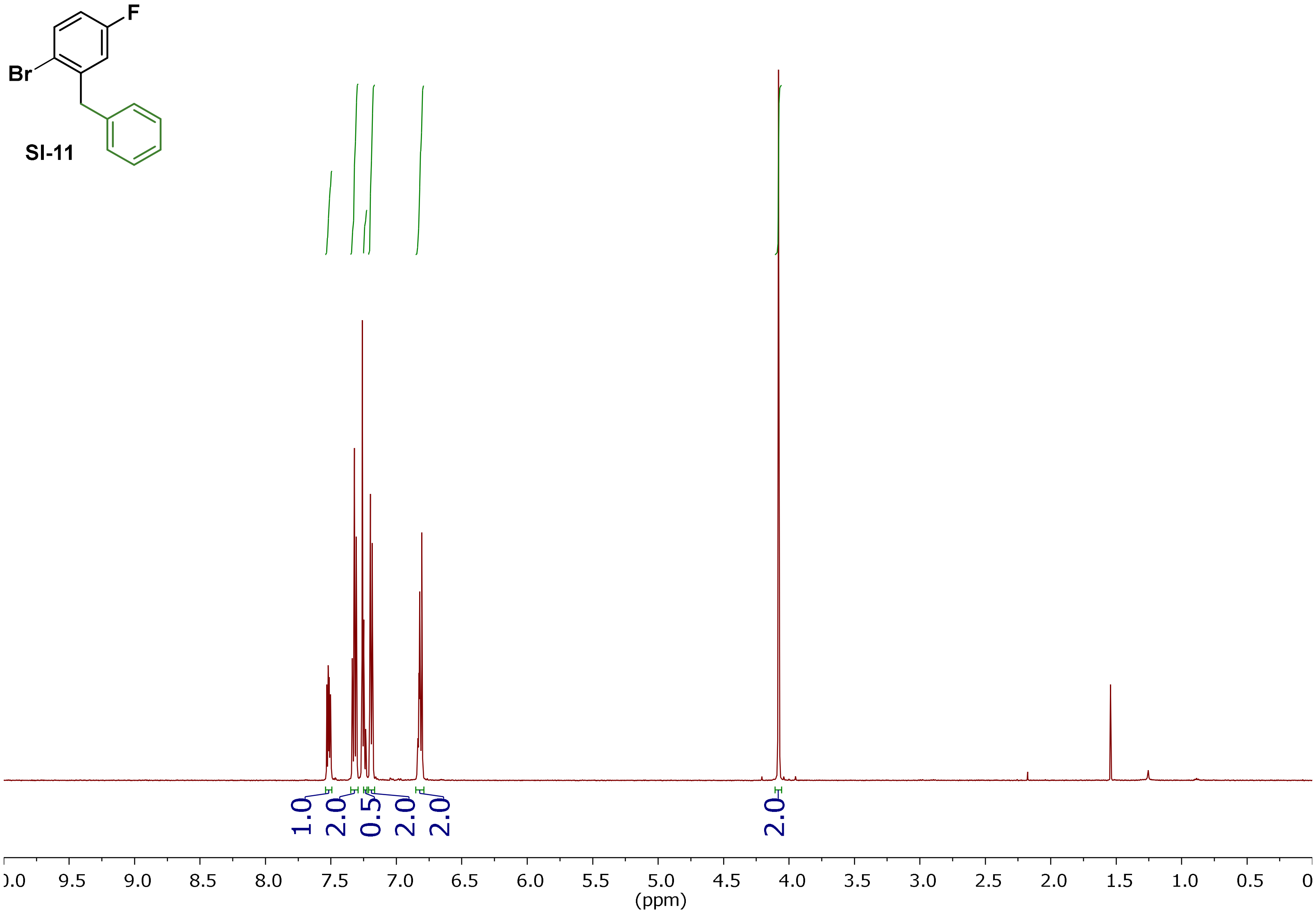

Supplementary Figure 84| ^{1}H-NMR spectrum (500 MHz, $CDCl_3$) for **SI-11**.

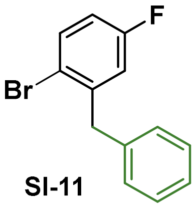

SI-11

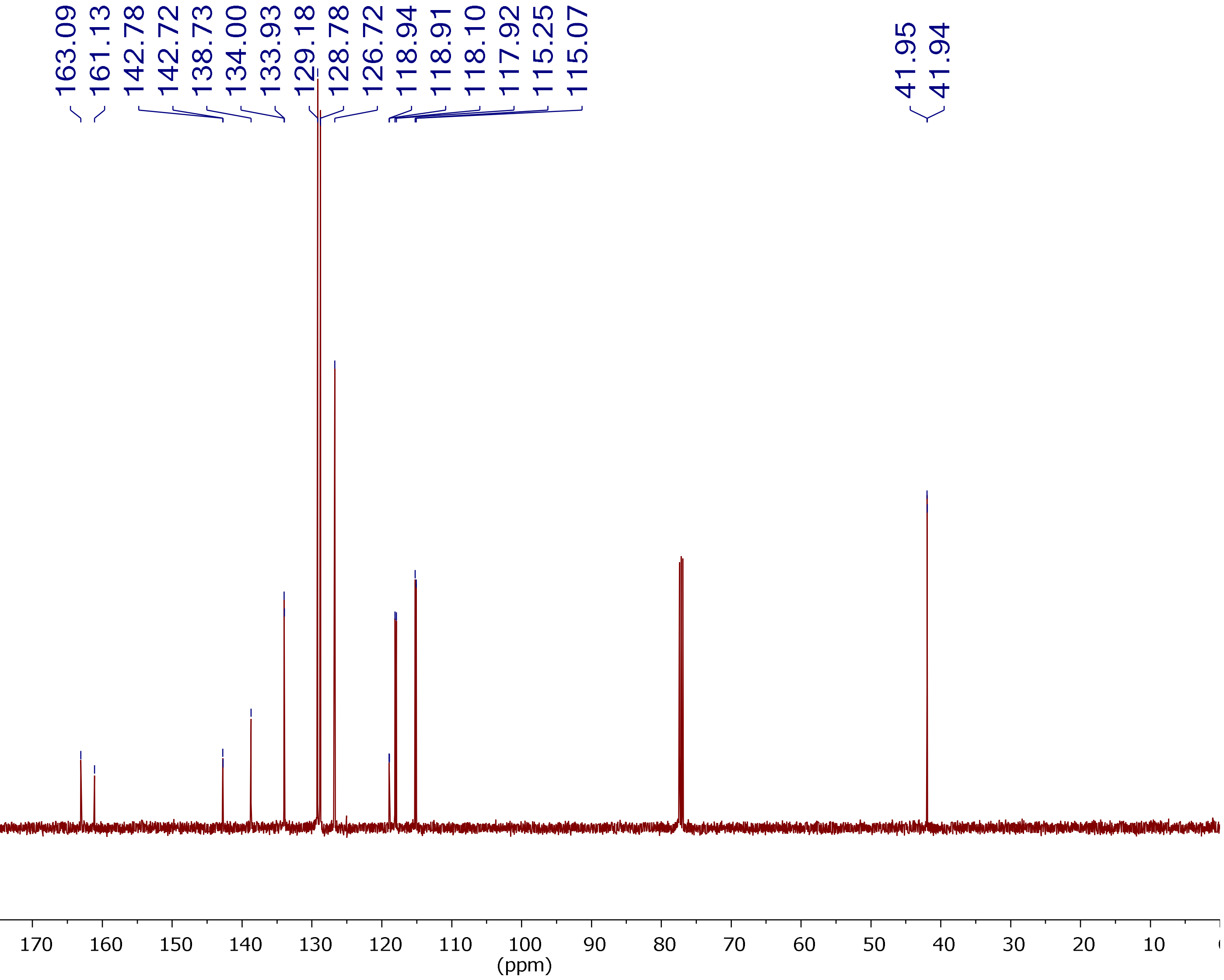

Supplementary Figure 85| ^{13}C-NMR spectrum (126 MHz, $CDCl_3$) for **SI-11**.

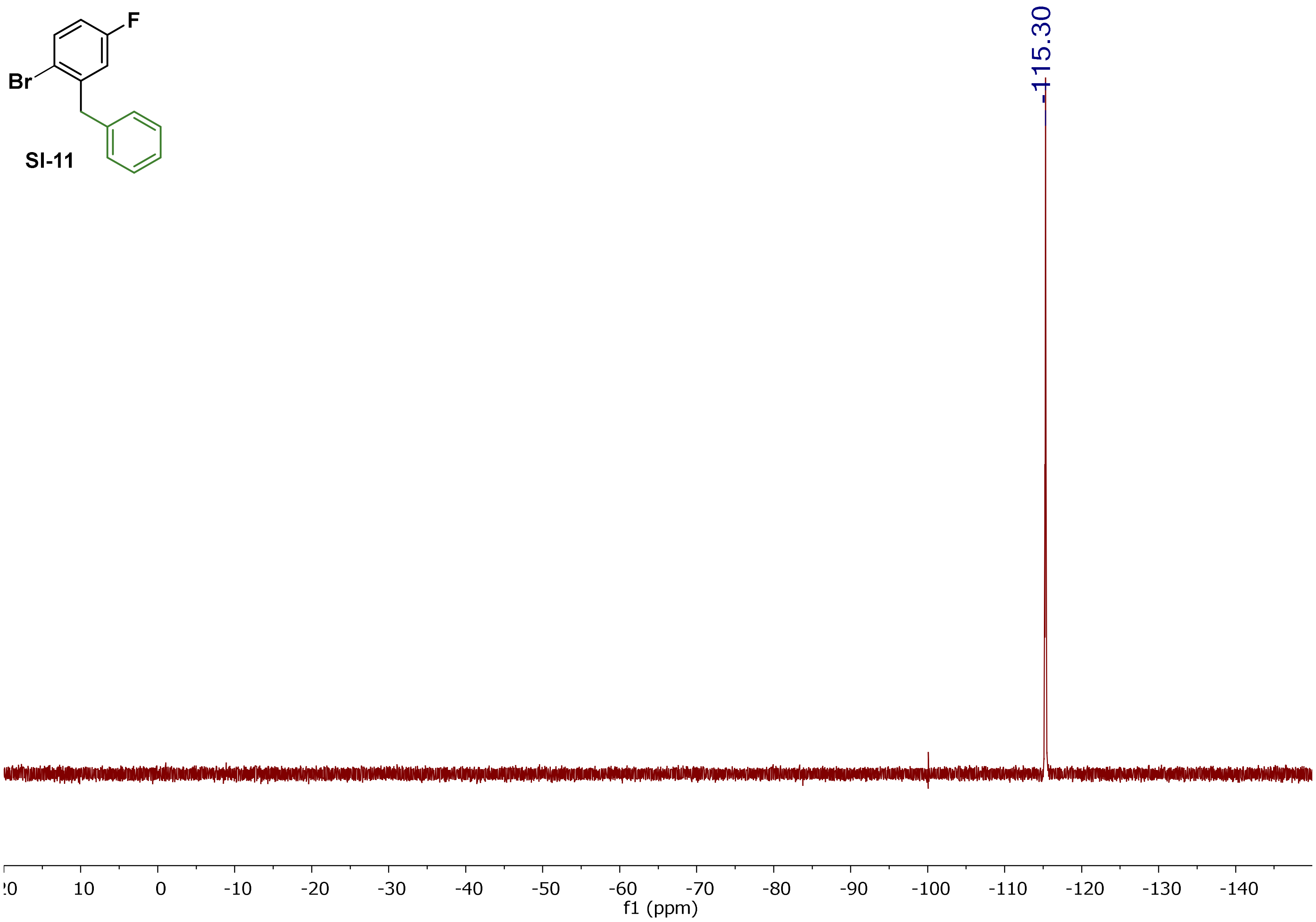

Supplementary Figure 86| ^{19}F-NMR spectrum (470 MHz, $CDCl_3$) for **SI-11**.

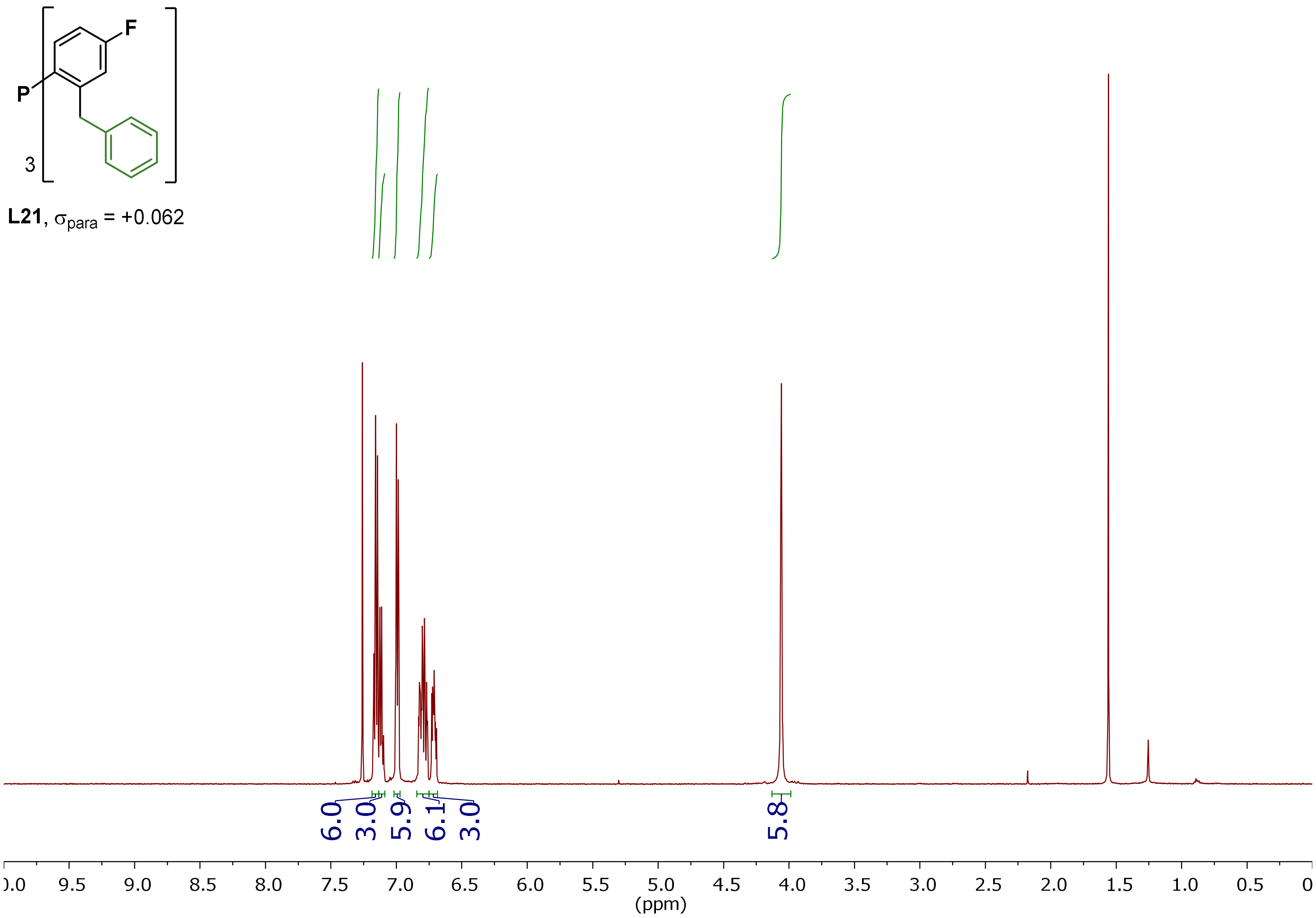

Supplementary Figure 87| ^{1}H-NMR spectrum (500 MHz, $CDCl_3$) for **L21**.

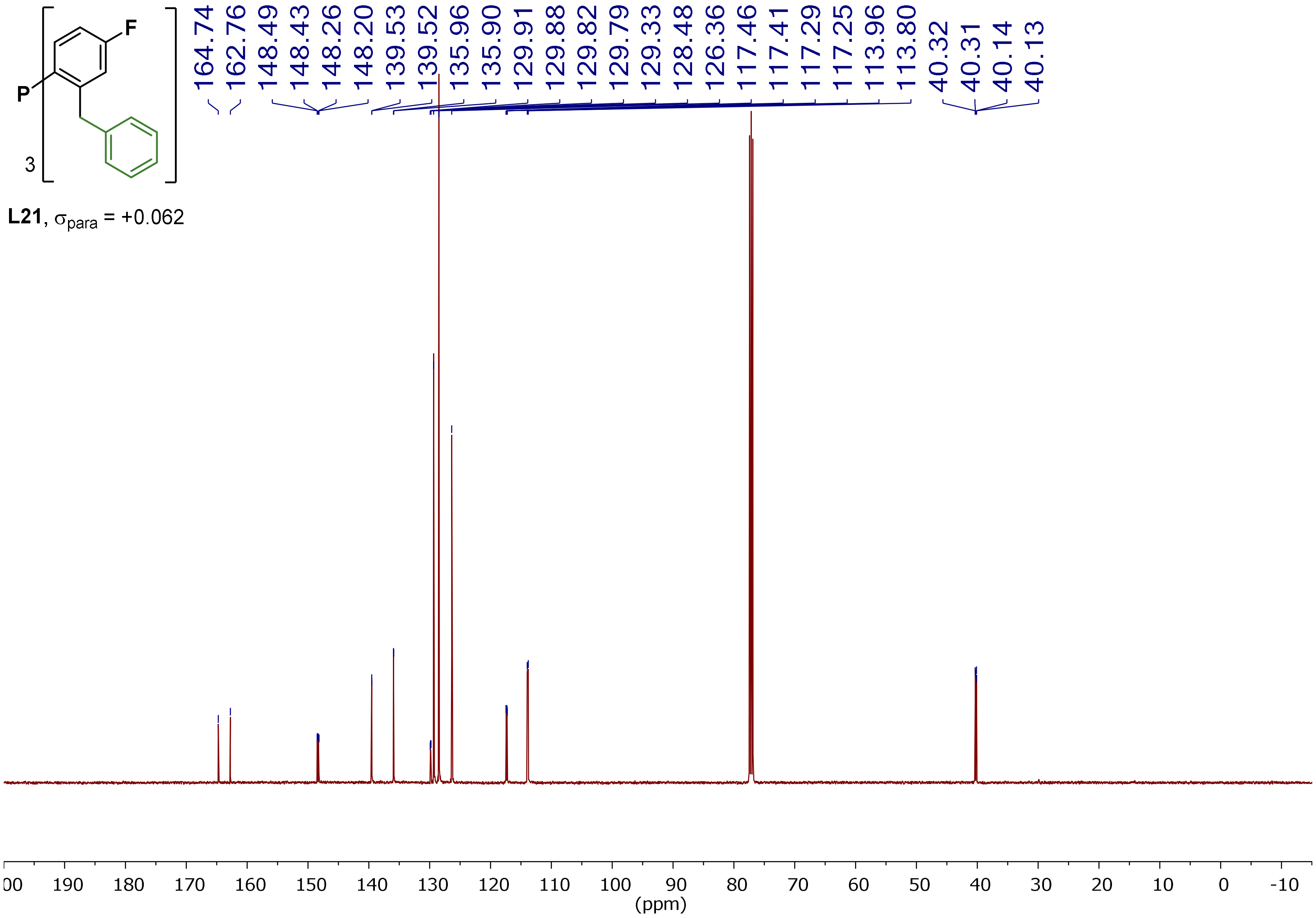

Supplementary Figure 88| ^{13}C-NMR spectrum (126 MHz, $CDCl_3$) for **L21**.

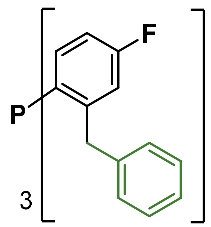

L21, σ_{para} = +0.062

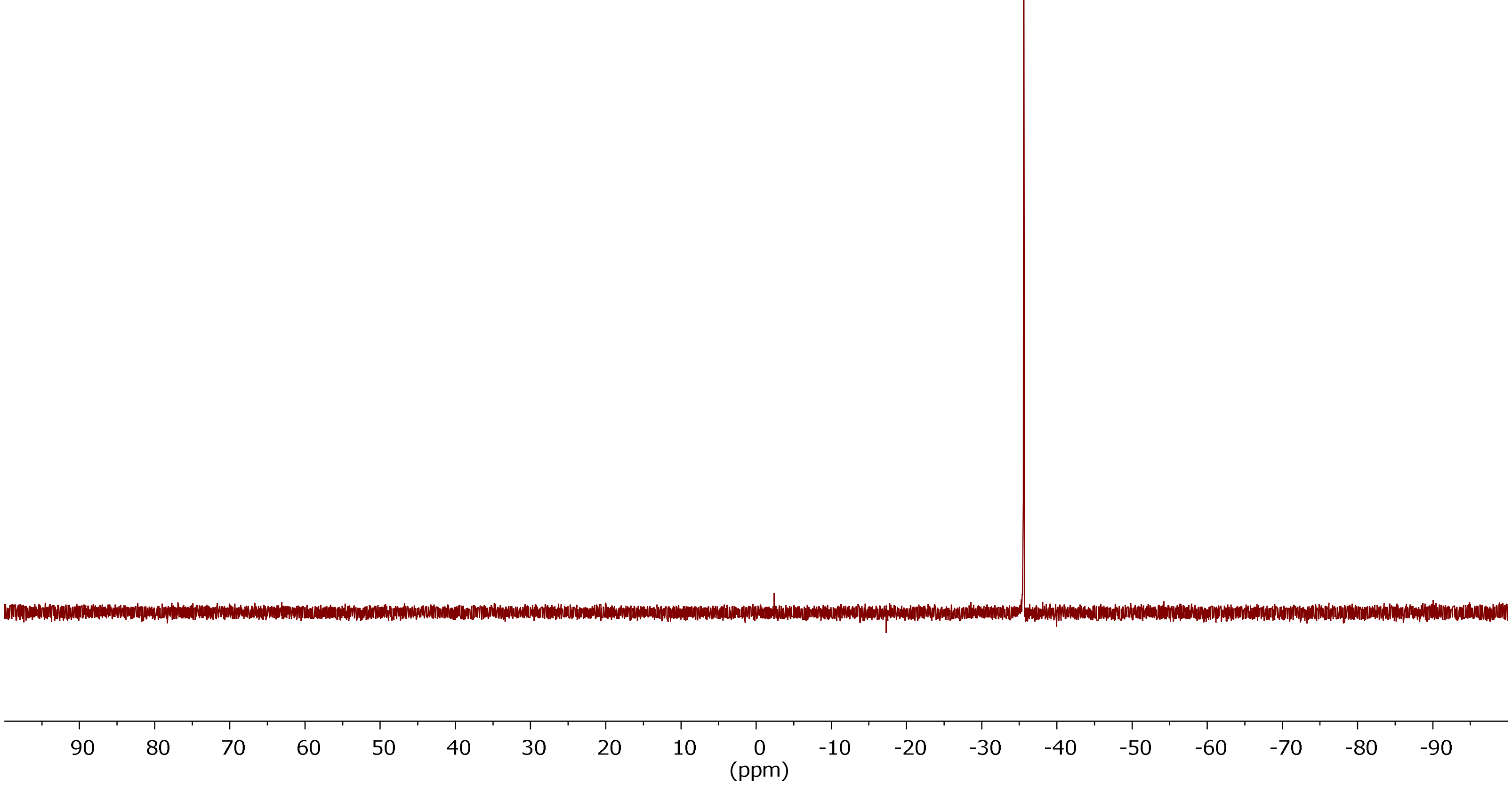

Supplementary Figure 89| ^{31}P-NMR spectrum (202 MHz, $CDCl_3$) for **L21**.

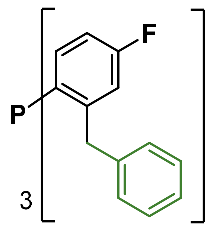

L21, σ_{para} = +0.062

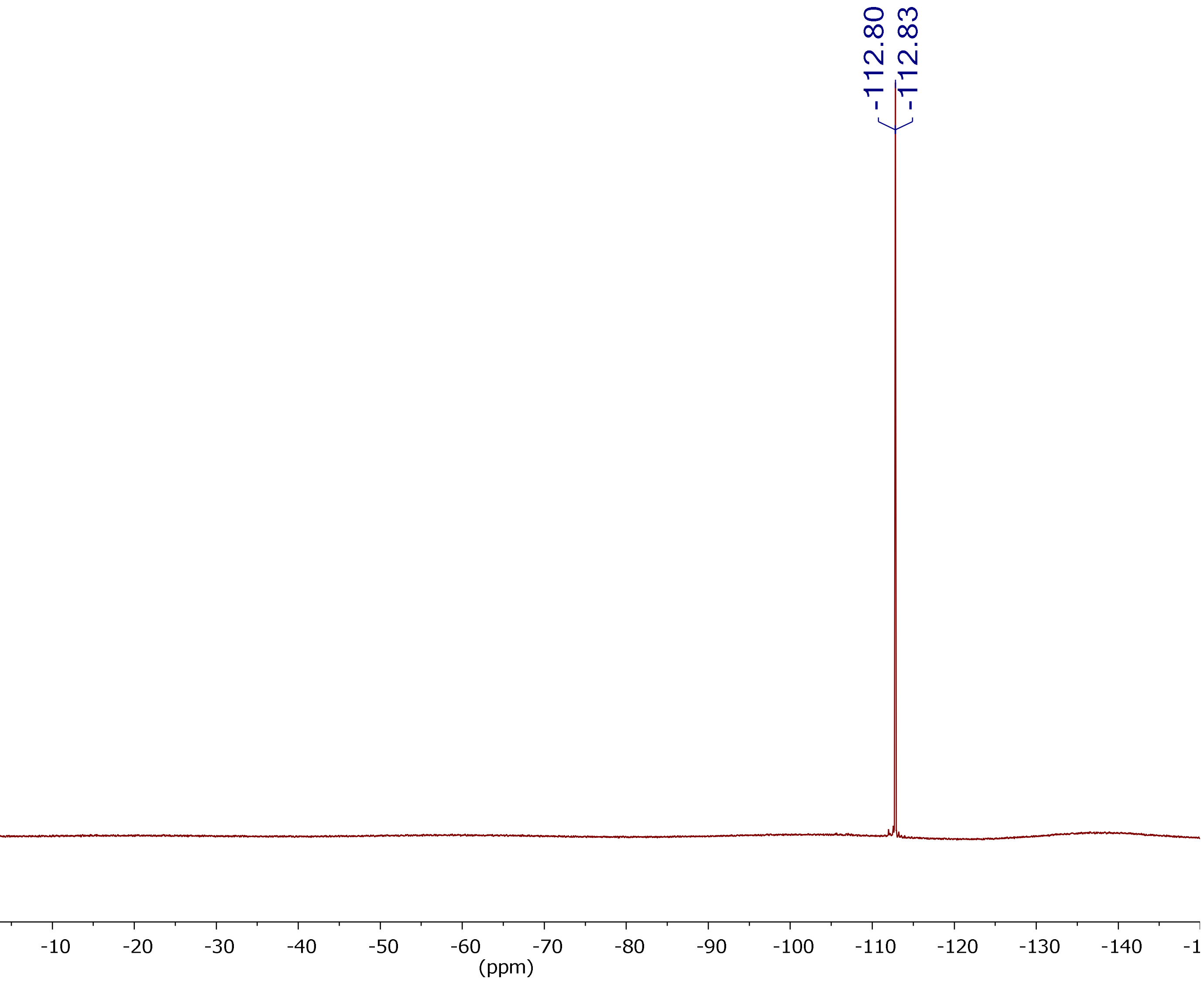

Supplementary Figure 90| ^{19}F-NMR spectrum (470 MHz, $CDCl_3$) for **L21**.

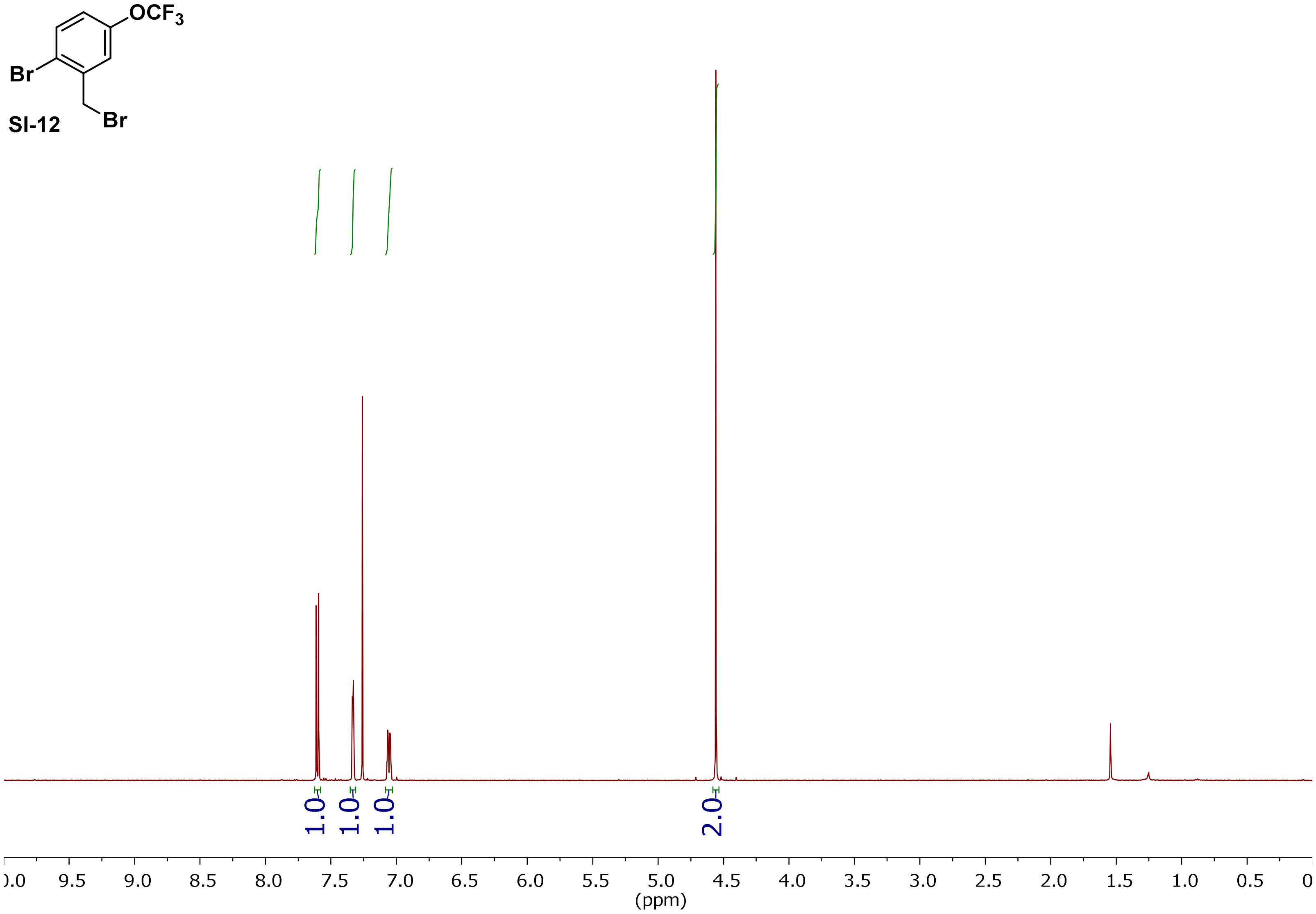

Supplementary Figure 91| ^{1}H-NMR spectrum (500 MHz, $CDCl_3$) for **SI-12**.

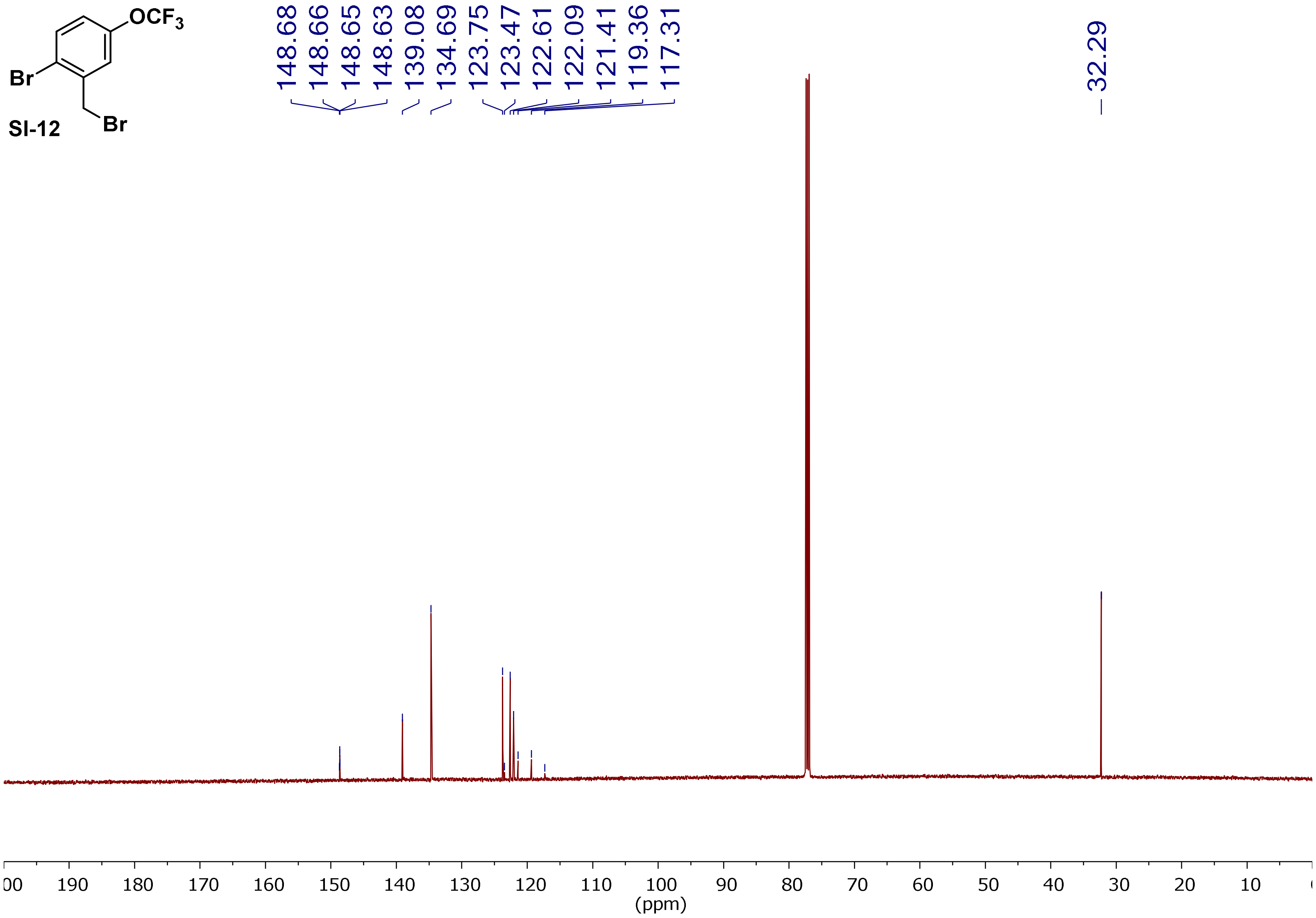

Supplementary Figure 92| ^{13}C-NMR spectrum (126 MHz, $CDCl_3$) for **SI-12**.

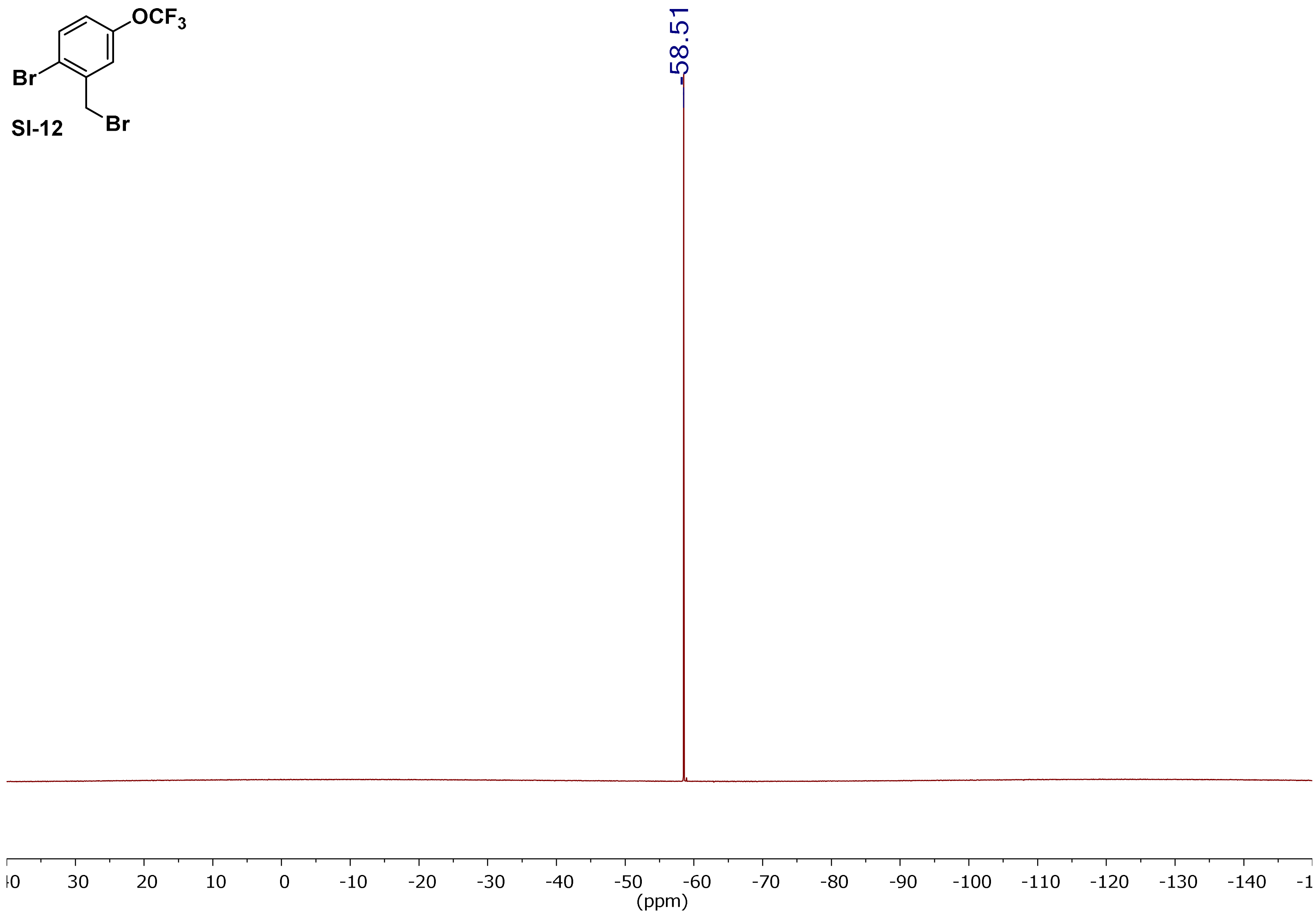

Supplementary Figure 93| ^{19}F-NMR spectrum (470 MHz, $CDCl_3$) for **SI-12**.

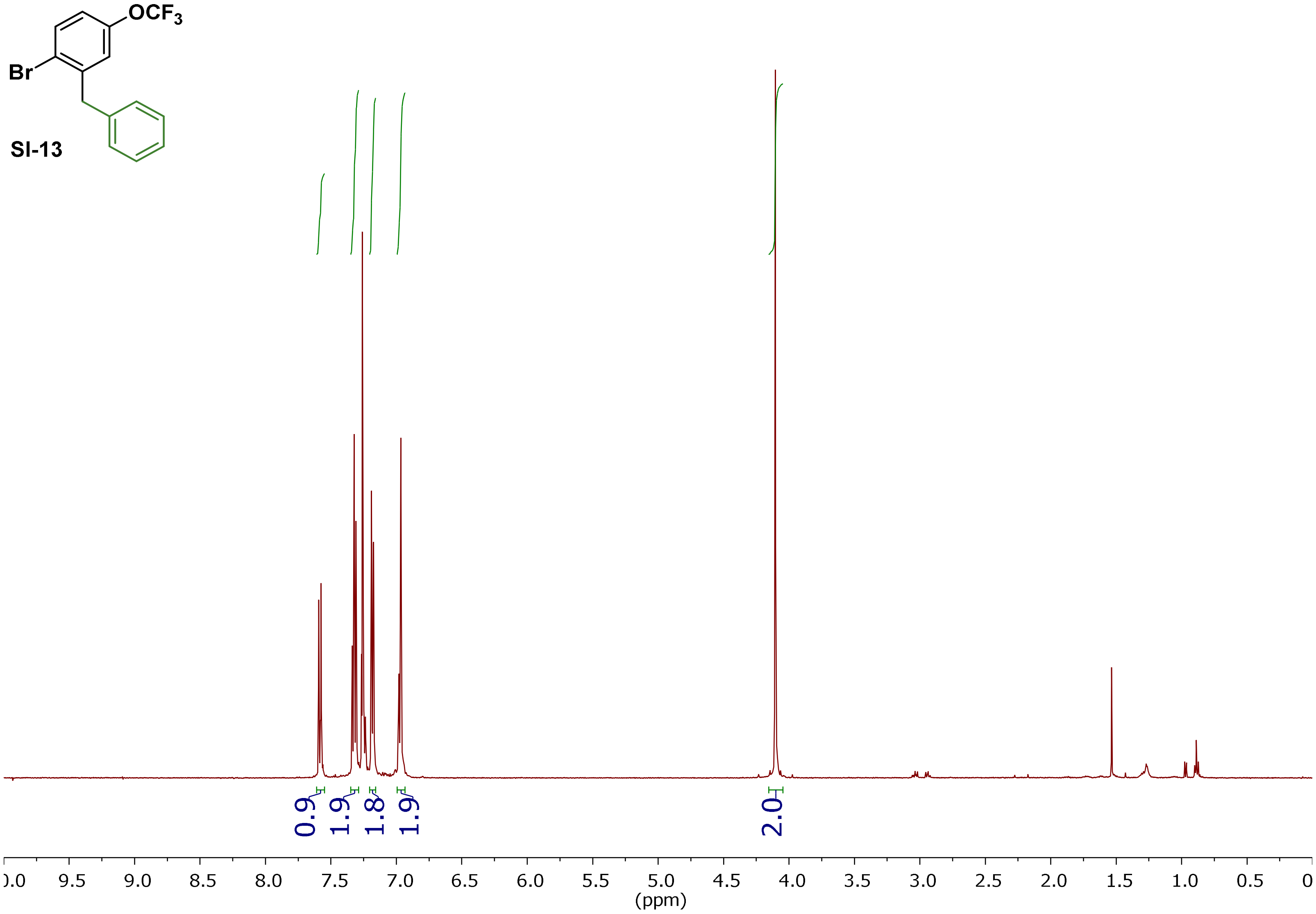

Supplementary Figure 94| ^{1}H-NMR spectrum (500 MHz, $CDCl_3$) for **SI-13**.

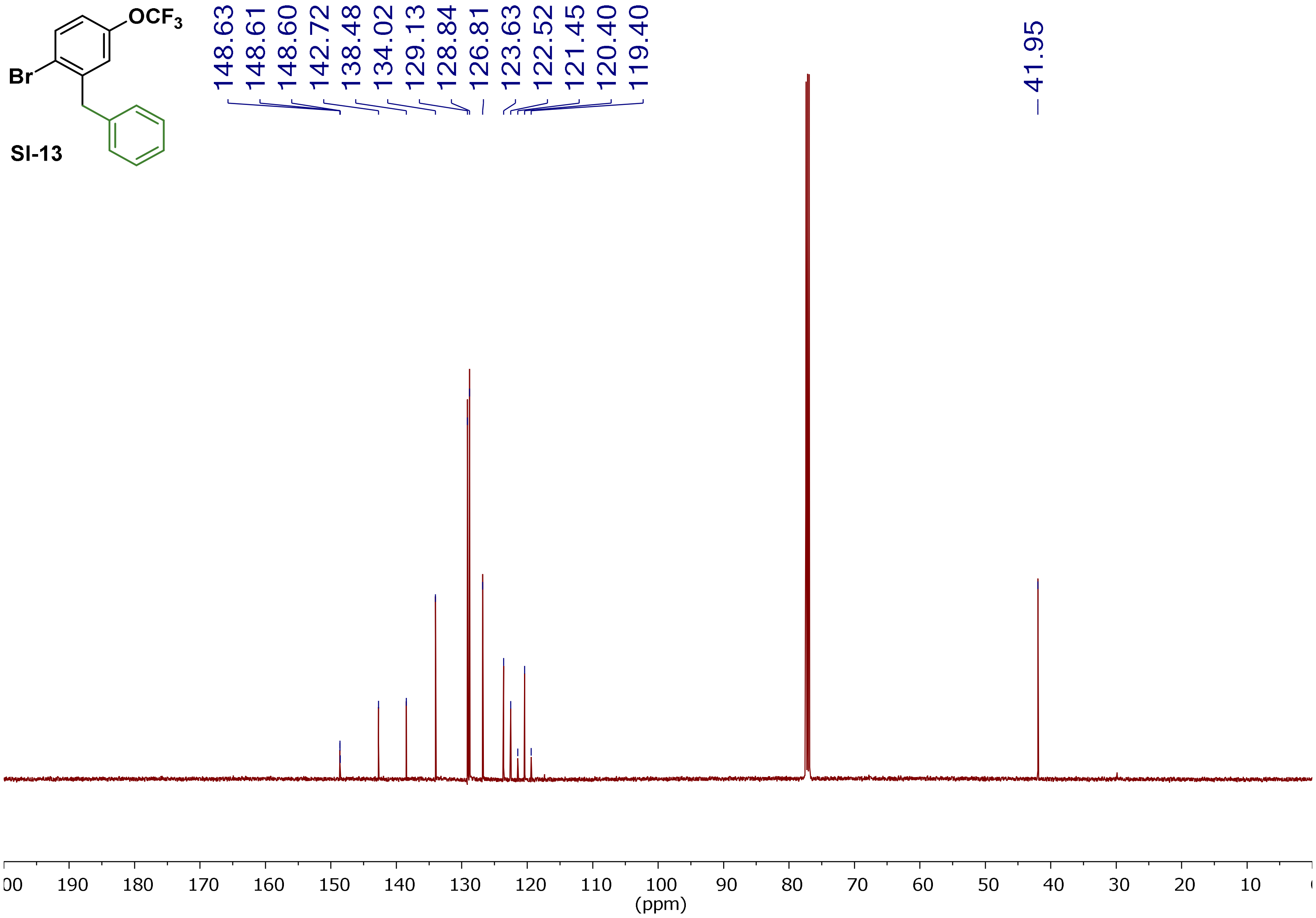

Supplementary Figure 95| ^{13}C-NMR spectrum (126 MHz, $CDCl_3$) for **SI-13**.

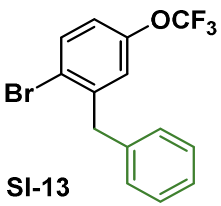
SI-13

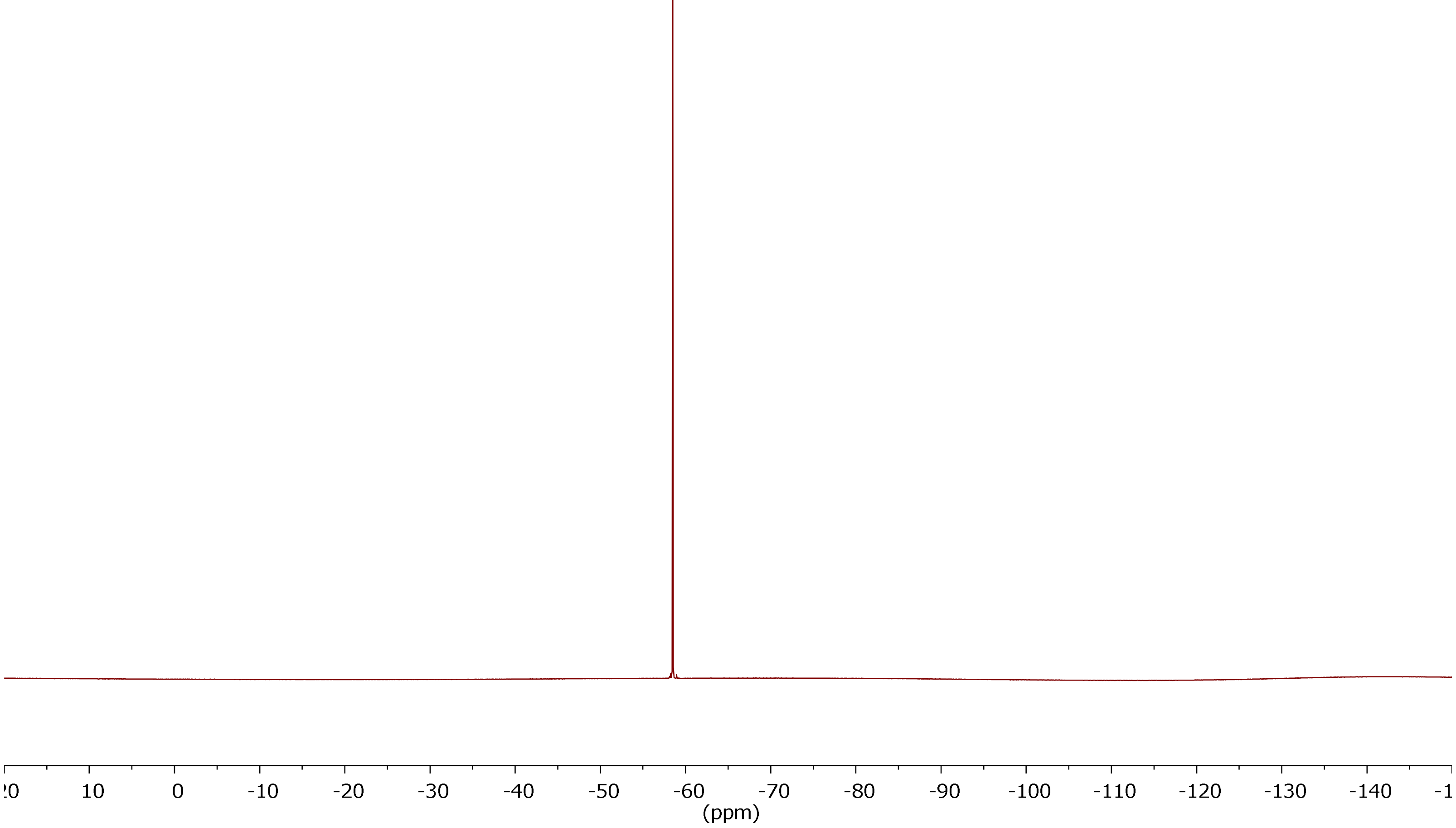

Supplementary Figure 96| ^{19}F-NMR spectrum (470 MHz, $CDCl_3$) for **SI-13**.

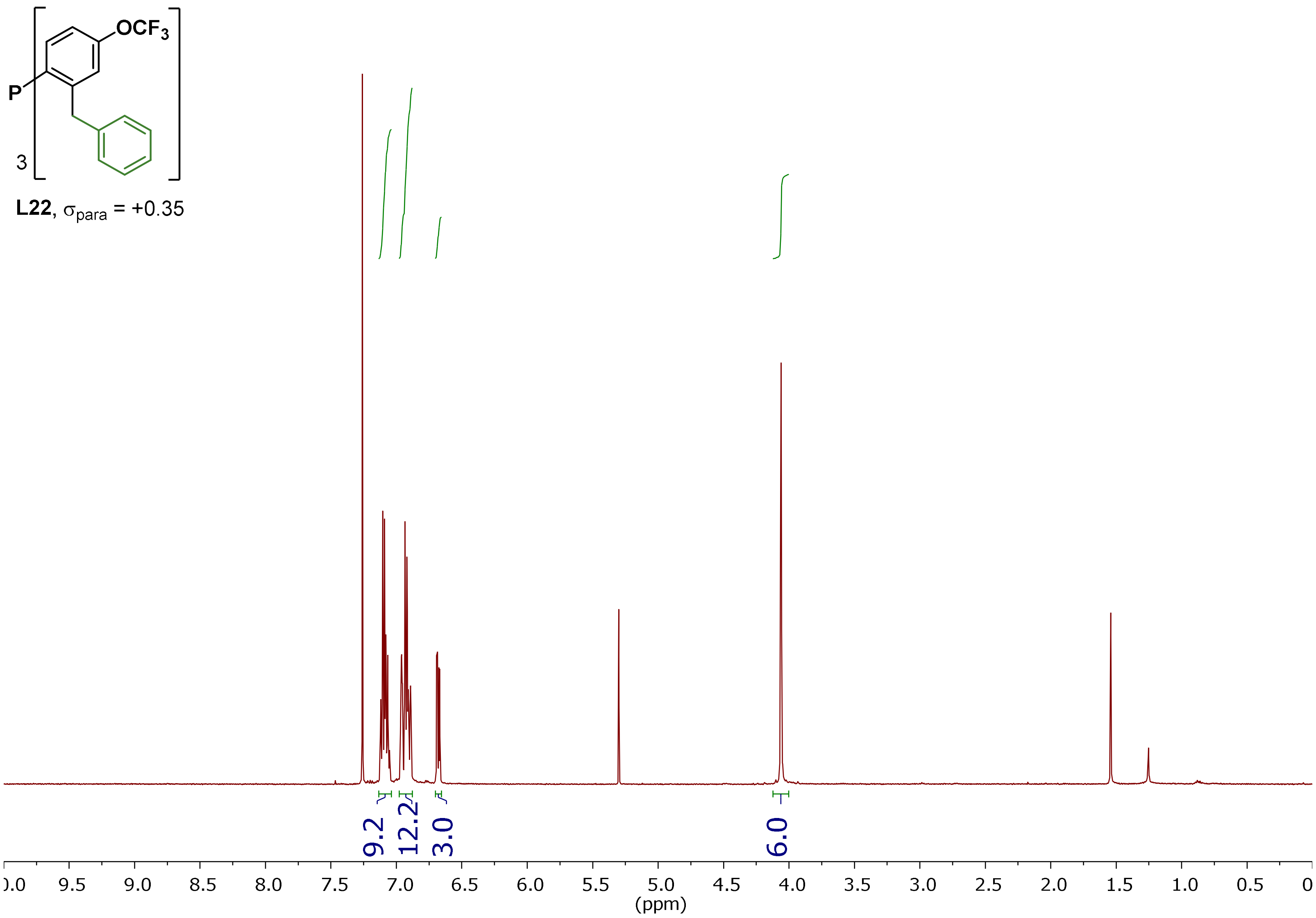

Supplementary Figure 97| ^{1}H-NMR spectrum (500 MHz, $CDCl_3$) for **L22**.

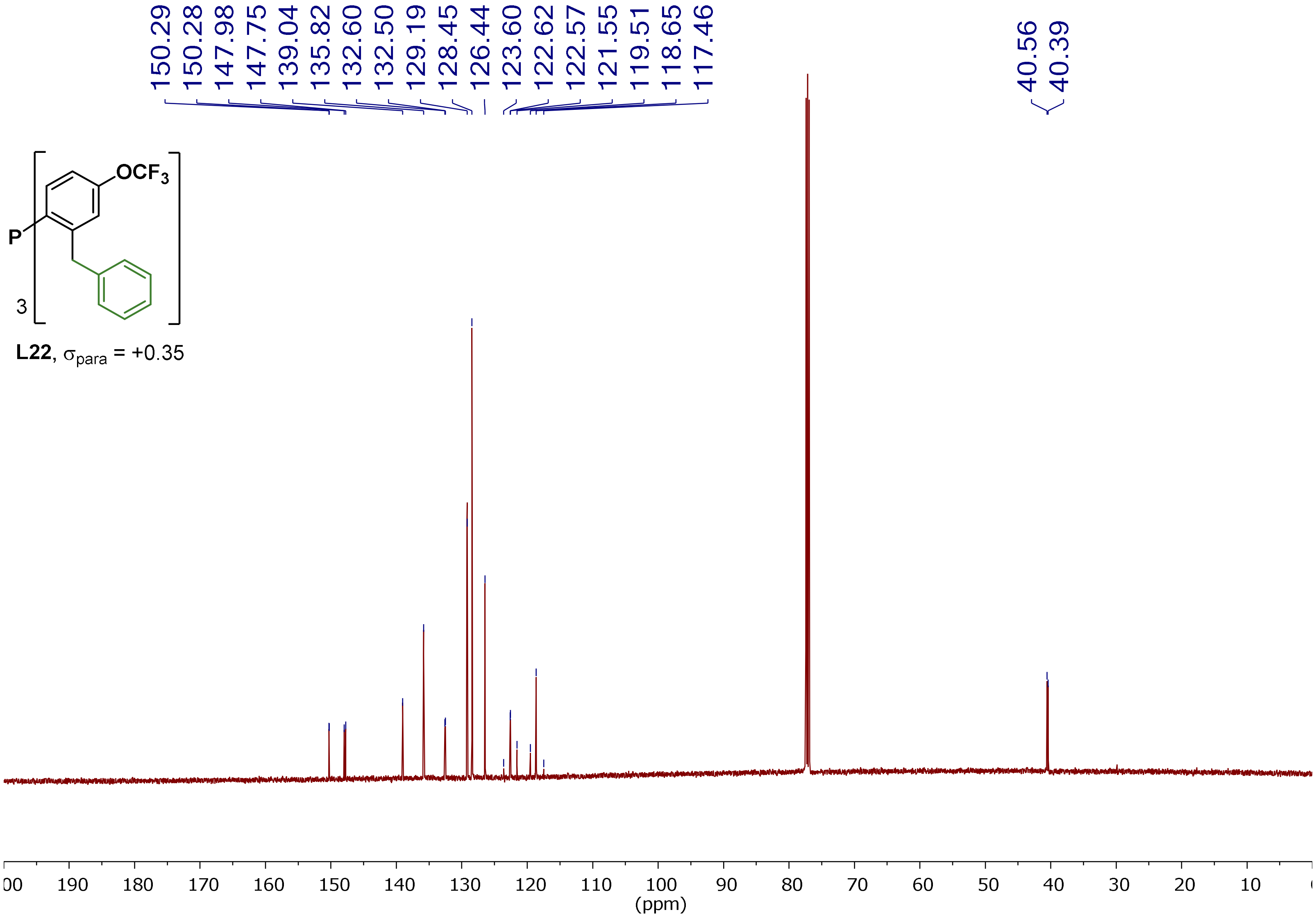

Supplementary Figure 98| ^{13}C-NMR spectrum (126 MHz, $CDCl_3$) for **L22**.

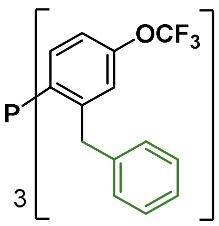

L22, σ_{para} = +0.35

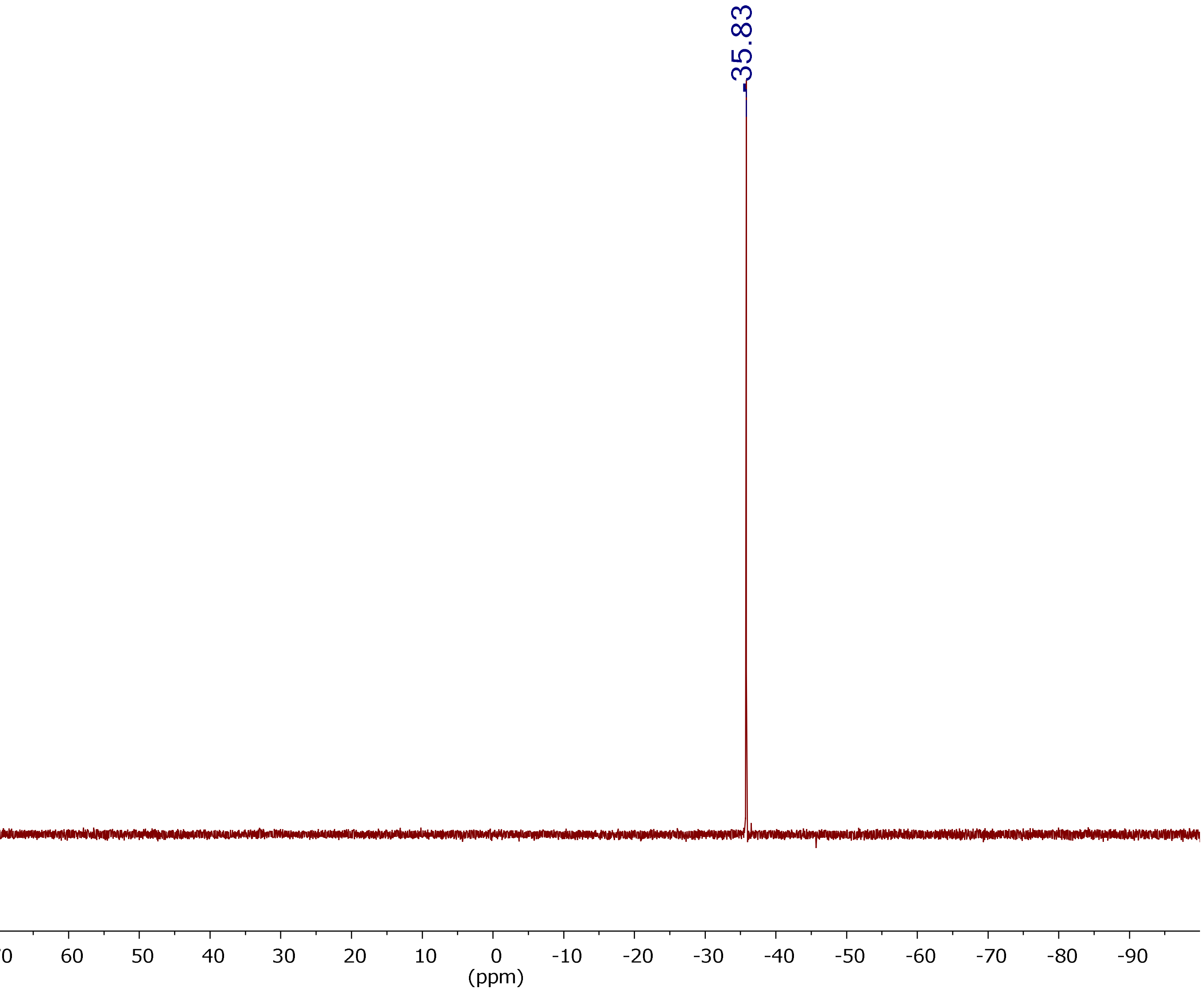

Supplementary Figure 99| ^{31}P-NMR spectrum (202 MHz, $CDCl_3$) for **L22**.

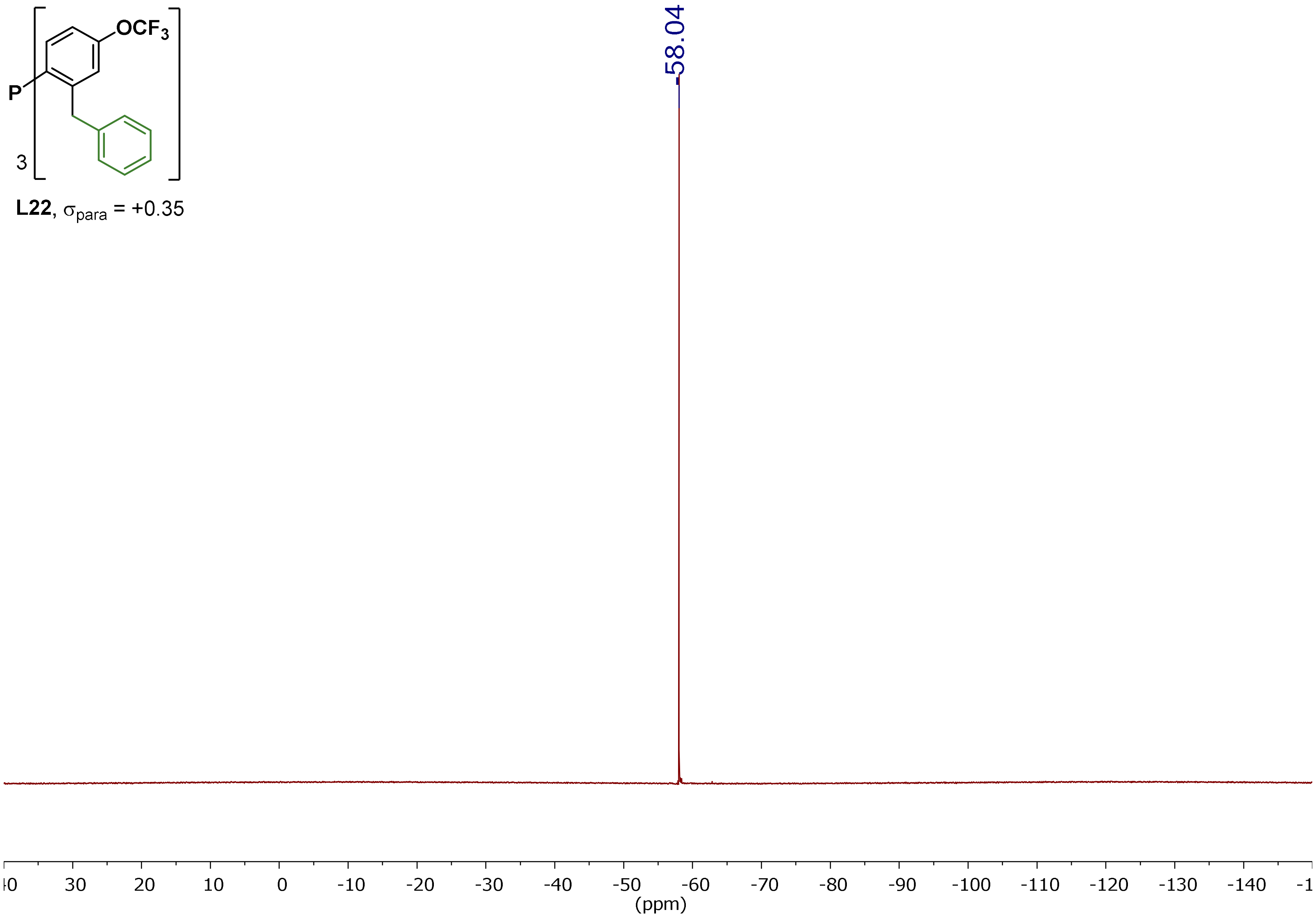

Supplementary Figure 100| ^{19}F-NMR spectrum (470 MHz, $CDCl_3$) for **L22**.

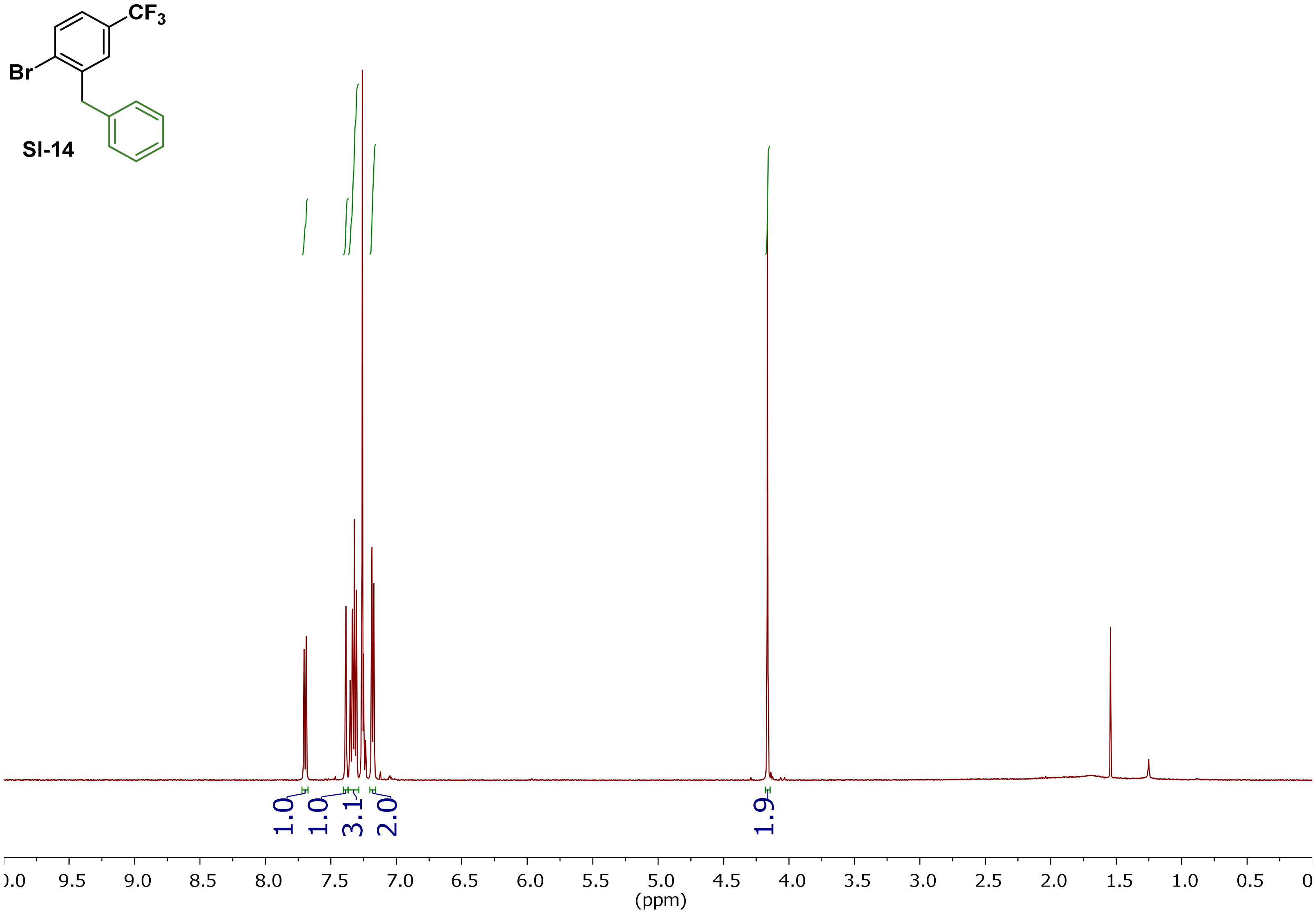

Supplementary Figure 101| ^{1}H-NMR spectrum (500 MHz, $CDCl_3$) for **SI-14**.

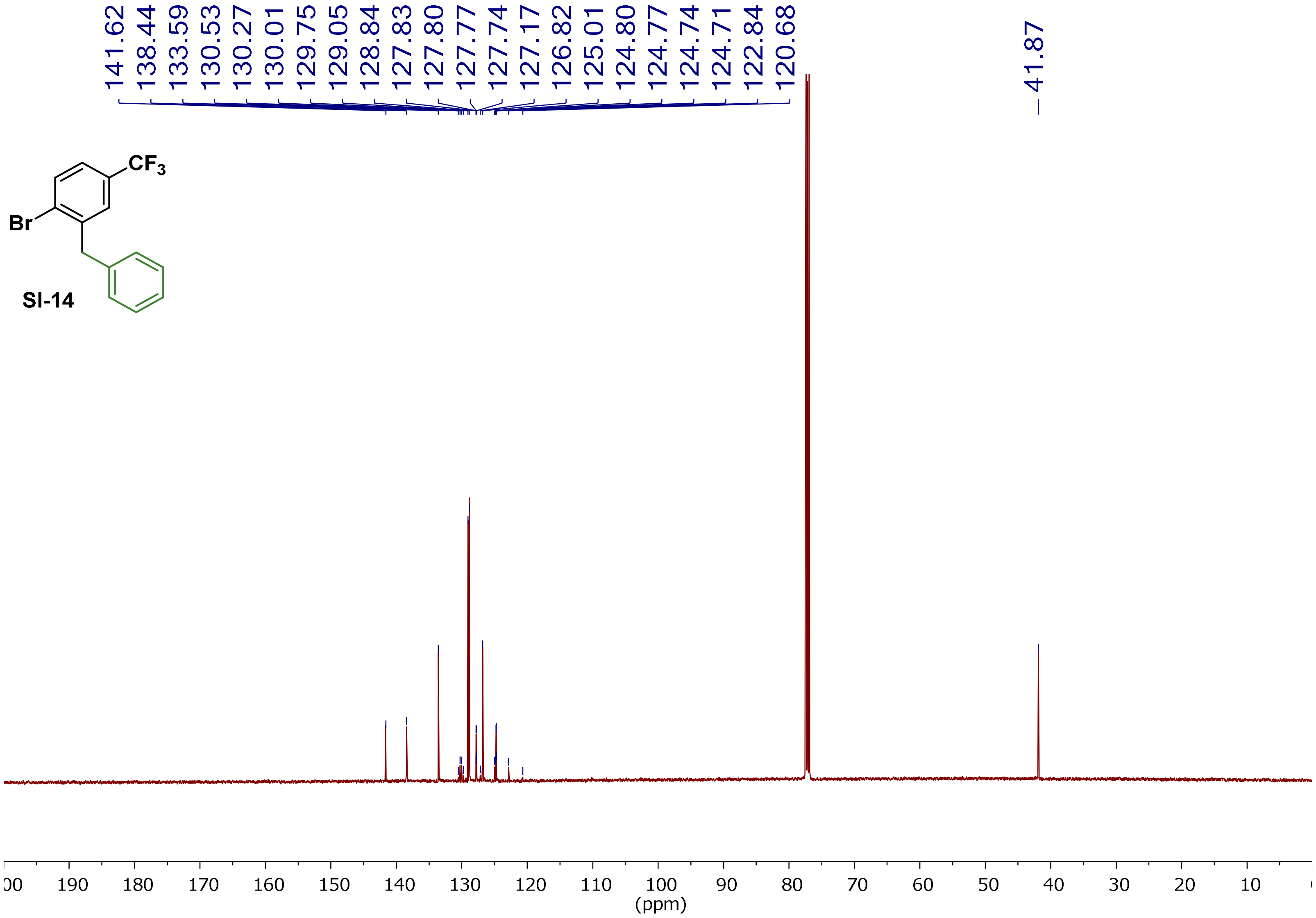

Supplementary Figure 102| ^{13}C-NMR spectrum (126 MHz, $CDCl_3$) for **SI-14**.

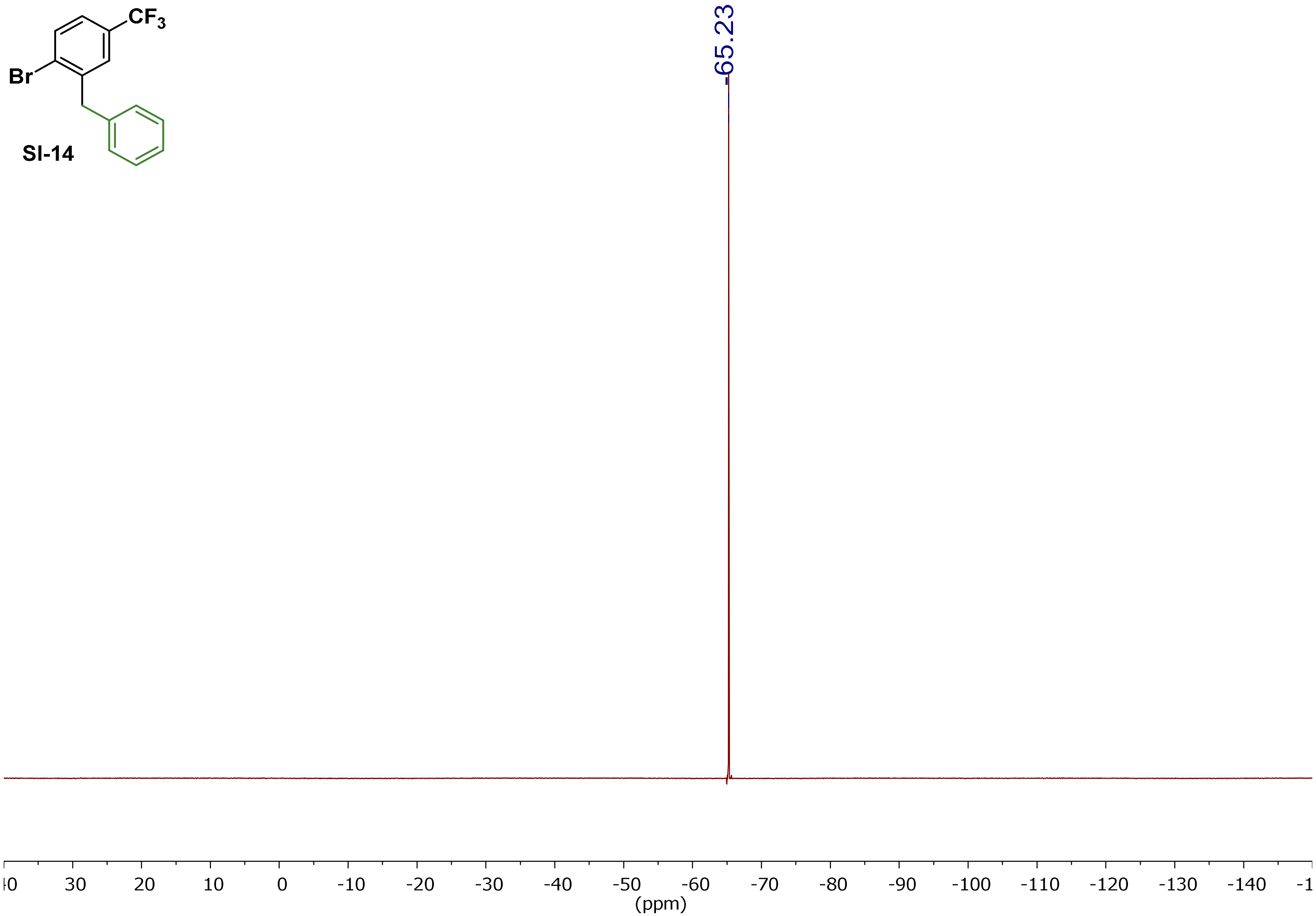

Supplementary Figure 103| ^{19}F-NMR spectrum (470 MHz, $CDCl_3$) for **SI-14**.

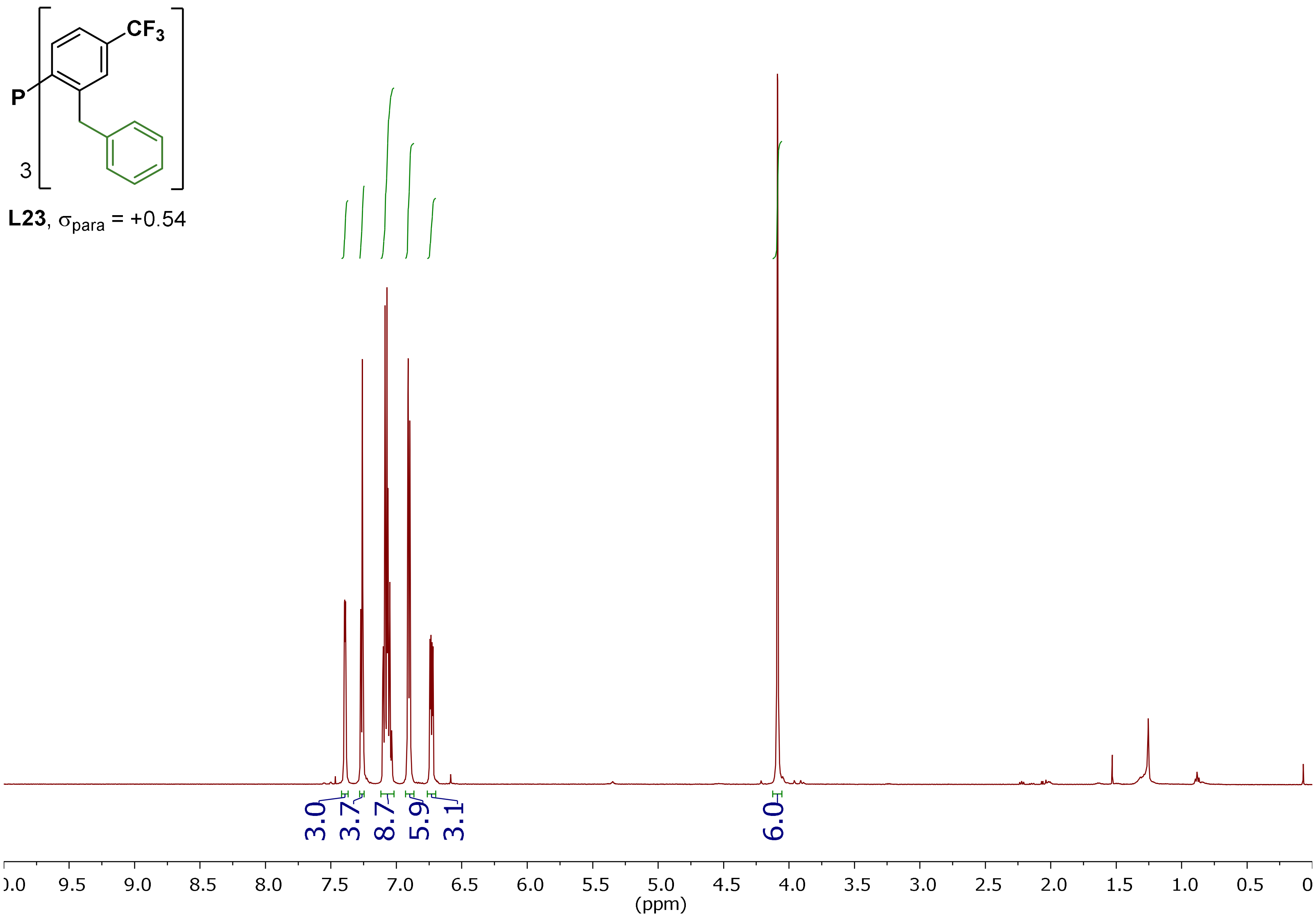

Supplementary Figure 104| ^{1}H-NMR spectrum (500 MHz, $CDCl_3$) for **L23**.

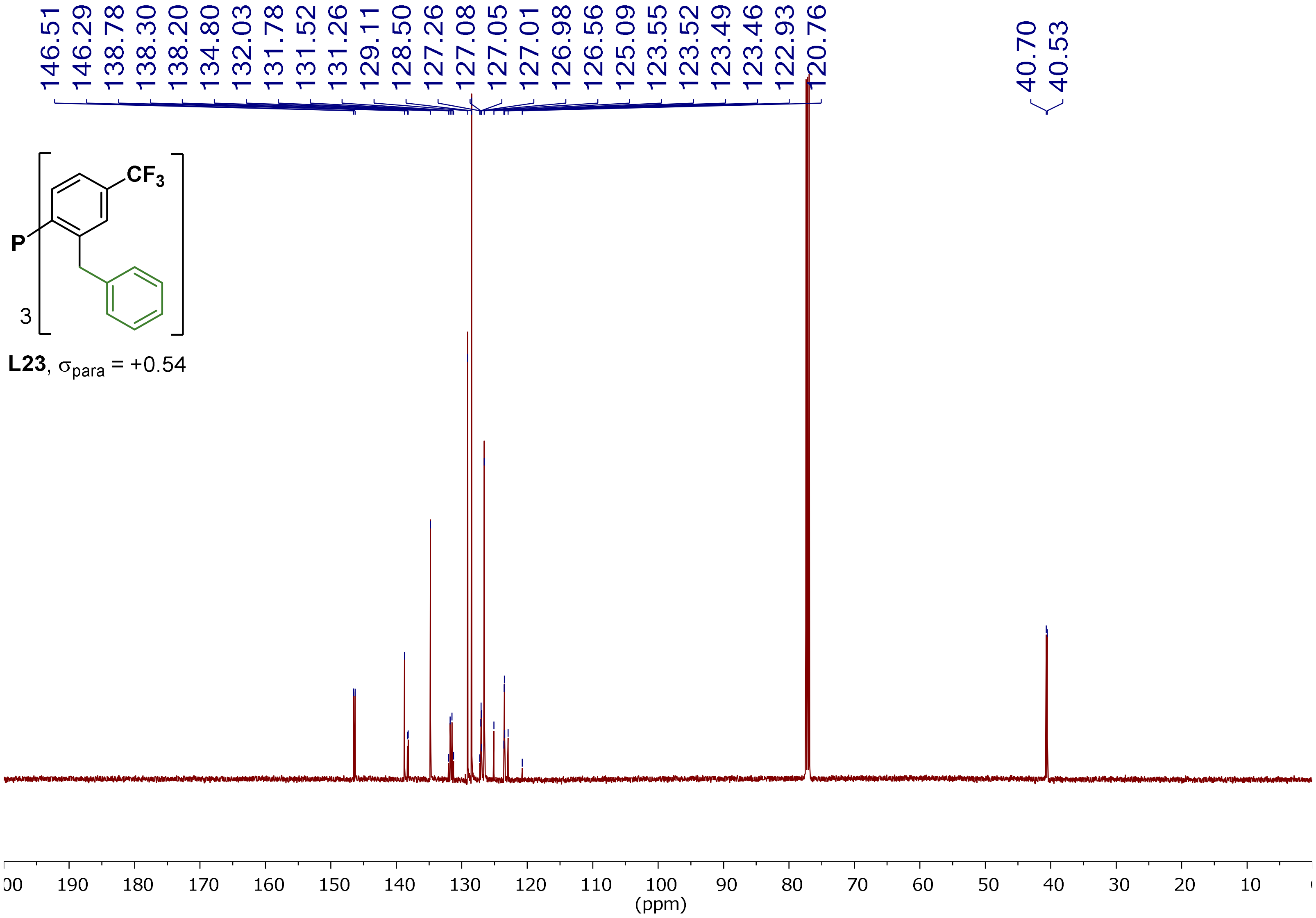

Supplementary Figure 105| ^{13}C-NMR spectrum (126 MHz, $CDCl_3$) for **L23**.

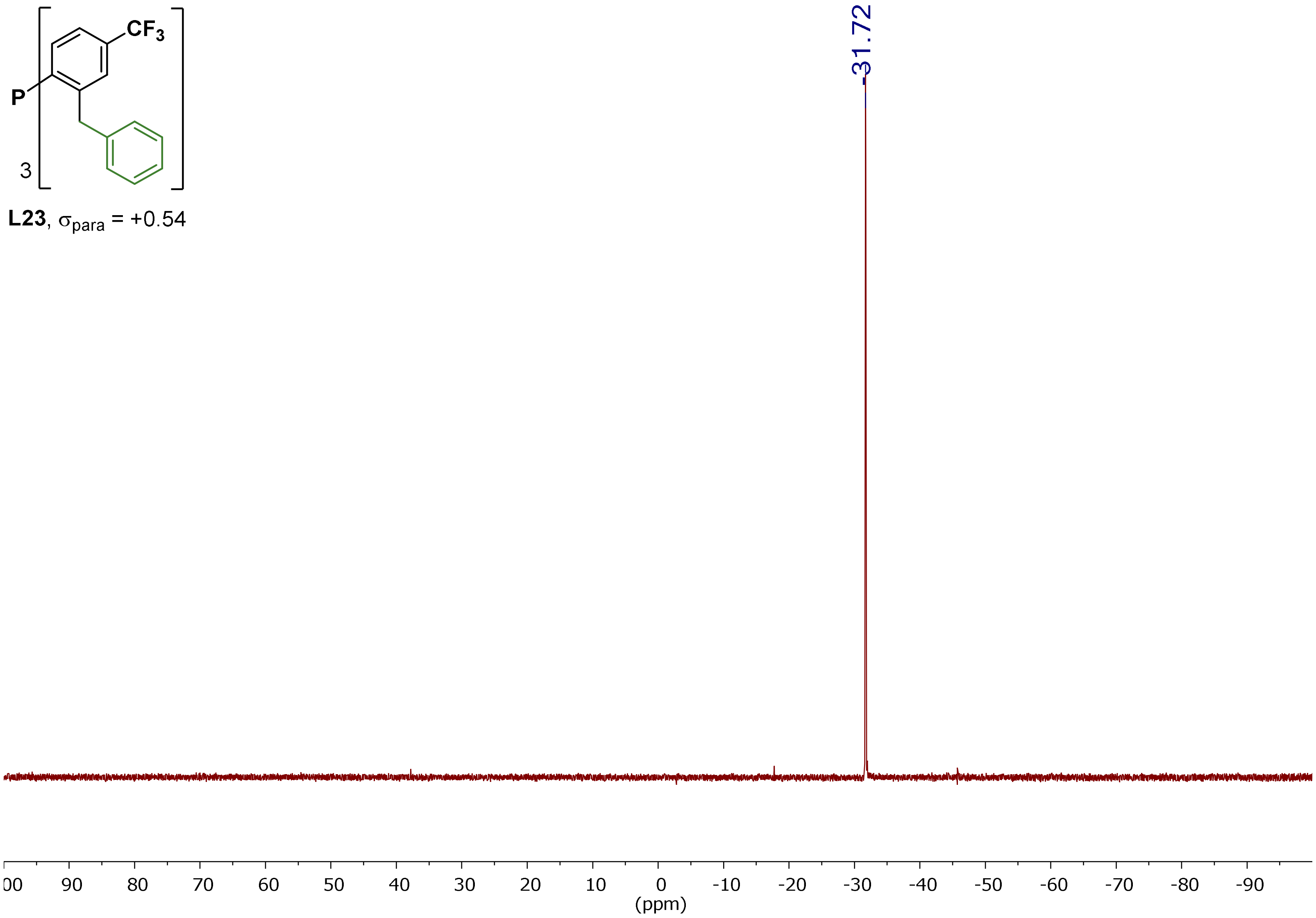

Supplementary Figure 106| ^{31}P-NMR spectrum (202 MHz, $CDCl_3$) for **L23**.

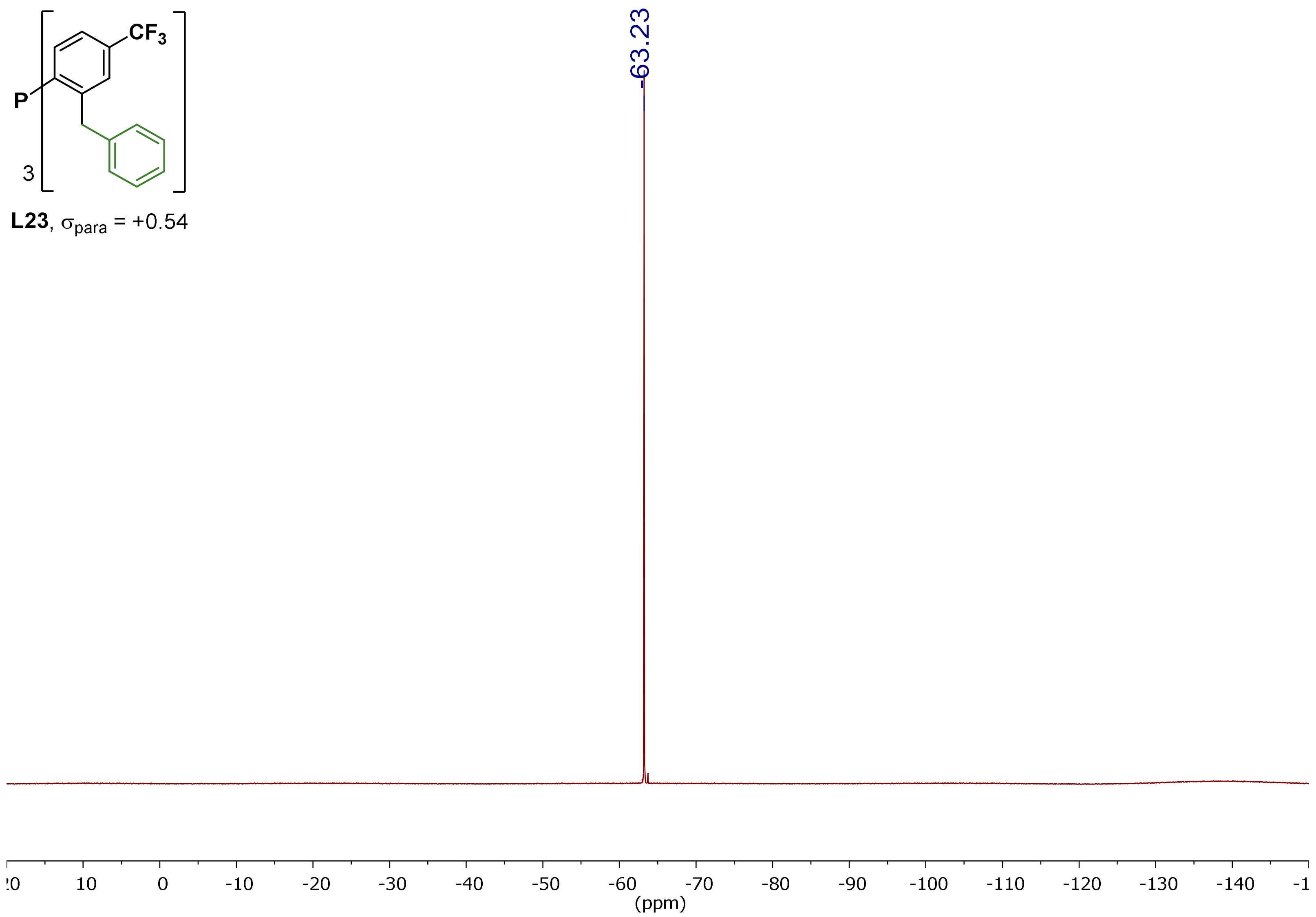

Supplementary Figure 107| ^{19}F-NMR spectrum (470 MHz, $CDCl_3$) for **L23**.

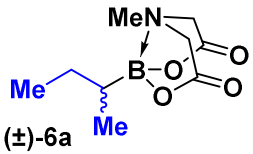

(±)-6a

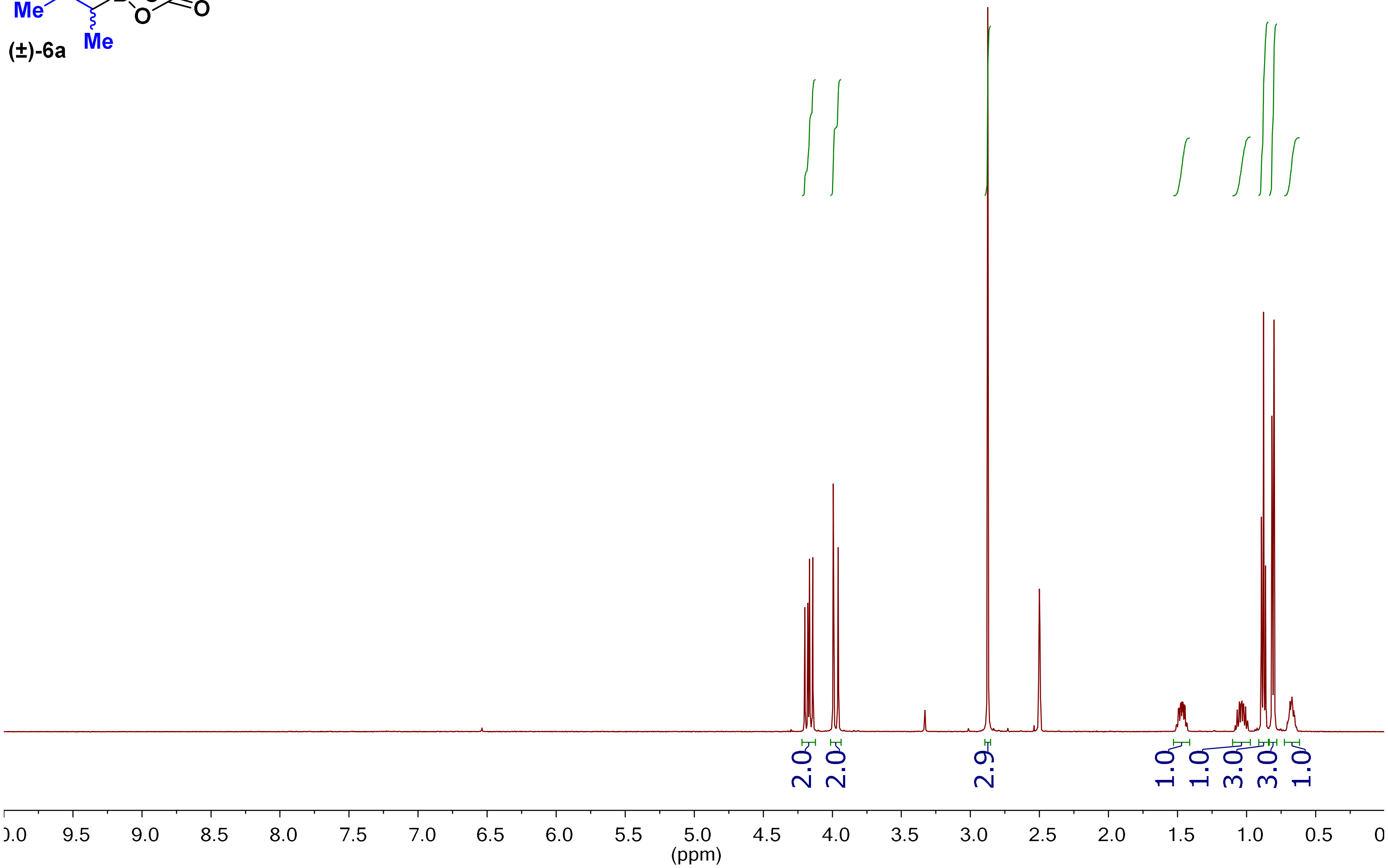

Supplementary Figure 108| ^{1}H-NMR spectrum (500 MHz, DMSO-*d6*) for **(±)-6b**.

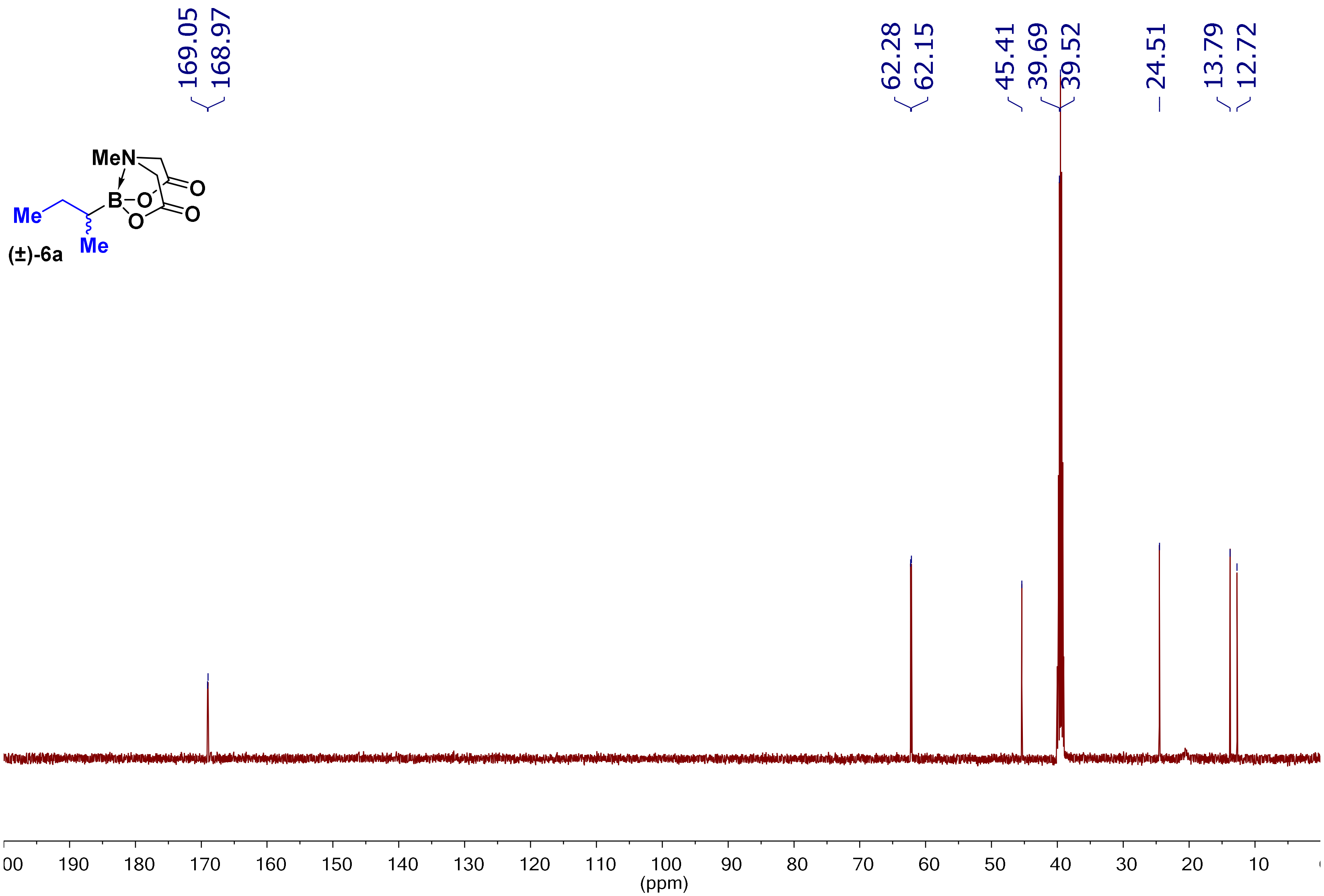

Supplementary Figure 109| ^{13}C-NMR spectrum (126 MHz, DMSO-*d6*) for **(±)-6b**.

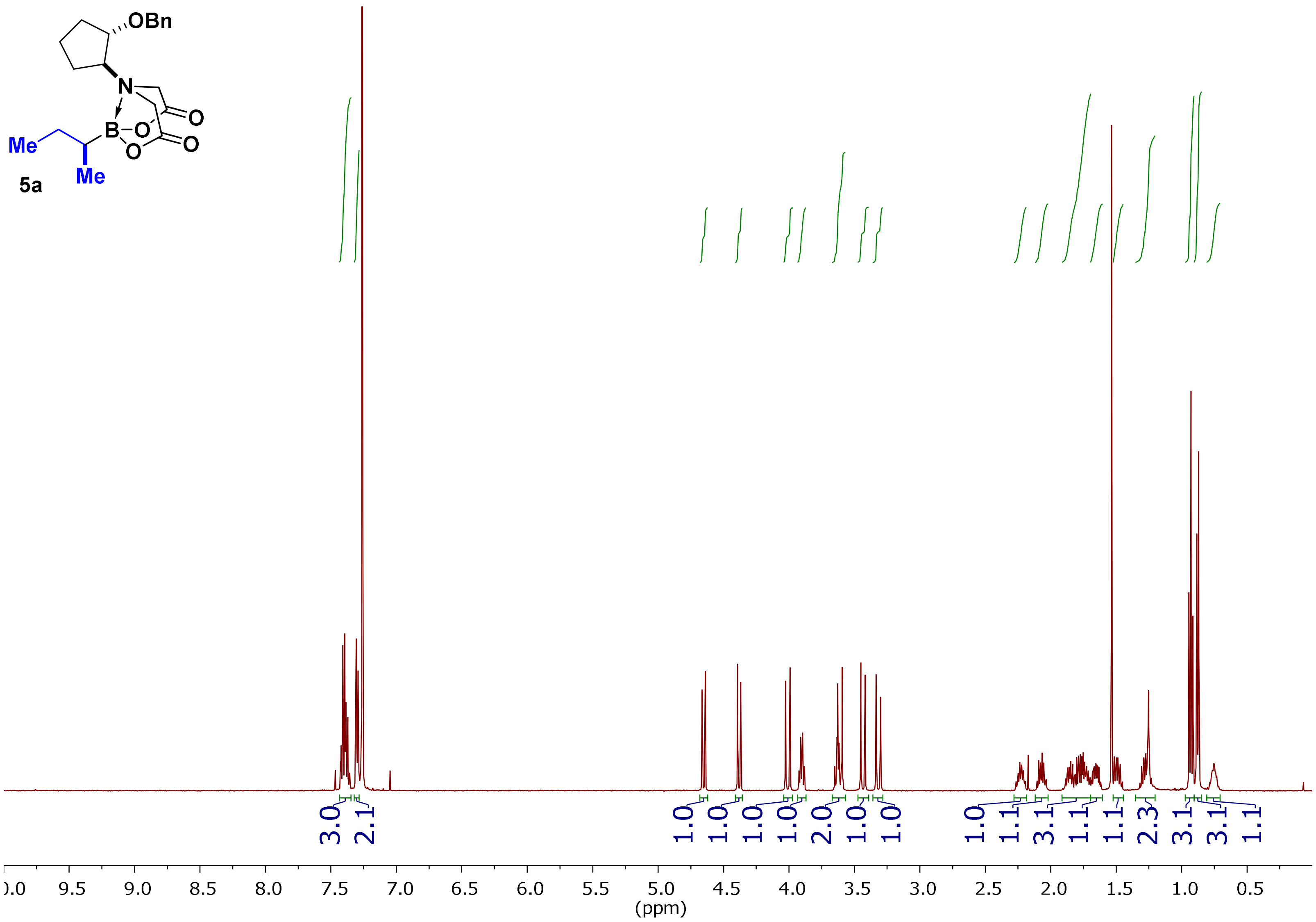

Supplementary Figure 110| ^{1}H-NMR spectrum (500 MHz, $CDCl_3$) for **5a**.

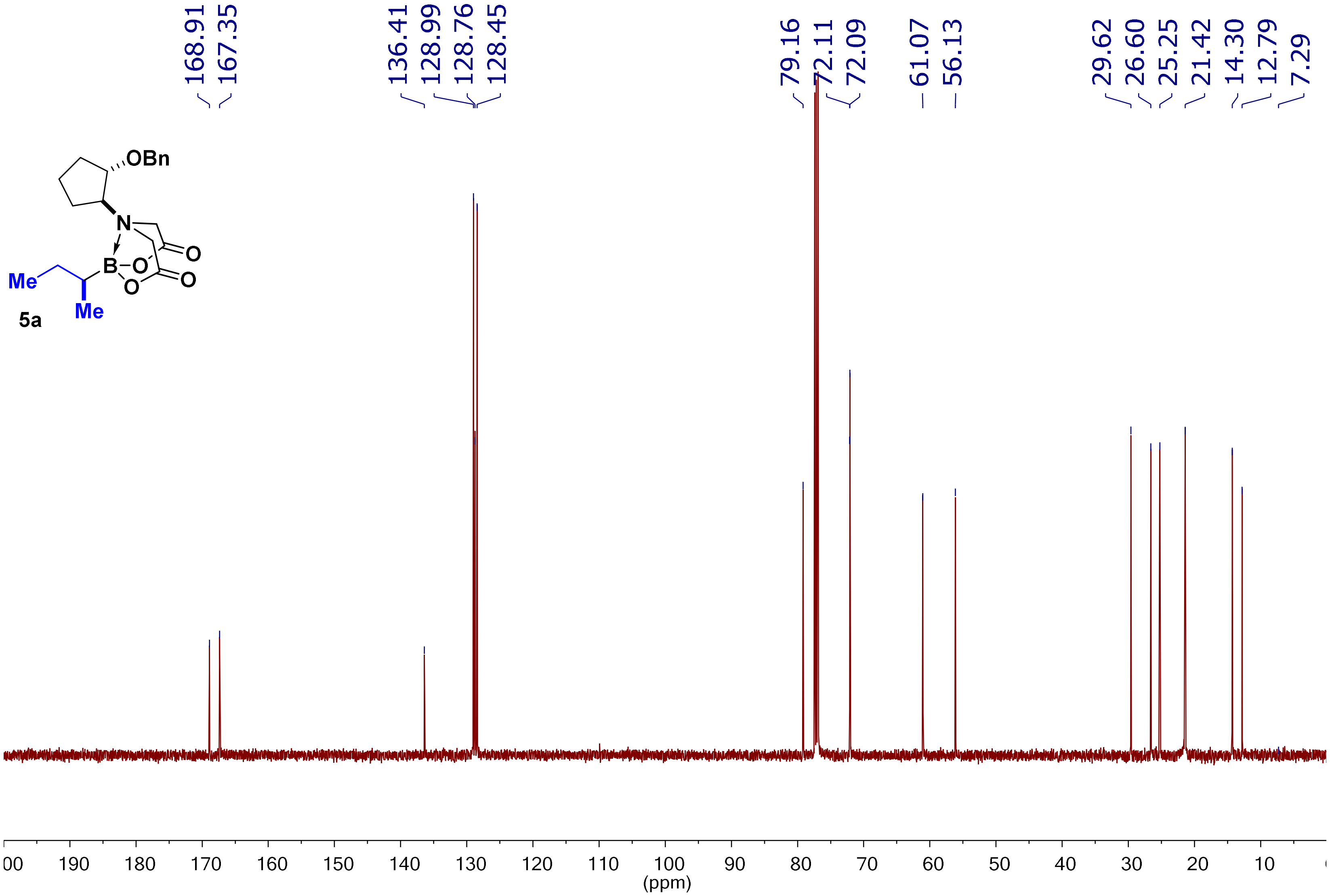

Supplementary Figure 111| ^{13}C-NMR spectrum (126 MHz, $CDCl_3$) for **5a**.

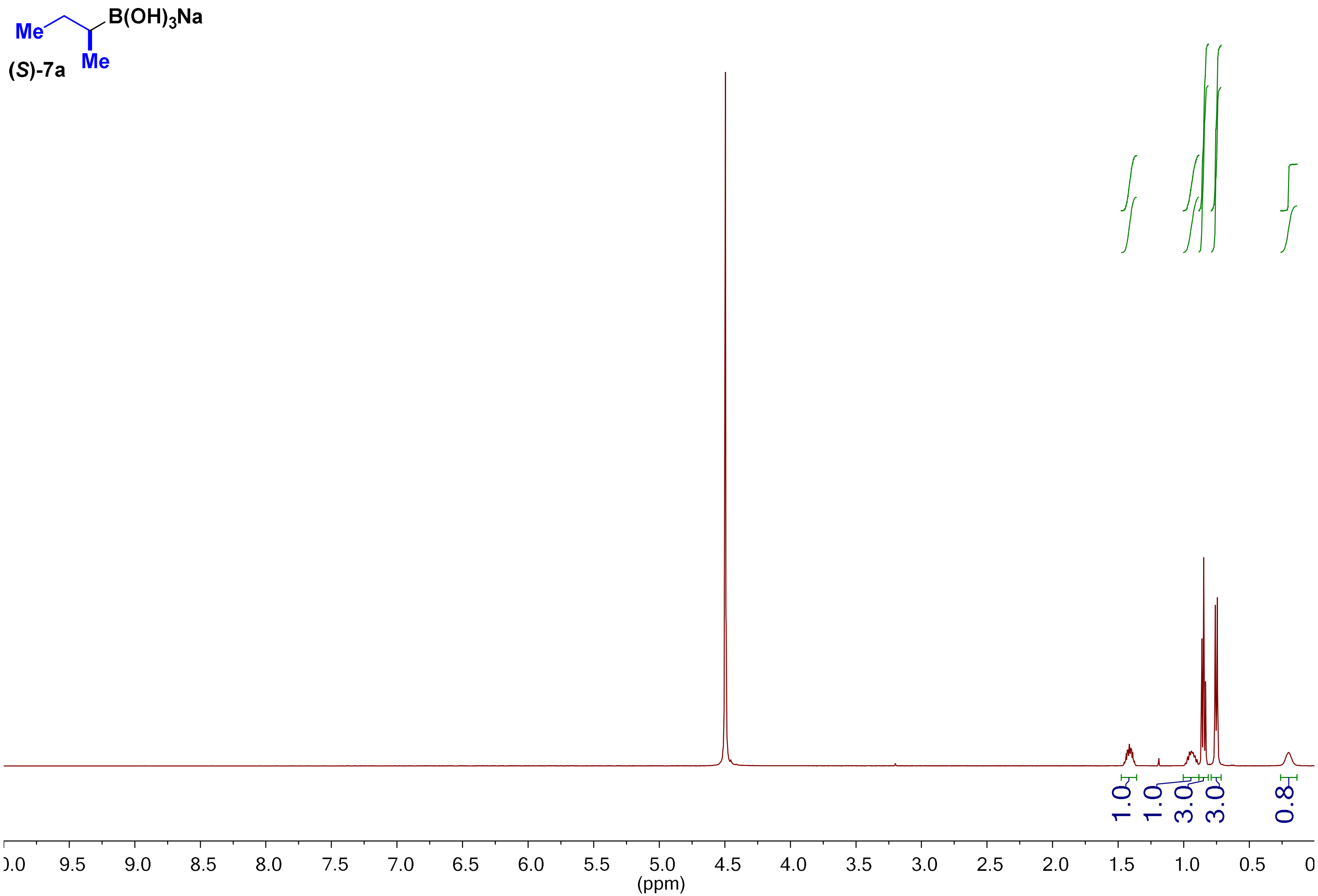

Supplementary Figure 112| ^{1}H-NMR spectrum (500 MHz, D_2O) for ***(S)*-7a**.

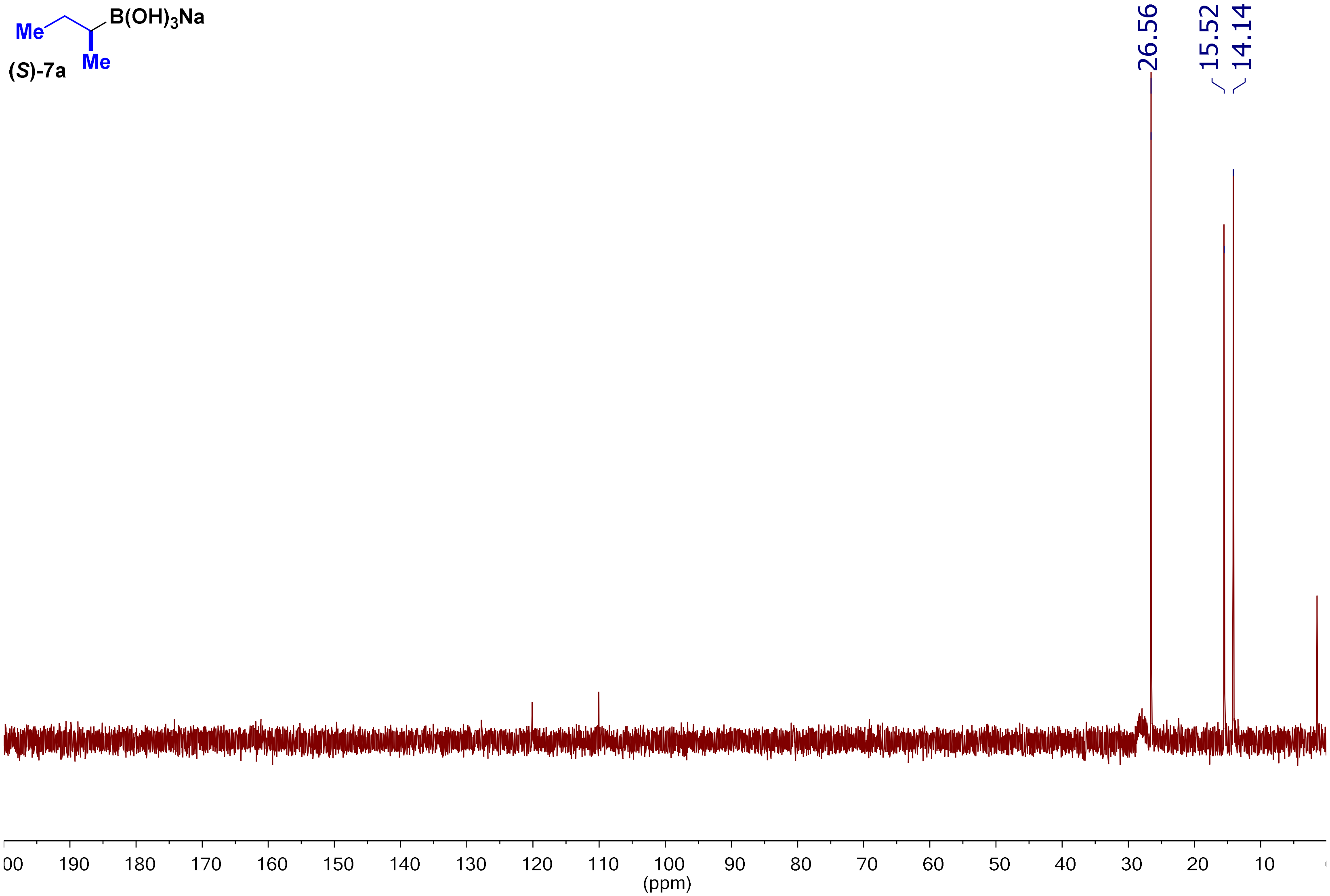

Supplementary Figure 113| ^{13}C-NMR spectrum (126 MHz, D_2O) for **(*S*)-7a**.

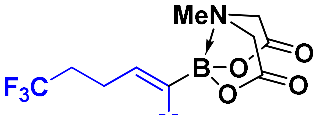

SI-15

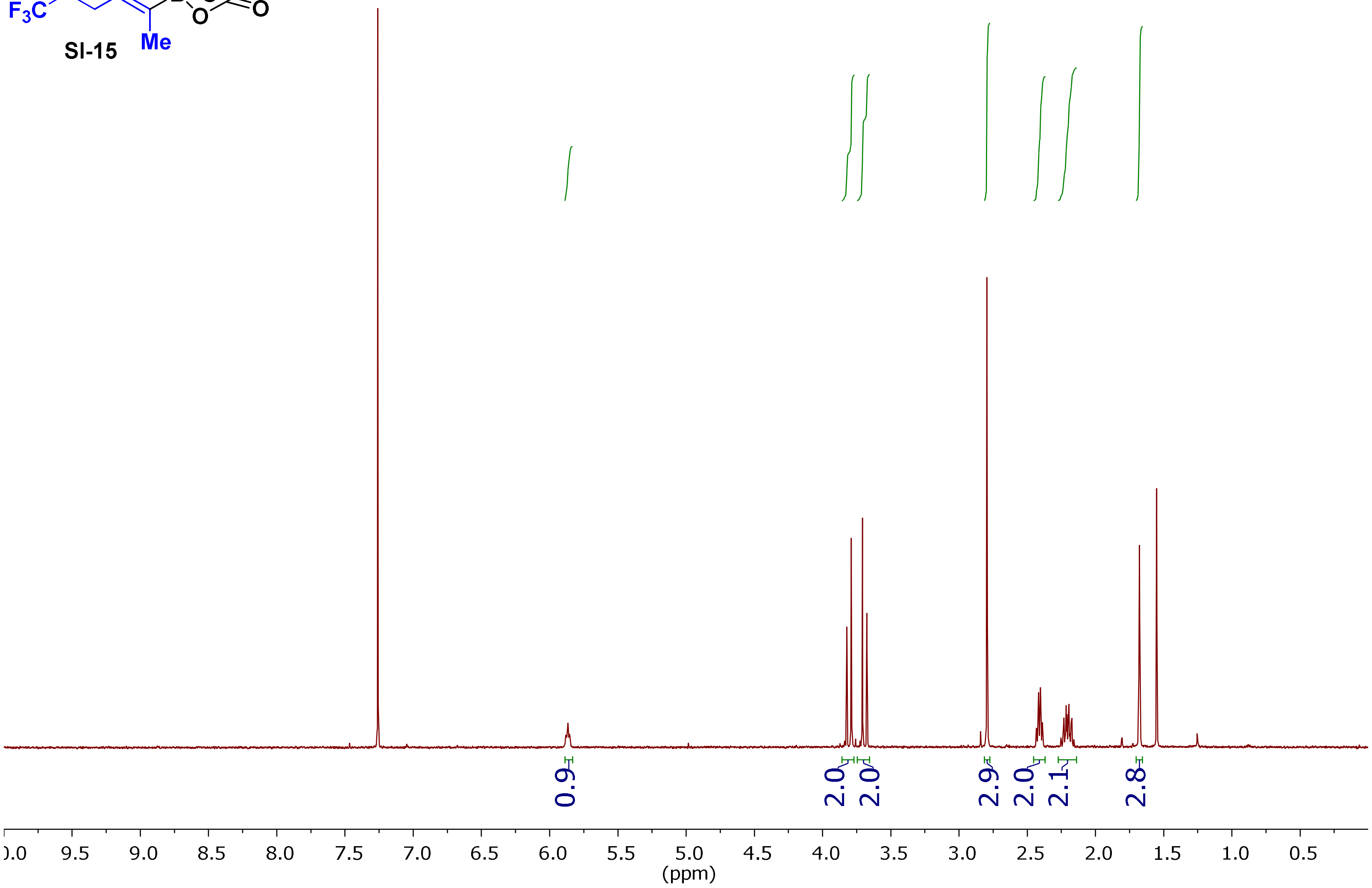

Supplementary Figure 114| ^{1}H-NMR spectrum (500 MHz, $CDCl_3$) for **SI-15**.

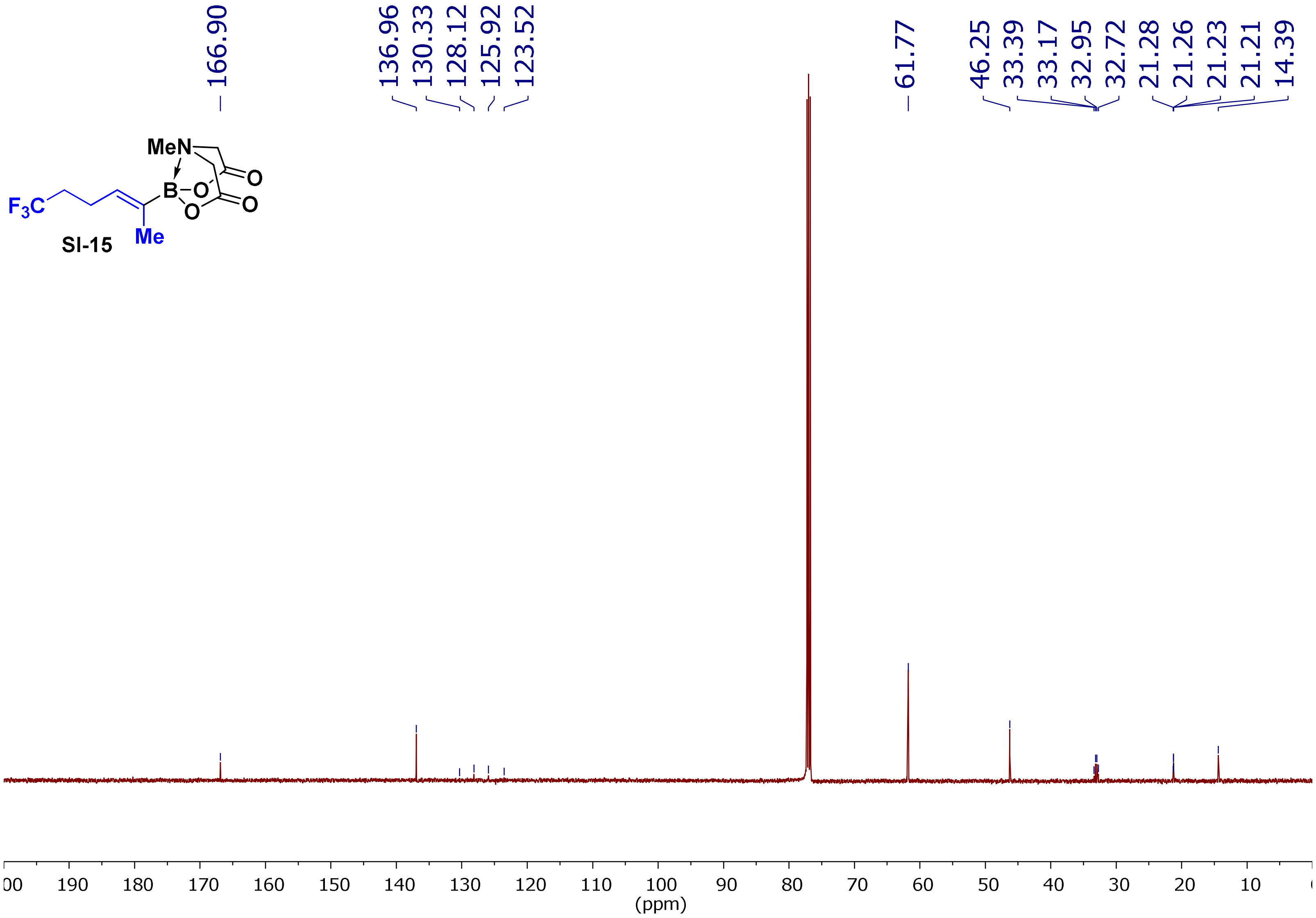

Supplementary Figure 115| ^{13}C-NMR spectrum (126 MHz, $CDCl_3$) for **SI-15**.

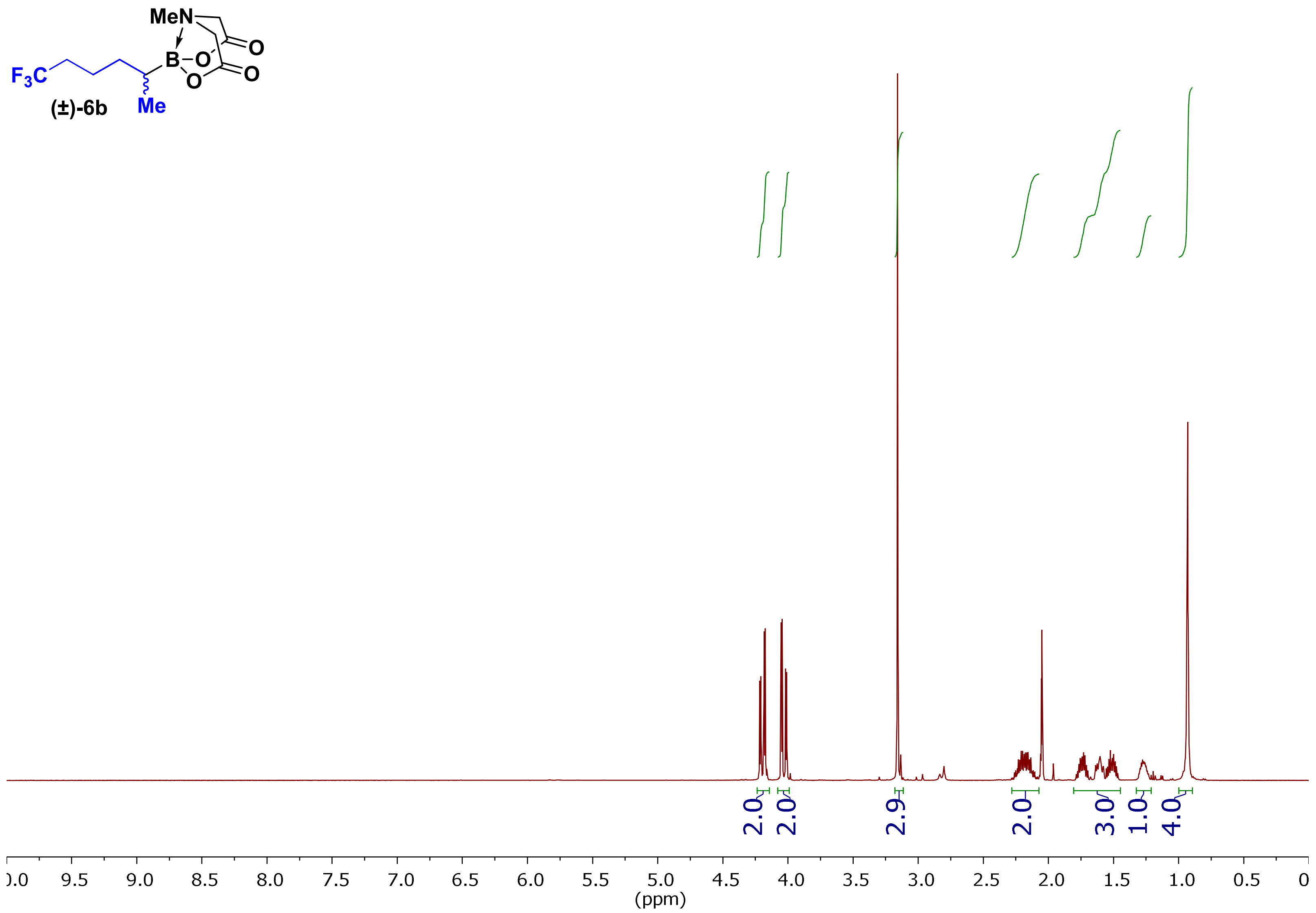

Supplementary Figure 116| ^{1}H-NMR spectrum (500 MHz, $CDCl_3$) for **(±)-6b**.

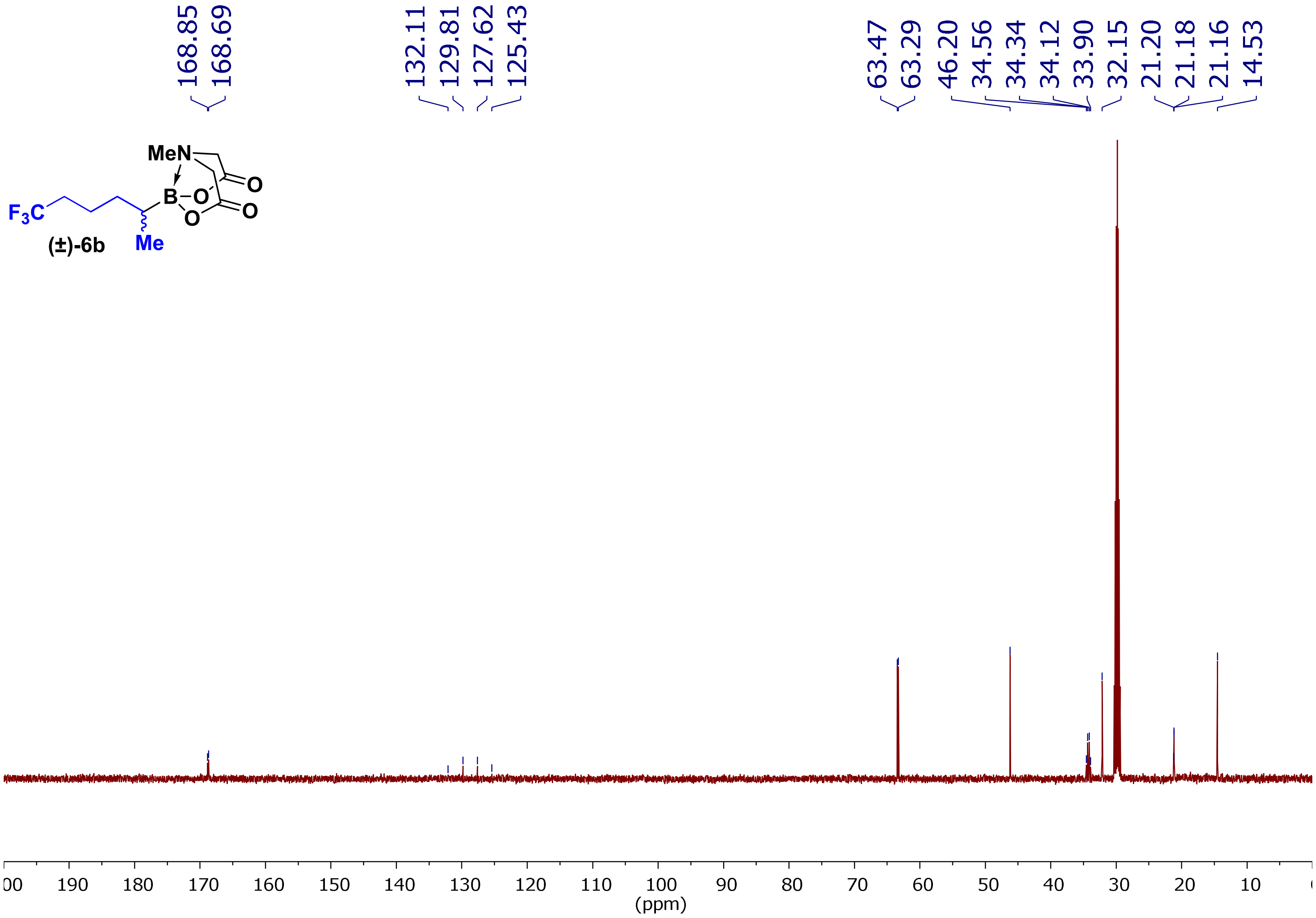

Supplementary Figure 117| ^{13}C-NMR spectrum (126 MHz, $CDCl_3$) for **(±)-6b**.

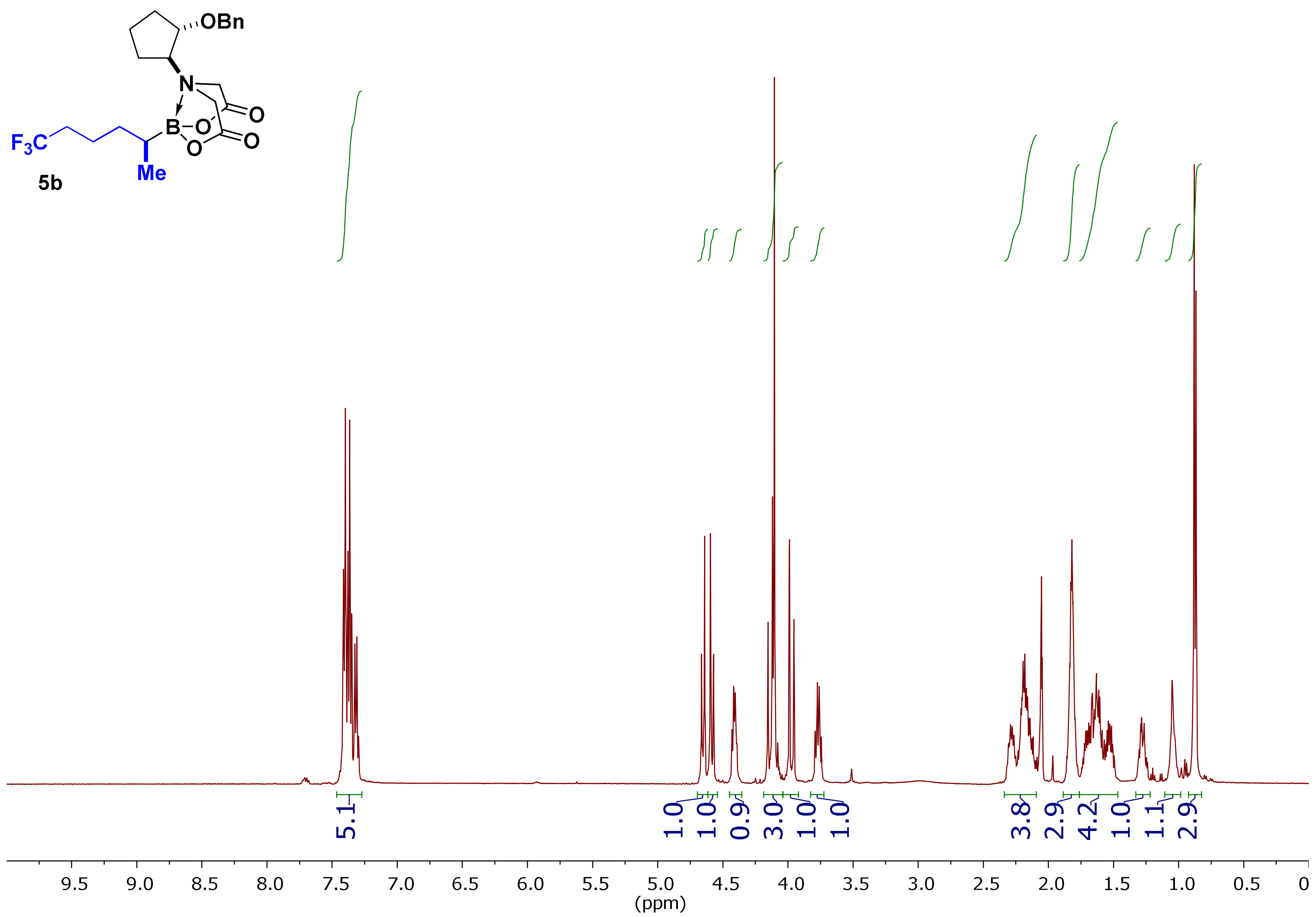

Supplementary Figure 118| ^{1}H-NMR spectrum (500 MHz, acetone-*d6*) for **5b**.

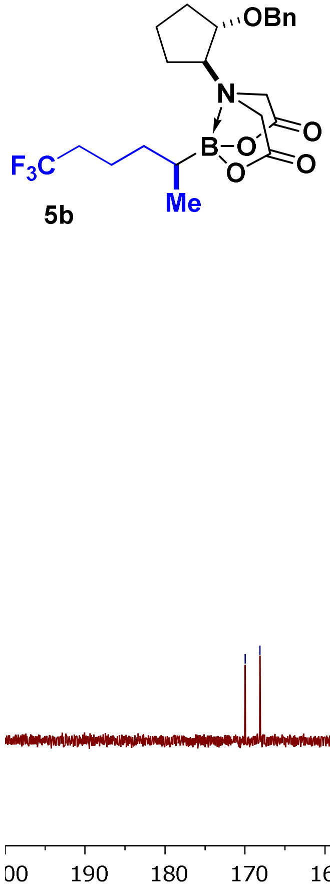

5b

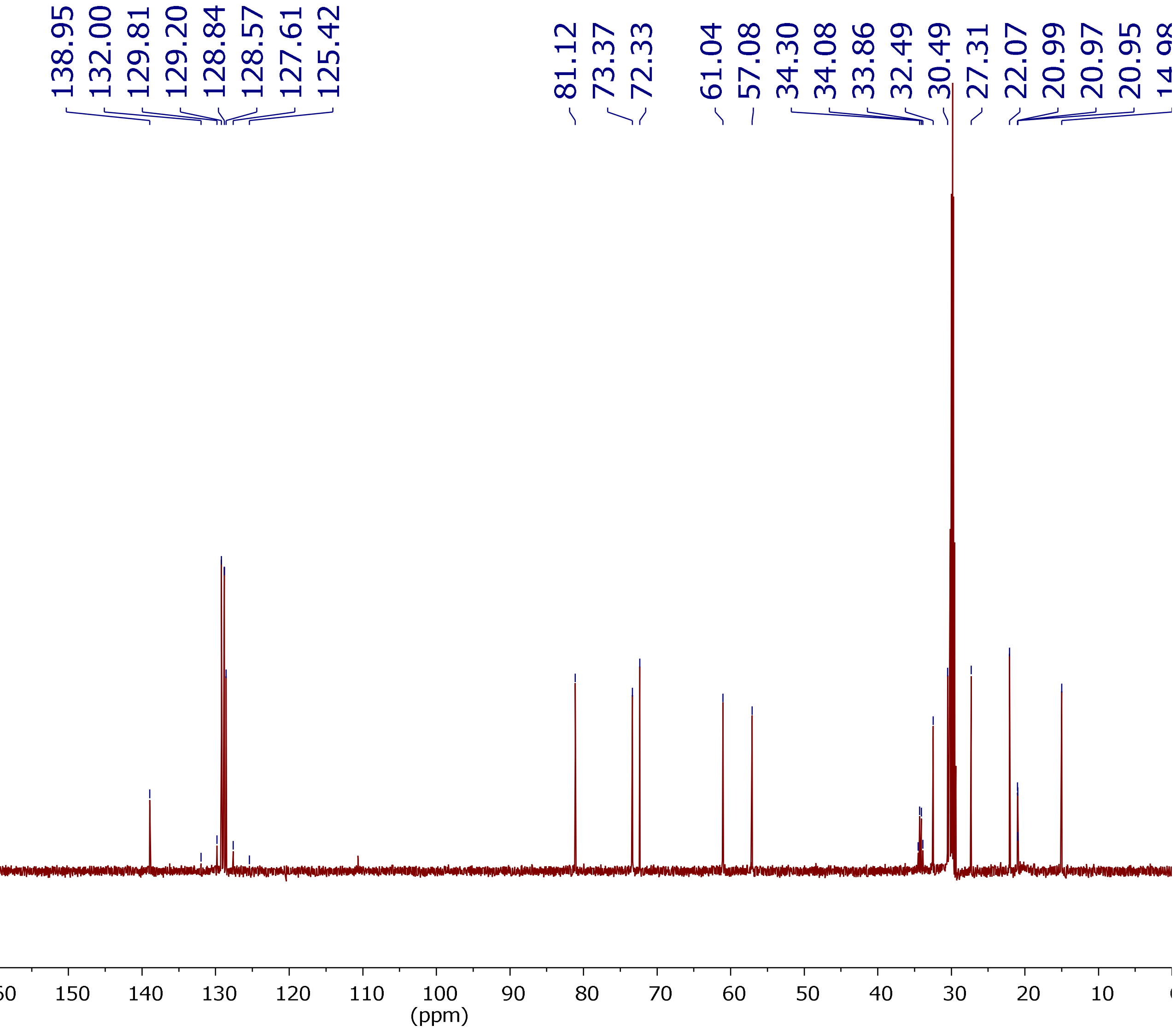

Supplementary Figure 119| ^{13}C-NMR spectrum (126 MHz, acetone-*d6*) for **5b**.

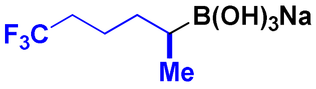

(-)-7b

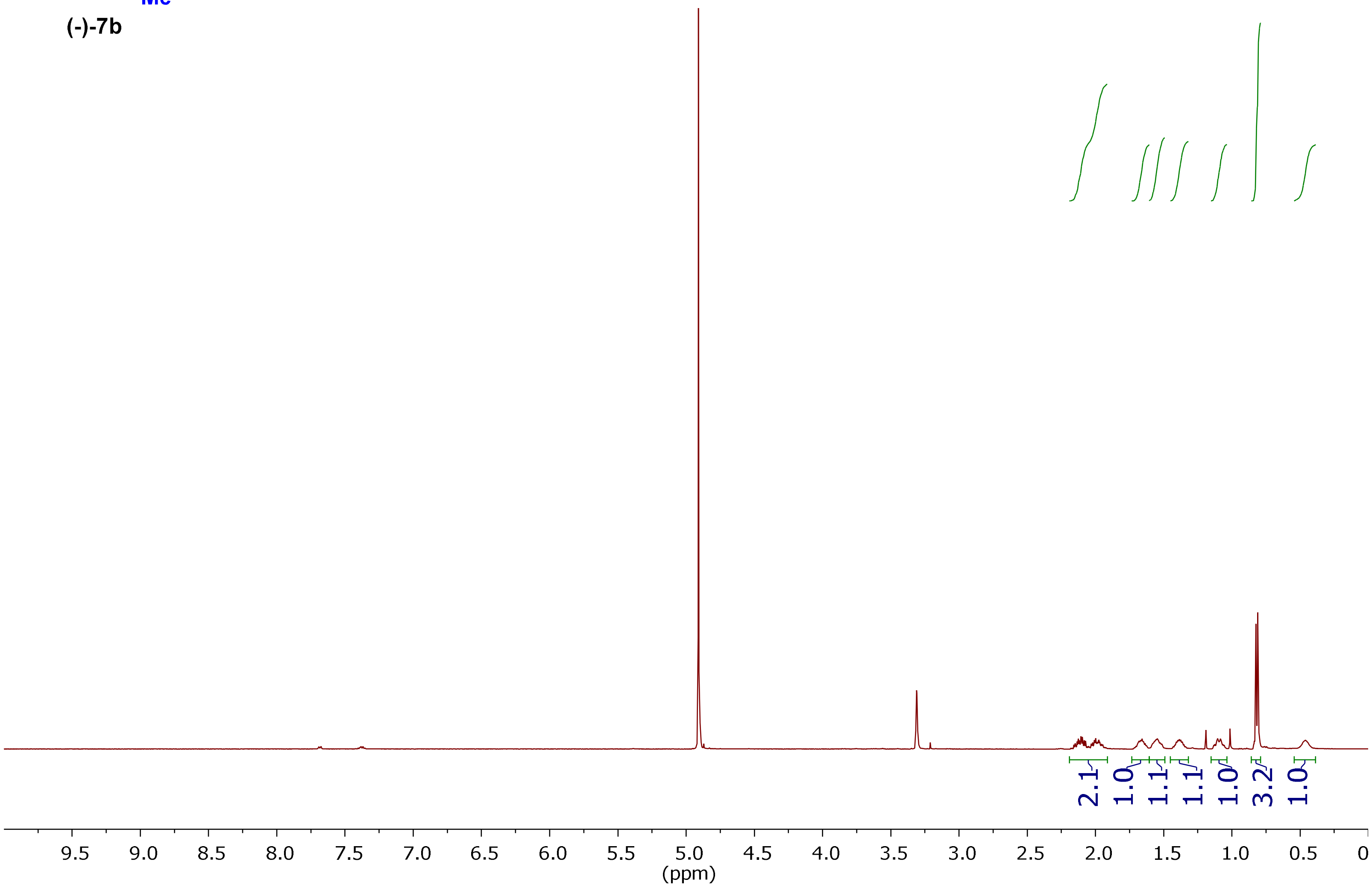

Supplementary Figure 120| ^{1}H-NMR spectrum (500 MHz, CD_3OD) for **(-)-7b**.

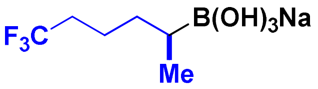

(-)-7b

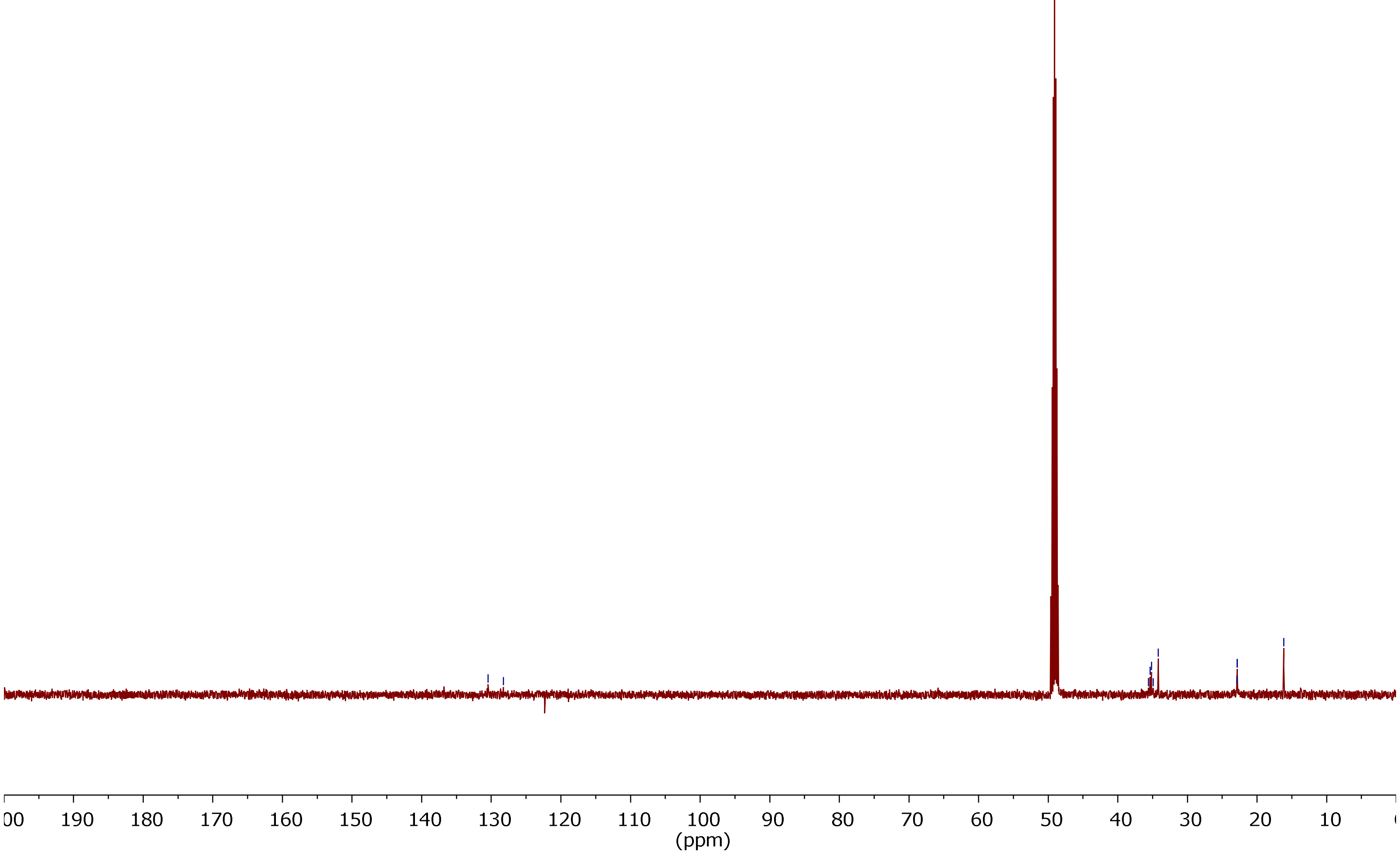

Supplementary Figure 121| ^{13}C-NMR spectrum (126 MHz, CD_3OD) for **(-)-7b**.

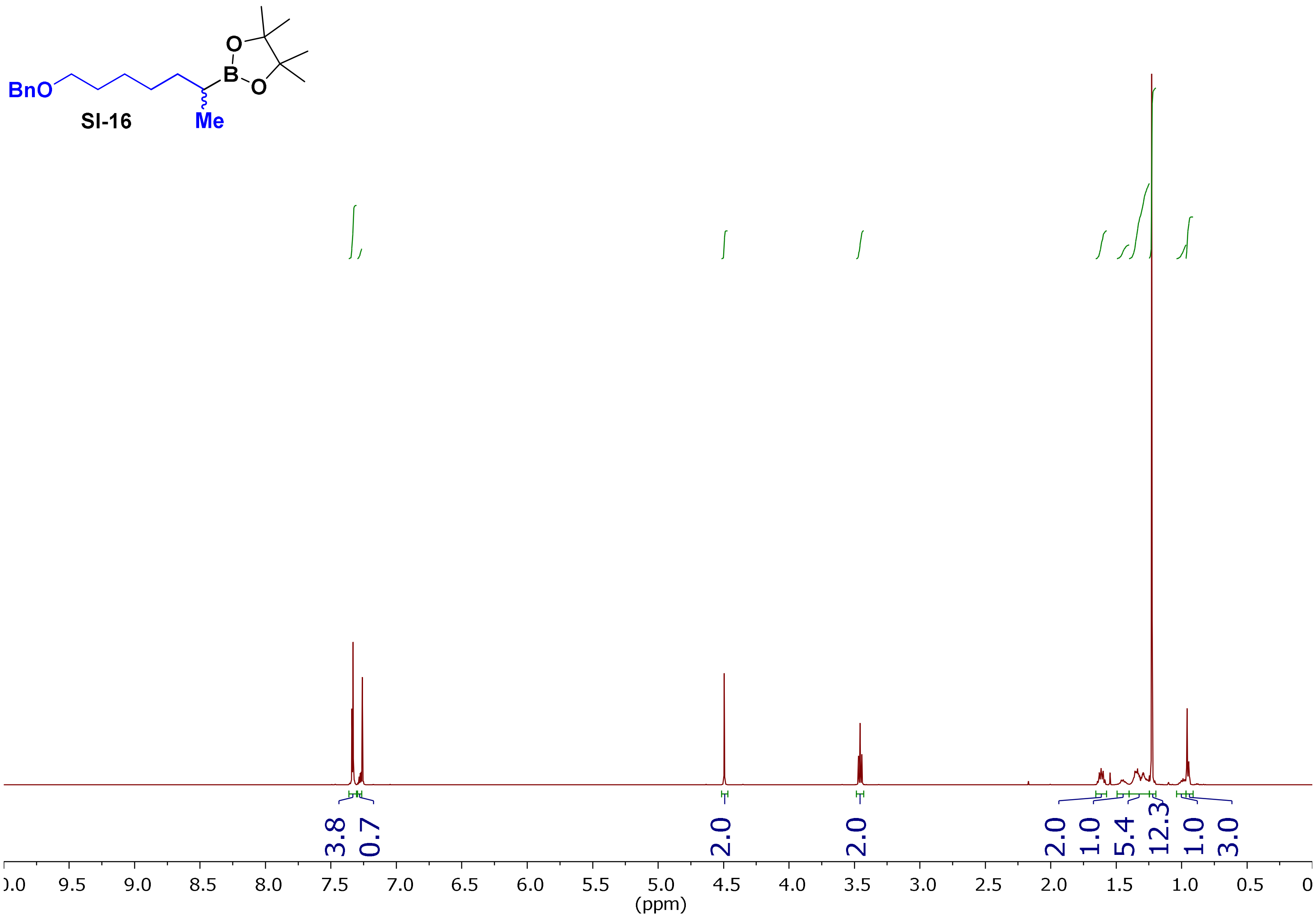

Supplementary Figure 122| ^{1}H-NMR spectrum (500 MHz, $CDCl_3$) for **SI-16**.

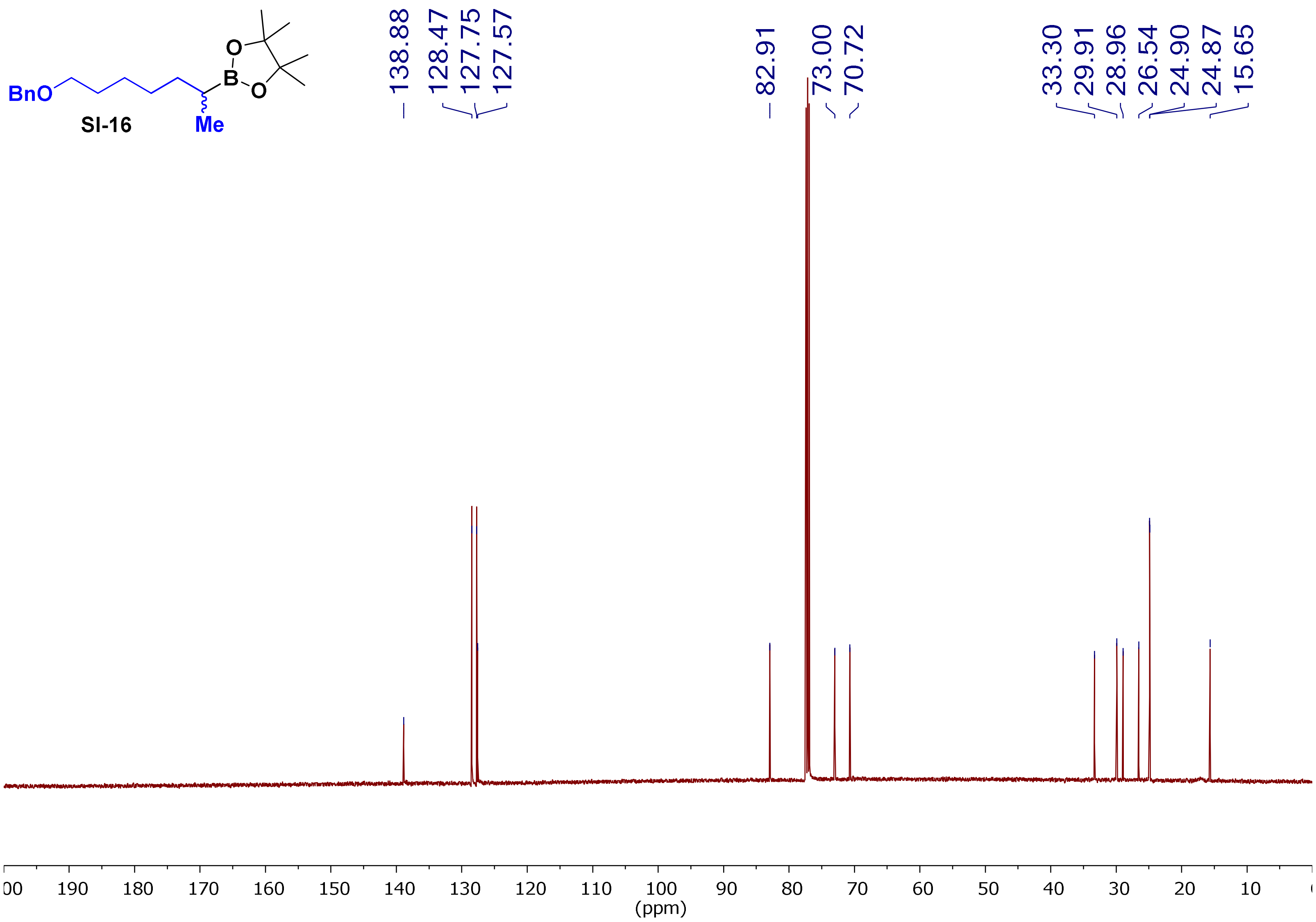

Supplementary Figure 123| ^{13}C-NMR spectrum (126 MHz, $CDCl_3$) for **SI-16**.

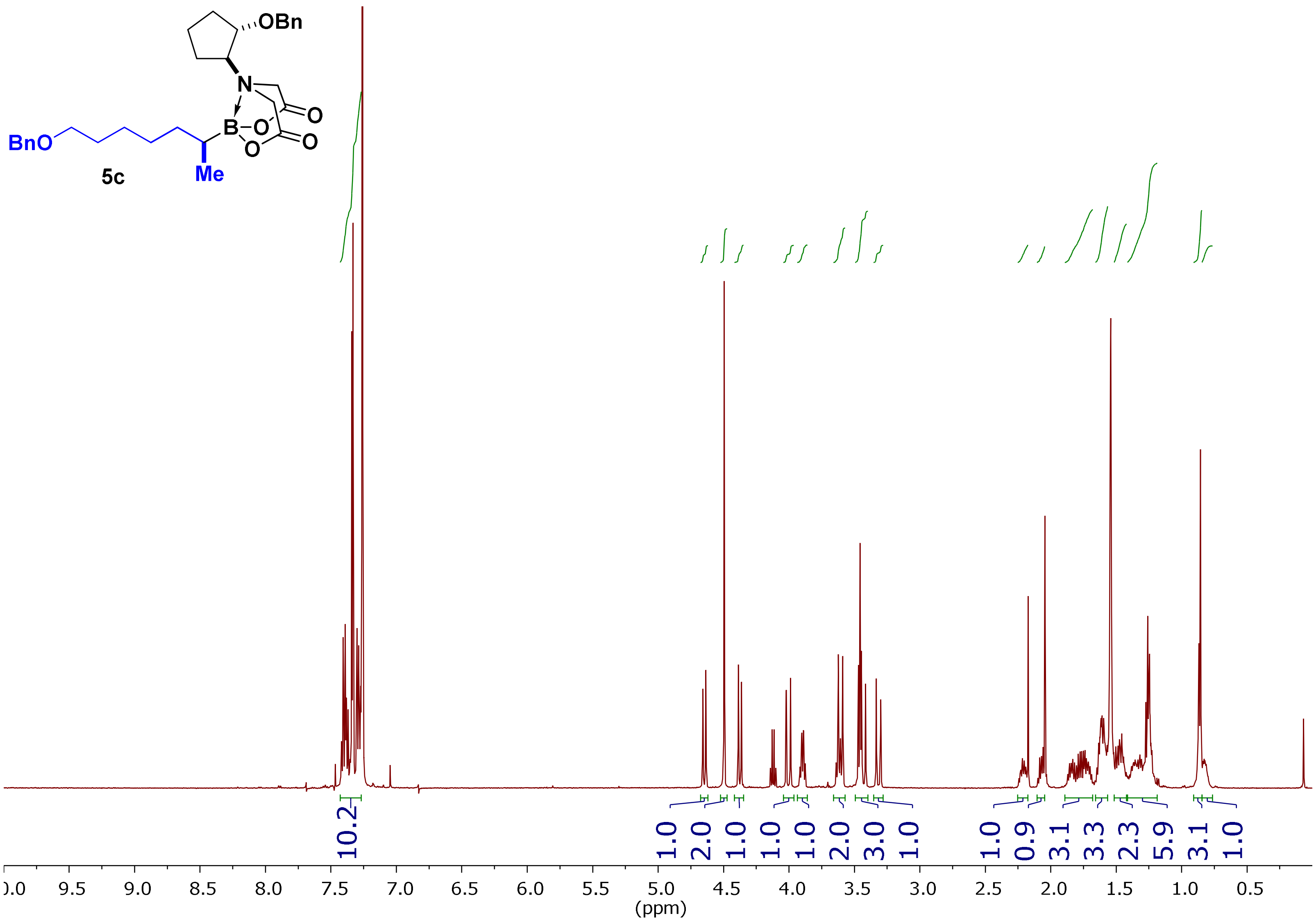

Supplementary Figure 124| ^{1}H-NMR spectrum (500 MHz, $CDCl_3$) for **5c**.

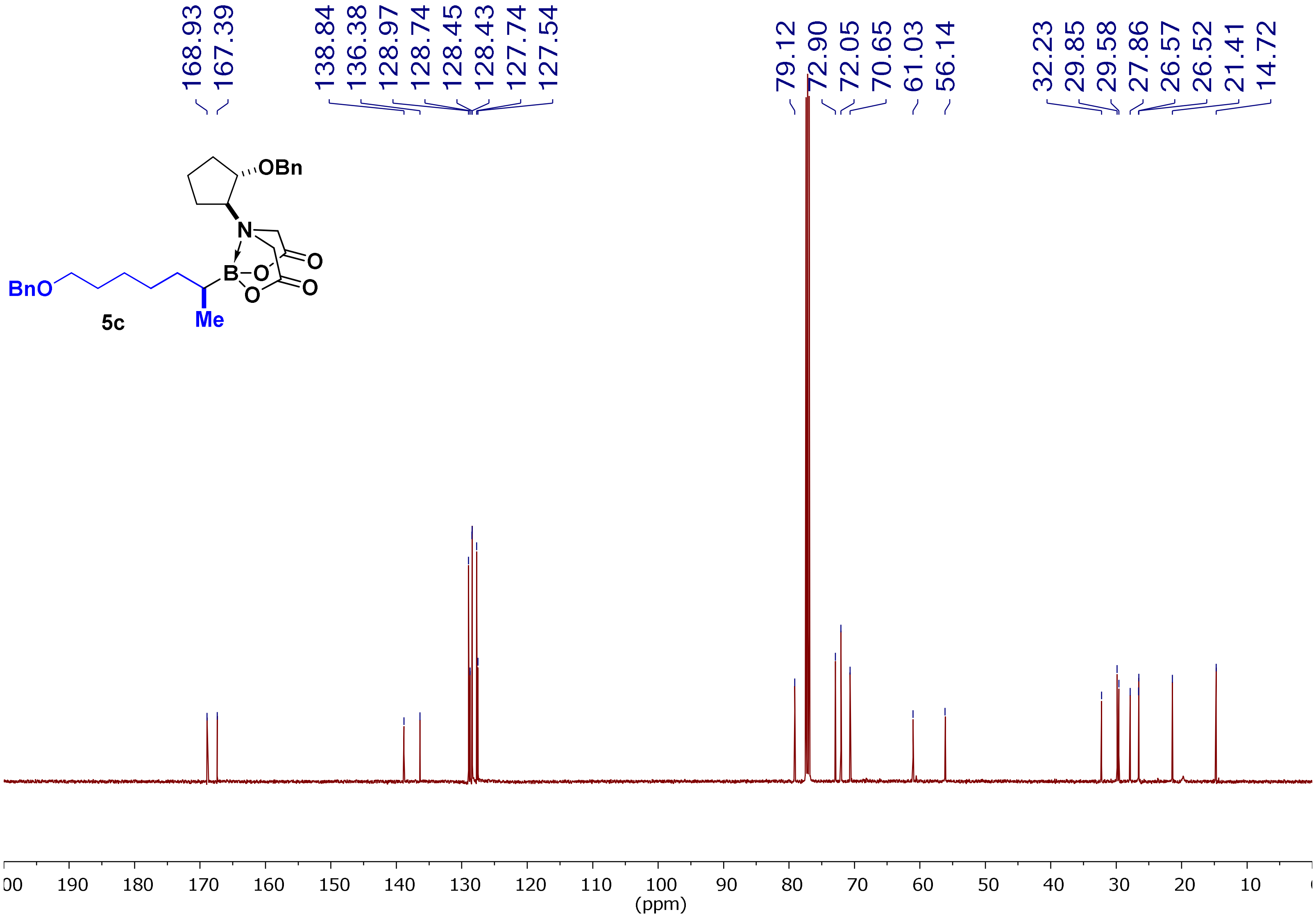

Supplementary Figure 125| ^{13}C-NMR spectrum (126 MHz, $CDCl_3$) for **5c**.

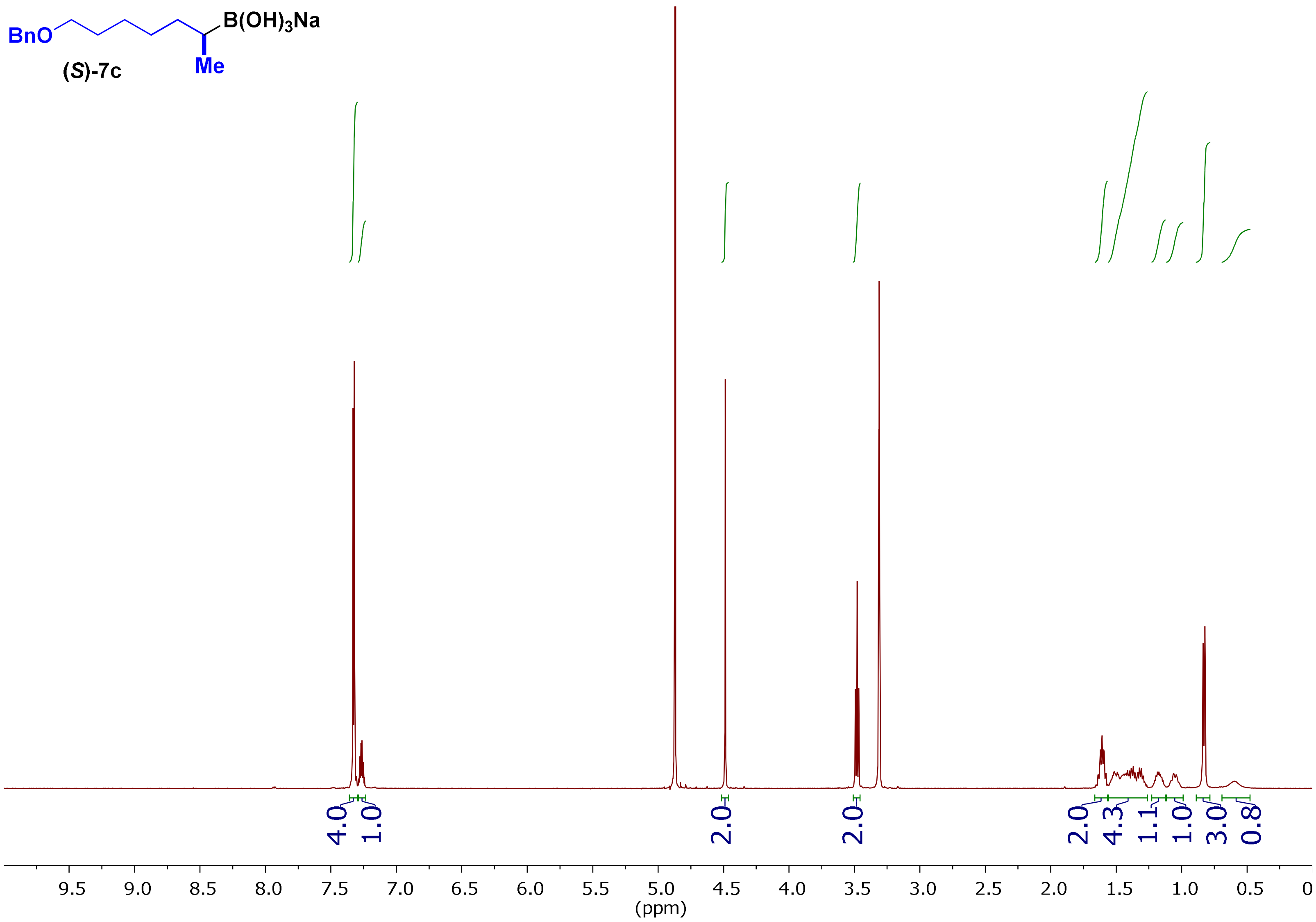

Supplementary Figure 126| ^{1}H-NMR spectrum (500 MHz, CD_3OD) for ***(S)*-7c**.

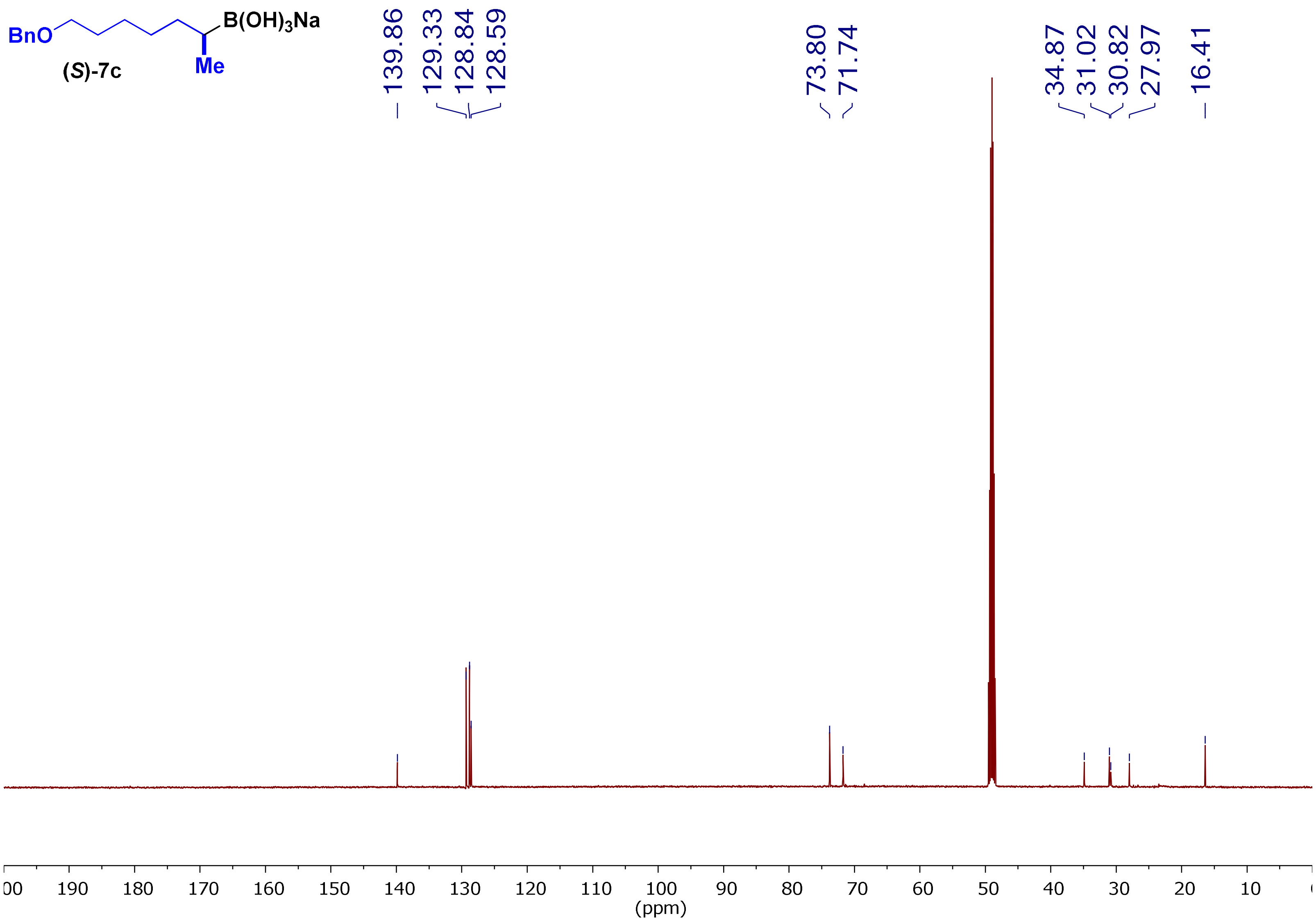

Supplementary Figure 127| ^{13}C-NMR spectrum (126 MHz, CD_3OD) for **(*S*)-7c**.

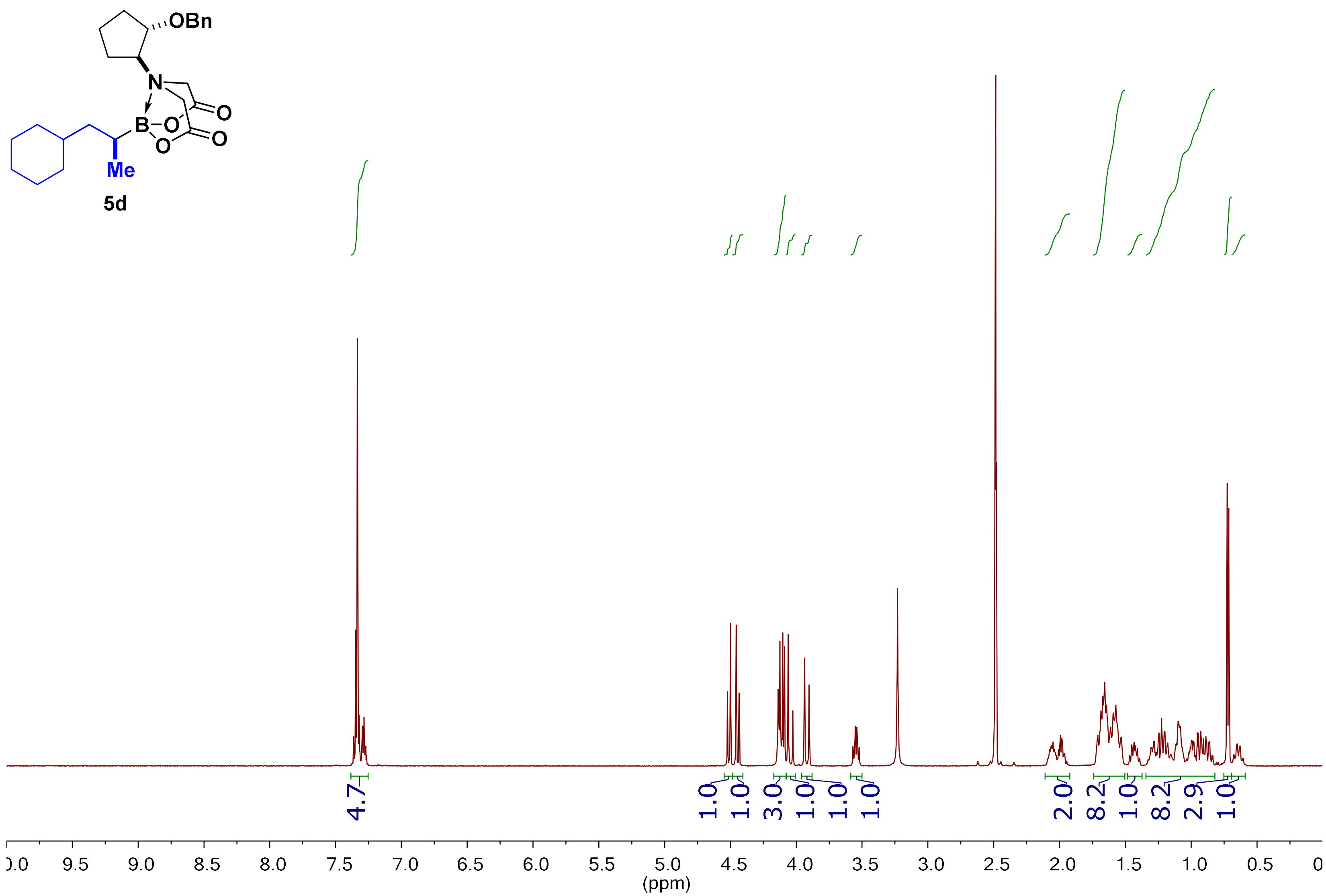

Supplementary Figure 128| ^{1}H-NMR spectrum (500 MHz, DMSO-*d6*) for **5d**.

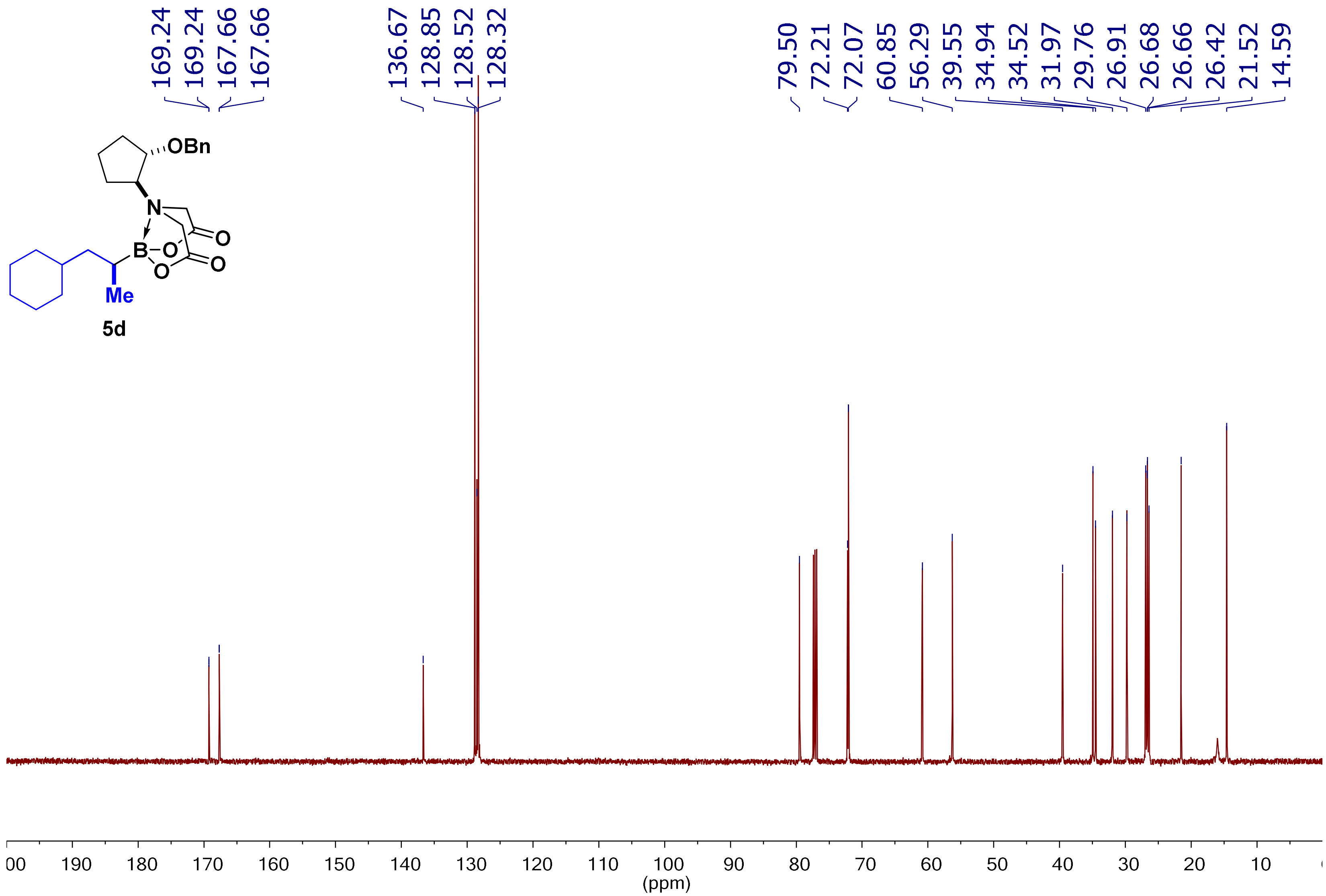

Supplementary Figure 129| ^{13}C-NMR spectrum (126 MHz, $CDCl_3$) for **5d**.

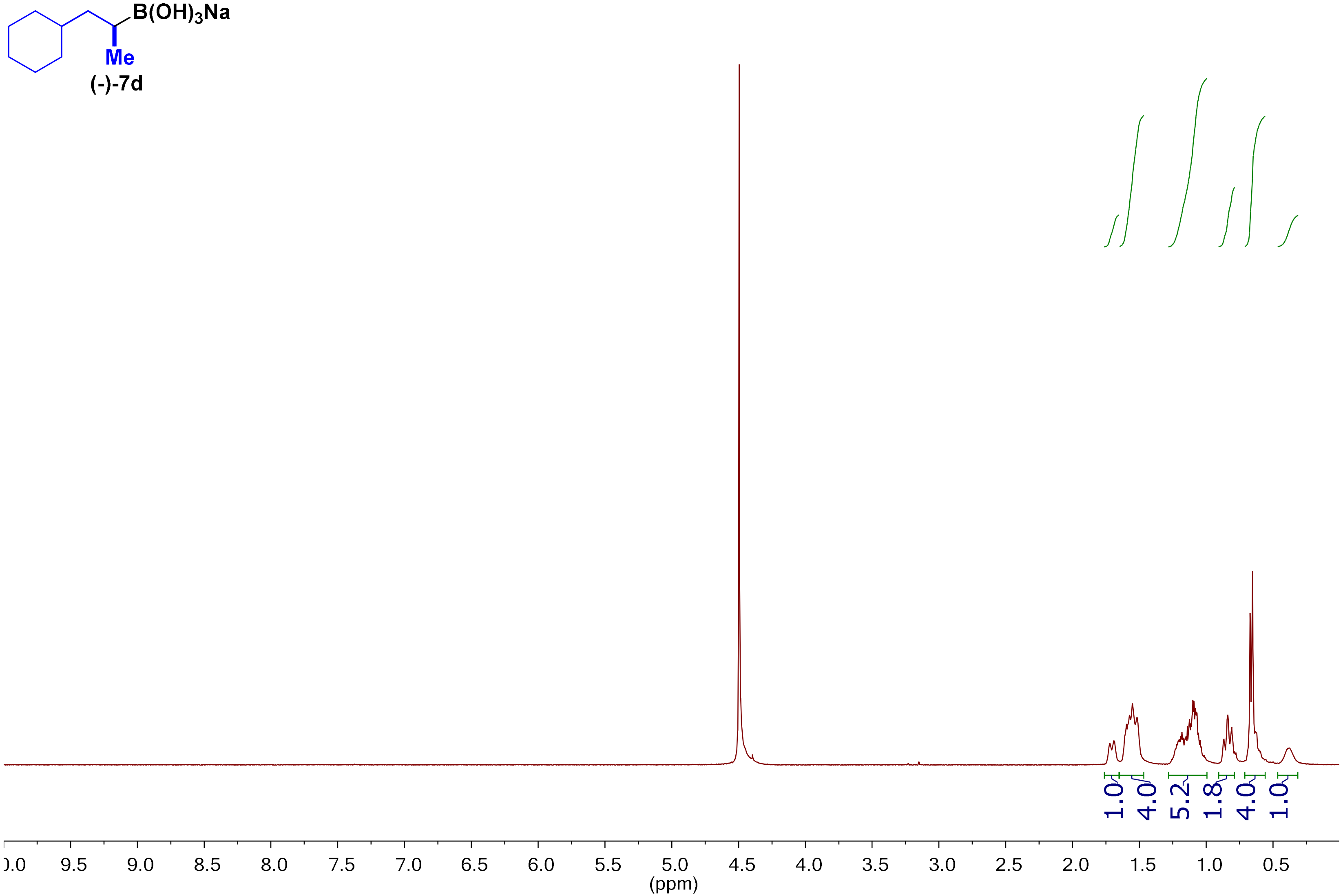

Supplementary Figure 130| ^{1}H-NMR spectrum (500 MHz, D_2O) for **(-)-7d**.

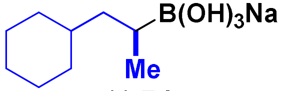

(-)-7d

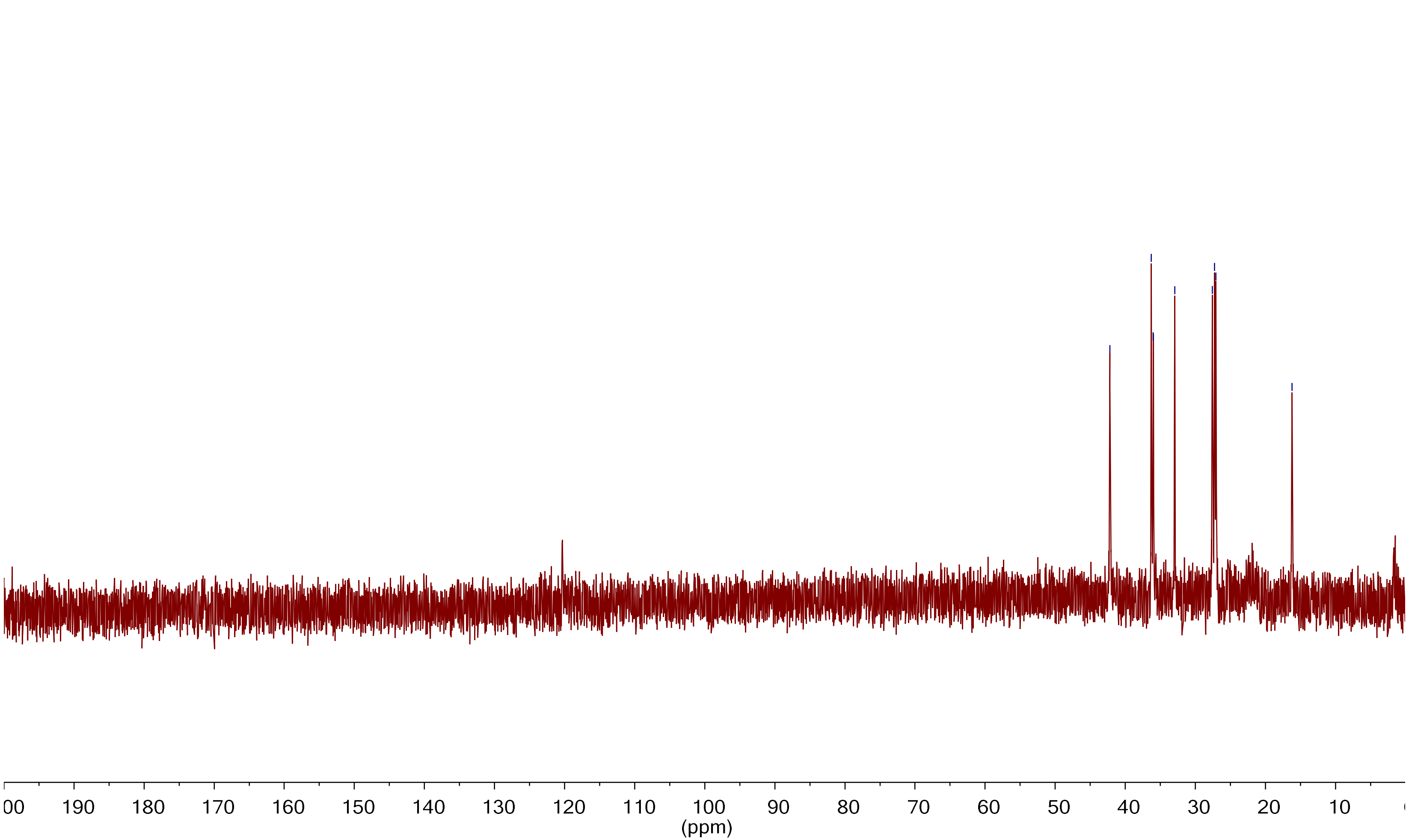

Supplementary Figure 131| ^{13}C-NMR spectrum (126 MHz, D_2O) for **(-)-7d**.

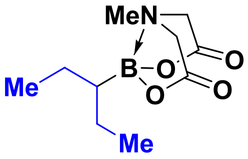

6e

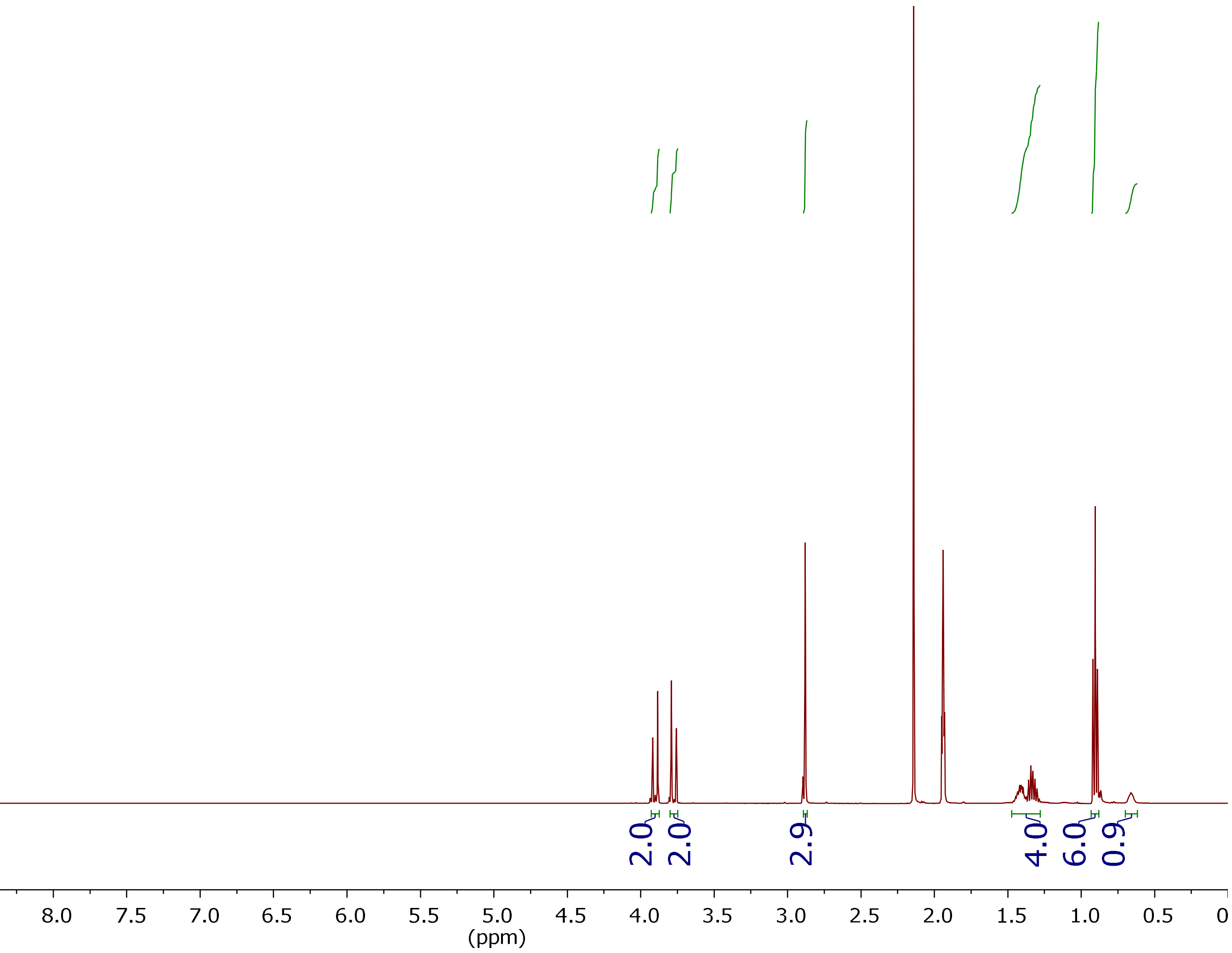

Supplementary Figure 132| ^{1}H-NMR spectrum (500 MHz, CD_3CN) for **6e**.

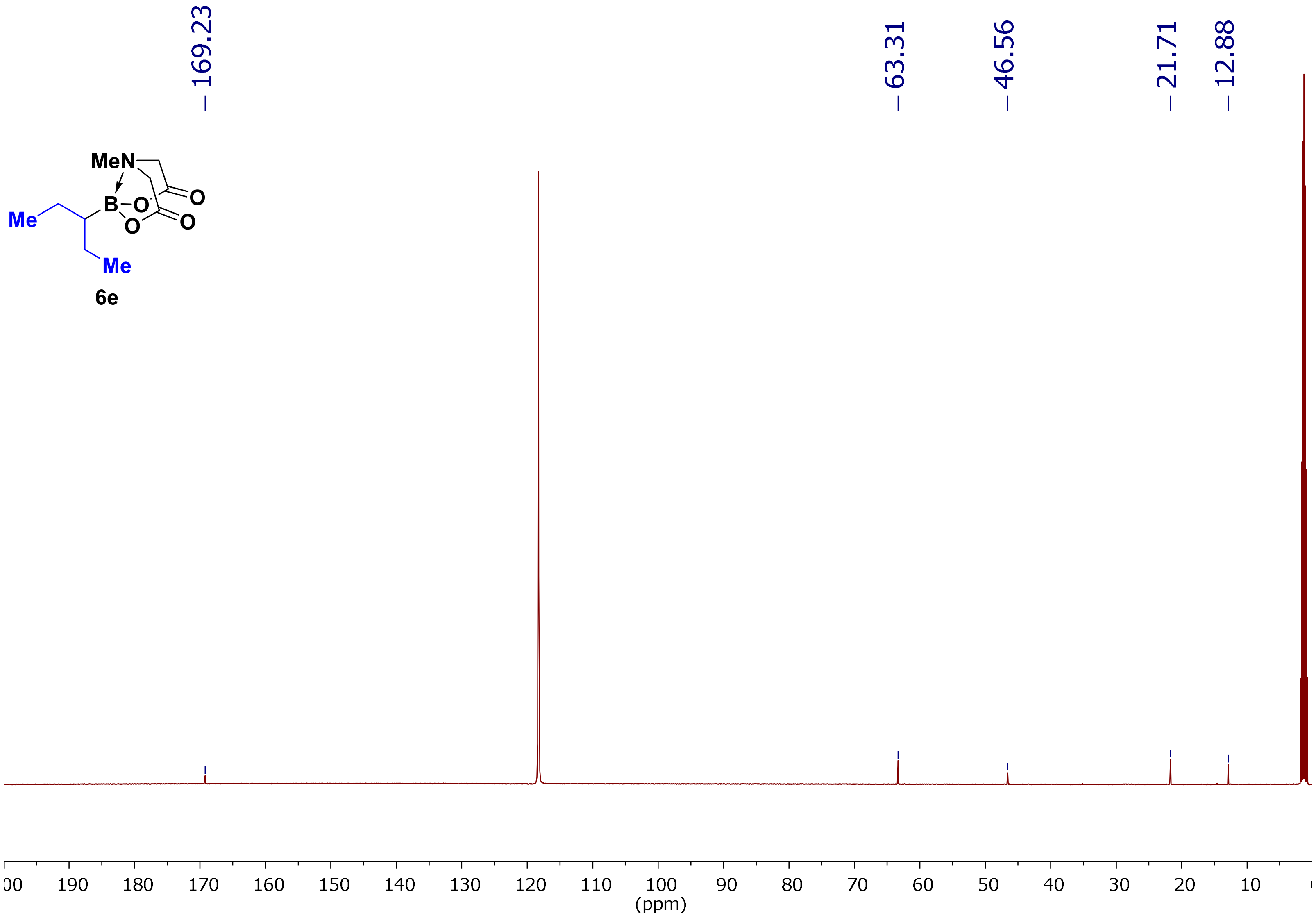

Supplementary Figure 133| ^{13}C-NMR spectrum (126 MHz, CD_3CN) for **6e**.

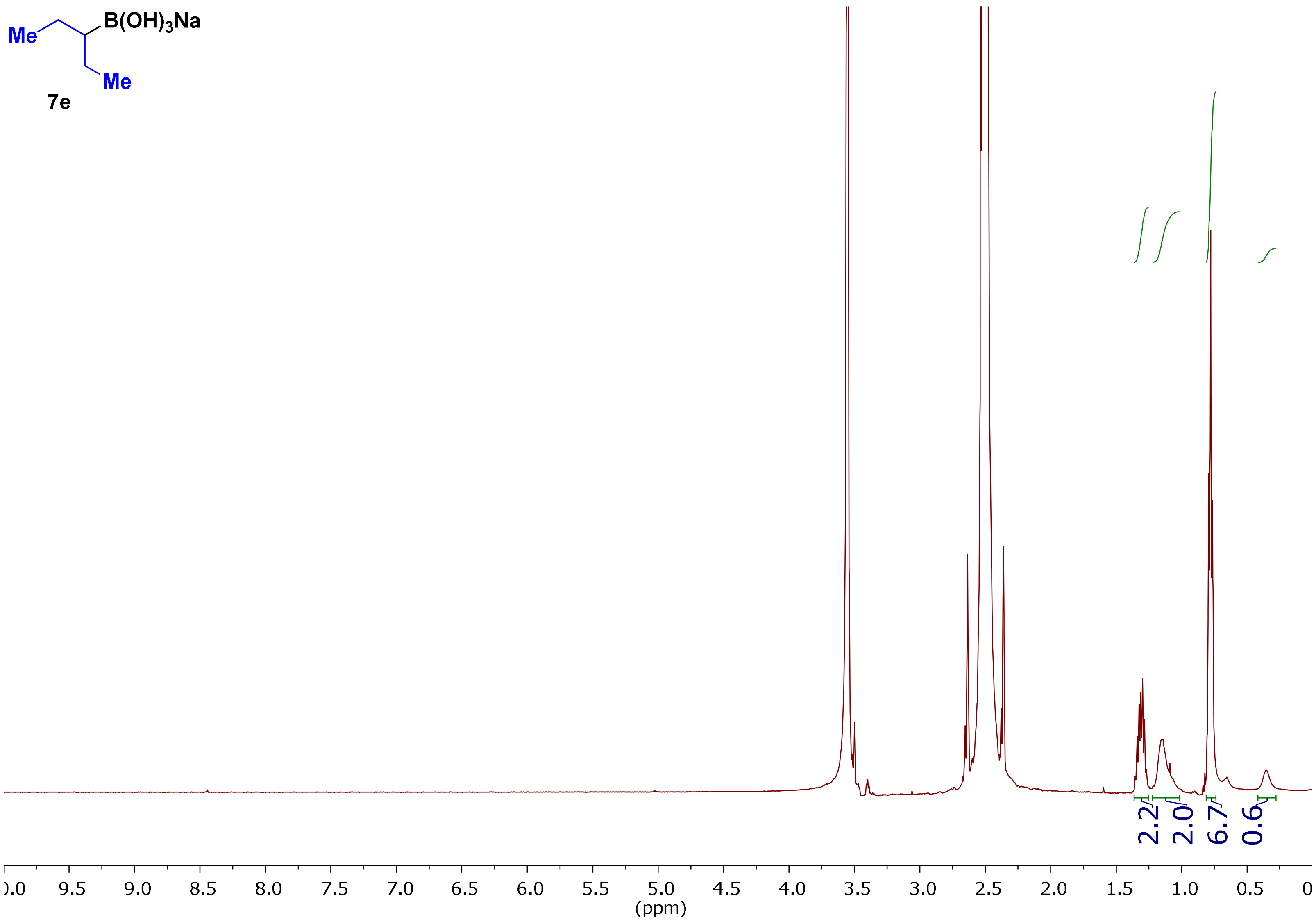

Supplementary Figure 134| ^{1}H-NMR spectrum (500 MHz, DMSO-*d6*) for **7e**.

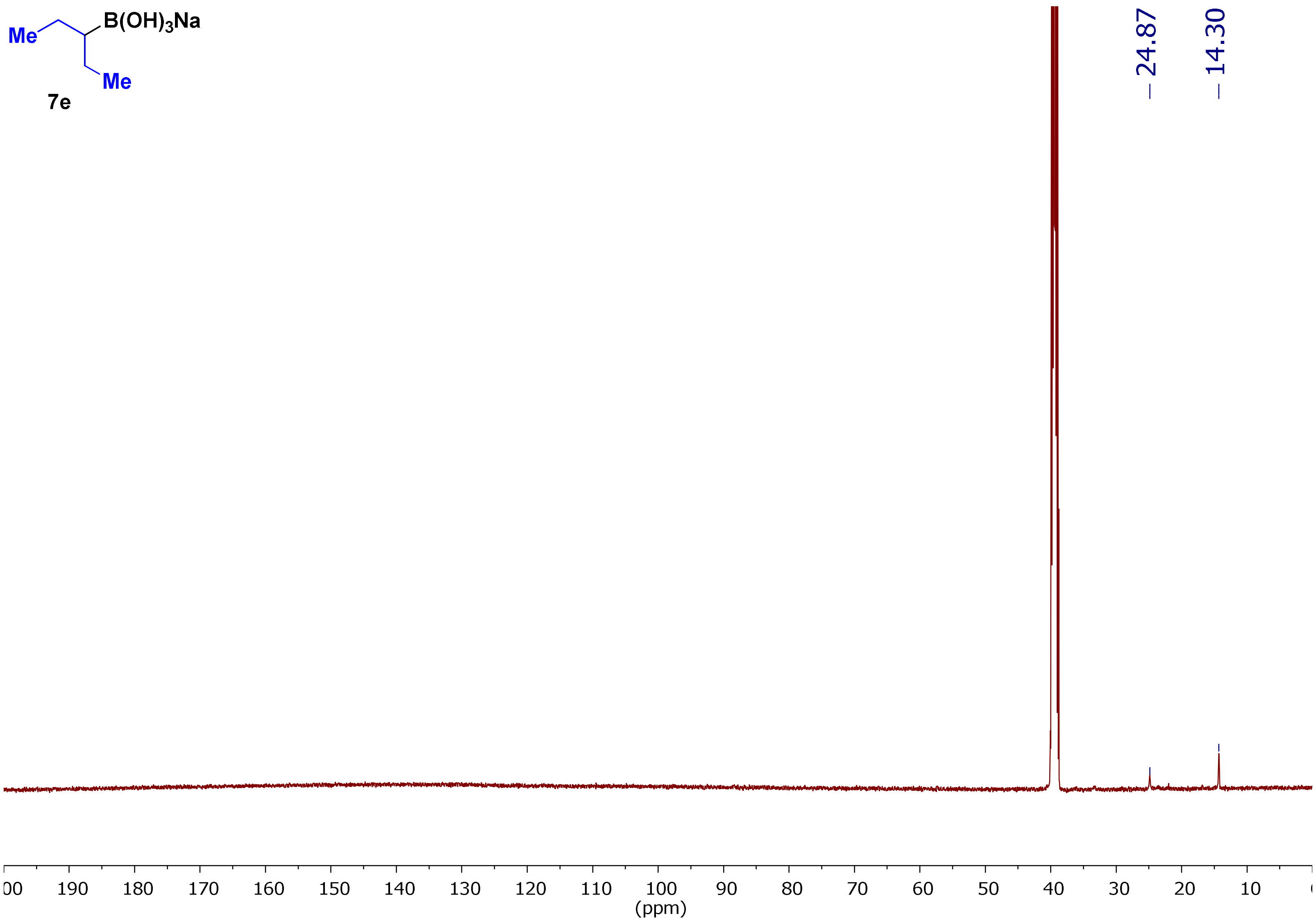

Supplementary Figure 135| ^{13}C-NMR spectrum (126 MHz, DMSO-*d6*) for **7e**.

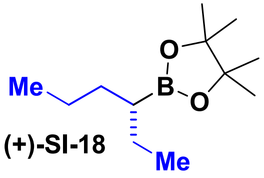

(+)-SI-18

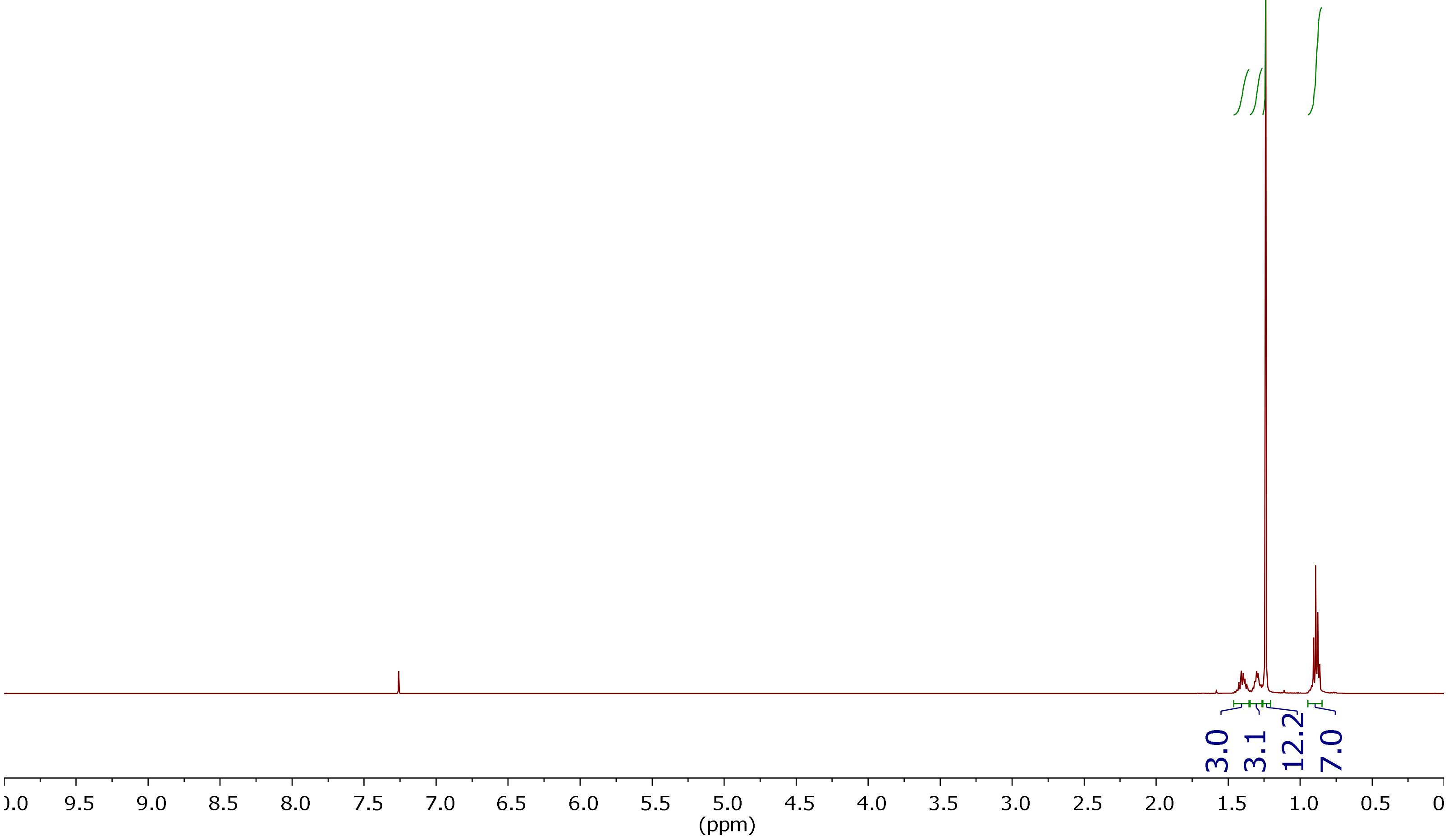

Supplementary Figure 136| ^{1}H-NMR spectrum (500 MHz, $CDCl_3$) for **(+)-SI-18**.

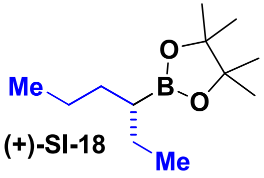

(+)-SI-18

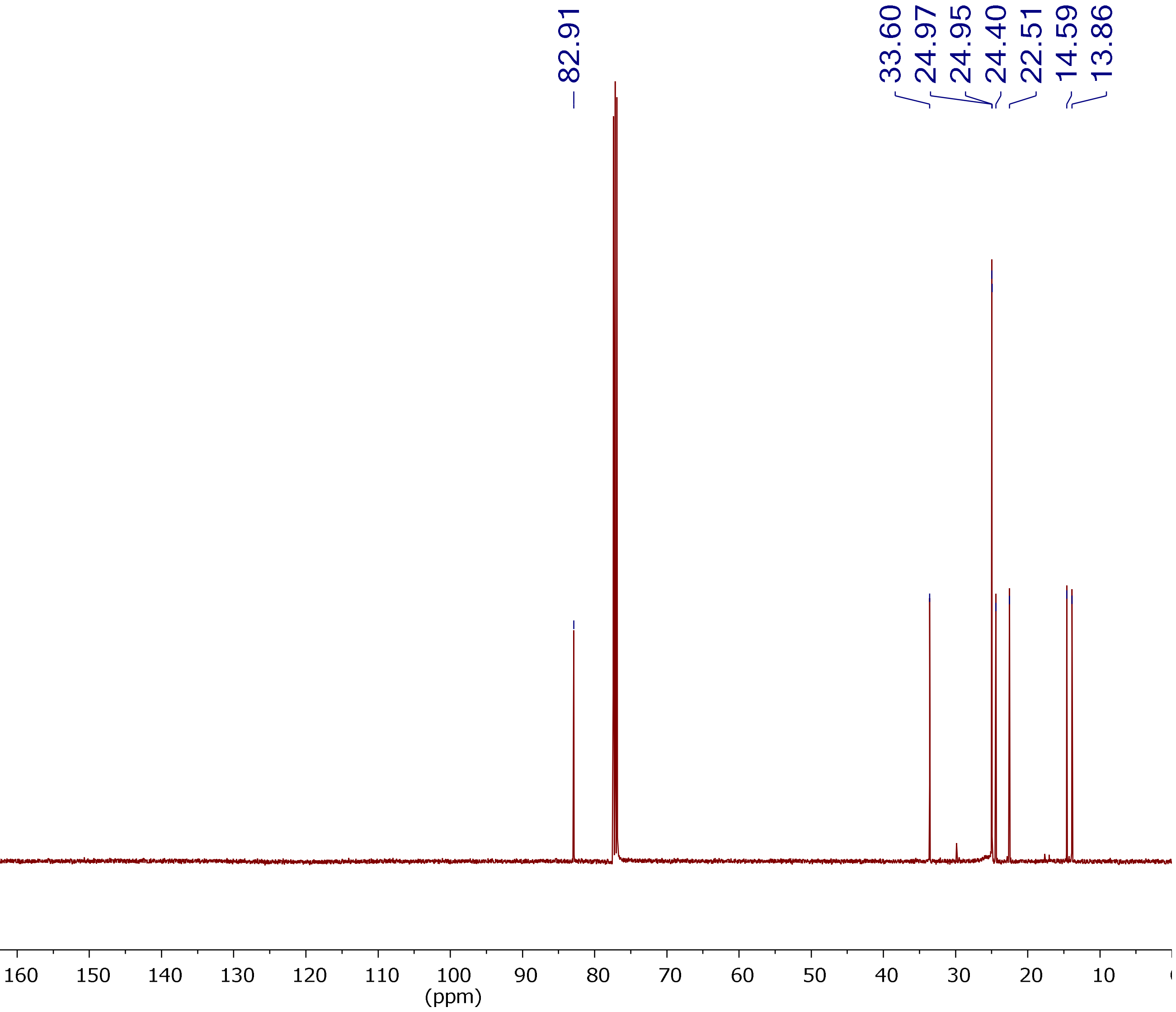

Supplementary Figure 137| ^{13}C-NMR spectrum (126 MHz, $CDCl_3$) for **(+)-SI-18**.

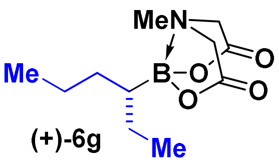

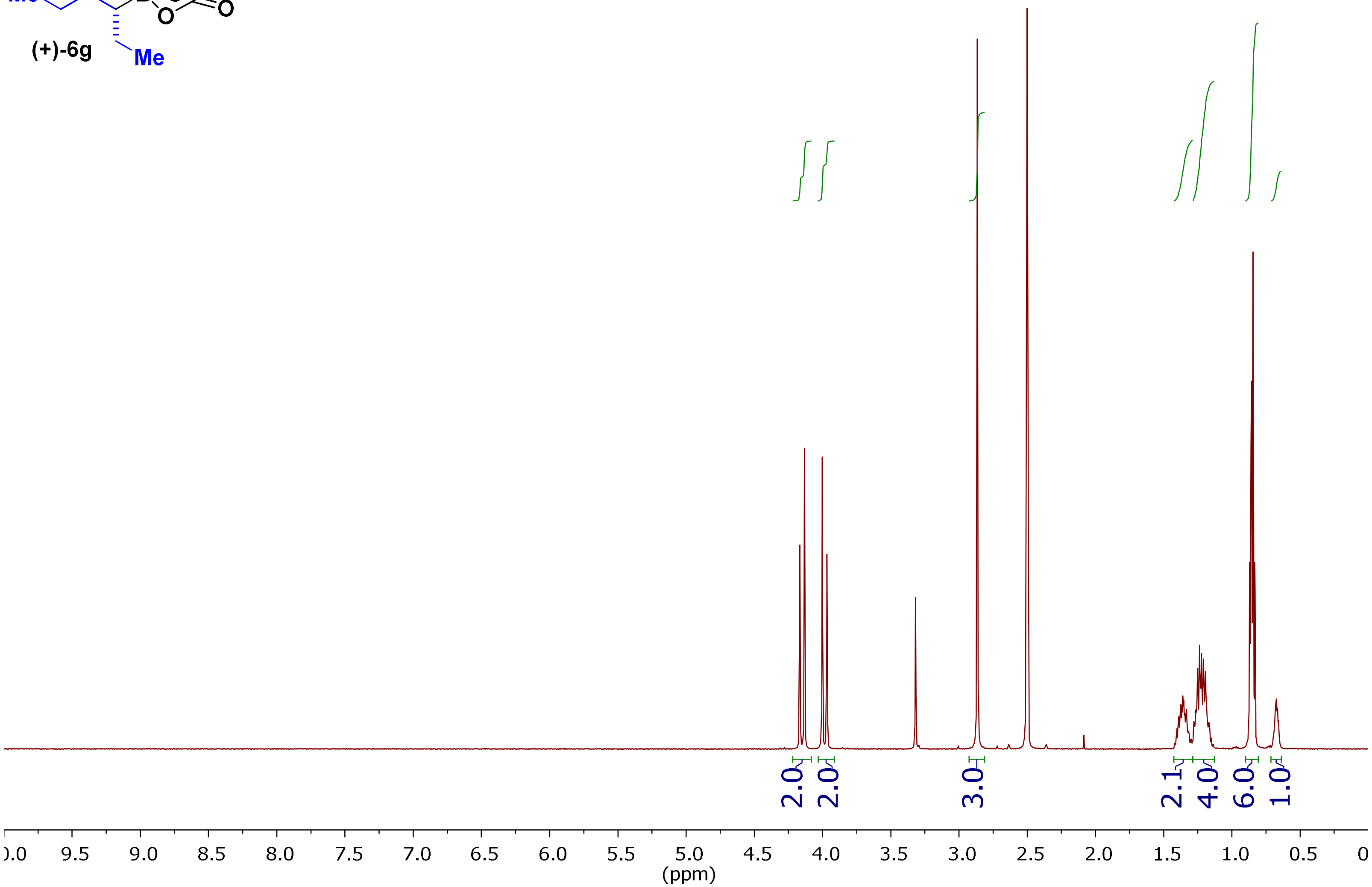

Supplementary Figure 138| ^{1}H-NMR spectrum (500 MHz, DMSO-*d6*) for **(+)-6g**.

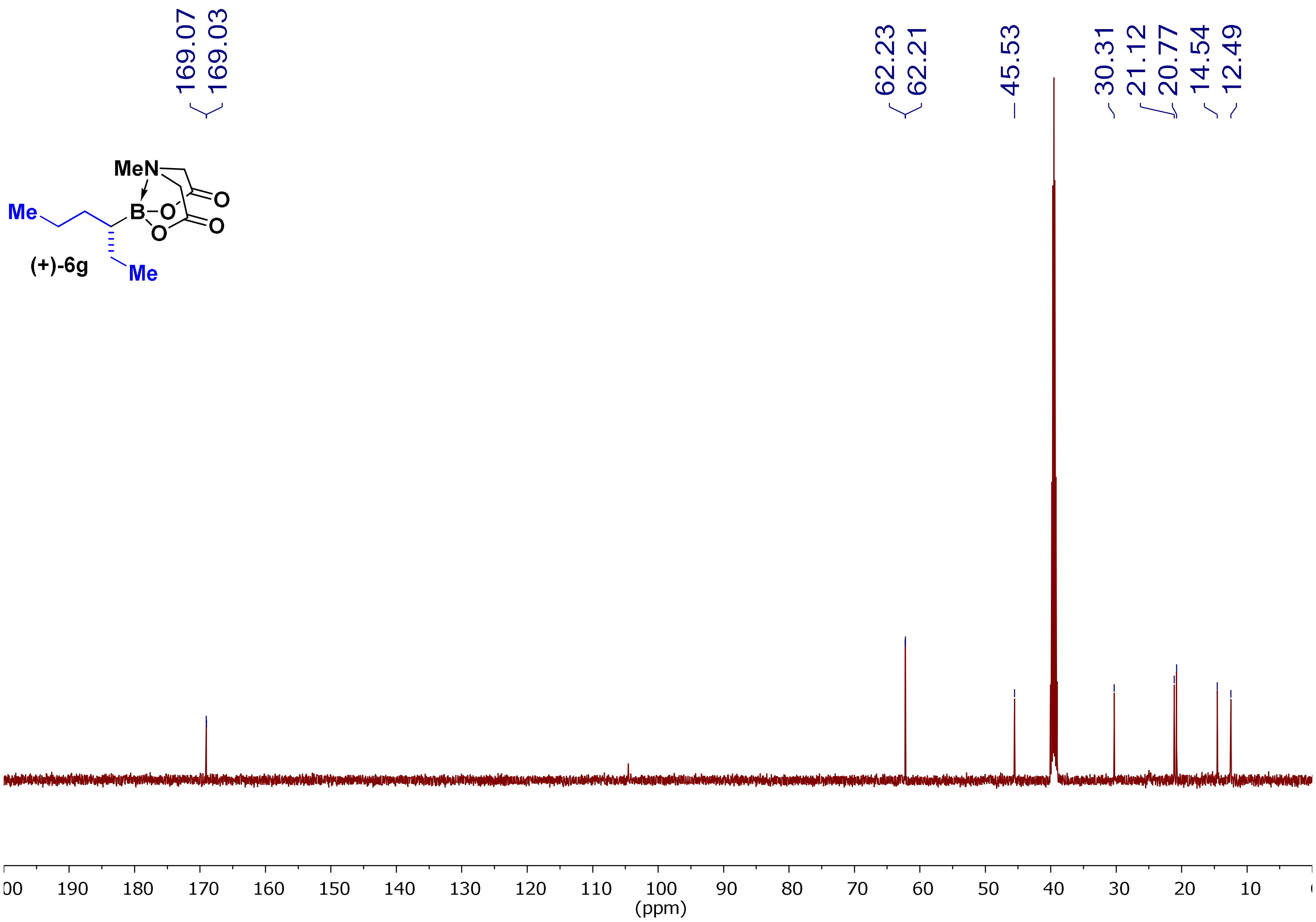

Supplementary Figure 139| ^{13}C-NMR spectrum (126 MHz, DMSO-*d6*) for **(+)-6g**.

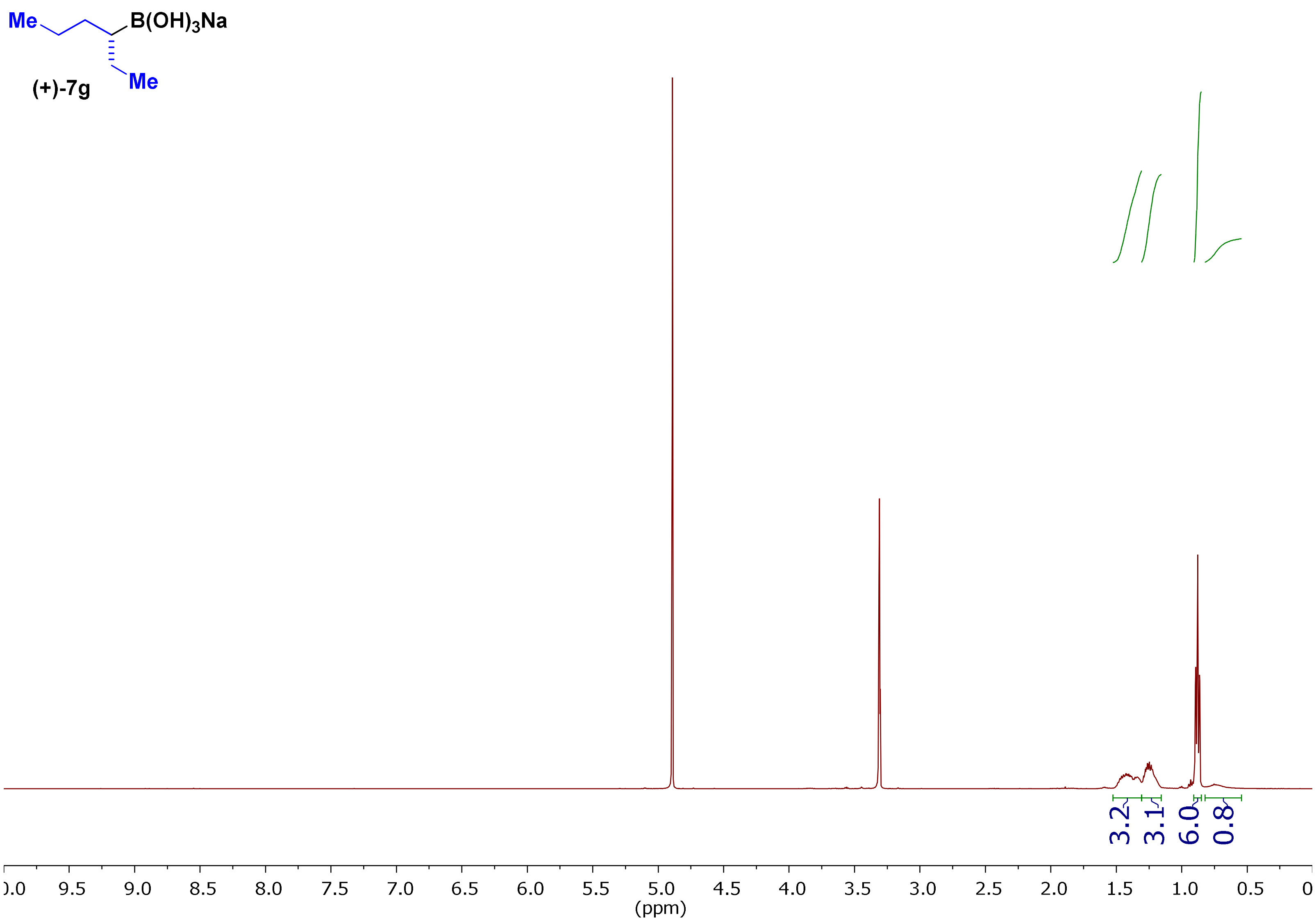

Supplementary Figure 140| ^{1}H-NMR spectrum (500 MHz, CD_3OD) for **(+)-7g**.

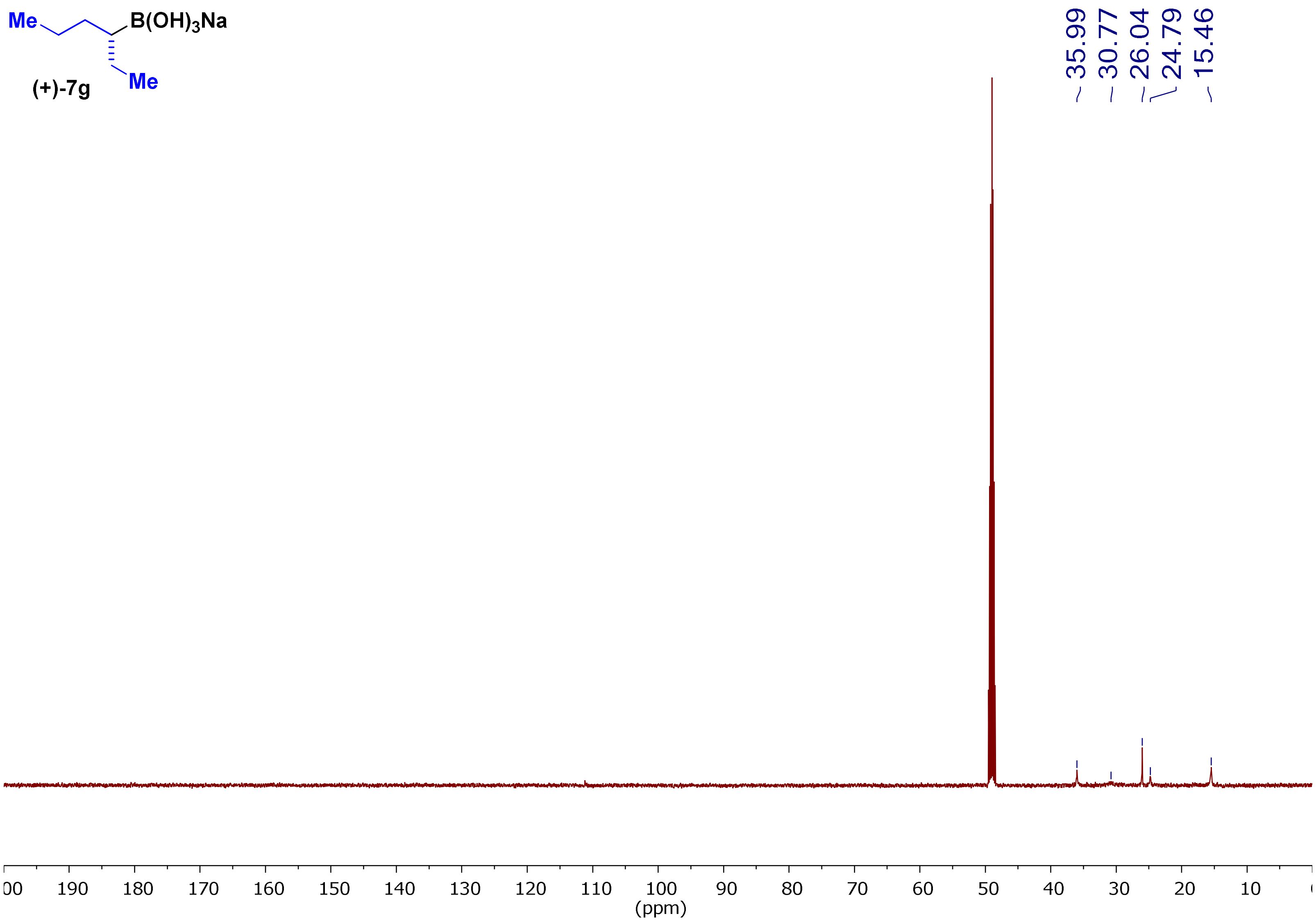

Supplementary Figure 141| ^{13}C-NMR spectrum (126 MHz, CD_3OD) for **(+)-7g**.

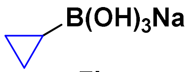

7h

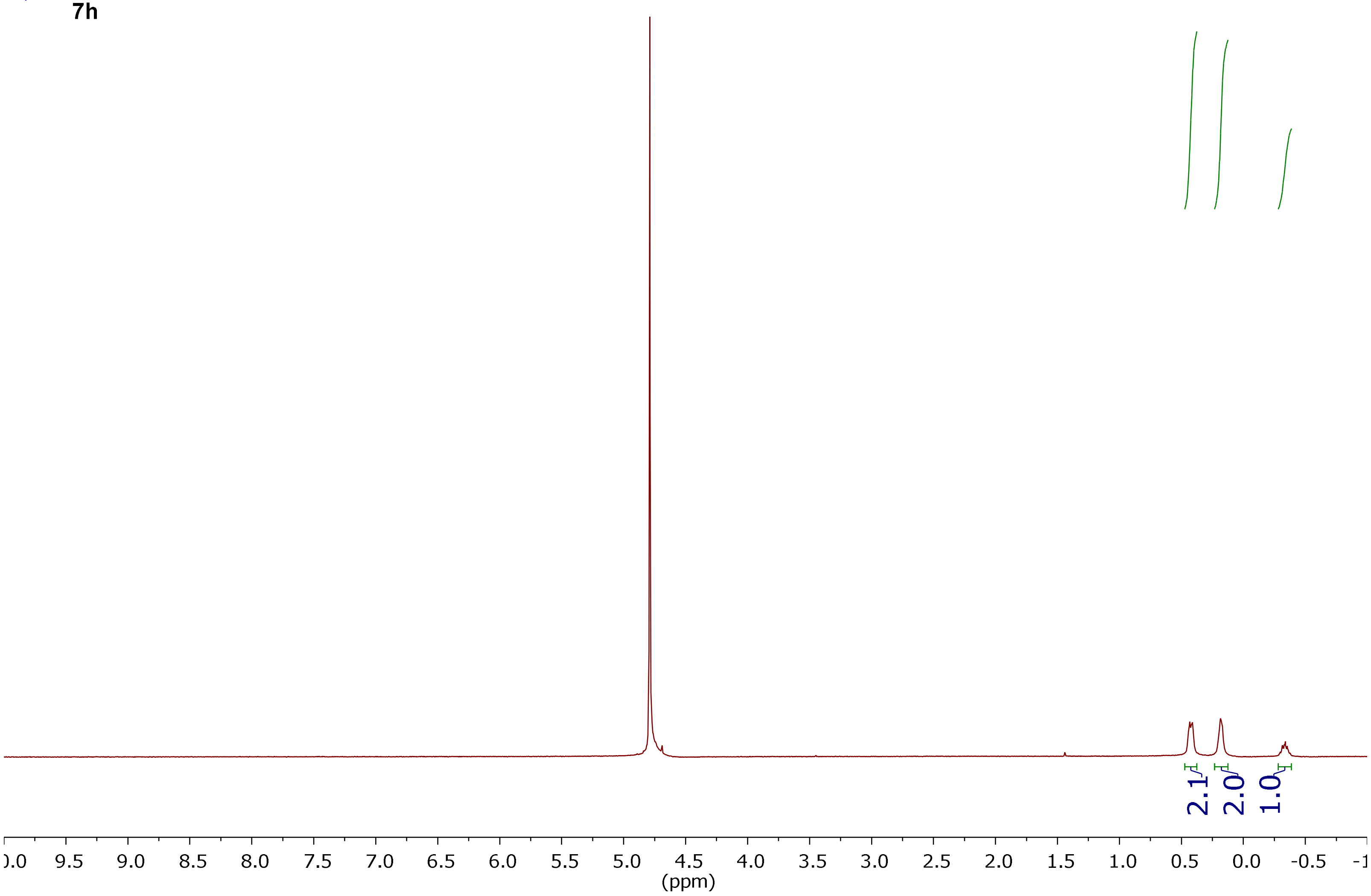

Supplementary Figure 142| ^{1}H-NMR spectrum (400 MHz, D_2O) for **7h**.

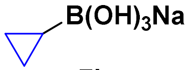

7h

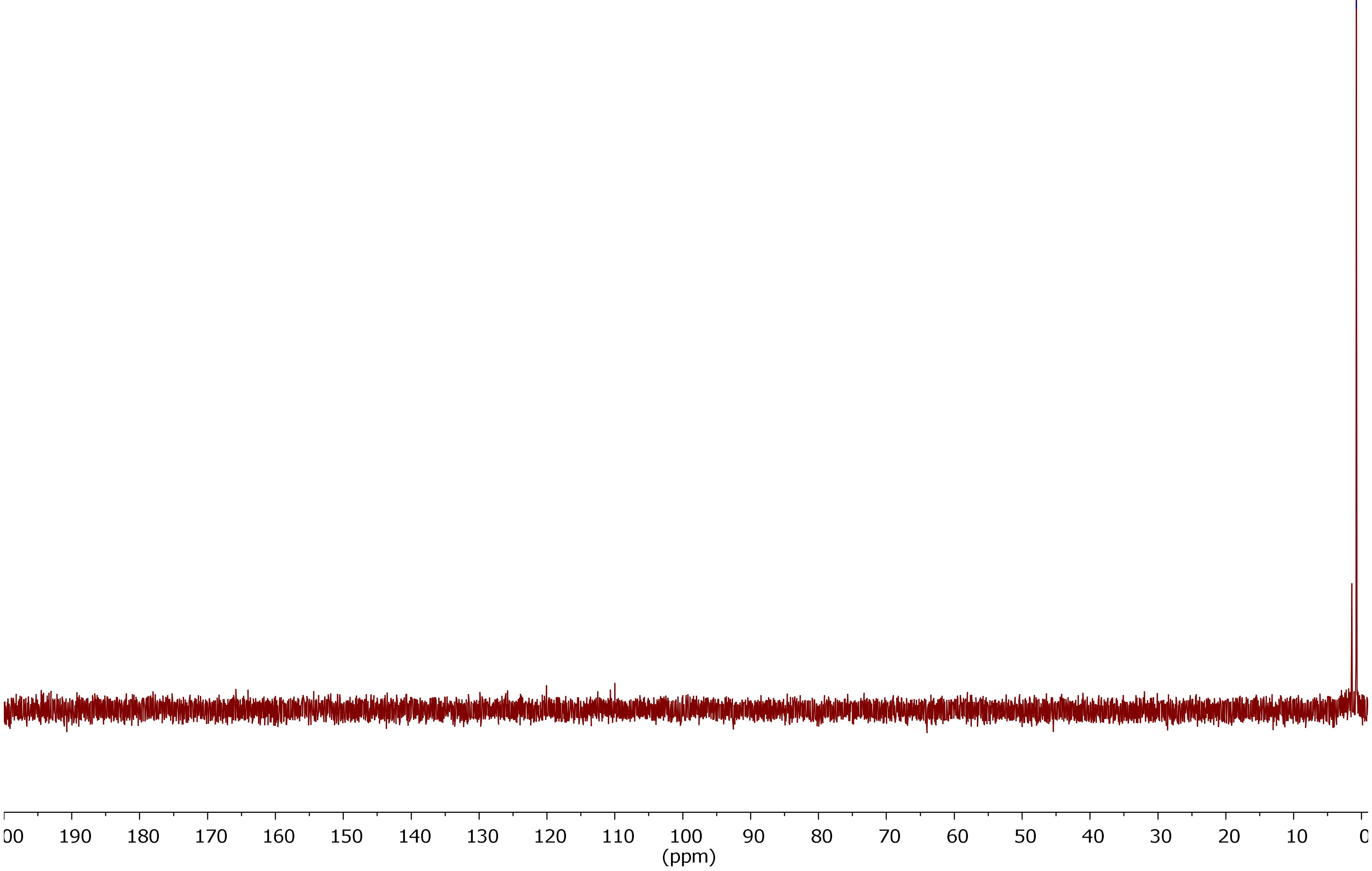

Supplementary Figure 143| ^{13}C-NMR spectrum (126 MHz, D_2O) for **7h**.

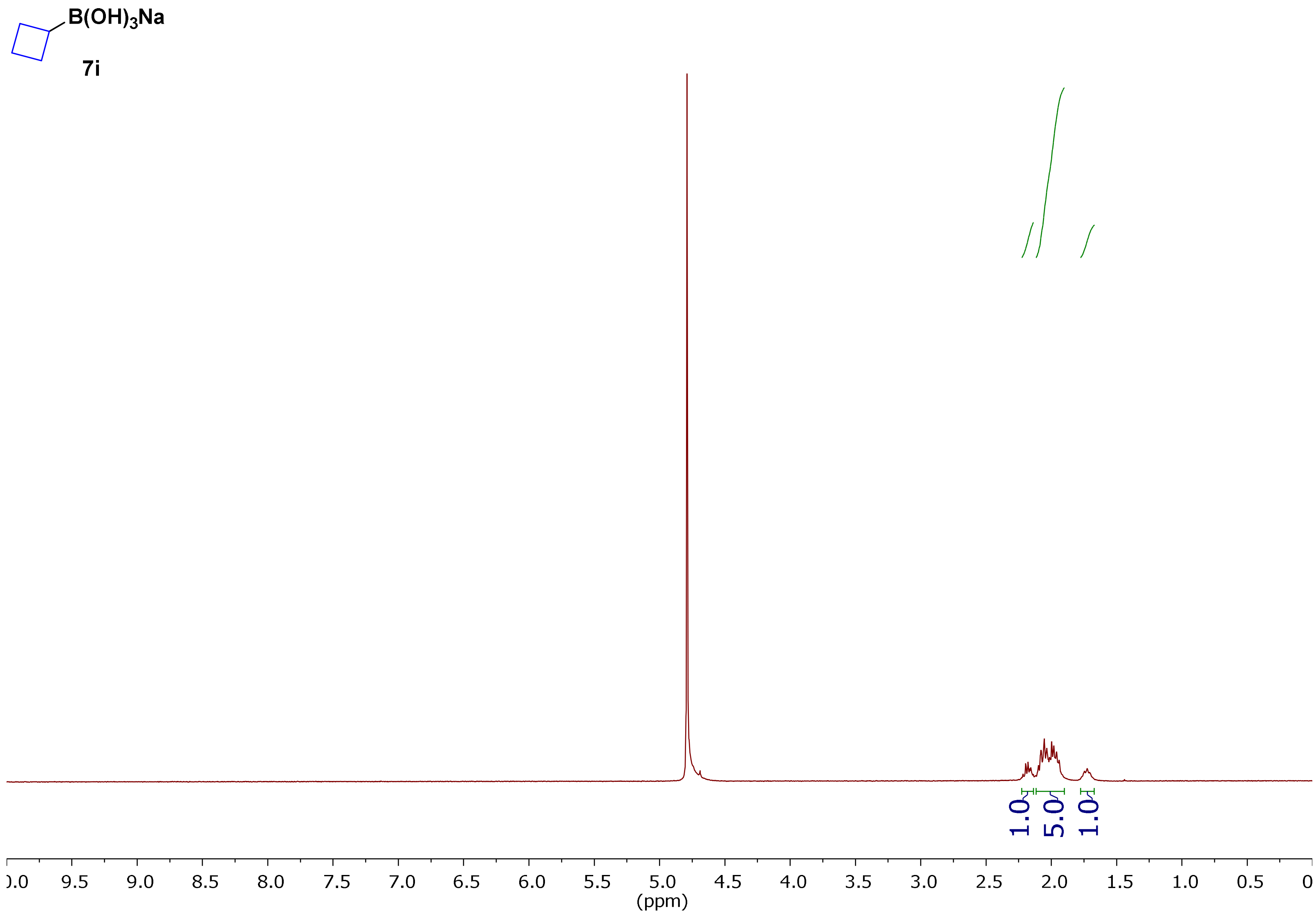

Supplementary Figure 144| ^{1}H-NMR spectrum (400 MHz, D_2O) for **7i**.

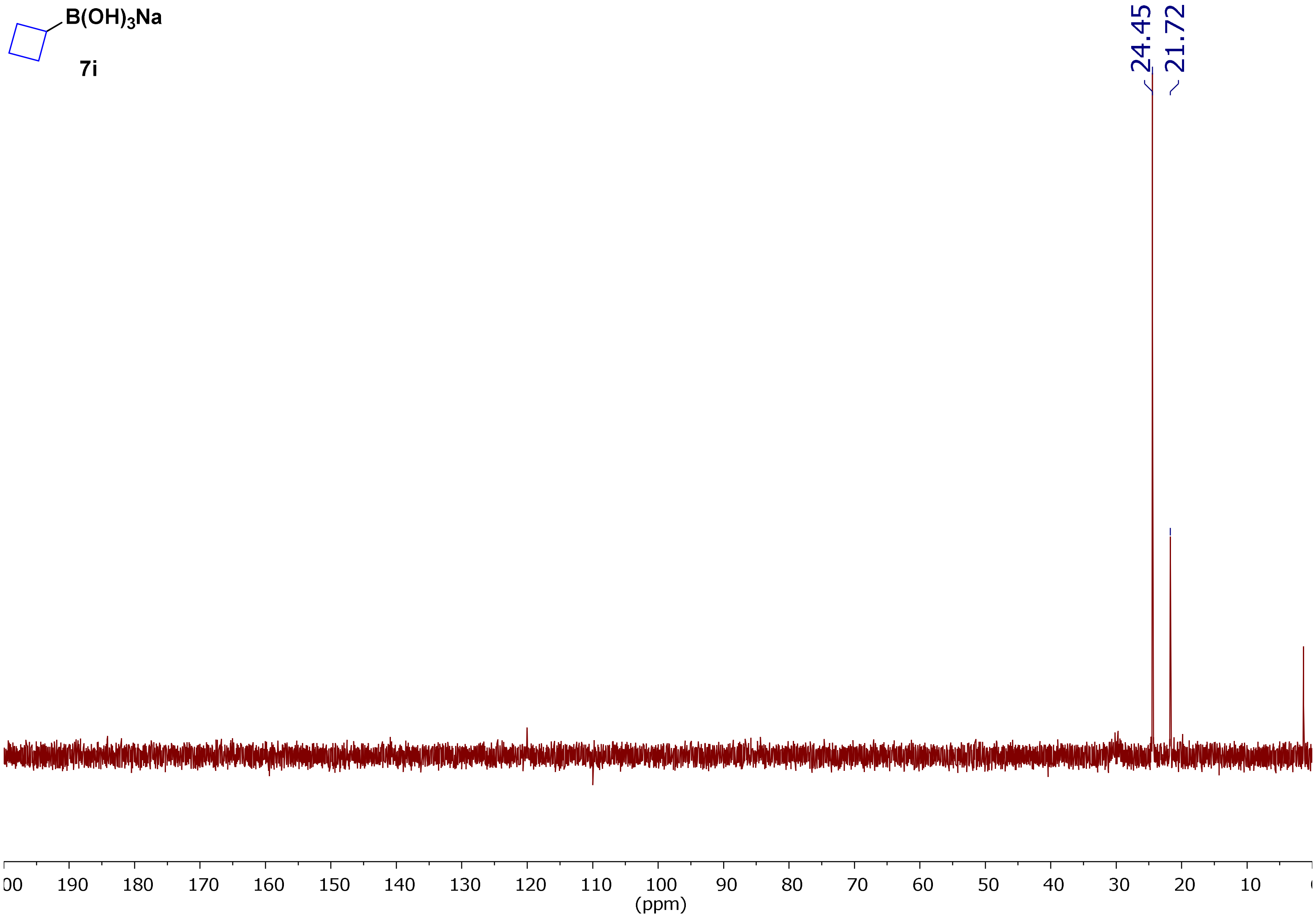

Supplementary Figure 145| [13]H-NMR spectrum (126 MHz, D_2O) for **7i**.

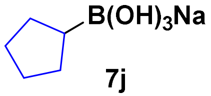

7j

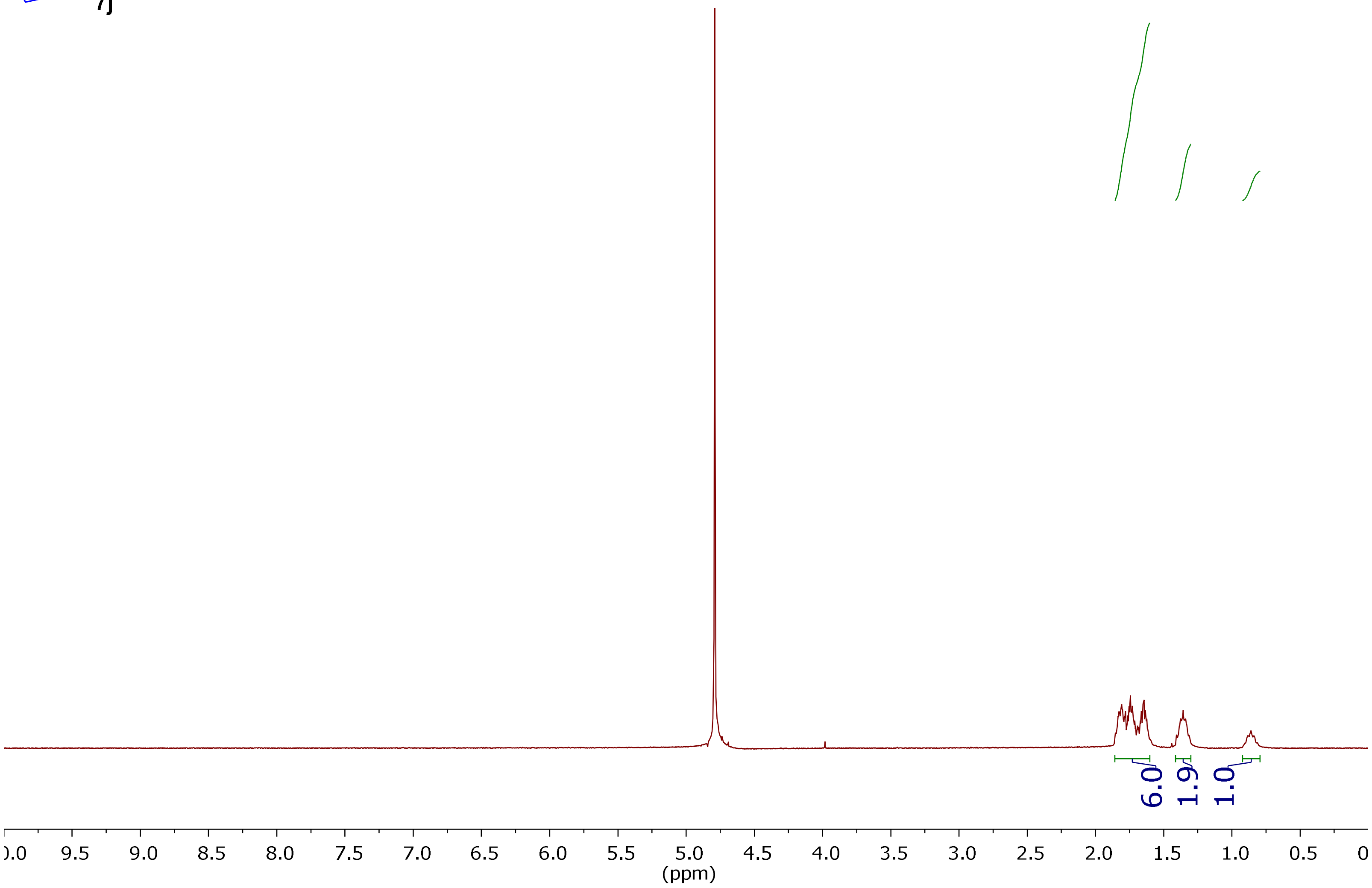

Supplementary Figure 146| ^{1}H-NMR spectrum (400 MHz, D_2O) for **7j**.

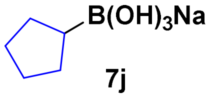

7j

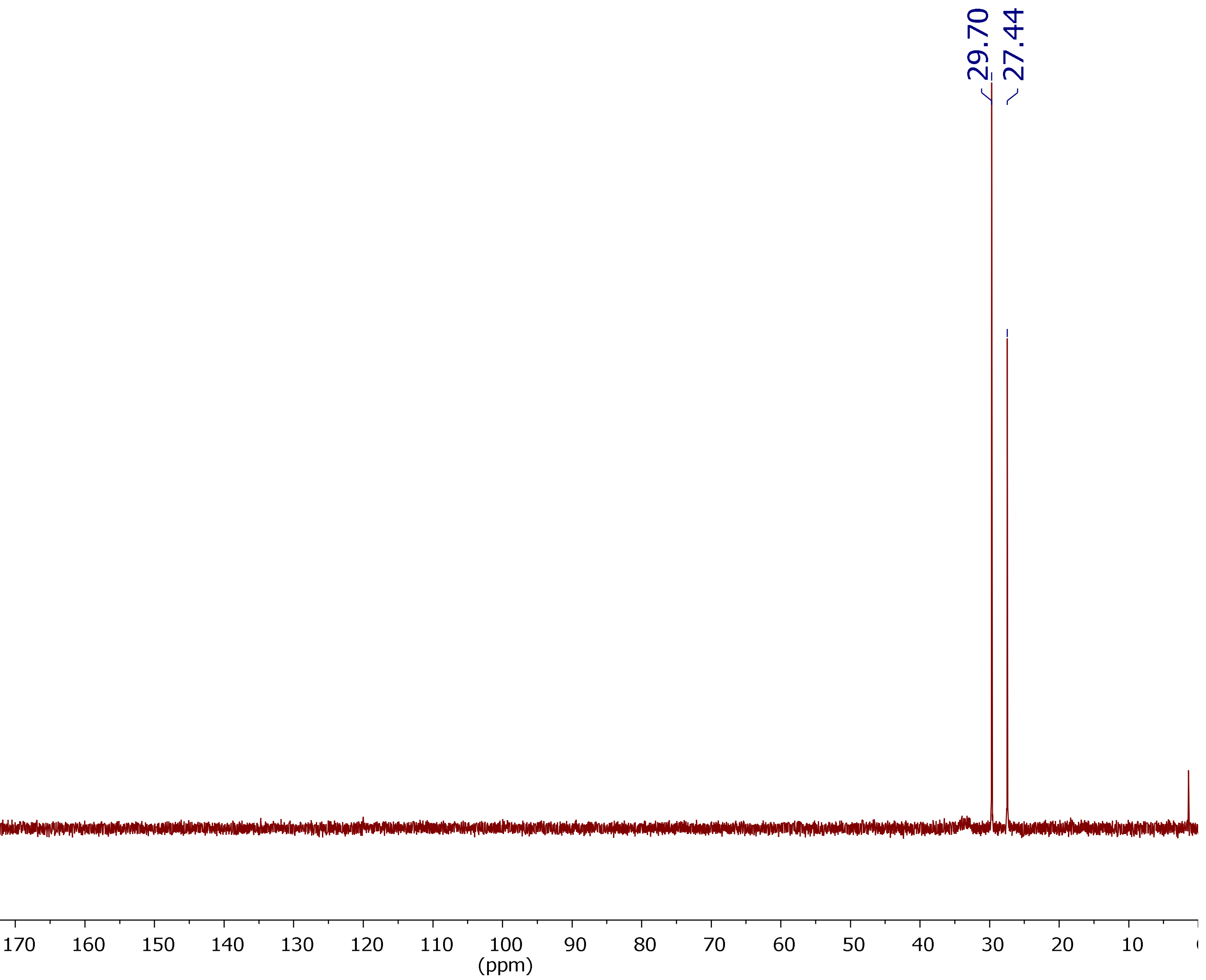

Supplementary Figure 147| ^{13}C-NMR spectrum (126 MHz, D_2O) for **7j**.

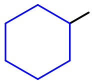

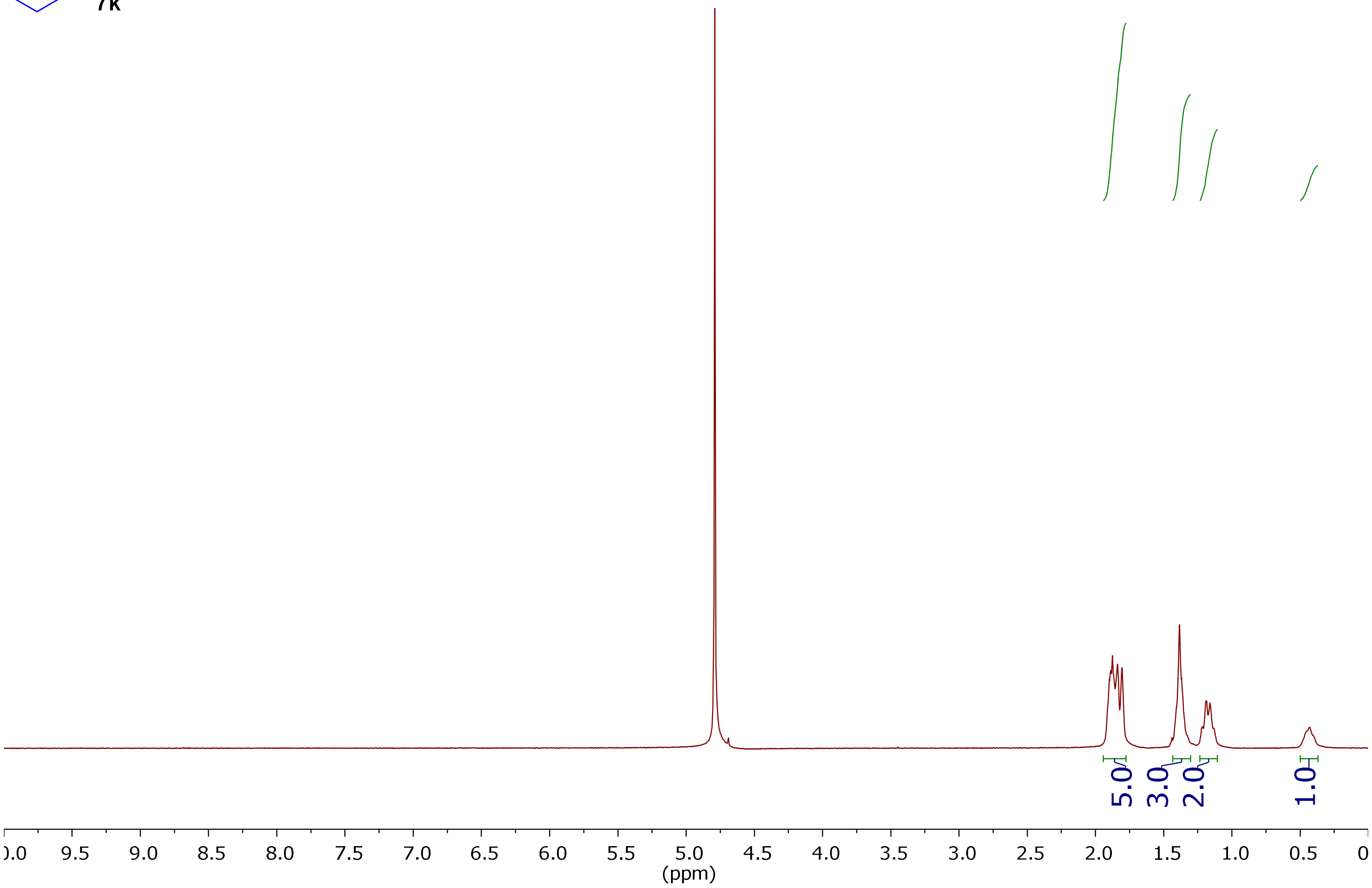

Supplementary Figure 148| ^{1}H-NMR spectrum (400 MHz, D_2O) for **7k**.

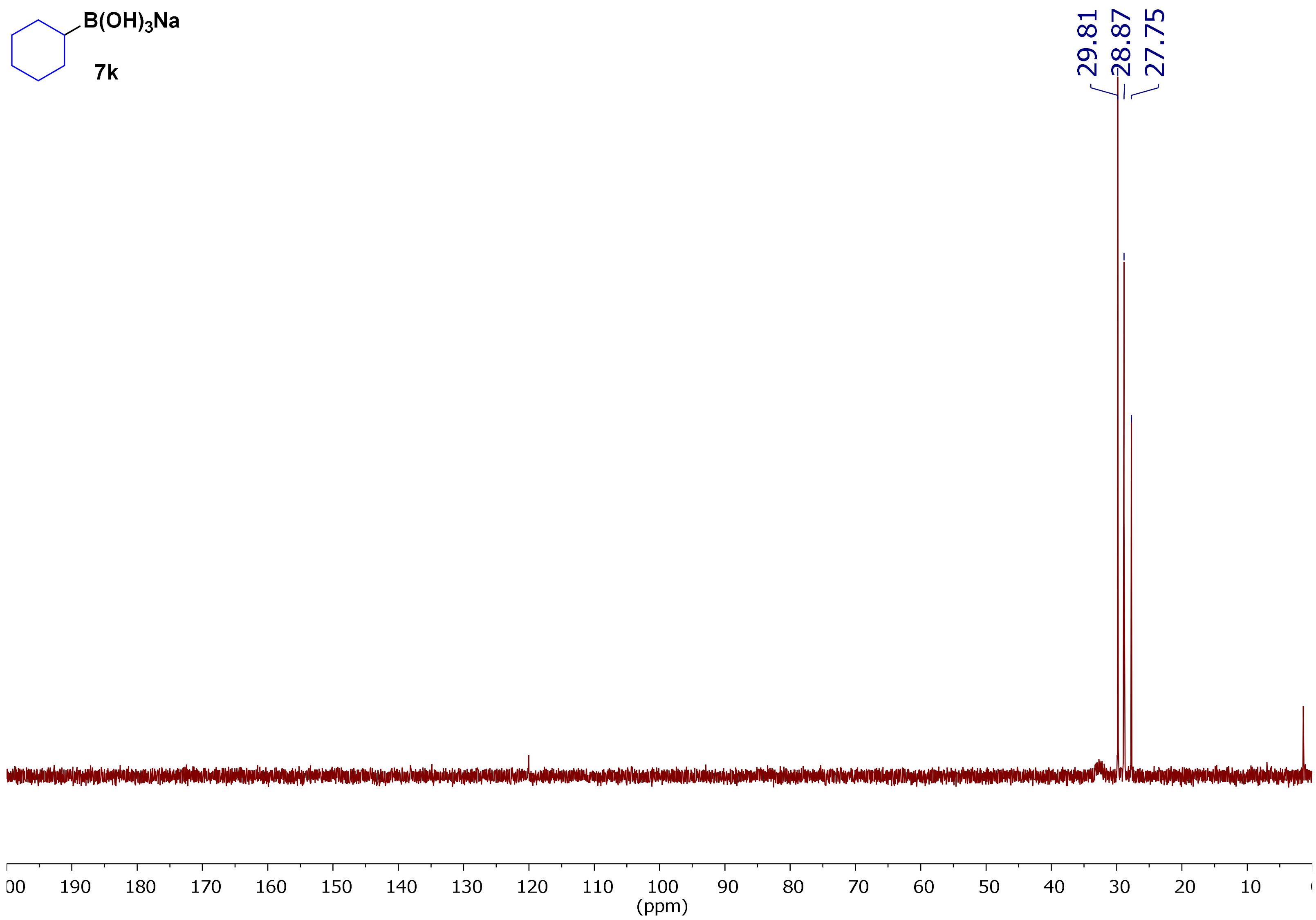

Supplementary Figure 149| ^{13}C-NMR spectrum (126 MHz, D_2O) for **7k**.

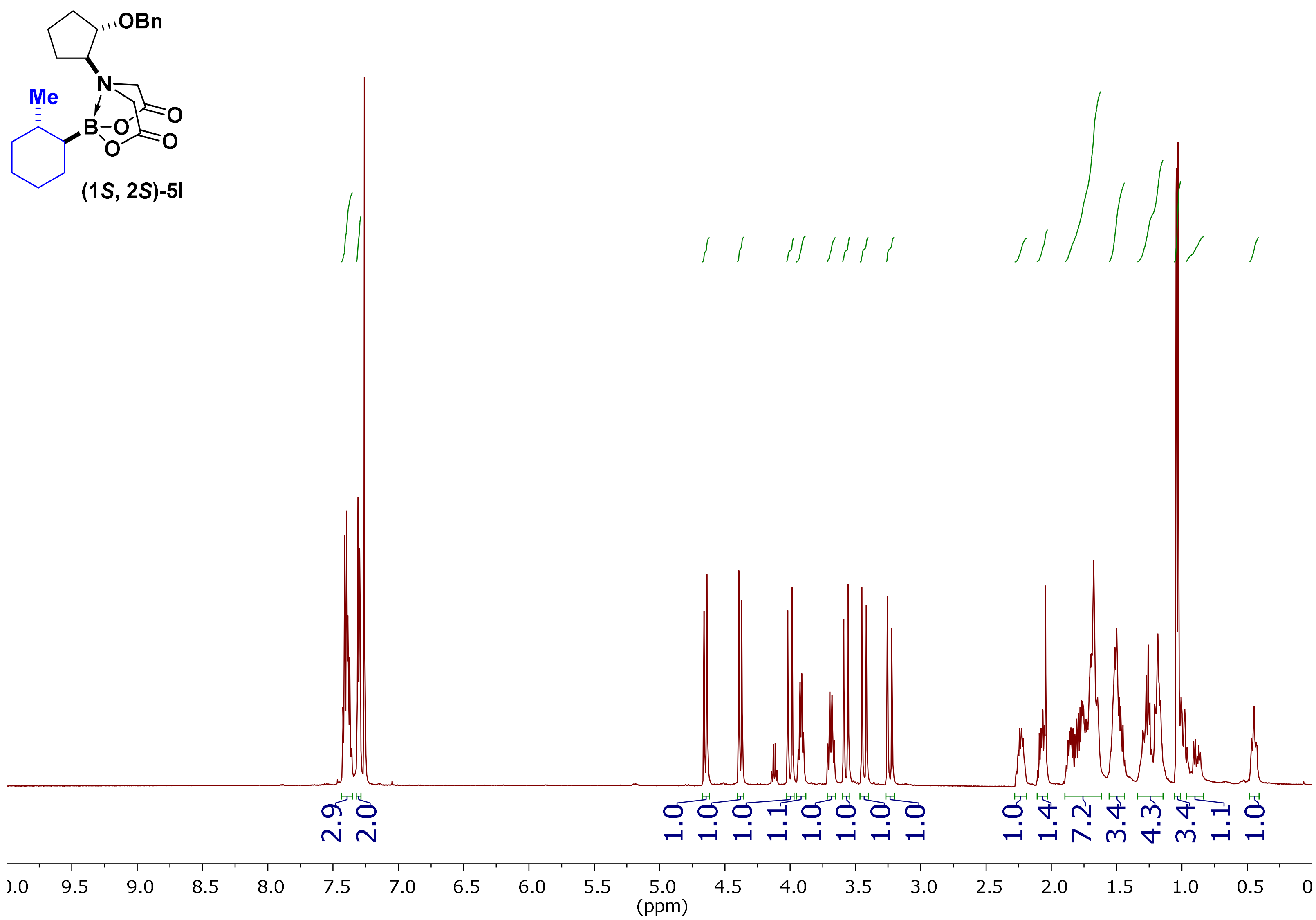

Supplementary Figure 150| ^{1}H-NMR spectrum (500 MHz, $CDCl_3$) for **(1*S*, 2*S*)-5l**.

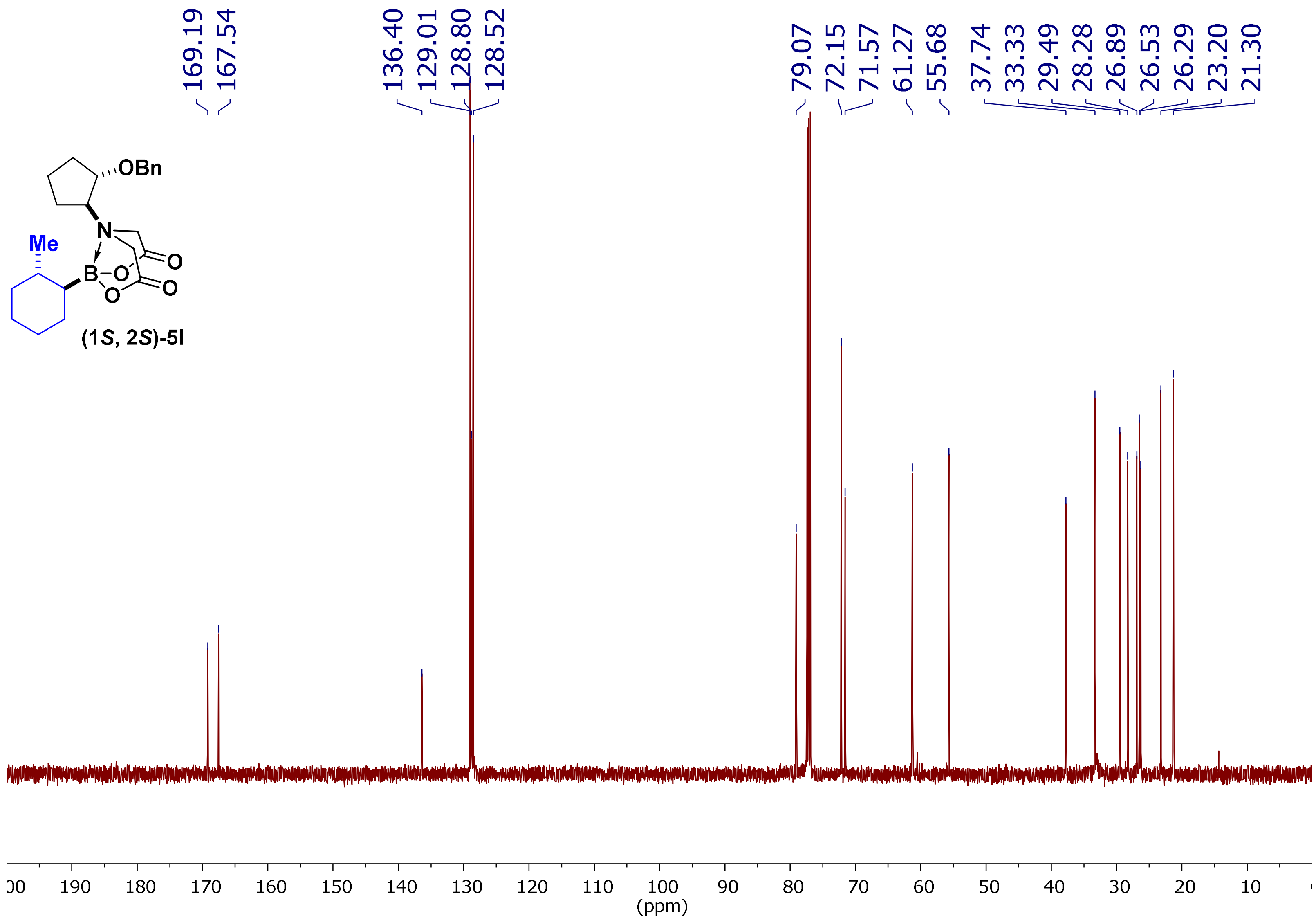

Supplementary Figure 151| ^{13}C-NMR spectrum (126 MHz, $CDCl_3$) for **(1*S*, 2*S*)-5l**.

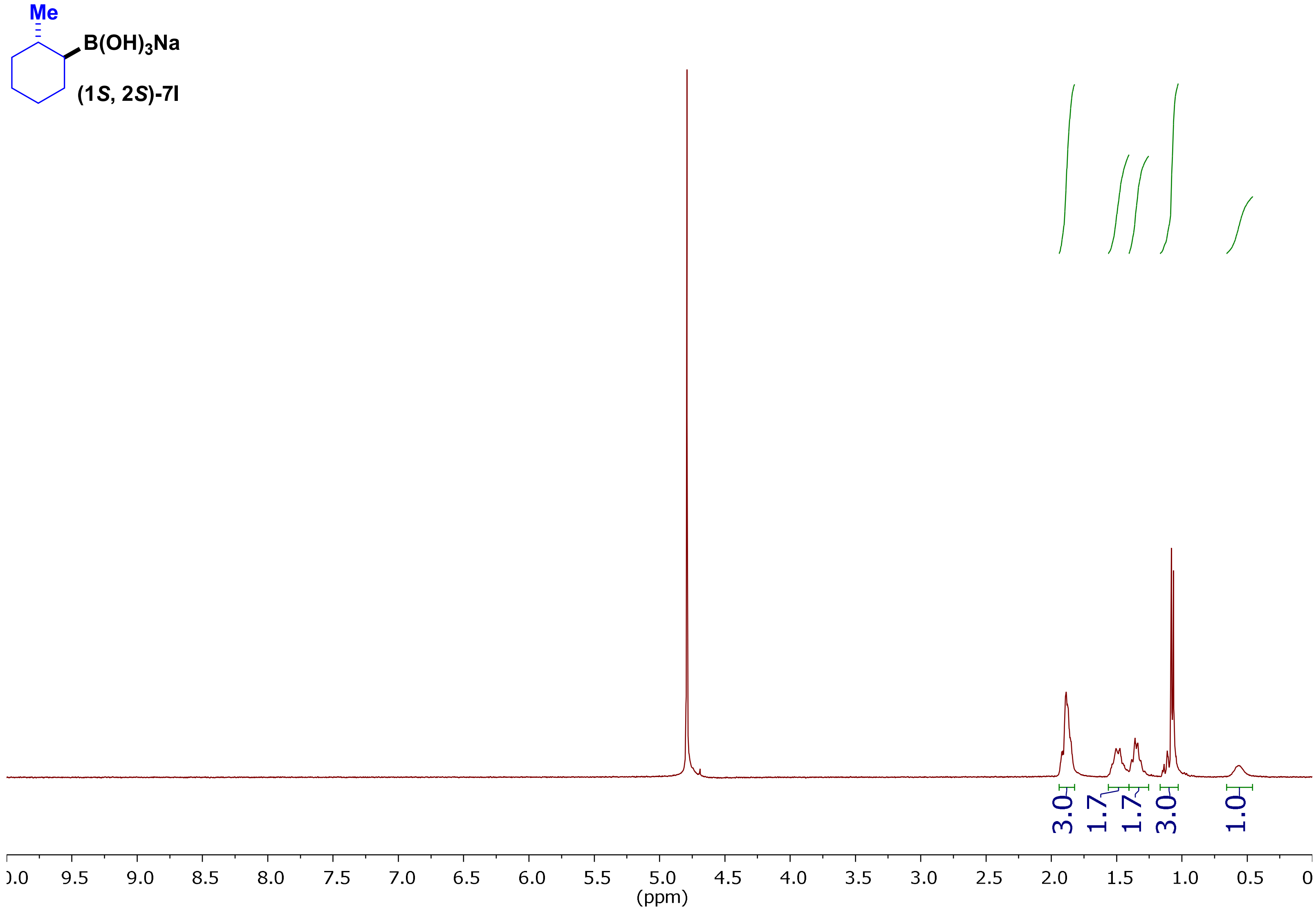

Supplementary Figure 152| ^{1}H-NMR spectrum (400 MHz, D_2O) for **(1*S*, 2*S*)-7l**.

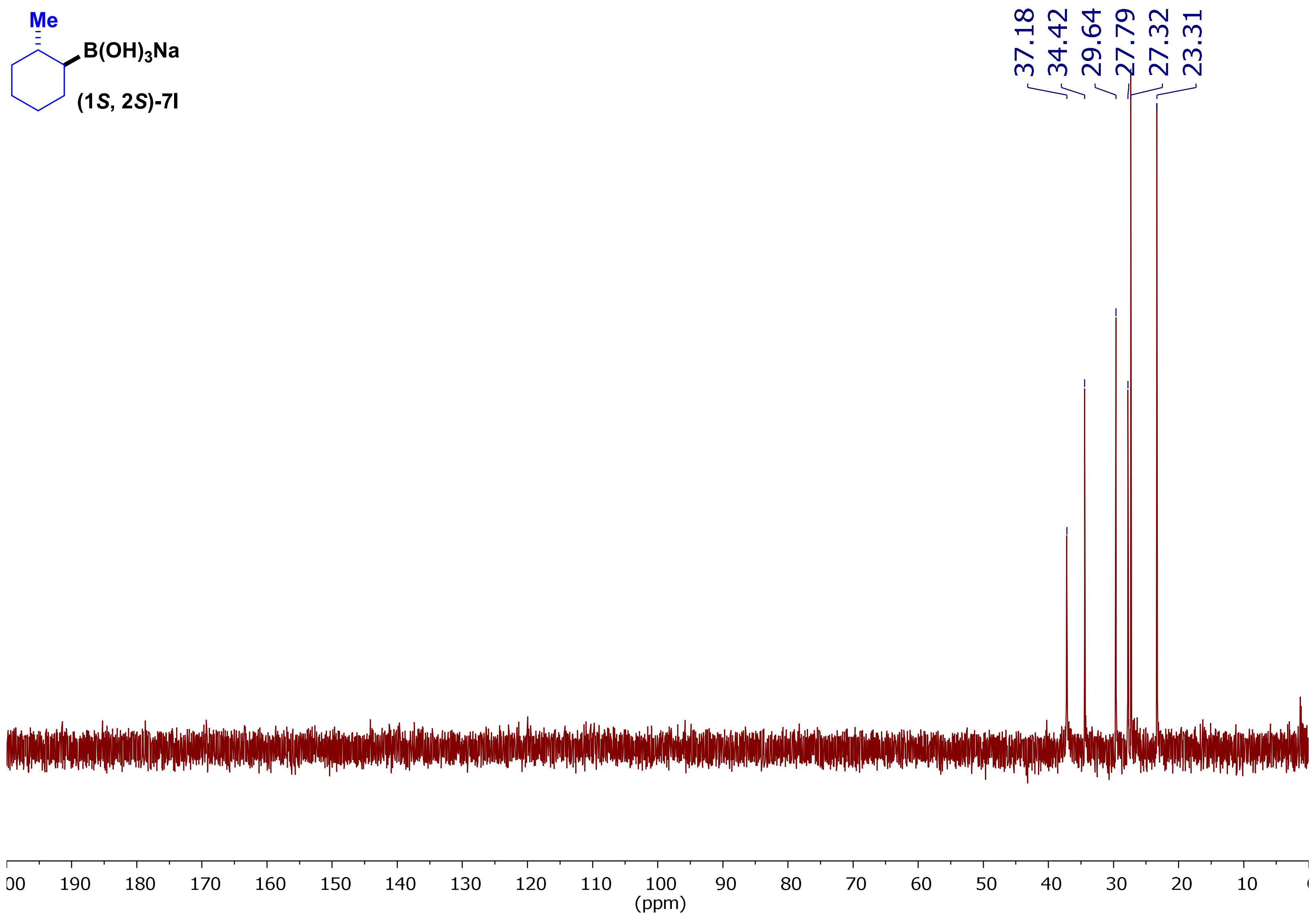

Supplementary Figure 153| ^{13}C-NMR spectrum (126 MHz, D_2O) for **(1*S*, 2*S*)-7l**.

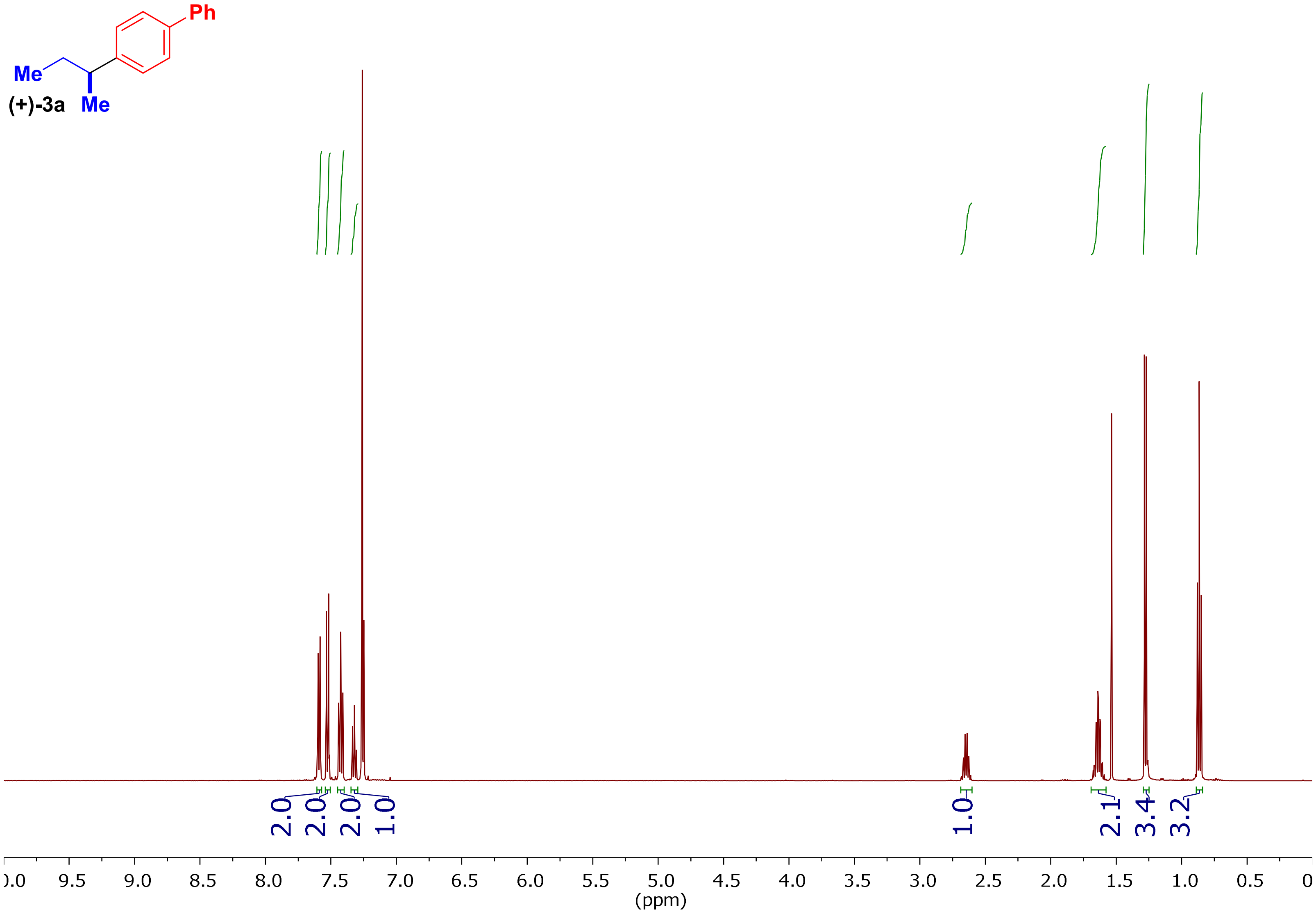

Supplementary Figure 154| ^{1}H-NMR spectrum (500 MHz, $CDCl_3$) for **(+)-3a**.

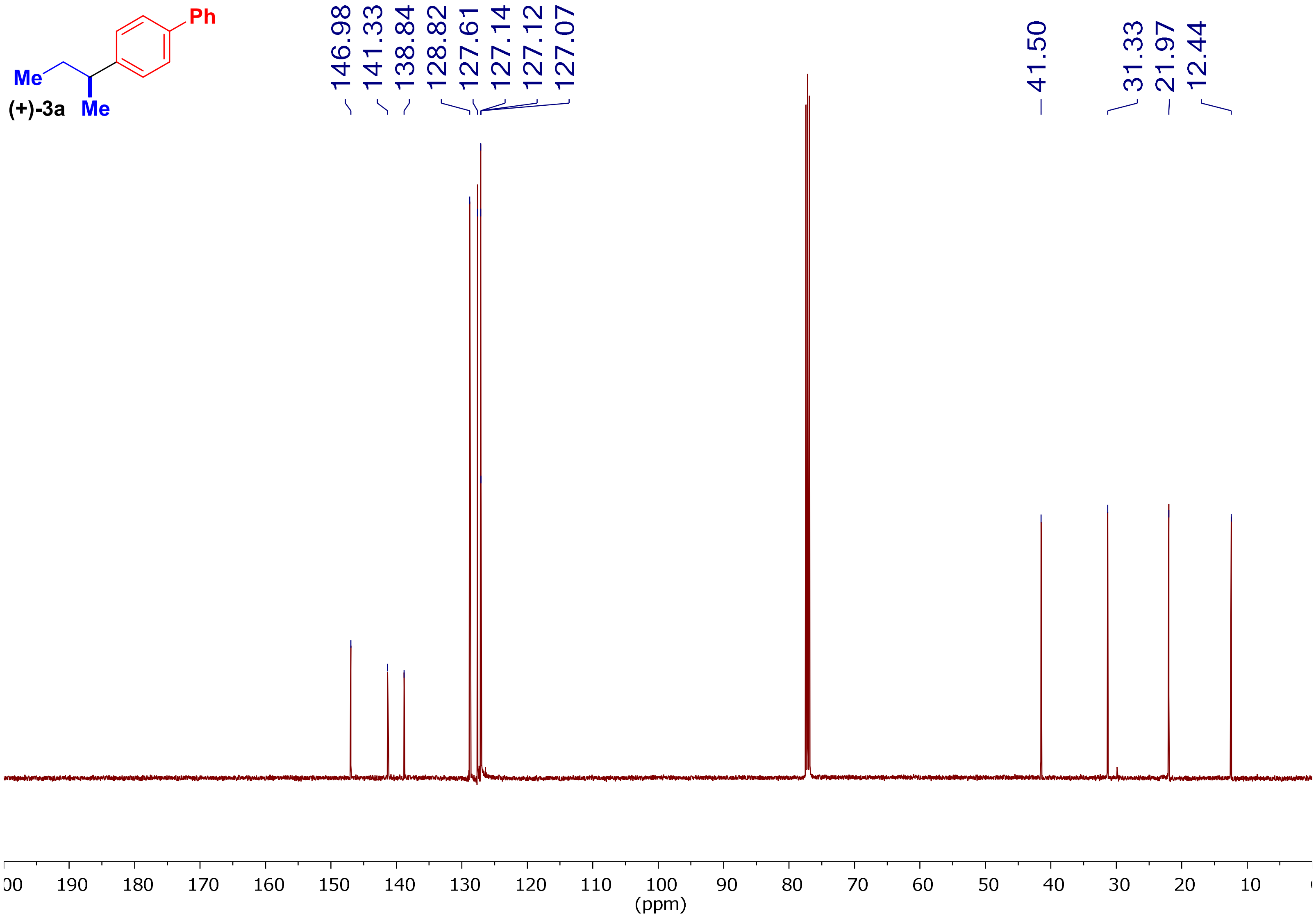

Supplementary Figure 155| ^{13}C-NMR spectrum (126 MHz, $CDCl_3$) for **(+)-3a**.

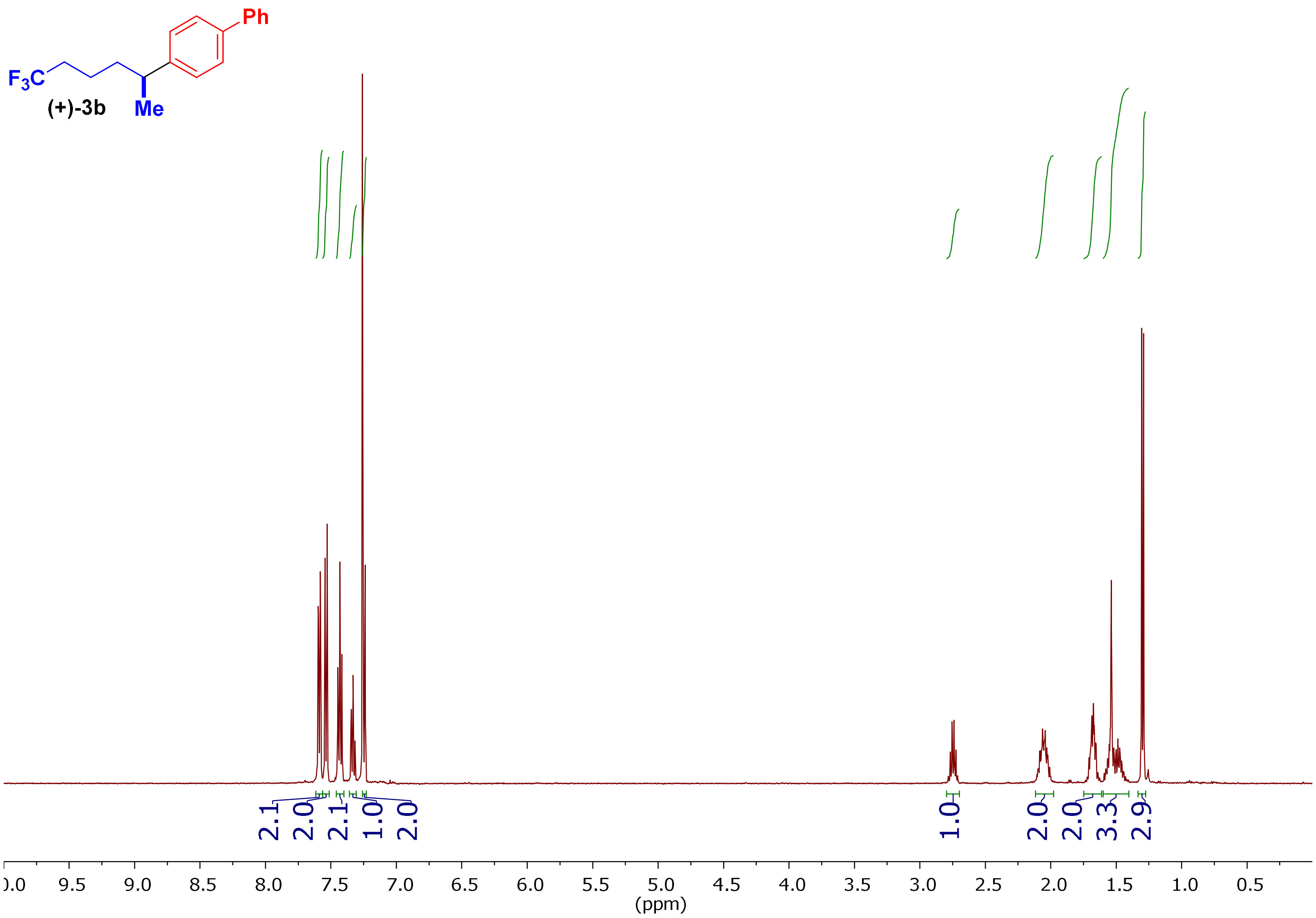

Supplementary Figure 156| ^{1}H-NMR spectrum (500 MHz, $CDCl_3$) for **(+)-3b**.

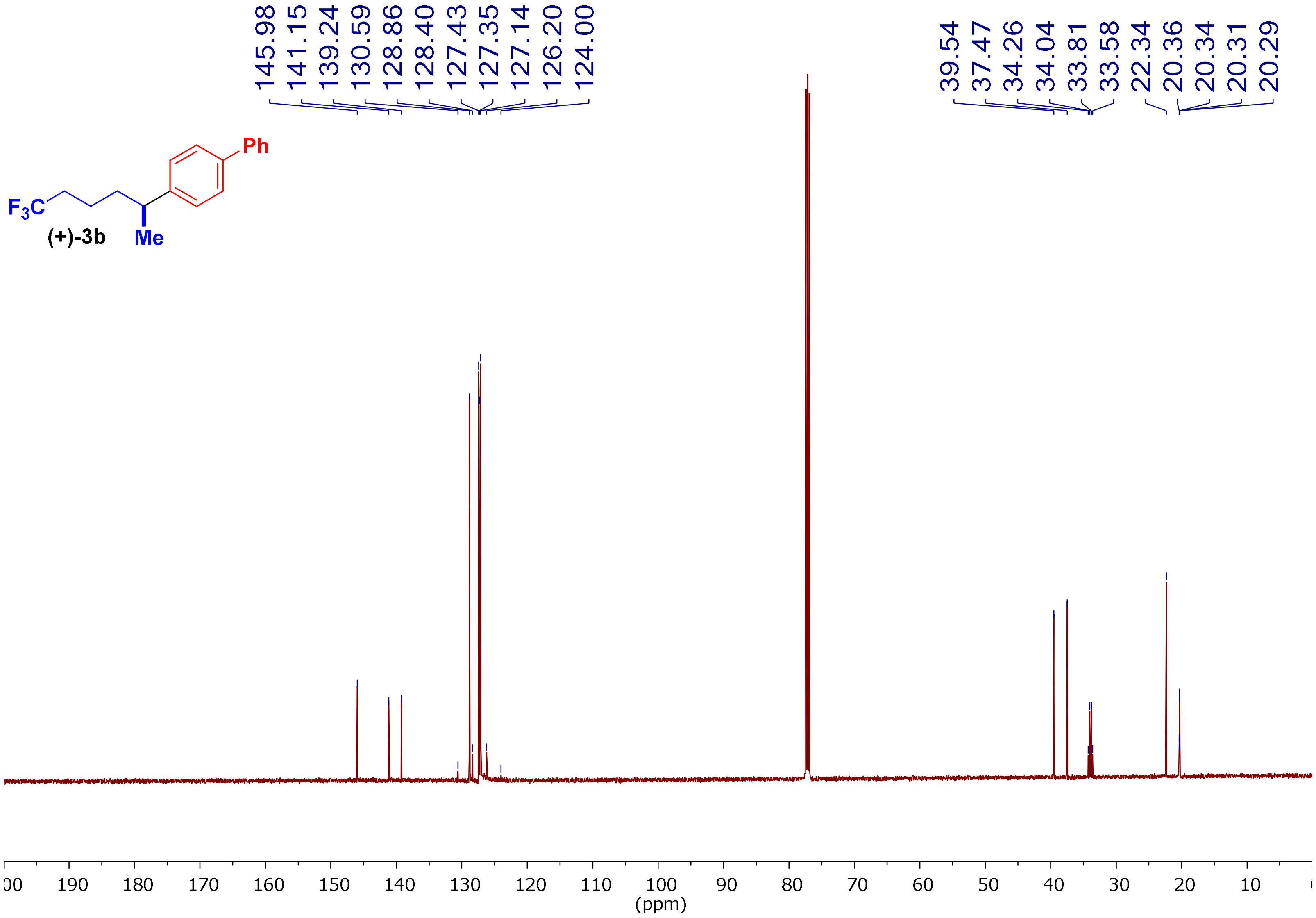

Supplementary Figure 157| ^{13}C-NMR spectrum (126 MHz, $CDCl_3$) for **(+)-3b**.

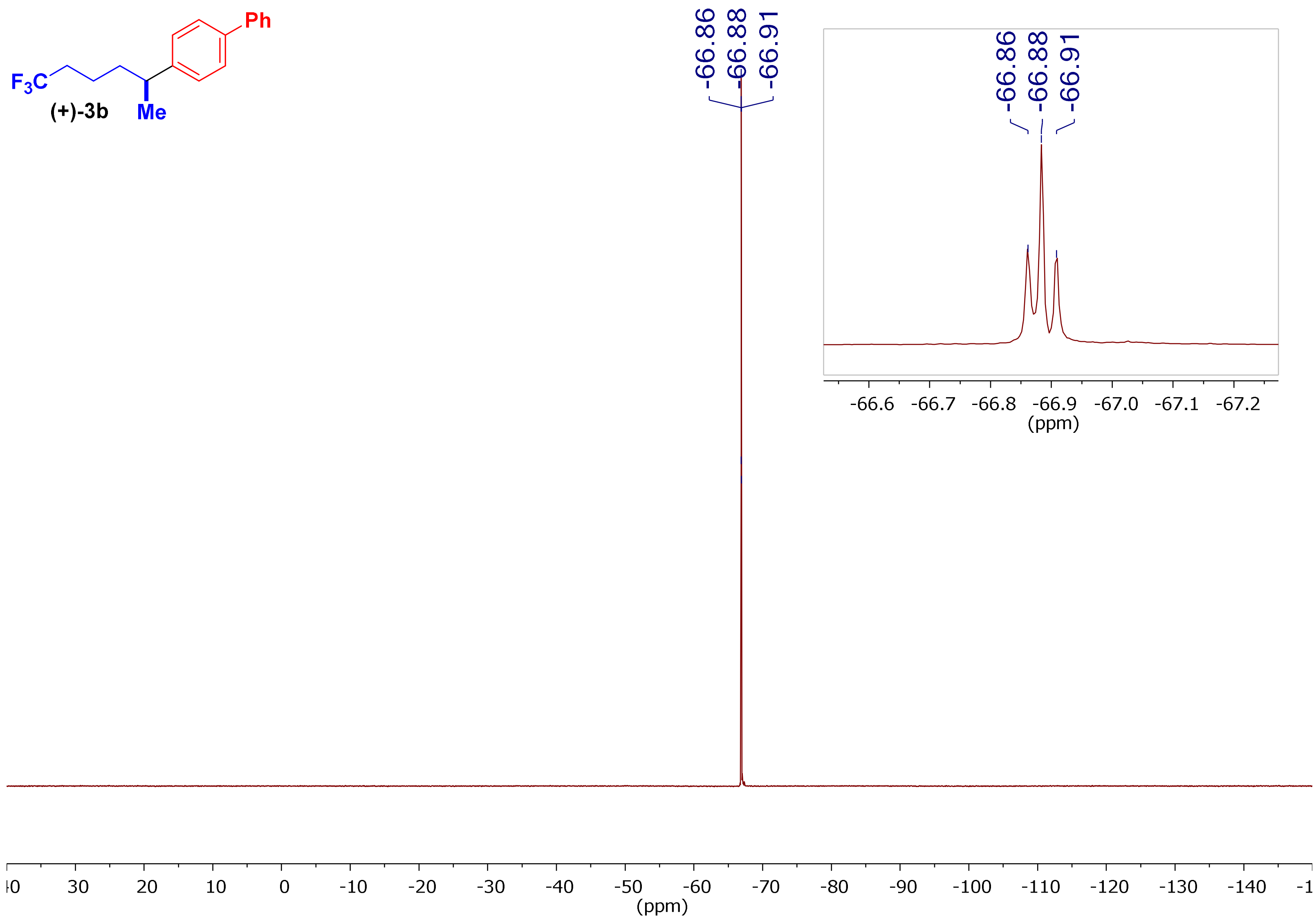

Supplementary Figure 158| ^{19}F-NMR spectrum (470 MHz, $CDCl_3$) for **(+)-3b**.

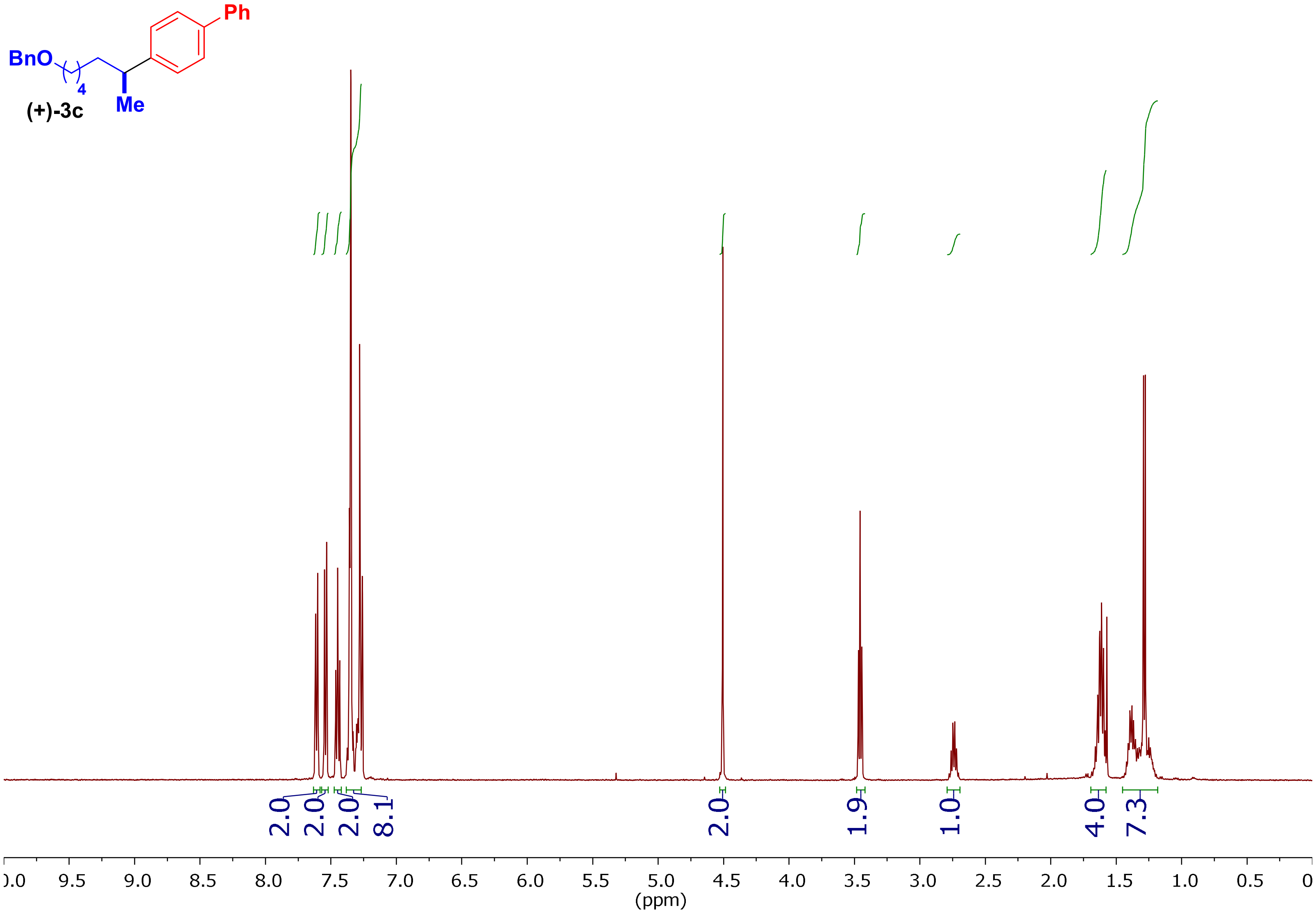

Supplementary Figure 159| ^{1}H-NMR spectrum (500 MHz, $CDCl_3$) for **(+)-3c**.

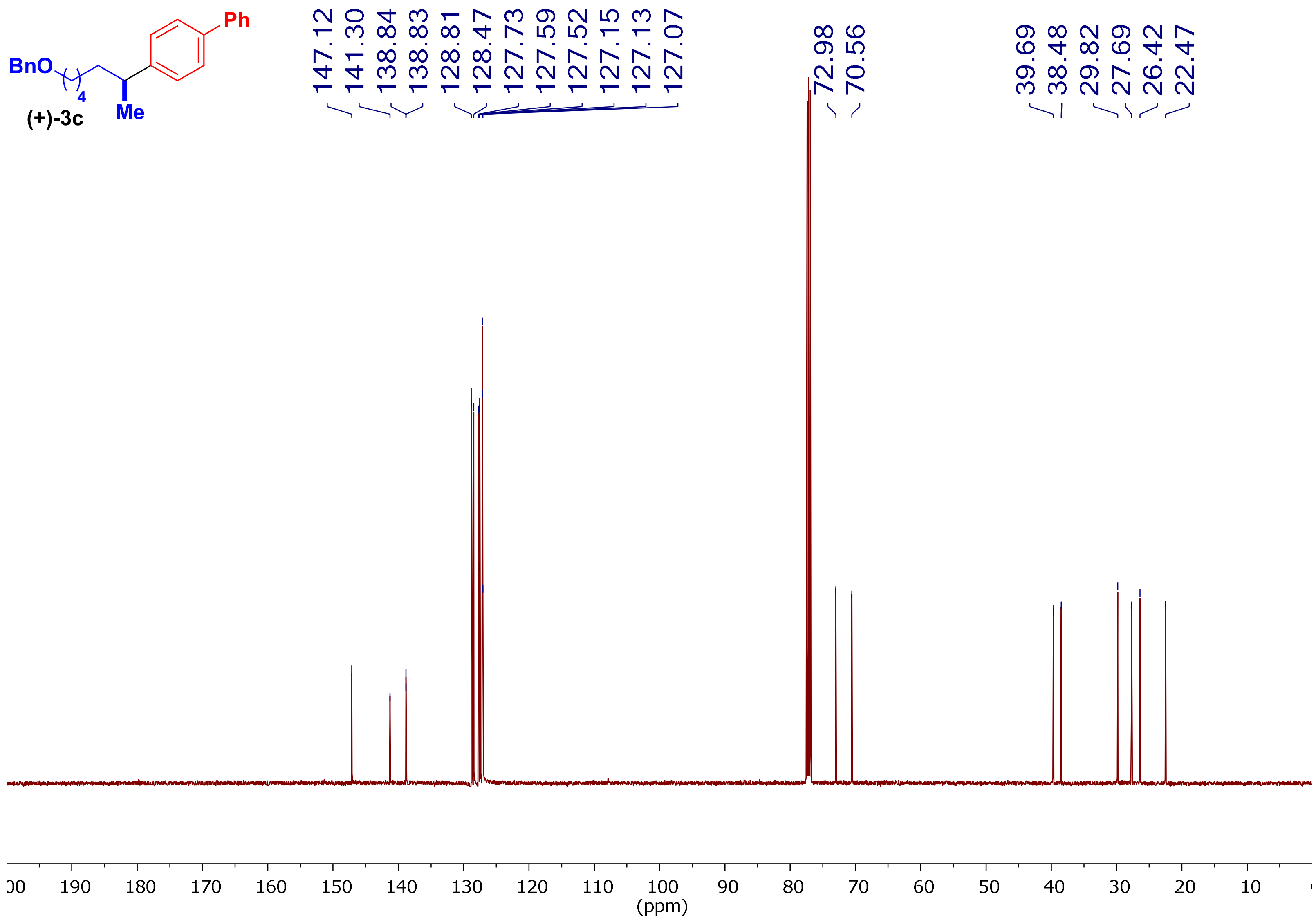

Supplementary Figure 160| ^{13}C-NMR spectrum (126 MHz, $CDCl_3$) for **(+)-3c**.

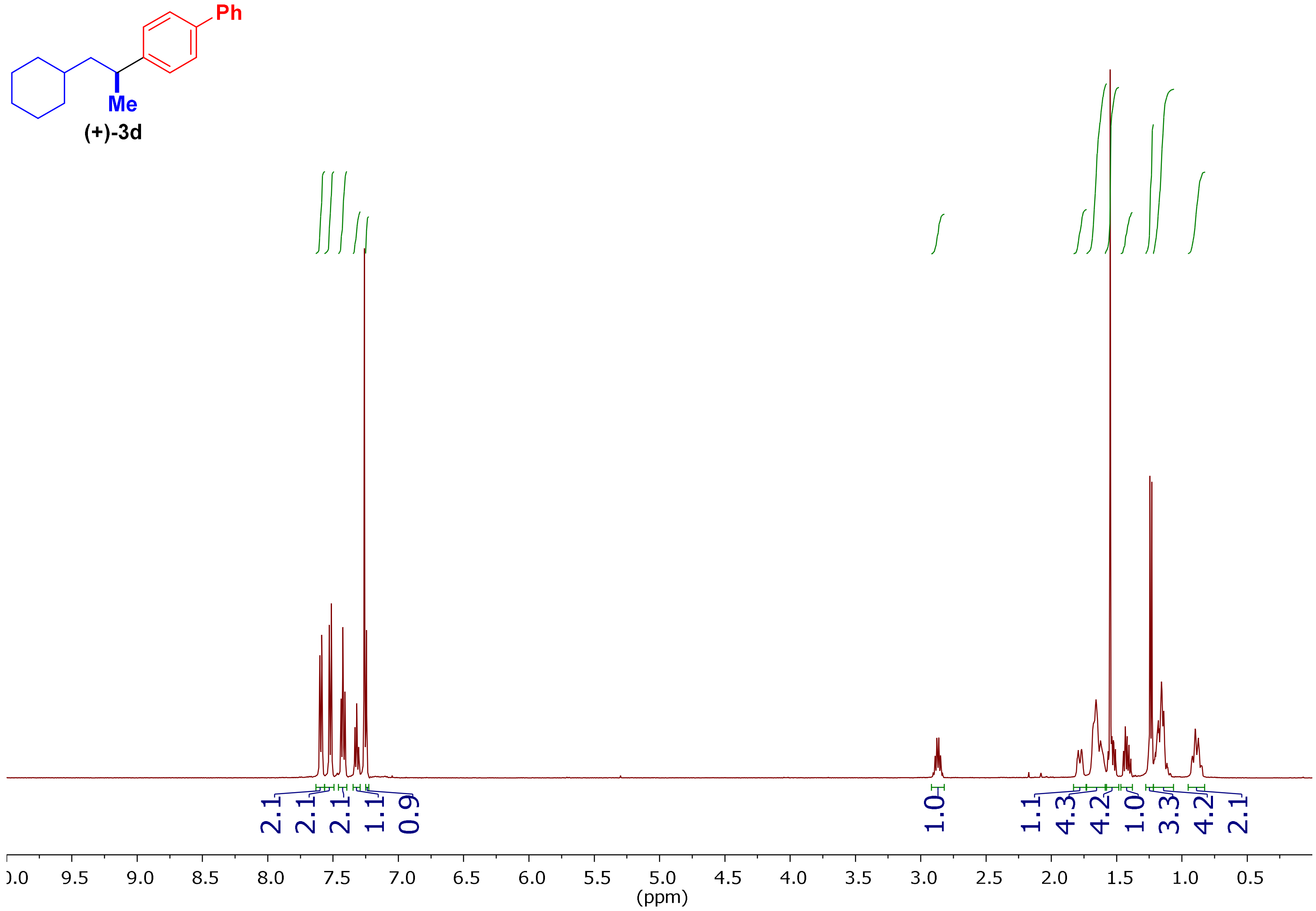

Supplementary Figure 161| ^{1}H-NMR spectrum (500 MHz, $CDCl_3$) for **(+)-3d**.

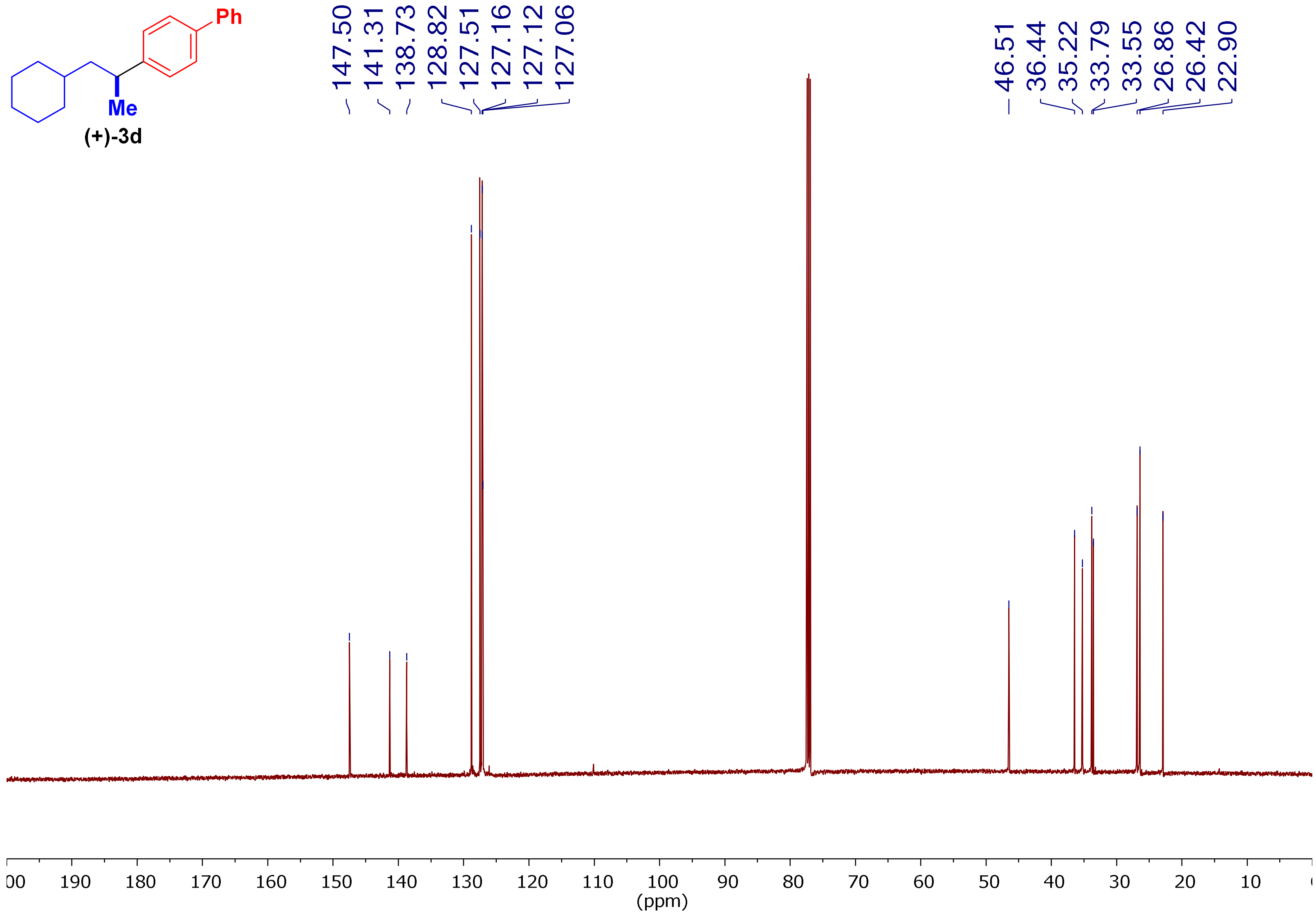

Supplementary Figure 162| ^{13}C-NMR spectrum (126 MHz, $CDCl_3$) for **(+)-3d**.

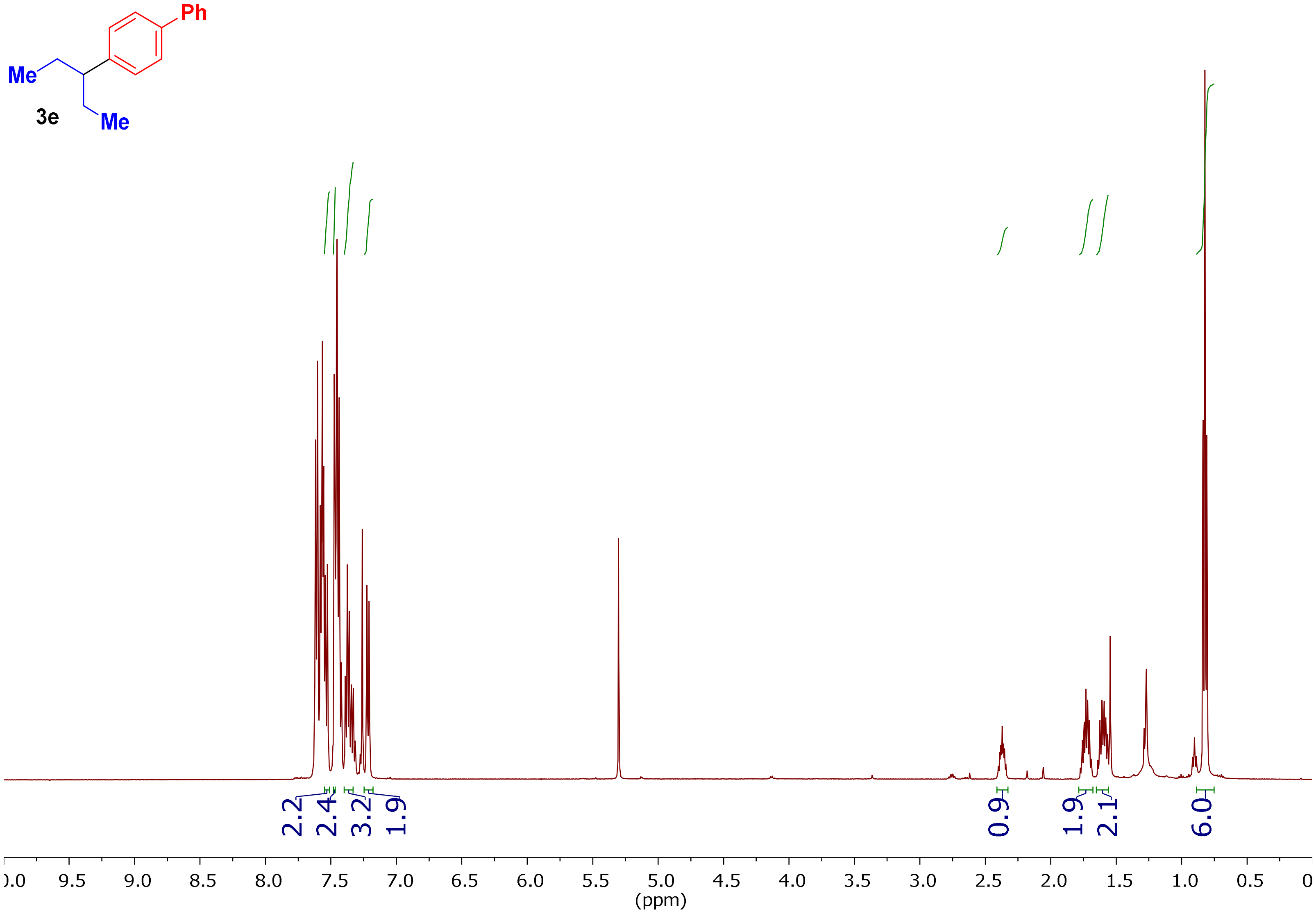

Supplementary Figure 163| ^{1}H-NMR spectrum (500 MHz, $CDCl_3$) for **3e**.

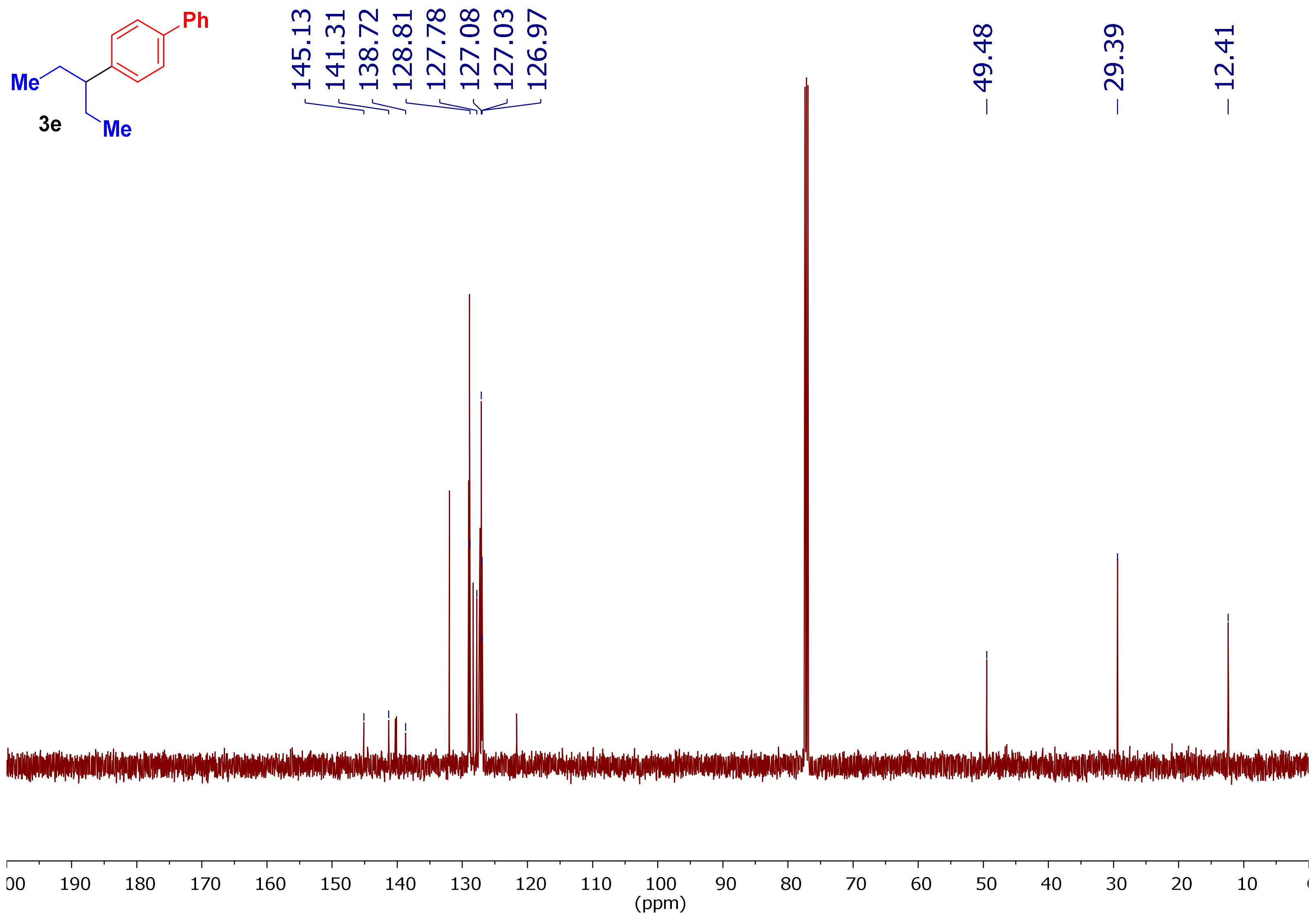

Supplementary Figure 164| ^{13}C-NMR spectrum (126 MHz, $CDCl_3$) for **3e**.

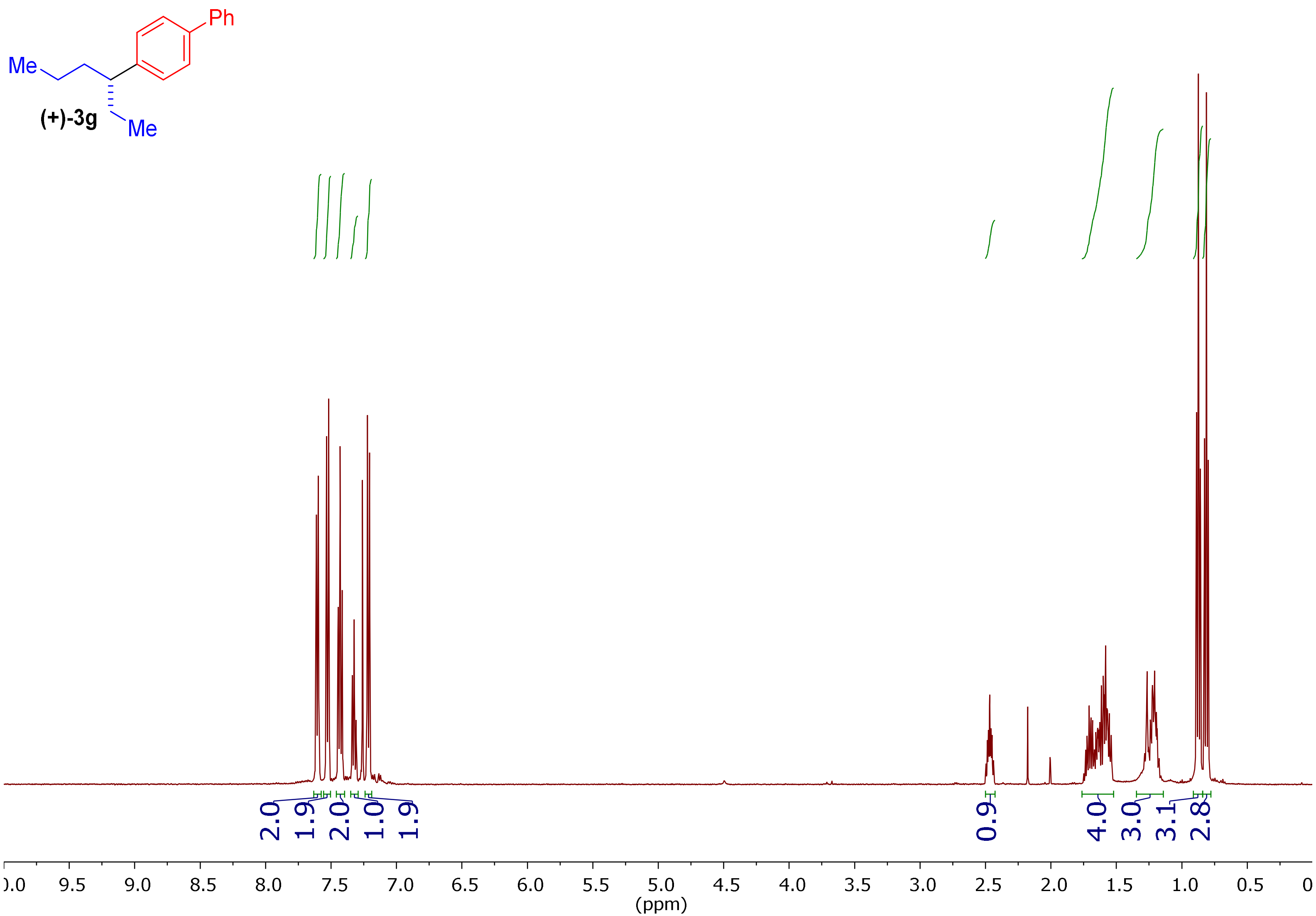

Supplementary Figure 165| ^{1}H-NMR spectrum (500 MHz, $CDCl_3$) for **(+)-3g**.

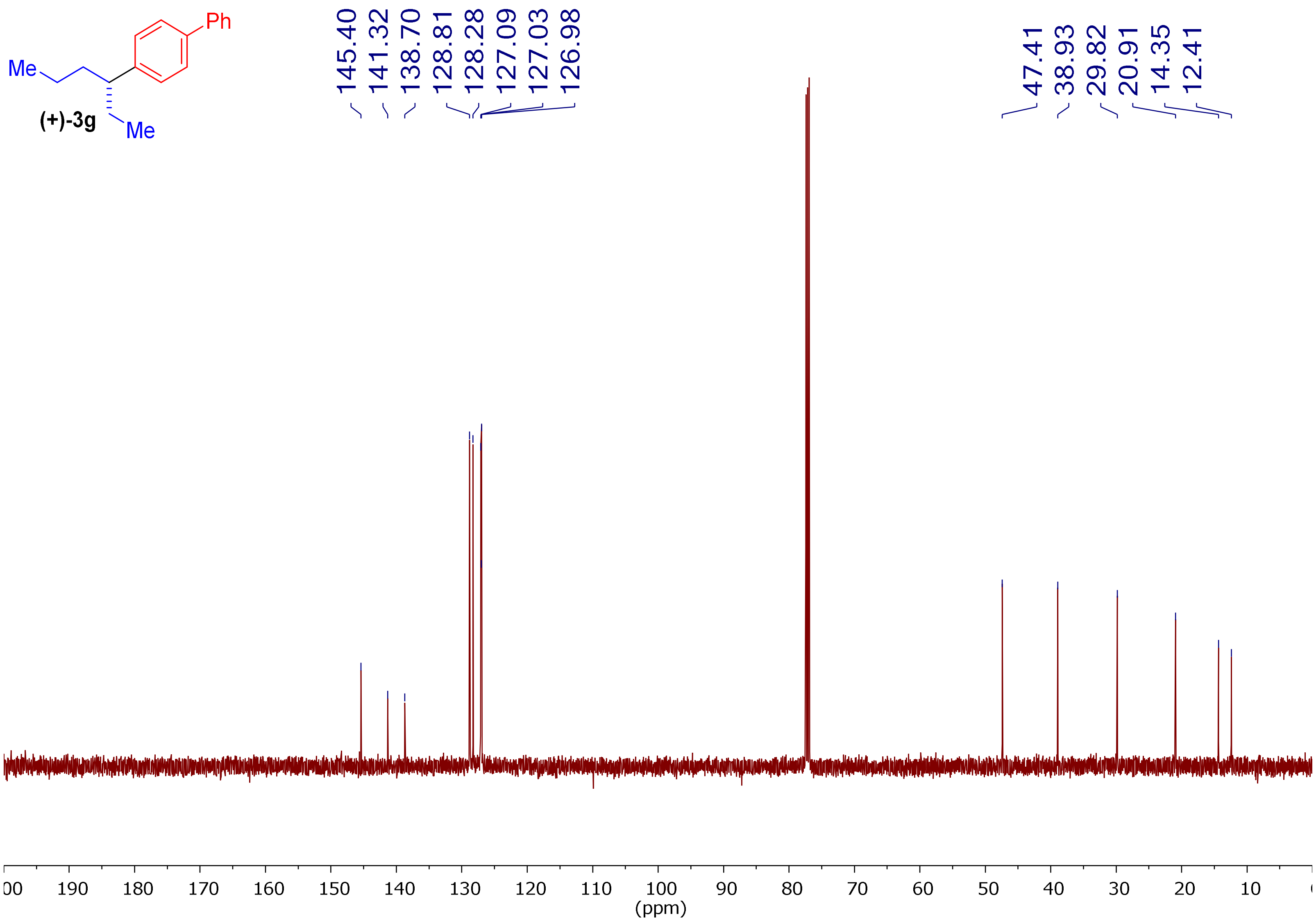

Supplementary Figure 166| ^{13}C-NMR spectrum (126 MHz, $CDCl_3$) for **(+)-3g**.

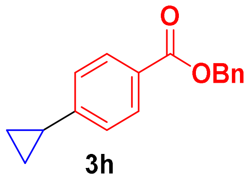

3h

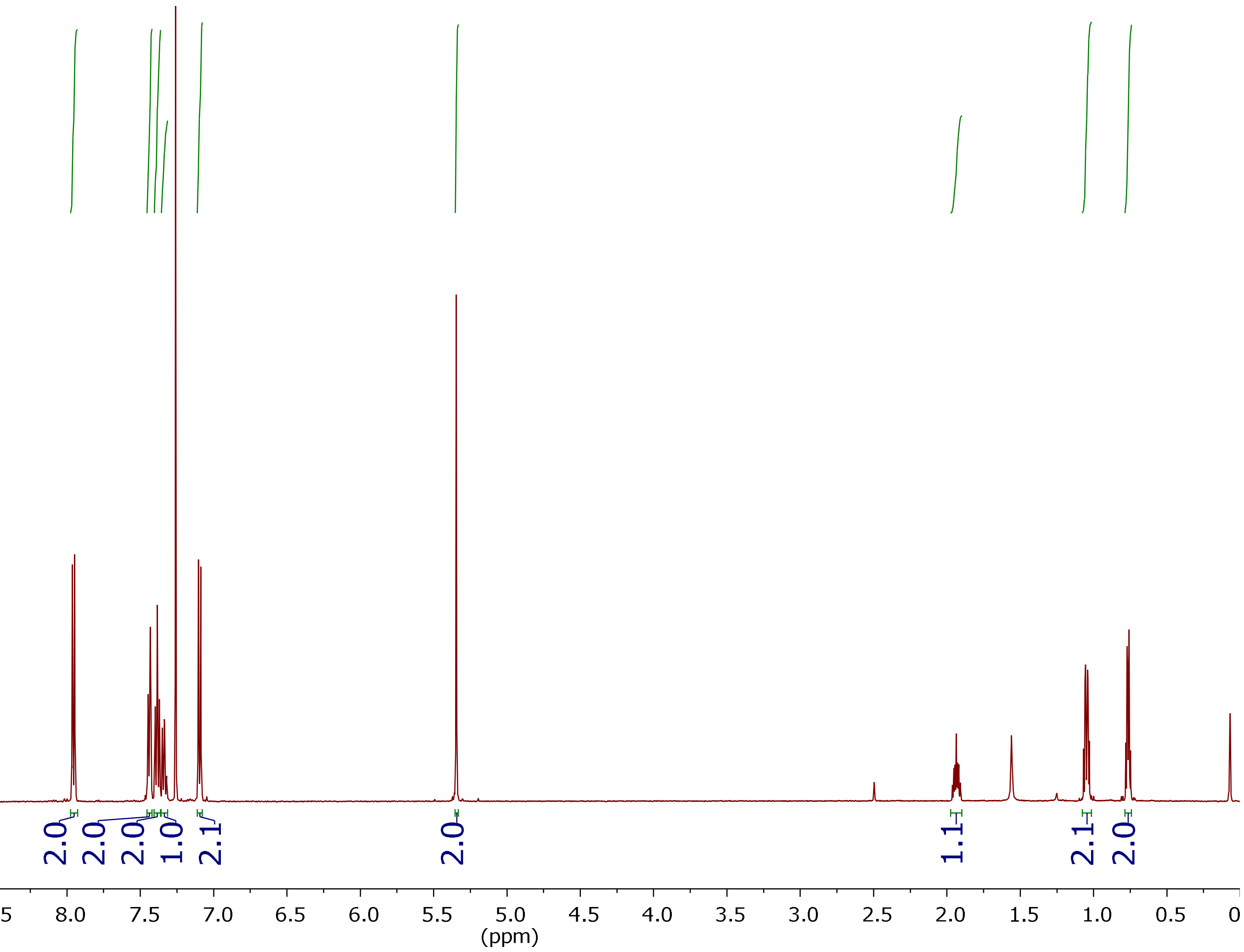

Supplementary Figure 167| ^{1}H-NMR spectrum (500 MHz, $CDCl_3$) for **3h**.

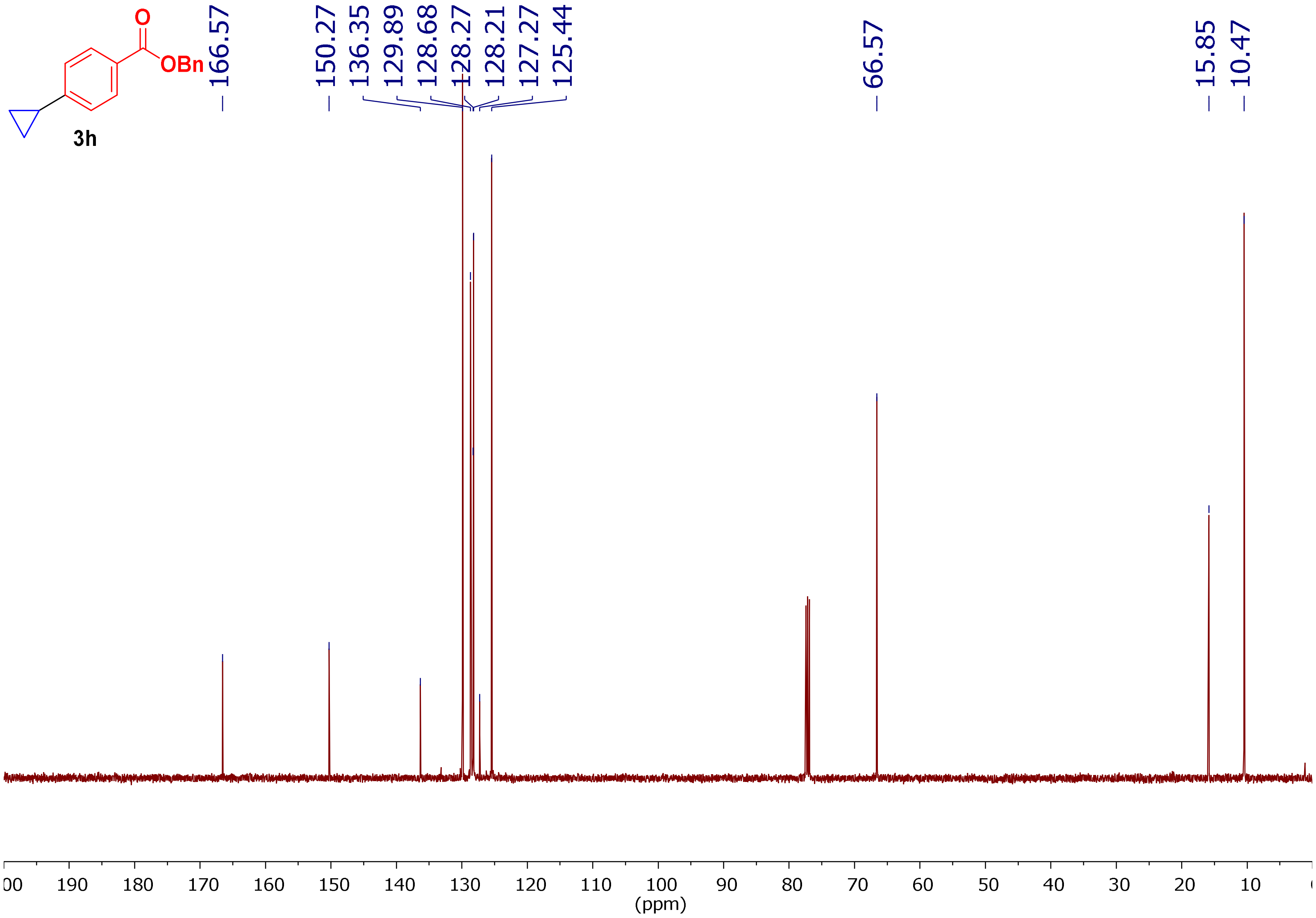

Supplementary Figure 168| ^{13}C-NMR spectrum (126 MHz, $CDCl_3$) for **3h**.

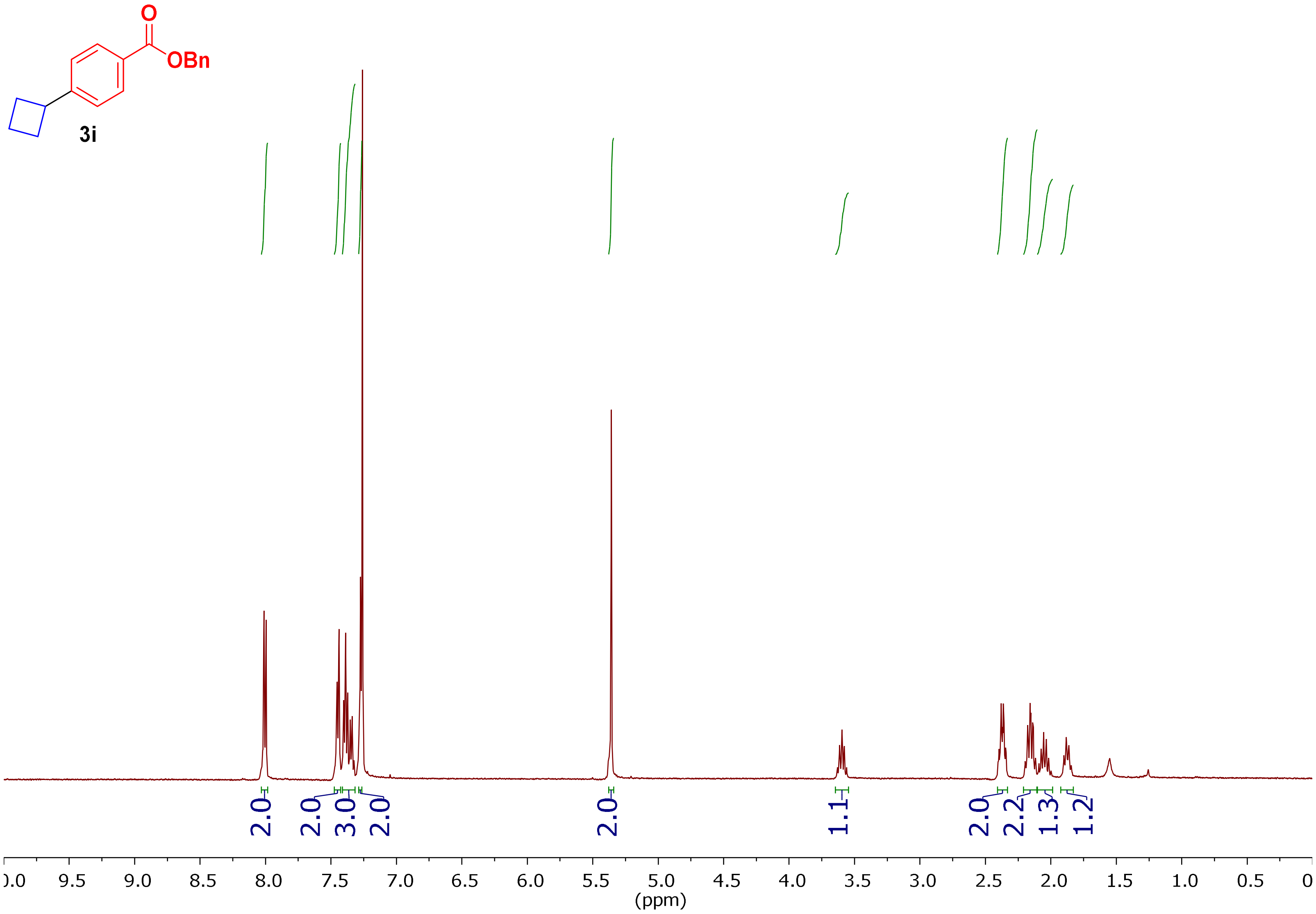

Supplementary Figure 169| ^{1}H-NMR spectrum (500 MHz, $CDCl_3$) for **3i**.

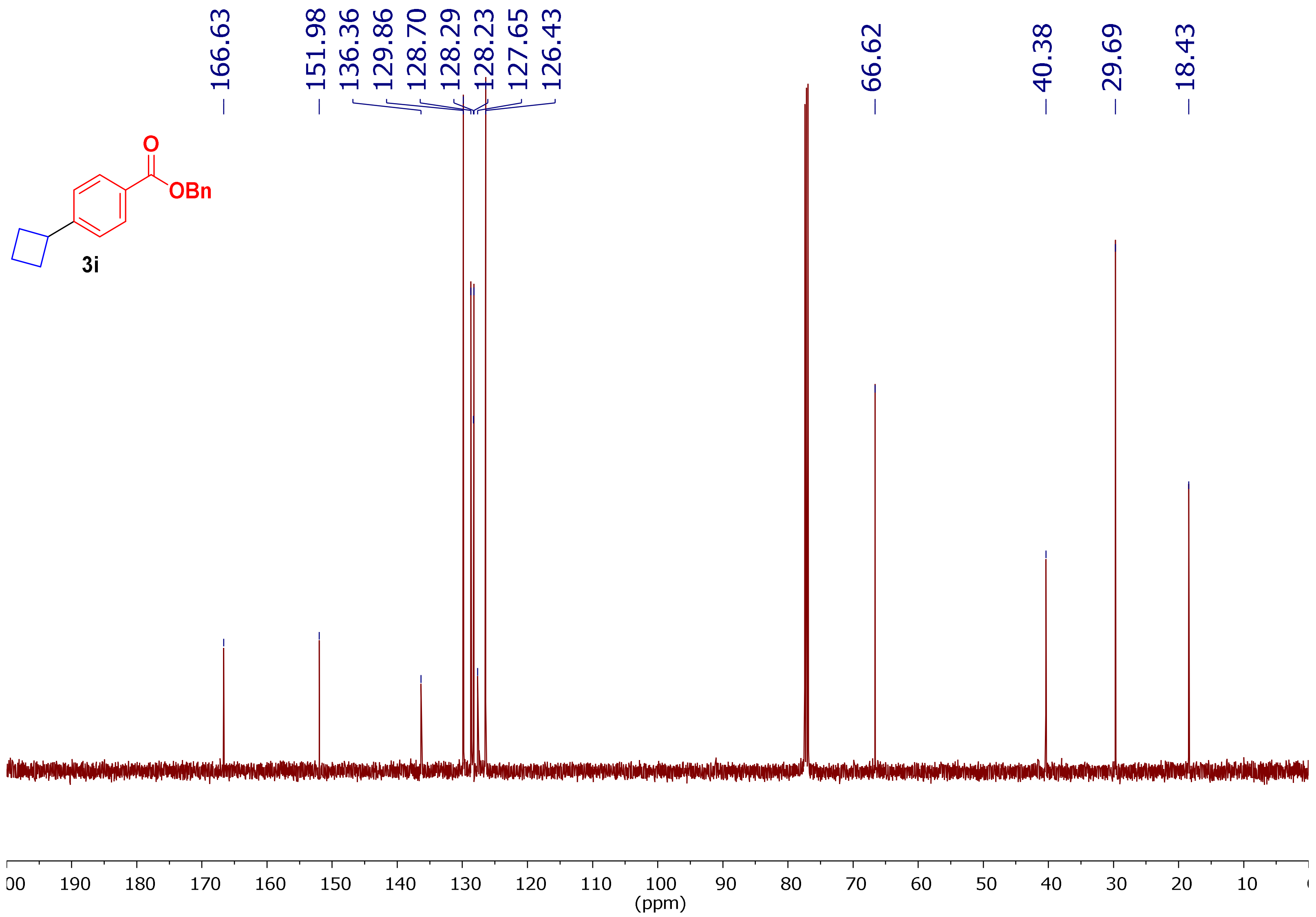

Supplementary Figure 170| ^{13}C-NMR spectrum (126 MHz, $CDCl_3$) for **3i**.

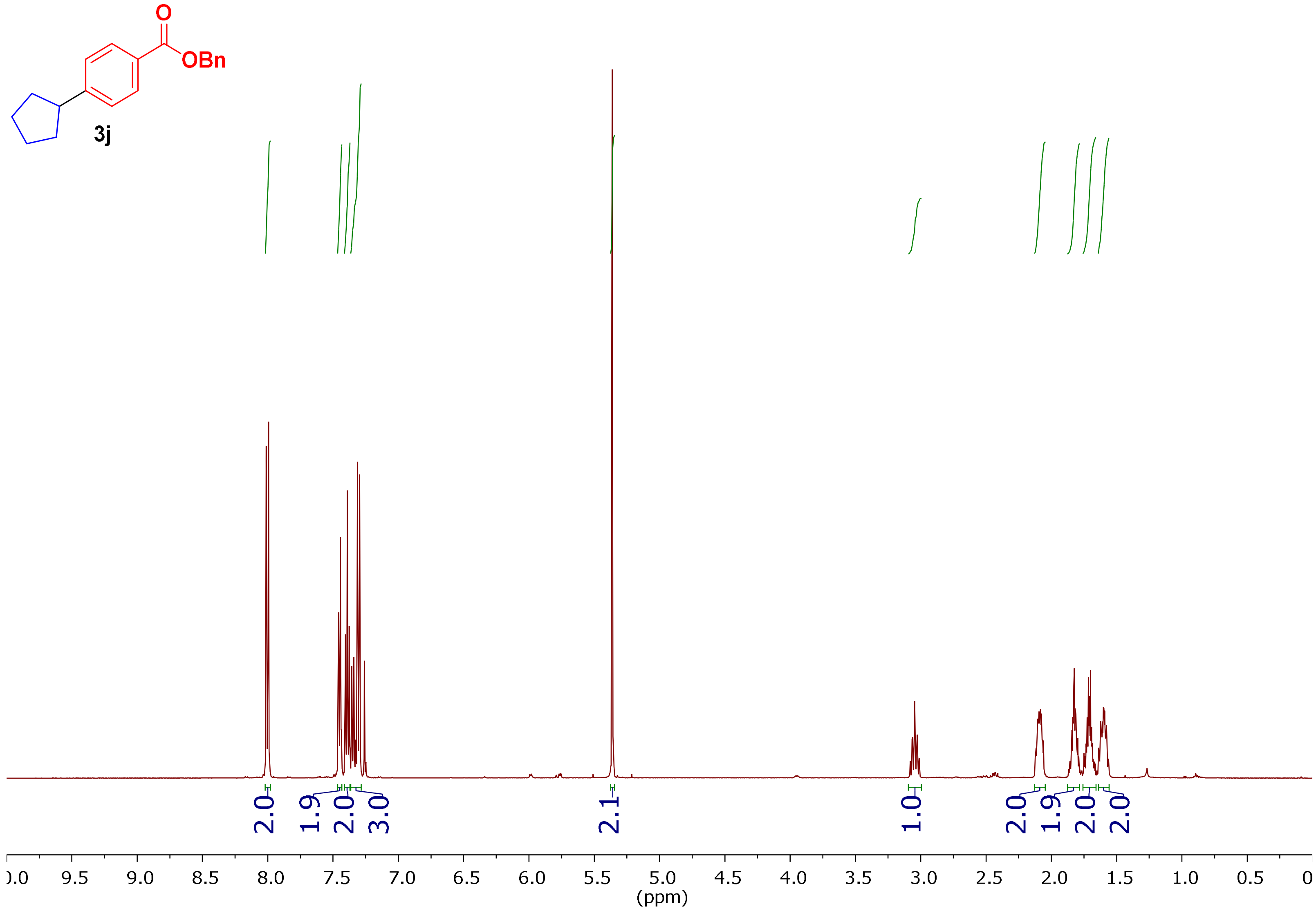

Supplementary Figure 171| ^{1}H-NMR spectrum (500 MHz, $CDCl_3$) for **3j**.

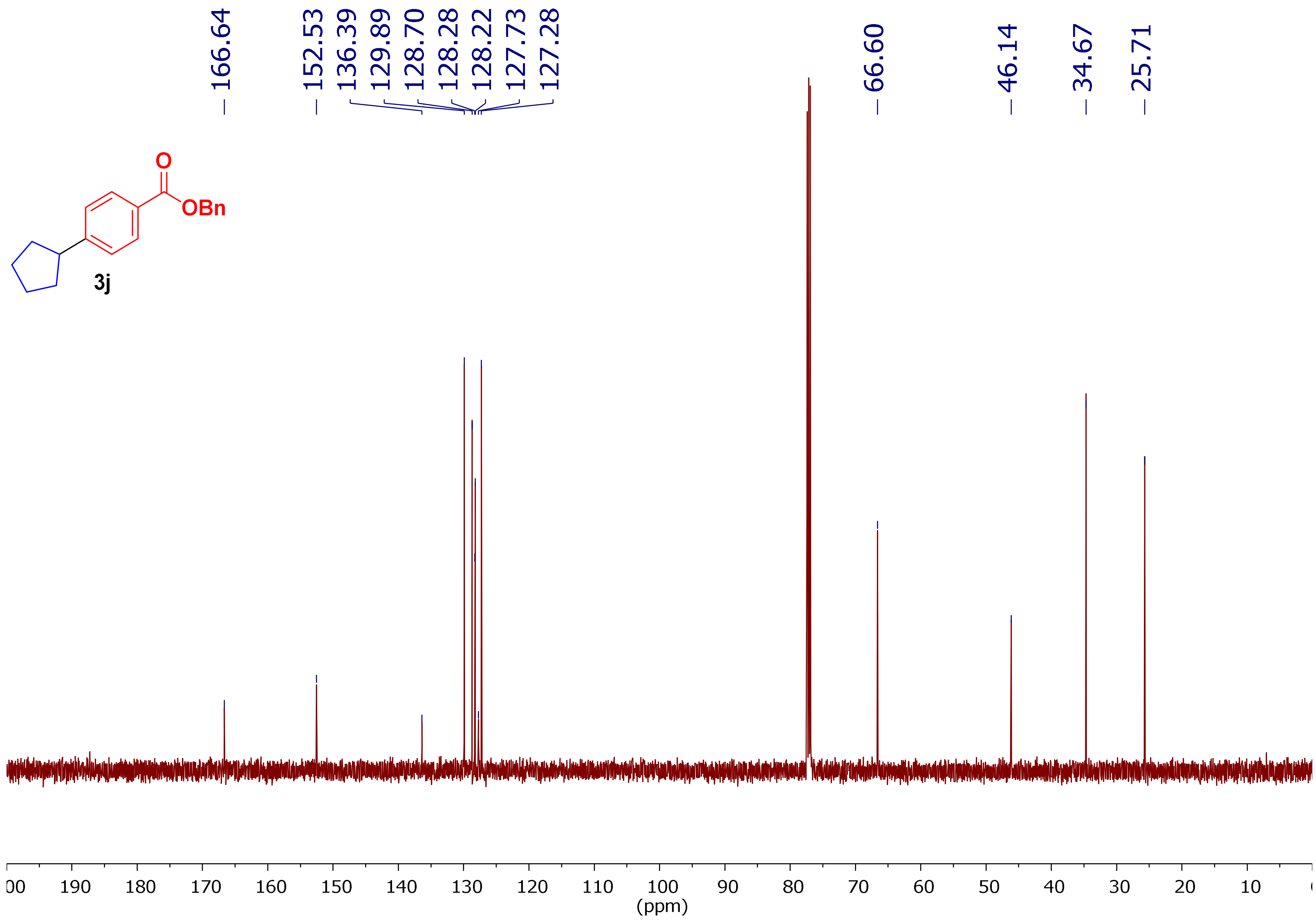

Supplementary Figure 172| ^{13}C-NMR spectrum (126 MHz, $CDCl_3$) for **3j**.

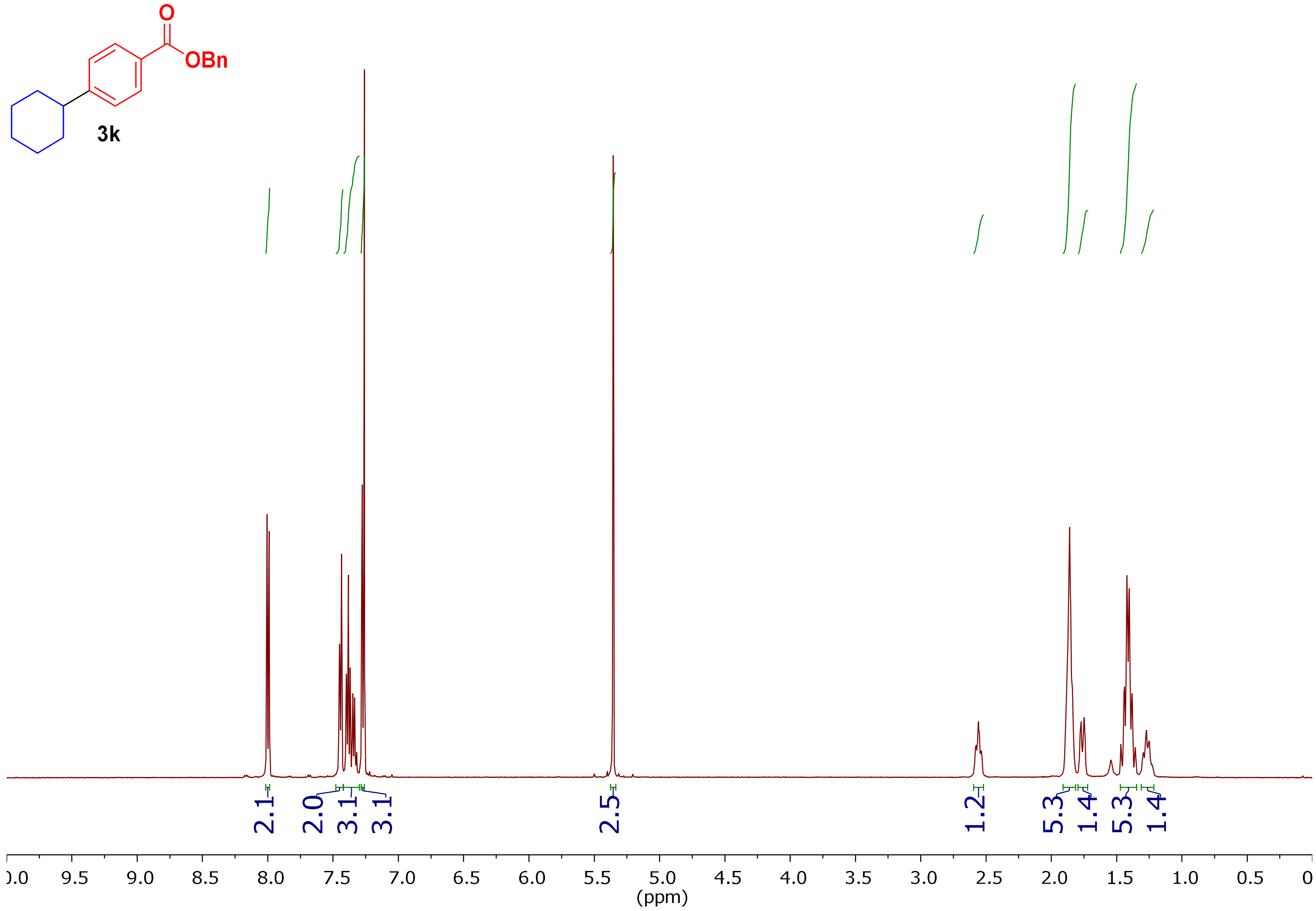

Supplementary Figure 173| ^{1}H-NMR spectrum (500 MHz, $CDCl_3$) for **3k**.

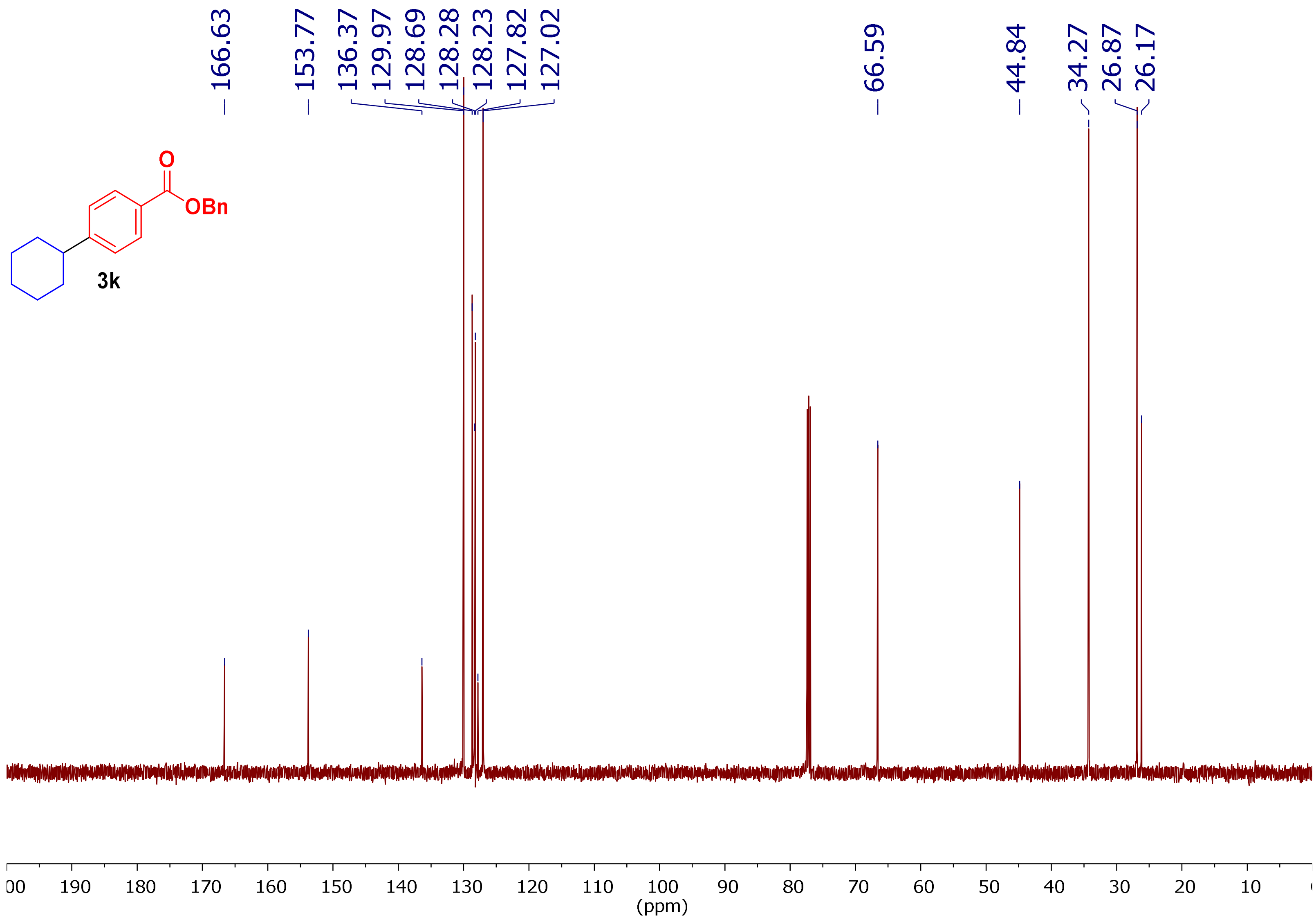

Supplementary Figure 174| ^{13}C-NMR spectrum (126 MHz, $CDCl_3$) for **3k**.

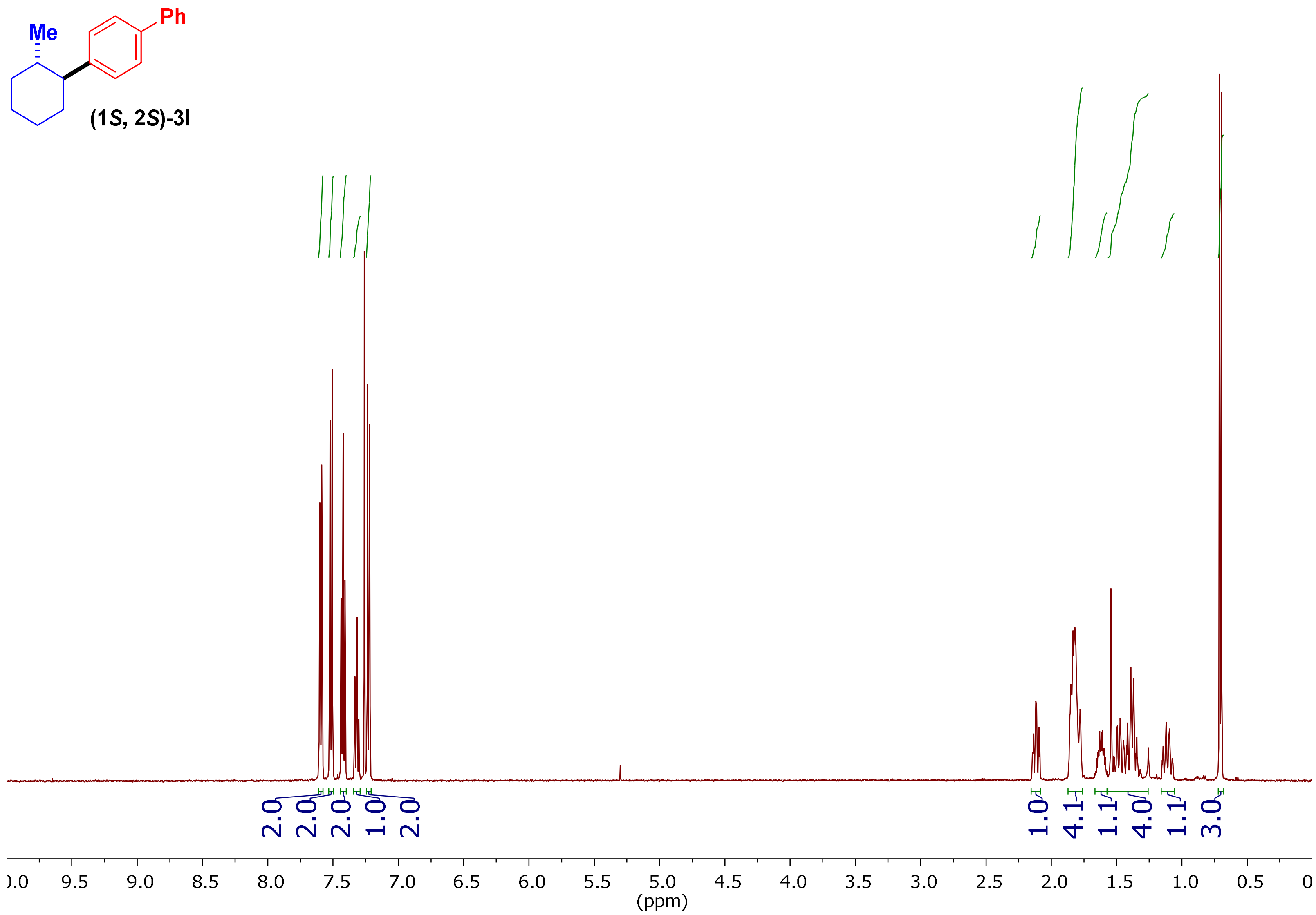

Supplementary Figure 175| ^{1}H-NMR spectrum (500 MHz, $CDCl_3$) for **(1*S*, 2*S*)-3l**.

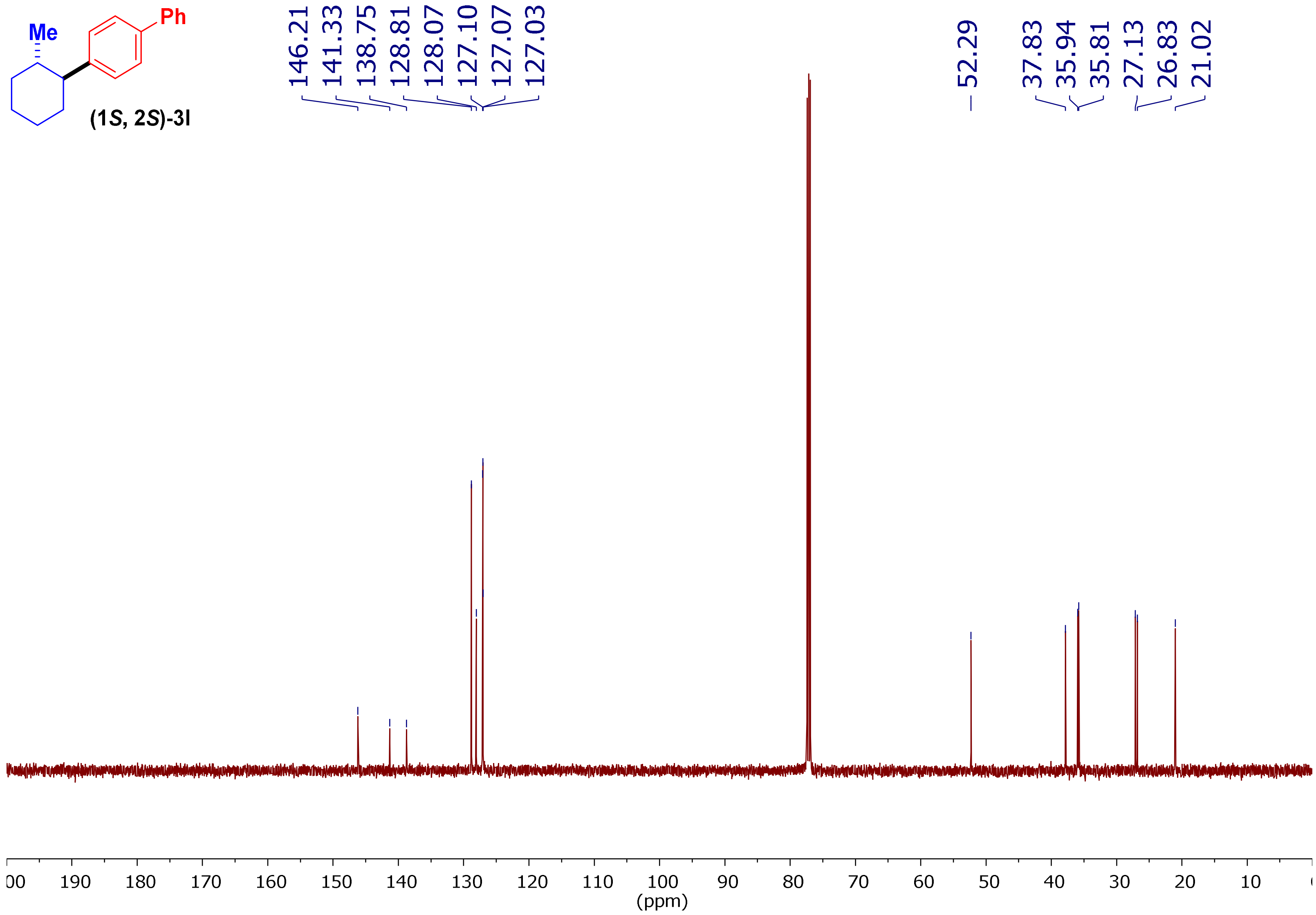

Supplementary Figure 176| ^{13}C-NMR spectrum (126 MHz, $CDCl_3$) for **(1*S*, 2*S*)-3l**.

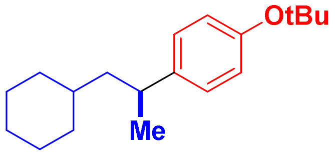

(+)-3m

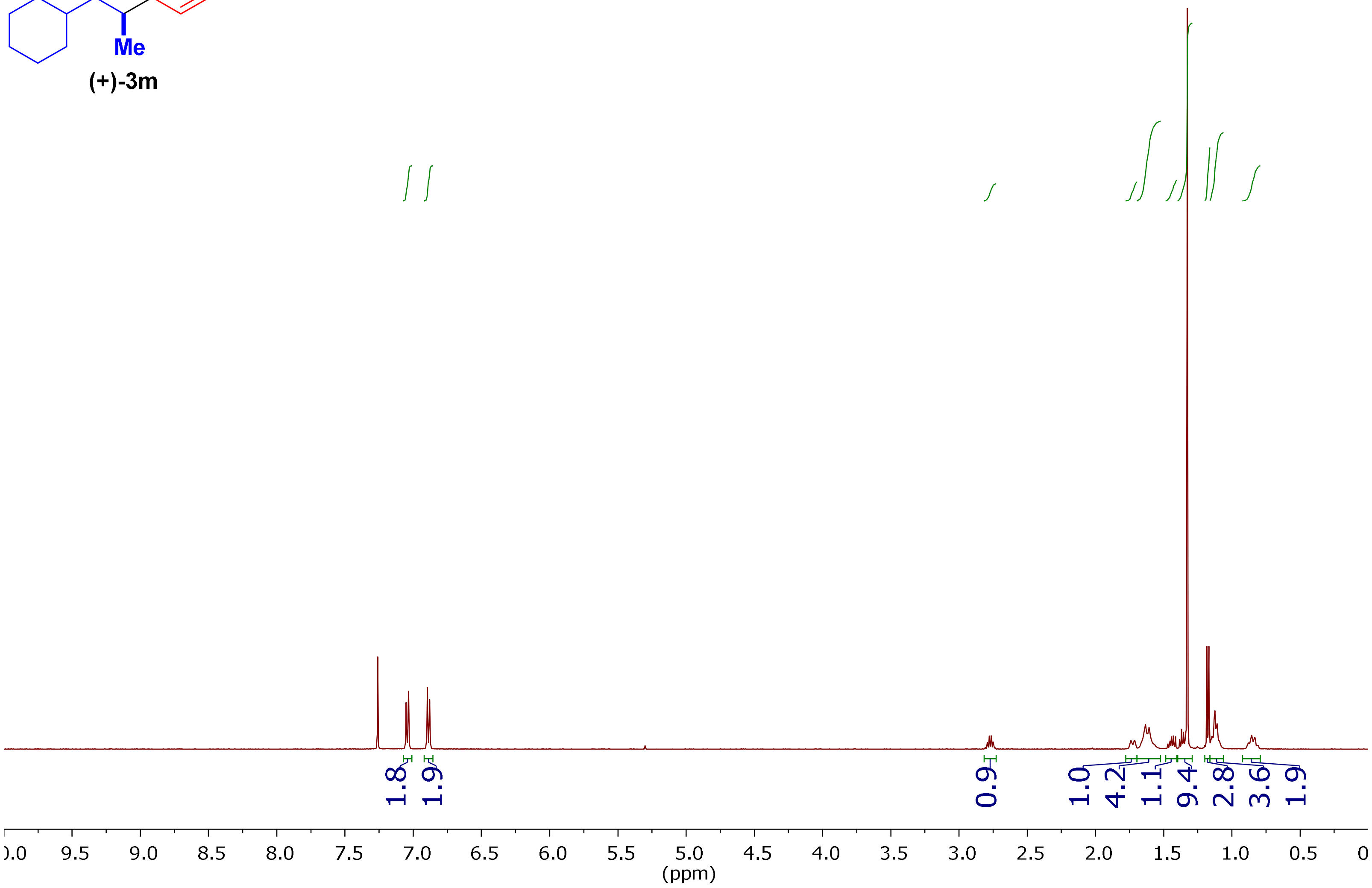

Supplementary Figure 177| ^{1}H-NMR spectrum (500 MHz, $CDCl_3$) for **(+)-3m**.

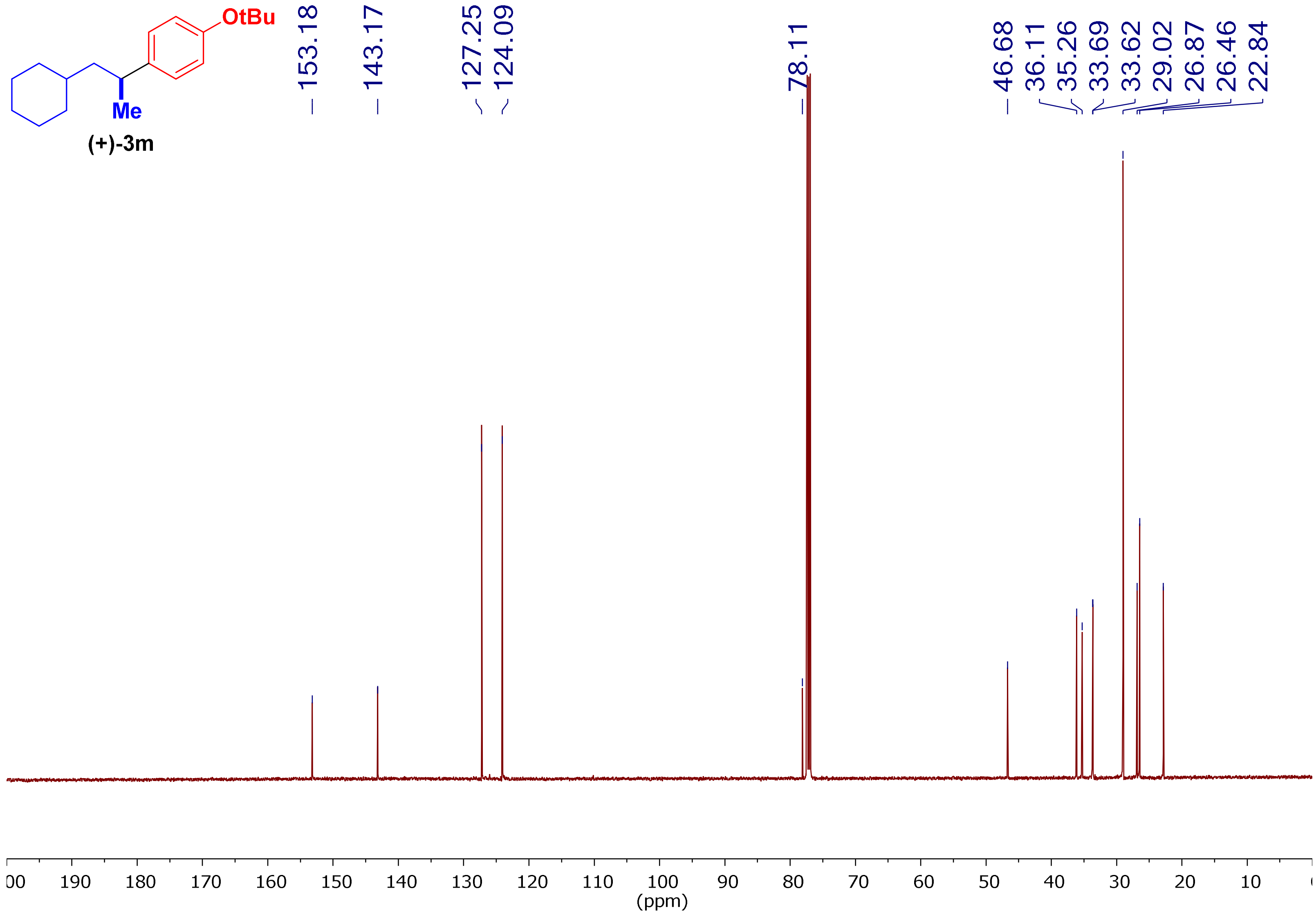

Supplementary Figure 178| ^{13}C-NMR spectrum (126 MHz, $CDCl_3$) for **(+)-3m**.

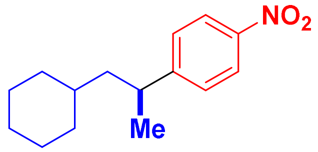

(+)-3n

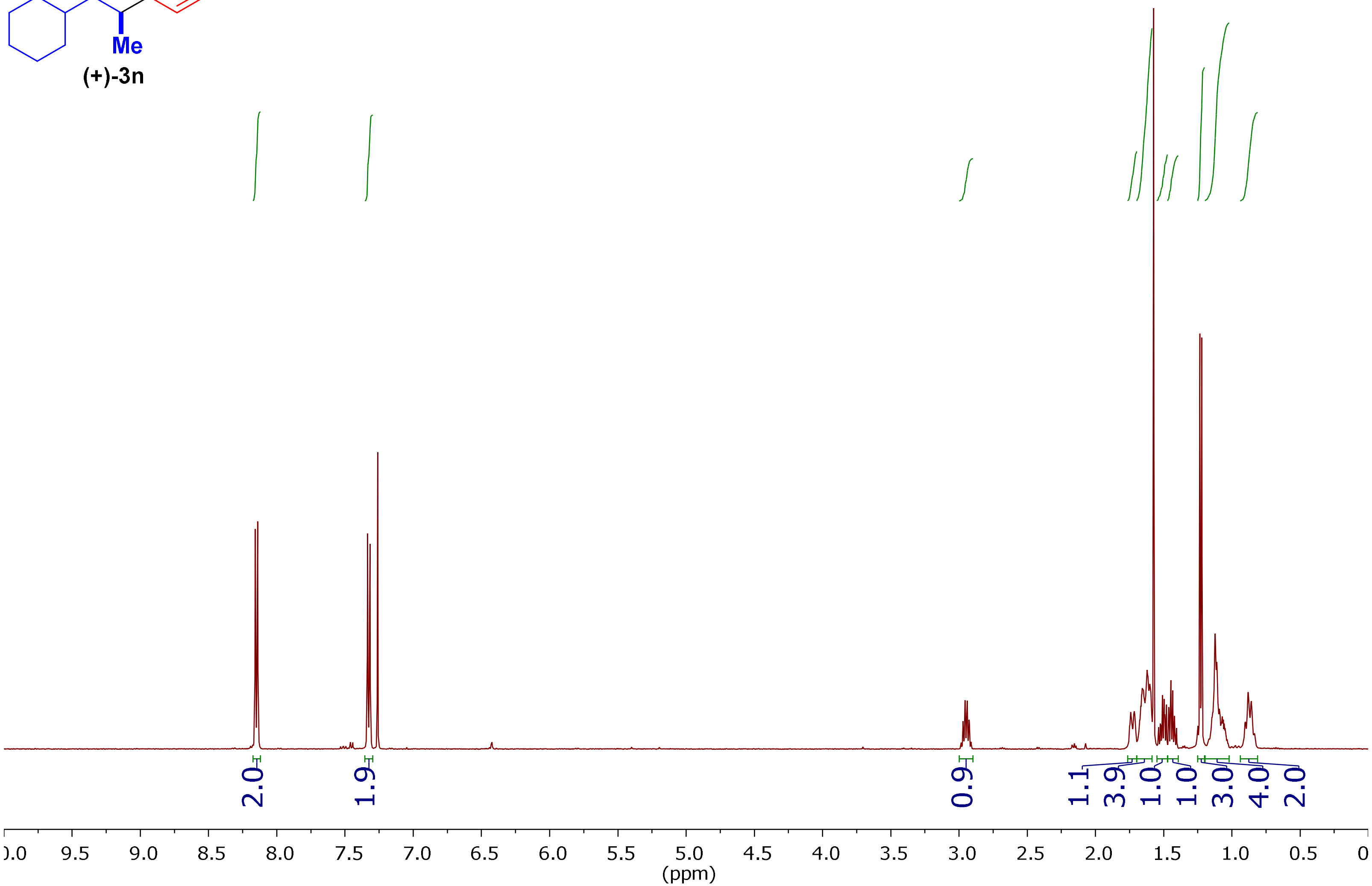

Supplementary Figure 179| ^{1}H-NMR spectrum (500 MHz, $CDCl_3$) for **(+)-3n**.

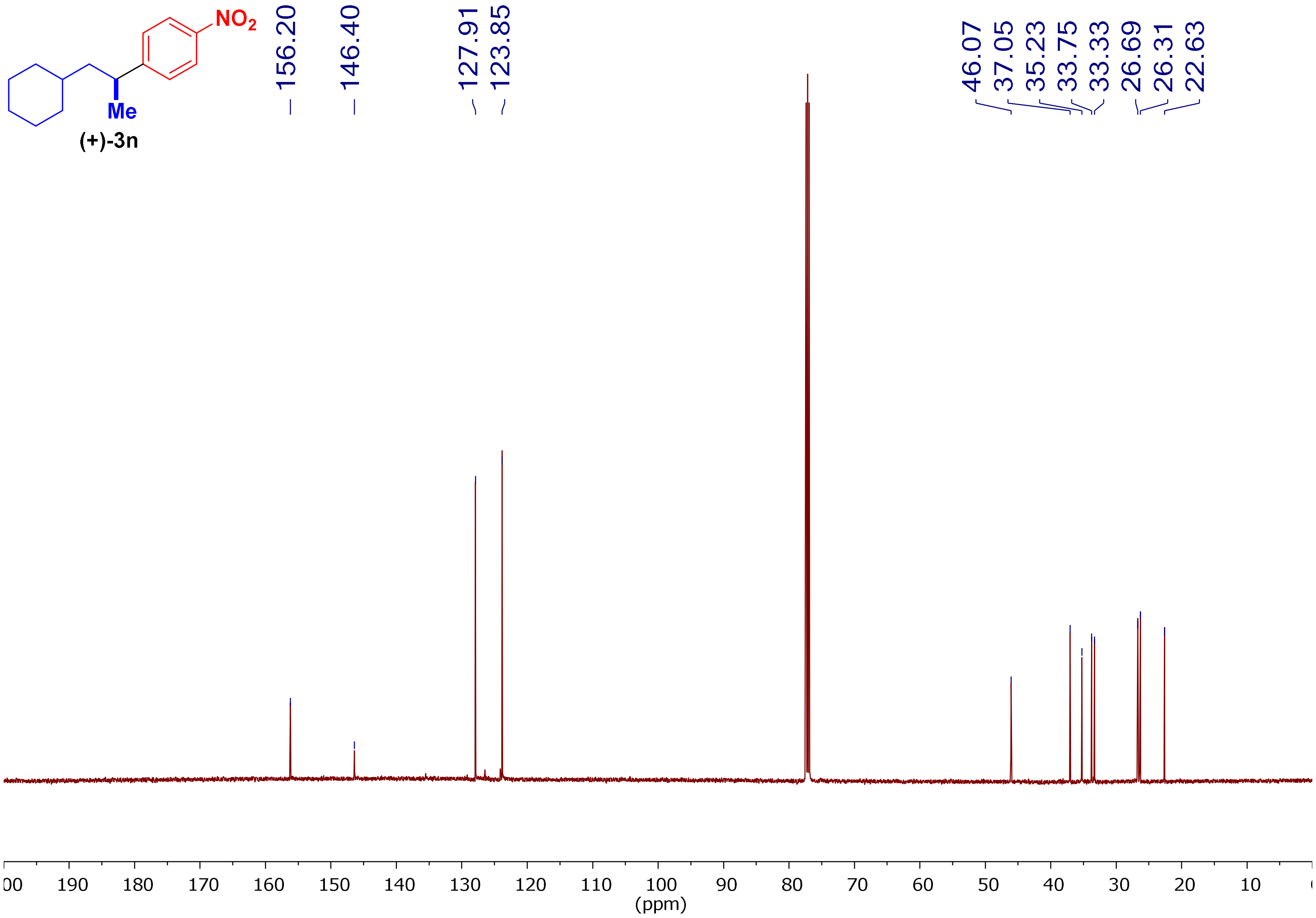

Supplementary Figure 180| ^{13}C-NMR spectrum (126 MHz, $CDCl_3$) for **(+)-3n**.

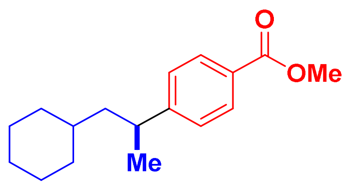

(+)-3o

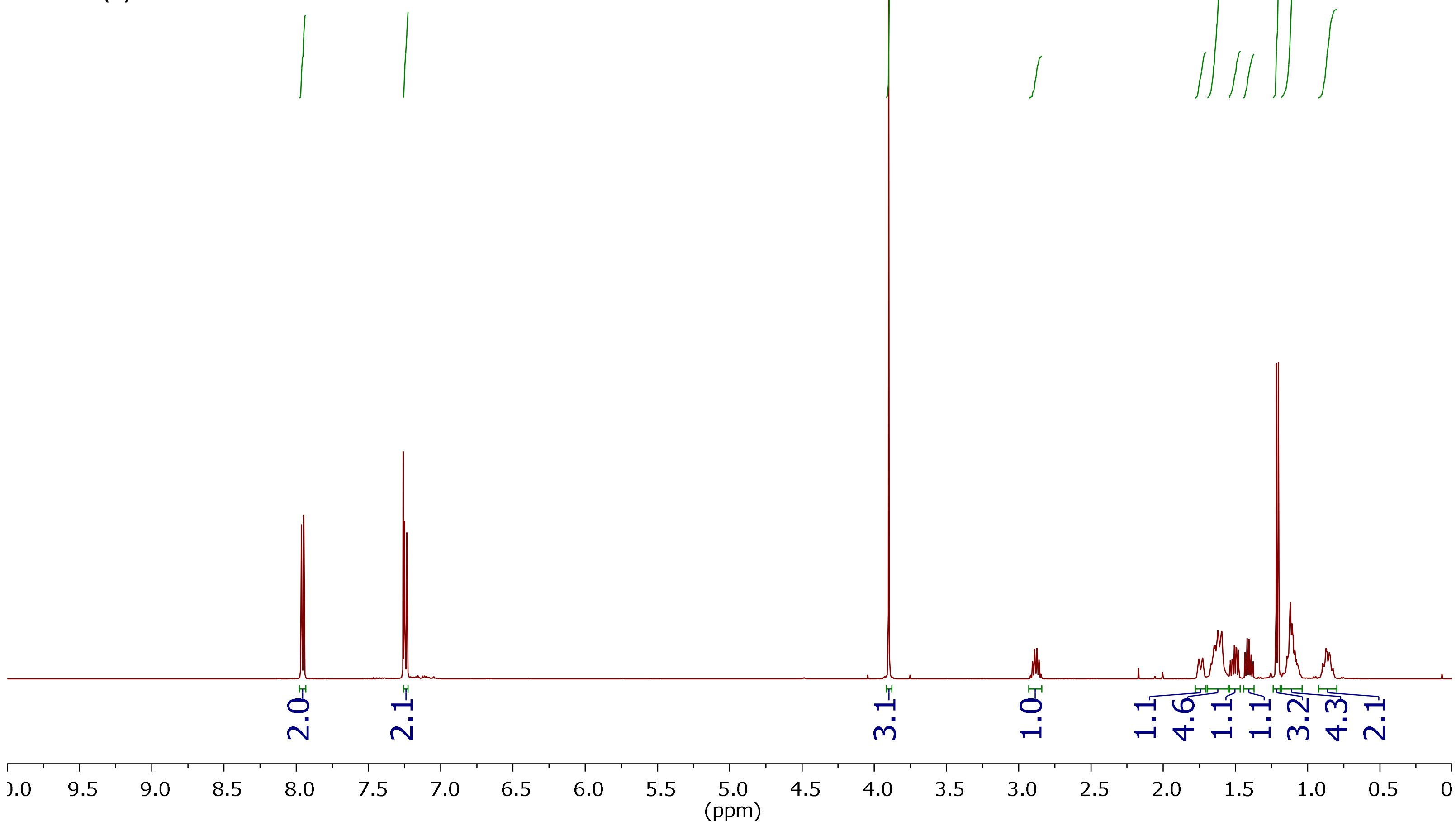

Supplementary Figure 181| ^{1}H-NMR spectrum (500 MHz, $CDCl_3$) for **(+)-3o**.

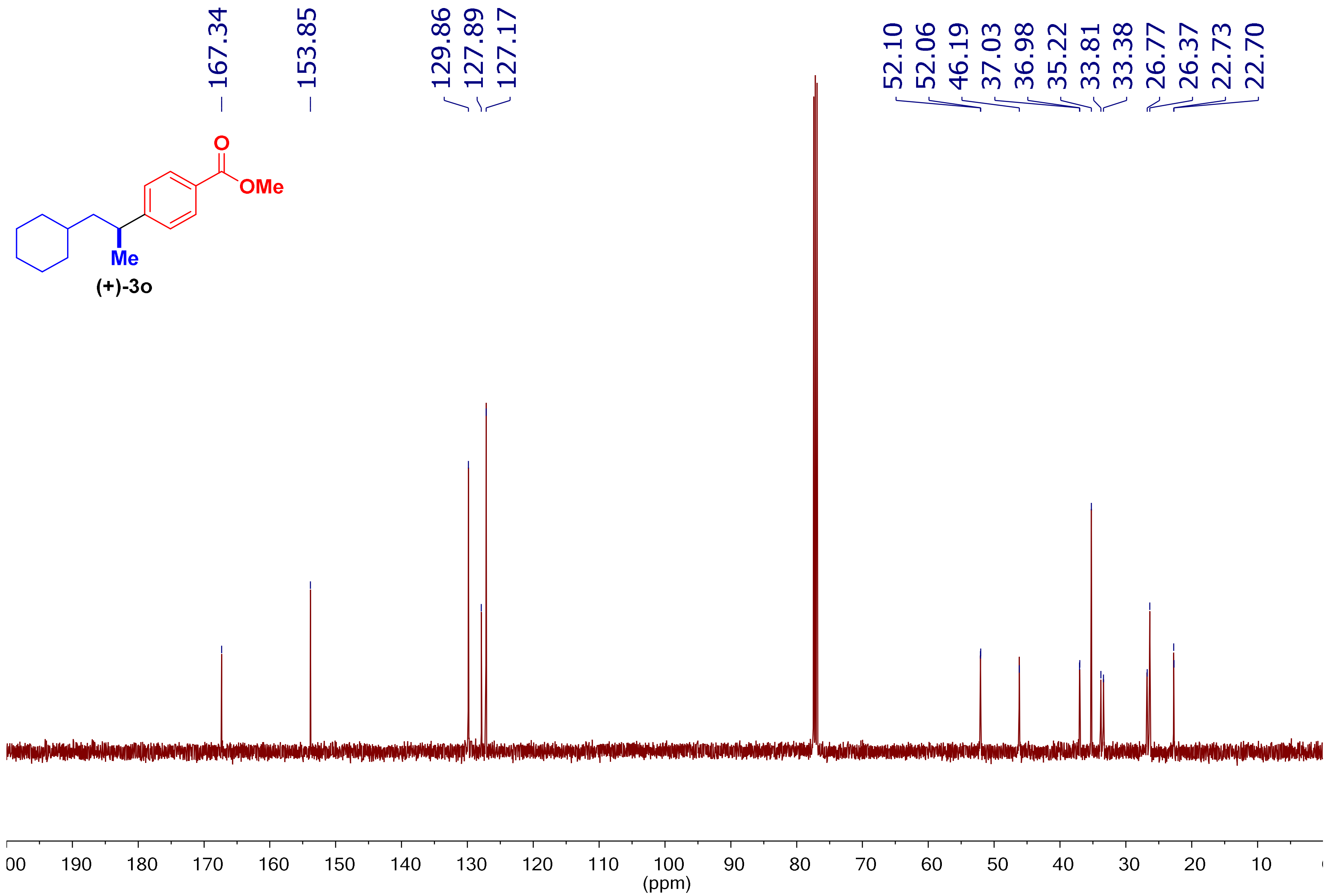

Supplementary Figure 182| ^{13}C-NMR spectrum (126 MHz, $CDCl_3$) for **(+)-3o**.

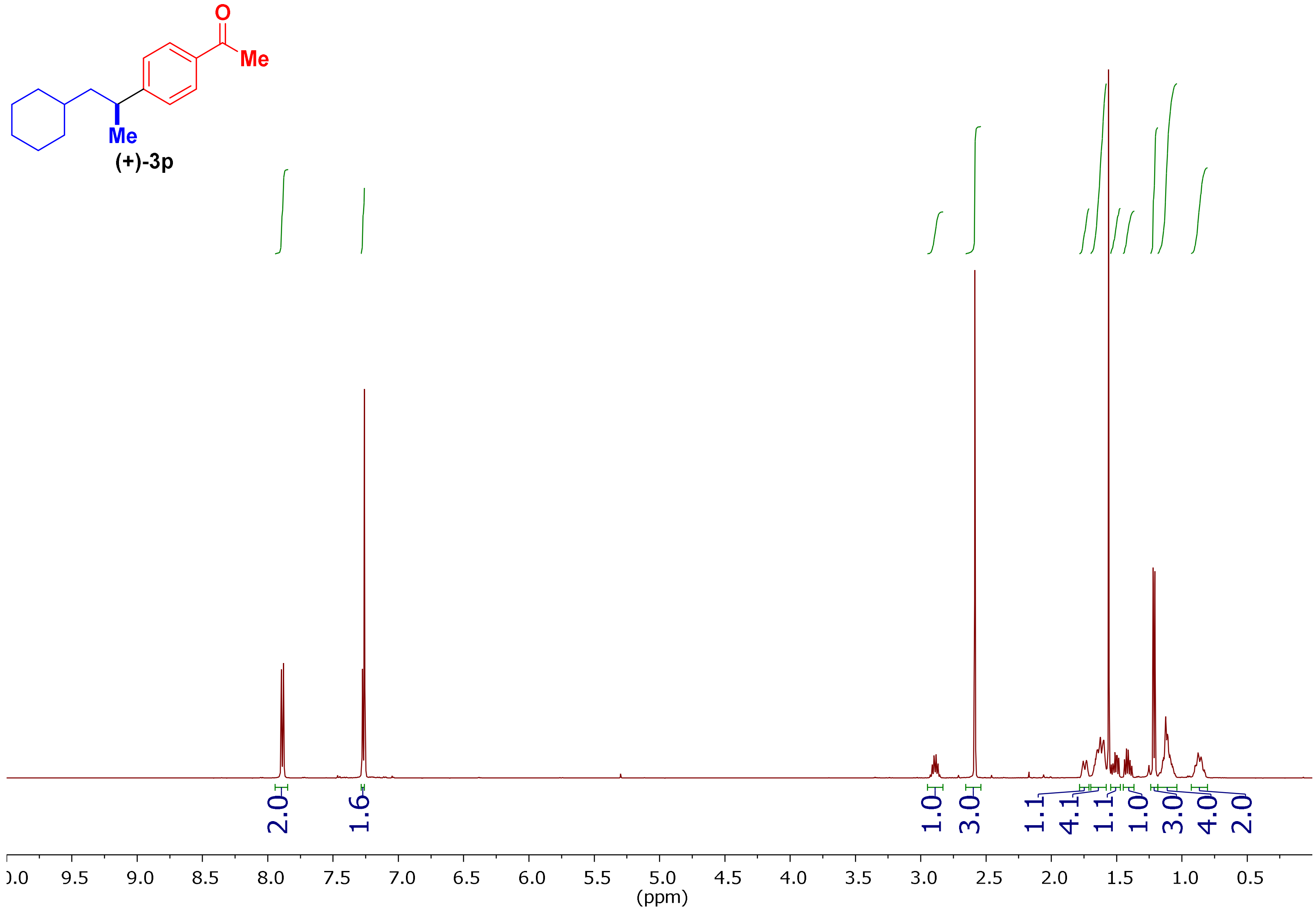

Supplementary Figure 183| ^{1}H-NMR spectrum (500 MHz, $CDCl_3$) for **(+)-3p**.

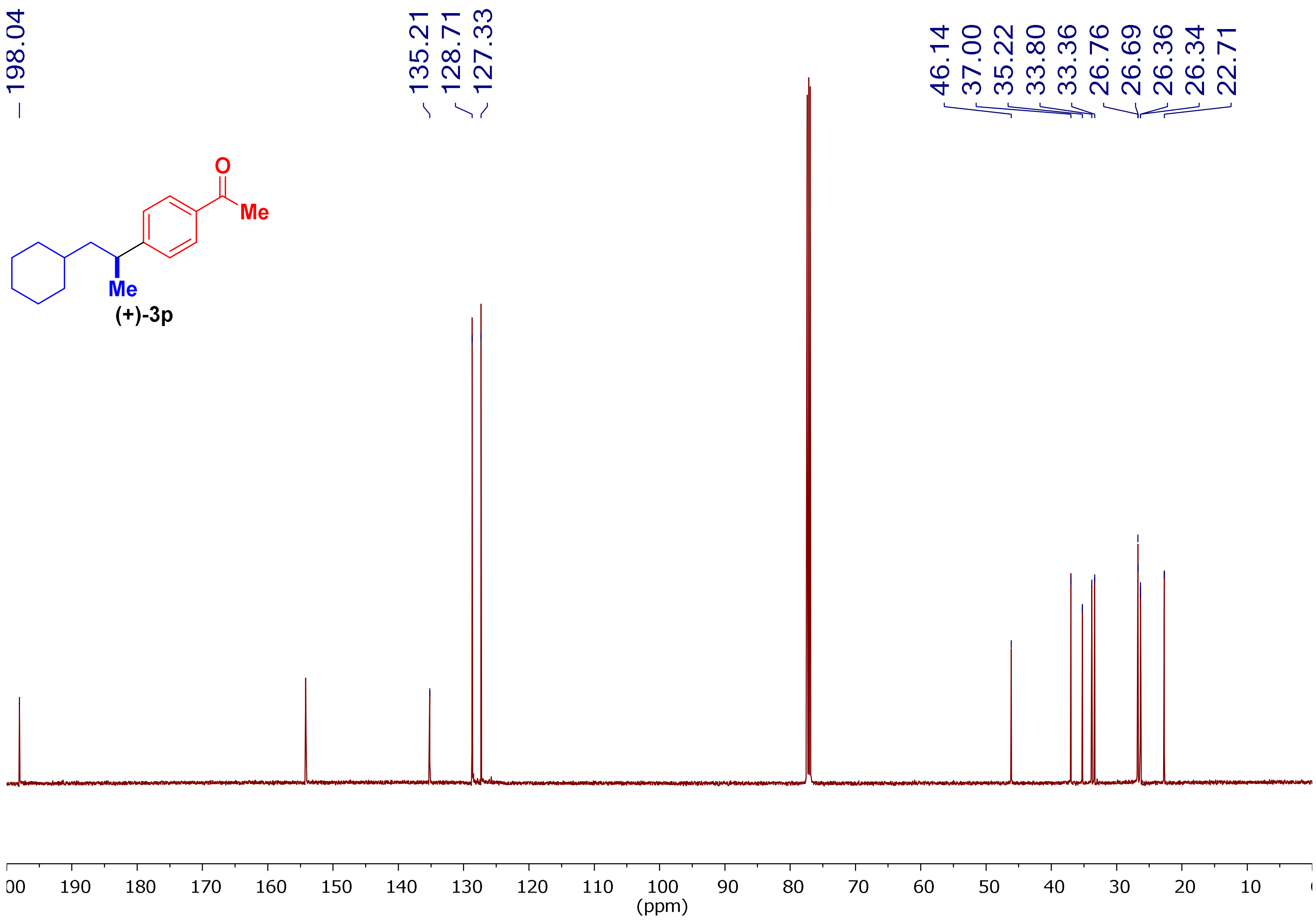

Supplementary Figure 184| ^{13}C-NMR spectrum (126 MHz, $CDCl_3$) for **(+)-3p**.

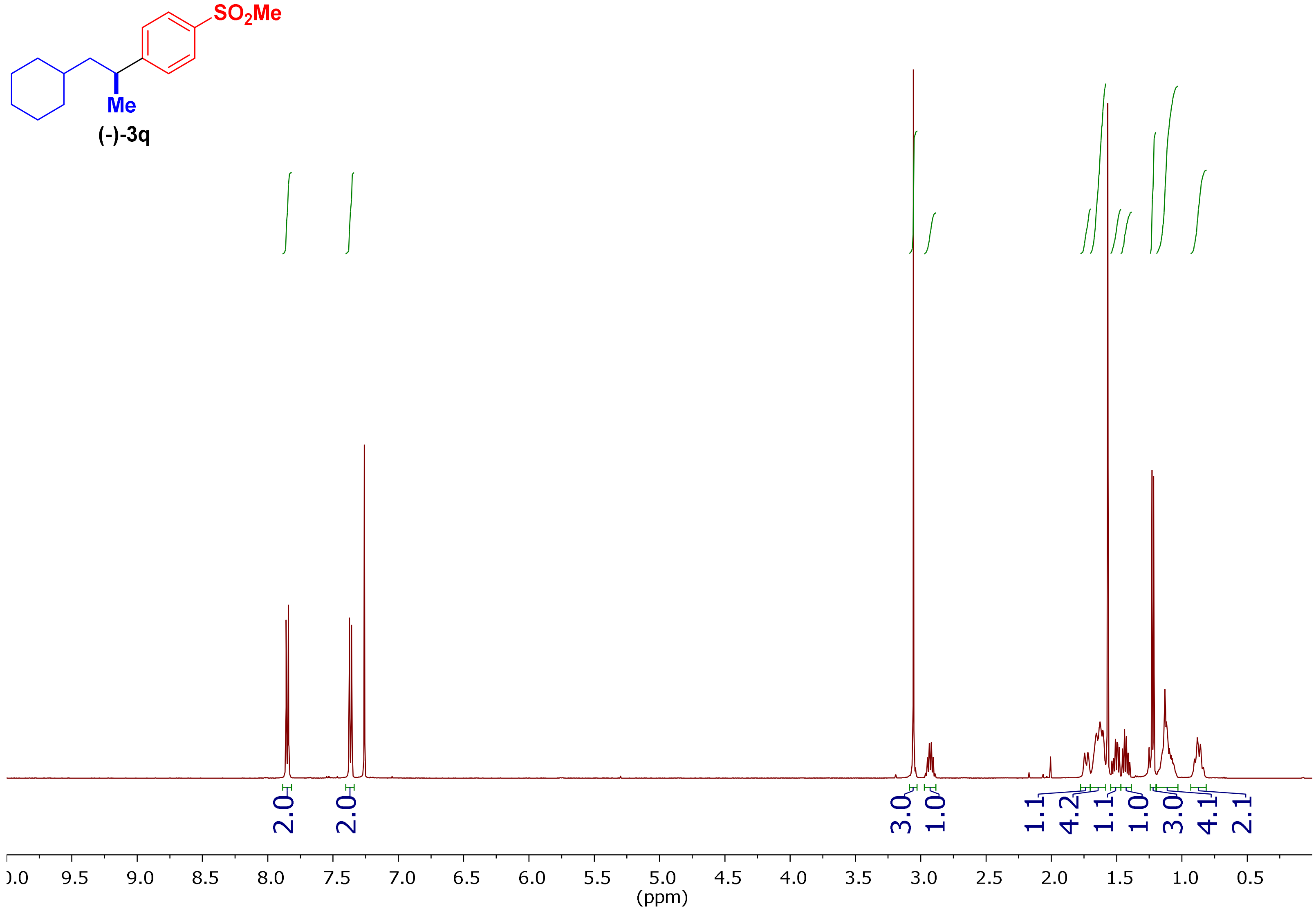

Supplementary Figure 185| ^{1}H-NMR spectrum (500 MHz, $CDCl_3$) for **(-)-3q**.

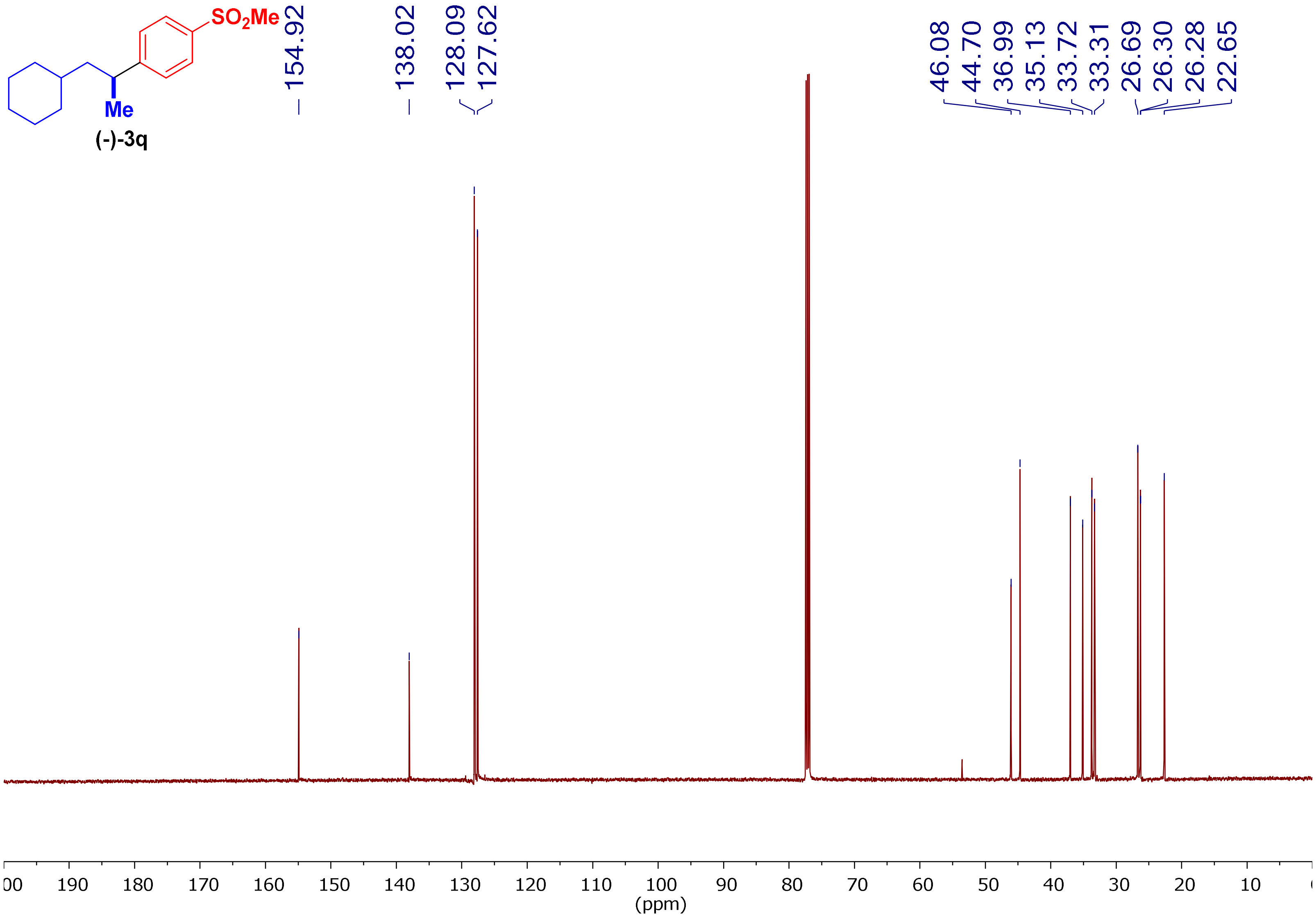

Supplementary Figure 186| ^{13}C-NMR spectrum (126 MHz, $CDCl_3$) for **(-)-3q**.

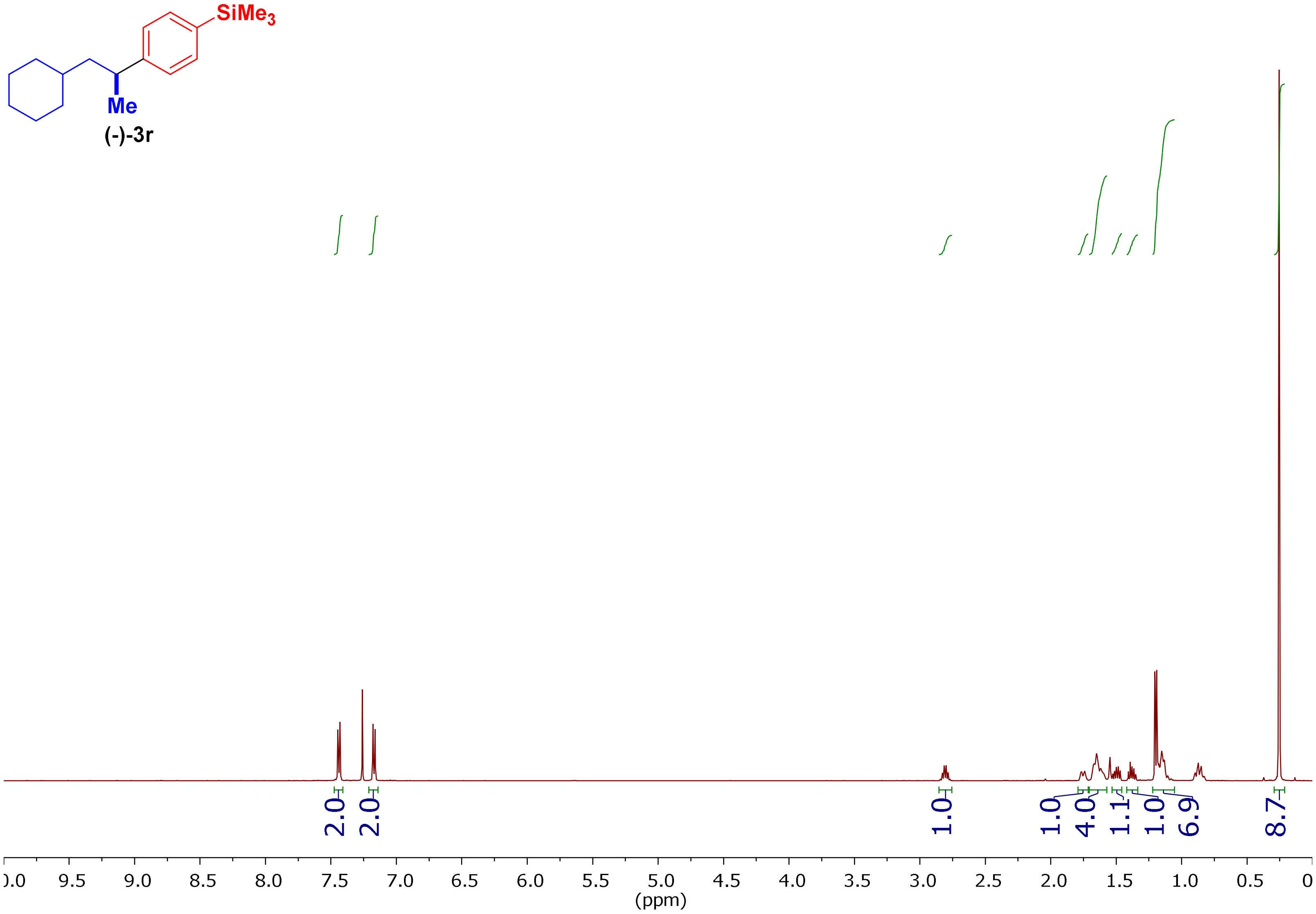

Supplementary Figure 187| ^{1}H-NMR spectrum (500 MHz, $CDCl_3$) for **(-)-3r**.

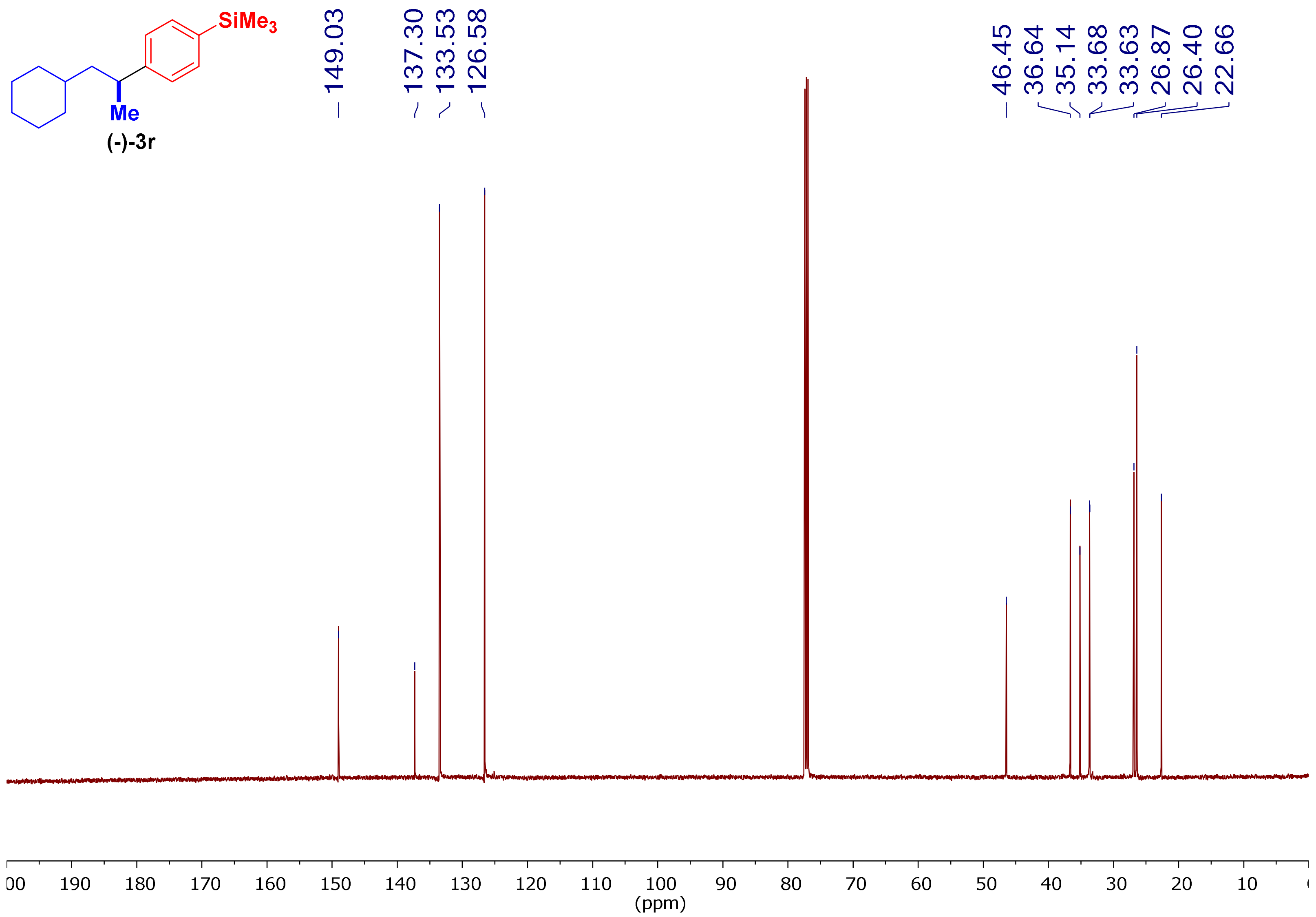

Supplementary Figure 188| ^{13}C-NMR spectrum (126 MHz, $CDCl_3$) for **(-)-3r**.

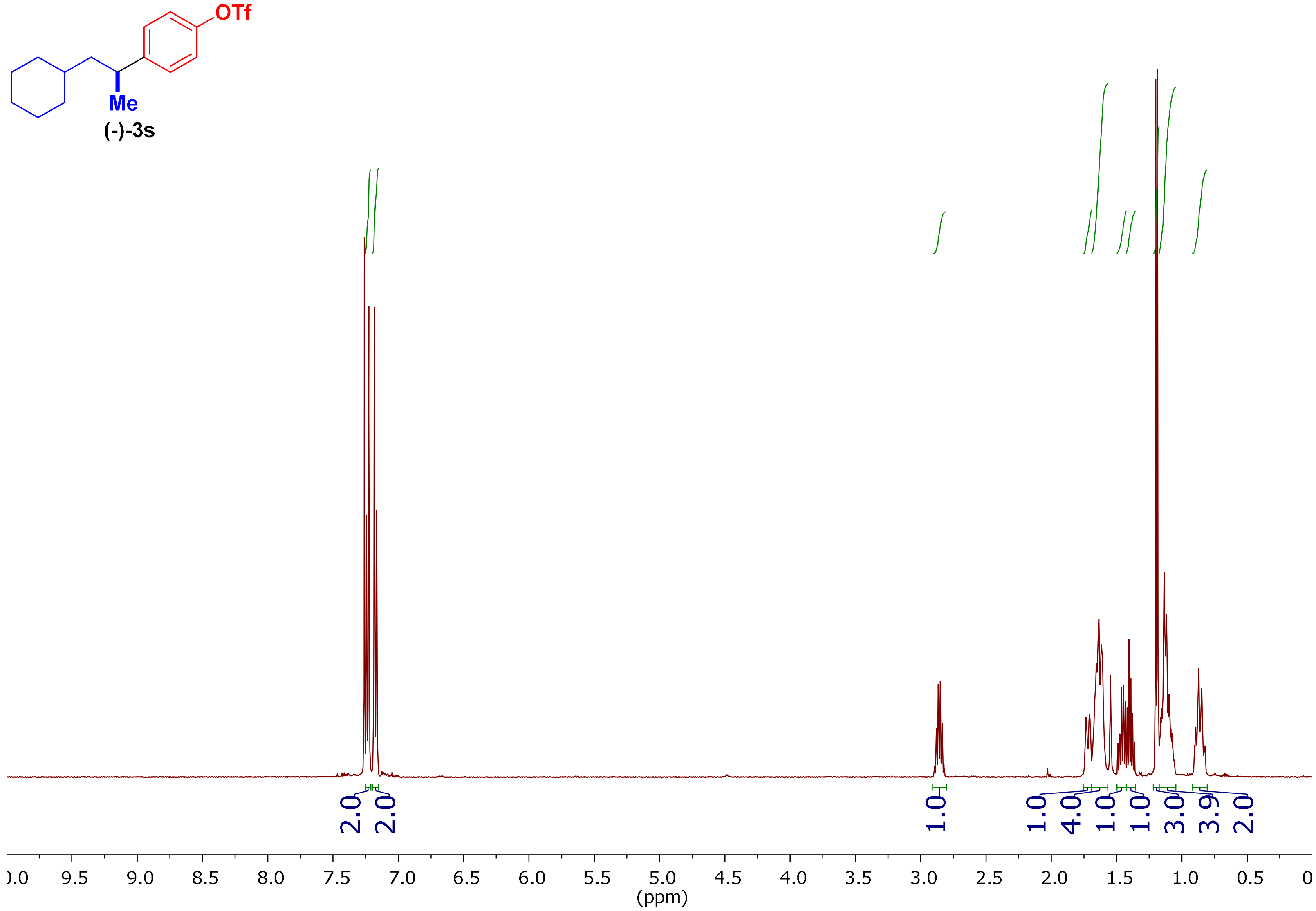

Supplementary Figure 189| ^{1}H-NMR spectrum (500 MHz, $CDCl_3$) for **(-)-3s**.

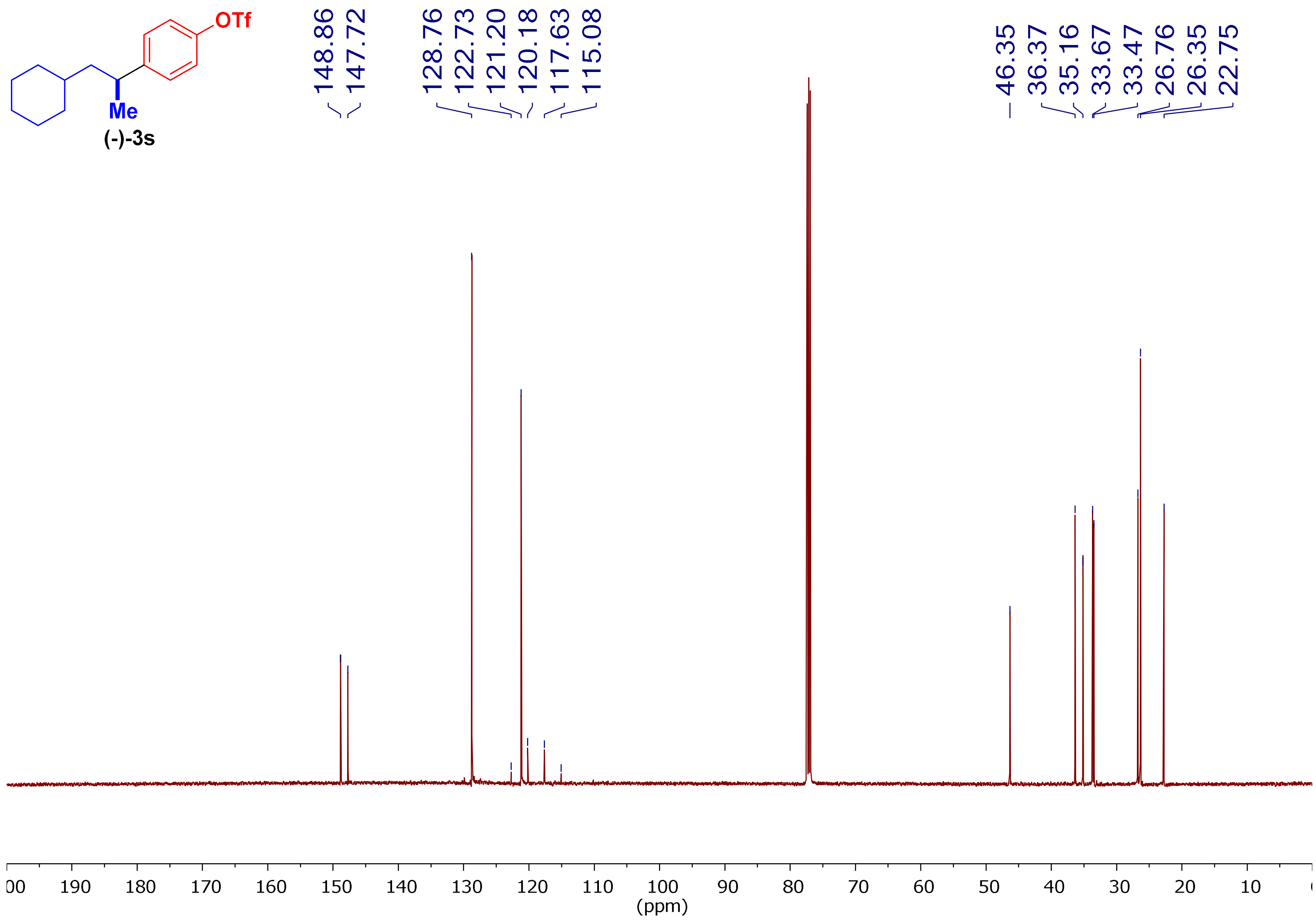

Supplementary Figure 190| ^{13}C-NMR spectrum (126 MHz, $CDCl_3$) for **(-)-3s**.

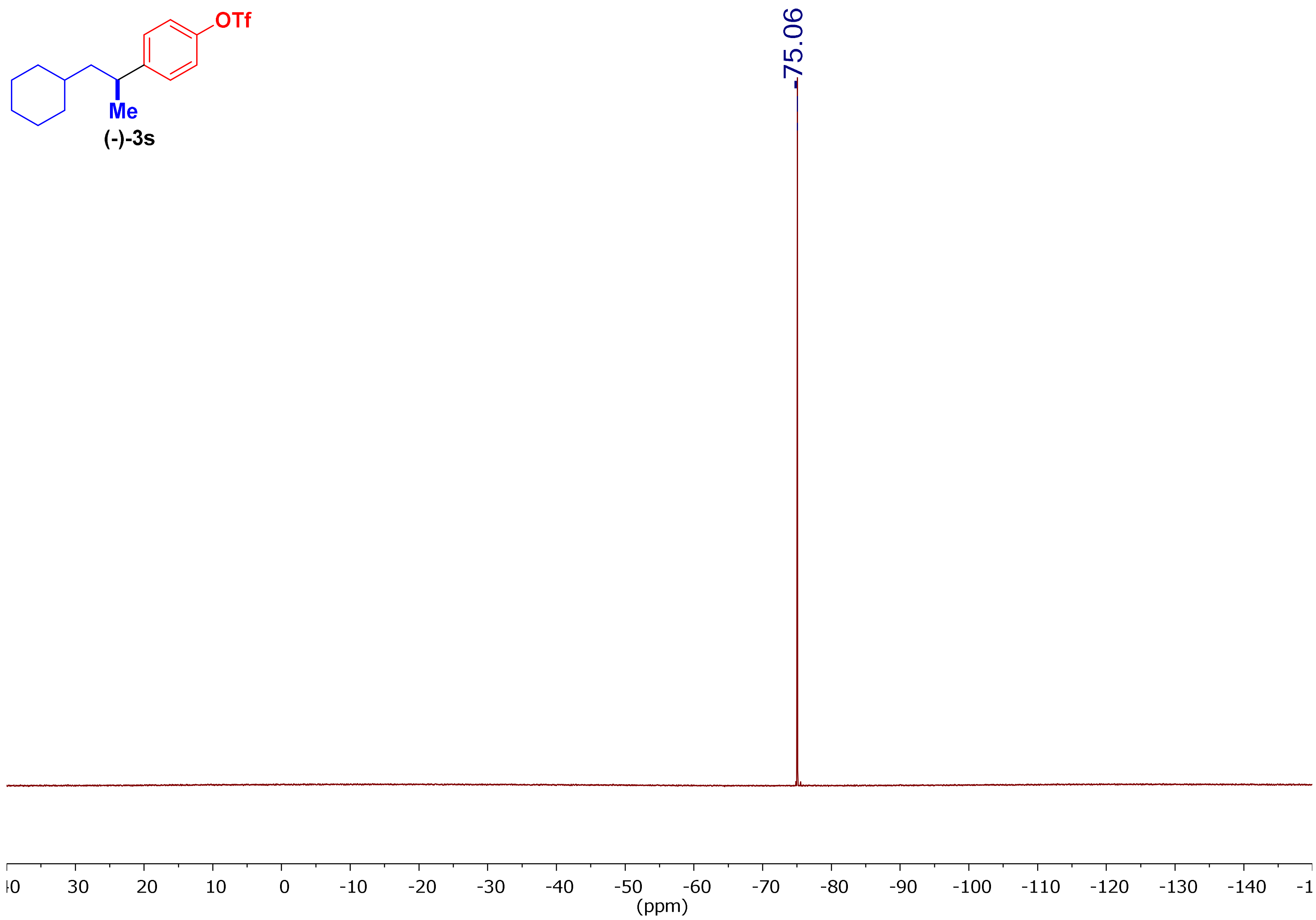

Supplementary Figure 191| ^{19}F-NMR spectrum (470 MHz, $CDCl_3$) for **(-)-3s**.

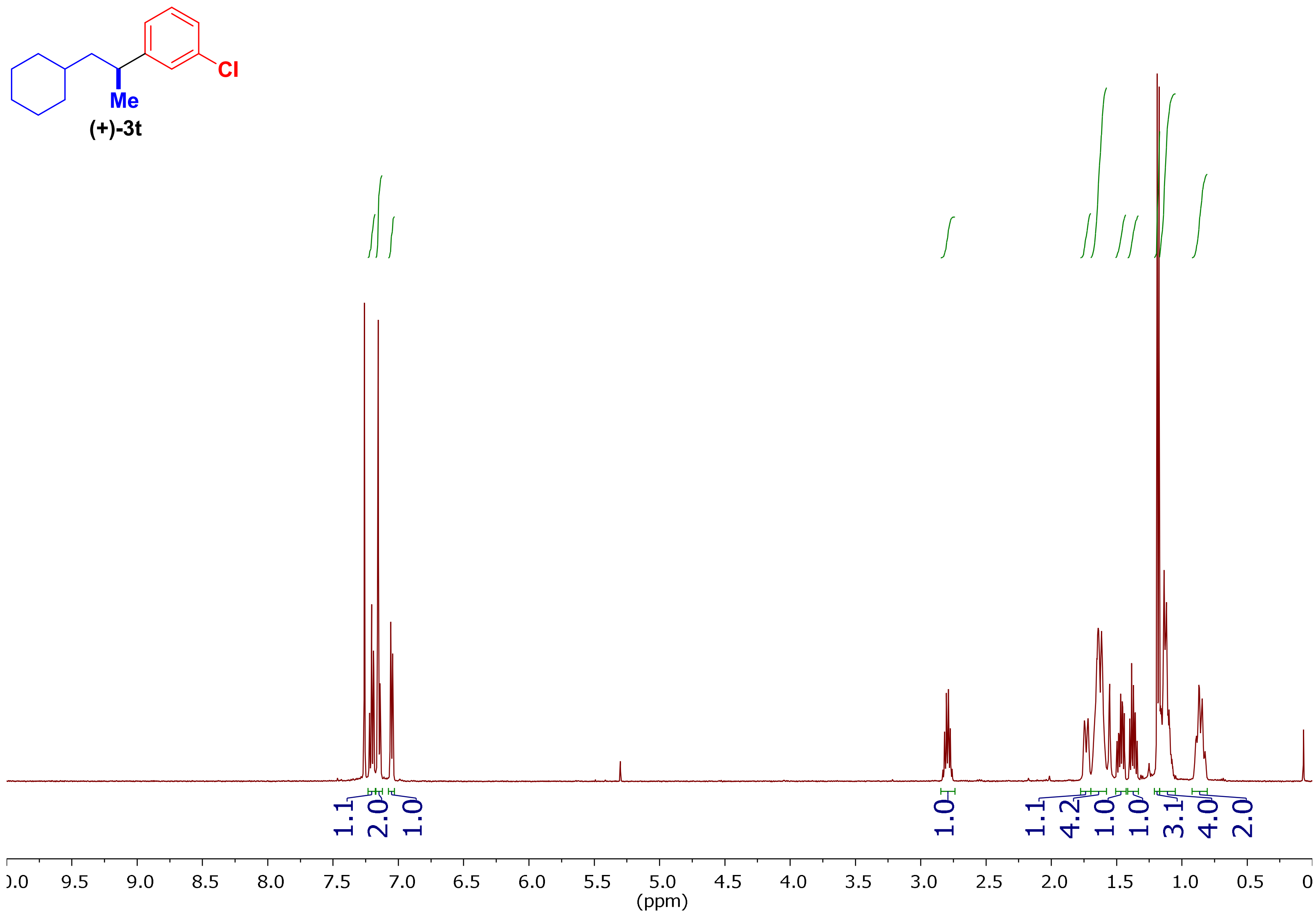

Supplementary Figure 192| ^{1}H-NMR spectrum (500 MHz, $CDCl_3$) for **(+)-3t**.

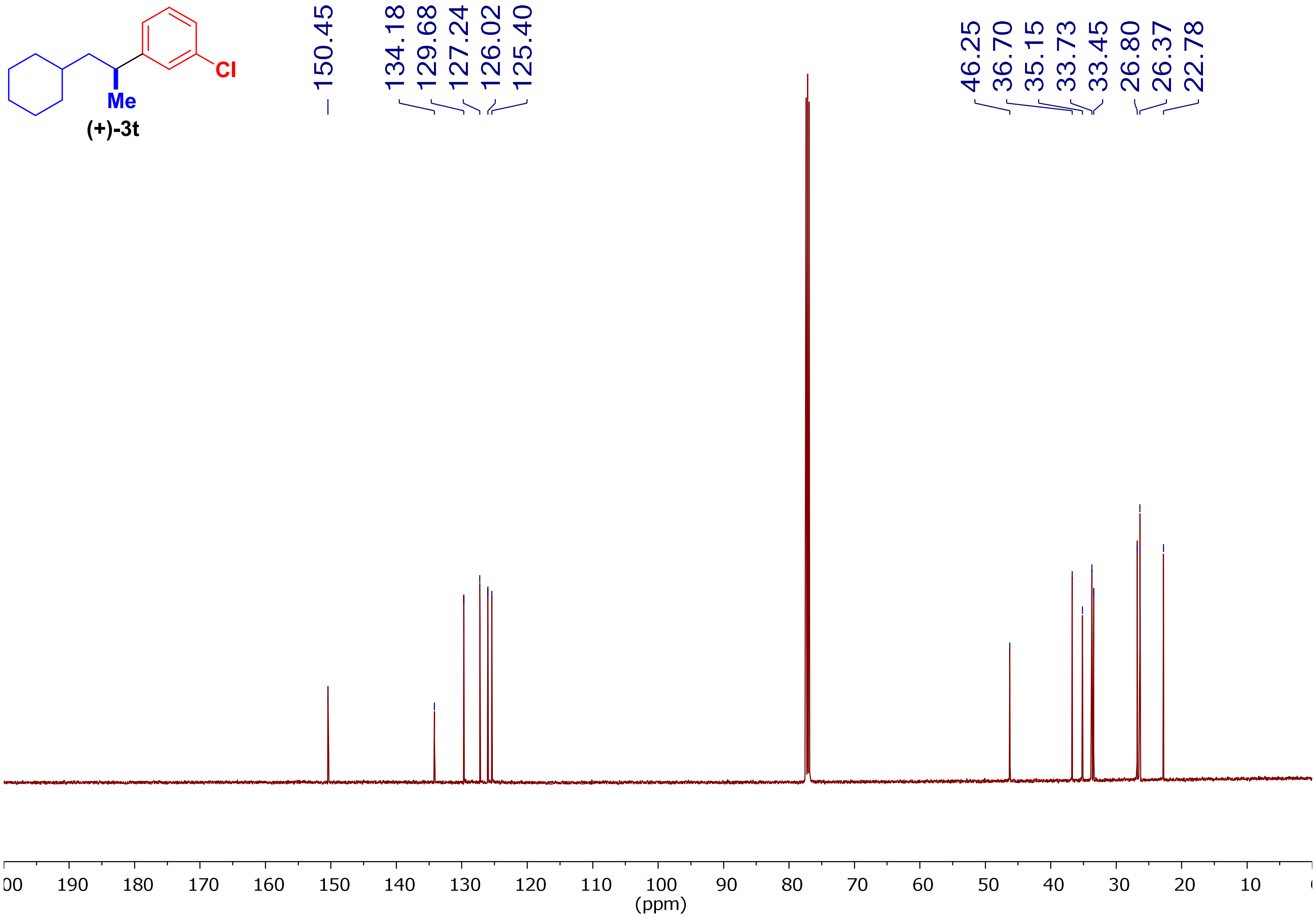

Supplementary Figure 193| ^{13}C-NMR spectrum (126 MHz, $CDCl_3$) for **(+)-3t**.

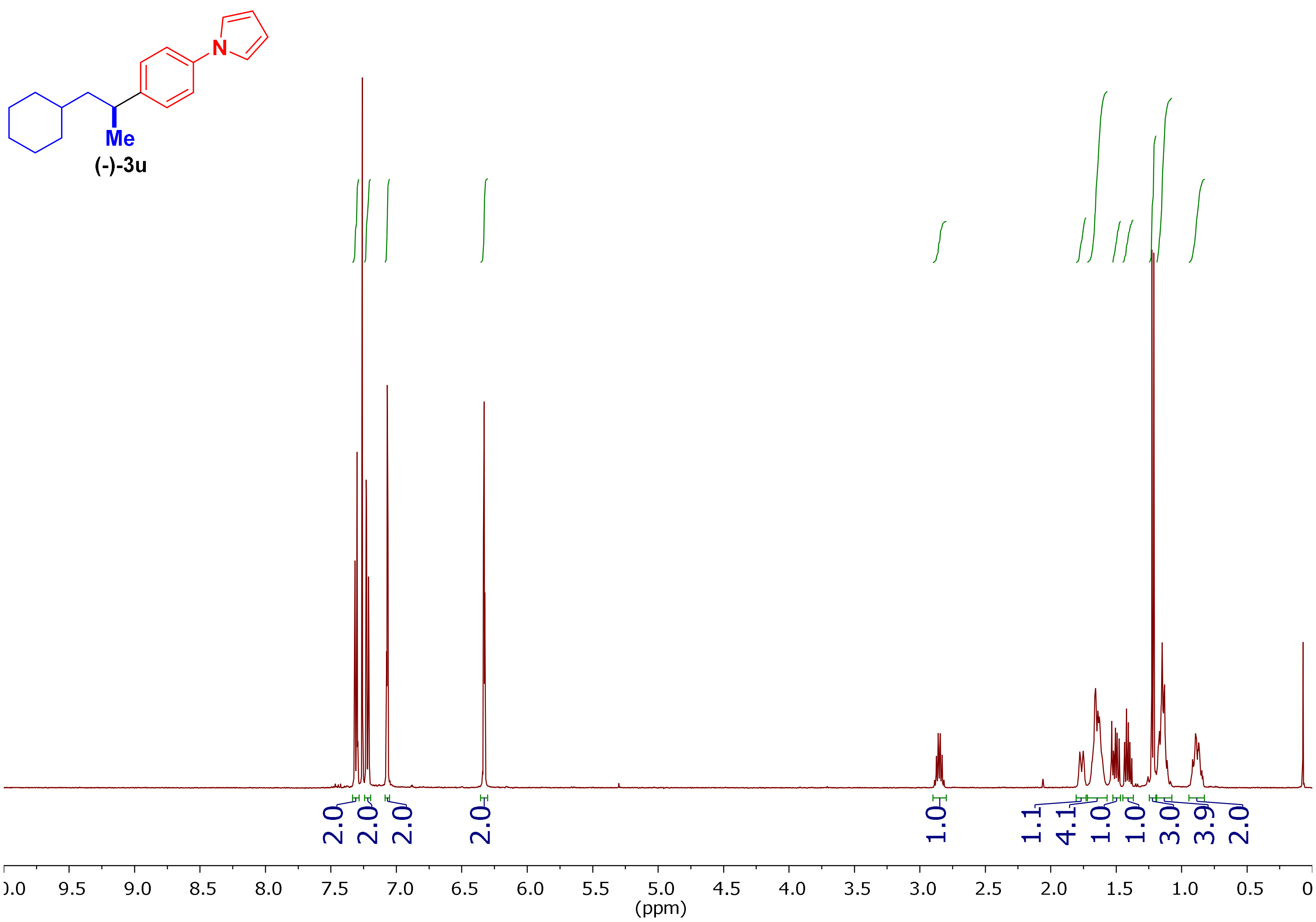

Supplementary Figure 194| ^{1}H-NMR spectrum (500 MHz, $CDCl_3$) for **(-)-3u**.

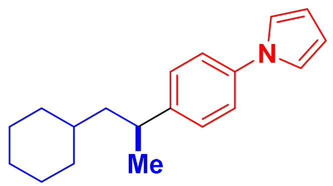

(-)-3u

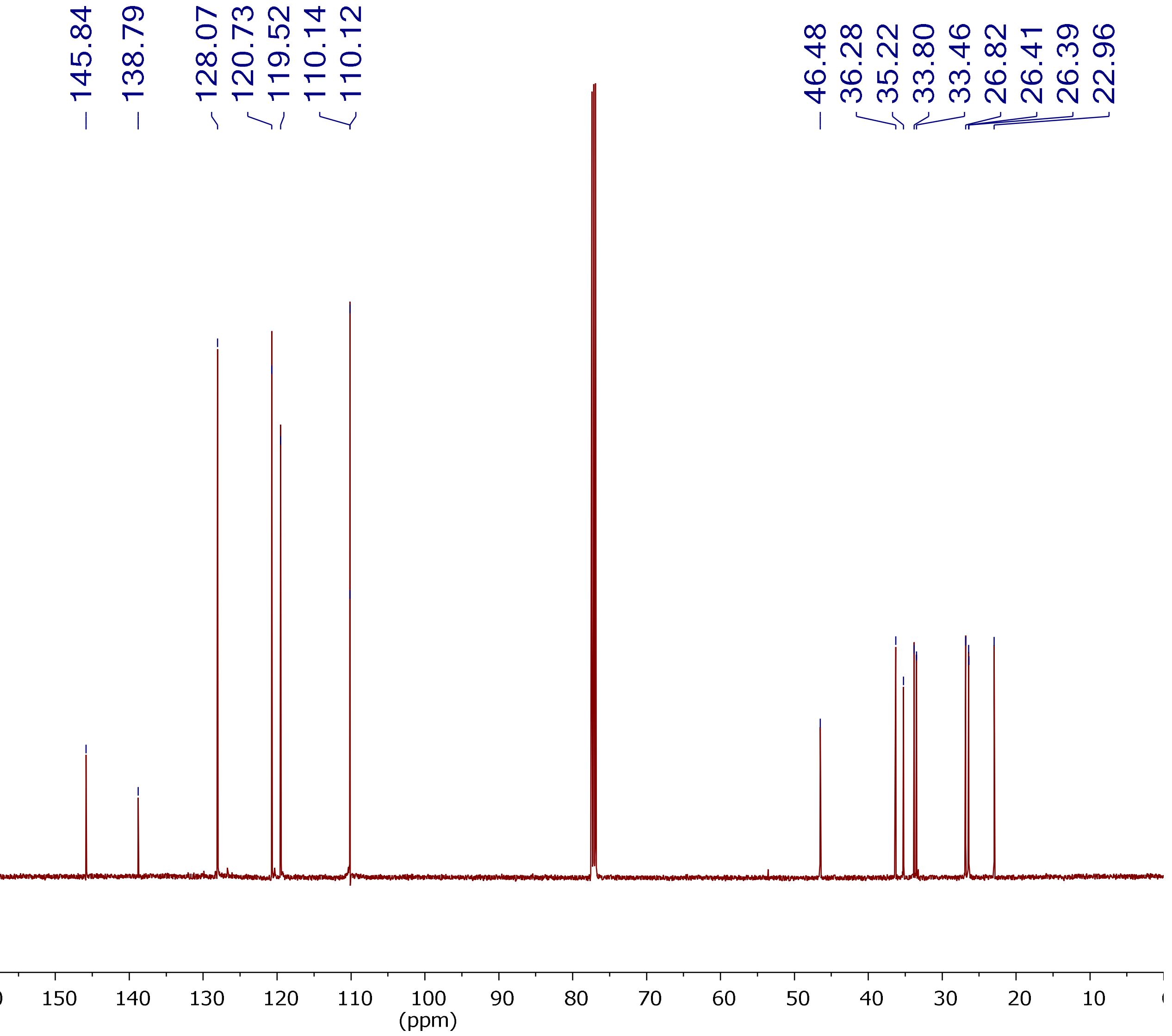

Supplementary Figure 195| ^{13}C-NMR spectrum (126 MHz, $CDCl_3$) for **(-)-3u**.

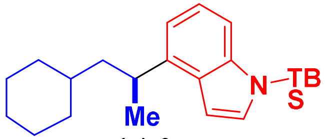

(+)-3v

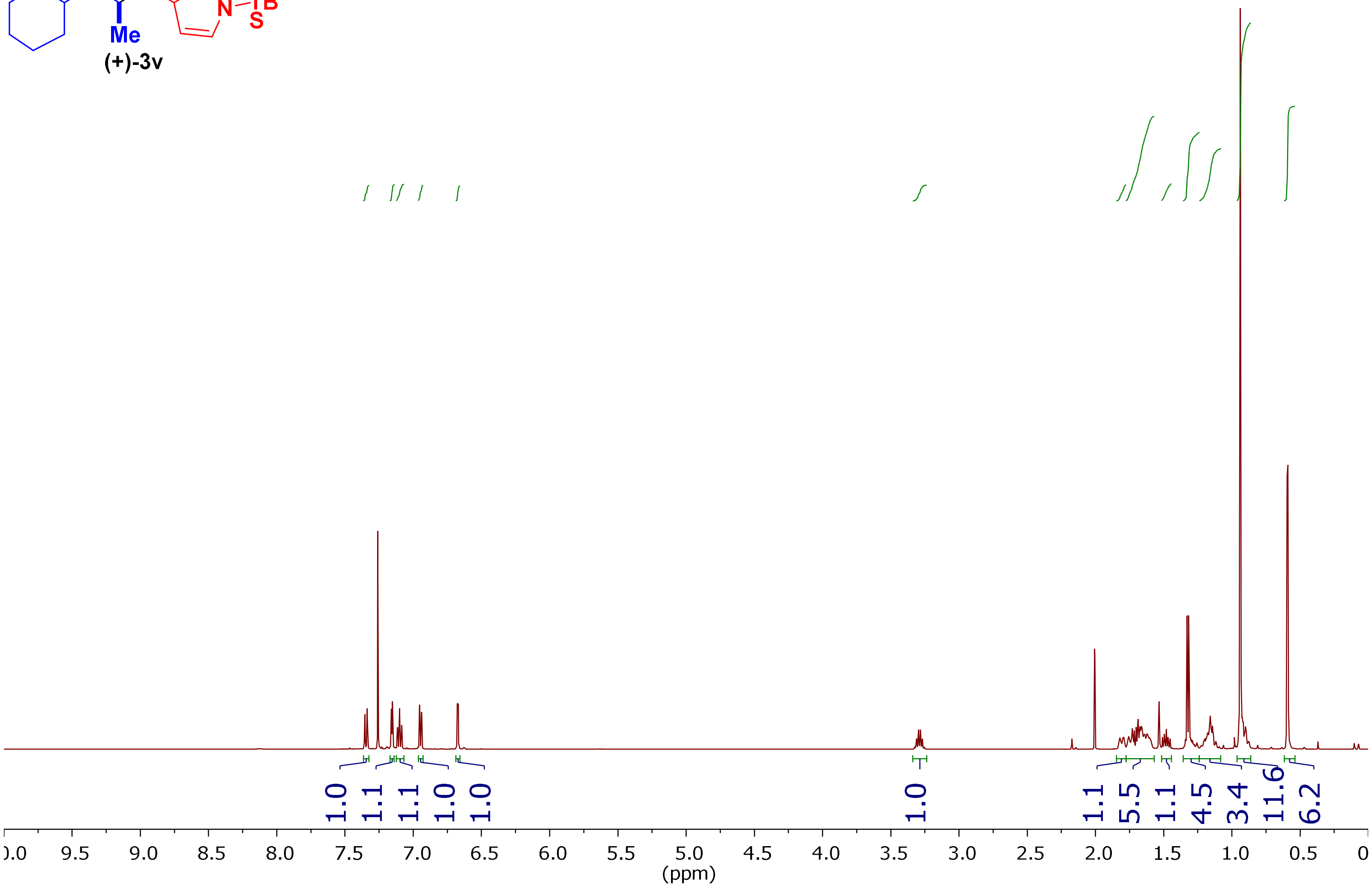

Supplementary Figure 196| ^{1}H-NMR spectrum (500 MHz, $CDCl_3$) for **(+)-3v**.

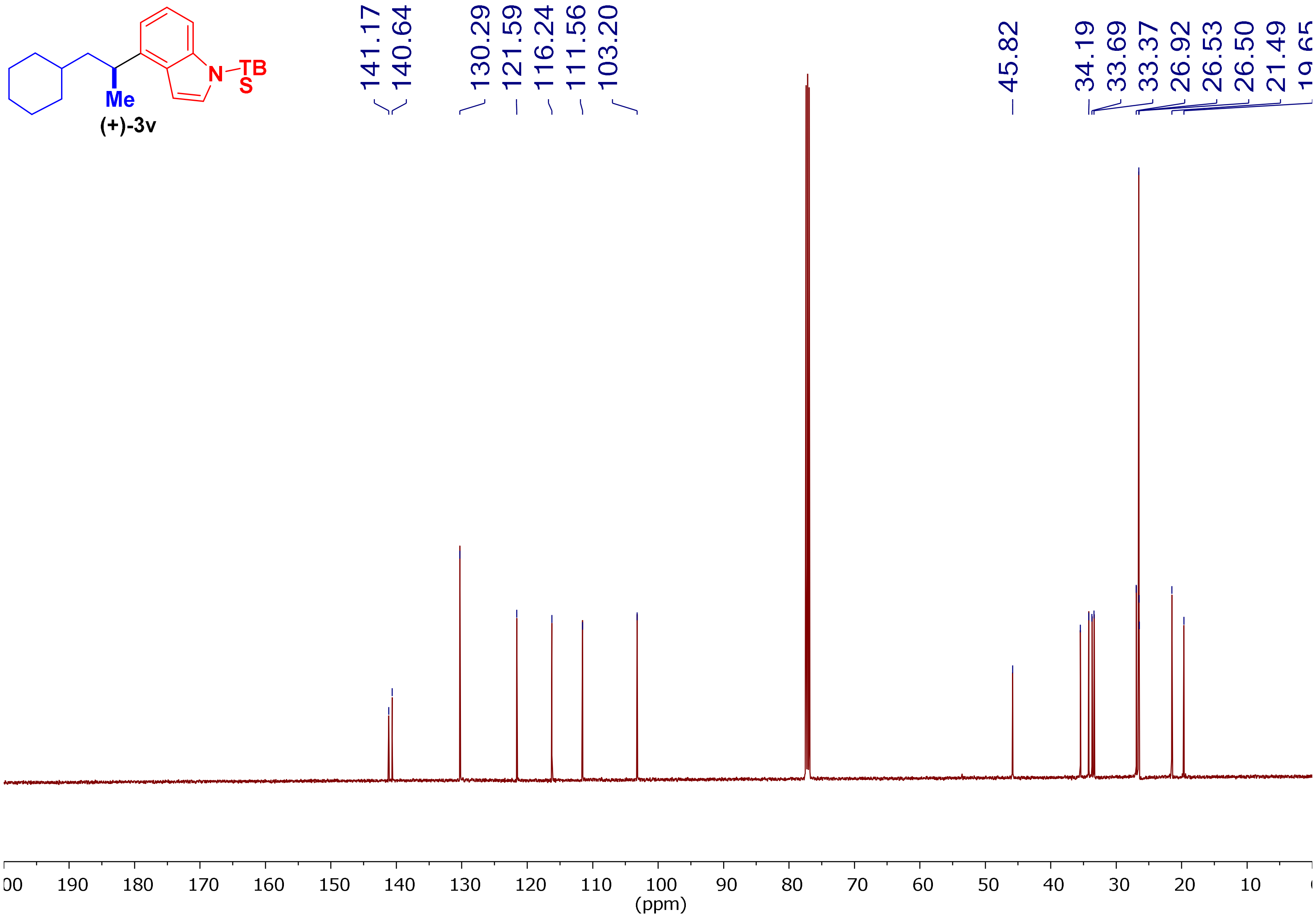

Supplementary Figure 197| ^{13}C-NMR spectrum (126 MHz, $CDCl_3$) for **(+)-3v**.

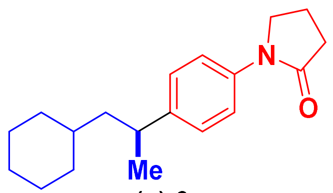

(+)-3w

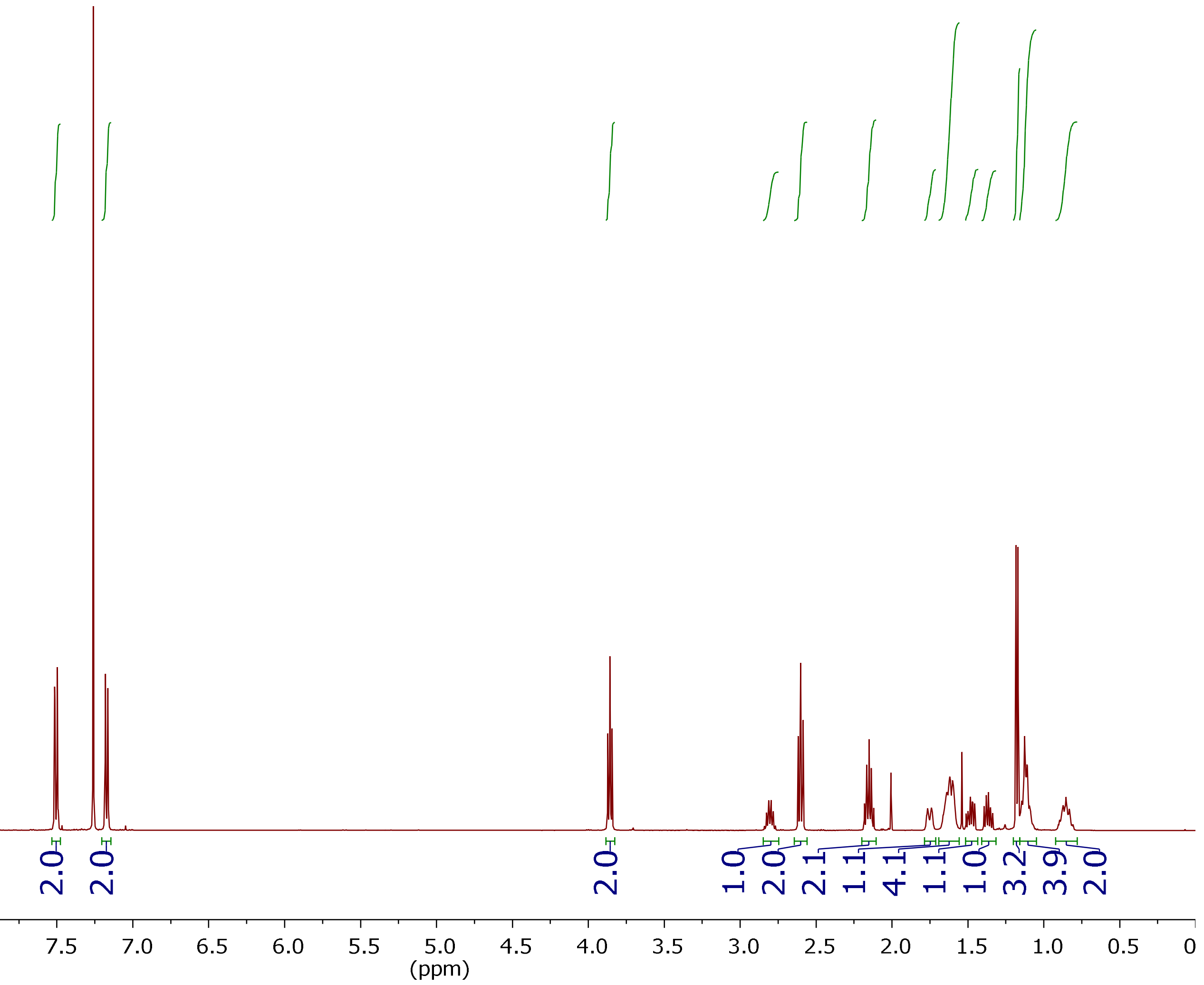

Supplementary Figure 198| ^{1}H-NMR spectrum (500 MHz, $CDCl_3$) for **(+)-3w**.

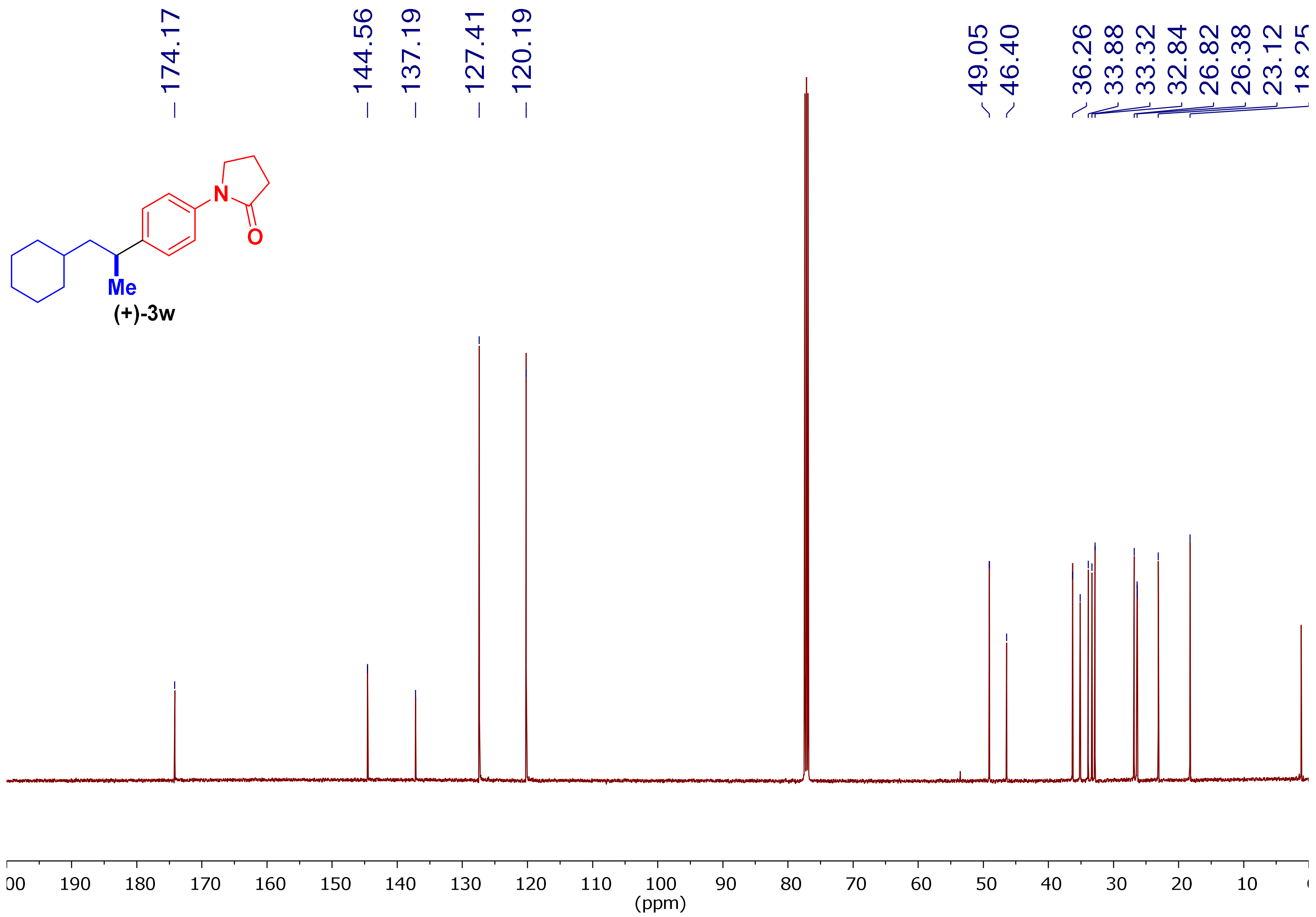

Supplementary Figure 199| ^{13}C-NMR spectrum (126 MHz, $CDCl_3$) for **(+)-3w**.

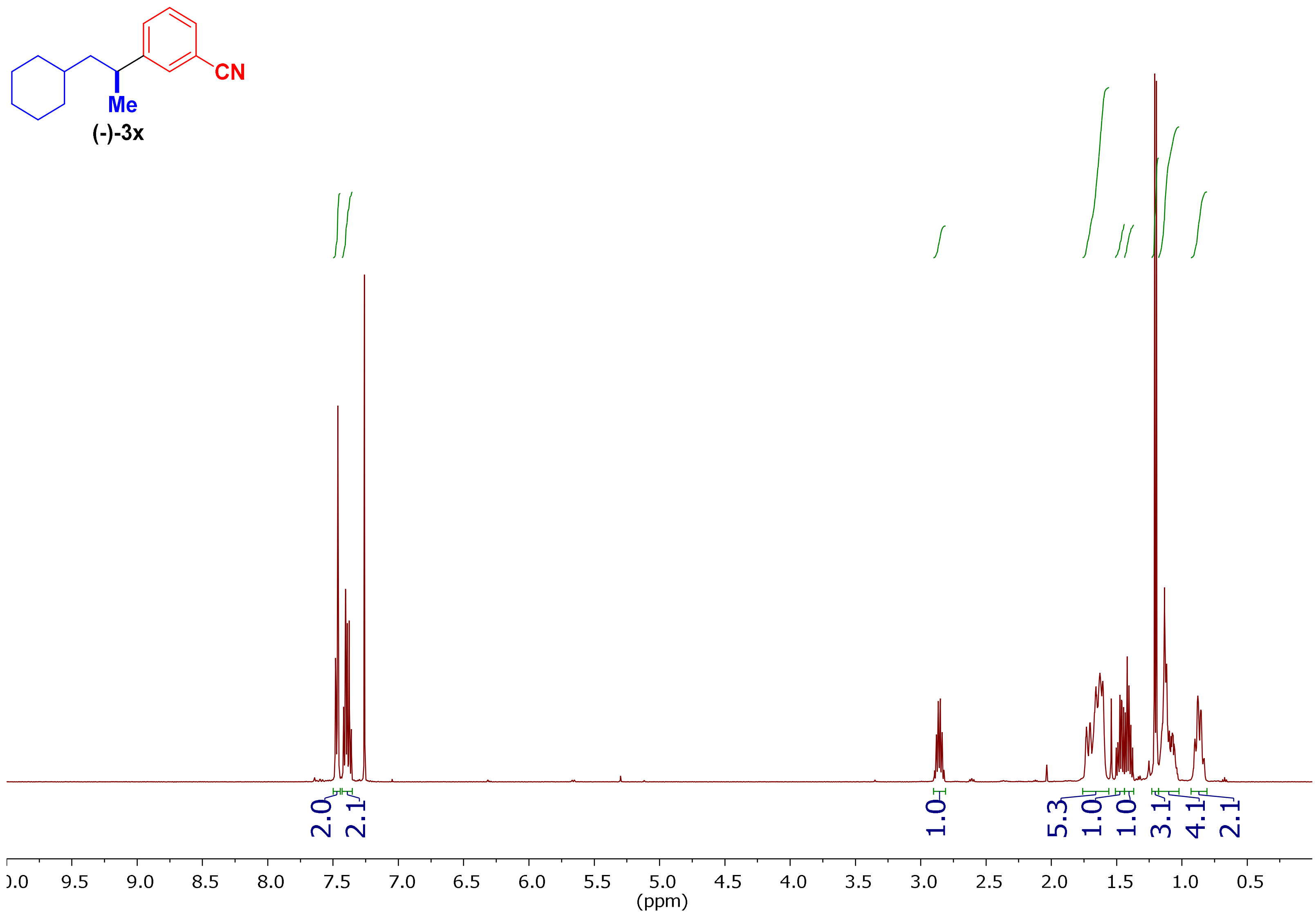

Supplementary Figure 200| ^{1}H-NMR spectrum (500 MHz, $CDCl_3$) for **(-)-3x**.

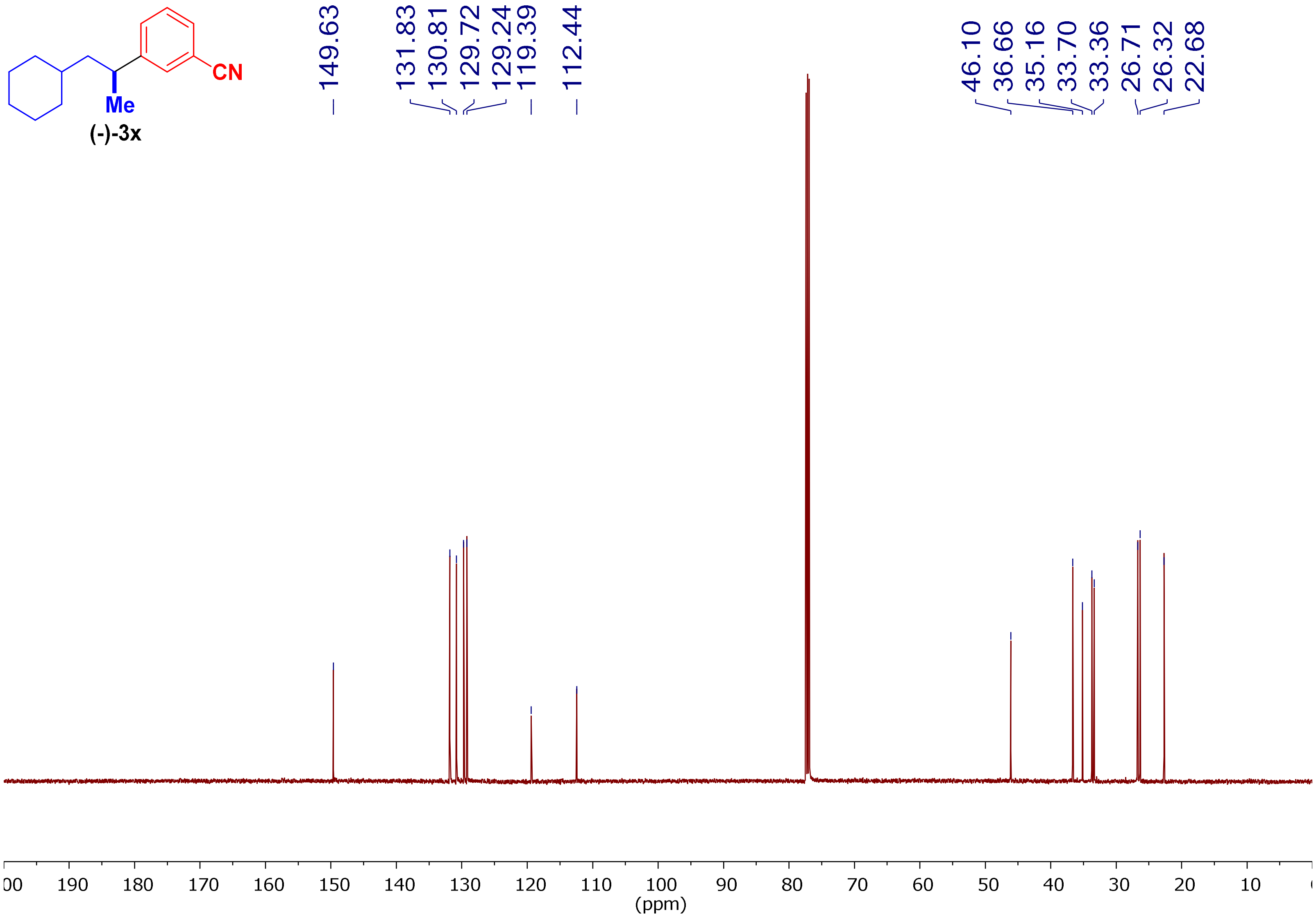

Supplementary Figure 201| ^{13}C-NMR spectrum (126 MHz, $CDCl_3$) for **(-)-3x**.

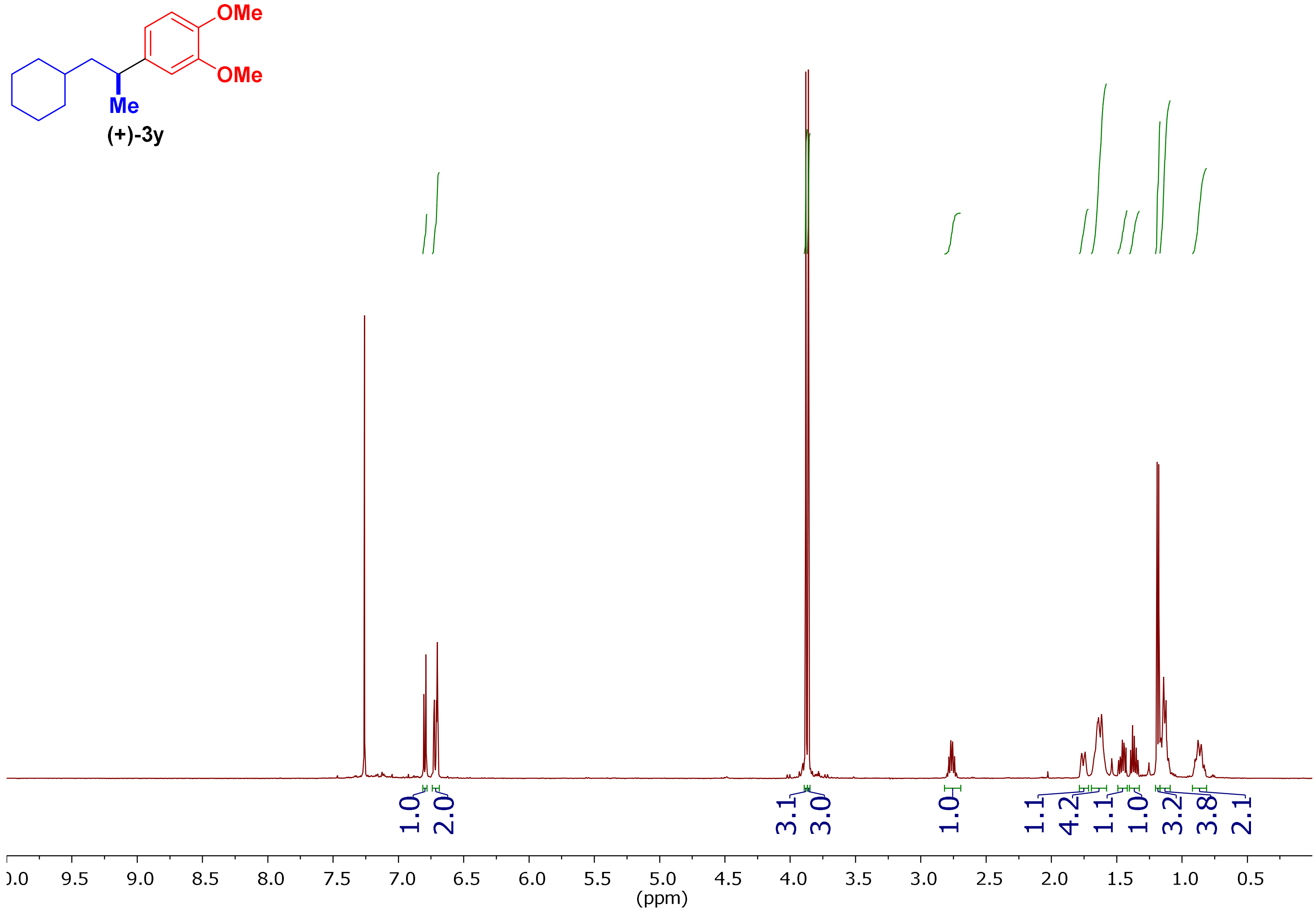

Supplementary Figure 202| ^{1}H-NMR spectrum (500 MHz, $CDCl_3$) for **(+)-3y**.

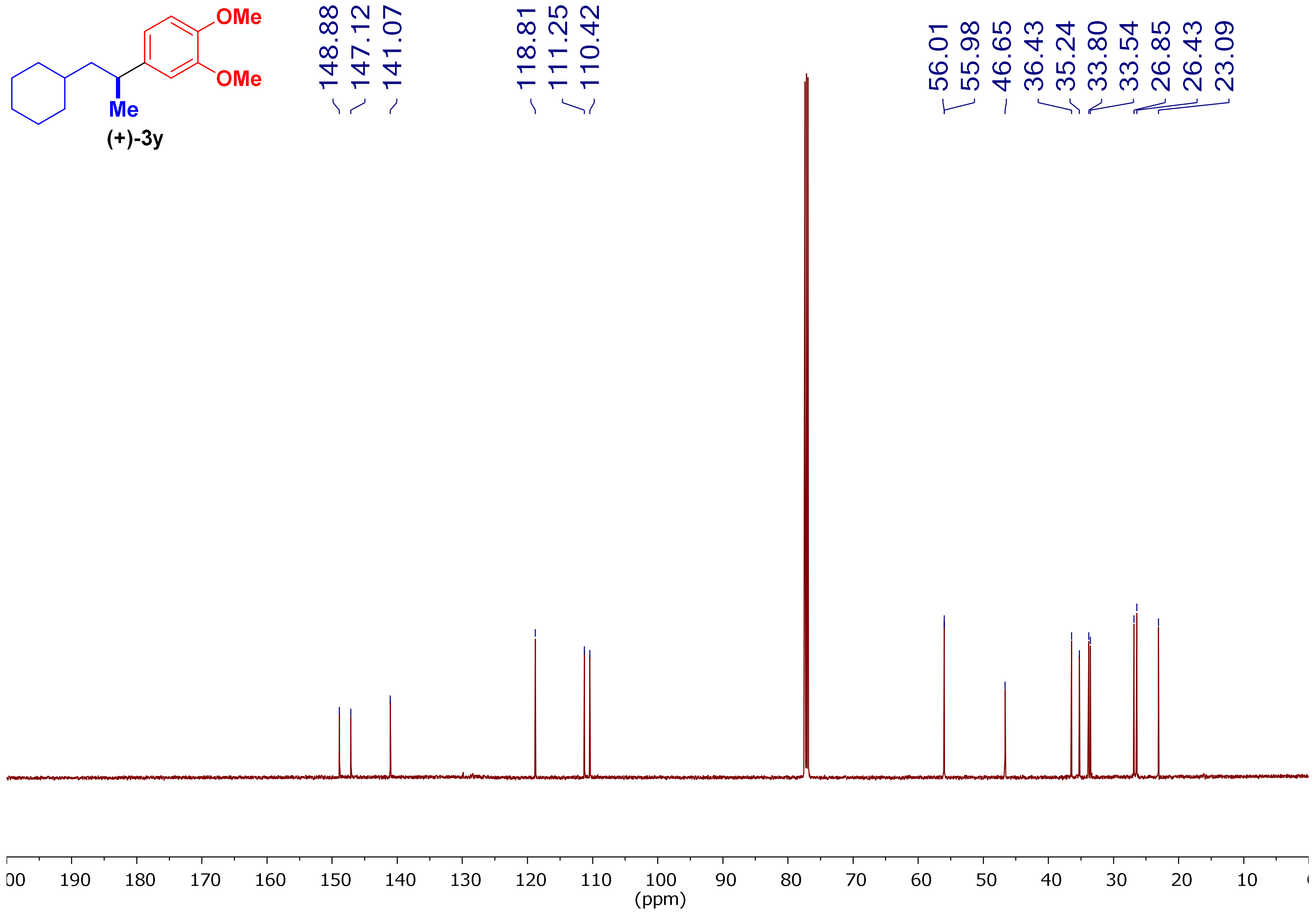

Supplementary Figure 203| ^{13}C-NMR spectrum (126 MHz, $CDCl_3$) for **(+)-3y**.

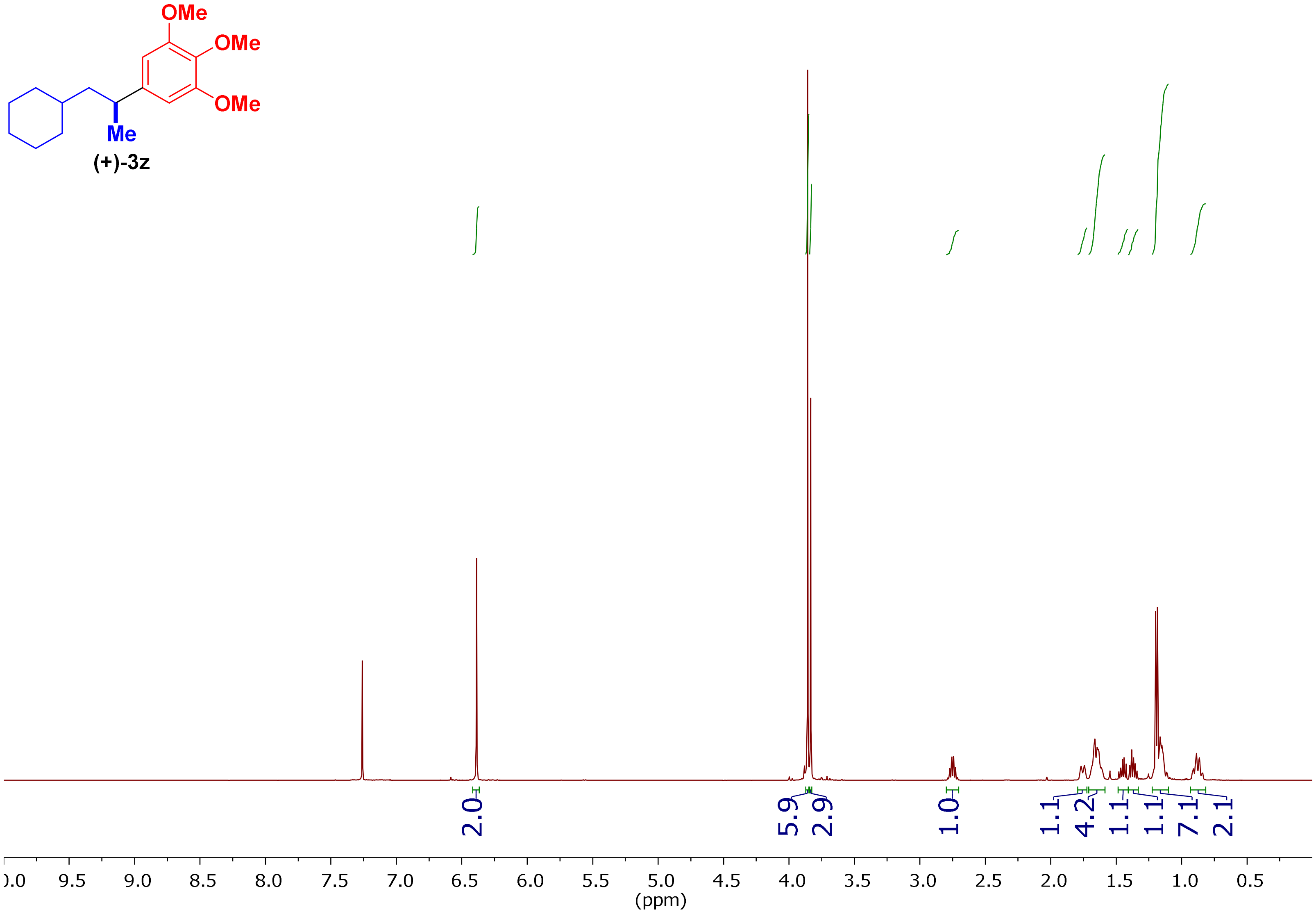

Supplementary Figure 204| ^{1}H-NMR spectrum (500 MHz, $CDCl_3$) for **(+)-3z**.

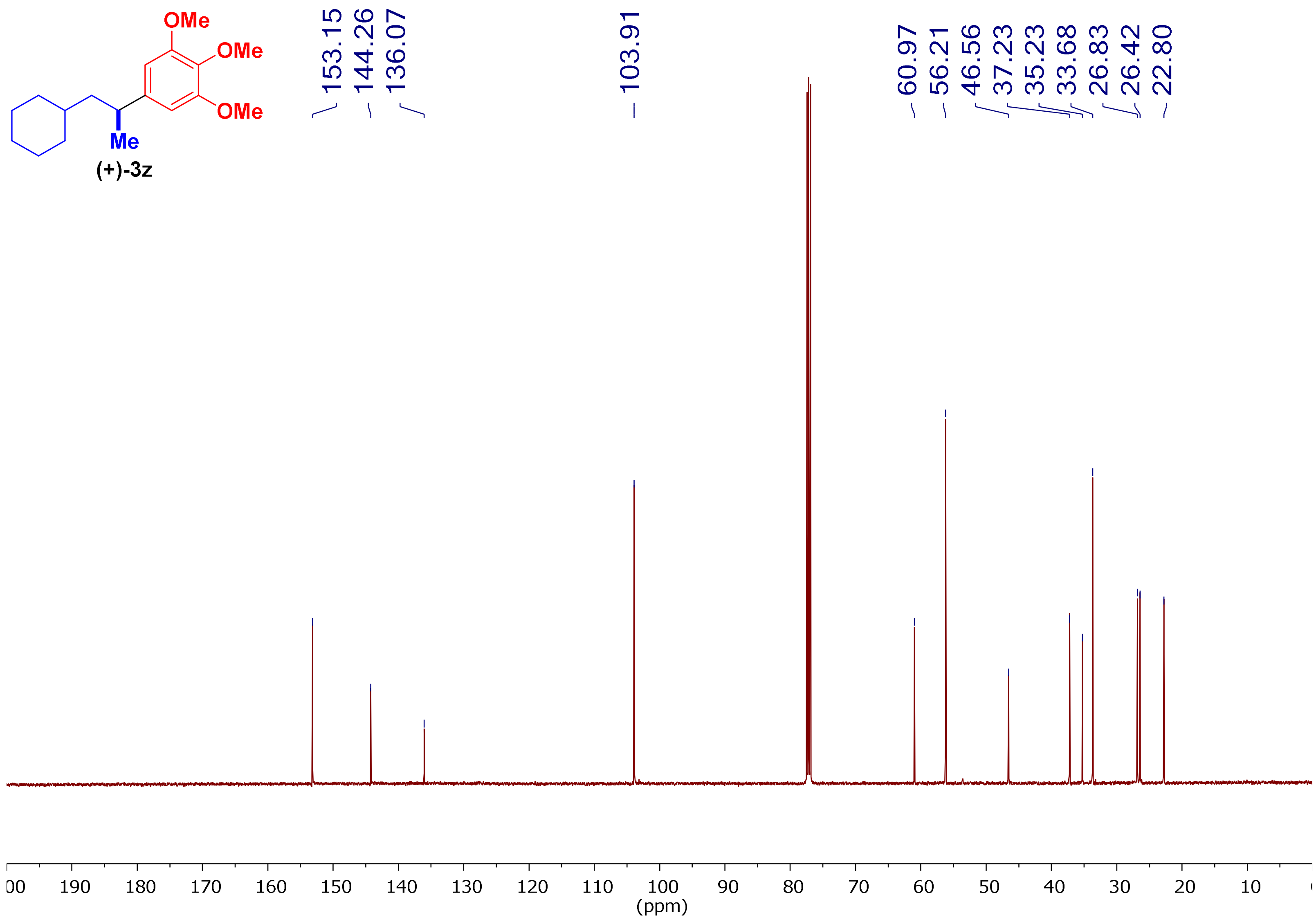

Supplementary Figure 205| ^{13}C-NMR spectrum (126 MHz, $CDCl_3$) for **(+)-3z**.

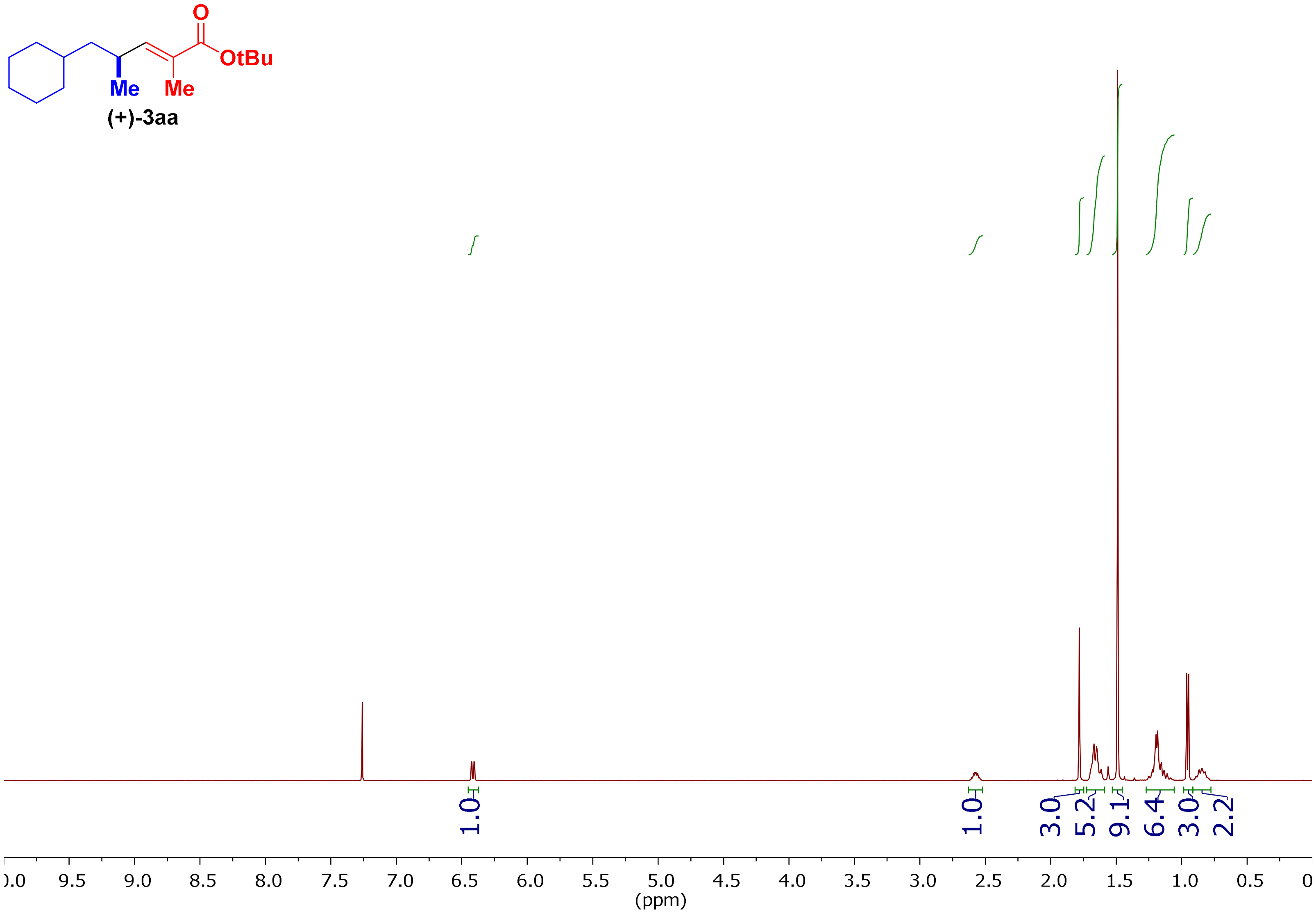

Supplementary Figure 206| ^{1}H-NMR spectrum (500 MHz, $CDCl_3$) for **(+)-3aa**.

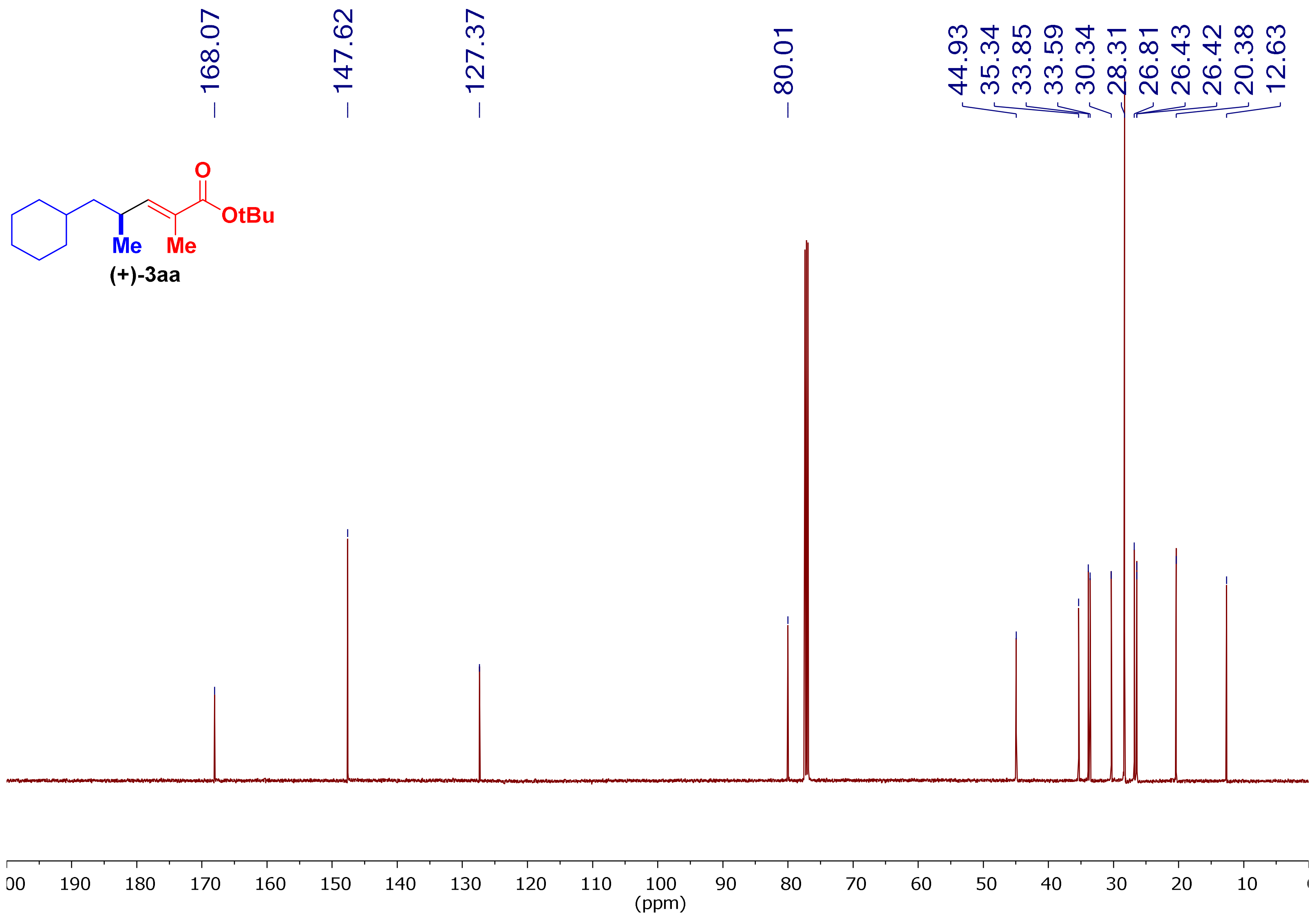

Supplementary Figure 207| ^{13}C-NMR spectrum (126 MHz, $CDCl_3$) for **(+)-3aa**.

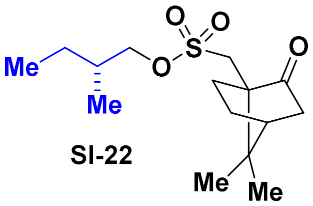

SI-22

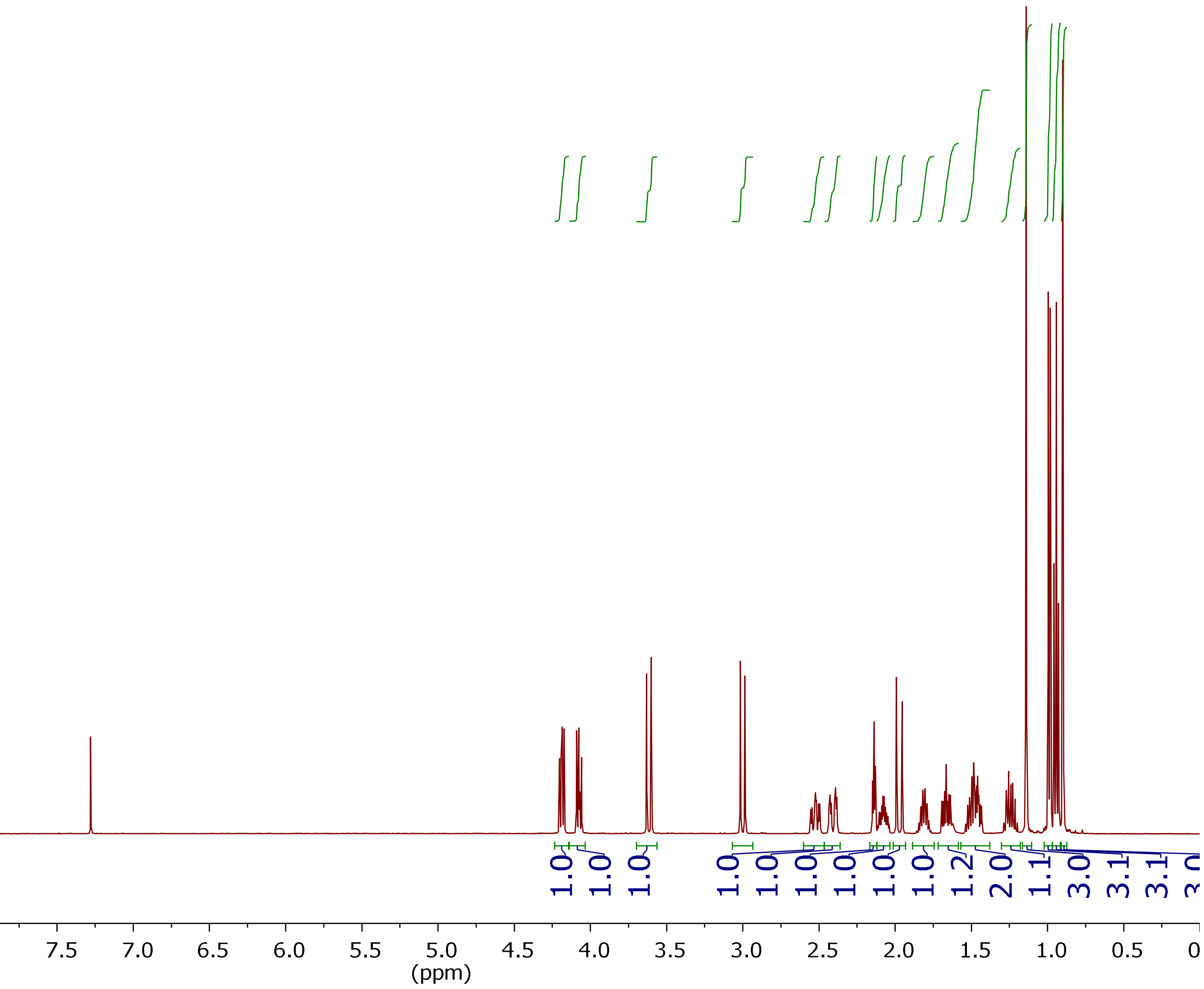

Supplementary Figure 208| ^{1}H-NMR spectrum (500 MHz, $CDCl_3$) for **SI-22**.

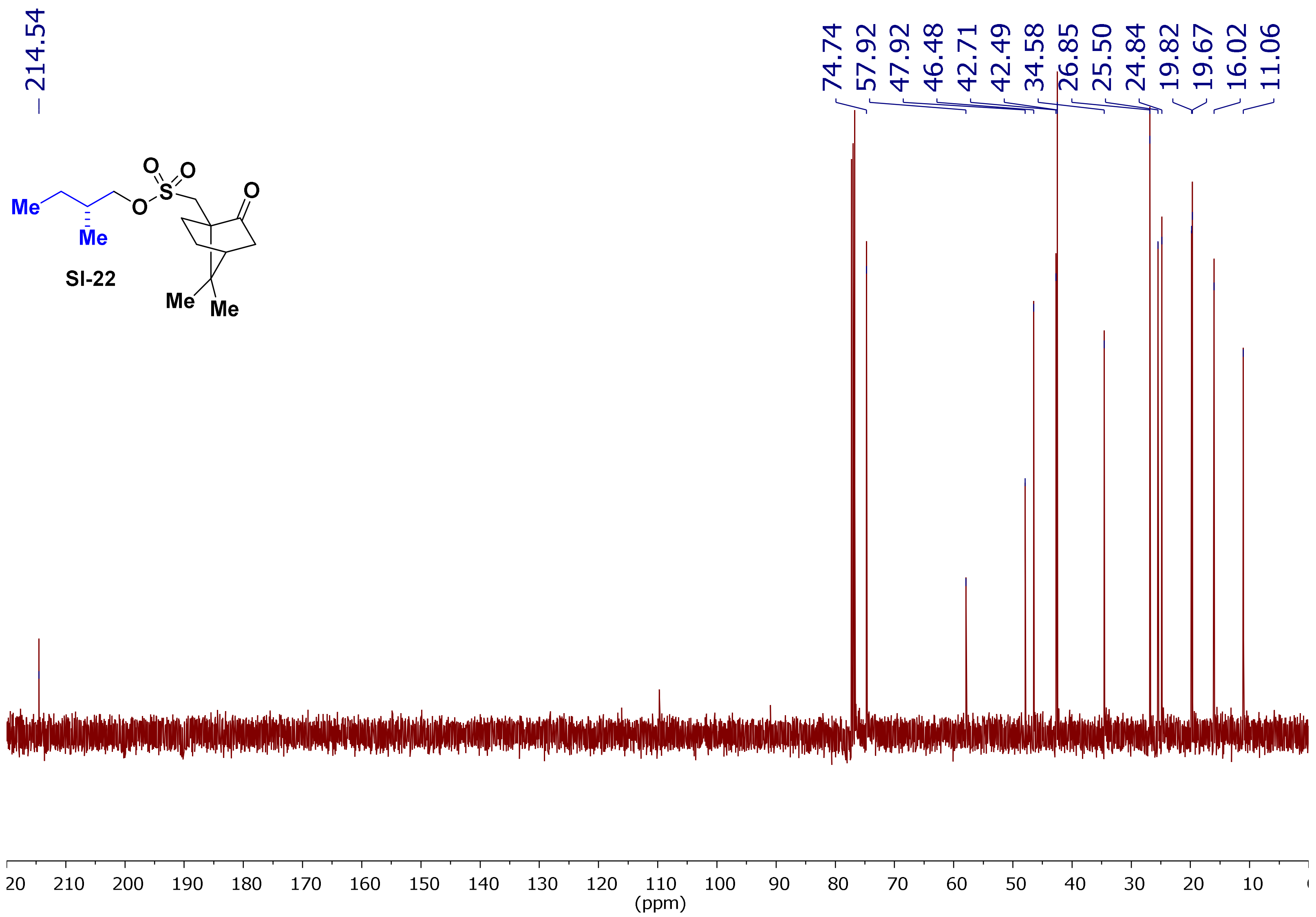

Supplementary Figure 209| ^{13}C-NMR spectrum (126 MHz, $CDCl_3$) for **SI-22**.

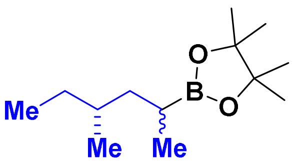

(4*R*)-SI-24

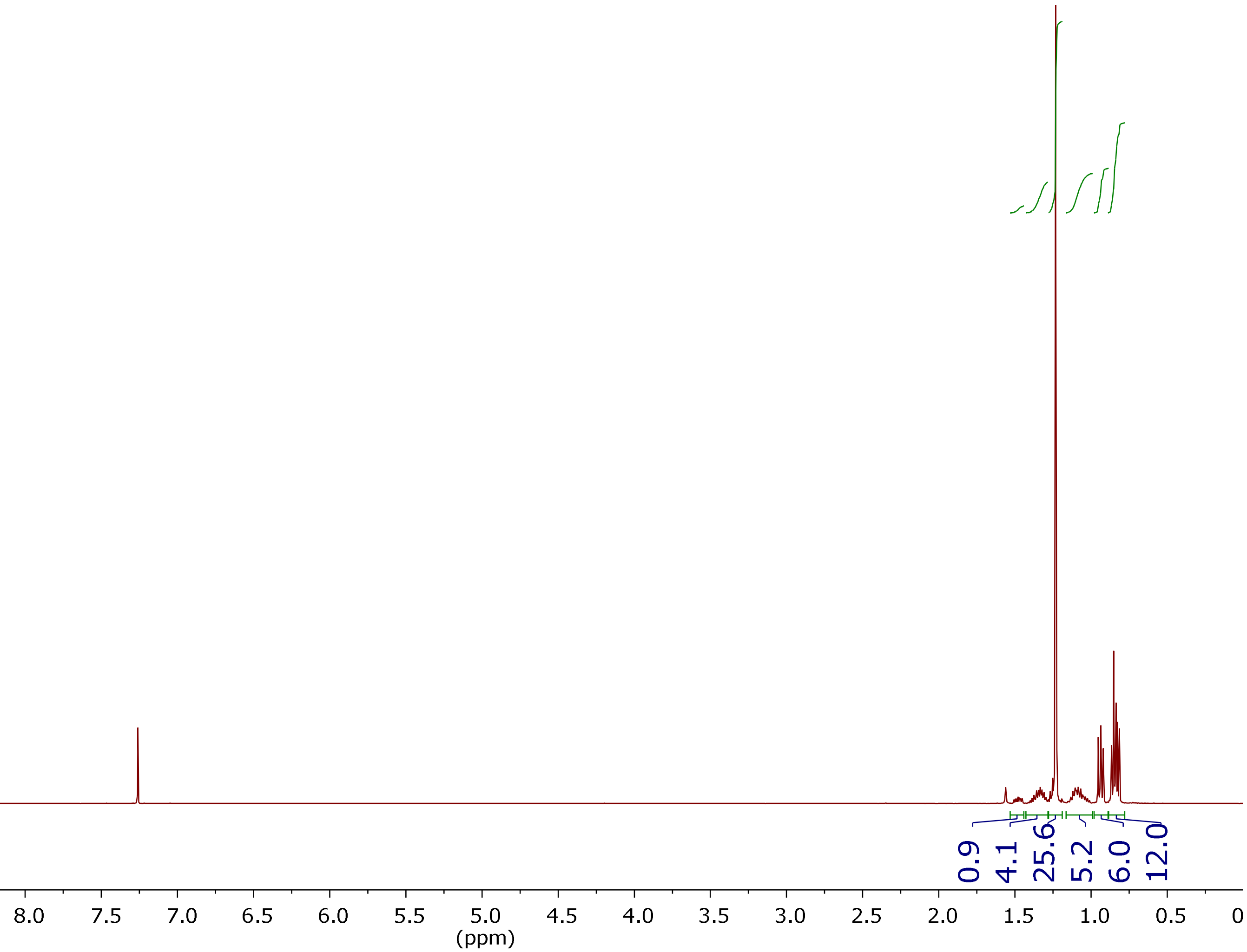

Supplementary Figure 210| ^{1}H-NMR spectrum (500 MHz, $CDCl_3$) for **(4*R*)-SI-24**.

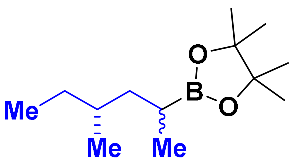

(4*R*)-SI-24

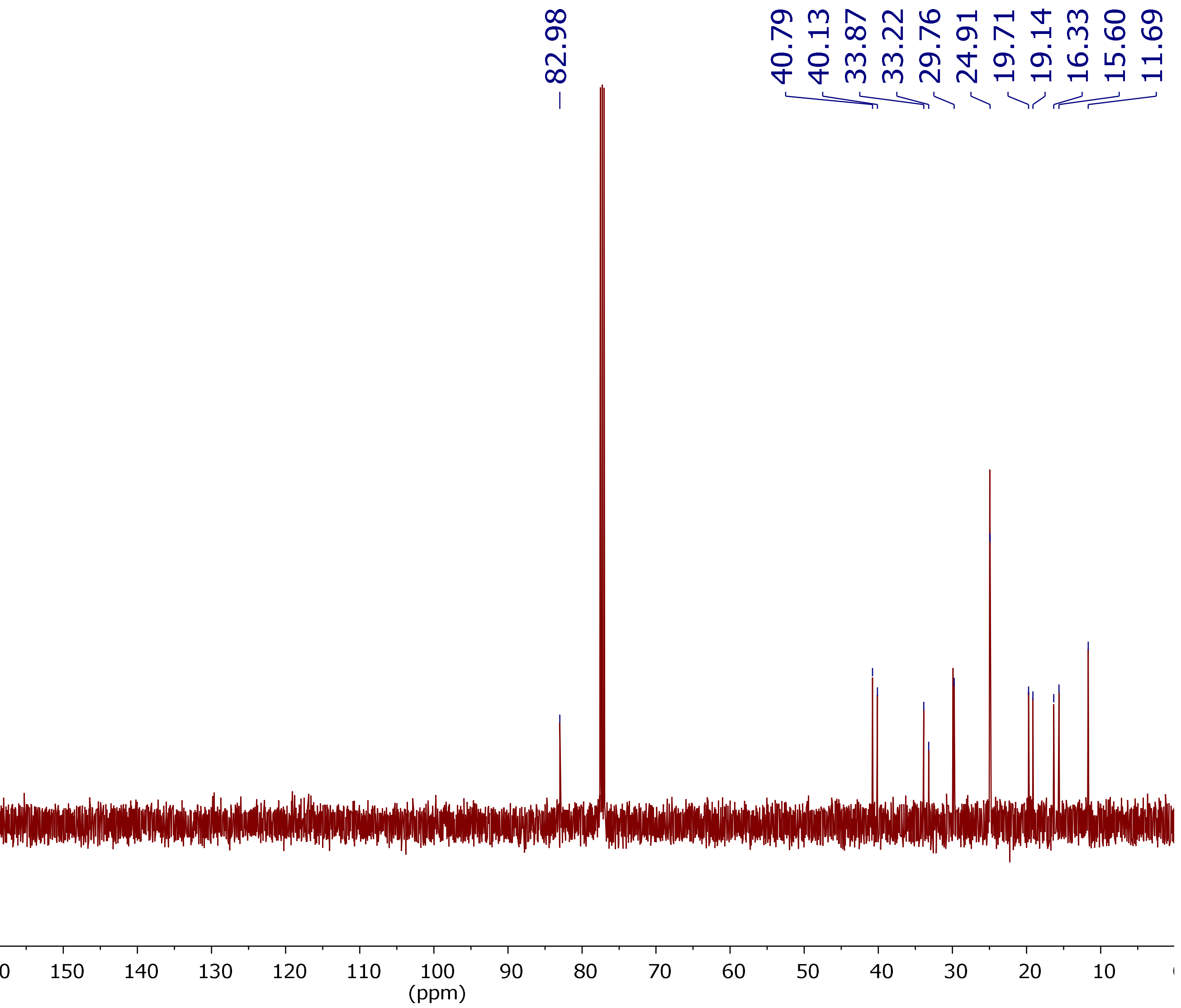

Supplementary Figure 211| ^{13}C-NMR spectrum (126 MHz, $CDCl_3$) for **(4*R*)-SI-24**.

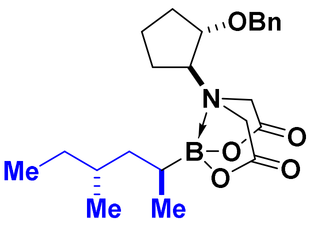

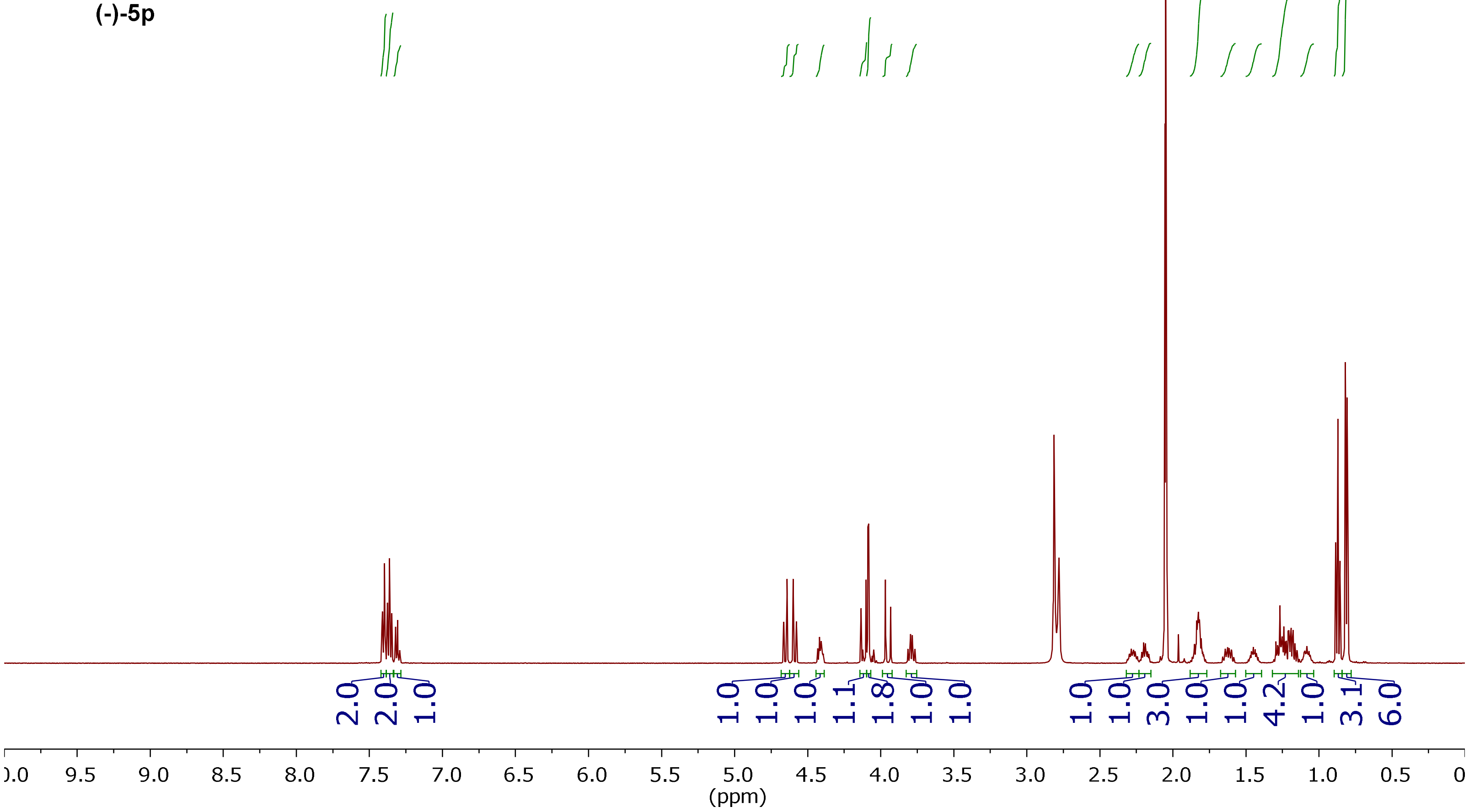

Supplementary Figure 212| ^{1}H-NMR spectrum (500 MHz, acetone-*d6*) for **(-)-5p**.

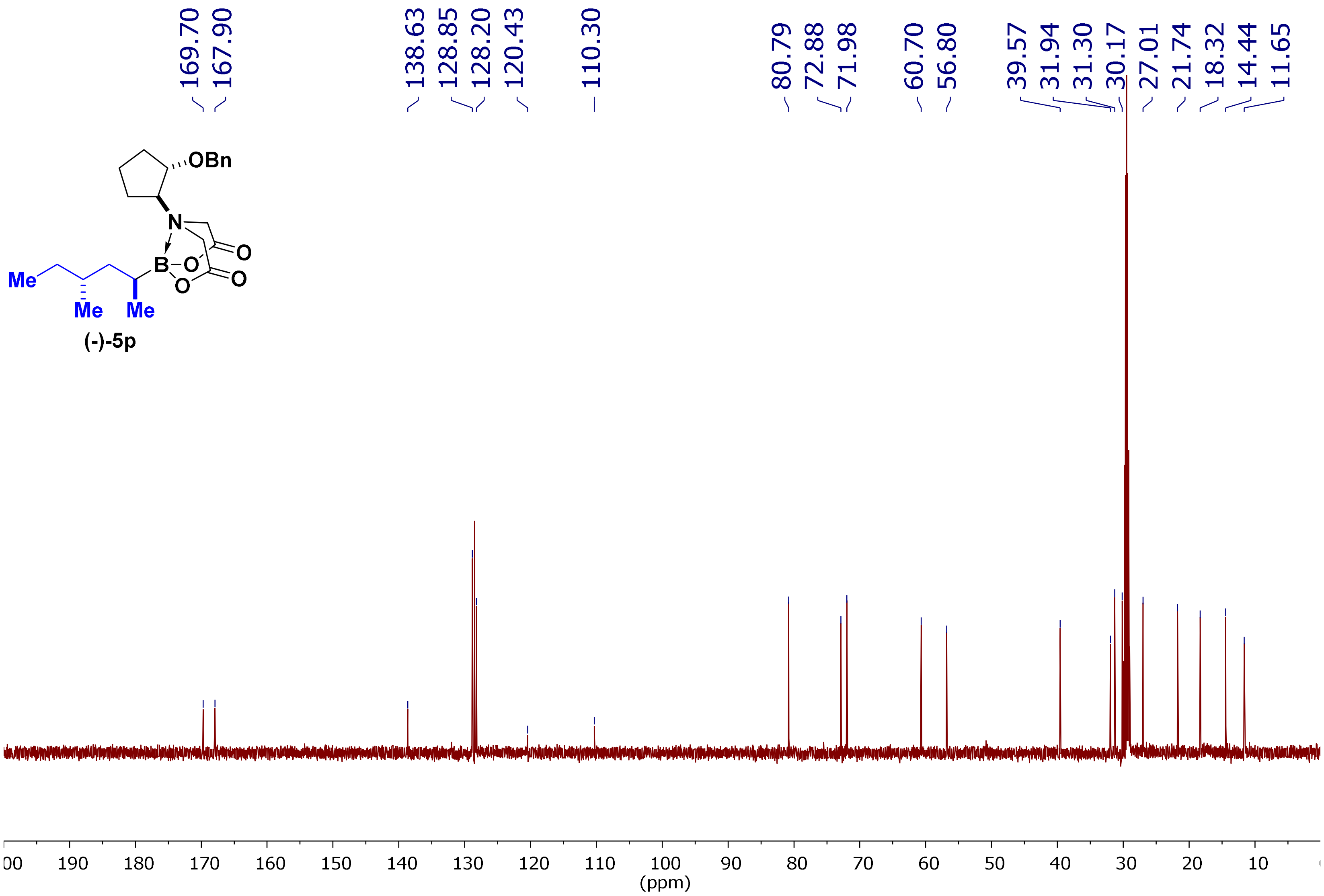

(-)-5p

Supplementary Figure 213| ^{13}C-NMR spectrum (126 MHz, acetone-*d6*) for **(-)-5p**.

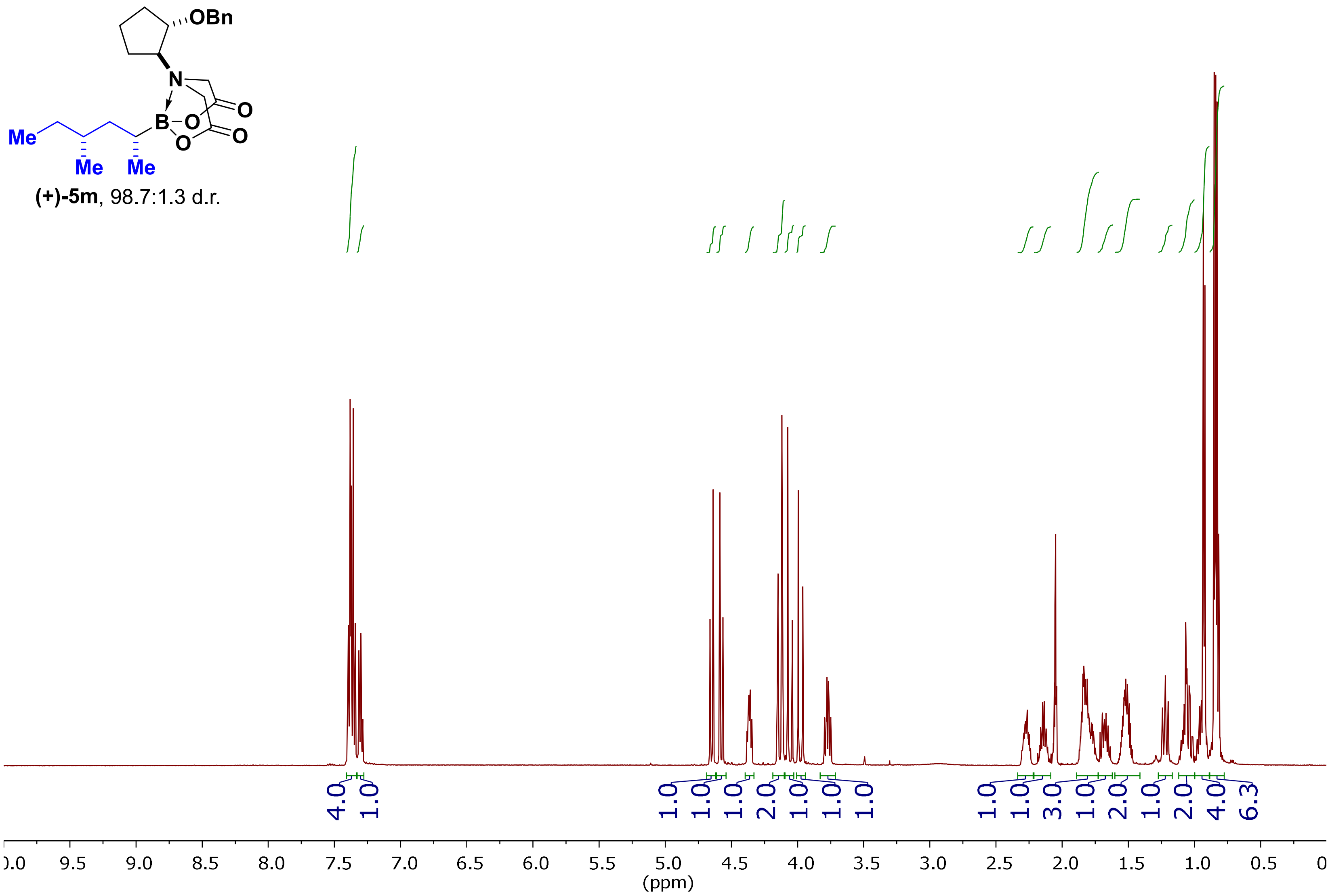

Supplementary Figure 214| ^{1}H-NMR spectrum (500 MHz, acetone-*d6*) for **(+)-5m**.

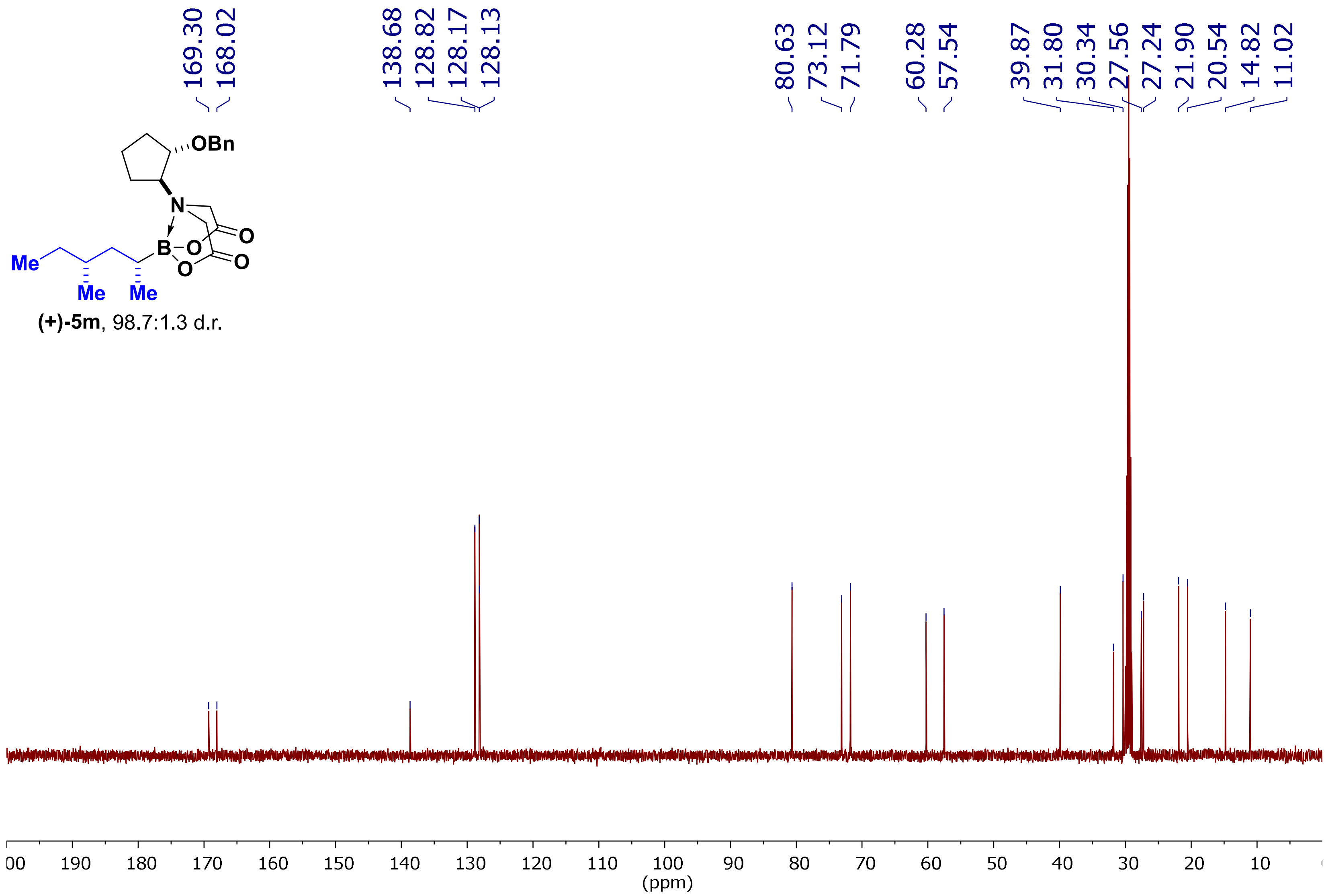

Supplementary Figure 215| ^{13}C-NMR spectrum (126 MHz, acetone-*d6*) for **(+)-5m**.

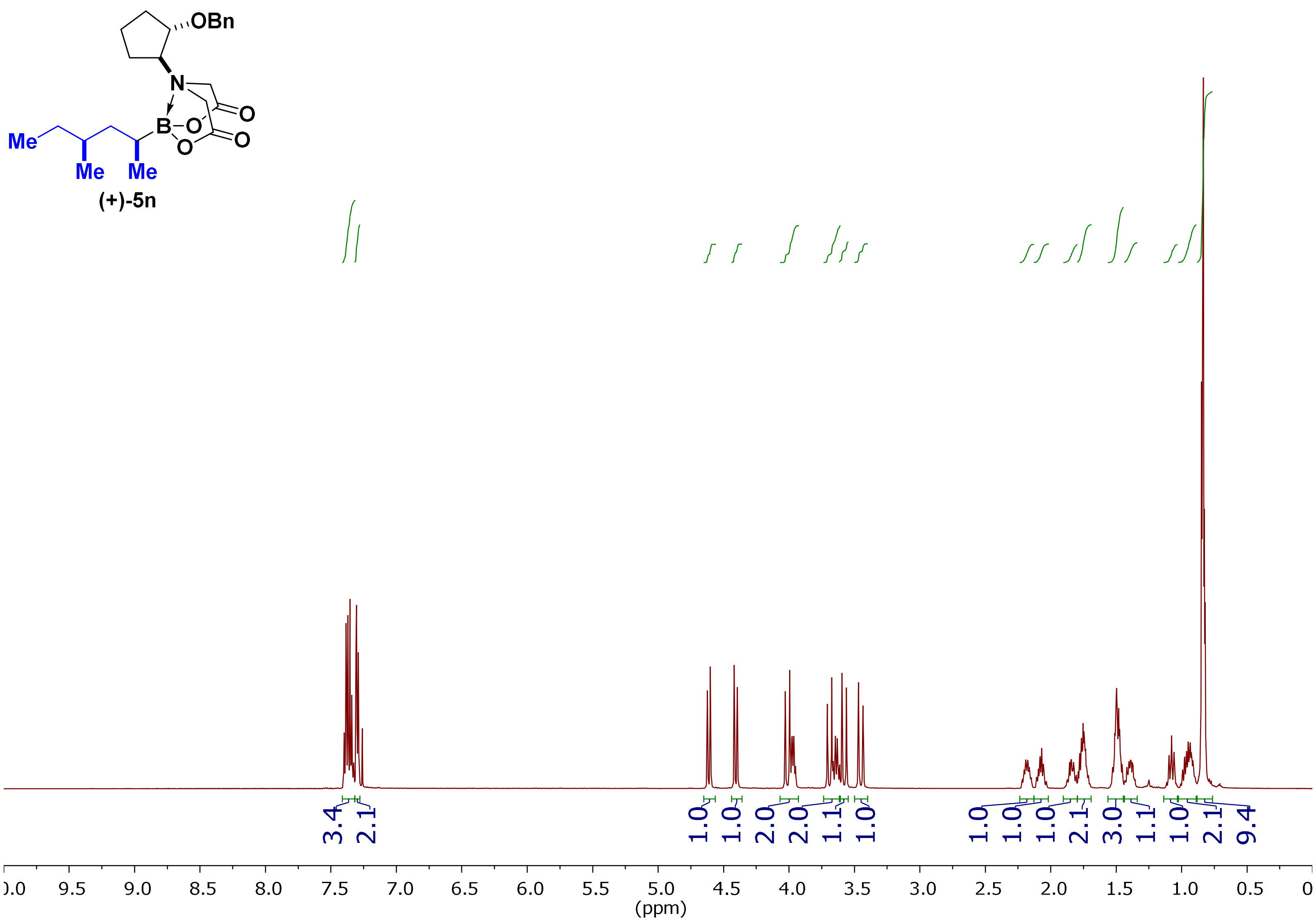

Supplementary Figure 216| ^{1}H-NMR spectrum (500 MHz, $CDCl_3$) for **(+)-5n**.

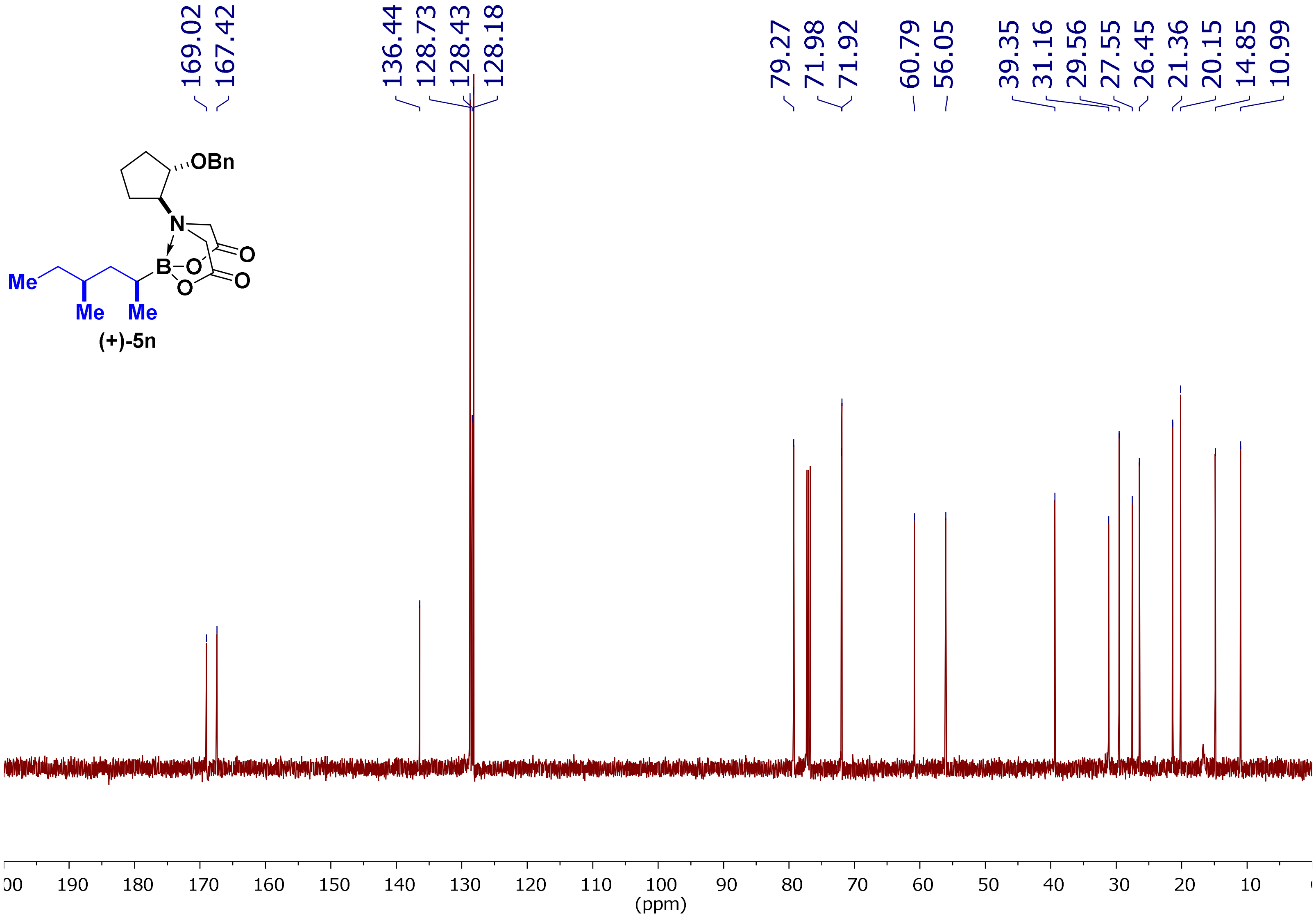

Supplementary Figure 217| ^{13}C-NMR spectrum (126 MHz, $CDCl_3$) for **(+)-5n**.

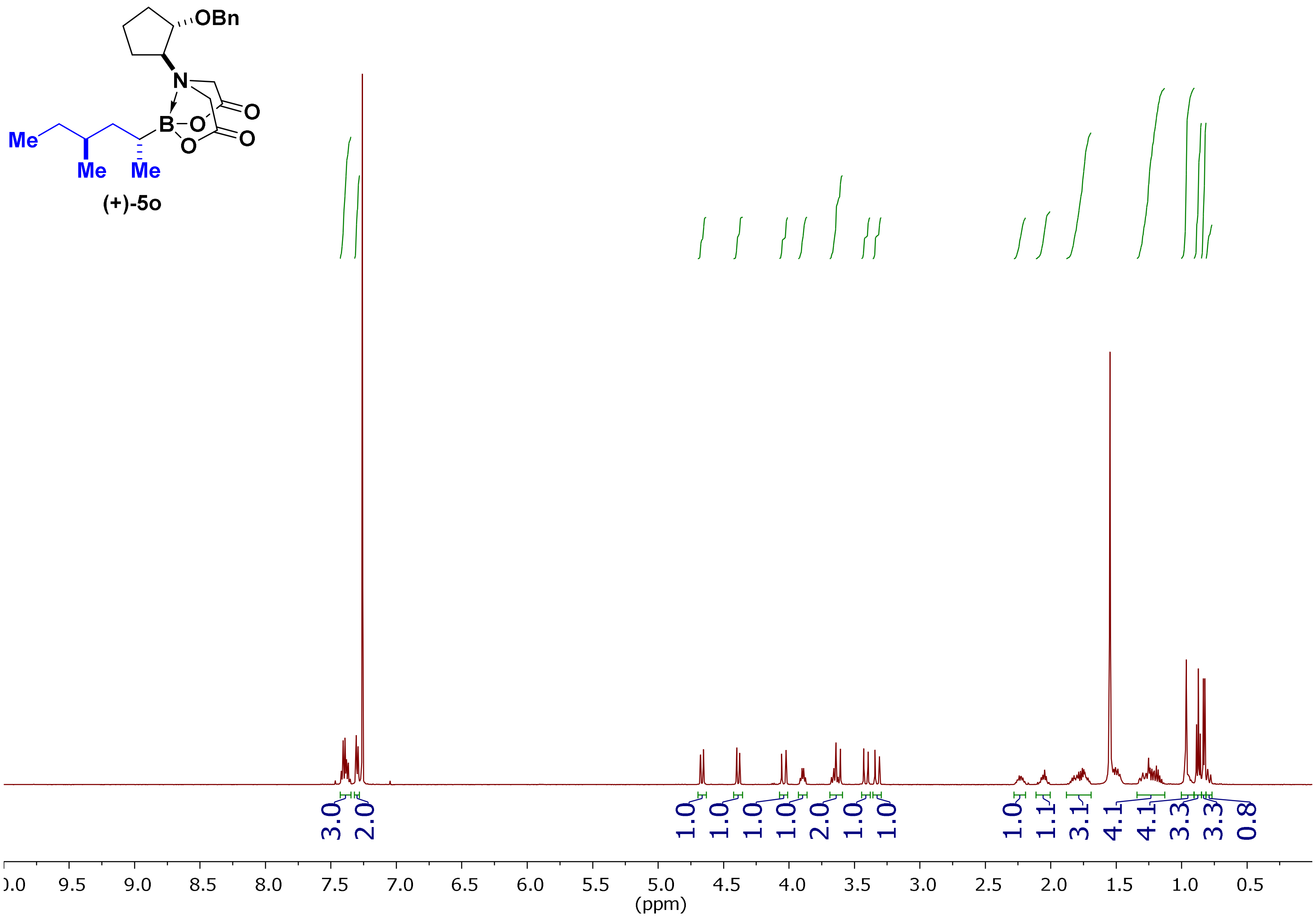

Supplementary Figure 218| ^{1}H-NMR spectrum (500 MHz, $CDCl_3$) for **(+)-5o**.

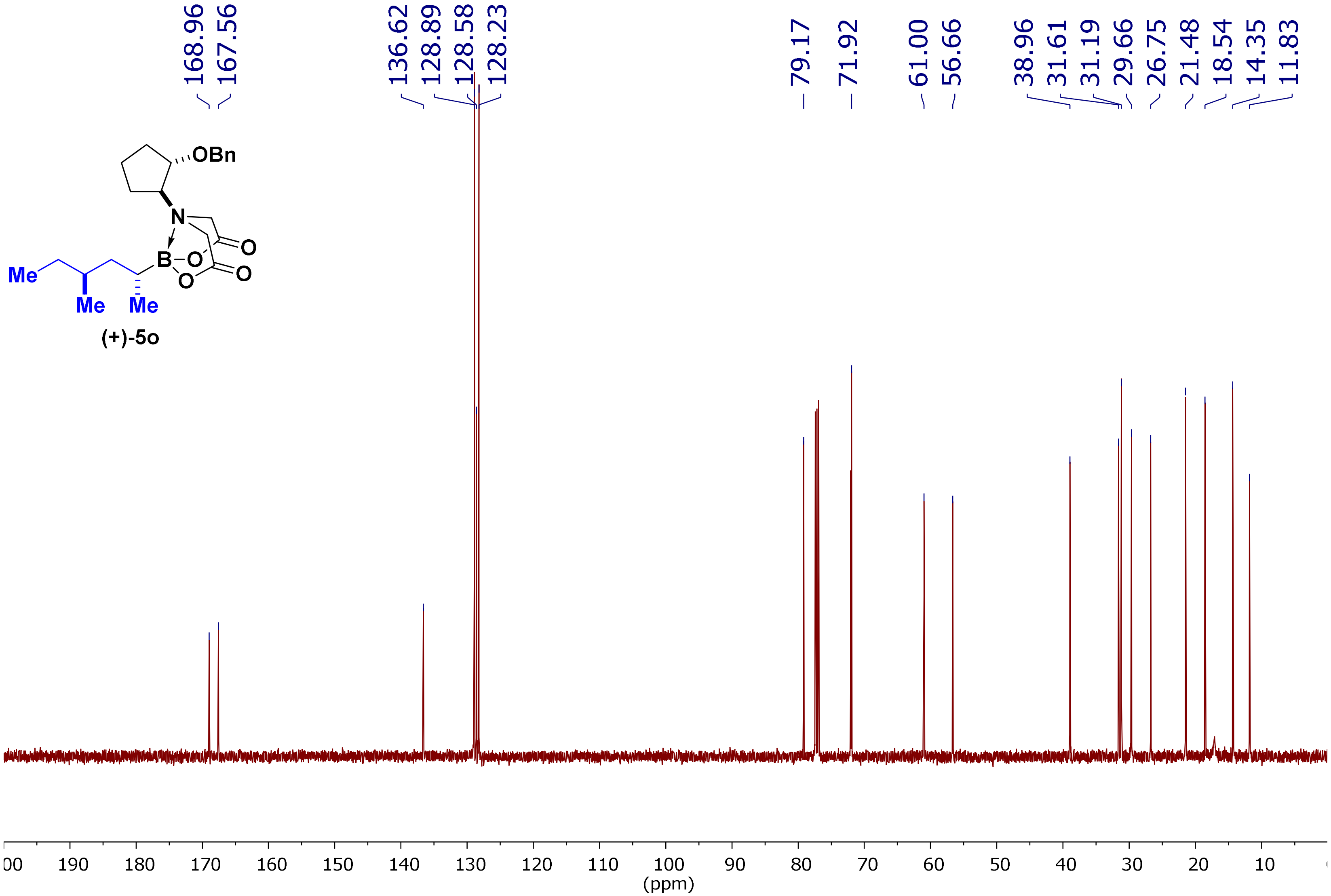

Supplementary Figure 219| ^{13}C-NMR spectrum (126 MHz, $CDCl_3$) for **(+)-5o**.

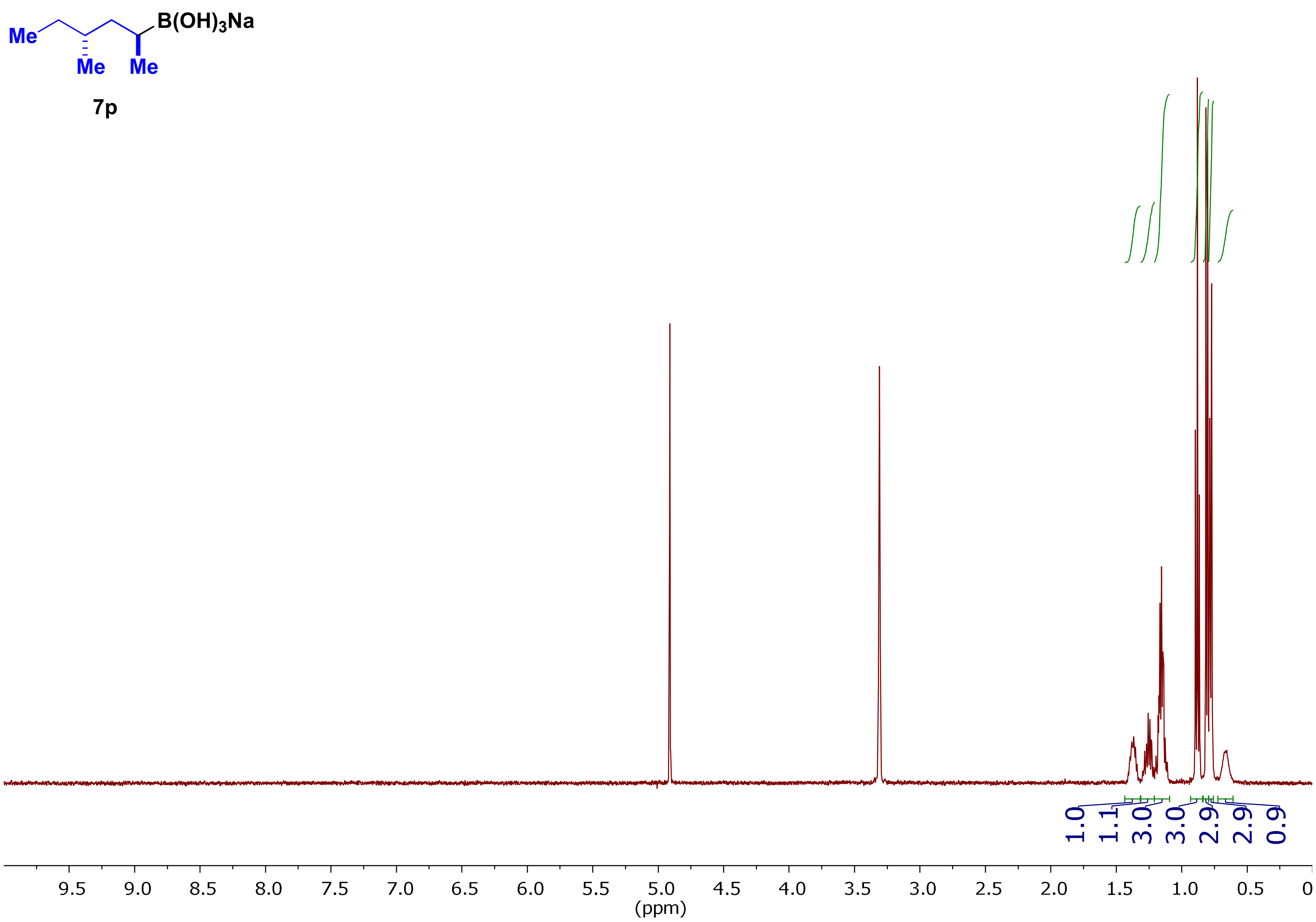

Supplementary Figure 220| ^{1}H-NMR spectrum (500 MHz, CD_3OD) for **7p**.

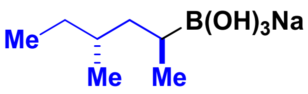

7p

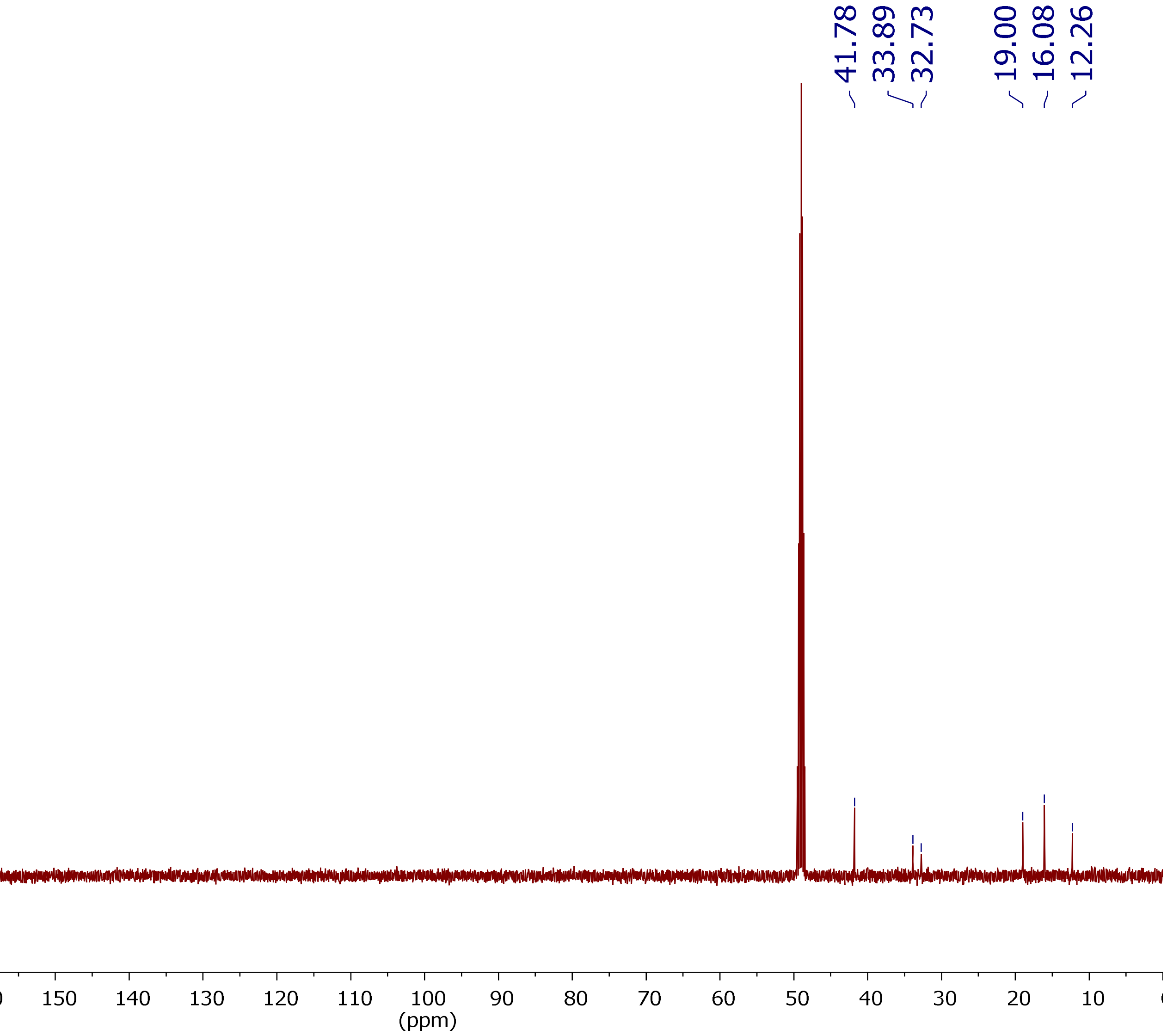

Supplementary Figure 221| ^{13}C-NMR spectrum (126 MHz, CD_3OD) for **7p**.

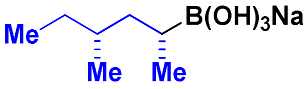

7m

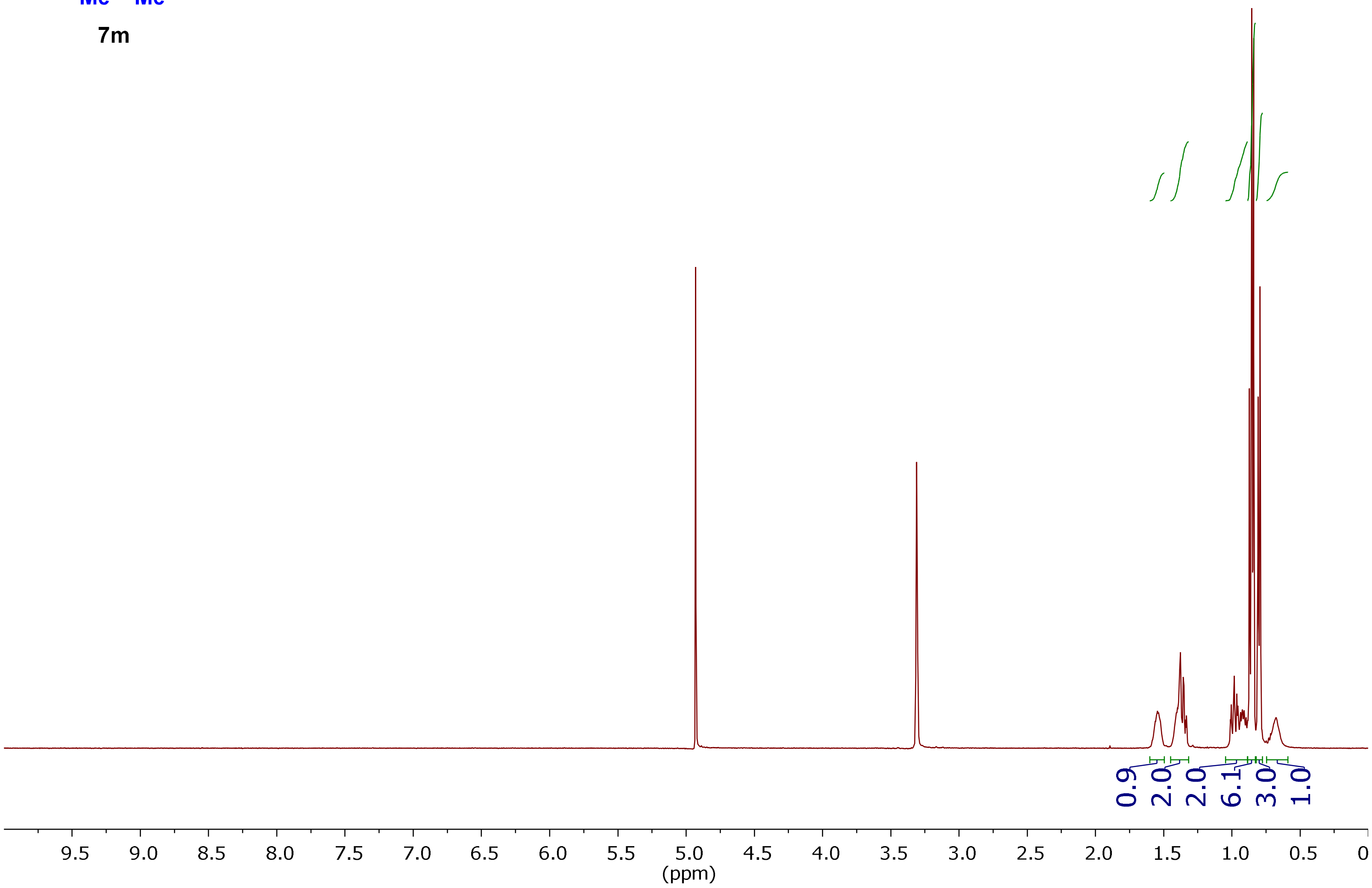

Supplementary Figure 222| ^{1}H-NMR spectrum (500 MHz, CD_3OD) for **7m**.

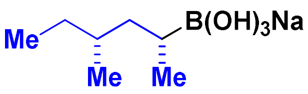

7m

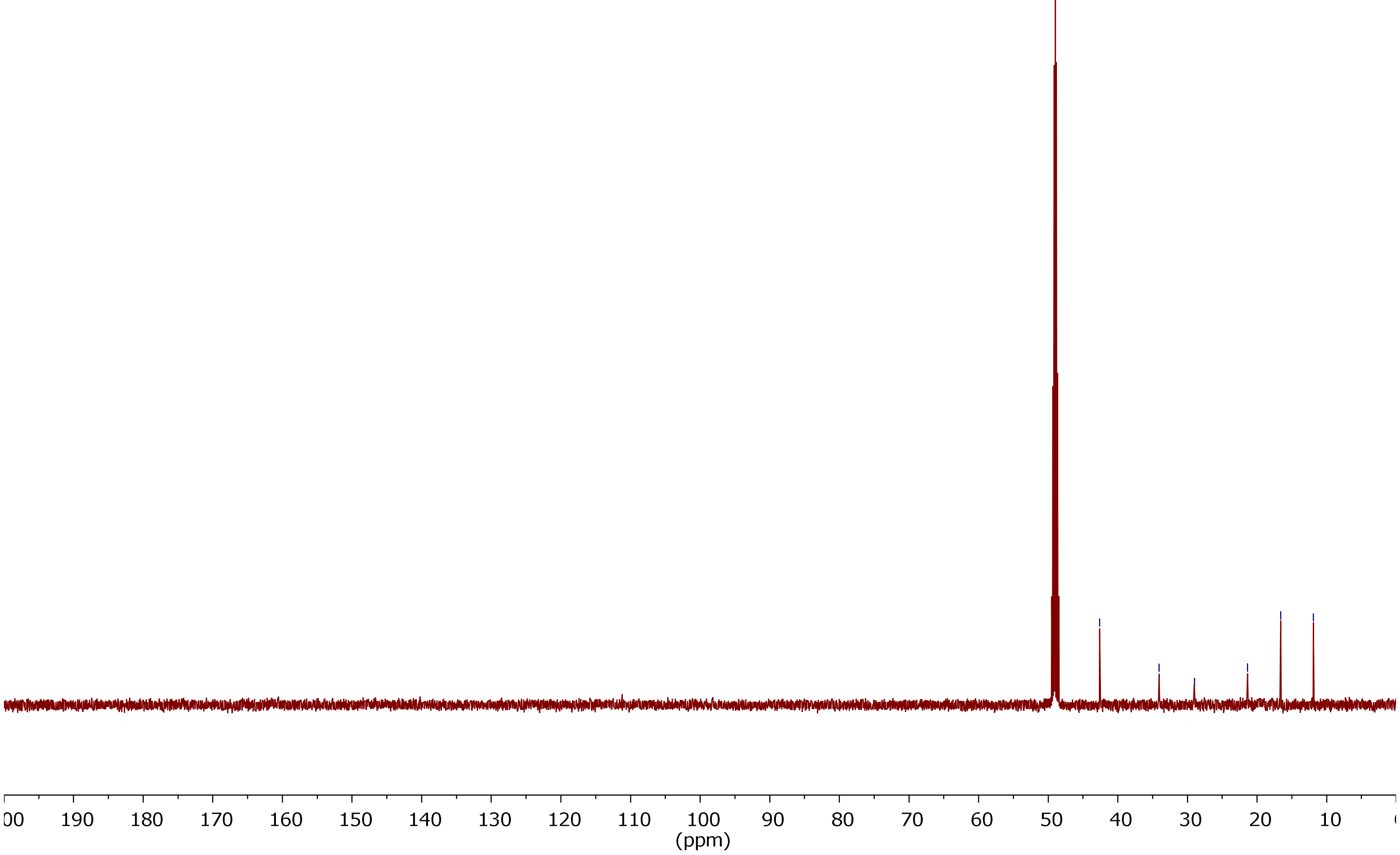

Supplementary Figure 223| ^{13}C-NMR spectrum (126 MHz, CD_3OD) for **7m**.

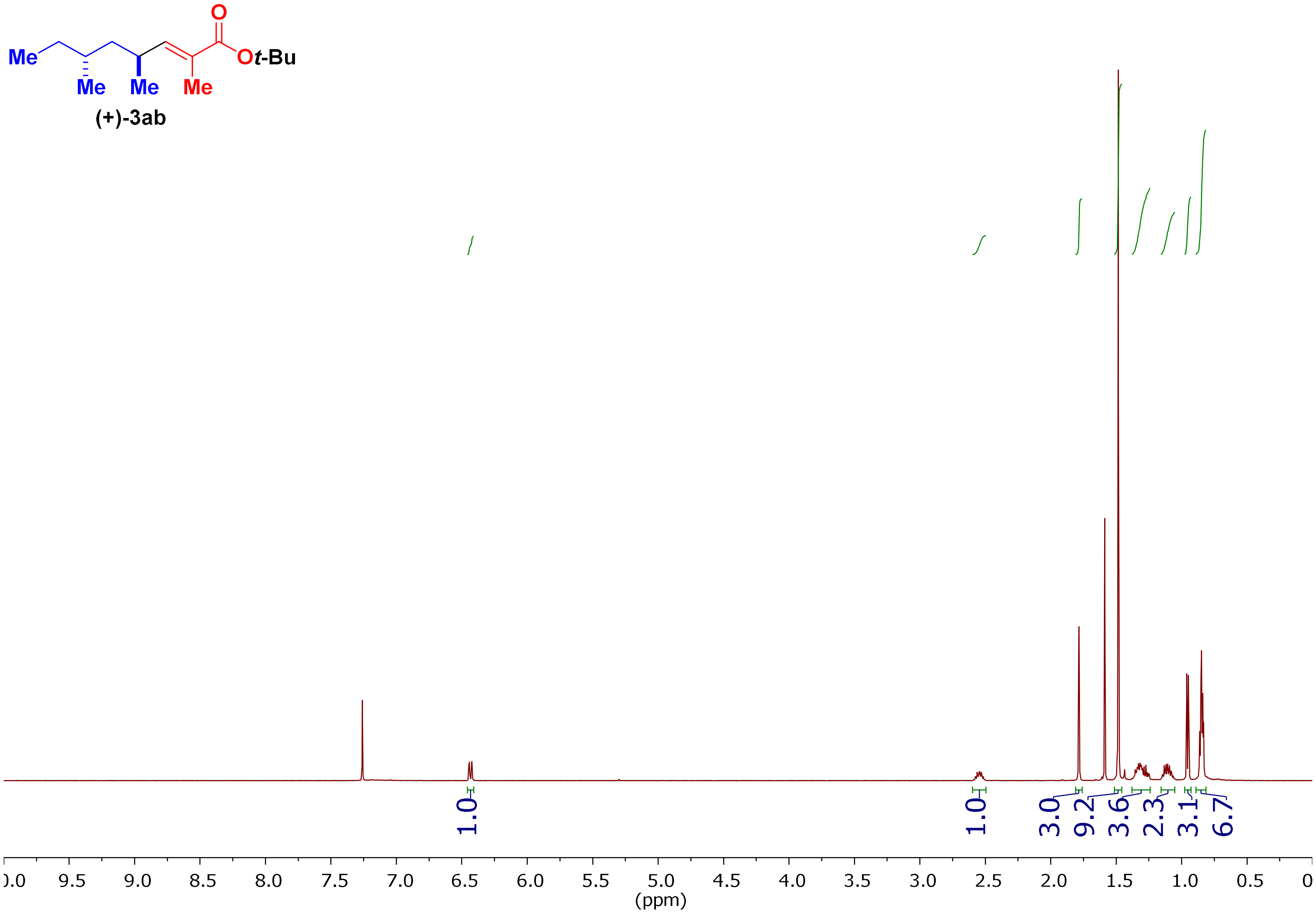

Supplementary Figure 224| ^1H-NMR spectrum (500 MHz, $CDCl_3$) for **(+)-3ab**.

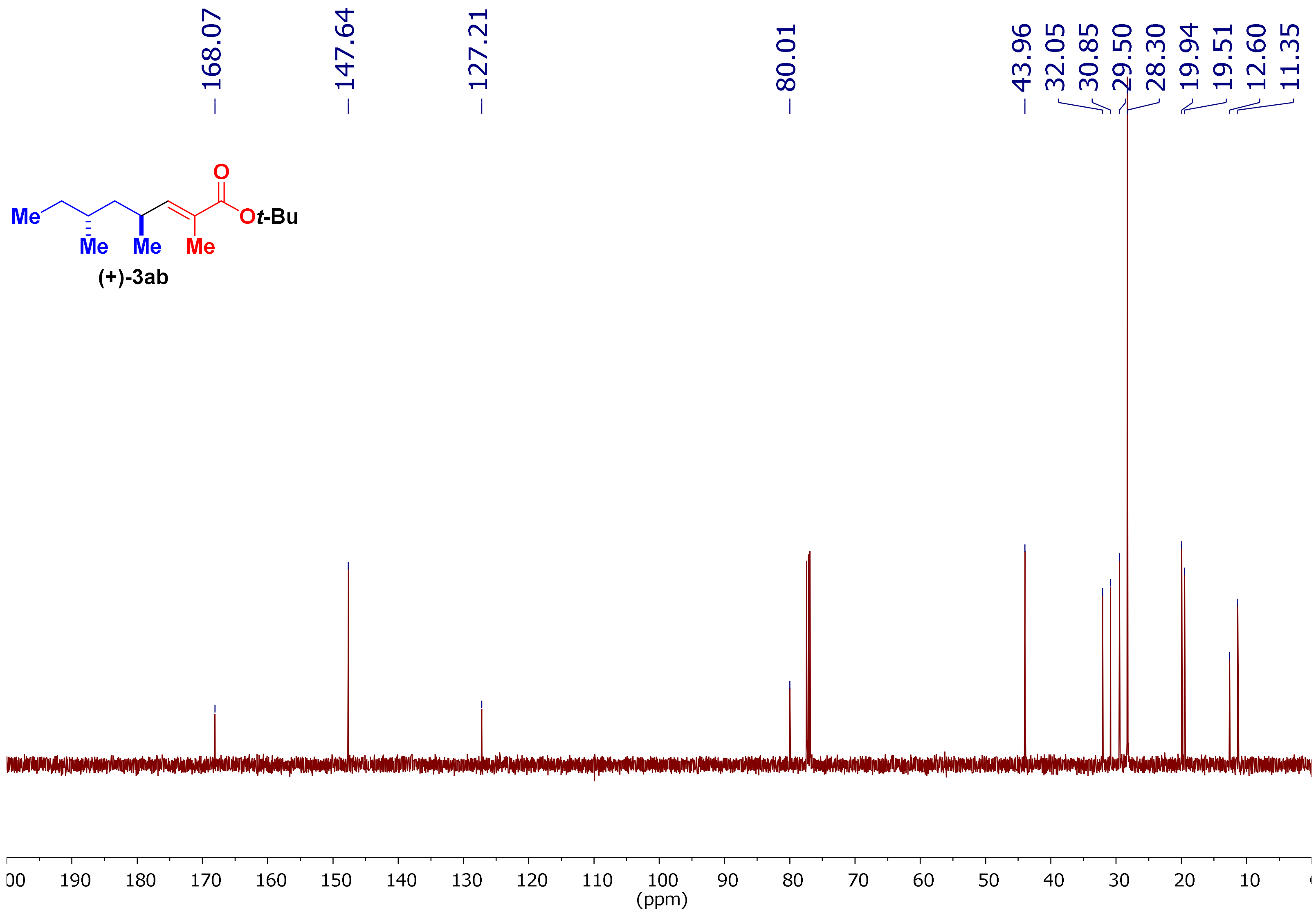

Supplementary Figure 225| ^{13}C-NMR spectrum (126 MHz, $CDCl_3$) for **(+)-3ab**.

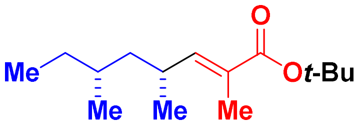

(-)-3ac

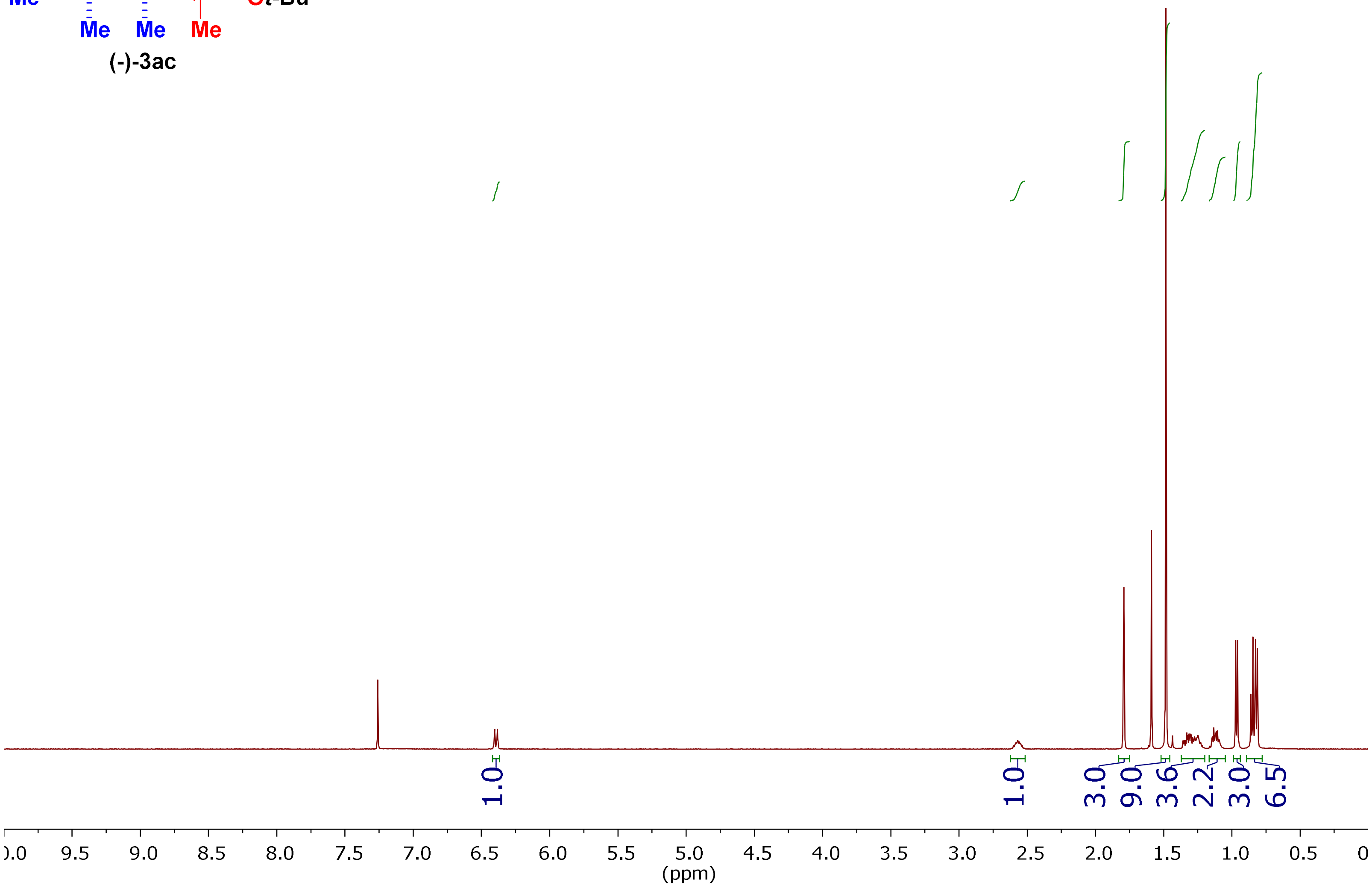

Supplementary Figure 226| ^{1}H-NMR spectrum (500 MHz, $CDCl_3$) for **(-)-3ac**.

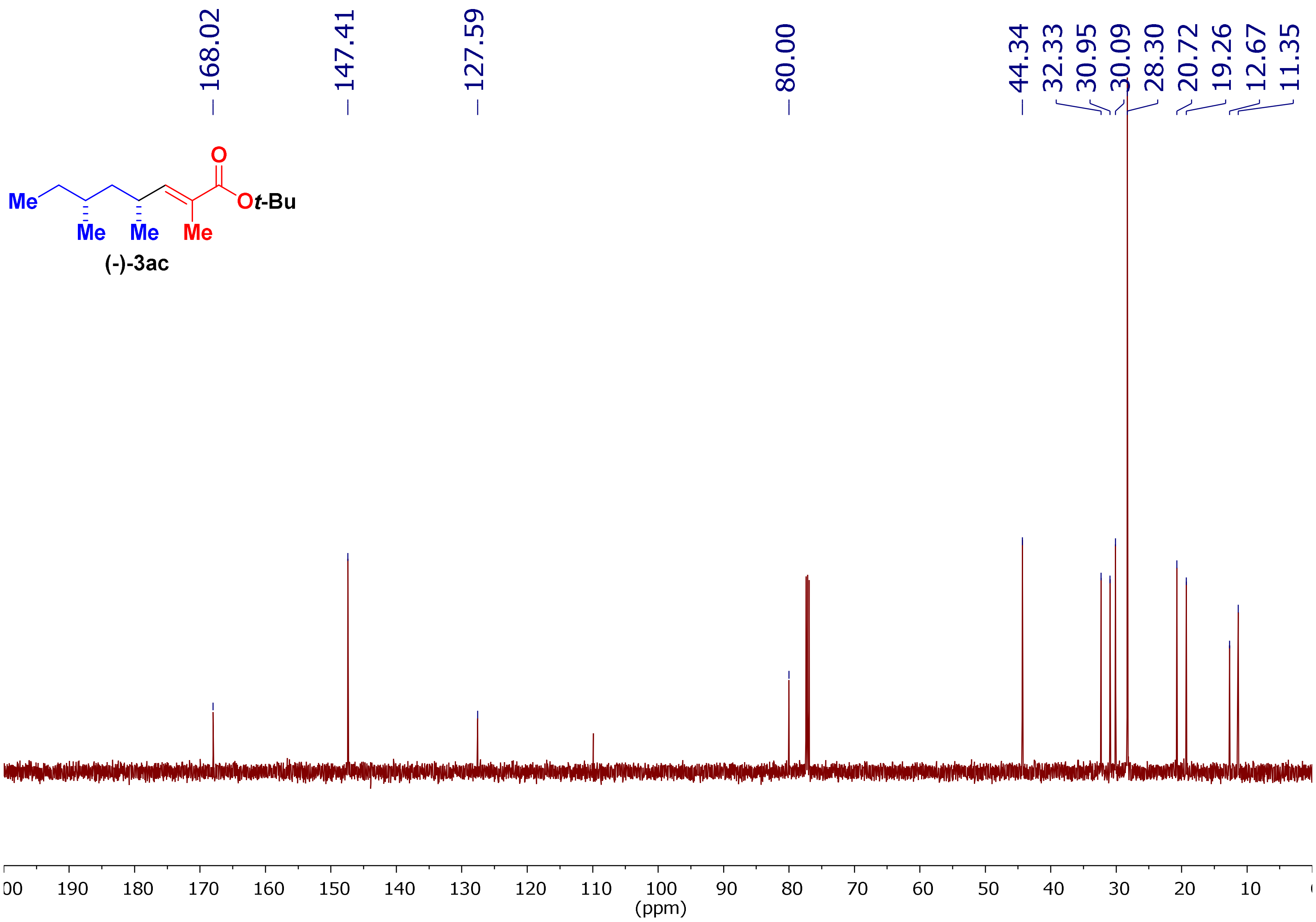

Supplementary Figure 227| ^{13}C-NMR spectrum (126 MHz, $CDCl_3$) for **(-)-3ac**.

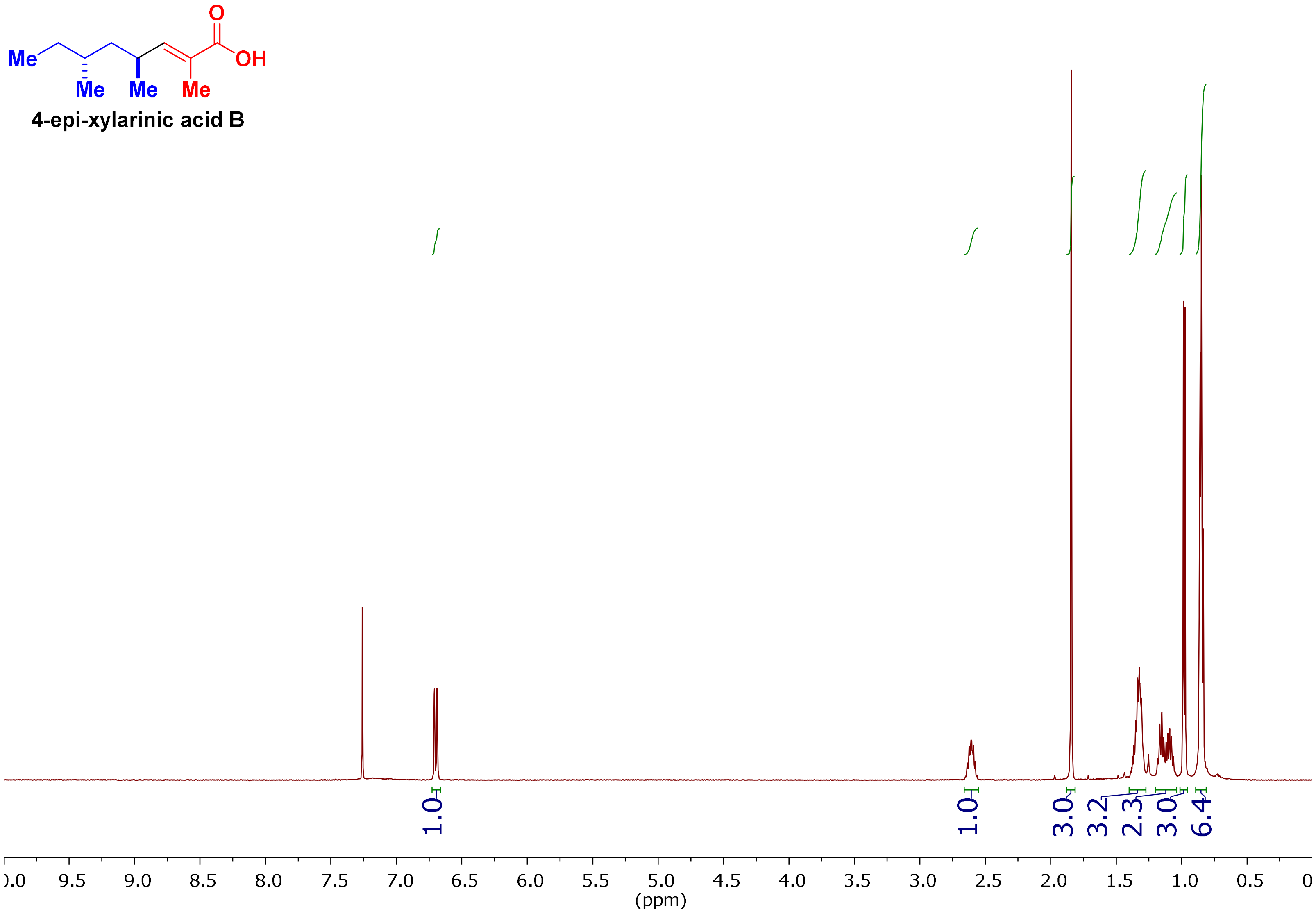

Supplementary Figure 228| ^{1}H-NMR spectrum (500 MHz, $CDCl_3$) for **4-*epi*-xylarinic acid B**.

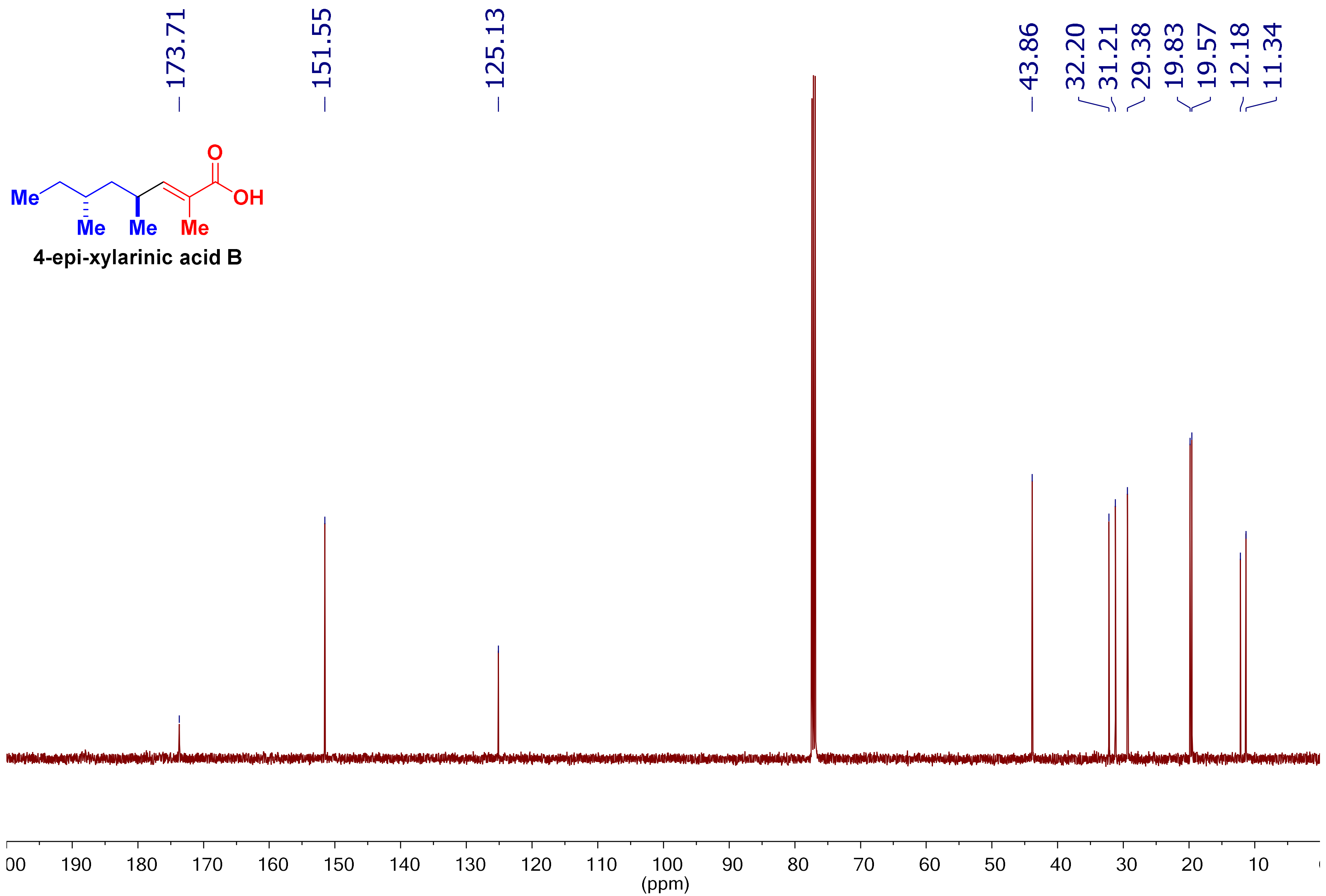

Supplementary Figure 229| ^{13}C-NMR spectrum (126 MHz, $CDCl_3$) for **4-*epi*-xylarinic acid B**.

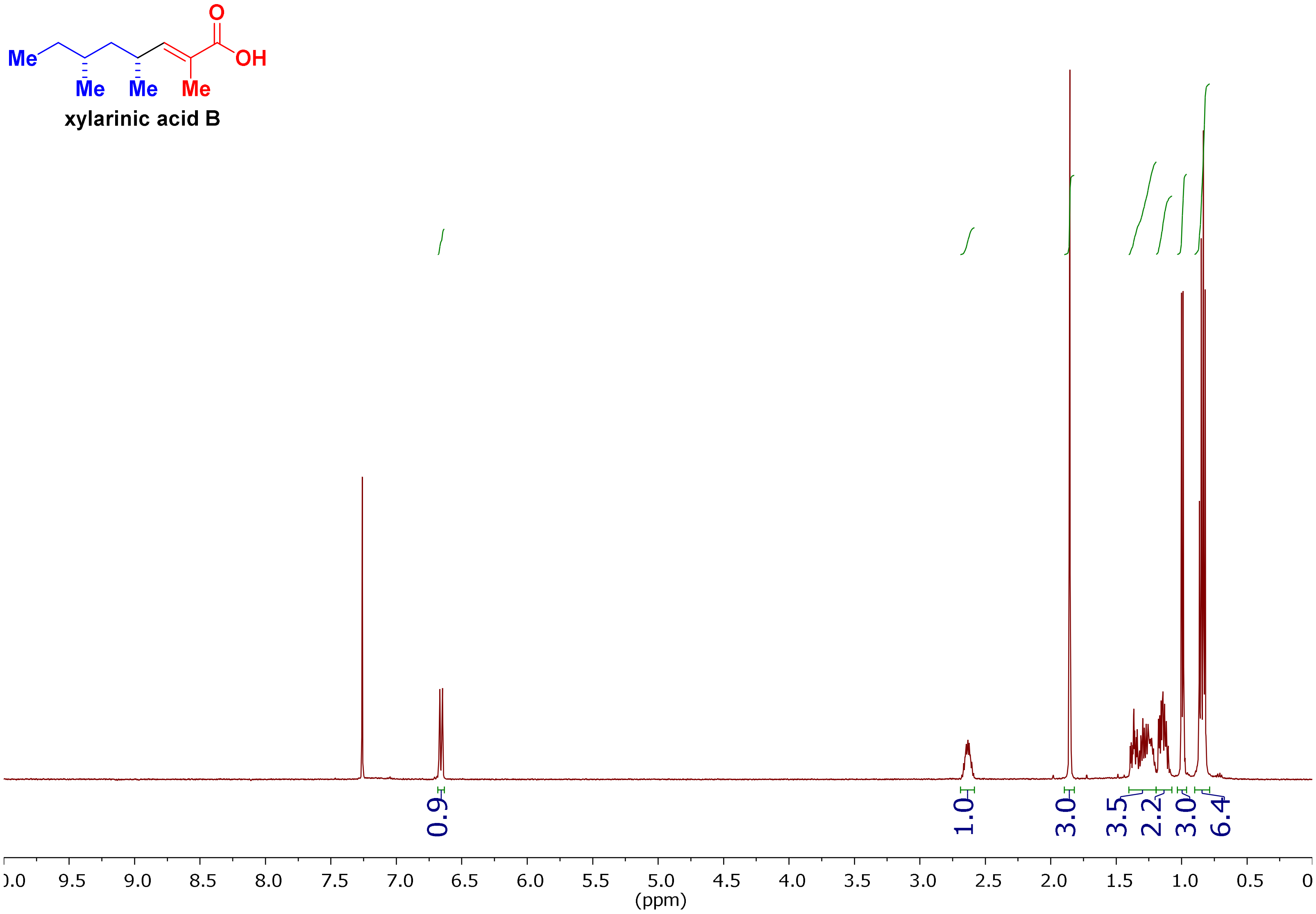

Supplementary Figure 230| ^{1}H-NMR spectrum (500 MHz, $CDCl_3$) for **xylarinic acid B**.

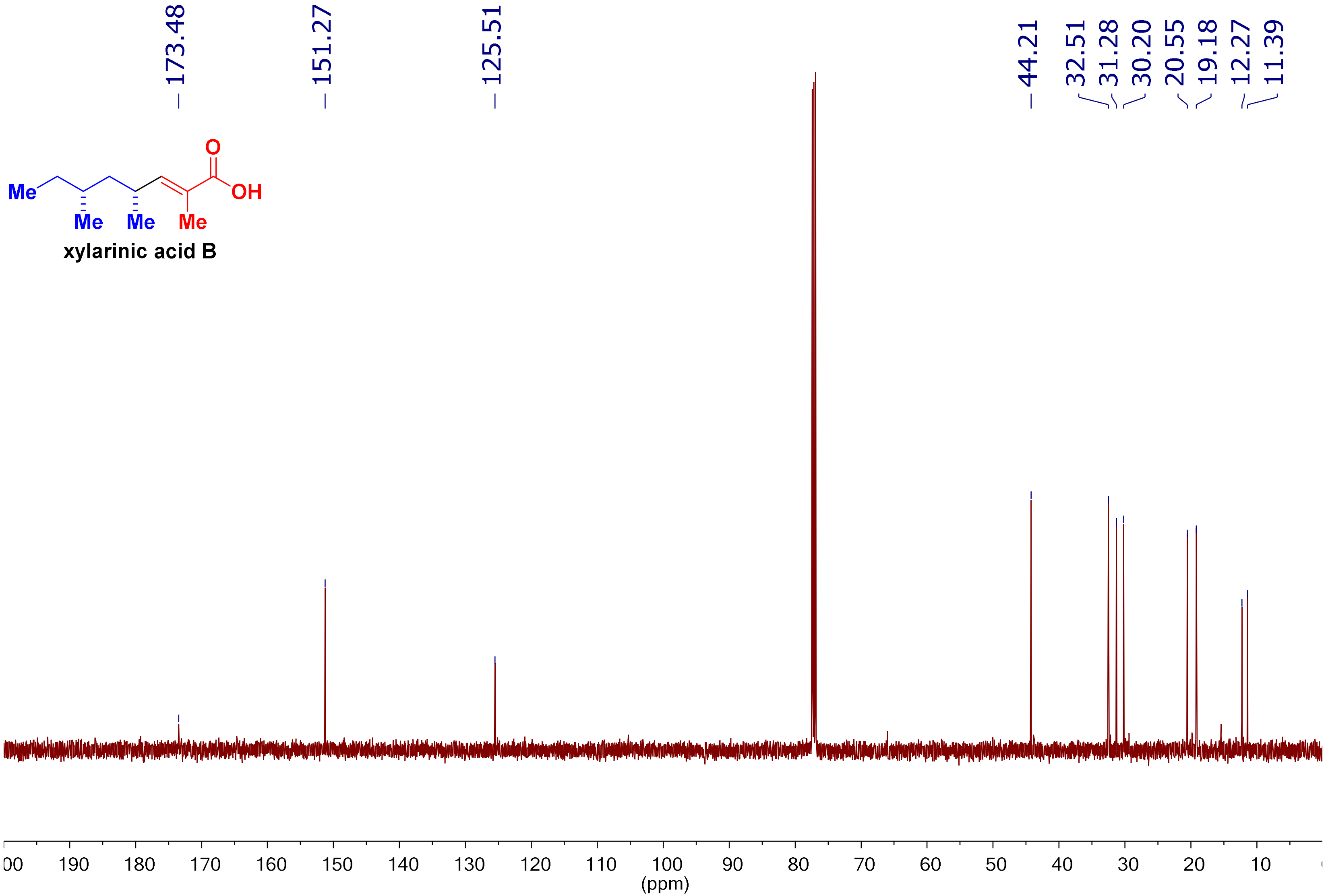

Supplementary Figure 231| ^{13}C-NMR spectrum (126 MHz, $CDCl_3$) for **xylarinic acid B**.

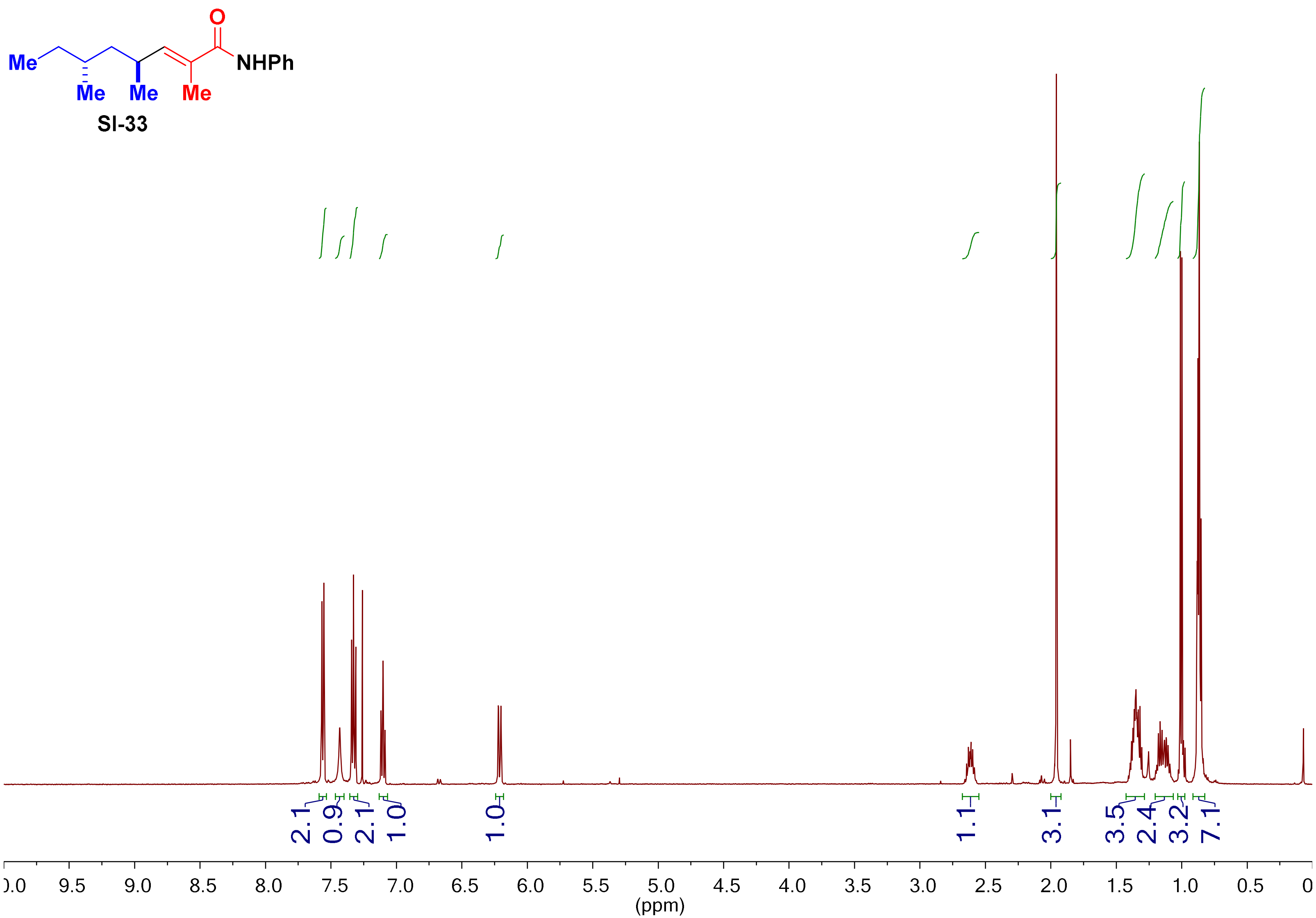

Supplementary Figure 232| ^{1}H-NMR spectrum (500 MHz, $CDCl_3$) for **SI-33**.

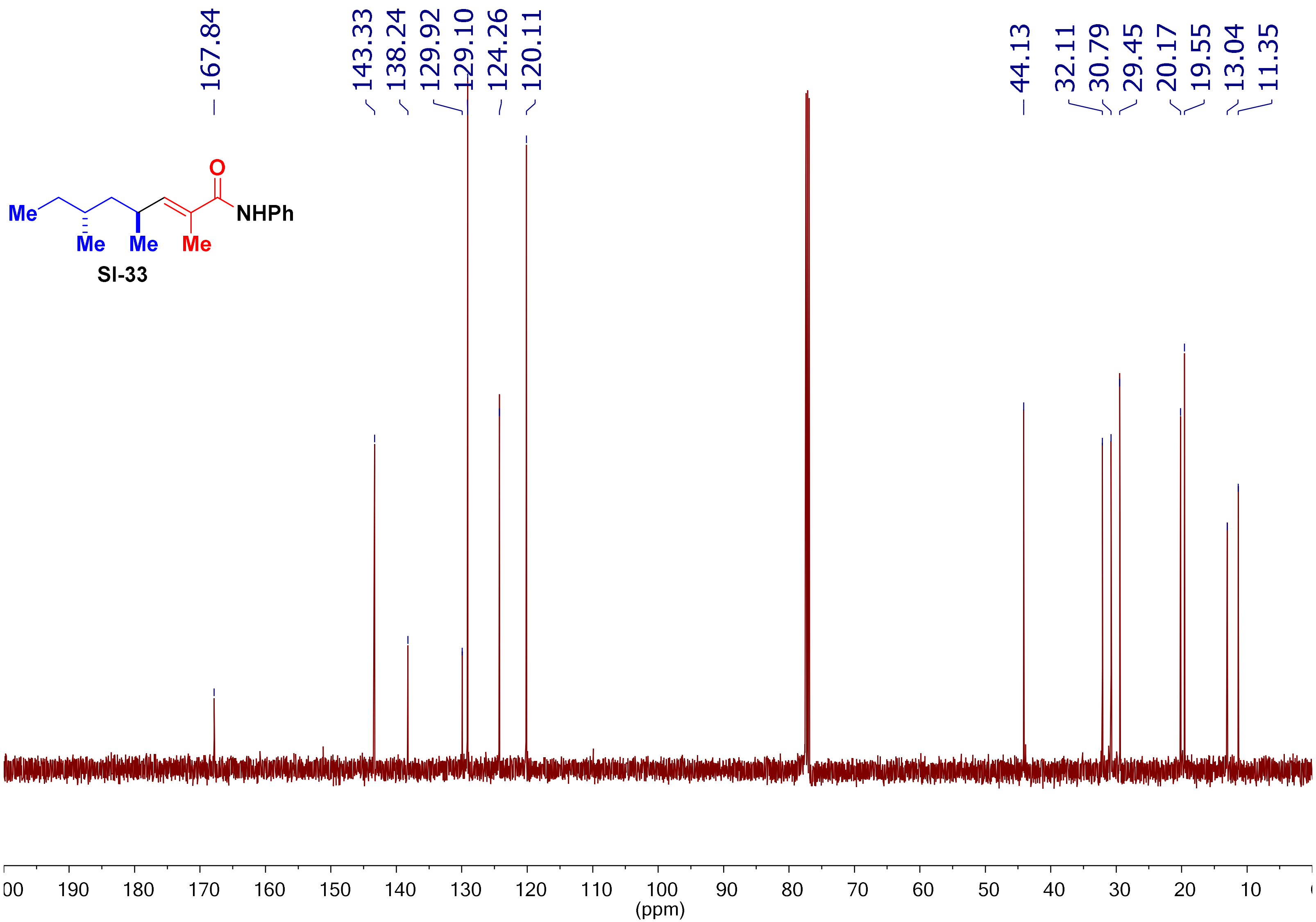

Supplementary Figure 233| ^{13}C-NMR spectrum (126 MHz, $CDCl_3$) for **SI-33**.

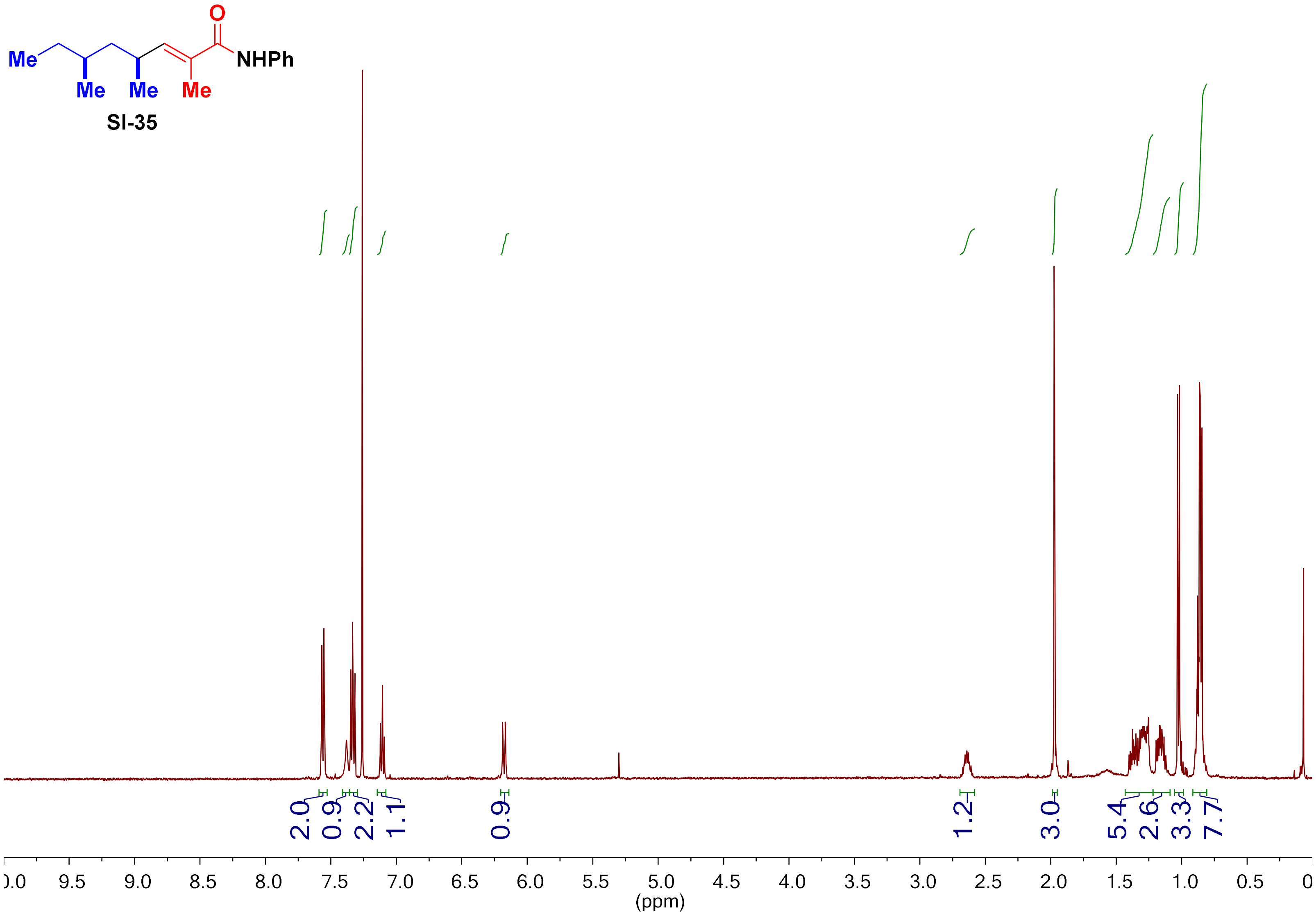

Supplementary Figure 234| ^{1}H-NMR spectrum (500 MHz, $CDCl_3$) for **SI-35**.

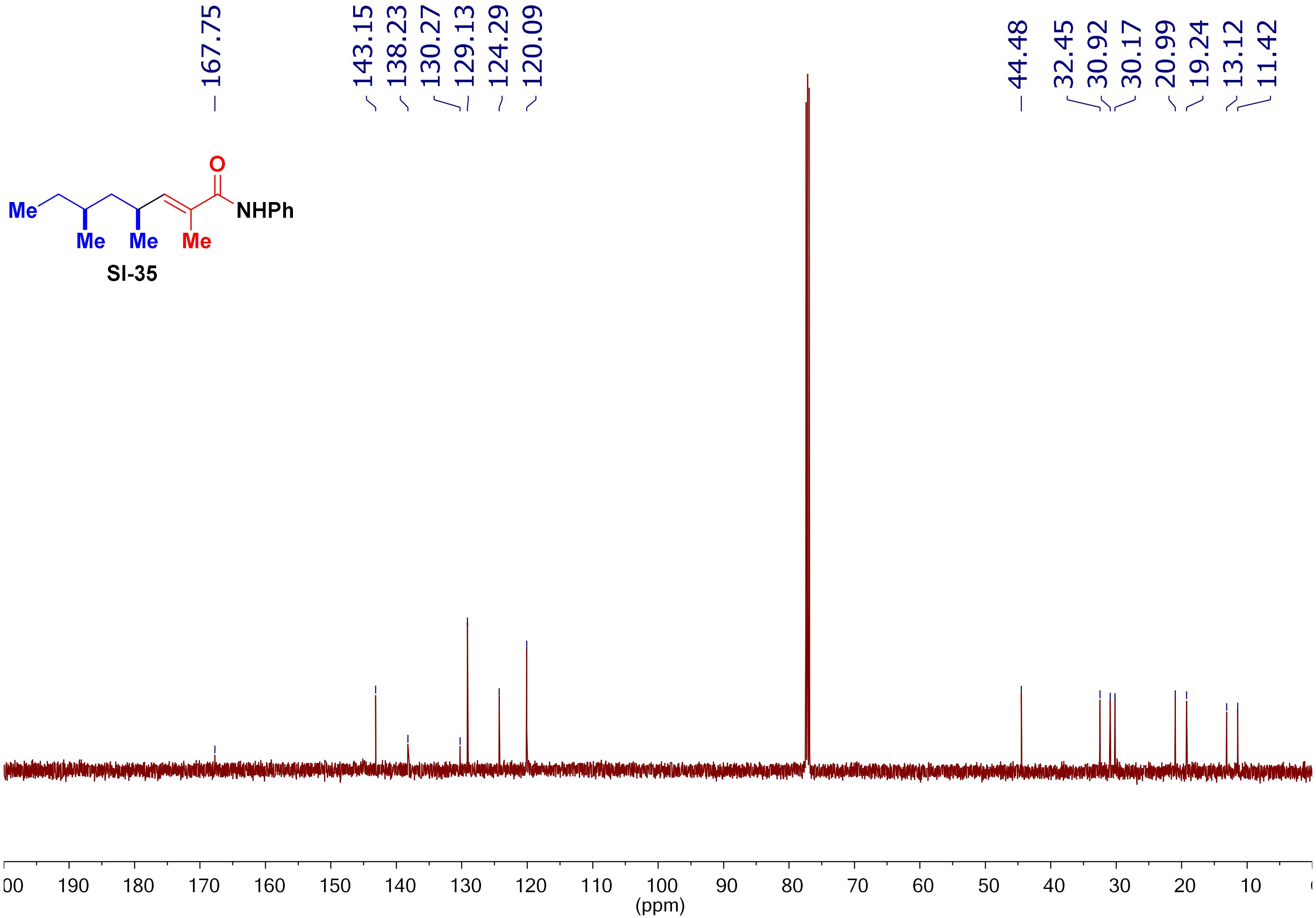

Supplementary Figure 235| ^{13}C-NMR spectrum (126 MHz, $CDCl_3$) for **SI-35**.

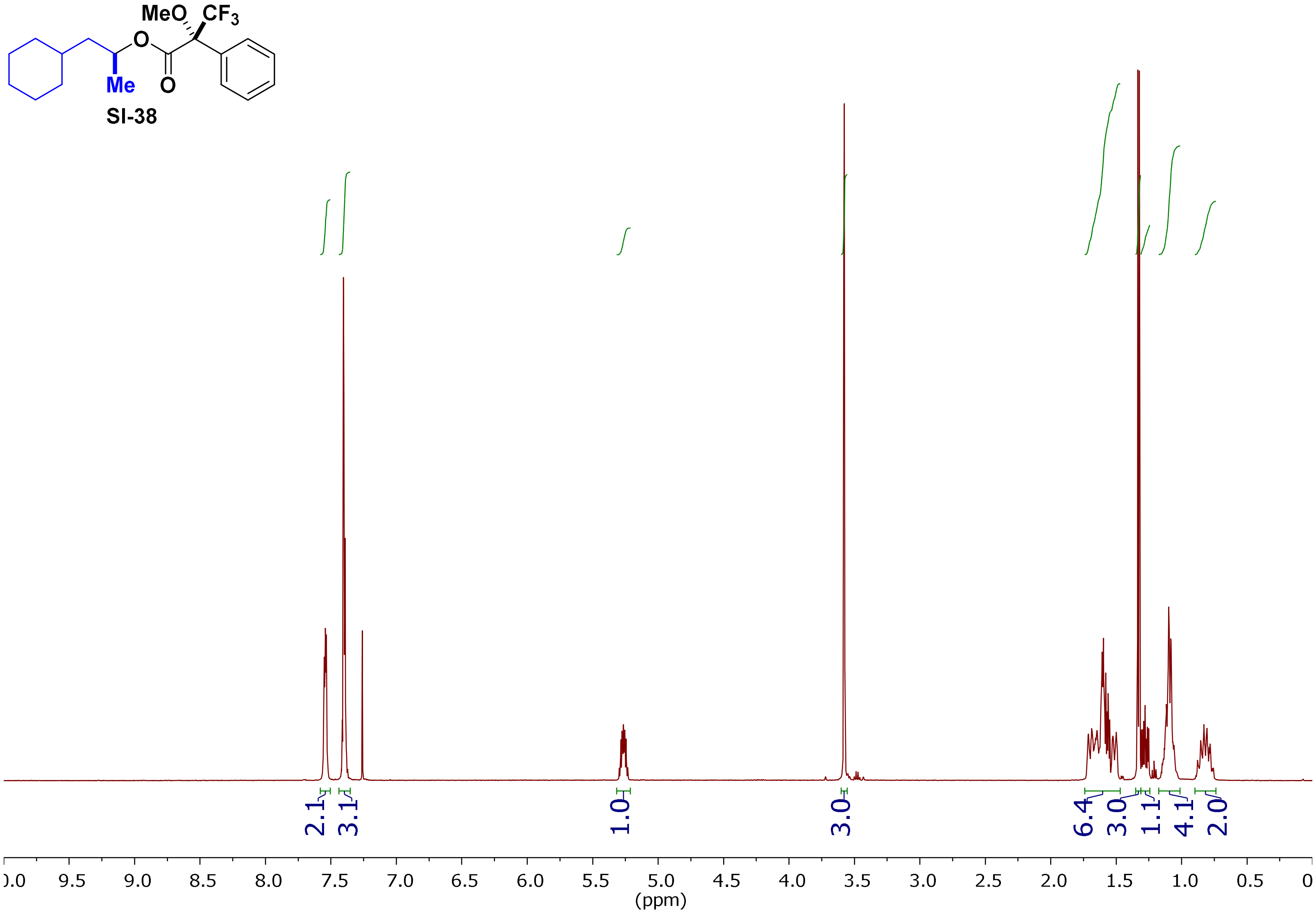

Supplementary Figure 236| ^{1}H-NMR spectrum (500 MHz, $CDCl_3$) for **SI-38**.

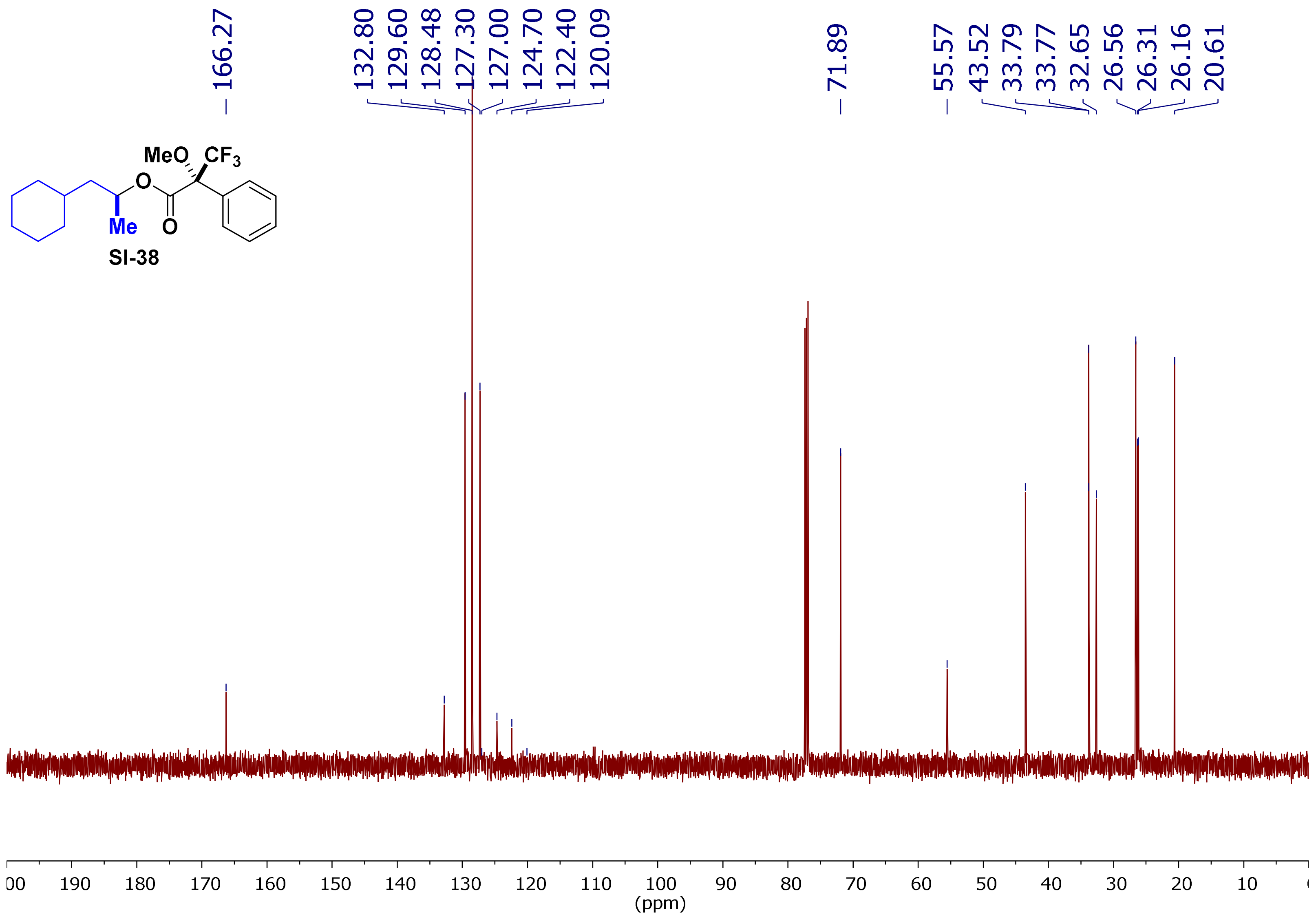

Supplementary Figure 237| ^{13}C-NMR spectrum (126 MHz, $CDCl_3$) for **SI-38**.

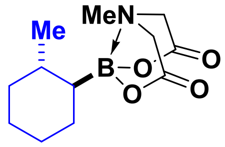

(1*S*, 2*S*)-6l

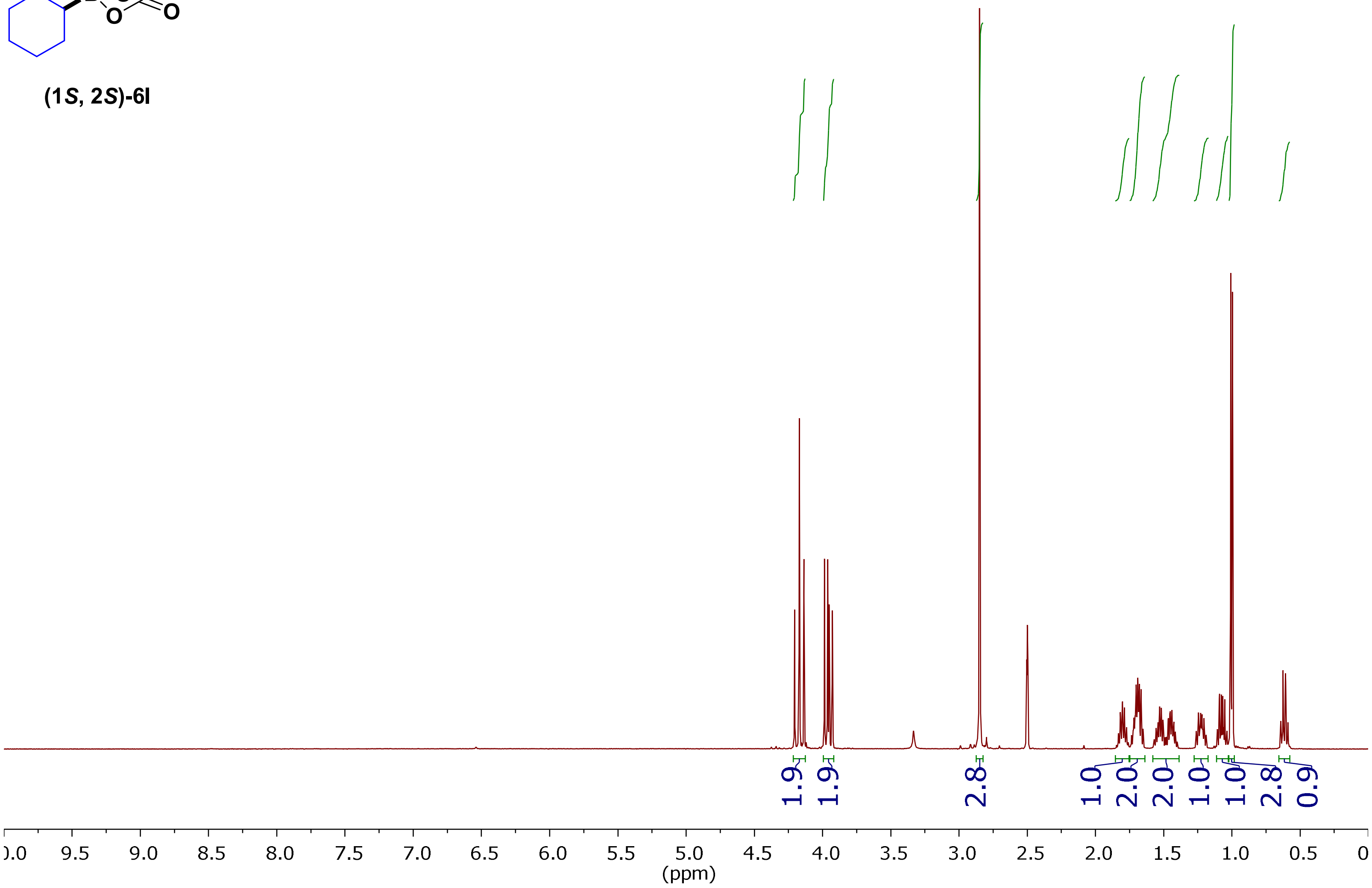

Supplementary Figure 238| ^{1}H-NMR spectrum (500 MHz, DMSO-*d6*) for **(1*S*, 2*S*)-6l**.

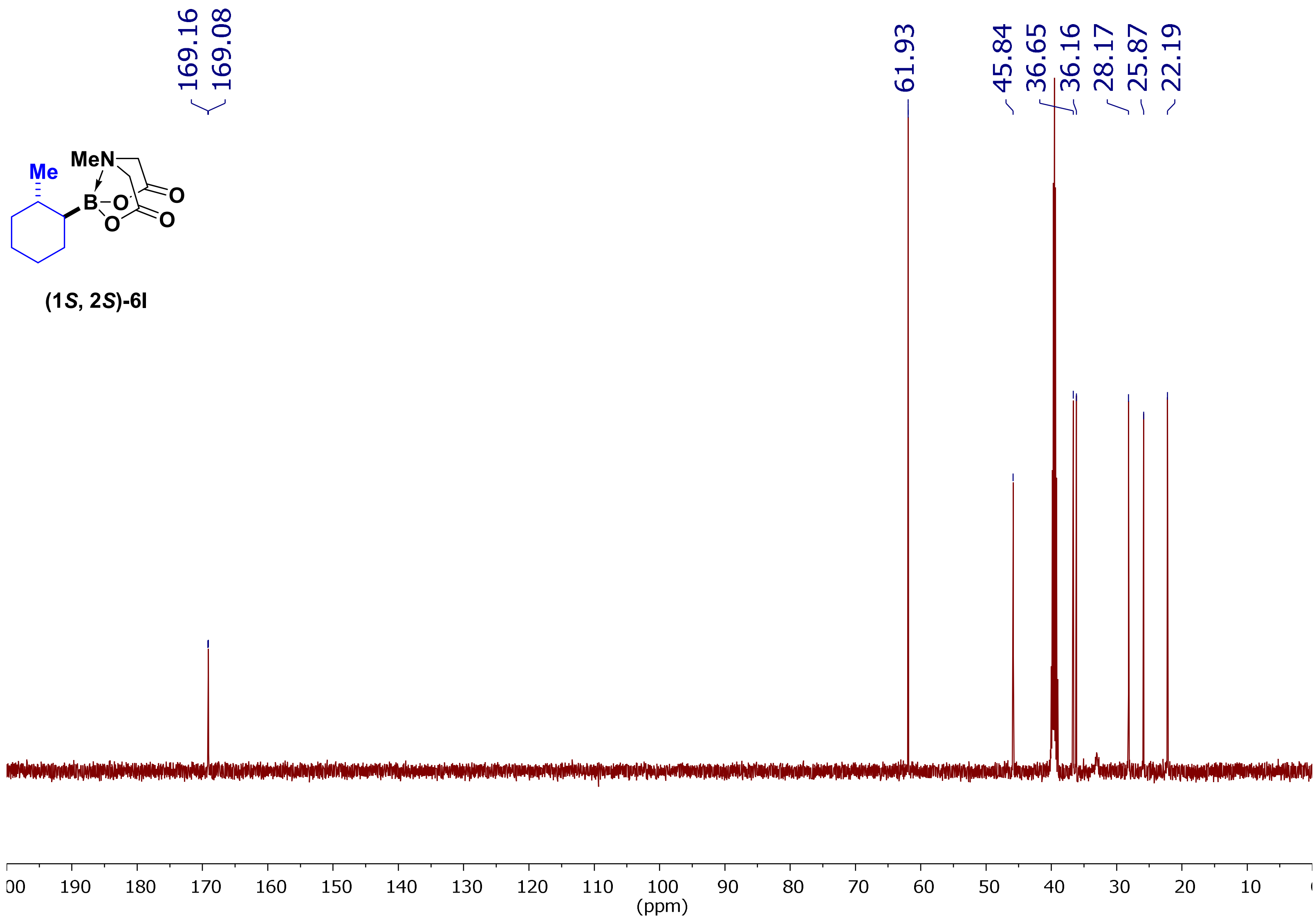

Supplementary Figure 239| ^{13}C-NMR spectrum (126 MHz, DMSO-*d6*) for **(1*S*, 2*S*)-6l**.

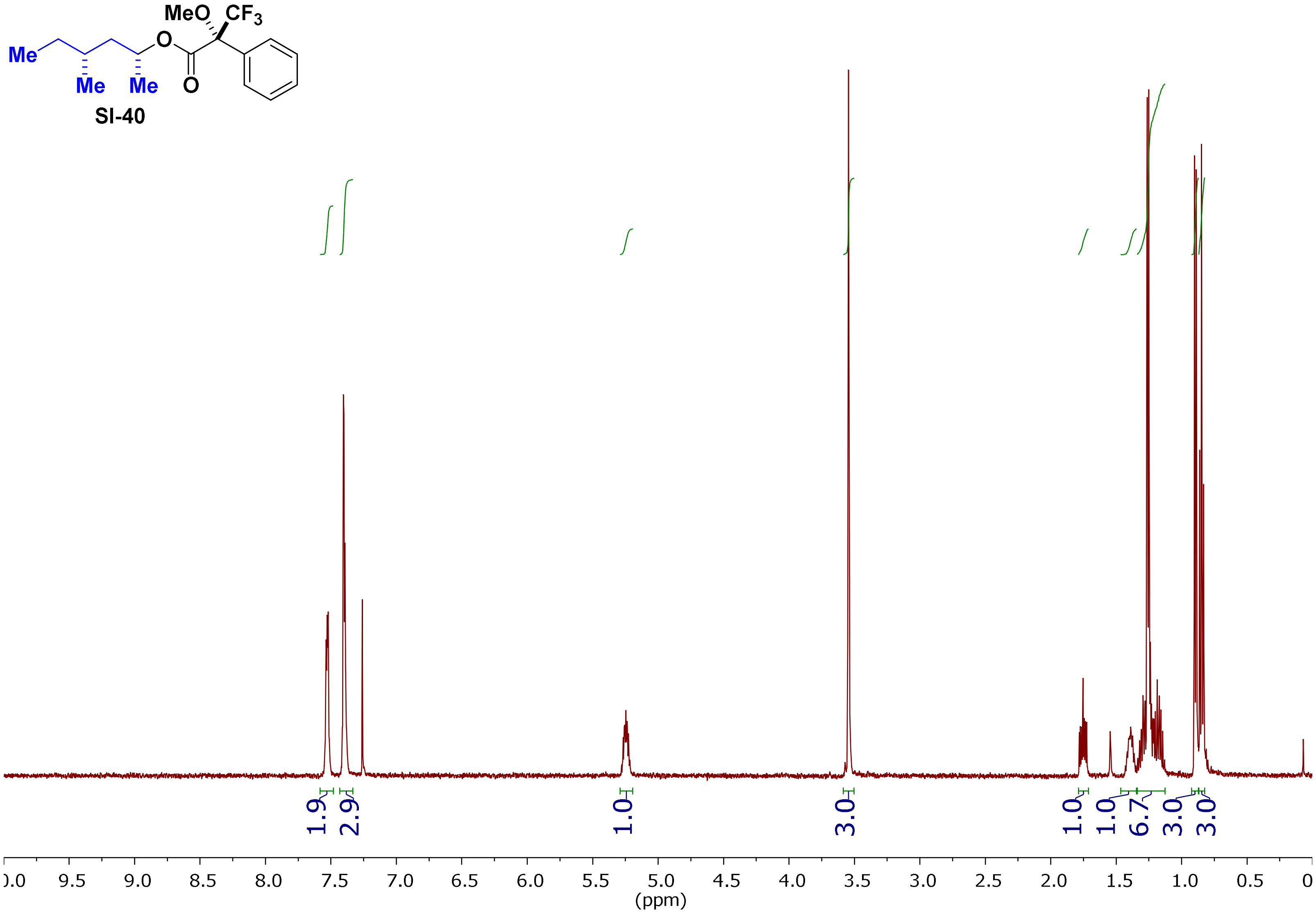

SI-40

Supplementary Figure 240| ^{1}H-NMR spectrum (500 MHz, $CDCl_3$) for **SI-40**.

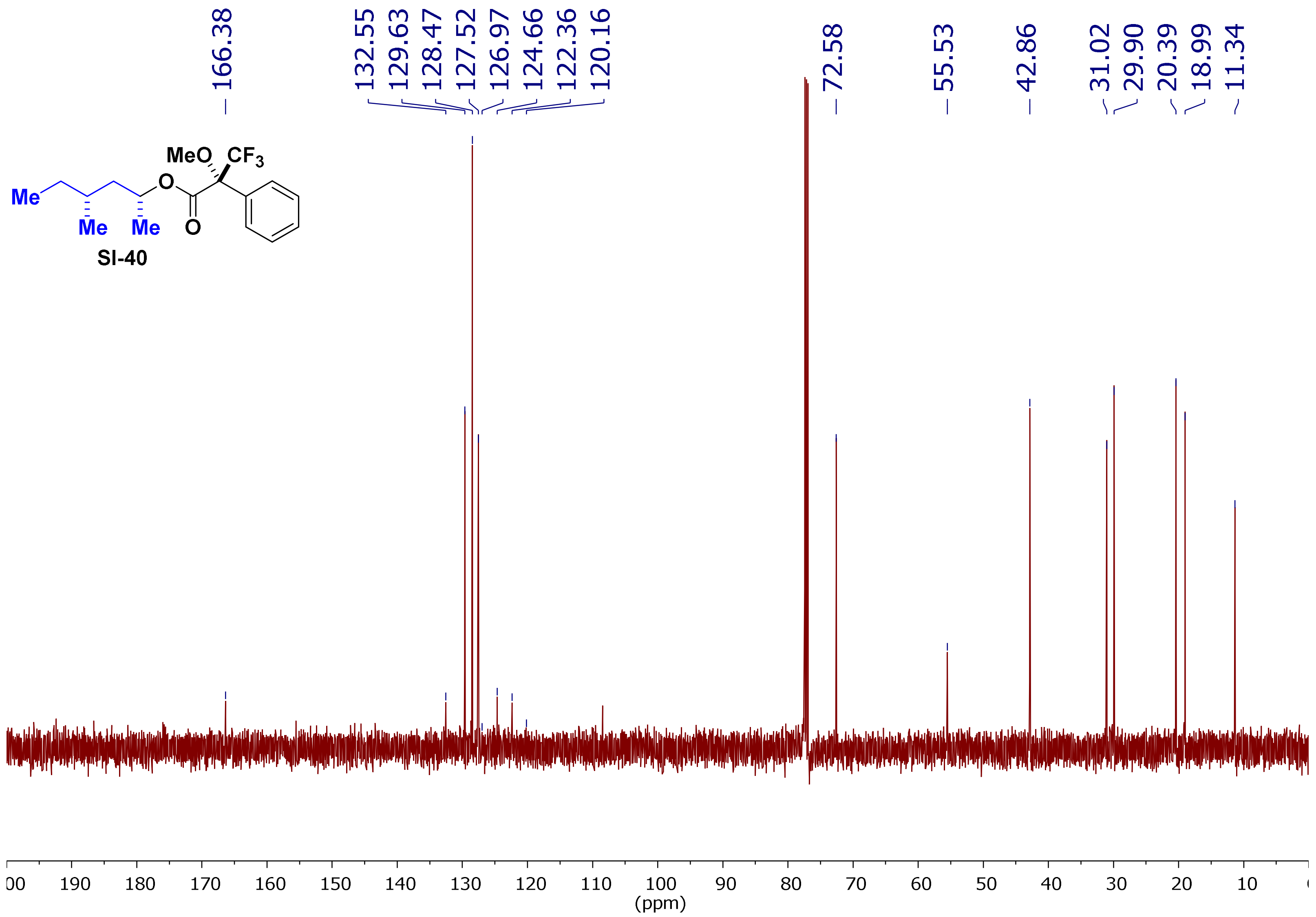

Supplementary Figure 241| ^{13}C-NMR spectrum (126 MHz, $CDCl_3$) for **SI-40**.

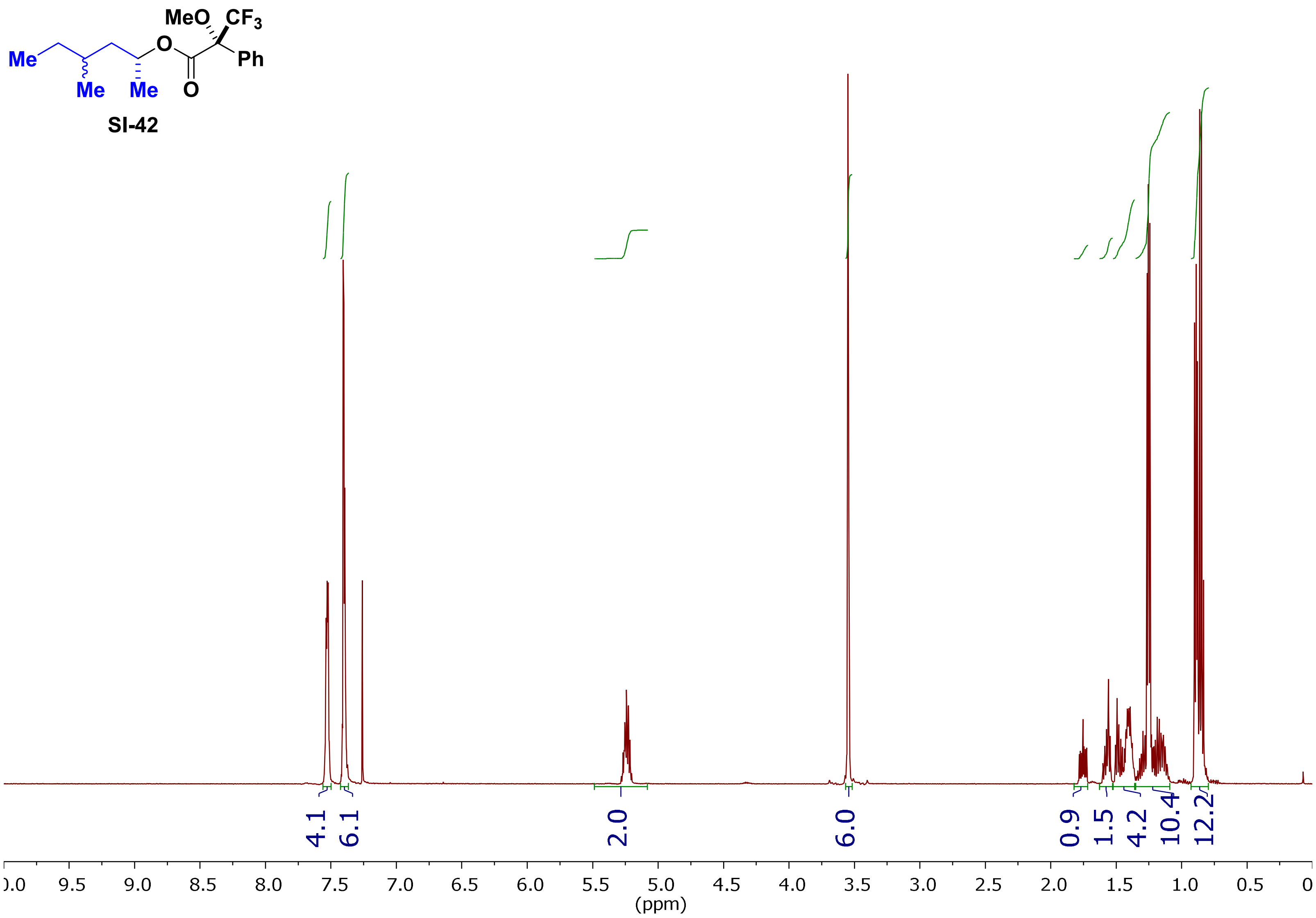

Supplementary Figure 242| ^{1}H-NMR spectrum (500 MHz, $CDCl_3$) for **SI-42**.

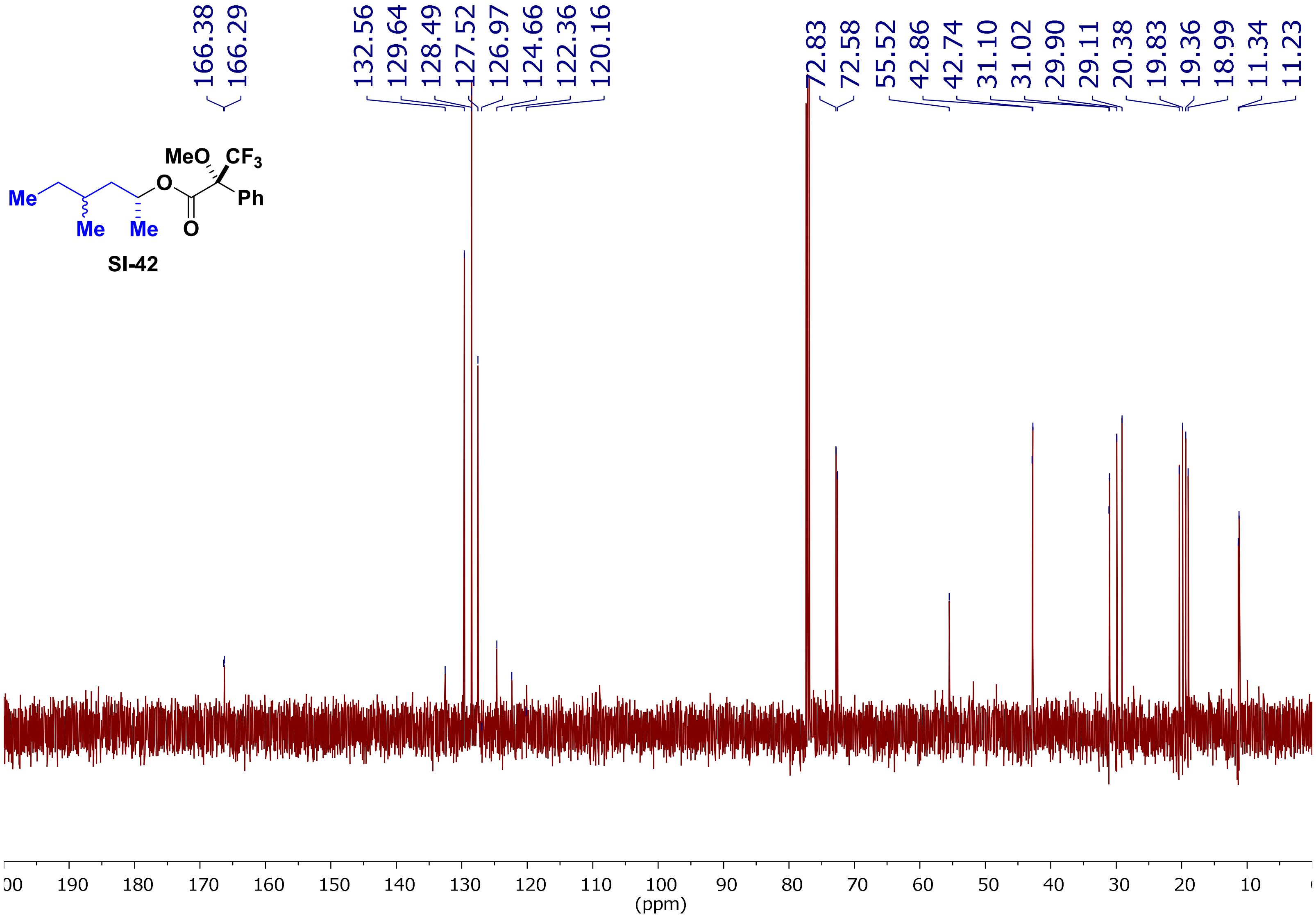

Supplementary Figure 243| ^{13}C-NMR spectrum (126 MHz, $CDCl_3$) for **SI-42**.

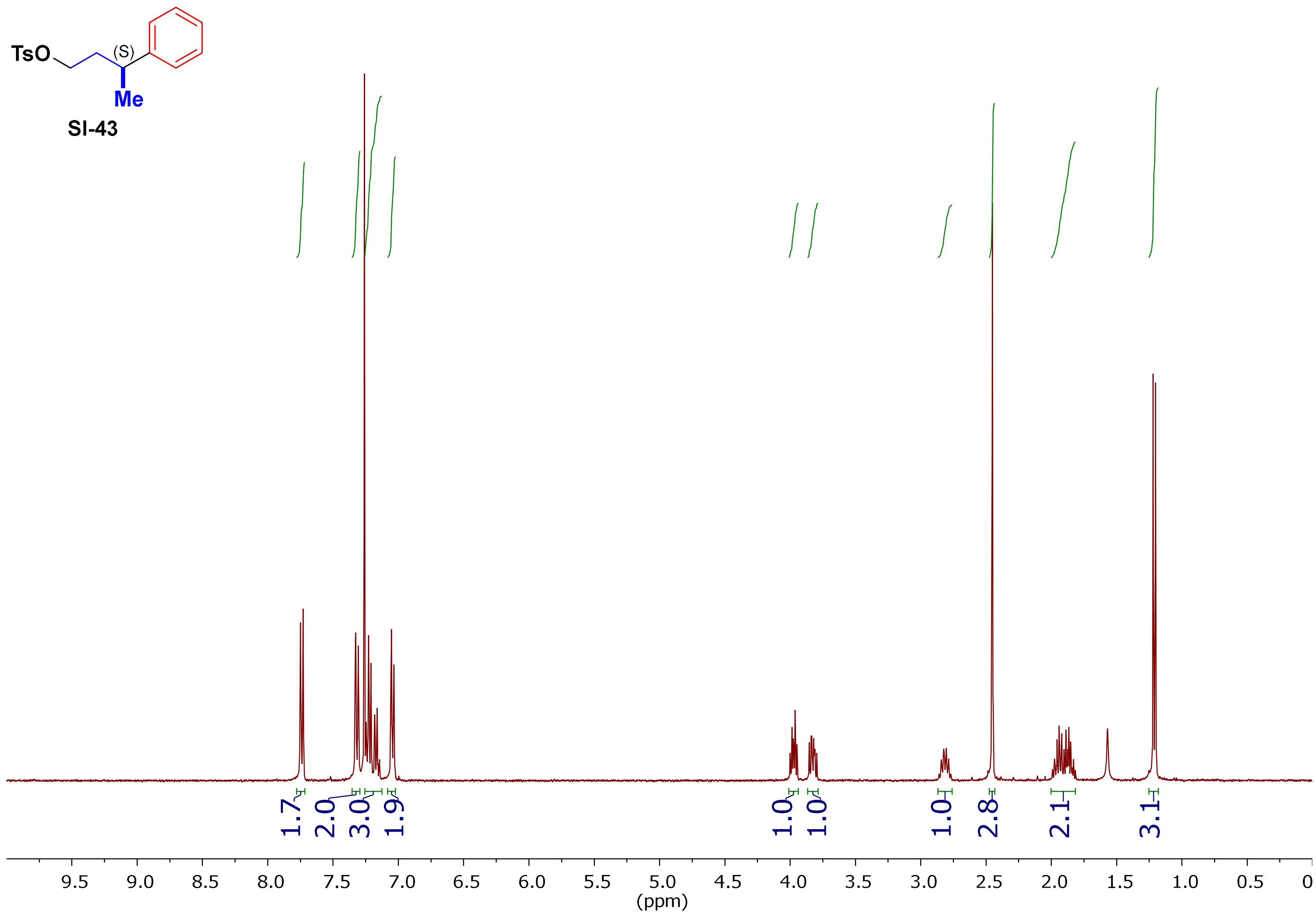

Supplementary Figure 244| ^{1}H-NMR spectrum (500 MHz, $CDCl_3$) for **SI-43**.

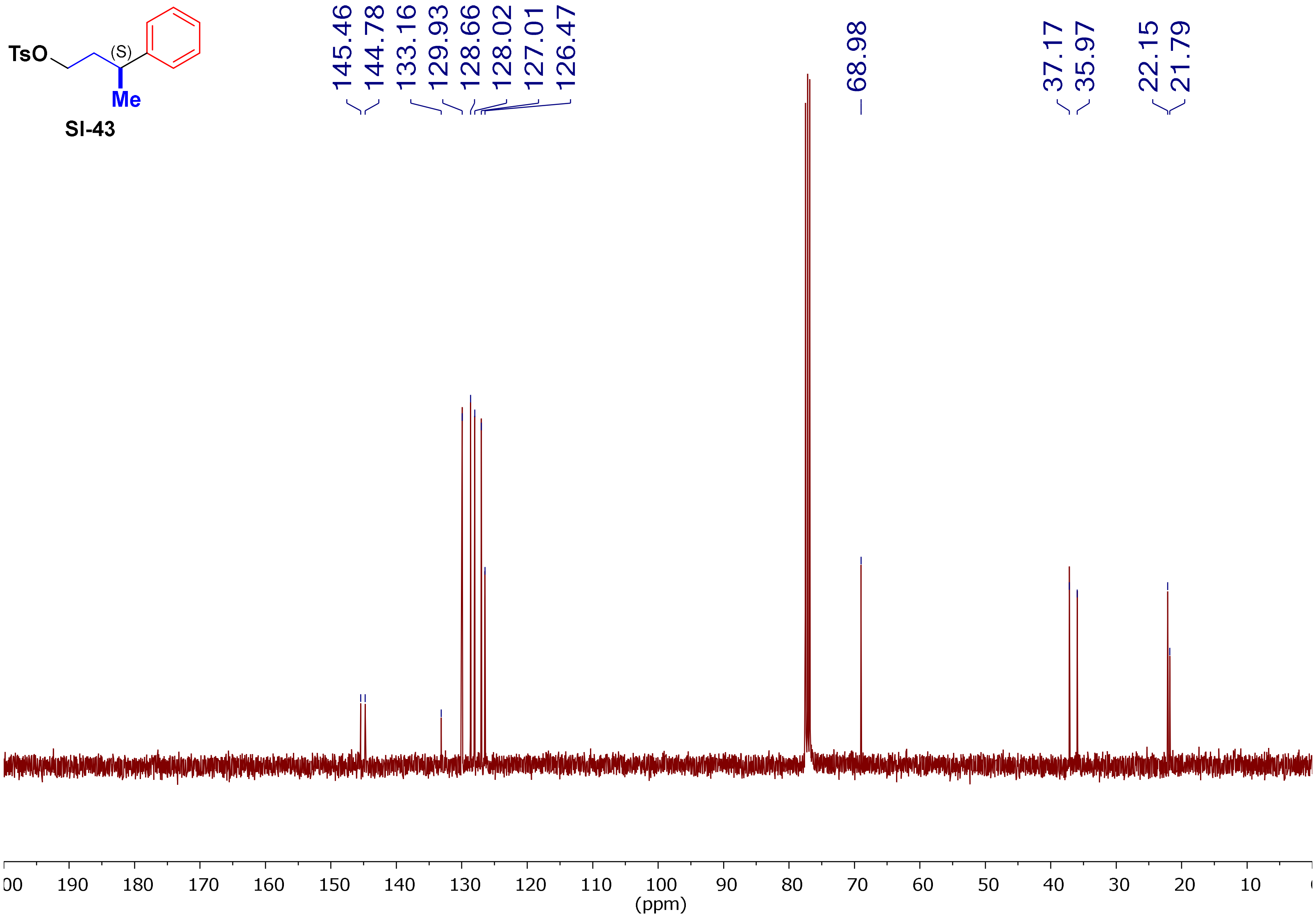

Supplementary Figure 245| ^{13}C-NMR spectrum (126 MHz, $CDCl_3$) for **SI-43**.

Supplementary References

1. Pangborn, A.B., Giardello, M.A., Grubbs, R.H., Rosen, R.K. & Timmers, F.J. Safe and Convenient Procedure for Solvent Purification. *Organometallics* **15,** 1518–1520 (1996).
2. Gottlieb, H.E., Kotlyar, V. & Nudelman, A. NMR Chemical Shifts of Common Laboratory Solvents as Trace Impurities. *Journal of Organic Chemistry* **62,** 7512–7515 (1997).
3. Li, J. & Burke, M.D. Pinene-derived iminodiacetic acid (PIDA). A powerful ligand for stereoselective synthesis and iterative cross-coupling of C(sp3) boronate building blocks. *Journal of the American Chemical Society* **133,** 13774–13777 (2011).
4. Anselmi, E., Abarbri, M., Duchêne, A., Langle-Lamandé, S. & Thibonnet, J. Efficient Synthesis of Substituted Styrenes and Biaryls (or Heteroaryls) with Regioselective Reactions of ortho-, meta-, and para-Bromobenzyl Bromide. *Synthesis* **44,** 2023–2040 (2012).
5. Fieser, L.F. & Fieser, M. *Reagents for organic synthesis.* 1st ed. (Wiley, New York, 1968).
6. Ziegler, C.B. & Heck, R.F. Palladium-catalyzed vinylic substitution with highly activated aryl halides. *Journal of Organic Chemistry* **43,** 2941–2946 (1978).
7. Moore, L.R. & Shaughnessy, K.H. Efficient aqueous-phase Heck and Suzuki couplings of aryl bromides using tri(4,6-dimethyl-3- sulfonatophenyl)phosphine trisodium salt (TXPTS). *Organic letters* **6,** 225–228 (2004).
8. Tang, W. *et al.* Formation of 2-Trifluoromethylphenyl Grignard Reagent via Magnesium−Halogen Exchange. Process Safety Evaluation and Concentration Effect. *Organic Process Research & Development* **13,** 1426–1430 (2009).
9. Le Gall, E., Aïssi, K., Lachaise, I. & Troupel, M. Synthesis of Symmetrical and Unsymmetrical Functionalized Arylphosphines from Chlorophosphines and Organozinc Reagents. *Synlett* **2006,** 954–956 (2006).
10. McCann, L.C., Hunter, H.N., Clyburne, J.A.C. & Organ, M.G. Higher-order zincates as transmetalators in alkyl-alkyl negishi cross-coupling. *Angewandte Chemie International Edition* **51,** 7024–7027 (2012).
11. McCann, L.C. & Organ, M.G. On the remarkably different role of salt in the cross-coupling of arylzincs from that seen with alkylzincs. *Angewandte Chemie International Edition* **53,** 4386–4389 (2014).
12. Fors, B.P. & Buchwald, S.L. Pd-catalyzed conversion of aryl chlorides, triflates, and nonaflates to nitroaromatics. *Journal of the American Chemical Society* **131,** 12898–12899 (2009).
13. Pena, M.A., Pérez Sestelo, J. & Sarandeses, L.A. New Synthetic Applications of Indium Organometallics in Cross-Coupling Reactions. *Synthesis* **2005,** 485–492 (2005).
14. Lennox, A.J.J. & Lloyd-Jones, G.C. Preparation of organotrifluoroborate salts. Precipitation-driven equilibrium under non-etching conditions. *Angewandte Chemie International Edition* **51,** 9385–9388 (2012).
15. Li, L., Zhao, S., Joshi-Pangu, A., Diane, M. & Biscoe, M.R. Stereospecific pd-catalyzed cross-coupling reactions of secondary alkylboron nucleophiles and aryl chlorides. *Journal of the American Chemical Society* **136,** 14027–14030 (2014).
16. Bruno, N.C., Tudge, M.T. & Buchwald, S.L. Design and Preparation of New Palladium Precatalysts for C-C and C-N Cross-Coupling Reactions. *Chemical science* **4,** 916–920 (2013).
17. Hong, K., Liu, X. & Morken, J.P. Simple access to elusive α-boryl carbanions and their alkylation. An umpolung construction for organic synthesis. *Journal of the American Chemical Society* **136,** 10581–10584 (2014).

18. Chowdhury, R. & Ghosh, S.K. Highly regio- and enantioselective organocatalytic conjugate addition of alkyl methyl ketones to a beta-silylmethylene malonate. *Organic letters* **11,** 3270–3273 (2009).
19. Roesner, S., Blair, D.J. & Aggarwal, V.K. Enantioselective installation of adjacent tertiary benzylic stereocentres using lithiation-borylation-protodeboronation methodology. Application to the synthesis of bifluranol and fluorohexestrol. *Chemical science* **6,** 3718–3723 (2015).
20. Mekala, S. & Hahn, R.C. A scalable, nonenzymatic synthesis of highly stereopure difunctional C4 secondary methyl linchpin synthons. *Journal of Organic Chemistry* **80,** 1610–1617 (2015).
21. Reiss, T. & Breit, B. Total synthesis of (+)-bourgeanic acid utilizing o-DPPB-directed allylic substitution. *Organic letters* **11,** 3286–3289 (2009).
22. Bergmann, J., Löfstedt, C., Ivanov, V.D. & Francke, W. Identification and Assignment of the Absolute Configuration of Biologically Active Methyl-Branched Ketones from Limnephilid Caddis Flies. *European Journal of Organic Chemistry* **2001,** 3175 (2001).
23. Jang, Y.-W. *et al.* Xylarinic acids A and B, new antifungal polypropionates from the fruiting body of Xylaria polymorpha. *The Journal of antibiotics* **60,** 696–699 (2007).
24. Cannillo, A. *et al.* Fast synthesis of complex enantiopure heterocyclic scaffolds by a tandem sequence of simple transformations on α-hydroxyaldehydes. *Chemistry: A European Journal* **19,** 9127–9131 (2013).
25. Buitrago, E., Tinnis, F. & Adolfsson, H. Efficient and Selective Hydrosilylation of Carbonyl Compounds Catalyzed by Iron Acetate and N -Hydroxyethylimidazolium Salts. *Advanced Synthesis & Catalysis* **354,** 217–222 (2012).
26. Duan, Z., Li, W. & Lei, A. Nickel-Catalyzed Reductive Cross-Coupling of Aryl Bromides with Alkyl Bromides. Et3N as the Terminal Reductant. *Organic letters* **18,** 4012–4015 (2016).
27. Wang, A., Fraga, R.P.A., Hörmann, E. & Pfaltz, A. Iridium-catalyzed asymmetric hydrogenation of unfunctionalized, trialkyl-substituted olefins. *Chemistry: An Asian journal* **6,** 599–606 (2011).
28. Bruker AXS, Inc.: Madison, Wisconsin, USA. *APEX2* (2014).
29. Krause, L., Herbst-Irmer, R., Sheldrick, G.M. & Stalke, D. Comparison of silver and molybdenum microfocus X-ray sources for single-crystal structure determination. *Journal of applied crystallography* **48,** 3–10 (2015).
30. Sheldrick, G.M. Crystal structure refinement with SHELXL. *Acta crystallographica. Section C: Structural chemistry* **71,** 3–8 (2015).
31. Spek, A.L. Structure validation in chemical crystallography. *Acta crystallographica. Section D: Biological crystallography* **65,** 148–155 (2009).
32. van der Sluis, P. & Spek, A.L. BYPASS. An effective method for the refinement of crystal structures containing disordered solvent regions. *Acta Crystallographica. Section A: Foundations of Crystallography* **46,** 194–201 (1990).
33. Flack, H.D. & Bernardinelli, G. Reporting and evaluating absolute-structure and absolute-configuration determinations. *Journal of applied crystallography* **33,** 1143–1148 (2000).
34. Flack, H.D. On enantiomorph-polarity estimation. *Acta Crystallographica. Section A: Foundations of Crystallography* **39,** 876–881 (1983).
35. Hooft, R.W.W., Straver, L.H. & Spek, A.L. Determination of absolute structure using Bayesian statistics on Bijvoet differences. *Journal of applied crystallography* **41,** 96–103 (2008).
